# Reactivity Factors in Catalytic Methanogenesis and Their Tuning upon Coenzyme F430 Biosynthesis

**DOI:** 10.1021/jacs.3c00469

**Published:** 2023-04-12

**Authors:** Priyam Bharadwaz, Mauricio Maldonado-Domínguez, Jakub Chalupský, Martin Srnec

**Affiliations:** J. Heyrovský Institute of Physical Chemistry, Czech Academy of Sciences, Prague 182 23, Czech Republic

## Abstract

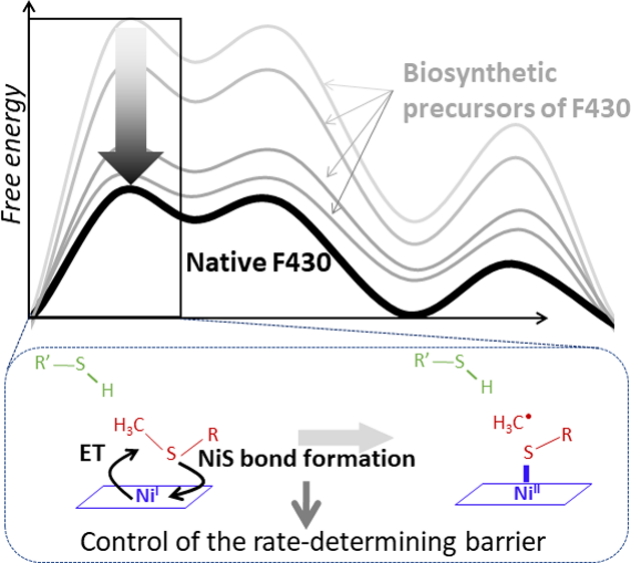

Methyl-coenzyme M reductase, responsible for the biological production of methane by catalyzing the reaction between coenzymes B (CoBS-H) and M (H_3_C-SCoM), hosts in its core an F430 cofactor with the low-valent Ni^I^ ion. The critical methanogenic step involves F430-assisted reductive cleavage of the H_3_C–S bond in coenzyme M, yielding the transient CH_3_ radical capable of hydrogen atom abstraction from the S–H bond in coenzyme B. Here, we computationally explored whether and why F430 is unique for methanogenesis in comparison to four identified precursors formed consecutively during its biosynthesis. Indeed, all precursors are less proficient than the native F430, and catalytic competence improves at each biosynthetic step toward F430. Against the expectation that F430 is tuned to be the strongest possible reductant to expedite the rate-determining reductive cleavage of H_3_C–S by Ni^I^, we discovered the opposite. The unfavorable increase in reduction potential along the F430 biosynthetic pathway is outweighed by strengthening of the Ni–S bond formed upon reductive cleavage of the H_3_C–S bond. We found that F430 is the weakest electron donor, compared to its precursors, giving rise to the most covalent Ni–S bond, which stabilizes the transition state and hence reduces the rate-determining barrier. In addition, the transition state displays high pro-reactive motion of the transient CH_3_ fragment toward the H–S bond, superior to its biosynthetic ancestors and likely preventing the formation of a deleterious radical intermediate. Thus, we show a plausible view of how the evolutionary driving force shaped the biocatalytic proficiency of F430 toward CH_4_ formation.

## Introduction

Methane is an important source of energy due to its heat of combustion, the highest among carbon-based fuels.^1,2^ Globally, 90–95% of methane is formed by methanogenic archaea in anoxic environments from CO_2_ and H_2_, acetate, methylamines, and methanol.^3,4^ In such way, nearly 1 billion tons of methane are produced every year.^5^

**Scheme 1:**
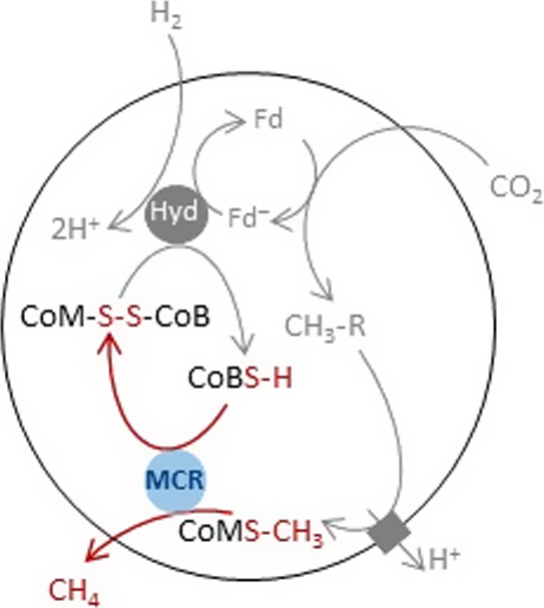
MCR Catalyzes the Reaction between Coenzymes M and B The reaction is highlighted in red and placed in the context of a simplified respiratory chain in archea, in which CO_2_ is reduced by H_2_ to propel the pumping of protons through the membrane and drive the synthesis of energy-rich ATP molecules. The acronyms Hyd, Fd, and Fd^–^ stand for hydrogenase and oxidized and reduced ferredoxin, respectively. The scheme is adapted from ref (13).

Methyl-coenzyme M reductase (MCR) is essential for catalysis of the terminal and rate-determining step in biological methanogenesis. Each of its two identical active sites hosts a nickel-containing cofactor F430, structurally similar to porphyrin, chlorophyll, and vitamin B12.^6−9^ This cofactor promotes the reaction between two co-substrates—methyl donor [methyl-coenzyme M: H_3_C–SCoM] and hydrogen donor [coenzyme B: CoB-SH], yielding methane and heterodisulfide CoMS–SCoB (Scheme 1).^10,11^ Note that MCR is also capable of catalyzing the reverse process as reported for anaerobic methanotrophic sulfate, nitrate, or Fe^III^-reducing bacteria.^8,10−12^

**Figure 1:**
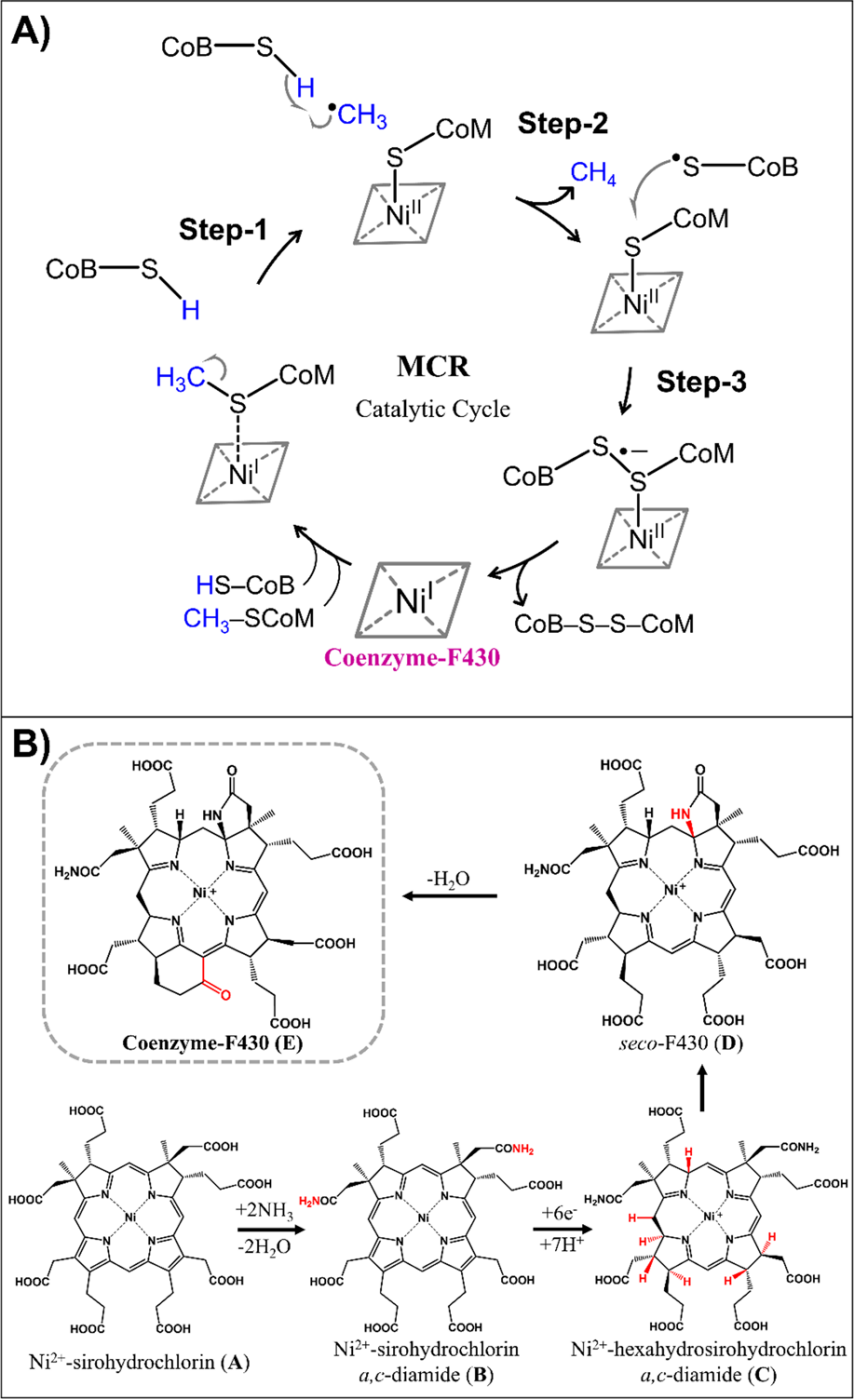
(A) Canonical catalytic cycle of the MCR (refs (21) and (24)). (B) Biosynthesis of the native cofactor F430 comprises four consecutive Ni-containing intermediates, as reported in ref (25). Labels **A**, **B**, **C**, **D**, and **E** are used for the cluster models from Figure 2 anchoring these biosynthetic precursors.

To date, a number of studies have been carried out toward understanding the mechanistic details for methane production by MCR.^14−22^ The current consensus on the reaction mechanism of MCR, which was first computationally proposed by Siegbahn and co-workers^21,23^ and later supported experimentally by Ragsdale and co-workers,^24^ is summarized in Figure 1A. Importantly, the main part of the catalytic cycle consists of three subsequent steps: (i) homolytic cleavage of the H_3_C–S_CoM_ bond with the concomitant oxidation of the nickel center from +I to +II and the formation of a bond between Ni^II^ and SCoM, followed by (ii) attack of the transient methyl radical to the H–S_CoB_ bond, which releases CH_4_ and the substrate ^·^S_CoB_ radical, the latter of which subsequently (iii) recombines with the Ni^II^-bound SCoM to form a disulfide product. These three steps are labeled in Figure 1A as **step 1**, **step 2**, and **step 3**, and this labeling will be used further in the text.

Recently, Warren and co-workers elucidated the biosynthetic pathway of F430,^25^ where the late stage comprises four enzymatically controlled steps in which the porphyrin-like skeleton is gradually modified including chelation, amidation, reduction by six electrons with addition of seven protons, lactamization, and closure of a propionate side chain coupled to water extrusion. All experimentally determined Ni-anchoring biosynthetic precursors of F430 are shown in Figure 1B.

Here, driven by curiosity about whether and how the evolutionary force forming such a complex and energy-demanding pathway lies in optimizing reactivity toward the CH_4_ formation (and its oxidation in the reverse process), we computationally investigated energetics and dissected key reactivity factors contributing to the consensual mechanism of methane production by the native F430 cofactor and its four biosynthetic precursors from Figure 1B.

## Methodology and Computational Details

### Structural Models

**Figure 2:**
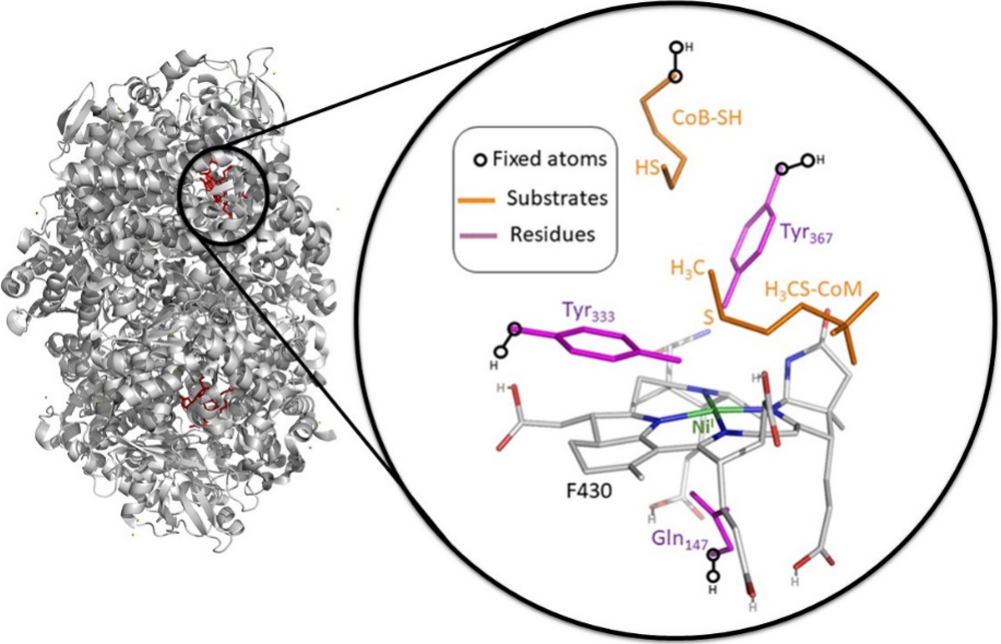
Cluster model of the MCR active site constructed from the X-ray crystal structure 1HBN. Hydrogens are omitted for clarity. Carboxylates in F430 are protonated (as schematically depicted), and the residues and the CoB-SH are truncated and capped by H atoms. In the preparatory stage, the capped C–H bonds were aligned and optimized along the original (crystallographic) C–C axes, while all non-hydrogen atoms were kept fixed. In all subsequent optimizations, only these capping H atoms along with the adjacent C atoms were kept fixed as indicated in the figure. The depicted model with the native F430 is labeled in the text as model **E**. Other models with the non-native cofactors corresponding to biosynthetic precursors from Figure 1B are labeled **A**, **B**, **C**, and **D**. All the cluster models (**A**–**E**) and their overlay are shown in Figure S1.

The cluster model of the active site of MCR, constructed from the crystal structure with PDB code 1HBN,^17^ consists of 190 atoms including (i) the Ni-containing F430 coenzyme; (ii) the two co-substrates CH_3_S-CoM and CoB-SH; and (iii) three residues, Tyr333, Tyr367, and Gln147 (Figure 2). If not stated otherwise, the carboxylate groups of the coenzyme are protonated, giving the MCR model the total charge of −1. In analogy, the cluster models of the fictitious MCR-like active sites anchoring various F430-related complexes (non-native coenzymes) were derived from the MCR native model. Such *non-native* coenzymes were identified as intermediates of the biosynthetic pathway of the native F430 (Figure 1B).^25^ The corresponding models are further labeled as **A**, **B**, **C**, **D**, and **E**, where the latest stands for the F430-containing MCR model, while **A**, **B**, **C**, and **D** are the active-site models anchoring biosynthetic precursors of F430: Ni^II^-sirohydrochlorin (**A**), Ni^II^-sirohydrochlorin *a,c*-diamide (**B**), Ni^II^-hexahydrosirohydrochlorin *a,c*-diamide (**C**), and *seco*-F430 (**D**). **A**/**B** and **C**/**D** models have the charge of −2 and −1, respectively. All the cluster models and their overlay are shown in Figure S1A,B. Comparison of the referential PDB geometry and its optimized form is presented in Figure S1C, supporting the truncation scheme employed throughout this work.

### Density Functional Theory Calculations

All of the structures were optimized at the B3LYP(D3)/BS1/CPCM(ε_r_ = 4) level of theory, i.e., we employed the B3LYP^26^ functional with the empirical correction to the dispersion effects using the original Grimme’s D3 damping function^27^ and in combination with the hybrid basis set BS1, which includes the LANL2TZ ECP basis set for Ni,^28^ 6-311G* for key atoms involved in reactivity (the SCH_3_ and SH groups of the H_3_CS-CoM and truncated CoB-SH substrates, respectively), and 6-31G* for the rest,^29^ while the conductor-like polarizable continuum model (CPCM)^30^ with a dielectric constant ε_r_ = 4 (explicit parameters for the CPCM solvent model are provided in Table S1) was used to represent the protein environment. Gibbs free energies were evaluated:
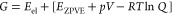
1where *E*_el_ are the B3LYP(D3)/BS2/CPCM single-point energies on top of the optimized geometries, calculated using the larger basis set BS2: LANL2TZ+ ECP for Ni and 6-311++G** for the rest; the [*E*_ZPVE_ + *pV* – *RT* ln *Q*] term includes the thermal enthalpic and entropic contributions of the solute energy with *E*_ZPVE_ and *Q* corresponding to zero-point vibrational energy and the molecular partition function, respectively, obtained from B3LYP(D3)/BS1/CPCM. Frequency calculations were performed with the rigid rotor/harmonic oscillator approximation (for *p* = 1 bar, *T* = 298 K). In all cases, the vibrational frequencies associated to the eight frozen atoms (Figure 2) were projected out from the hessian, yielding the consistent number of degrees of freedom for minima (3*n* – 24) and for transition states 3*n* – 25, where *n* is the total number of atoms in the cluster model. Only these degrees of freedom were included when calculating entropic and enthalpic contributions. All calculations were carried out using Gaussian 16.^31^

Atomic charges and delocalization indices were obtained by integration of the DFT-optimized electron density using the atoms-in-molecules (AIM) theory as implemented in the AIMAll program.^32^ Atomic basins were integrated using the Proaim method,^32^ where a “Fine” interatomic surface mesh, an outer angular integration quadrature of 7200 grid points, and a maximum integration radius of 13.0 Bohr were used for all atoms. Electrostatic potential (ESP) maps were obtained by probing the 0.001 isodensity surface with a point charge. All AIM atomic charges are included in the Supporting Information.

### Redox Potentials and Validation of the Methodology

The critical step in the MCR catalytic cycle includes the reductive cleavage of the H_3_C–SCoM bond with the concomitant oxidation of Ni^I^ to Ni^II^ (Figure 1). Thus, the reduction potential of the metal center in the F430 cofactor must be key for successful C–S cleavage, and its accurate evaluation is a prerequisite for a reliable description of reaction energetics of such a critical catalytic step. The reduction (redox) potentials (*E*°, in V) are calculated as follows:
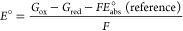
2where *G*_ox_ and *G*_red_ are the Gibbs free energies of the oxidized and reduced forms of the structural models containing Ni^II^ and Ni^I^, respectively, and *E*_abs_^°^ (reference) is the absolute potential of a reference electrode. *F* is the Faraday constant. If not stated otherwise, the absolute potential of a normal hydrogen electrode (NHE) in water with the value of *E*_NHE_^°^ = 4.28 eV was used.^33^

To validate the computational methodology for calculation of Gibbs free energies from eq 2, we calculated reduction potentials of three structurally well-defined synthetic complexes, for which the experimental data are available.^34−37^ The calculated potentials presented in Figure S2 are in all cases in good agreement with the experiments (deviation of calculated reduction potentials from experimental data are provided in Figure S2). Note that the redox-active molecular orbital in Ni^I/II^ is the in-plane *d*_*x*^2^ – *y*^2^_-based orbital. Orbital rendering and visualization were achieved with the aid of Charmol.^38^

### Multiconfigurational Calculations

The presented results were calculated using the ANO-RCC-DZP^39^ basis set, where Coulomb and exchange two-electron integrals were approximated by the resolution of identity (RIJK) with our own auxiliary basis set generated for ANO-RCC-DZP by a procedure similar to ref (40). Scalar relativistic effects were accounted for via the second-order Douglas–Kroll–Hess (DKH2) approximation.^41^

The complete active space self-consistent field (CASSCF)^42^ calculations were based on an active space composed of two Ni 3d orbitals (*d*_*z*^2^_ and *d*_*x*^2^ – *y*^2^_), the S 3p_*z*_ orbital of the Ni–S bond, the N 2p_*x*_ and 2p_*y*_ orbitals of the Ni–N bonds, and the methyl singly occupied orbital. State-specific CASSCF calculations were performed for the doublet ground state on top of DFT-optimized geometries. All calculations were carried out with the in-house ORZ program developed in the group of Yanai et al.^43^

### Kinetic Energy Distribution (KED) Analysis

Kinetic energy of the *i*th atom ⟨*T_i_*⟩ in the molecular systems of *n* atoms is derived from kinetic energies of the 3*n* normal modes ⟨*T*_α_⟩:
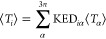
3where KED_*i*α_ is the fraction of kinetic energy associated with the *i*th atom in the mode α with real (or imaginary) frequency. Note the number of 3*n* modes also includes translational and rotational degrees of freedom. KED values are readily obtainable from cartesian atomic displacements; in this study, these are obtained from the B3LYP(D3)/BS1/CPCM frequency calculations as
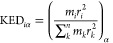
4ranging between 0 and 1; *r_i_* and *m_i_* are the displacement and the mass of the *i*th atom, respectively (*k* runs over all *n* atoms). The complete theory is provided in ref (44), while some of its applications are refs (45) and (46). For purposes of the presented study, only KED within the reactive mode with one imaginary frequency was analyzed and correlated with other properties. Mapping of KED values on molecular structures was carried out with the aid of Charmol.^38^

## Results and Discussion

### Redox Potentials: Models with the Native F430 vs Biosynthetic Precursors of F430

The Ni center present in F430 switches between the +I and +II oxidation states during the catalytic process (Figure 1A). This raises the question of whether the redox activity of F430, controlled by its reduction potential *E*^°^, is the key variable that makes F430 a unique catalyst for methane production compared to all four F430 biosynthetic precursors presented in Figure 1B. Naturally, such hypothesis is only viable if the chemical modifications made on the macrocyclic ring during the biosynthetic pathway of F430 translate into substantial changes of *E*^°^.

Table 1 summarizes the results for enzymatic active-site models **A**–**E**, which host the F430 cofactor (model **E**) and its biosynthetic precursors (models **A**, **B**, **C**, and **D**), respectively. As seen, *E*^°^ varies significantly across the series. The active-site models containing early-stage biosynthetic precursors are very strong reductants, while the native active site with F430 is the strongest oxidant across the series (the span of *E*° is almost ∼1.5 V). We also note in passing that the oxidized state Ni^II^ in **A**–**D** resides in the triplet state with two unpaired electrons in *d*_*x*^2^ – *y*^2^_ and *d*_*z*^2^_, while model **E** adopts the singlet state (where the *d*_*x*^2^ – *y*^2^_ orbital remains unoccupied) with the *S*_ox_ = 1 state lying only ∼1 kcal mol^–1^ above the ground state. Interestingly, the splitting of the singlet/triplet spin states of the Ni^II^ center follows the same trend as the reduction potential (cf. Table 1), both indicating that the stability of the triplet state of Ni^II^ decreases in the order **B** > **A** > **C** > **D** > **E**. The reduced Ni^I^ form displayed a doublet ground spin state in all cases, with an unpaired electron in the *d*_*x*^2^ – *y*^2^_ orbital.

**Table 1:** Redox Potentials for the Couple Ni^II^/Ni^I^ in All Cluster Models **A**–**E** from Figure 2 (and Figure S1) Calculated Using eq 2 and Referenced to the NHE^a^

cluster model	*S*_ox_/*S*_red_	*E*°_calc_ (V)	oxidized form: singlet/triplet gap (kcal mol^–1^)	reduced form: doublet/quartet gap (kcal mol^–1^)
**A**		–1.77	5.8	12.8
**B**		–1.95	6.7	12.9
**C**		–1.11	4.9	17.2
**D**		–0.88	1.8	26.9
**E**		–0.53	0.9	20.0

a The ground spin states for both oxidized and reduced forms along with the spin-state energetics are also shown. Note that the oxidized form of the Ni center in the triplet state in **A–E** is axially coordinated by the glutamine residue, Gln147 (the reduced form and the oxidized form in the singlet state have no axial ligation to Ni).

To sum this section, we conclude that biosynthetic modifications of the macrocyclic ring of the Ni complex have a sizable impact on the redox activity of Ni, and hence, its contribution to reactivity will be addressed in the following sections.

### Reaction Energetics: Models with the Native F430 vs Biosynthetic Precursors of F430

**Figure 3:**
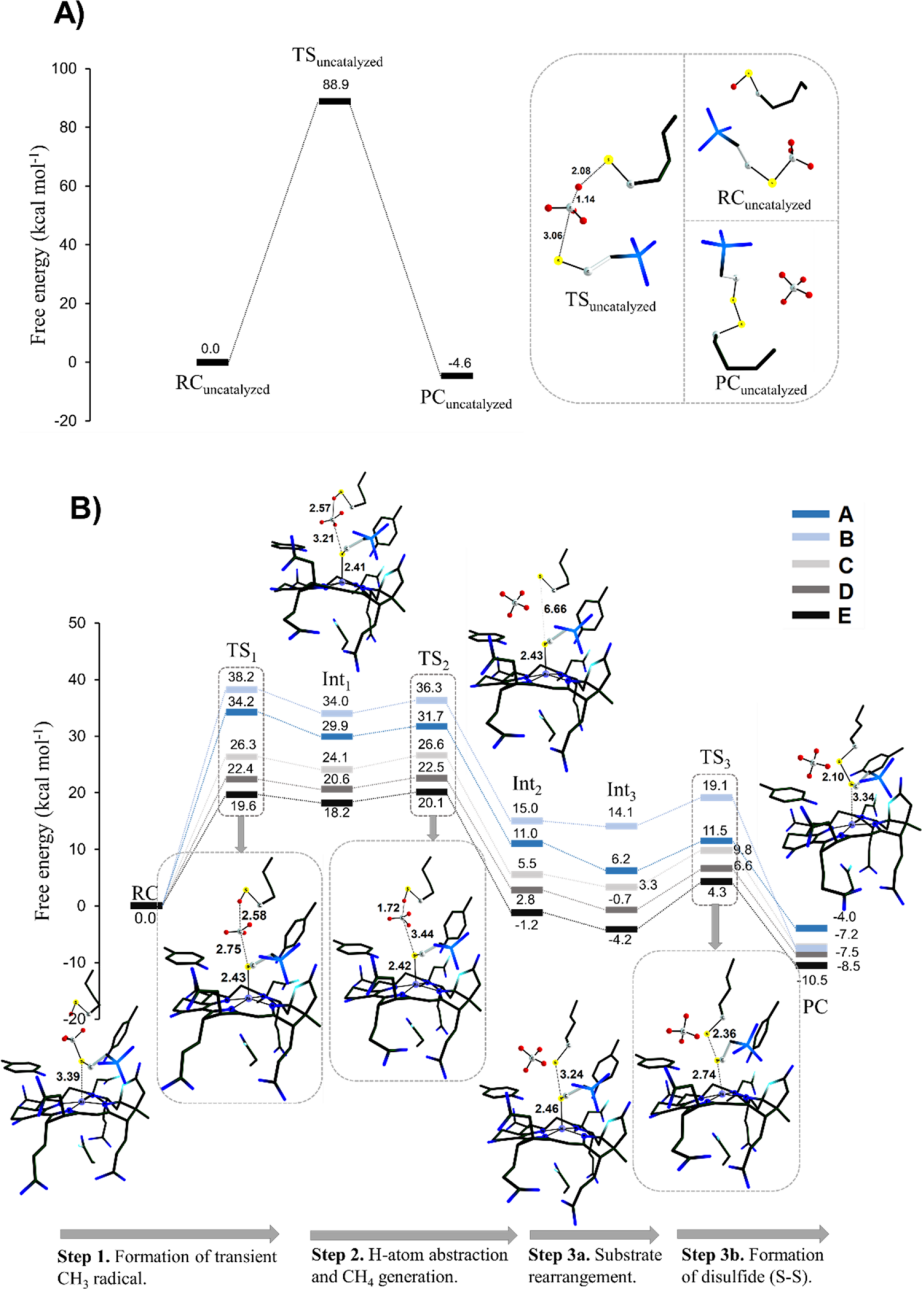
(A) Free-energy profile of uncatalyzed one-step reaction summarized in Scheme 1 along with the structures and critical distances (in Å). (B) Free-energy profile for catalyzed methanogenesis within enzyme-like models (**E** representing MCR with the native F430; **A**–**D** hosting biosynthetic precursors of F430). For the sake of clarity, all the critical structures along the catalytic reaction coordinate are displayed for model **E**, and only key hydrogen atoms are visualized. The potential energies and enthalpies of all key geometries are given in Table S2.

As a referential system, we first calculated the energetics of the uncatalyzed reaction between the CH_3_S-CoM and CoB-SH substrates, i.e., in the absence of a Ni-macrocycle complex, while imposing a homogeneous water-like surrounding through the continuum solvation model with ε_r_ = 80.0. The corresponding reaction mechanism and its energetics are described in Figure 3A and in detail in Figure S3, where the intrinsic reaction coordinate (IRC) with the key structures is shown. From this IRC, the uncatalyzed process begins with the cleavage of the S–CH_3_ bond in coenzyme M, which further initiates the cleavage of the S–H bond in coenzyme B with the concomitant formation of CH_4_. All these three events occur in one single-step process with a free-energy barrier (Δ*G*^≠^) of ∼89 kcal mol^–1^, with S–S bond formation between coenzymes M and B occurring in a second step characterized by essentially no barrier. The free energy of reaction is Δ*G*_0_ = −4.6 kcal mol^–1^. These results clearly show that such a direct reaction is unfeasible. *Why and how does this change when MCR participates in the reaction?* Here, we take advantage of the consensual mechanism for the catalytic CH_4_ formation from Figure 1A and evaluate its energetics for five active-site models **A**–**E**, one with the native F430 and four with its biosynthetic precursors from Figure 1B. In all cases, the reaction mechanism consists of three consecutive steps, each associated with its own barrier (Figure 3B).

**Figure 4:**
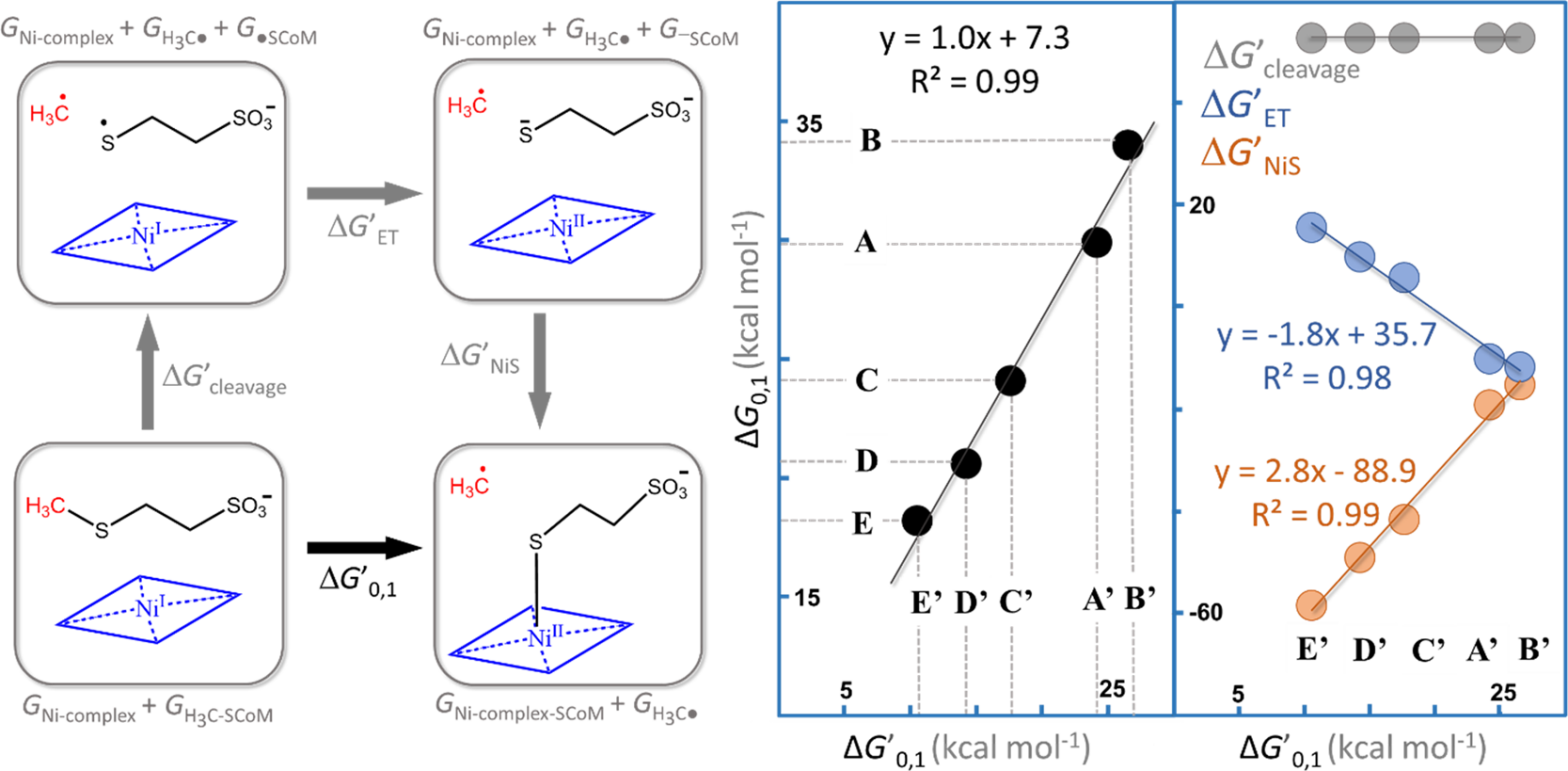
Thermodynamic cycle of the first catalytic step involving the formation of the transient methyl radical coupled with one-electron oxidation of the Ni^I^ center and Ni-thiolate bond formation. The whole process is dissected into three steps: (i) the homolytic cleavage of the S–CH_3_ bond (Δ*G*′_cleavage_), (ii) electron transfer from the Ni^I^ center to the S-radical (Δ*G*′_ET_); and (iii) the Ni–S bond formation (Δ*G*′_NiS_). Note that Δ*G*′ values were calculated from Gibbs free energies of individual CPCM(ε_r_ = 4.0)-solvated species, as indicated in the figure and using eq 1; the sum of three sequential Δ*G*’s steps equals to Δ*G*′_0,**1**_, which is analogous to the free energy of reaction Δ*G*_0,**1**_ from Figure 3B. The labels **A′**, **B′**, **C′**, **D′**, and **E′** are used in parallel to labels for the full models **A–E**; the “prime” symbol refers to the fact that the model consists of individual (infinitely separated) moieties such as the Ni^I/II^-complex, H_3_C–SCoM, ^–/^^·^SCoM, CH_3_^·^, and the Ni^II^–SCoM complex. Of note, the individual Ni complexes from the upper-right corner of the cycle are calculated in the singlet ground spin state (details in Tables S4A and S5), while the Ni^II^–SCoM complexes from the lower-right corner of the cycle are obtained in the triplet ground state, which is in line with the local triplet state of Ni in the Int_**1**_ structures of models **A**–**E** from Figure 3. Importantly, if the triplet state for the individual Ni^II^-complexes from the upper-right corner is considered, the correlation patterns seen in the rightmost graph in this figure are preserved (cf. Figure S8 and Table S4B): the change in Δ*G*′_NiS_ dominates the change in Δ*G*′_0,**1**_ and outcompetes the unfavorable change in Δ*G*′_ET_ in going from **B′**/**A′** to **E′**.

The first barrier Δ*G*_**1**_^≠^ (associated with **step 1** from Figure 4, vide infra) involves formation of the transient methyl radical and must be critical for catalysis due to the direct participation of the redox-active Ni center of the cofactor via Ni^I^ oxidation and Ni^II^-thiolate bond formation. Such chemical events strongly suggest that Δ*G*_**1**_^≠^ should be sensitive to modifications of the macrocyclic ring. Indeed, the calculations show that Δ*G*_**1**_^≠^ varies significantly in going from model **A** to **E** with the lowest barrier for **E** containing the native F430 cofactor (∼20 kcal mol^–1^; structures of all minima and transition states appearing along the pathway in Figure 3B are depicted in Figure S4). In addition, Δ*G*_**1**_^≠^ appears to decrease almost monotonously in the transition from **A** to **E** (**B** > **A** > **C** > **D** > **E**), suggesting that all chemical steps along biosynthesis of F430 are evolutionary designed to make the CH_4_ formation feasible. Importantly, the change of Δ*G*_**1**_^≠^ correlates with the change of free energy of reaction of **step 1** (Δ*G*_0,**1**_) in the ratio ∼1:1 (Figure S5). Such one-to-one correlation is the consequence of geometric- and electronic-structure similarity of the transition state TS_**1**_ with the first methyl radical-containing intermediate Int_**1**_ (i.e., TS_**1**_ is late along the reaction coordinate), which is further reflected by a small energetic difference between TS_**1**_ and Int_**1**_ (*G*_TS**1**_ – *G*_Int**1**_ < 5 kcal mol^–1^; reaction **step 1** is strongly endergonic in all **A**–**E**; Figure 3B). This implies that Δ*G*_**1**_^≠^ and its change across **A**–**E** must be controlled thermodynamically. In all cases, along the reaction coordinate of **step 1**, the oxidized Ni adopts the local triplet state (*S*_Ni^II^_ = 1) that couples antiferromagnetically to the transient CH_3_ radical (cf., spin densities in Table S3).

We note that the carboxylate groups located on the periphery of the macrocyclic cofactors in the set **A**–**E** are protonated in our study (Figure 2), while these groups are likely charged in the native MCR enzyme. Thus, we evaluated the electrostatic effect of these charged groups on Δ*G*_**1**_^≠^ and Δ*G*_0,**1**_ and their evolution across **A**–**E** and we found that both Δ*G*_**1**_^≠^ and Δ*G*_0,**1**_ are systematically downshifted by ∼5 kcal mol^–1^ (Figure S6); Δ*G*_**1**_^≠^ for the MCR model **E** reaches 14 kcal mol^–1^, which is close to the experimental value of 13.2 kcal mol^–1^.^24^ Thus, it seems that the charged carboxylate groups on the corphinoid periphery in F430 play an important role in reducing the rate-determining barrier and possibly in anchoring the macrocycle in MCR. Nevertheless, our results suggest that they do not contribute to differences in reactivity between F430 and its precursors. Also, we note that the distortion of tetrapyrrole macrocycles is found in nature, as is the case of *cytochrome c*, to maximize the efficiency of this kind of cofactors through the fine-tuning of their electronic properties by protein-imposed geometry constraints.^47^ In the case of cofactor F430, the native geometry as obtained by XRD in ref (17) deviates minimally (RMSD = 1.0 Å) from the DFT-optimized cluster model **E**, suggesting that the maturation process suffices to reach catalytic efficiency and cofactor distortion was not necessary during the evolutionary process, leading to F430.

In the case of **step 2** from Figure 3B, the transient methyl radical abstracts a hydrogen atom from the coenzyme HS-CoB. The energetics of this step is practically independent of the chemical character of the cofactor: the barrier Δ*G*_**2**_^≠^ in all cases is only ∼2 kcal mol^–1^ and the reaction free energy associated with this step Δ*G*_0,**2**_ is exergonic (≈−19 kcal mol^–1^). Regarding the possible formation and fate of a CH_3_ radical species, we carried out an analysis of KED (ranging from 0 to 1) of the reactive mode of transition state in **step 1** (TS_**1**_). The analysis reveals that most of the kinetic energy is concentrated in the motion of the nascent methyl radical (cf. KED_CH_3__ in all **A**–**E** cases exceeds 0.78; Figure S7), with the highest KED_CH_3__ = 0.88 calculated for the native **E**. This result suggests that the shallow Int_**1**_ state can be bypassed by a ballistic trajectory of CH_3_ toward the key H–S bond, leading to direct methane production after passing TS_**1**_. Such dynamically controlled reactivity goes beyond the traditional transition state theory description, and the KED analysis was proposed by us as an applicable tool when nonequilibrium reactions are suspected,^43^ as is potentially the case of the CH_3_S-CoM cleavage/CoB-SH oxidation sequence. Very high pro-reactive motion of the CH_3_ fragment toward the H–S bond would prevent accumulation of the radical intermediate, potentially dangerous for the protein structure and its function. In this context, it is also worth stressing that Siegbahn, using a similar computational protocol, reported a single barrier for methane production with TS_**1**_ followed by a barrierless **step 2**.^21^ In either mechanism, the methyl radical would be very transient.

In **step 3**, the CoBS^·^ radical combines with the Ni^II^-bound thiolate of CoM to generate a disulfide anion radical (Figure 3B). Notably, we recognize two distinct stages of **step 3**: (i) substrate translocation to approach SCoM (**step 3a**) and (ii) the S–S bond formation (**step 3b**). Concerning **step 3a**, the translocation of the CoBS-radical closer to SCoM in going from Int_**2**_ to Int_**3**_ is exergonic in all models **A**–**E**. Formation of the disulfidic bond in **step 3b** requires overcoming a barrier, which is found to be the highest (Δ*G*_**3**_^≠^ = 8.5 kcal mol^–1^) for model **E**, with the native cofactor F430, and the lowest for **B** (5.0 kcal mol^–1^). While Δ*G*_**3**_^≠^ is quite variable in the series **A**–**E**, it stays lower in energy as compared to the rate-determining Δ*G*_**1**_^≠^.

Overall, relative to the uncatalyzed reaction (Figure 3A), the participation of any of the studied Ni macrocycles enormously reduces the highest barrier of the reaction highlighted in red in Scheme 1. However, the early-stage biosynthetic precursors anchored in models **A** and **B** still possess very high barriers (∼38 and ∼34 kcal mol^–1^ for **B** and **A**, respectively) to catalyze the reaction, while two late-stage precursors in models **C** and **D** become better but still inefficient catalysts, rendering their respective rate-determining barriers roughly 3 and 7 kcal mol^–1^ higher than the barrier displayed by model **E** with the native F430.

### Reactivity Factor Analyses of the Key Reaction **Step 1**

To elucidate the factors contributing to energy differences of key catalytic **step 1** in the series **A**–**E**, we decomposed this step into three elementary events and constructed the thermodynamic cycle shown in Figure 4. The first elementary step is homolytic cleavage of the substrate S–CH_3_ bond and is energetically independent of the chemical character of the Ni complex (Δ*G*′_cleavage_). In contrast, the second and third events are dependent on the properties of the Ni complex because they correspond to Ni^I^-to-thiyl electron transfer (ET) and Ni^II^-thiolate bond formation (Δ*G*′_ET_ and Δ*G*′_NiS_), respectively. Of note, the Δ*G*′_cleavage_, Δ*G*′_ET_, and Δ*G*′_NiS_ terms in Figure 4 are obtained using free energies of individual moieties (at infinite separation) such as the *isolated* Ni complex (in the absence of amino residues and coenzymes), *isolated* coenzyme M, etc., as indicated in the figure. The thermodynamic dissection of **step 1** into three terms is sensible, as evidenced by the correlation between the free energy of reaction Δ*G*_0,**1**_ from Figure 3B and the sum of the three thermodynamic terms Δ*G*′_0,**1**_ (= Δ*G*′_cleavage_ + Δ*G*′_ET_ + Δ*G*′_NiS_), where the correlation slope reaches nearly the ideal value of 1 (Figure 4, *left graph*). The detailed inspection of the two decisive individual contributions Δ*G*′_ET_ and Δ*G*′_NiS_ is provided in the following sections.

### Ni^I/II^ Redox Potential and Its Correlation with Reactivity in **Step 1**

The change of Δ*G*′_ET_ is given by the difference in reduction potentials of Ni complexes (cf. Figure 4 and Tables S4 and S5). Importantly, *E°* increases as F430 biosynthetic precursors gradually become more similar to F430, suggesting an appealing possibility that the reduction potential of the Ni center is responsible for the variability of the free energies of reaction and activation in **step 1**. From Figure 4 (*right graph*), Δ*G*′_ET_ correlates nicely with Δ*G*′_0,**1**_ and thus with the actual free energy of reaction Δ*G*_0,**1**_. However, the obtained correlation is counter-intuitive: an easier oxidation of Ni^I^ is linked to a more endergonic reaction (with a more positive Δ*G*_0,**1**_). In other words, the reductive cleavage of the S–CH_3_ bond facilitated by the Ni cofactor is the most favorable in the native active-site **E**, despite the fact that F430 is the least capable of donating electrons among Ni complexes. This implies that the effect of *E*° on reaction energetics must be outweighed by another contribution, which we reveal in the upcoming analysis.

### Ni–S Bond Formation and Its Correlation with Reactivity in **Step 1**

The factor that dominates over the effect of *E*° is the free energy of bond formation between Ni^II^ and thiolate (Δ*G*′_NiS_). From Figure 4 (*right*), Δ*G*′_NiS_ correlates with Δ*G*′_0,**1**_ so that a stronger Ni^II^–S bond yields a more favorable reaction. The strongest bond is calculated for the native F430 in **E**, which pulls down Δ*G*_0,**1**_ and also the barrier Δ*G*_**1**_^≠^. Moreover, the correlation slope for Δ*G*′_NiS_ vs Δ*G*′_0,**1**_ is 2.8 as compared to the slope of −1.8 calculated for *E*° vs Δ*G*′_0,**1**_ (Figure 4). Thus, the change in the Ni–S bond strength across systems **A**–**E** dominates the change in Δ*G*′_0,**1**_, outweighing the counter-acting effect of *E*° by a factor of ∼1.5. As a result, the overall change in [Δ*G*′_ET_ + Δ*G*′_NiS_] quantitatively yields differences seen in free-energy profiles of **step 1** within **A**–**E** in Figure 3B. Our finding also suggests that the driving force for the biosynthesis of F430 consists in the electronic-structure adjustment of Ni to lower the barrier for methane production by strengthening the Ni–S bond formed during the catalytic **step 1**.

**Figure 5:**
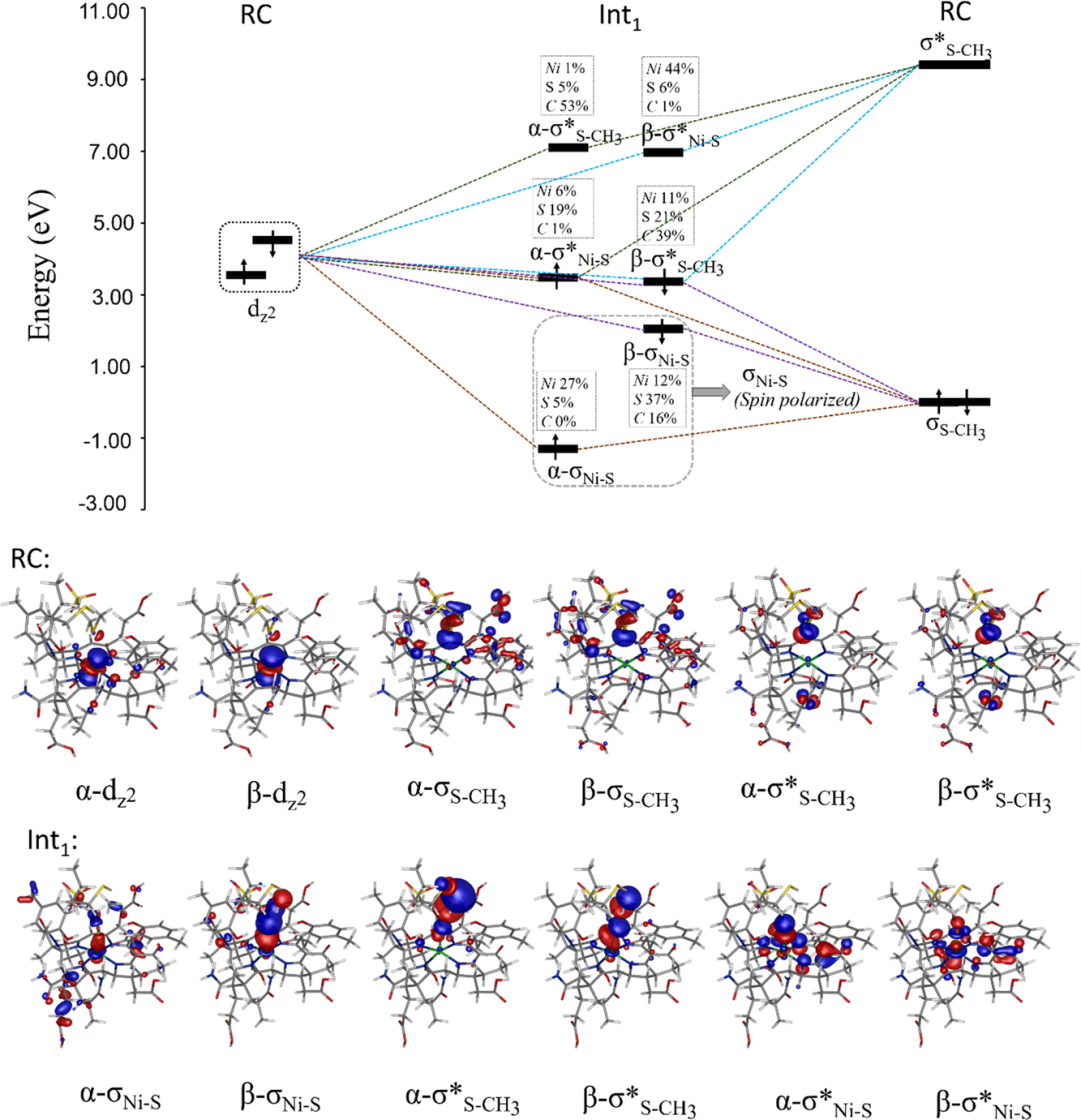
Representative MO diagram and molecular orbitals for **step 1** going from RC to Int_**1**_ as shown for **E**. The analogous MO diagrams for models **A**–**D** are given in Figure S9. MO energies are shown relative to the σ_S – CH_3__ energy, which serves as a reference value of 0 eV.

The representative molecular orbital (MO) diagram is given in Figure 5. It describes the four-electron three-center interaction between bonding/antibonding orbitals σ_S – CH_3__/σ_S – CH_3__^*^ of coenzyme M and the Ni-*d*_*z*^2^_ orbital in going from RC to Int_**1**_ in **step 1**. First, we notice a strong spin polarization of the Ni–S bond, with bonding α- and β-electron to be spatially and energetically distorted so that the α-electron is lower in energy and located more on Ni (due to exchange stabilization of α-electrons on Ni^II^), while the β-electron is higher in energy and located more on thiolate (cf. α-σ_Ni – S_ vs β-σ_Ni – S_ MOs). This immediately implies a significant covalent character of the Ni–S bond, with a pronounced thiyl character on S. Second, one of the two electrons initially located on Ni^I^ is transferred to the σ_S – CH_3__^*^ orbital, while one of the two σ_S – CH_3__electrons translocates to σ_Ni – S_^*^. Among the set **A**–**E**, the strongest Ni–S bond (calculated for **E** as shown in Figure 4) is the most covalent as reflected by the atomic composition of α-σ_Ni – S_ and β-σ_Ni – S_ with the largest Ni character in α-σ_Ni – S_ and thiyl character on S in β-σ_Ni – S_ (Figure S10). Conversely, the weakest Ni–S bond (calculated for **B**; Figure 4) is the least covalent as evidenced by the lowest Ni character in α-σ_Ni – S_ and thiyl character on S in β-σ_Ni – S_ (Figure S10). The most pronounced electron donation from thiolate to Ni in Int_**1**_ found for the native model **E** indicates the native corphinoid ligand to be a weaker electron donor compared to its biosynthetic precursors.

The electron-donation ability of the macrocyclic ligands in **A**–**E** was analyzed by means of AIM integration of the DFT-optimized electron density of their RC state (from Figure 3B). To this aim, the Ni ion was replaced by a point charge (*q*_P_) whose value varies from 0 to 1*e* in 0.1*e* steps. It probes ligand polarization as *q*_P_ grows. Ligand polarization *P*(*q*_P_) is calculated as
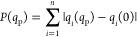
5where *n* is the number of atoms in the system, *q_i_*(*q*_P_) is the charge of the *i*th atom in response to the point charge *q*_P_, and *q_i_*(0) is the charge of the *i*th atom when *q*_P_ = 0*e*. As shown in Figure S11, *P*(*q*_p_) varies linearly in **A**–**E**, with the ligand in **A** being the most sensitive to increasing *q*_P_ (reflected in its slope *m*_**A**_ = 1.34, the highest in the set). Such a polarization gradually gets less responsive as the biosynthetic precursor approaches the native ligand F430 anchored in model **E**, for which the slope of *P*(*q*_p_) is 1.02. Thus, the corphinoid ligand in F430 is tuned to be the least responsive to the electric charge of the coordinated Ni ion, following the trend seen for the Ni–S bond strength and hence for the barrier height of the rate-determining step. The observed trend in ligand polarizability can be intuitively understood since the non-native cofactor **A** features the largest π-system which becomes increasingly saturated en route to **E**. Now, we will interrogate the connection of the distinct polarizabilities of ligands in **A**–**E** with the strengthening of the nascent Ni–S bond, which emerges as a key determinant of the efficacy of MCR.

**Figure 6:**
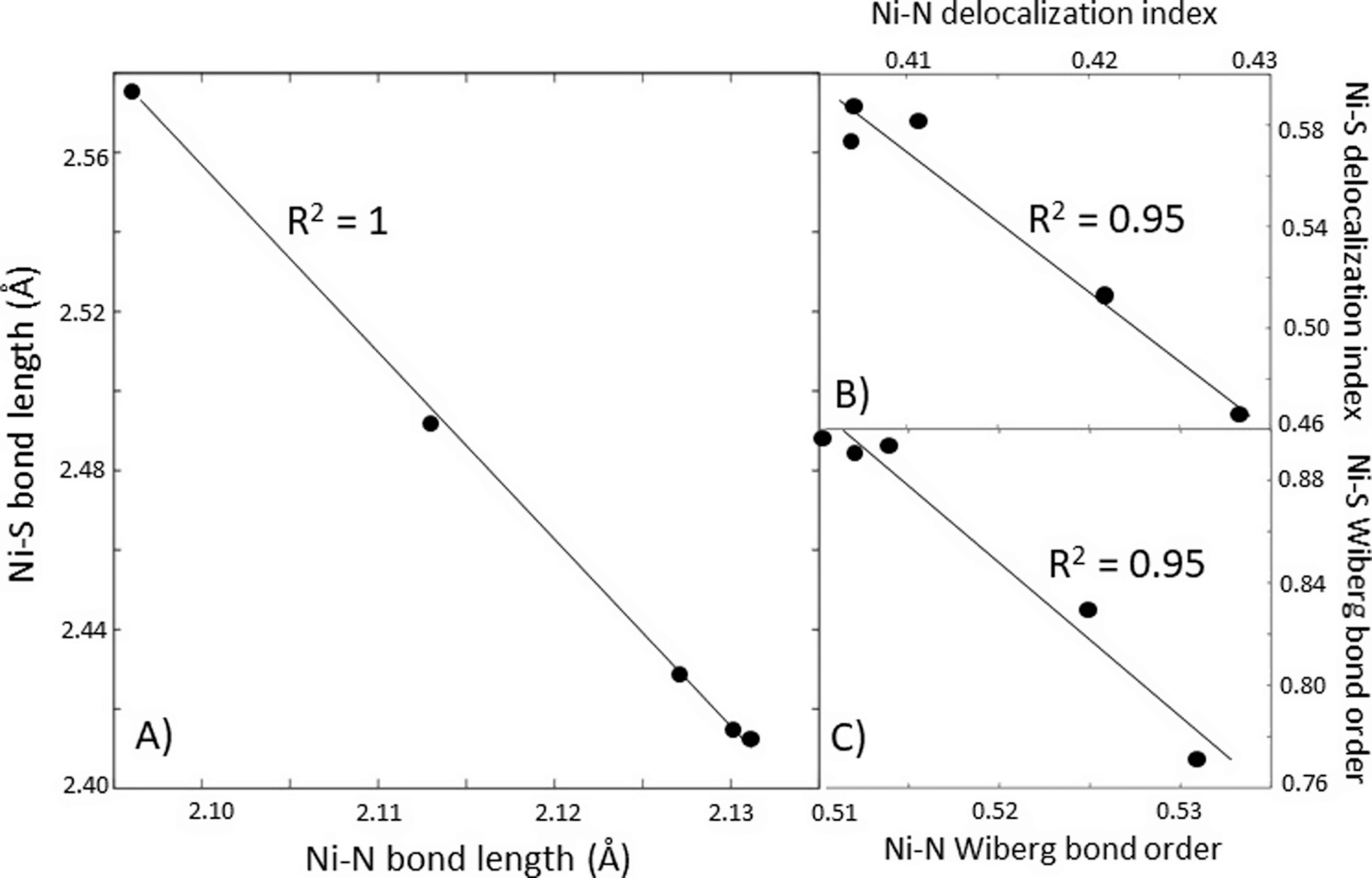
Correlation plots between the average length of the four bonds formed by Ni with the four ligating nitrogen atoms of porphyrin-like skeleton and Ni–S_CoM_ length (A); between the Ni–S and average Ni–N delocalization indices, obtained using the AIM formalism (B); and between the Ni–S and average Ni–N bond orders obtained through the Wiberg bond order analysis in Löwdin orthogonalized basis^49^ (C). Correlations were performed for the Int_**1**_ structures of the models **A**–**E**.

The bonding between Ni^II^ and the macrocyclic ligands in **A**–**E** was calculated through AIM-derived atomic charges (*q*_AIM_), which allowed a direct estimation of differential interaction strengths and delocalization indices as measures of electron sharing. From Figure S12, the average *q*_AIM_ of the four ligating N atoms is the least and the most negative for **E** (−1.15*e*) and **A** (−1.17*e*), respectively, while *q*_AIM_ for the Ni^II^ gradually gets more positive going back in the biosynthetic pathway (+1.17*e* for **E**, +1.21*e* for **A**). With these charge differences, we estimated the differential Ni^II^-ligand interaction by means of Coulombic law to be ca. 12 kcal mol^–1^ weaker in **E** than in **A** (see Supporting Information for the details and Table S6 with the values for systems **A**–**E**). This quantity, derived from electrostatics, misses the covalent component of bonding. However, the fact that the ligating N atoms in the native F430 bear the lowest electron density translates into the smallest Ni–N bond order, which translates into the largest Ni–S bond order (and hence, the highest covalency) due to electron-donation competition. The electrostatic analysis agrees with the change in both Ni–N and Ni–S bond lengths, displaying the shortest Ni–S and the longest Ni–N distances in the native **E** (Figure 6). Such a negative proportionality between the Ni–N and Ni–S distances (and their bond orders) is also observed in Figure S13 for the simplified models used in the thermodynamic cycle analysis in Figure 4. As a final and qualitative note, the macrocyclic π-system is the least conjugated in model **E**, which implies the densest π-system within the **A**–**E** set, i.e., the most capable of displacing σ electron density from the π-region. This effect may contribute to a lower σ-electron density on N (due to σ/π-electron density polarization^48^), leading to a lower Ni–N covalency. We also largely attribute to this effect the trends observed in the reduction potential and splitting of the spin (singlet/triplet) states of the Ni^II^ center in **A**–**E**, discussed earlier in the text. As for the metal-ring π-interactions, we do not expect them to affect the trends in Ni–S/Ni–N covalency and thus the reactivity due to full electron occupancy of *d*_π_(*xz*/*yz*) orbitals. Although it is reasonable to expect electronic repulsion between *d*_π_ and π-ring electrons to be less pronounced for more conjugated models (e.g., **A**) compared to less conjugated **E**, the π-metal/ring repulsion is expected to be approximately the same in both redox states of Ni and, therefore, to have a minor impact on the observed trends in **A**–**E**.

In addition, a consistent trend is obtained for Ni–S vs Ni–N Mulliken bond order derived from CASSCF calculations, which were performed on top of the DFT-optimized Int_**1**_ structures of the models **A**–**E** (Figure S14). Here, the observed trend is rationalized through the configuration interaction (CI) vector analysis of the CASSCF wavefunctions: the admixture of the S *p_z_* → Ni *d*_*z*^2^_ excited states in the ground state gradually increases from **B**/**A** to **E**, while the contributions of N *p_x/y_* → Ni *d*_*x*^2^ – *y*^2^_ excited states generally decrease. It is also noticeable that the dominant determinant with the contribution of ∼60% to the total CASSCF wavefunction (in case of all models) corresponds to the antiferromagnetic coupling between the triplet configuration on the nickel ion and the methyl radical, while the two subsequent determinants (each with the weight of 15%) are characterized as methyl radicals combined with the open-shell singlet configuration on the nickel ion. Thus, ∼90% of the total CASSCF wavefunction comes from a single configuration with three singly occupied orbitals, which is fully consistent with the presented DFT calculations.

### Connection between Kinetic Energy Distribution within the Transition-State Reactive Mode in **Step 1** and Macrocycle Electron-Donation Ability

**Figure 7:**
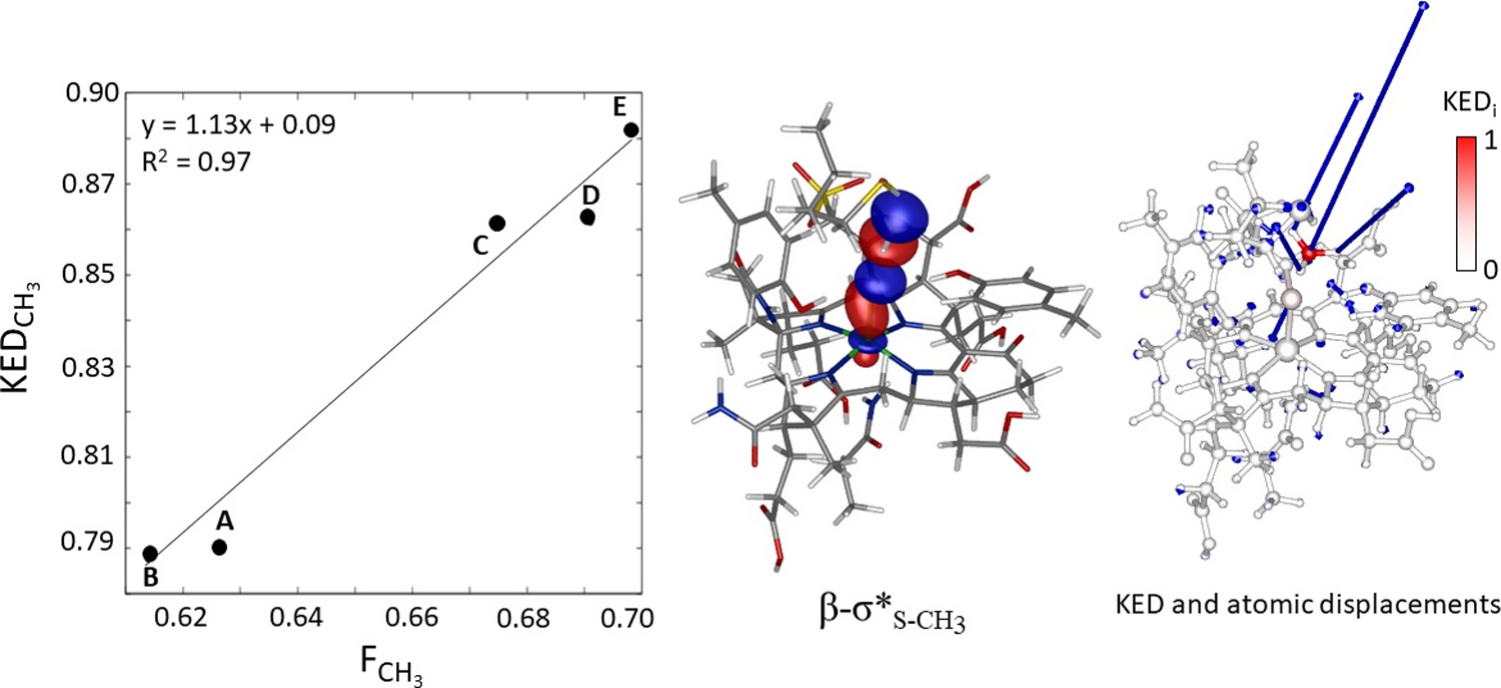
Correlation plot between fraction of kinetic energy of the reactive mode belonging to the transient CH_3_ radical (KED_CH_3__) and fraction of the atomic *p*_C_ orbital in σ_S – CH_3__^*^ relative to the atomic *p*_S_ orbital (*F*_CH_3__) for **A**–**E**, where *F*_CH_3__ calculated as (*p*_C_[%]/(*p*_C_[%] + *p*_S_[%])), (*left*). Note that an analogous correlation of KED_CH_3__with the % of *p*_C_ in σ_S – CH_3__^*^ is given in Figure S15. Relevant β-σ_S – CH_3__^*^orbital for the key S–CH_3_ cleavage step, associated with KED concentrated in the motion of the CH_3_ radical (*center*). KED mapping and atomic displacements in the reactive mode of the S–CH_3_ cleavage step (*right*).

As already noticed, a β-electron from Ni-*d*_*z*^2^_ transfers to the unoccupied antibonding σ_S – CH_3__^*^ orbital along the reaction coordinate of **step 1** and thereby weakens the S–CH_3_ bond. Electron transfer is accomplished at TS_**1**_, yielding the essentially broken S–CH_3_ bond with a kinetic energy distribution within the TS**_1_** reactive mode that is predominantly localized on the transient methyl radical (KED_CH_3__= 0.88 for model **E**; see earlier text, Figure S7). The singly occupied β-σ_S – CH_3__^*^ orbital at TS_**1**_ (β-σ_S – CH_3__^*^ in Int_**1**_) keeps its antibonding character between the *p*_S_ and *p*_C_ atomic orbitals, the latter of which is dominant (e.g., ∼19 vs ∼43% for model **E**). Considering only these two components, the fraction of *p*_C_ in σ_S – CH_3__^*^ relative to *p*_S_, which is given as *F*_CH_3__ = (*p*_C_[%]/(*p*_C_[%] + *p*_S_[%])), lies within the range of ∼0.6–0.7 across **A**–**E**. This is close to the ∼0.8–0.9 range for the corresponding KED_CH_3__ values. Indeed, as shown in Figure 7, *F*_CH_3__ correlates quantitatively with KED_CH_3__. A larger asymmetry in β-σ_S – CH_3__^*^ in favor of the CH_3_ group, which yields a more concentrated KED on CH_3_, is directly linked to a larger asymmetry in β-σ_Ni – S_ in favor of the sulfur component, which, in turn, and due to the above-mentioned spin polarization, is connected with a larger α-σ_Ni – S_ asymmetry in favor of the Ni component. The magnitude of KED_CH_3__ and its evolution across the models **A**–**E** is therefore a direct consequence of Ni–S bond covalency and corphinoid electron-donation ability.

### Redox and Ni–S Bond Strength Factors and Their Effects on Reaction **Step 3**

As already shown above, **step 3** is not found to be rate-determining and therefore not as critical as **step 1**. However, it is still interesting to understand whether and how the interplay of redox vs Ni–S bond strength, which controls the energetics of **step 1**, also affects **step 3**. Following Figure 3B, **step 3** leads to formation of the disulfidic bond between coenzymes M and B, with the concomitant one-electron reduction of the Ni^II^ center and Ni–S bond cleavage. Thus, from the perspective of the cofactor, **step 3** can be considered as a reverse process of **step 1**.

Indeed, **step 3** is calculated to be the least exergonic for the model **E** with the native F430, while it becomes gradually more exergonic in going backward from late- to early-stage biosynthetic precursors (**step 3b** in Figure 3B). In parallel, the cleavage of the Ni–S bond gets increasingly feasible as reduction of the Ni^II^ center becomes less favorable in going from **E** to **A**. Formation of the S–S bond is independent of the Ni cofactor. In line with the trend in reaction free energy of the third step seen across the set, the same is seen for the barrier Δ*G*_**3**_^≠^. To gain a better insight in **step 3**, we deconstructed it through a thermodynamic cycle (Figure S16) to three distinct events: (i) the homolytic cleavage of the Ni–S bond (Δ*G*′_NiS-cleavage_), (ii) one-electron transfer from the anionic thiolate of SCoM to the Ni^II^ center (Δ*G*′_ET_), and (iii) the S–S bond formation (Δ*G*′_S–S_) between CoBS and SCoM. Overall, the sum of all three contributions (Δ*G*′_0,3_ = Δ*G*′_NiS-cleavage_ + Δ*G*′_ET_ + Δ*G*′_S–S_) is calculated to be the least negative for **E**. Since Δ*G*′_0,**3**_ correlates satisfactorily with the reaction free energy of **step 3** (Δ*G*_0,**3**_; Figure 3B), we conclude that the native **E** has the least favorable **step 3** due to the least favorable Ni–S bond cleavage. While both the reductive cleavage of S–CH_3_ and the Ni–S bond formation are essentially accomplished at TS**_1_** in **step 1**, the oxidative S–S bond formation (i.e., the reduction of Ni) is more advanced than disruption of the Ni–S bond at TS_**3**_ in **step 3** (Figure S17). Thus, asynchronicity in favor of the Ni reduction, which is most pronounced in **E**, outcompetes the unfavorable Ni–S bond cleavage. As a consequence, the opposite change in Δ*G*_**3**_^≠^ is less pronounced as compared to the change in rate-determining Δ*G*_**1**_^≠^ when passing from **A** to **E** (Figure S18).

### Key Reaction Factors in the Context of a Recently Proposed Mechanism

**Figure 8:**
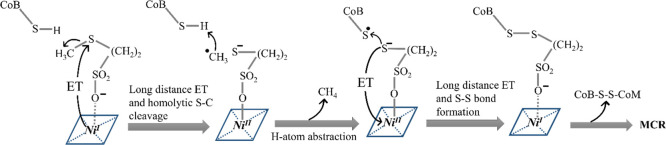
Alternative mechanism for production of methane by MCR as reported in ref (22).

Another mechanism proposed in literature for MCR-catalyzed production of CH_4_ is sketched in Figure 8.^22^ Here, we briefly make a remark on the viability of such a mechanism in light of our findings on the redox properties of the cofactor. As we elaborate in this work, the biosynthetic maturation of F430 through four distinct steps systematically fine-tunes the rate-determining **step 1** from Figure 1. In contrast, the alternative pathway reported in ref (22) seems triggered by long-distance electron transfer from Ni^I^ to the remote S–CH_3_ bond of coenzyme M. In such a mechanism, the Ni center remains coordinated by the sulfonate group of the coenzyme M along the whole catalytic process. If the Ni–OSO_2_R bond were not significantly strengthened during the oxidation of the Ni center, the electron transfer from Ni to the distant S–CH_3_ bond (Ni oxidation) would be central to this rate-determining barrier, in contrast to the canonical mechanism in Figure 1. Since Ni oxidation is less favorable in going from early-stage through late-stage precursors to the native F430, the alternative mechanism recently proposed by Ragsdale and co-workers^22^ could therefore be incompatible with the assumption that maturation of the cofactor F430 evolved to make production of CH_4_ effective. This deserves further investigation.

## Conclusions

**Scheme 2:**
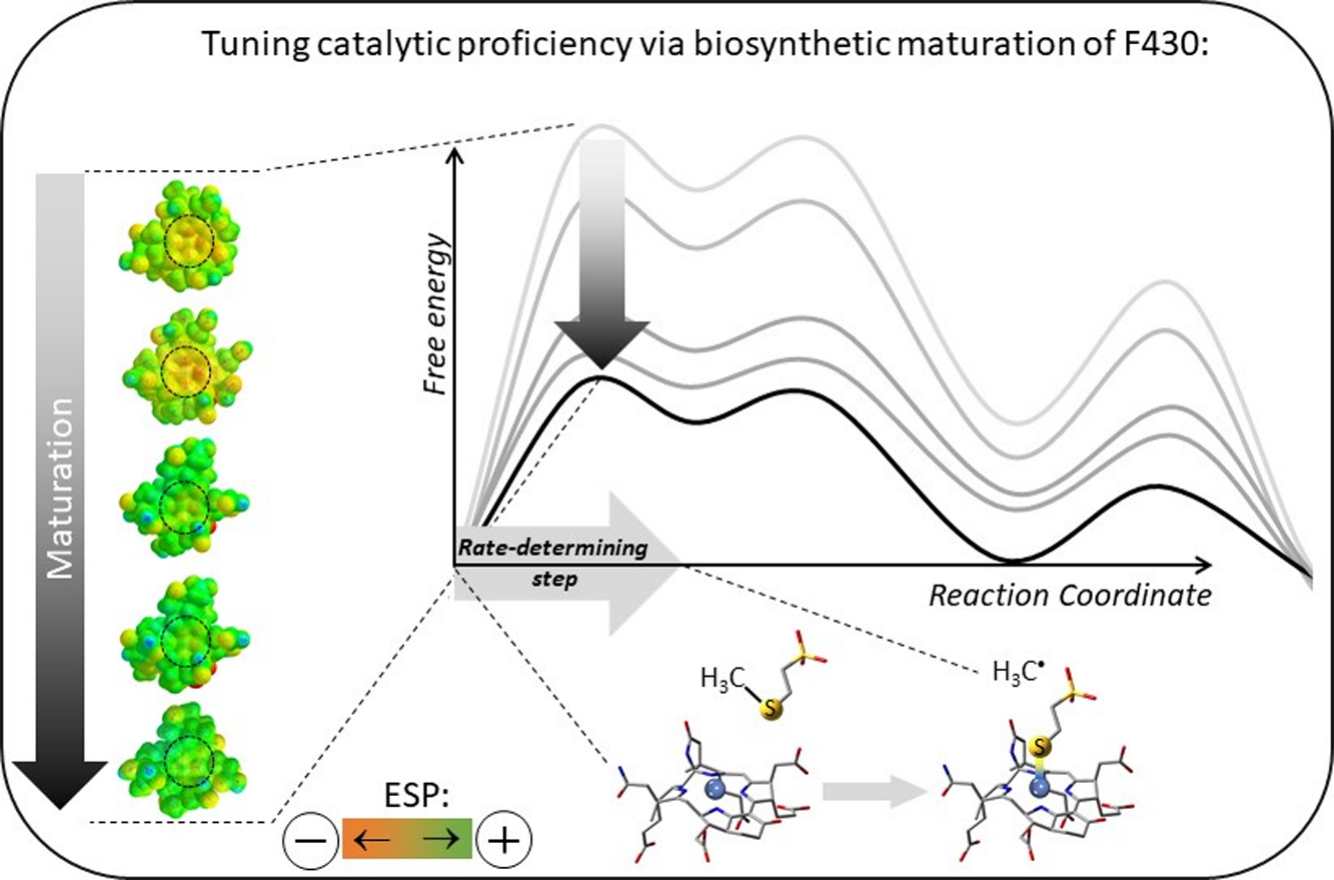
Biosynthesis of the F430 Cofactor Includes Four Sequentially Modified Precursors, Which Are Found to Be Less Efficient Catalysts in Production of CH_4_ Than the Native F430 The driving force for the highest catalytic competence of F430 is the formation of the strongest Ni–S bond in the rate-determining step that is allowed by the lowest electron-donation ability of the corphinoid ligand, as reflected by the least negative ESP in the zone bounded by dashed circles in the ESP-contoured structures.

Methanogenesis is a form of anaerobic respiration. As such, it has been evolutionary tuned to be as effective as possible. A final step in such a unique metabolic pathway produces methane from the reaction between a thiol and a thioether, which is catalyzed by MCR. The power horse of this enzyme is the active site hosting the Ni-containing F430 cofactor, whose catalytic properties were studied under the prism of evolutionary driving force for its biosynthesis. To this aim, we took advantage of four recently discovered biosynthetic precursors of F430 and investigated their reactivity relative to the native F430. Indeed, we found that F430 is best-suited for catalysis, displaying the lowest barrier for the rate-determining step involving the reductive cleavage of the thioether S–CH_3_ bond by the Ni^I^ center (Scheme 2). Surprisingly, the native F430 cofactor has the highest reduction potential and, therefore, from this perspective, it would be the least effective reductant for the cleavage of the S–CH_3_ bond. In fact, there is another factor that makes F430 the most effective in facilitating this critical step and that outweighs the unfavorable reduction potential: the strength of the Ni–S bond, which is formed upon the reductive S–CH_3_ cleavage and which is the strongest across the series of the studied active-site models anchoring F430 and its four biosynthetic precursors (Scheme 2). The strongest Ni–S bond is attributed to the highest covalent character, result of the weakest electron-donation ability of the native porphyrin-like F430 skeleton, which arises from complex (energy-demanding) chemical modifications in the biosynthetic pathway of F430. We also found that the transient methyl radical formed upon the reductive cleavage of S–CH_3_ concentrates most of the kinetic energy of the reactive mode at the corresponding transition state, which may facilitate a subsequent ballistic hydrogen atom abstraction from coenzyme B. Such a *dynamic* feature, which is again best-suited for the native F430 TS (∼90% of the kinetic energy of the reactive mode), is a direct consequence of the composition of the σ_S – CH_3__^*^ orbital at the TS, which in turn depends on the electronic-structure properties of the Ni cofactor, namely, the electron-donation ability of the corphinoid skeleton.

## Supporting Information Available

Depiction and cartesian coordinates of all models employed for the calculation of reduction potentials and for the modeling of reactivity; representative MO diagrams for models **A**–**D**; energetics for all studied thermodynamic cycles; and spin densities, kinetic energy distributions, and electron density analyses (PDF)
