# Supplementary material for: Reactivity Factors in Catalytic Methanogenesis and Their Tuning upon Coenzyme F430 Biosynthesis
Source: J Am Chem Soc. 2023 Apr 12;145(16):9039–51. doi: 10.1021/jacs.3c00469 (PMC10141249; doi:10.1021/jacs.3c00469)
Supplement: Supplementary file 1 — ja3c00469_si_001.pdf [file ja3c00469_si_001.pdf]

Supporting Information

Reactivity factors in catalytic methanogenesis and their tuning upon coenzyme F430 biosynthesis

Priyam Bharadwaz,[a] Mauricio Maldonado-Domínguez,[a] Jakub Chalupský,[a] and Martin Srnec[a,*]

[a] *J. Heyrovský Institute of Physical Chemistry, Czech Academy of Sciences, Prague, Czech Republic*

Table of Contents Page

Cluster models **A**–**E** and their overlay, optimized at the B3LYP(D3)/BS1/CPCM (ε_r = 4) level of theory. S4

Explicit parameters for used CPCM solvent model. S5

Reduction potentials of the F430 cofactor and its cognates calculated in various solutions and compared with the experimental data reported in the literature. S6

Potential energy profile for uncatalyzed reaction between the substrates CH_3S-CoM and CoB-SH in the absence of Ni-macrocycle complex. S7

Optimized structures of all minima and transition states across the reaction mechanism. S8

Correlation plot between $\Delta G_1^{\neq}$ and $\Delta G_{0,1}$. S9

Relative potential energies (ΔE), enthalpies (ΔH) and free energies (ΔG) of all key structures along the reaction coordinate from **Figure 3B.** S10

Mulliken spin density of all stationary points along the reaction coordinate in **A**-**E**. S11

Evaluation of $\Delta G_1^{\neq}$ for all charged carboxylate residues of models **A**-**E.** S13

Correlation plot of activation energy barrier $\Delta G_1^{\neq}$ between charged and protonated carboxylic acid residues in **A**-**E**. S13

Kinetic energy distribution (KED) of the CH_3 fragment of the reactive mode of TS1 in **A**-**E**. S14

Gibbs free energies associated in each step for thermodynamic cycle from **Figure 4** and **Figure S8** S15

Thermodynamic cycle of the first catalytic step involving triplet oxidized state of Ni S16

Reduction potentials of **A**-**E** calculated in both aqueous medium and ε_r = 4. S17

Representative MO diagram and molecular orbitals are shown for **A**-**D**. S18

Change of atomic composition of molecular orbitals key for Ni-S bond formation. S20

Polarization of the macrocyclic ligand in systems **A**-**E**, in response to a point charge S21

Charges for the coordinating N atoms and the Ni cation in the RC state of systems **A**-**E** S22

Calculation of Ni^{II}-macrocyclic ligand interaction for systems **A**-**E** using Coulomb's law S23

Correlation plot between Ni–N and Ni–S_{CoM} bond length and bond order from **Figure 4**. S24

Correlation plot between Mulliken Ni–N and Ni–S_{CoM} bond order from CASSCF calcs. S25

The correlation of KED_{CH_3} with the % of p_C in $\sigma^*_{S-CH_3}$ for **A**–**E**. S26

Thermodynamic cycle for the third step in the catalytic cycle from **Figure 1**. S27

Correlation plot between the Ni−S and S−S bond lengths at TS_3. S28

Correlation plot between the activation energy barrier of $\Delta G_1^{\neq}$ and $\Delta G_3^{\neq}$. S28

References S29

Cartesian coordinates of all optimized geometries. S30

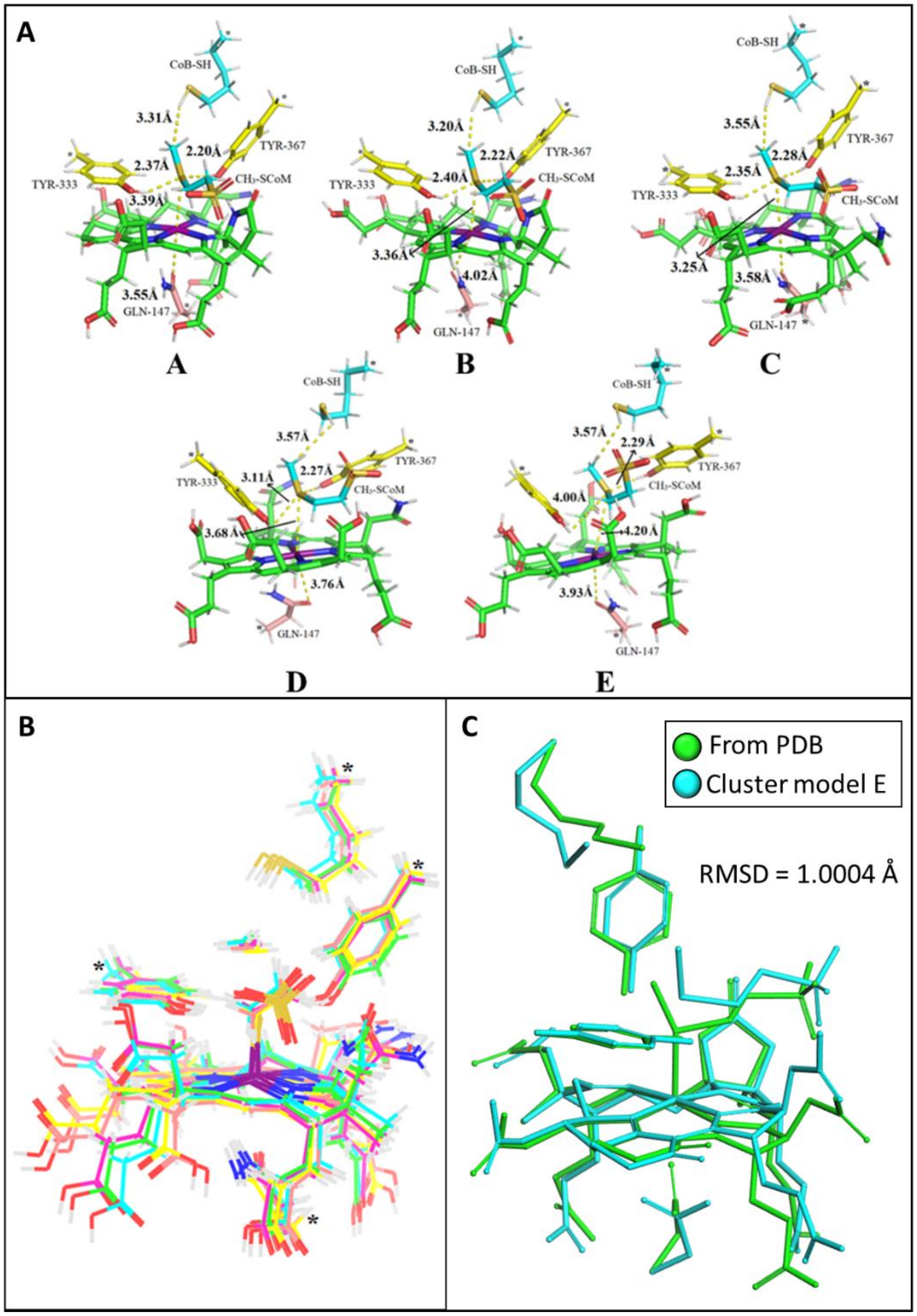

Figure S1. A. Optimized cluster models (**A**-**E**) anchoring F430 and the biosynthetic precursors of F430. All models were built from crystal structure 1HBN. All the key geometric parameters are shown in angstroms (Å); asterisks indicate the truncation point, which were kept fixed during optimizations. **B.** Overlay of cluster models **A**-**E**. **C.** Overlay of cluster model **E** (in cyan) and the reference structure taken from the PDB (in green), including the root mean square difference (RMSD) between the cartesian coordinates of all non-H atoms in both models.

Table S1: The following parameters were used in the conductor-like polarizable continuum model (CPCM) calculation to mimic the solvent environment for models **A** to **E**.

To specify the molecular cavity, we used universal force field (UFF) radii where the sphere radius is multiplied by an electrostatic scaling factor of 1.1. The molecular surface is represented by Van der Waals surface. The UFF estimated atomic radii for respective atoms are listed below.

Atoms types	Radii (r_j, in Å)
H	0.354
C (tetrahedral)	0.757
C (resonant)	0.729
N (trigonal)	0.685
N (resonant)	0.699
O (resonant)	0.680
O (trigonal)	0.634
O (linear)	0.639
S (tetrahedral, +6 oxidation state)	1.027
S (tetrahedral, +2 oxidation state)	1.064
S (trigonal)	0.854
S (resonant)	1.077
Ni	1.164

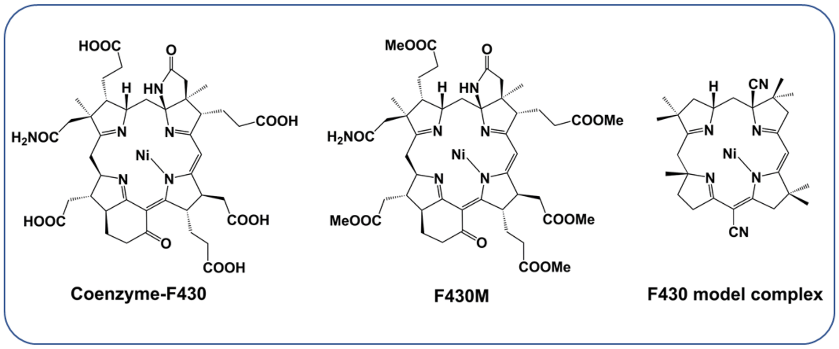

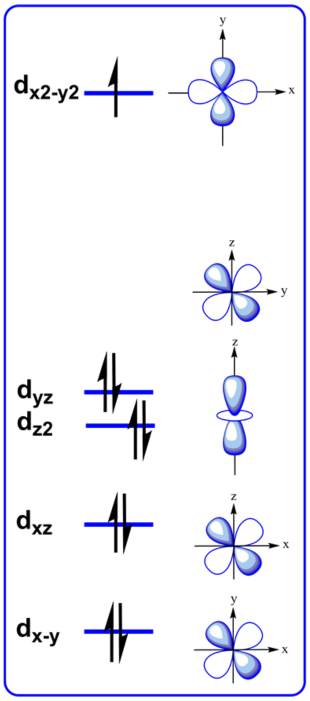

System	S_{ox}[a]	S_{red}[b]	$E°_{calc}$ [V]	$E°_{expt}$ [V]	$E°_{error}$[c] [V]	Reference electrode[d] at T=298K	solvent
Coenzyme-F430[e]	1 (1)	½ (½)	-0.64	-0.65	0.01	NHE	H_2O (pH=10.4)
Pentamethyl ester F430M[f]	1 (N/A)	½ (½)	-1.27	-1.29	0.02	Fc/Fc⁺	THF
	1 (N/A)	½ (½)	-1.31	-1.32	0.01	Fc/Fc⁺	DMF
	1 (0)	½ (½)	-1.25	-1.15	0.10	Fc/Fc⁺	CH_3CN
F430 model complex[g]	1 (1)	½ (½)	-0.88	-1.05	0.17	SCE	CH_3CN

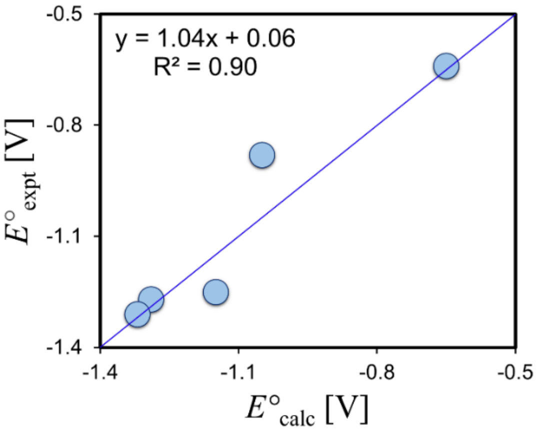

Figure S2. Reduction potentials of the F430 cofactor and its cognates calculated in various solutions and compared with the experimental data reported in the literature. [a]Ground spin states calculated for oxidized forms (experimental data in parentheses). [b]Ground spin states calculated for reduced forms (experimental data in parentheses). [c]Calculated unsigned deviation of E^o_{calc} from E^o_{expt} are shown (the mean unsigned error is 0.06 V). [d]Absolute potential of the reference electrode: (*i*) normal hydrogen electrode (NHE) in water: 4.28 eV (Ref. 1); (*ii*) ferrocene/ferrocenium (Fc/Fc^+): 5.17, 4.97 and 4.98 eV in THF (Ref. 2), DMF (Ref. 2) and CH_3CN (Ref. 3), respectively; (*iii*) saturated calomel electrode (SCE): 4.60 eV in CH_3CN (Ref. 3). [e]Experimental data taken from Ref. 4 & 5. [f]Experimental data taken from Ref 6. [g]Experimental data taken from Ref. 7. The orbital splitting of the *d* manifold of square-planar Ni^I is also shown. The correlation plot between E^o_{calc} and E^o_{expt} is also provided. Note that we used dielectric constants 78.3, 7.4, 37.2, 35.7 for H_2O, THF, DMF and CH_3CN respectively. QM model we considered as shown in the top of **Figure S2** for the isolated systems without the presence of any axial ligation.

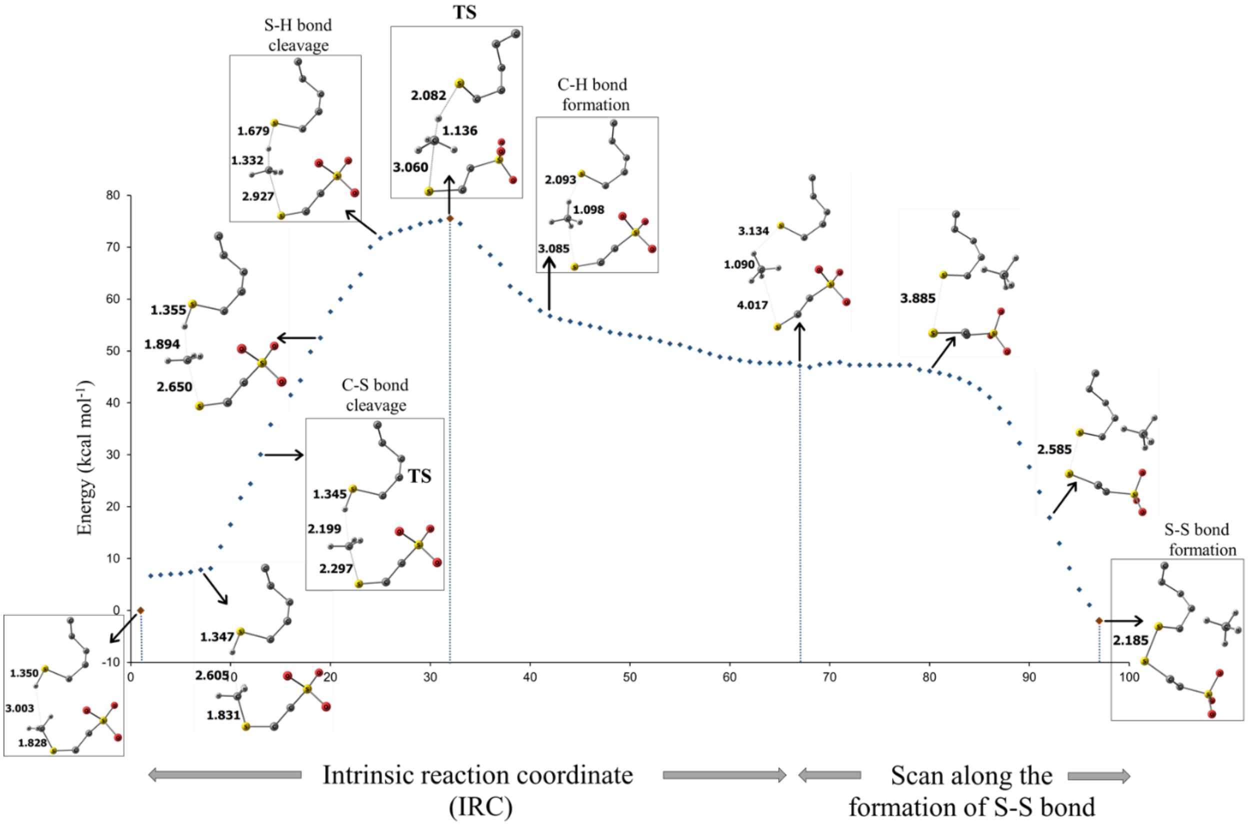

Figure S3. B3LYP(D3)/BS1/CPCM(ε_r = 80.0) potential energy profile for uncatalyzed reaction between the CH_3S-CoM and CoB-SH substrates (*i.e.*, in the absence of a Ni-macrocycle complex). Intrinsic reaction coordinate (IRC) for the first (single-barrier) step that includes three events: S–CH3 bond cleavage, the S–H bond cleavage and CH_4 formation. For the second step, which corresponds the S-S bond formation, we performed scan calculations which indicate that this step is almost barrierless. Key structures with some selected interatomic distances (in Å) along the reaction trajectory are displayed. The structures in the box represent the early stage accomplishment for the of cleavage and formation of H_3C–S, S–H, C–H and S–S bond respectively. We note that a search for alternative transition states were unsuccessful.

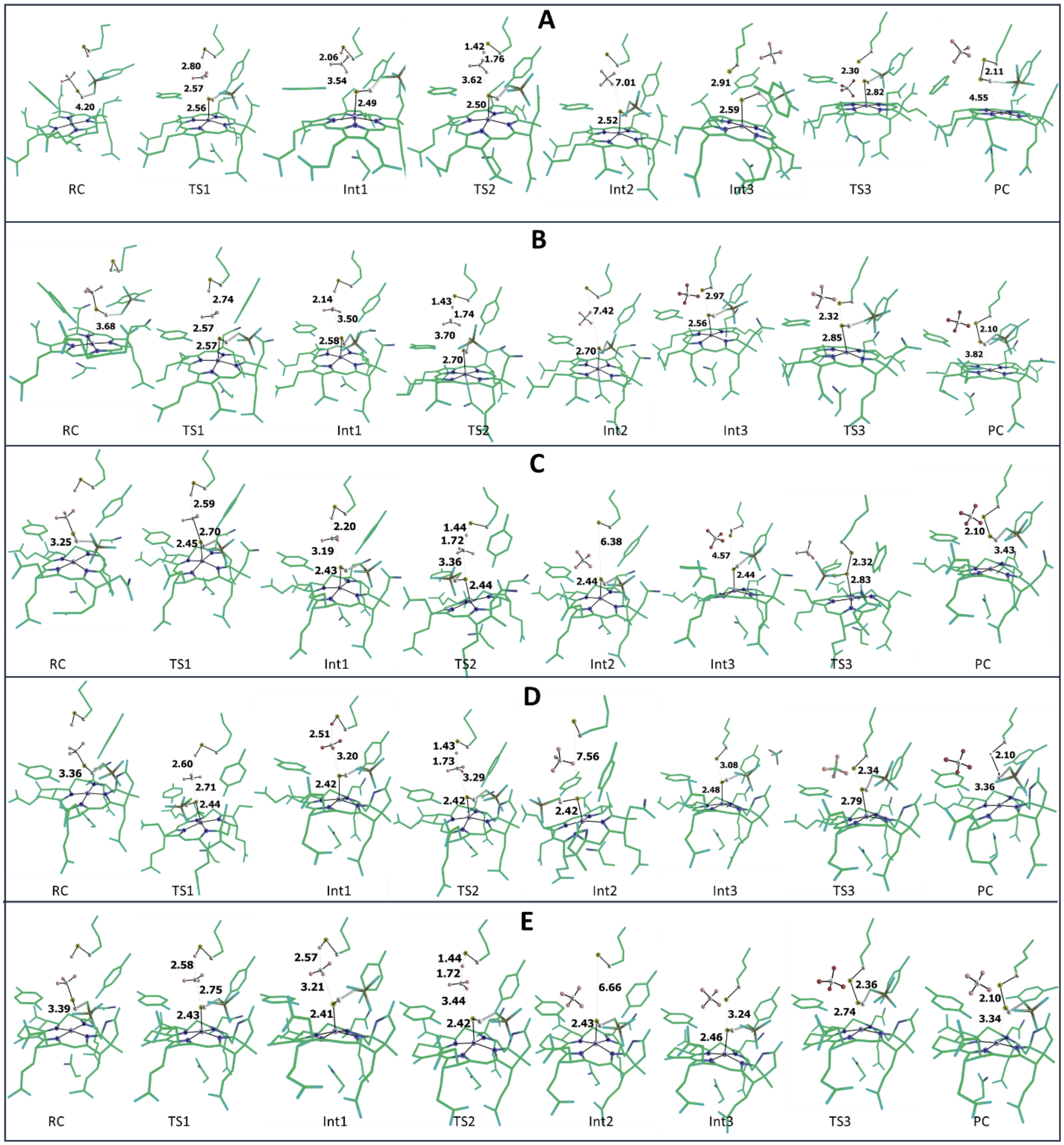

Figure S4. All key points along the reaction pathway from **Figure 4** are shown here. The selected bond lengths are displayed (in Å). For the sake of clarity, hydrogen atoms (except the key hydrogens involved in the reaction) are not depicted.

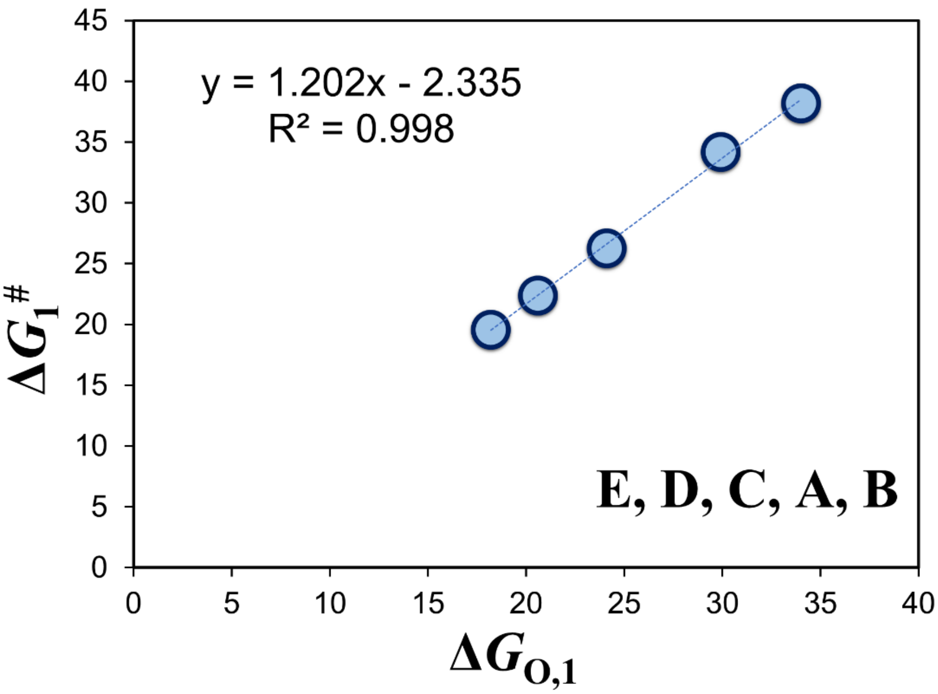

Figure S5: Correlation plot between the free-energy barrier $\Delta G_1^{\neq}$ and the free energy of reaction ($\Delta G_{0,1}$) for step **3** from the catalytic cycle (energies in kcal mol^{-1}).

Table S2: Relative potential energies (Δ*E*), enthalpies (Δ*H*) and free energies (Δ*G*) of all key structures appearing along the catalytic reaction coordinate from **Figure 3B** (main text), as calculated with respect to the initial reactant complex (RC) of the model **A-E** in each model. The values for the uncatalyzed reaction from **Figure 3A** are also provided.

	**RC**	**TS_1**	**Int_1**	**TS_2**	**Int_2**	**Int_3**	**TS_3**	**PC**
				**Δ*E*/Δ*H*/Δ*G*** (kcal mol^{-1})				
**A**	0.0/0.0/0.0	39.1/ 37.3/34.2	27.8/26.5/29.9	28.3/25.9/31.7	15.6/16.1/11.0	0.3/2.6/6.2	4.3/6.1/11.5	-5.1/-4.7/-4.0
**B**	0.0/0.0/0.0	44.3/42.1/38.2	41.3/38.6/34.0	42.7/38.5/36.3	19.9/19.7/15.0	10.2/12.4/14.1	15.3/16.8/19.1	-10.1/-9.0/-7.5
**C**	0.0/0.0/0.0	32.2/29.5/26.3	31.4/29.0/24.1	32.5/28.8/26.6	9.8/10.2/5.5	8.5/9.1/3.3	11.2/10.5/9.8	-6.9/-5.7/-7.2
**D**	0.0/0.0/0.0	23.0/20.6/22.4	20.4/18.5/20.6	23.2/20.1/22.5	0.2/1.1/2.8	-5.6/-4.4/-0.7	0.0/1.2/6.6	-13.8/-12.9/-8.5
**E**	0.0/0.0/0.0	21.3/19.0/19.6	19.5/17.4/18.2	21.1/17.5/20.1	-2.3/-1.8/-1.2	-7.2/-6.3/-4.2	-1.3/-0.3/4.3	-11.7/-11.3/--10.5

	RC	TS	PC
	**Δ*E*/Δ*H*/Δ*G*** (kcal mol^{-1})		
**Uncatalyzed reaction**	0.0/0.0/0.0	76.5//81.2/88.9	-2.3/-1.7/-4.6

Table S3: Mulliken spin density analysis of all the stationary points calculated along the reaction trajectory in **A-E** (trajectory displayed in **Figure 3B**).

Model	Stationary point	Mulliken spin density			
		Ni	CH_3-SCoM		CoB-SH
			S_{CoM}	C_{CoM}	S_{CoB}
**A**	RC	0.884	0.000	0.000	0.000
	TS_1	1.554	0.226	-0.682	0.000
	Int_1	1.731	0.172	-0.930	-0.044
	TS_2	1.735	0.175	-0.900	-0.056
	Int2	1.755	0.179	0.010	-0.768
	Int3	1.702	-0.044	0.000	-0.531
	TS3	1.242	-0.001	0.000	-0.142
	PC	0.888	0.000	0.000	0.000
**B**	RC	0.922	0.000	0.000	0.000
	TS_1	1.535	0.208	-0.651	0.000
	Int_1	1.804	0.234	-0.932	-0.049
	TS_2	1.700	0.168	-0.855	-0.114
	Int2	1.710	0.151	-0.016	-0.842
	Int3	1.778	-0.001	0.000	-0.509
	TS3	1.297	0.015	0.000	-0.108
	PC	0.881	0.000	0.000	0.000
**C**	RC	0.931	0.000	0.000	0.000
	TS_1	1.561	0.195	-0.781	0.000
	Int_1	1.684	0.168	-0.951	-0.099
	TS_2	1.699	0.156	-0.834	-0.239
	Int2	1.704	0.194	0.0	-0.867
	Int3	1.704	0.224	-0.005	-0.801
	TS3	1.066	-0.041	0.000	-0.128
	PC	0.900	0.000	0.000	0.000

**D**	RC	0.922	0.0	0.0	0.0
	TS_1	1.535	0.208	-0.645	-0.030
	Int_1	1.690	0.227	-0.922	-0.009
	TS_2	1.701	0.168	-0.901	-0.241
	Int2	1.710	0.151	-0.016	-0.842
	Int3	1.631	0.031	0.0	-0.616
	TS3	1.297	0.015	0.0	-0.108
	PC	0.878	0.0	0.0	0.0
**E**	RC	0.884	0.0	0.0	0.0
	TS_1	1.554	0.226	-0.669	-0.022
	Int_1	1.731	0.172	-0.985	-0.106
	TS_2	1.735	0.175	-0.900	-0.223
	Int2	1.754	0.183	-0.019	-0.785
	Int3	1.616	0.041	0.0	-0.657
	TS3	1.149	0.015	0.0	-0.254
	PC	0.921	0.0	0.0	0.0

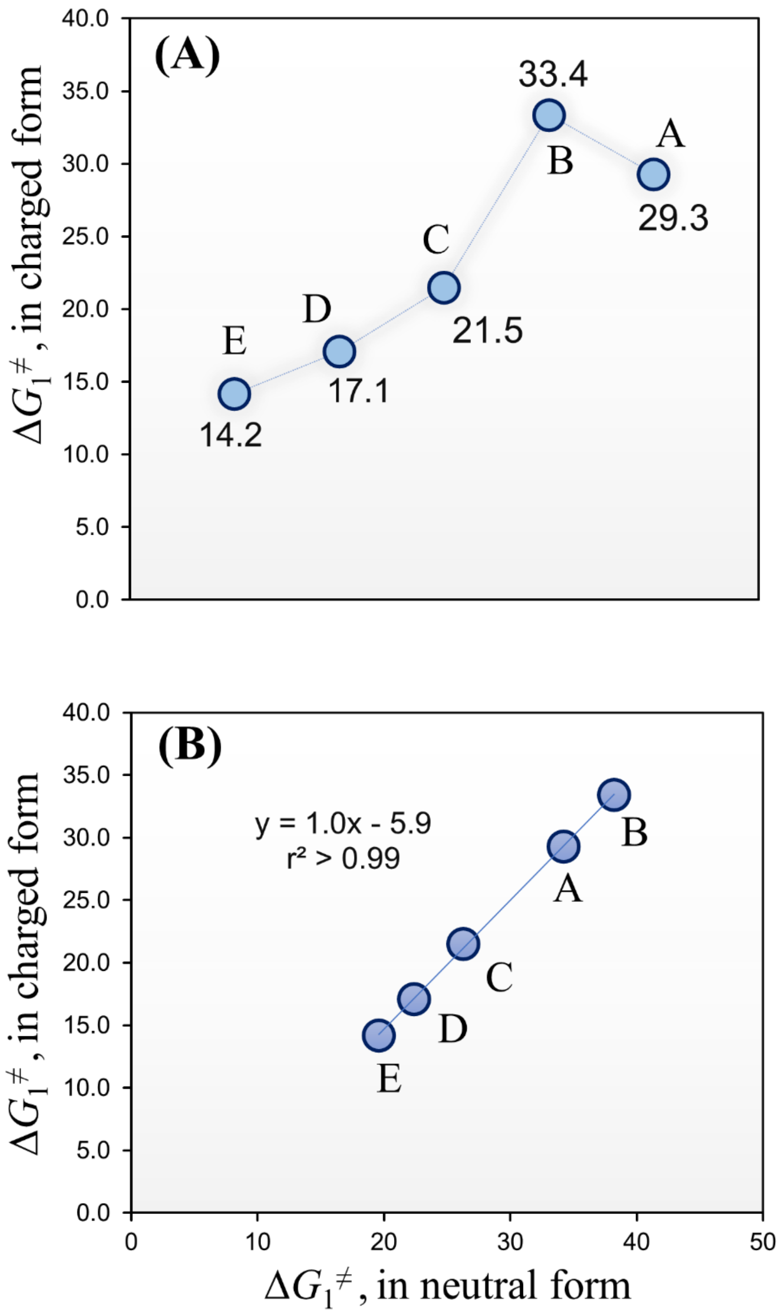

Figure S6. **(A)** Evaluation of $\Delta G_1^{\neq}$ for **step 1** with deprotonated carboxylate groups of the macrocycle in models **A-E**. (**B**) Correlation plot of $\Delta G_1^{\neq}$ calculated for models **A-E** with deprotonated peripheral carboxylates (total charge in models **A** to **E** are -10, -8, -7, -7 and -6 respectively) vs. $\Delta G_1^{\neq}$ calculated for the corresponding models with protonated carboxylic groups (total charge in protonated models **A** to **E** are -2, -2, -1, -1 and -1 respectively). The calculated energies are in kcal mol^{-1}.

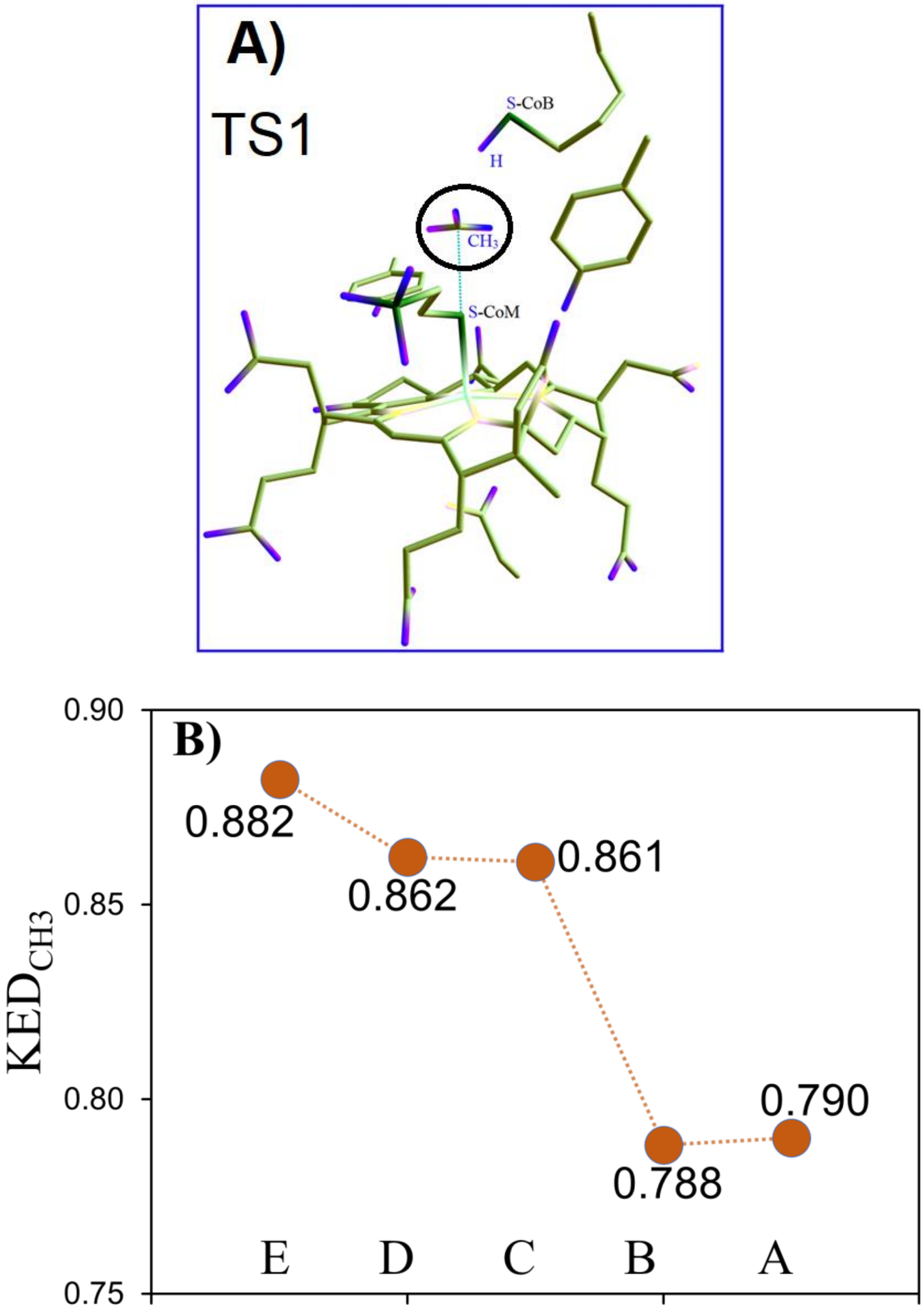

Figure S7. The TS_1 structure (panel **A**) and fraction of kinetic energy of the reactive mode at TS_1 belonging to the transient methyl-radical fragment (panel **B**), as calculated for all models **A**–**E**.

Table S4: Reduction potentials calculated for the Ni^{I}/Ni^{II} couple in the isolated Ni-complex (from the model **A'-E'**) and Gibbs free energies of each event in thermodynamic cycle of **step 1** from **Figure 4** in the main text (**A'-E'** defined in **Figure 4** and **S8**). Reduction potentials of the F430 cofactor and its precursors (from **Figure 4**) calculated using eq. 2. The normal hydrogen electrode NHE in water, with absolute potential of 4.28 eV was taken as reference (Ref. 1). In these the QM calculations, solvation was mimicked using CPCM (ε_r = 4). The details of ground spin states for both oxidized and reduced form (spin states S_{ox} and S_{red}, respectively) and spin states gaps are further provided in **Table S5A**.

(A) Explicit values are provided from **Figure 4**.

	E'^{O} (V)	$\Delta G'_{cleavage}$	$\Delta G'_{ET}$	$\Delta G'_{NiS}$	$\Delta G'_{0,1}$
**E'**	-0.21	53.1	15.6	-58.4	10.5
**D'**	-0.45	53.1	10.1	-49.0	14.2
**C'**	-0.63	53.1	6.0	-41.5	17.6
**B'**	-1.39	53.1	-11.5	-15.1	26.5
**A'**	-1.32	53.1	-9.9	-19.0	24.1

(B) Explicit values are provided from **Figure S8**.

	E'^{O} (V)	$\Delta G'_{cleavage}$	$\Delta G'_{ET}$	$\Delta G'_{NiS}$	$\Delta G'_{0,1}$
**E'**	-0.21	53.1	15.6	-58.4	10.5
**D'**	-0.45	53.1	10.2	-49.1	14.2
**C'**	-0.44	53.1	10.5	-46.0	17.6
**B'**	-1.08	53.1	-4.2	-22.4	26.5
**A'**	-1.00	53.1	-2.5	-26.4	24.1

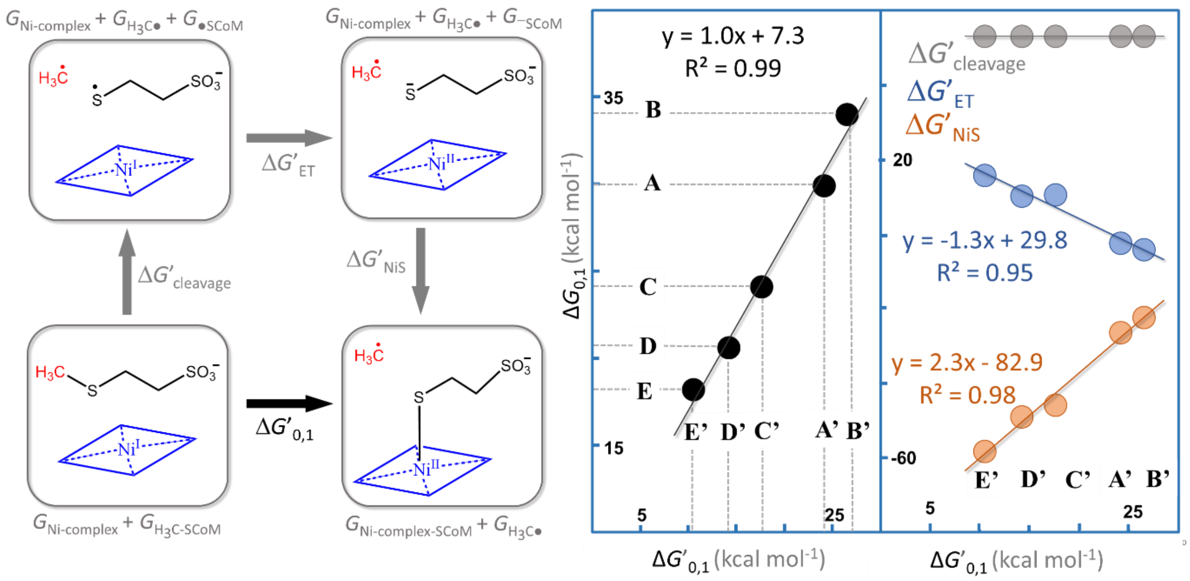

Figure S8: Thermodynamic cycle of the first catalytic step involving the formation of the transient methyl radical coupled with one-electron oxidation of the Ni^{I} center and Ni–thiolate bond formation. Note that, the thermodynamic cycle is constructed using triplet oxidized state of Ni in the upper-right corner of the cycle (**Table S4B**). The whole process is dissected into three steps: (*i*) the homolytic cleavage of the S–CH_3 bond ($\Delta G'_{cleavage}$), (*ii*) electron transfer from the Ni^{I} center to the S-radical ($\Delta G'_{ET}$); (*iii*) the Ni–S bond formation ($\Delta G'_{NiS}$). Note that $\Delta G'$ values were calculated from Gibbs free energies of individual CPCM(ε_r=4.0)-solvated species, as indicated in the figure and using eq. 1; the sum of three sequential ΔG's steps equals to $\Delta G'_{0,1}$, which is analogous to the free energy of reaction $\Delta G_{0,1}$ from **Figure 3B**. The labels **A'**, **B'**, **C'**, **D'** and **E'** are used in parallel to labels for the full models **A-E**; the "prime" symbol refers to the fact that the model consists of individual (infinitely separated) moieties such as the $Ni^{I/II}$-complex, H_3C–SCoM, $^{-/\bullet}$SCoM, $CH_3^{\bullet}$, the Ni^{II}–SCoM complex.

Table S5: Reduction potentials of F430 cofactor and its precursors (in their isolated forms without the presence of any axial ligation and second-shell residues; as considered in **Figure 4**) calculated by using eq. 2 and considering two different solvation microenvironments **(A)** ε_r = 4 and **(B)** aqueous medium (ε_r = 80.0). The normal hydrogen electrode NHE in water, with absolute potential of 4.28 eV was taken as reference (Ref. 1). The ground spin states for both oxidized and reduced form (spin states S_{ox} and S_{red}, respectively) as well as the spin state splittings in the oxidized forms (singlet/triplet gaps) are shown. The right panels in **(A)** and **(B)** show correlation plots between reduction potentials of the models **A-E** (models from **Table 1**) and the isolated cofactors (isolated form) calculated in ε_r = 4 or 80 (isolated forms from **Figure 4** and **Table S4**)

(A) In eps = 4

Cofactor only	S_{ox} / S_{red}	$E°_{calc}$ (V)	Oxidized form: Singlet/triplet gap (kcal mol^{-1})
A'	0 / ½	-1.32	7.4
B'	0 / ½	-1.39	7.3
C'	0 / ½	-0.63	4.5
D'	0 / ½	-0.45	0.1
E'	1 / ½	-0.21	0.2

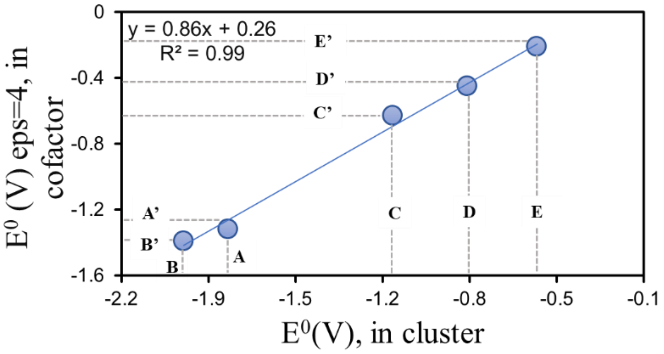

(B) In aqueous medium, ε_r = 80

Cofactor only	S_{ox} / S_{red}	$E°_{calc}$ (V)	Oxidized form: Singlet/triplet gap (kcal mol^{-1})
A'	0 / ½	-1.10	6.2
B'	0 / ½	-1.21	8.5
C'	0 / ½	-0.80	5.7
D'	1 / ½	-0.67	1.9
E'	1 / ½	-0.64	2.3

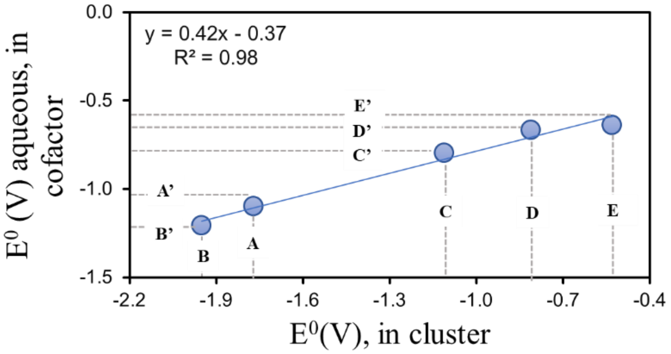

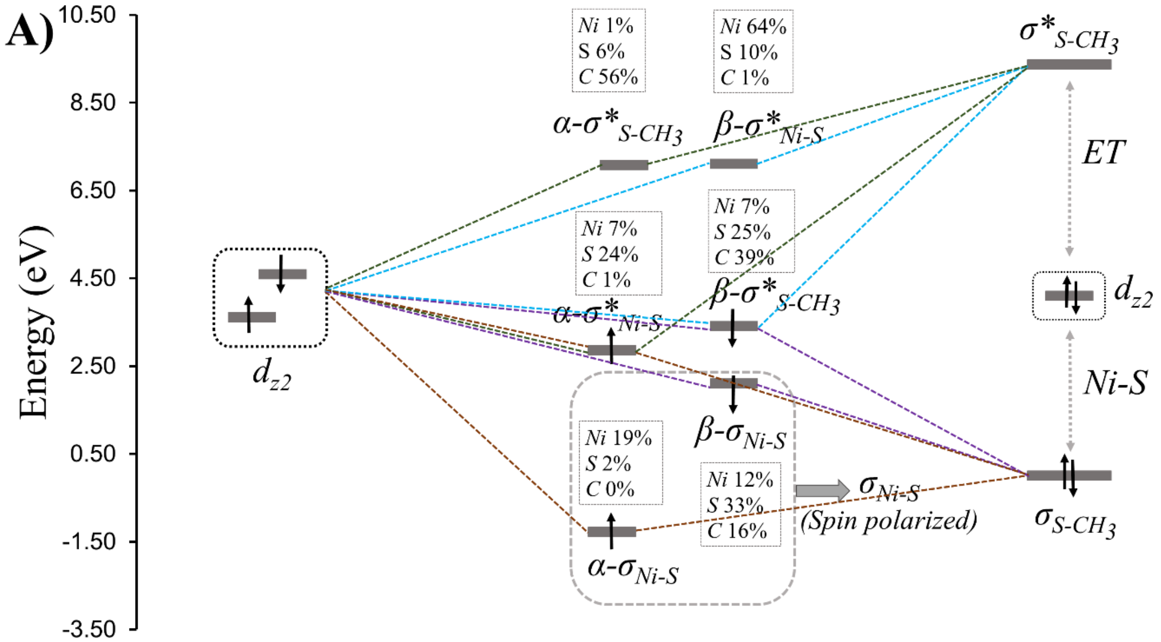
A)
Energy (eV)
10.50
8.50
6.50
4.50
2.50
0.50
-1.50
-3.50
Ni 1%
S 6%
C 56%
Ni 64%
S 10%
C 1%
σ*S-CH3
α-σ*S-CH3
β-σ*Ni-S
ET
Ni 7%
S 24%
C 1%
Ni 7%
S 25%
C 39%
β-σ*S-CH3
dz2
α-σ*Ni-S
Ni-S
β-σNi-S
Ni 19%
S 2%
C 0%
Ni 12%
S 33%
C 16%
σNi-S
(Spin polarized)
σS-CH3
α-σNi-S

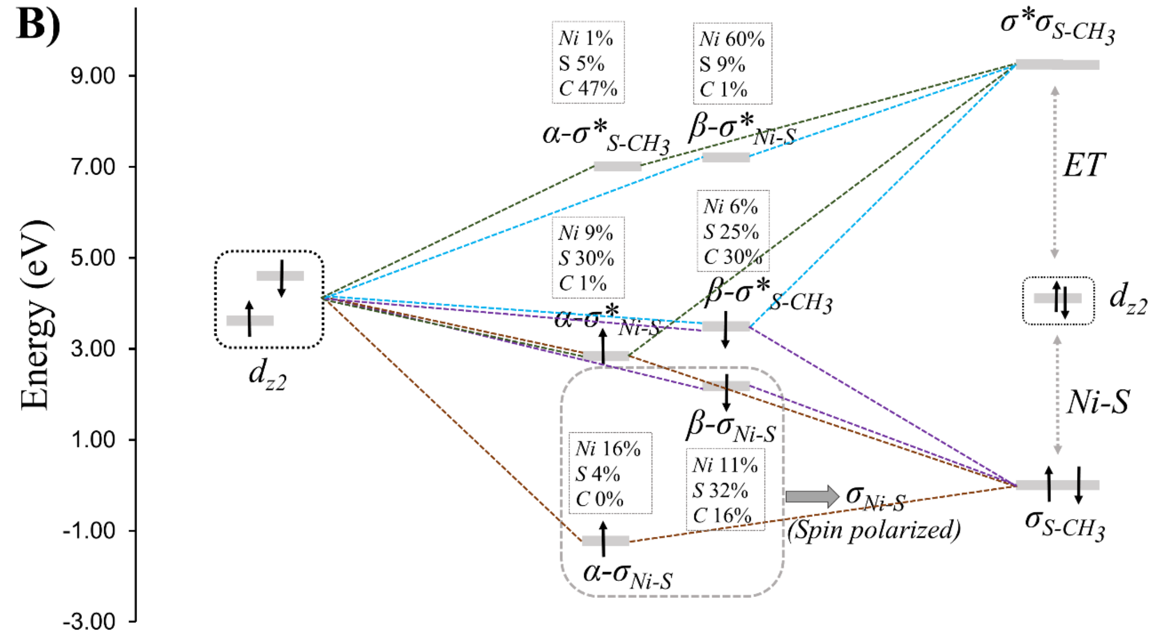
B)
Energy (eV)
9.00
7.00
5.00
3.00
1.00
-1.00
-3.00
Ni 1%
S 5%
C 47%
Ni 60%
S 9%
C 1%
σ*σS-CH3
α-σ*S-CH3
β-σ*Ni-S
ET
Ni 9%
S 30%
C 1%
Ni 6%
S 25%
C 30%
β-σ*S-CH3
dz2
α-σ*Ni-S
Ni-S
β-σNi-S
Ni 16%
S 4%
C 0%
Ni 11%
S 32%
C 16%
σNi-S
(Spin polarized)
σS-CH3
α-σNi-S

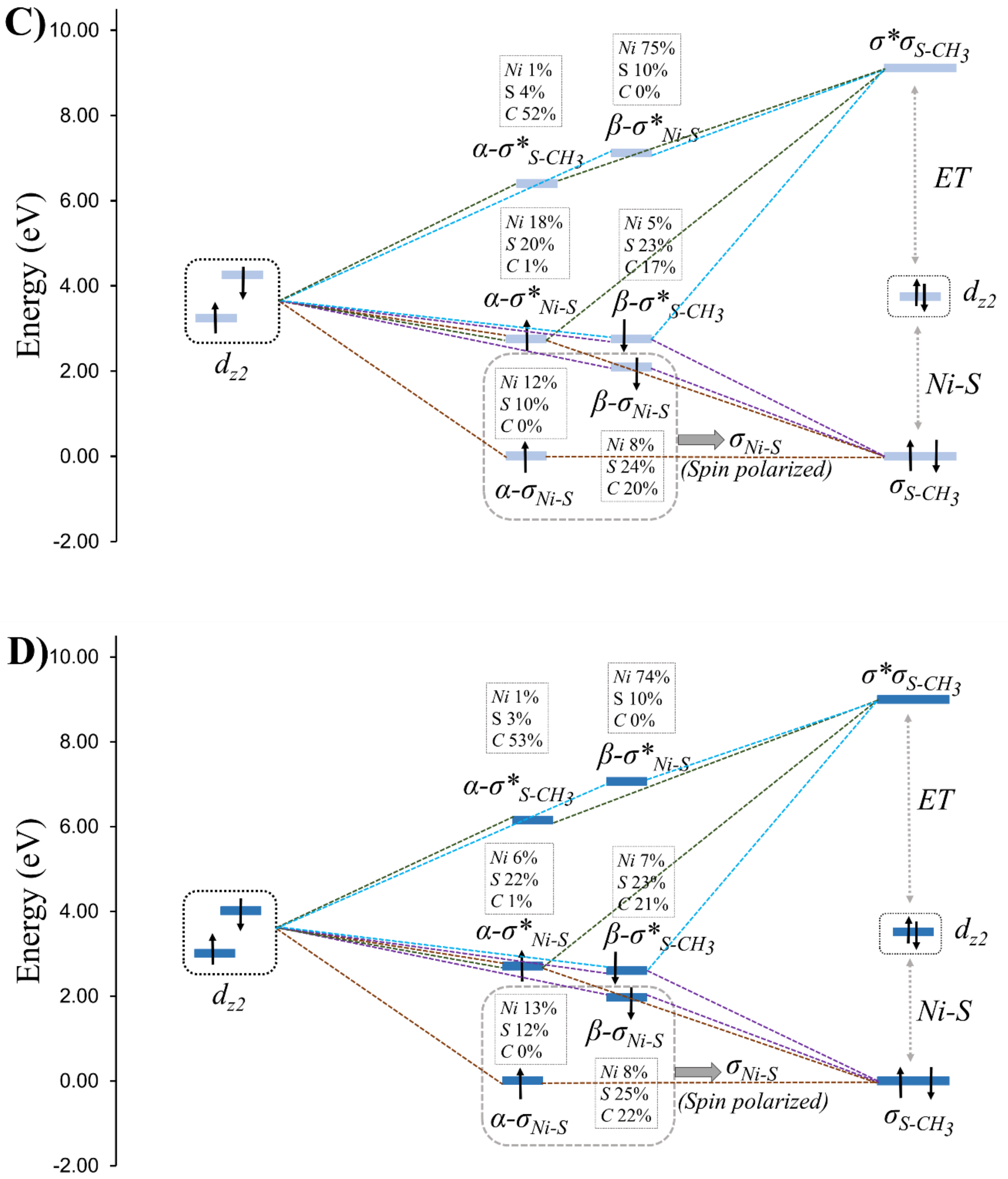

Figure S9: Representative MO diagrams and molecular orbitals α/β-d_{z^2}, α/β-$\sigma_{\mathrm{S-CH_3}}$ and α/β-$\sigma^*_{\mathrm{S-CH_3}}$ of RC, and α/β-$\sigma_{\mathrm{Ni-S}}$, α/β-$\sigma^*_{\mathrm{S-CH_3}}$, α-$\sigma^*_{\mathrm{Ni-S}}$ and β-$\sigma^*_{\mathrm{Ni-S}}$ of Int$_1$ are shown for **A-D**, respectively. In each plot, MO energies are shown relative to the $\sigma_{\mathrm{S-CH_3}}$ energy, which serves as a reference value of 0 eV.

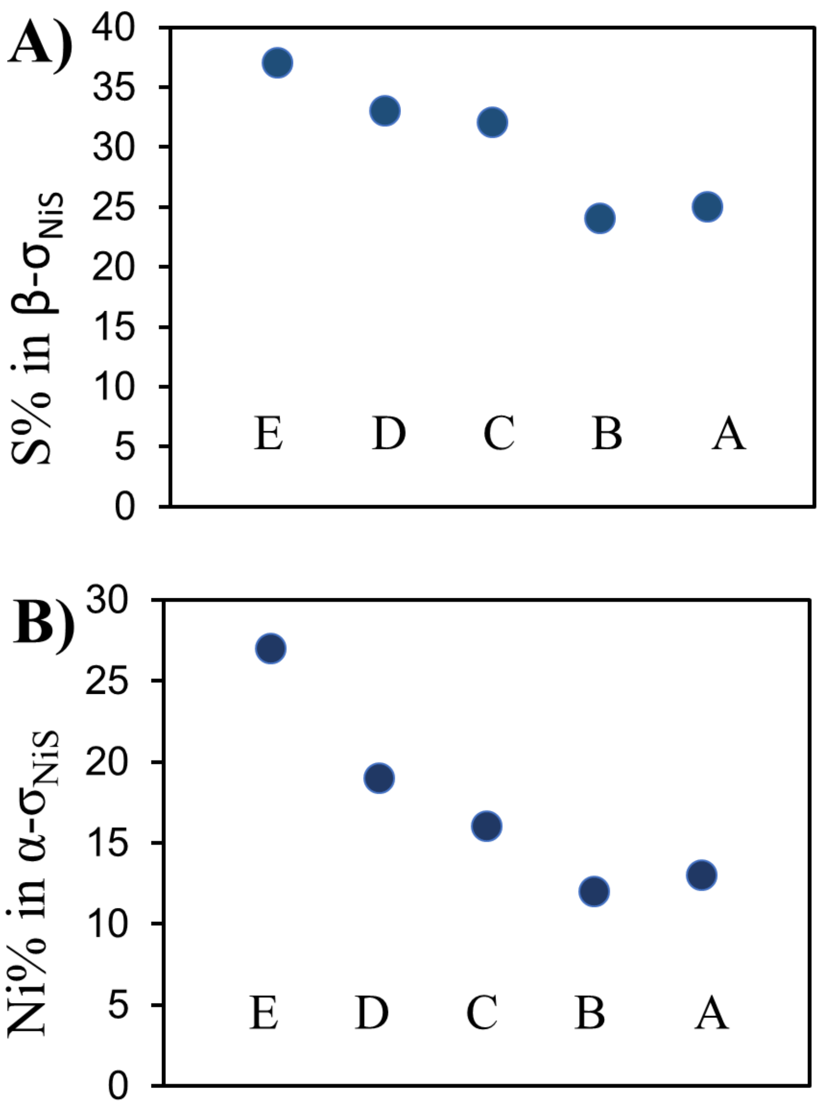

Figure S10: Change in the atomic composition in molecular orbitals key for Ni-S bond formation. Namely for **A)** and **B),** thiyl character on S in β-σ_{NiS} and Ni character in α-$\sigma_{Ni\text{-}S}$ is plotted for all **A-E** (from **Figure 5** and **Figure S9**).

Polarization of the macrocyclic ligand in systems A-E, in response to a point charge

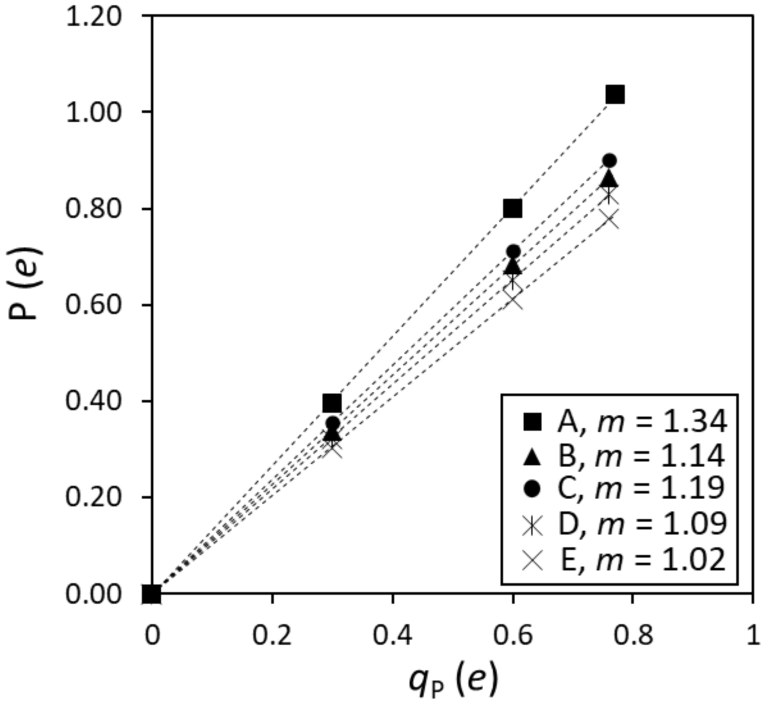

Figure S11: Polarization $P(q_P)$ of the macrocyclic ligands in the RC state of systems **A-E** from **Figure 3B**, calculated as $P(q_P) = \sum_{i=1}^{n} |q_i(q_P) - q_i(0)|$, where *n* is the number of atoms in the system, $q_i(q_P)$ is the charge of the *i*-th atom in response to the point charge q_P, and $q_i(0)$ is the charge of the *i*-th atom when $q_P = 0e$. All charges were integrated using the AIM formalism on the DFT-optimized electron density.

..

Charges for the coordinating N atoms and the Ni cation in the RC state of systems A-E

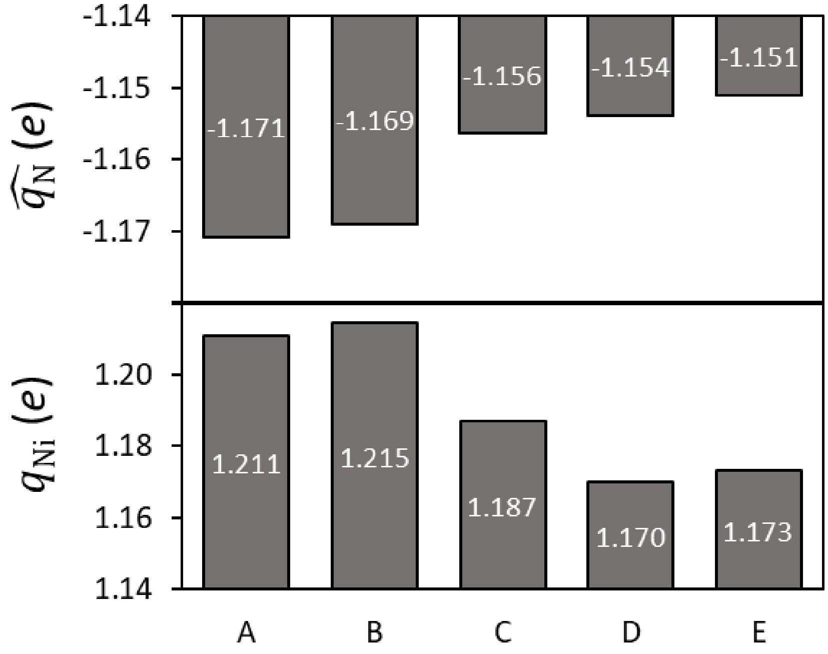

Figure S12: Average charge of all four coordinating nitrogen atoms in the macrocyclic ligand ($\widehat{q_N}$, *top*), and charge of the nickel cation (q_{Ni}, bottom), calculated for all models **A**-**E** in the Int_1 state, calculated through integration of the DFT-optimized electron density using the AIM formalism.

Calculation of Ni^{II}-macrocyclic ligand interaction for systems A-E using Coulomb's law

The interaction between the Ni^{II} ion and the macrocyclic ligand in systems **A-E** in the Int1 state was calculated by means of Coulomb's law, namely:

$$\Delta E_{Ni-ligand} = 4 \times \frac{1}{4\pi \times \varepsilon_0 \times \varepsilon_{solvent}} \times \frac{\widehat{q_N} \times q_{Ni}}{\widehat{r_{Ni-N}}} \times F \times N_A \qquad \text{(S1)}$$

Where ε_0 is the vacuum permittivity, $\varepsilon_{solvent}$ is the dielectric constant of the solvent (set to 4 in our calculations), $\widehat{q_N}$ is the average charge of the coordinating nitrogen atoms in the macrocycle and q_{Ni} is the charge of the nickel ion, integrated using the AIM protocol (in Coulombs), $\widehat{r_{Ni-N}}$ is the average Ni-N distance (in meters), F is Faraday's constant and N_A is Avogadro's number. The prefactor "4" reflects that there are four Ni-N interactions being considered. All values used to evaluate eq. S1 are condensed in **Table S6**.

Table S6: Values employed to evaluate the electrostatic interaction between the Ni^{II} ion and the macrocyclic ligand in systems **A-E** by means of Coulomb's law, as stated in eq. S1.

System	$\widehat{q_N}$ (e)	q_{Ni} (e)	$\widehat{r_{Ni-N}}$ (Å)	$\Delta E_{Ni-ligand}$ (kcal mol^{-1})
**A**	-1.171	1.211	2.113	-222.7
**B**	-1.169	1.215	2.096	-224.8
**C**	-1.156	1.187	2.127	-214.2
**D**	-1.153	1.171	2.130	-210.4
**E**	-1.151	1.173	2.131	-210.4

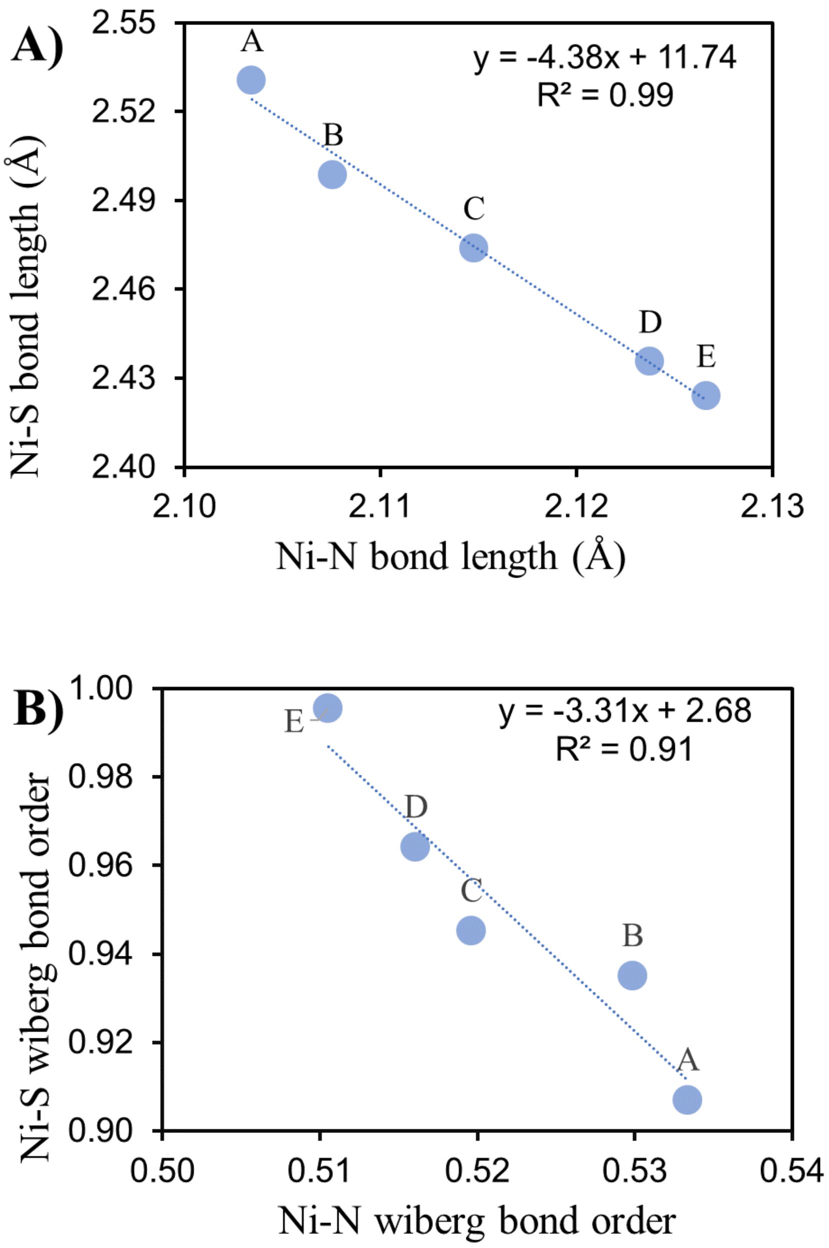

Figure S13: A) Correlation plot between the Ni–N (averaged Ni–N using all four coordinating nitrogen atoms of the porphyrin-like skeleton) and Ni–S_{CoM} bond length of coenzyme M. **B)** Correlation plot between the averaged Ni–N bond order and the Ni–S bond order derived from Wiberg bond order analysis in Lowdin orthogonalized basis (Ref. 8) using thermodynamic cycle for Ni–S bond formation step ($\Delta G'_{NiS}$) in **Figure 4** for the set of **A**-**E**.

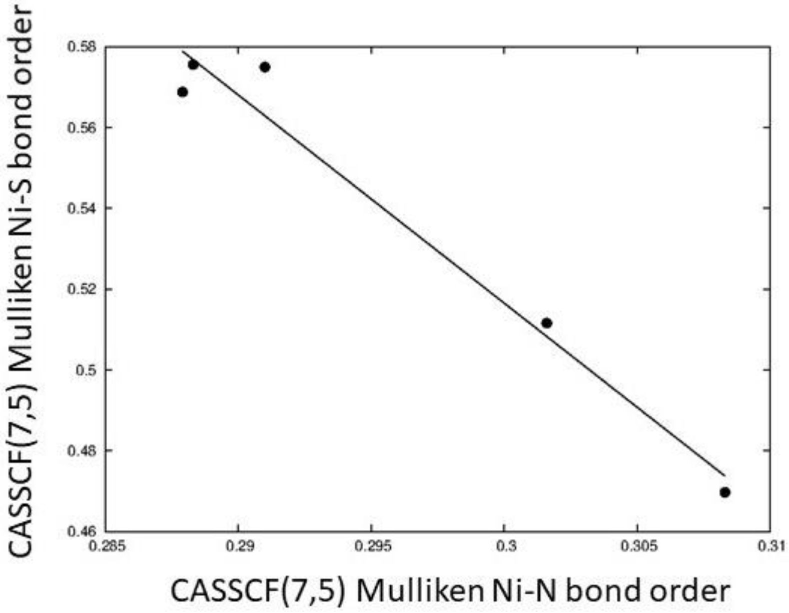

Figure S14: Correlation plot between the averaged Mulliken Ni–N bond order and the Mulliken Ni–S bond order as obtained from the CASSCF(7-in-5) calculations on top of the DFT-optimized Int_1 structures of all the models **A-E** (Int_1 – the product of **step 1**). Details on the CASSCF method are described in the Computation Section in the main text.

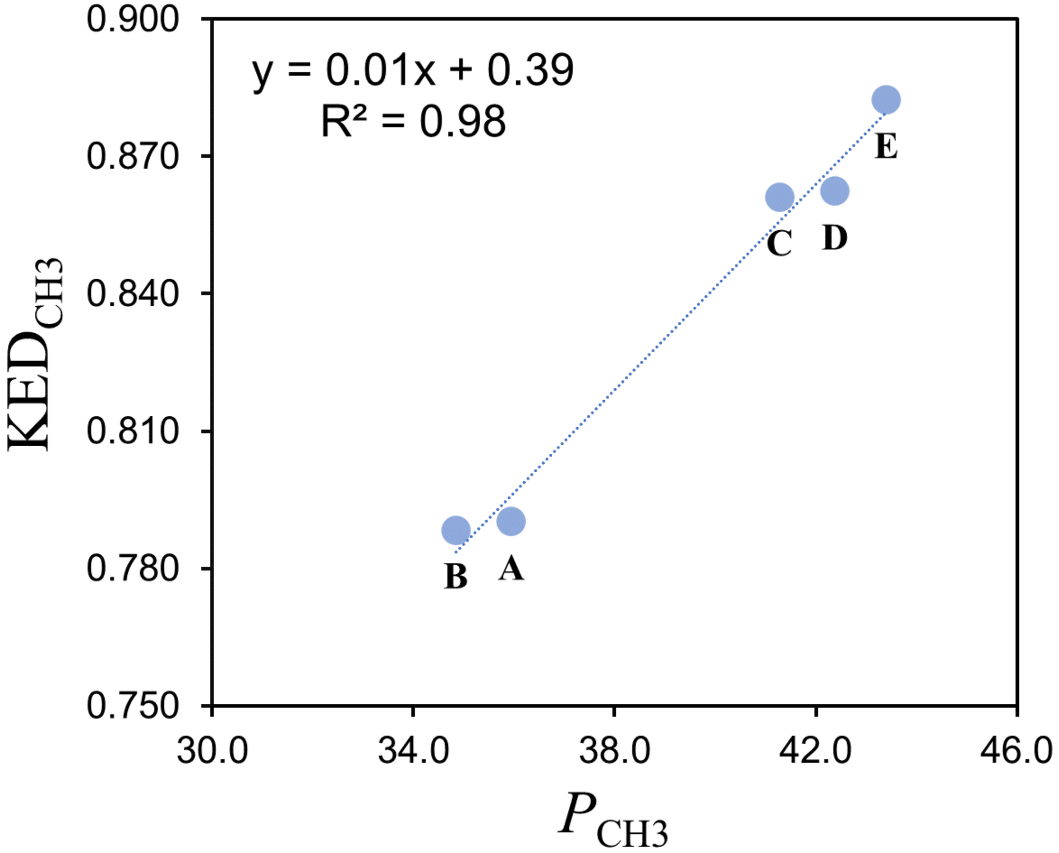

Figure S15: Fraction of kinetic energy of the reactive mode stored on the transient CH3 fragment (KED_{CH3}) and contribution of CH3 in % to the composition of the singly occupied β-$\sigma^*_{S\text{-}CH3}$ orbital (P_{CH3}) for the set of **A**–**E**. All values calculated at the respective TS_1 structures.

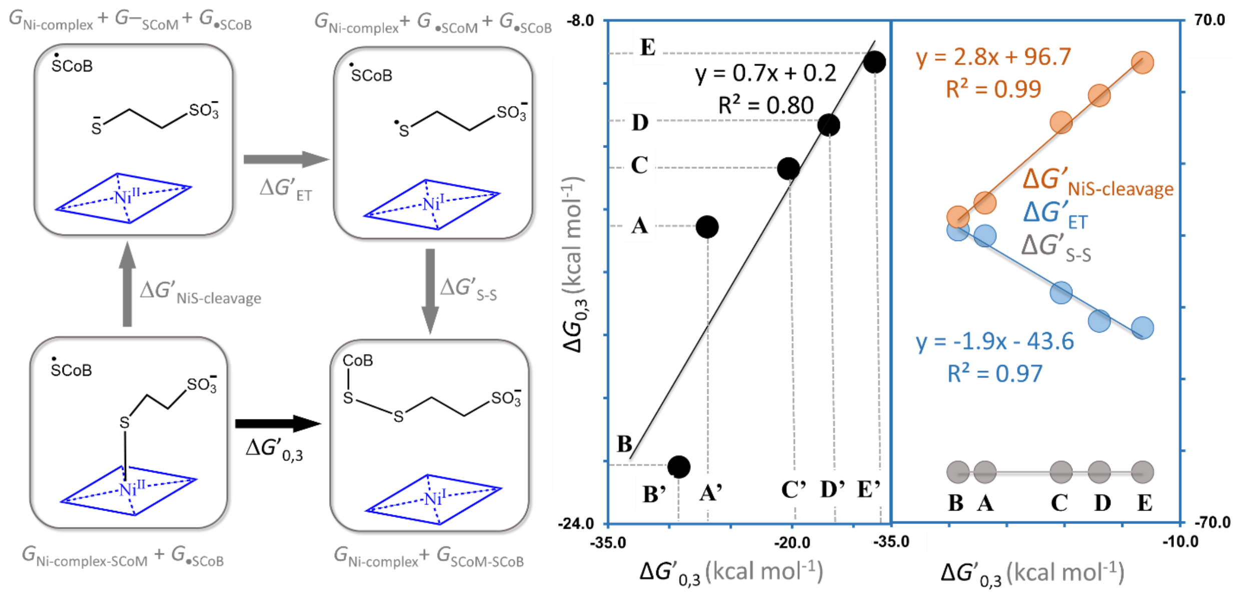

Figure S16. Thermodynamic cycle for **step 3** of the catalytic cycle from **Figure 1**. This step involves the formation of the heterodisulfide product in concomitant with the one electron transfer from SCoB to Ni(II) resulting in the generation of Ni(I) center. The whole process is dissected into three steps: (*i*) the homolytic cleavage of the Ni–S bond ($\Delta G'_{NiS\text{-cleavage}}$), (*ii*) one electron transfer from the anionic S-CoM to Ni^{II} center ($\Delta G'_{ET}$) and (*iii*) the S–S bond formation ($\Delta G'_{S\text{-}S}$) between the substrates S-CoM and S-CoB. Note that Δ*G*'s were calculated from Gibbs free energies of individual species as indicated in the figure and using eq. 1; the sum of three sequential Δ*G*'s steps equals to $\Delta G'_{0,3}$, which is analogous to free energy of reaction $\Delta G_{0,3}$ from **Figure 3B**. The labels **A'**, **B'**, **C'**, **D'** and **E'** are used in parallel to labels for the full models **A-E**; the "prime" symbol refers to the fact that the model consists of individual (infinitely separated) moieties such as the $Ni^{I/II}$-complex, H_3C–SCoM, $^{-/\bullet}$SCoM, $CH_3^{\bullet}$, the Ni^{II}–SCoM complex.

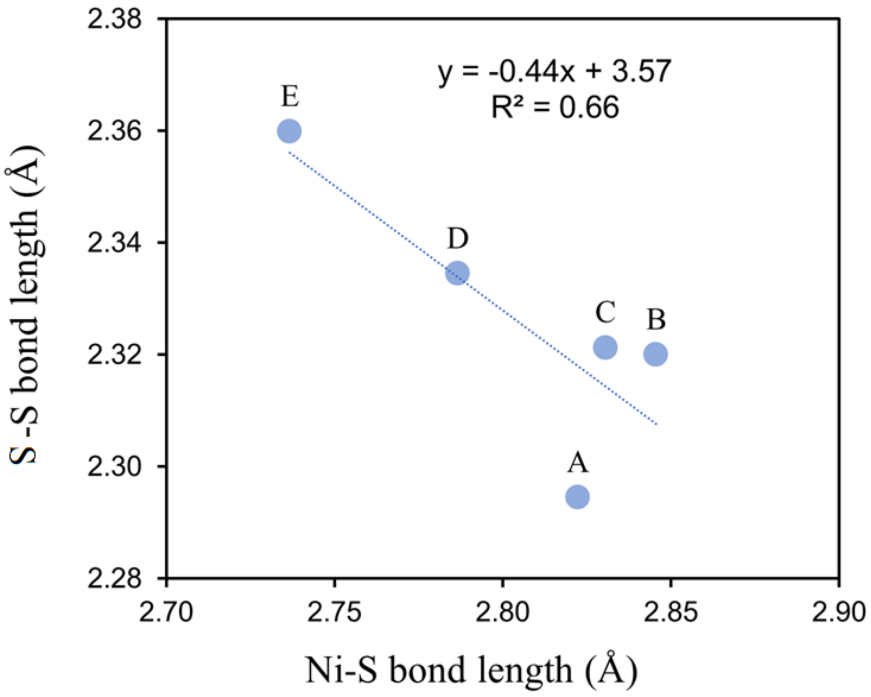

Figure S17. Correlation plot between the Ni–S and S–S bond lengths at TS_3.

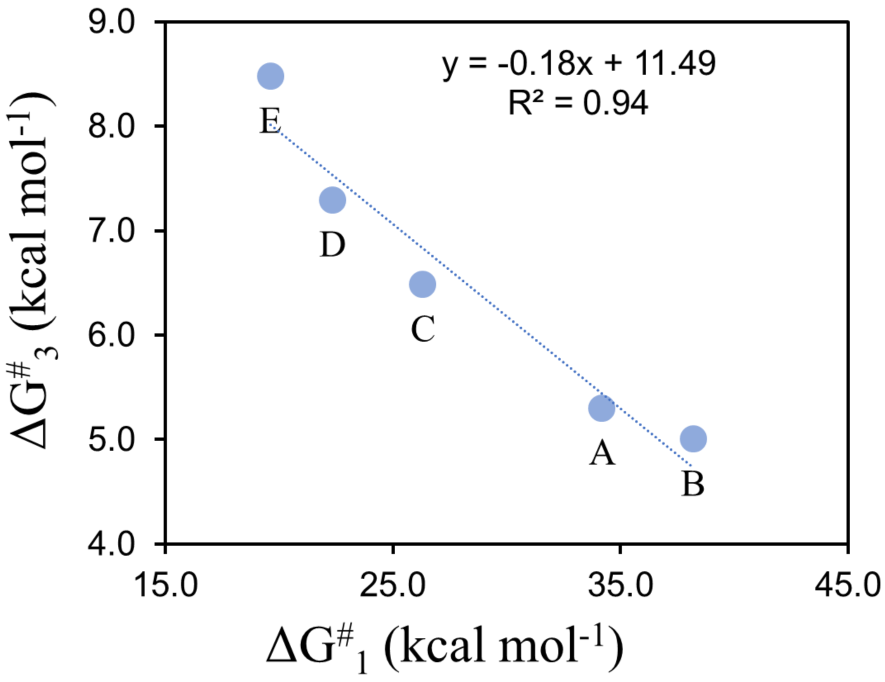

Figure S18. Correlation plot between the free-energy barrier of the rate determining **step 1** ($\Delta G_1^{\neq}$) and **step 3** ($\Delta G_3^{\neq}$). Note that $\Delta G_3^{\neq}$ was calculated as the difference in free energies between TS_3 and Int_3.

References:

1. Kelly, C. P.; Cramer, C. J.; Truhlar, D. G. Aqueous Solvation Free Energies of Ions and Ion-Water Clusters Based on an Accurate Value for the Absolute Aqueous Solvation Free Energy of the Proton. *J. Phys. Chem. B* **2006**, *110*, 16066−16081.

2. Romańczyk, P. P.; Rotko, G.; Kurek, S. S. The Redox Potential of the Phenyl Radical/Anion Couple and the Effect Thereon of the Lithium Cation: A Computational Study. *Electrochem. Commun*. **2014**, *48*, 21−23.

3. Namazian, M.; Li, C. Y.; Coote, M. L. Benchmark Calculations of Absolute Reduction Potential of Ferricinium/Ferrocene Couple in Nonaqueous Solutions. *J. Chem. Theory Comput.* **2010**, *6*, 2721−2725.

4. Holliger, C.; Pierik, A. J.; Reijerse, E. J.; Hagen, W. R. A spectro-electrochemical study of factor F430 nickel (II/I) from methanogenic bacteria in aqueous solution. *J. Am. Chem. Soc*. **1993**, *115*, 5651 – 5656.

5. Zilbermann, I.; Maimon, E.; Cohen, H.; Meyerstein, D. Redox chemistry of nickel complexes in aqueous solutions. *Chem. Rev*. **2005**, *105*, 2609 – 2626.

6. Jaun, B.; Pfaltz, A. Coenzyme F430 from methanogenic bacteria: reversible one-electron reduction of F430 pentamethyl ester to the nickel (I) form. *J. Chem. Soc., Chem. Commun.* **1986**, *513*, 1327 – 1329.

7. Kratky, C.; Fässler, A.; Pfaltz, A.; Kräutler, B.; Jaun, B.; Eschenmoser, A. Chemistry of corphinoids: structural properties of corphinoid nickel (II) complexes related to coenzyme F430. *J. Chem. Soc., Chem. Commun.* **1984**, *20*, 1368 – 1371.

8. Lu, T.; Chen, F. Multiwfn: a multifunctional wavefunction analyzer. *J. Comput. Chem.* **2012**, 33, 580 – 592.

This dataset contains all geometry coordinates of the calculated structures, divided into the following parts:

i) Reactants (RC), Transition States (TS_1, TS_2 and TS_3), Intermediates (Int_1, Int_2 and Int_3) and products (PC) for **A**-**E**, optimized at B3LYP-D3/BS1/CPCM(ε_r = 4) level of theory as shown in **Figure 3B**. Basis set BS1 specifies LANL2TZ ECP basis set for Ni, 6-311G* for key atoms involved in reactivity (the SCH_3 and SH groups of the H_3CS-CoM and truncated CoB-SH substrates, respectively) and 6-31G* for the rest.

ii) Reactant, transition state and product ($RC_{uncatalyzed}$, $TS_{uncatalyzed}$ and $PC_{uncatalyzed}$) optimized for uncatalyzed reaction, optimized at B3LYP-D3/BS1/CPCM(water) as shown in **Figure 3A**. Basis set BS1 specifies 6-311G* for key atoms involved in reactivity (the SCH_3 and SH groups of the H_3C-SCoM and truncated CoB-SH substrates, respectively) and 6-31G* for the rest.

iii) Complexes considered in proposed thermodynamic cycle of the first catalytic step involving the formation of the transient methyl radical coupled with one one-electron oxidation of the Ni^I center and Ni–thiolate bond formation, calculated at B3LYP-D3/BS1/CPCM(ε_r = 4) level of theory as shown in **Figure 4**. Basis set BS1 specifies LANL2TZ ECP basis set for Ni, 6-311G* for key atoms involved in reactivity (the SCH_3 and SH groups of the H_3C-SCoM and truncated CoB-SH substrates, respectively) and 6-31G* for the rest.

a) CH_3-SCoM, CH_3-radical, SCoM-radical and SCoM-anion.
b) Ni^I, Ni^{II} and Ni^{II}-SCoM for **A**.
c) Ni^I, Ni^{II} and Ni^{II}-SCoM for **B**.
d) Ni^I, Ni^{II} and Ni^{II}-SCoM for **C**.
e) Ni^I, Ni^{II} and Ni^{II}-SCoM for **D**.
f) Ni^I, Ni^{II} and Ni^{II}-SCoM for **E**.

iv) Complexes considered in proposed thermodynamic cycle for **step 3** of the catalytic cycle from **Figure S14**. This step involves the formation of the heterodisulfide product, concomitant with the one electron transfer from SCoB to Ni(II) resulting in the generation of Ni(I). All are optimized at B3LYP-D3/BS1/CPCM(ε_r = 4) level of theory as shown in **Figure S14**. Basis set BS1 specifies

LANL2TZ ECP basis set for Ni, 6-311G* for key atoms involved in reactivity (the SCH_3 and SH groups of the H_3CS-CoM and truncated CoB-SH substrates, respectively) and 6-31G* for the rest.

a) SCoB-radical, SCoM-anion, SCoM-radical and CoMS-SCoB.
b) Ni^{II}-SCoM, Ni^{II}, Ni^{I} and for **A**.
c) Ni^{II}-SCoM, Ni^{II}, Ni^{I} and for **B**.
d) Ni^{II}-SCoM, Ni^{II}, Ni^{I} and for **C**.
e) Ni^{II}-SCoM, Ni^{II}, Ni^{I} and for **D**.
f) Ni^{II}-SCoM, Ni^{II}, Ni^{I} and for **E**.

v) Reactive mode composition factor analysis of TS_1 for **A-E** calculated at B3LYP-D3/BS1/CPCM(ε_r = 4) level of theory as shown in **Figure 7**. Basis set BS1 specifies LANL2TZ ECP basis set for Ni, 6-311G* for key atoms involved in reactivity (the SCH_3 and SH groups of the H_3CS-CoM and truncated CoB-SH substrates, respectively) and 6-31G* for the rest.

vi) Reactants and transition states (TS1) of charged carboxylic groups for **A-E** calculated at B3LYP-D3/BS1/CPCM(ε_r = 4) level of theory. Basis set BS1 specifies LANL2TZ ECP basis set for Ni, 6-311G* for key atoms involved in reactivity (the SCH_3 and SH groups of the H_3C-SCoM and truncated CoB-SH substrates, respectively) and 6-31G* for the rest. The total number of charges for **A-E** are -10, -8, -7, -7 and -6 respectively.

vii) Optimized structures of experimentally well-defined synthetic F430 complexes *viz*. **Coenzyme-F430**, **Pentamethyl ester F430M** and **F430 model complex** calculated in B3LYP-D3/BS1/CPCM(solvents are specified in **Figure S2)**. Basis set BS1 specifies LANL2TZ ECP basis set for Ni and 6-31G* for the rest.

a) Optimized reduced (doublet) and oxidized (triplet and singlet) state of **Coenzyme-F430** in aqueous medium in *p*H = 10.4.
b) Optimized reduced (doublet) and oxidized (triplet and singlet) state of **Pentamethyl ester F430M** in solvent THF.
c) Optimized reduced (doublet) and oxidized (triplet and singlet) state of **Pentamethyl ester F430M** in solvent DMF.

d) Optimized reduced (doublet) and oxidized (triplet and singlet) state of **Pentamethyl ester F430M** in solvent CH_3CN.

e) Optimized reduced (doublet) and oxidized (triplet and singlet) state of **F430 model complex** in solvent CH_3CN.

viii) Optimized structure of reduced and oxidized states of cofactor **A**-**E** in aqueous solvent and epsilon=4 as mentioned in **Table S3**, calculated in B3LYP-D3/BS1/CPCM. Basis set BS1 specifies LANL2TZ ECP basis set for Ni and 6-31G* for the rest.

a) Optimized reduced (doublet) and oxidized (triplet and singlet) state of **A** in aqueous solvent.

b) Optimized reduced (doublet) and oxidized (triplet and singlet) state of **A** in epsilon=4.

c) Optimized reduced (doublet) and oxidized (triplet and singlet) state of **B** in aqueous solvent.

d) Optimized reduced (doublet) and oxidized (triplet and singlet) state of **B** in epsilon=4.

e) Optimized reduced (doublet) and oxidized (triplet and singlet) state of **C** in aqueous solvent.

f) Optimized reduced (doublet) and oxidized (triplet and singlet) state of **C** in epsilon=4.

g) Optimized reduced (doublet) and oxidized (triplet and singlet) state of **D** in aqueous solvent.

h) Optimized reduced (doublet) and oxidized (triplet and singlet) state of **D** in epsilon=4.

i) Optimized reduced (doublet) and oxidized (triplet and singlet) state of **E** in aqueous solvent.

j) Optimized reduced (doublet) and oxidized (triplet and singlet) state of **E** in epsilon=4.

ix) Single point structure of reactants **A**-**E** without presence of Ni as mentioned in **Figure 3B**, calculated at B3LYP-D3/6-311++G**/CPCM(ε_r = 4) level of theory. Structure **A**-**E** are prepared by removing Ni atom on top of doing a single point calculation from optimized structure of reactants **A**-**E** in **Figure 3B**.

x) Single point structure of reactants of cofactor **A**-**E** without presence of Ni as mentioned in **Figure 4,** calculated at B3LYP-D3/6-311++G**/CPCM(ε_r = 4) level of theory. Structure **A**-**E** are prepared by removing Ni atom on top of doing a single point calculation from optimized structure of reactants **A**-**E** in **Figure 4**.

---

i) Reactants (RC), Transition States (TS_1, TS_2 and TS_3), intermediates (Int_1, Int_2 and Int_3) and products (PC) for **A-E.**

A
RC
A-RC-opt.gjf.log

Temperature 298.150 Kelvin. Pressure 1.00000 Atm.

Zero-point correction= 1.431092 (Hartree/Particle)
Thermal correction to Energy= 1.532063
Thermal correction to Enthalpy= 1.533007
Thermal correction to Gibbs Free Energy= 1.271808
Sum of electronic and zero-point Energies= -5896.894480
Sum of electronic and thermal Energies= -5896.793510
Sum of electronic and thermal Enthalpies= -5896.792566
Sum of electronic and thermal Free Energies= -5897.053764

6	0.619009000	-2.022940000	-6.328040000
6	0.755678000	-2.254984000	-4.841090000
6	0.787857000	-3.544001000	-4.285897000
6	0.875896000	-1.168498000	-3.962995000
6	0.939243000	-3.739687000	-2.910471000
6	1.021446000	-1.342910000	-2.592027000
6	1.060051000	-2.636718000	-2.057048000
8	1.189519000	-2.875771000	-0.720580000
6	1.572113000	5.331956000	-1.220919000
6	0.015816000	2.598140000	4.626207000
6	3.758021000	-6.594428000	1.594156000
6	4.208799000	-0.922446000	-4.729518000
7	1.452825000	2.072124000	-0.832367000
6	1.515296000	6.596836000	-2.081931000
6	0.770801000	1.765398000	5.678251000
7	0.271604000	1.311038000	1.720124000
8	4.653755000	-6.911119000	2.353981000
8	3.578996000	-0.254462000	-5.528367000
7	3.340889000	-0.116961000	-0.605162000
8	2.216160000	8.860022000	-2.189897000
8	1.886210000	2.857549000	7.549867000
8	2.153499000	7.932395000	-0.141809000
8	2.789666000	2.999333000	5.492100000
6	3.095061000	1.419339000	-2.515322000
6	-0.264600000	3.288937000	0.409497000
6	0.206090000	-0.486030000	3.378037000
6	3.963312000	-2.077228000	0.741071000
28	1.762819000	0.557360000	0.515028000
6	2.067791000	2.198839000	-2.061625000
6	-0.422984000	2.448750000	1.502338000

6	1.191125000	-1.353627000	2.834935000
6	4.133010000	-1.225117000	-0.346815000
6	1.372379000	3.286824000	-2.896089000
6	-1.507738000	2.670704000	2.569145000
6	1.574820000	-2.637075000	3.380313000
6	5.038534000	-1.460483000	-1.445124000
6	0.741070000	4.145163000	-1.760808000
6	-0.934935000	1.781566000	3.712403000
6	2.687711000	-3.052233000	2.675322000
6	4.770477000	-0.479653000	-2.376765000
6	0.611569000	3.104443000	-0.659018000
6	-0.130377000	0.762568000	2.919477000
6	2.955547000	-2.026187000	1.701617000
6	3.713458000	0.340273000	-1.829942000
6	0.239445000	2.561829000	-3.672026000
6	0.800705000	-3.447669000	4.388725000
6	6.035308000	-2.579000000	-1.530132000
6	-0.770679000	3.442872000	-4.372439000
6	-3.962401000	1.963770000	2.956223000
6	-0.175887000	-4.320299000	3.608519000
6	7.267799000	-2.323162000	-0.648670000
8	-0.728514000	4.649533000	-4.517952000
8	-4.546752000	2.917886000	3.433886000
8	0.059713000	-5.466812000	3.260828000
6	-2.804181000	2.029849000	1.987134000
6	3.411183000	-4.364436000	2.758096000
8	-1.281780000	-3.652300000	3.283012000
6	2.304089000	4.006971000	-3.876124000
6	-1.753722000	4.144221000	2.920970000
6	2.945777000	-5.326729000	1.651193000
6	5.282756000	-0.393494000	-3.790750000
6	1.993703000	7.825523000	-1.342182000
6	1.833642000	2.579323000	6.370387000
7	2.008726000	-1.014936000	1.796682000
8	3.372735000	-7.388507000	0.564871000
8	3.989274000	-2.238219000	-4.530988000
6	-10.078950000	-2.957404000	-1.736527000
6	-10.010700000	-2.031301000	-2.955916000
6	-9.578223000	-0.592916000	-2.623496000
6	-8.193651000	-0.413916000	-1.979444000
6	-6.991960000	-0.750530000	-2.875984000
16	-6.486661000	-2.530686000	-2.949417000
6	-1.570870000	-1.282512000	0.175300000
8	-4.154142000	-3.063033000	-0.479675000
16	-2.000638000	-0.144428000	-1.209606000
6	-2.659836000	-1.608257000	1.202752000

8	-4.641952000	-3.160408000	1.972219000
16	-3.601202000	-3.135338000	0.910105000
8	-2.589425000	-4.249053000	1.049666000
6	-8.411609000	-0.383922000	2.701462000
6	-7.397832000	0.230280000	1.765956000
6	-6.495964000	-0.605201000	1.090560000
6	-7.337128000	1.604849000	1.505742000
6	-5.577178000	-0.094595000	0.180617000
6	-6.400550000	2.136094000	0.613538000
6	-5.511372000	1.284825000	-0.049627000
8	-4.593985000	1.831761000	-0.907915000
6	4.531915000	6.163750000	1.876469000
6	4.208027000	4.896953000	2.668613000
6	3.872131000	3.706166000	1.765850000
7	3.387782000	2.613180000	2.411555000
8	4.082011000	3.730386000	0.551549000
1	0.118110000	-2.864215000	-6.820362000
1	0.036929000	-1.117492000	-6.533244000
1	0.687510000	-4.410700000	-4.936011000
1	0.864886000	-0.161573000	-4.363387000
1	0.962361000	-4.738792000	-2.485496000
1	1.106158000	-0.486607000	-1.935457000
1	1.384708000	-2.044292000	-0.243822000
1	-7.929342000	-1.078079000	3.401260000
1	-9.151108000	-0.973359000	2.141724000
1	-6.488986000	-1.673756000	1.284351000
1	-8.020573000	2.278635000	2.018253000
1	-4.913731000	-0.770717000	-0.346254000
1	-6.334984000	3.206330000	0.441497000
1	-3.859548000	1.186228000	-1.033303000
1	1.172024000	5.587836000	-0.233886000
1	2.606847000	5.018278000	-1.045426000
1	-0.555736000	3.378658000	5.140183000
1	0.748275000	3.103830000	3.987526000
1	2.093674000	6.513721000	-3.004854000
1	0.482551000	6.799380000	-2.400540000
1	0.091969000	1.375912000	6.441438000
1	1.263548000	0.918048000	5.188730000
1	3.454511000	1.611798000	-3.519760000
1	-0.889305000	4.175316000	0.367418000
1	-0.279207000	-0.811927000	4.293188000
1	-0.241515000	4.529078000	-2.051765000
1	-1.718474000	1.314614000	4.320082000
1	-0.316873000	1.892448000	-3.003548000
1	0.680597000	1.915031000	-4.439950000
1	1.461839000	-4.102069000	4.963356000

1	0.249162000	-2.806209000	5.083012000
1	6.360047000	-2.728083000	-2.564625000
1	5.575253000	-3.527012000	-1.225622000
1	7.769016000	-1.392507000	-0.944800000
1	-3.135493000	2.613118000	1.122411000
1	-2.575692000	1.025050000	1.635689000
1	3.243863000	-4.839326000	3.730838000
1	4.494626000	-4.217089000	2.671934000
1	3.161538000	4.446721000	-3.358176000
1	1.765678000	4.789289000	-4.415526000
1	2.693504000	3.302498000	-4.619755000
1	-2.422030000	4.232414000	3.781216000
1	-0.819242000	4.669031000	3.137059000
1	-2.238440000	4.659509000	2.085396000
1	1.896298000	-5.598406000	1.812609000
1	2.982807000	-4.841847000	0.668486000
1	6.186867000	-0.999888000	-3.909792000
1	5.515971000	0.630512000	-4.094730000
1	-9.078427000	-3.146692000	-1.333899000
1	-10.520346000	-3.926242000	-1.999049000
1	-9.329147000	-2.464142000	-3.699274000
1	-10.999357000	-1.986748000	-3.434657000
1	-9.617590000	0.011067000	-3.542396000
1	-10.324916000	-0.158693000	-1.941998000
1	-8.087882000	0.640576000	-1.688983000
1	-8.123523000	-0.977449000	-1.044639000
1	-6.113870000	-0.181894000	-2.561055000
1	-7.198533000	-0.471985000	-3.915726000
1	-5.771099000	-2.581059000	-1.801299000
1	-0.754450000	-0.764833000	0.685319000
1	-1.142830000	-2.191818000	-0.253812000
1	-2.192535000	-1.751589000	2.180285000
1	-3.400340000	-0.816793000	1.304157000
1	5.260134000	5.939548000	1.091592000
1	3.642678000	6.580090000	1.394240000
1	5.067486000	4.602106000	3.287605000
1	3.378512000	5.064882000	3.368065000
1	-8.974247000	0.345108000	3.299602000
1	1.593381000	-1.888986000	-6.815475000
1	4.963115000	6.920084000	2.541055000
6	-2.523957000	-1.282311000	-2.537097000
1	-2.778822000	-0.659781000	-3.395531000
1	-1.693426000	-1.934373000	-2.804465000
1	-3.380769000	-1.867714000	-2.221462000
1	2.493652000	9.618292000	-1.640664000
1	3.444219000	3.519550000	5.995504000

1	3.935232000	-8.185698000	0.599236000
1	-1.775991000	-4.100072000	2.528214000
1	3.145403000	-2.475287000	-4.965353000
1	-2.412730000	3.311304000	-5.298145000
1	3.100773000	2.668428000	3.381532000
1	2.986329000	1.859630000	1.847343000
1	-5.106867000	0.710357000	3.791652000
1	-10.683219000	-2.516669000	-0.933653000
1	4.635442000	-2.928607000	0.797092000
1	6.978167000	-2.179865000	0.399158000
6	8.283426000	-3.439349000	-0.711035000
8	8.221470000	-4.425783000	-1.417453000
8	9.320005000	-3.217429000	0.134312000
1	9.929518000	-3.974125000	0.037454000
8	-4.292453000	0.687582000	3.253332000
8	-1.793667000	2.697221000	-4.858358000

TS1
A-TS_1-opt.gjf.log

Temperature 298.150 Kelvin. Pressure 1.00000 Atm.

Zero-point correction= 1.427053 (Hartree/Particle)
Thermal correction to Energy= 1.529252
Thermal correction to Enthalpy= 1.530196
Thermal correction to Gibbs Free Energy= 1.264013
Sum of electronic and zero-point Energies= -5896.826779
Sum of electronic and thermal Energies= -5896.724580
Sum of electronic and thermal Enthalpies= -5896.723636
Sum of electronic and thermal Free Energies= -5896.989819

6	-0.191835000	7.578036000	0.765143000
6	-0.012988000	6.346137000	-0.087045000
6	0.876568000	6.325220000	-1.171706000
6	-0.767911000	5.192354000	0.154495000
6	1.014524000	5.195687000	-1.975057000
6	-0.646359000	4.053292000	-0.638414000
6	0.255935000	4.045139000	-1.711363000
8	0.430233000	2.966480000	-2.517611000
6	-0.596649000	-2.481446000	4.477033000
6	2.526929000	-5.088159000	-0.431221000
6	7.169024000	2.933833000	-3.033208000
6	1.362296000	4.046515000	4.619285000
7	-0.458342000	-0.590323000	1.805724000
6	-1.179834000	-2.897807000	5.827685000
6	3.817808000	-5.117486000	-1.276246000

7	0.733373000	-2.335670000	-0.314945000
8	6.998653000	3.636704000	-4.009862000
8	0.687264000	3.535477000	5.491081000
7	1.271832000	1.614126000	0.909032000
8	-0.742295000	-4.076749000	7.837310000
8	5.413459000	-6.642300000	-0.245040000
8	0.906166000	-4.065390000	6.304288000
8	5.541680000	-4.435098000	0.194326000
6	-0.296513000	1.628057000	2.812065000
6	-0.875659000	-2.966435000	1.403883000
6	2.344272000	-2.346213000	-2.151135000
6	3.019320000	2.250740000	-0.698215000
28	0.796352000	-0.257012000	0.136546000
6	-0.780856000	0.349122000	2.759330000
6	-0.079883000	-3.221971000	0.291182000
6	2.758122000	-0.988006000	-2.137711000
6	2.219130000	2.492123000	0.415918000
6	-1.788454000	-0.225998000	3.768049000
6	-0.027460000	-4.596039000	-0.394416000
6	3.694109000	-0.392496000	-3.058163000
6	2.233205000	3.695377000	1.210688000
6	-1.588490000	-1.757840000	3.531107000
6	1.317774000	-4.474344000	-1.181470000
6	3.905435000	0.906909000	-2.634366000
6	1.274330000	3.520701000	2.185932000
6	-0.973529000	-1.778105000	2.135647000
6	1.484765000	-2.963713000	-1.277895000
6	3.086029000	1.089434000	-1.465709000
6	0.696523000	2.214044000	1.979150000
6	-3.210977000	0.217298000	3.274829000
6	4.355843000	-1.094876000	-4.215202000
6	3.155226000	4.861226000	1.022722000
6	-4.329719000	-0.309120000	4.137528000
6	-2.542521000	-4.301515000	-0.993670000
6	5.572238000	-1.861391000	-3.729662000
6	4.502184000	4.637976000	1.726759000
8	-4.759511000	0.212581000	5.148687000
8	-3.169111000	-3.341987000	-1.394593000
8	6.638937000	-1.369248000	-3.415112000
6	-1.165771000	-4.696948000	-1.465658000
6	4.844904000	1.924970000	-3.210634000
8	5.341546000	-3.192819000	-3.637219000
6	-1.555894000	0.279392000	5.198472000
6	-0.104348000	-5.774967000	0.586316000
6	6.205808000	1.904981000	-2.495980000
6	0.866787000	4.494120000	3.259364000

6	-0.211879000	-3.728072000	6.639368000
6	4.990794000	-5.519492000	-0.423547000
7	2.388913000	-0.077770000	-1.195999000
8	8.302515000	2.992536000	-2.290024000
8	2.695593000	4.249180000	4.762577000
6	-10.525549000	3.440760000	-2.770323000
6	-10.088584000	3.852593000	-1.355219000
6	-9.651392000	2.677908000	-0.462473000
6	-8.537412000	1.757121000	-0.987058000
6	-7.101292000	2.293904000	-0.959967000
16	-6.721273000	3.472874000	-2.346567000
6	-0.582974000	-0.431268000	-3.001935000
8	-0.242171000	-1.456870000	-5.880401000
16	-1.086014000	0.433647000	-1.447754000
6	-1.668505000	-1.279882000	-3.646766000
8	-2.188570000	-3.001302000	-5.570289000
16	-0.990632000	-2.357477000	-4.951790000
8	-0.098235000	-3.331733000	-4.240208000
6	-8.650003000	-1.602546000	-2.389679000
6	-7.306624000	-1.407756000	-1.729146000
6	-6.183973000	-1.041990000	-2.483765000
6	-7.140928000	-1.526062000	-0.340850000
6	-4.946848000	-0.796777000	-1.889097000
6	-5.912793000	-1.279367000	0.273622000
6	-4.802356000	-0.911391000	-0.500870000
8	-3.627516000	-0.686877000	0.143371000
6	3.994386000	-3.268820000	4.569313000
6	4.062743000	-3.307742000	3.041819000
6	3.470055000	-2.069901000	2.366335000
7	3.844584000	-1.888761000	1.071915000
8	2.712697000	-1.302149000	2.963218000
1	-0.671781000	8.389933000	0.200064000
1	-0.836202000	7.365412000	1.626045000
1	1.481369000	7.204976000	-1.384432000
1	-1.461820000	5.174977000	0.992634000
1	1.717722000	5.177363000	-2.803132000
1	-1.211940000	3.157674000	-0.408254000
1	-0.012010000	2.175717000	-2.107117000
1	-8.542385000	-1.690661000	-3.476625000
1	-9.312008000	-0.746853000	-2.202811000
1	-6.272647000	-0.948459000	-3.563977000
1	-7.992056000	-1.811877000	0.275091000
1	-4.086601000	-0.542129000	-2.493776000
1	-5.790110000	-1.379256000	1.348274000
1	-2.887797000	-0.474939000	-0.482852000
1	-0.256673000	-3.390509000	3.970372000

1	0.303232000	-1.871756000	4.612931000
1	2.302804000	-6.115597000	-0.130293000
1	2.699497000	-4.511640000	0.485217000
1	-1.474541000	-2.045354000	6.446305000
1	-2.093298000	-3.492610000	5.691270000
1	3.715898000	-5.841442000	-2.089750000
1	4.018533000	-4.134750000	-1.703306000
1	-0.644646000	2.248987000	3.629518000
1	-1.436379000	-3.805076000	1.801632000
1	2.766175000	-2.967920000	-2.930727000
1	-2.540942000	-2.297502000	3.546734000
1	1.239379000	-4.933507000	-2.172654000
1	-3.354187000	-0.107891000	2.241833000
1	-3.257068000	1.309171000	3.315914000
1	3.662507000	-1.774344000	-4.718274000
1	4.710514000	-0.362885000	-4.947293000
1	2.695816000	5.776991000	1.409371000
1	3.323294000	5.047854000	-0.042556000
1	4.345305000	4.467939000	2.799598000
1	-0.926386000	-4.062209000	-2.322857000
1	-1.204211000	-5.735733000	-1.817384000
1	4.413186000	2.929872000	-3.142158000
1	5.007162000	1.738523000	-4.278013000
1	-0.545714000	0.045645000	5.545179000
1	-2.281794000	-0.146788000	5.895886000
1	-1.674790000	1.366100000	5.246208000
1	0.092682000	-6.718594000	0.064179000
1	0.618808000	-5.669420000	1.400507000
1	-1.100051000	-5.849046000	1.026660000
1	6.085767000	2.069564000	-1.418372000
1	6.666542000	0.915250000	-2.606014000
1	-0.221591000	4.584478000	3.321783000
1	1.278467000	5.484480000	3.040902000
1	-9.653346000	3.280442000	-3.414578000
1	-11.143757000	4.214583000	-3.239200000
1	-9.282604000	4.594845000	-1.428379000
1	-10.920540000	4.360145000	-0.847927000
1	-9.353988000	3.066310000	0.522389000
1	-10.536327000	2.048683000	-0.286765000
1	-8.526117000	0.859758000	-0.356560000
1	-8.772781000	1.408732000	-1.999079000
1	-6.408996000	1.455647000	-1.066760000
1	-6.894548000	2.792712000	-0.007520000
1	-5.412130000	3.147996000	-2.481628000
1	0.250640000	-1.073857000	-2.727951000
1	-0.212586000	0.309893000	-3.715317000

1	-2.148421000	-1.941692000	-2.922425000
1	-2.436536000	-0.666332000	-4.126425000
1	4.540770000	-2.407373000	4.967908000
1	2.960480000	-3.211820000	4.919605000
1	5.096312000	-3.442200000	2.701175000
1	3.509189000	-4.179928000	2.665408000
1	-9.194115000	-2.491522000	-2.044337000
1	0.743234000	7.993949000	1.162132000
1	4.445426000	-4.184336000	4.966949000
6	-2.891363000	1.928069000	-2.499799000
1	-3.614782000	1.845337000	-1.699917000
1	-2.340403000	2.854697000	-2.572825000
1	-3.133410000	1.427583000	-3.427877000
1	-0.065382000	-4.605422000	8.301856000
1	6.255277000	-4.758341000	0.777010000
1	8.875929000	3.664193000	-2.706447000
1	6.149112000	-3.598126000	-3.265452000
1	2.948797000	3.879170000	5.629892000
1	-5.517240000	-1.768937000	4.314184000
1	4.376580000	-2.603762000	0.591982000
1	3.343569000	-1.215121000	0.495952000
1	-3.933601000	-4.766912000	0.183036000
1	-11.088797000	2.499076000	-2.776592000
1	3.661019000	3.069267000	-1.010749000
1	4.996657000	3.735034000	1.348088000
6	5.449050000	5.802513000	1.570799000
8	5.190301000	6.874858000	1.060777000
8	6.671911000	5.525760000	2.088840000
1	7.219133000	6.323826000	1.958271000
8	-3.064237000	-5.140824000	-0.060735000
8	-4.824104000	-1.492181000	3.683688000

Int_1
A-Int_1-opt.gjf.log
Temperature 298.150 Kelvin. Pressure 1.00000 Atm.

Zero-point correction= 1.429103 (Hartree/Particle)
Thermal correction to Energy= 1.530042
Thermal correction to Enthalpy= 1.530986
Thermal correction to Gibbs Free Energy= 1.275233
Sum of electronic and zero-point Energies= -5896.853107
Sum of electronic and thermal Energies= -5896.752168
Sum of electronic and thermal Enthalpies= -5896.751224
Sum of electronic and thermal Free Energies= -5897.006977

6	-0.406442000	7.528048000	-1.123325000
6	-0.085708000	6.162969000	-1.688311000

6	0.917336000	5.987014000	-2.653710000
6	-0.796247000	5.025937000	-1.282993000
6	1.193038000	4.734041000	-3.197871000
6	-0.527968000	3.762718000	-1.809140000
6	0.470125000	3.604875000	-2.782448000
8	0.771828000	2.409508000	-3.346419000
6	-2.472966000	-0.704897000	4.350434000
6	2.034556000	-4.967323000	1.567729000
6	7.028279000	1.752116000	-1.295092000
6	1.720090000	5.223461000	2.849429000
7	-0.477050000	0.243584000	1.516446000
6	-3.827030000	-0.437409000	5.013039000
6	3.476193000	-5.124496000	1.086835000
7	0.672986000	-2.234821000	0.328023000
8	6.662083000	2.774873000	-1.851407000
8	0.872842000	5.153214000	3.717928000
7	1.581204000	1.809046000	0.071827000
8	-5.180101000	-0.864900000	6.910296000
8	4.199913000	-5.912867000	3.280672000
8	-3.153377000	-1.848157000	6.878520000
8	5.691442000	-5.766338000	1.596163000
6	-0.130330000	2.651251000	1.637443000
6	-1.228674000	-2.053066000	1.842443000
6	2.506718000	-3.061324000	-1.056118000
6	3.526942000	1.675934000	-1.440000000
28	0.933288000	-0.149695000	-0.064534000
6	-0.802854000	1.500937000	1.953259000
6	-0.336561000	-2.765072000	1.048262000
6	3.085678000	-1.841818000	-1.497483000
6	2.667375000	2.363039000	-0.584502000
6	-2.018470000	1.480512000	2.892665000
6	-0.395139000	-4.294649000	0.877007000
6	4.343402000	-1.750820000	-2.193987000
6	2.800253000	3.750071000	-0.210939000
6	-2.347292000	-0.053485000	2.960207000
6	1.073048000	-4.575757000	0.417142000
6	4.626172000	-0.406024000	-2.348332000
6	1.769249000	4.017537000	0.663889000
6	-1.273804000	-0.675486000	2.072538000
6	1.461391000	-3.231820000	-0.185262000
6	3.544195000	0.306187000	-1.713794000
6	1.022325000	2.792436000	0.818439000
6	-3.173437000	2.262952000	2.185204000
6	5.248896000	-2.920501000	-2.452807000
6	3.955531000	4.632151000	-0.575523000
6	-4.476744000	2.203833000	2.942334000

6	-2.728492000	-4.184999000	-0.422549000
6	5.787022000	-3.475162000	-1.138318000
6	5.116648000	4.387028000	0.406369000
8	-4.733602000	2.806368000	3.968560000
8	-3.302667000	-4.012556000	-1.479893000
8	6.135850000	-2.797076000	-0.182520000
6	-1.296800000	-4.671118000	-0.338852000
6	5.874679000	0.176645000	-2.950783000
8	5.829064000	-4.816280000	-1.114399000
6	-1.646289000	2.133825000	4.237188000
6	-0.826142000	-5.056473000	2.137518000
6	7.021033000	0.377849000	-1.930372000
6	1.459840000	5.315649000	1.359673000
6	-3.976646000	-1.132225000	6.345070000
6	4.432900000	-5.644254000	2.123216000
7	2.618881000	-0.600201000	-1.218696000
8	7.537671000	1.745878000	-0.050731000
8	3.046451000	5.199314000	3.131788000
6	-10.353844000	1.721790000	-3.057062000
6	-10.105104000	2.490101000	-1.750536000
6	-9.500672000	1.622630000	-0.629283000
6	-8.192560000	0.874251000	-0.938524000
6	-6.909980000	1.709399000	-0.990586000
16	-6.788004000	2.820331000	-2.475649000
6	-0.176735000	-1.423067000	-2.932127000
8	0.267221000	-3.138472000	-5.492490000
16	-0.720173000	0.024068000	-1.920417000
6	-1.320850000	-2.173682000	-3.596880000
8	-1.929842000	-4.264056000	-5.089477000
16	-0.712561000	-3.645863000	-4.483103000
8	-0.072889000	-4.502119000	-3.430293000
6	-7.890608000	-2.923402000	-1.852038000
6	-6.597883000	-2.332433000	-1.354686000
6	-5.651986000	-1.864905000	-2.277043000
6	-6.347628000	-2.104793000	0.005416000
6	-4.509255000	-1.177261000	-1.877037000
6	-5.207017000	-1.415383000	0.426453000
6	-4.283214000	-0.935132000	-0.516703000
8	-3.212953000	-0.232093000	-0.066555000
6	5.190013000	-1.855197000	4.379911000
6	4.651218000	-2.359564000	3.036234000
6	3.880226000	-1.232056000	2.358556000
7	4.435017000	-0.724283000	1.229036000
8	2.861814000	-0.765060000	2.877682000
1	-0.990976000	8.132718000	-1.831247000
1	-0.999741000	7.445617000	-0.205388000

1	1.497954000	6.846843000	-2.983776000
1	-1.566414000	5.119473000	-0.519224000
1	1.979132000	4.605182000	-3.936684000
1	-1.061331000	2.892864000	-1.444819000
1	0.292129000	1.673321000	-2.873088000
1	-7.785855000	-3.247164000	-2.894289000
1	-8.678273000	-2.154806000	-1.847900000
1	-5.812993000	-2.037221000	-3.338627000
1	-7.064041000	-2.449534000	0.748600000
1	-3.782750000	-0.836265000	-2.605814000
1	-5.034537000	-1.215663000	1.478341000
1	-2.453490000	-0.247497000	-0.722256000
1	-2.358742000	-1.788253000	4.247581000
1	-1.662716000	-0.384887000	5.011616000
1	1.701162000	-5.907660000	2.017937000
1	1.996062000	-4.206036000	2.355819000
1	-4.013443000	0.629973000	5.173433000
1	-4.647406000	-0.778507000	4.367102000
1	3.538342000	-5.784860000	0.212772000
1	3.873485000	-4.160968000	0.754154000
1	-0.485334000	3.564418000	2.102308000
1	-2.003056000	-2.635943000	2.321659000
1	2.985430000	-3.968806000	-1.408059000
1	-3.290362000	-0.229586000	2.433492000
1	1.102620000	-5.370004000	-0.337885000
1	-3.313401000	1.848074000	1.185353000
1	-2.888555000	3.316480000	2.103561000
1	4.756552000	-3.730919000	-2.999293000
1	6.117347000	-2.615956000	-3.050054000
1	3.670251000	5.689145000	-0.552045000
1	4.298967000	4.427642000	-1.594393000
1	4.831495000	4.725360000	1.411314000
1	-0.831489000	-4.359202000	-1.280544000
1	-1.346175000	-5.768950000	-0.389621000
1	5.667811000	1.136004000	-3.435639000
1	6.231900000	-0.493894000	-3.740011000
1	-0.786193000	1.627253000	4.685617000
1	-2.475146000	2.118694000	4.948218000
1	-1.364418000	3.180156000	4.084654000
1	-0.689951000	-6.134804000	1.991893000
1	-0.245048000	-4.751074000	3.012504000
1	-1.881400000	-4.883736000	2.354915000
1	7.002992000	-0.394173000	-1.155992000
1	7.991132000	0.294514000	-2.440993000
1	0.411464000	5.595085000	1.226950000
1	2.077002000	6.118279000	0.943572000

1	-9.416103000	1.537994000	-3.591627000
1	-11.021881000	2.273261000	-3.728084000
1	-9.466904000	3.360142000	-1.949989000
1	-11.057267000	2.894264000	-1.379439000
1	-9.348468000	2.244545000	0.265023000
1	-10.252502000	0.869107000	-0.351968000
1	-8.030522000	0.135533000	-0.142639000
1	-8.286467000	0.295754000	-1.865052000
1	-6.052273000	1.035433000	-1.036515000
1	-6.811327000	2.316808000	-0.084942000
1	-5.437044000	2.664008000	-2.669027000
1	0.361253000	-2.105769000	-2.277351000
1	0.527094000	-1.071596000	-3.690933000
1	-2.042294000	-2.543786000	-2.864891000
1	-1.846957000	-1.553181000	-4.330564000
1	5.852373000	-0.993775000	4.230151000
1	4.360581000	-1.533869000	5.017056000
1	5.459611000	-2.731298000	2.397306000
1	3.952624000	-3.183156000	3.213922000
1	-8.310377000	-3.777953000	-1.305128000
1	0.492607000	8.109242000	-0.880766000
1	5.763275000	-2.621563000	4.912583000
6	-3.463655000	2.083524000	-2.779867000
1	-3.470230000	1.638255000	-1.796641000
1	-3.036607000	3.066694000	-2.912343000
1	-3.591689000	1.442393000	-3.639665000
1	-5.204838000	-1.345194000	7.760011000
1	6.288117000	-6.065088000	2.308500000
1	7.577330000	2.681455000	0.259865000
1	6.078269000	-5.101566000	-0.205455000
1	3.131019000	5.079585000	4.097104000
1	-6.166327000	1.355614000	2.933244000
1	5.080106000	-1.289904000	0.684156000
1	3.847985000	-0.098907000	0.688504000
1	-4.222836000	-3.581463000	0.550658000
1	-10.803892000	0.744232000	-2.842797000
1	4.324732000	2.259181000	-1.885363000
1	5.317352000	3.315801000	0.491366000
6	6.386181000	5.093792000	0.021165000
8	6.495732000	6.065272000	-0.691487000
8	7.482559000	4.521375000	0.621377000
1	8.265243000	5.027055000	0.327569000
8	-3.358322000	-3.997420000	0.759452000
8	-5.366149000	1.352329000	2.372557000

TS_2

A-TS_2-opt.gjf.log

Temperature 298.150 Kelvin. Pressure 1.00000 Atm.

Zero-point correction= 1.428555 (Hartree/Particle)
Thermal correction to Energy= 1.528297
Thermal correction to Enthalpy= 1.529241
Thermal correction to Gibbs Free Energy= 1.277322
Sum of electronic and zero-point Energies= -5896.853338
Sum of electronic and thermal Energies= -5896.753597
Sum of electronic and thermal Enthalpies= -5896.752653
Sum of electronic and thermal Free Energies= -5897.004571

6	-0.331666000	7.522494000	-1.151307000
6	-0.020668000	6.154476000	-1.713897000
6	0.988165000	5.967134000	-2.671044000
6	-0.747607000	5.025743000	-1.314617000
6	1.254251000	4.711019000	-3.213021000
6	-0.489067000	3.759703000	-1.838387000
6	0.515154000	3.590399000	-2.803181000
8	0.807006000	2.390671000	-3.363267000
6	-2.467906000	-0.731465000	4.358156000
6	2.027077000	-4.979741000	1.537501000
6	7.020335000	1.768432000	-1.271046000
6	1.691966000	5.220331000	2.869756000
7	-0.486447000	0.233414000	1.520088000
6	-3.819661000	-0.470578000	5.028084000
6	3.468338000	-5.134187000	1.054587000
7	0.665167000	-2.237108000	0.317779000
8	6.654913000	2.791784000	-1.826763000
8	0.843593000	5.143294000	3.736584000
7	1.570494000	1.808365000	0.086342000
8	-5.161917000	-0.907998000	6.930709000
8	4.189277000	-5.953702000	3.237950000
8	-3.134828000	-1.889890000	6.882989000
8	5.681799000	-5.788428000	1.556123000
6	-0.148206000	2.642095000	1.648408000
6	-1.232022000	-2.066298000	1.838901000
6	2.498388000	-3.055066000	-1.072013000
6	3.521910000	1.684448000	-1.418589000
28	0.923944000	-0.149382000	-0.062162000
6	-0.815902000	1.488256000	1.961754000
6	-0.342541000	-2.772460000	1.036953000
6	3.078751000	-1.832698000	-1.502865000
6	2.658170000	2.366682000	-0.563778000
6	-2.027848000	1.460311000	2.905691000

6	-0.402352000	-4.300573000	0.854814000
6	4.337687000	-1.737254000	-2.196427000
6	2.786093000	3.753086000	-0.186646000
6	-2.350732000	-0.074977000	2.969554000
6	1.064793000	-4.579111000	0.390852000
6	4.622448000	-0.391481000	-2.338445000
6	1.750379000	4.016152000	0.683822000
6	-1.278513000	-0.690059000	2.075771000
6	1.453066000	-3.231008000	-0.202387000
6	3.540222000	0.316495000	-1.700046000
6	1.005941000	2.788818000	0.832589000
6	-3.188892000	2.241280000	2.206611000
6	5.241585000	-2.905911000	-2.465235000
6	3.939350000	4.639531000	-0.546711000
6	-4.487883000	2.174345000	2.970367000
6	-2.735737000	-4.174150000	-0.441645000
6	5.778219000	-3.474246000	-1.156018000
6	5.100349000	4.394731000	0.435335000
8	-4.743819000	2.777277000	3.996566000
8	-3.307973000	-3.983905000	-1.497005000
8	6.126517000	-2.806391000	-0.192834000
6	-1.306295000	-4.667250000	-0.362511000
6	5.872498000	0.195421000	-2.933514000
8	5.819939000	-4.815553000	-1.146075000
6	-1.652193000	2.110324000	4.250949000
6	-0.832475000	-5.071233000	2.110226000
6	7.014392000	0.394737000	-1.907683000
6	1.433943000	5.312498000	1.379620000
6	-3.961430000	-1.171848000	6.357600000
6	4.423434000	-5.670008000	2.084328000
7	2.612881000	-0.592828000	-1.213808000
8	7.527610000	1.760947000	-0.025805000
8	3.017944000	5.204515000	3.154444000
6	-10.281753000	1.777100000	-3.132427000
6	-10.053438000	2.552935000	-1.826141000
6	-9.510180000	1.680233000	-0.678326000
6	-8.208746000	0.901302000	-0.935732000
6	-6.907243000	1.709462000	-0.936749000
16	-6.681033000	2.790729000	-2.428214000
6	-0.183528000	-1.408454000	-2.936196000
8	0.264344000	-3.111813000	-5.505983000
16	-0.726444000	0.038450000	-1.923342000
6	-1.326395000	-2.153097000	-3.609577000
8	-1.933495000	-4.238547000	-5.109988000
16	-0.717056000	-3.622441000	-4.499798000
8	-0.078984000	-4.482406000	-3.448997000

6	-7.886123000	-2.875886000	-1.911650000
6	-6.596577000	-2.292819000	-1.397508000
6	-5.644503000	-1.812741000	-2.307036000
6	-6.352594000	-2.091083000	-0.032362000
6	-4.499822000	-1.139701000	-1.888942000
6	-5.209069000	-1.418161000	0.407129000
6	-4.277557000	-0.927878000	-0.522989000
8	-3.203214000	-0.243173000	-0.055037000
6	5.171498000	-1.897672000	4.382935000
6	4.634825000	-2.401477000	3.038169000
6	3.859808000	-1.276110000	2.361591000
7	4.417343000	-0.759030000	1.237712000
8	2.835368000	-0.818776000	2.877425000
1	-0.908095000	8.131488000	-1.862227000
1	-0.930093000	7.445728000	-0.236201000
1	1.581319000	6.820264000	-2.996089000
1	-1.522626000	5.127835000	-0.556814000
1	2.045190000	4.573161000	-3.944994000
1	-1.033984000	2.895776000	-1.477505000
1	0.316656000	1.662433000	-2.888777000
1	-7.770448000	-3.201523000	-2.952127000
1	-8.670403000	-2.103921000	-1.917220000
1	-5.802062000	-1.964039000	-3.372338000
1	-7.074843000	-2.445476000	0.700531000
1	-3.768255000	-0.787342000	-2.607321000
1	-5.040221000	-1.238155000	1.463250000
1	-2.443890000	-0.248594000	-0.711383000
1	-2.350776000	-1.814096000	4.250964000
1	-1.655494000	-0.411258000	5.016605000
1	1.693513000	-5.923253000	1.980858000
1	1.989460000	-4.224305000	2.331224000
1	-4.008268000	0.595547000	5.194181000
1	-4.641960000	-0.810977000	4.384265000
1	3.528830000	-5.783919000	0.172490000
1	3.868140000	-4.167626000	0.733842000
1	-0.506175000	3.552580000	2.116280000
1	-2.004316000	-2.652598000	2.317394000
1	2.976500000	-3.960210000	-1.430654000
1	-3.295453000	-0.253126000	2.446392000
1	1.092944000	-5.367725000	-0.370099000
1	-3.332155000	1.829497000	1.205972000
1	-2.908292000	3.296092000	2.127497000
1	4.748391000	-3.710288000	-3.019822000
1	6.110968000	-2.597170000	-3.058970000
1	3.650677000	5.695499000	-0.520194000
1	4.283813000	4.439580000	-1.566113000

1	4.813383000	4.729201000	1.441072000
1	-0.840443000	-4.352205000	-1.302827000
1	-1.360220000	-5.764617000	-0.418532000
1	5.665980000	1.156328000	-3.415492000
1	6.233949000	-0.471417000	-3.723910000
1	-0.788471000	1.605163000	4.693957000
1	-2.477899000	2.089794000	4.965523000
1	-1.374602000	3.158136000	4.100938000
1	-0.696800000	-6.148533000	1.956769000
1	-0.250616000	-4.772276000	2.986921000
1	-1.887536000	-4.899718000	2.329614000
1	6.992181000	-0.378026000	-1.134144000
1	7.986811000	0.310784000	-2.413778000
1	0.384517000	5.587452000	1.245473000
1	2.048182000	6.118030000	0.964907000
1	-9.334159000	1.583278000	-3.646034000
1	-10.932786000	2.326743000	-3.821284000
1	-9.383166000	3.401444000	-2.013190000
1	-11.004912000	2.987804000	-1.489547000
1	-9.376038000	2.304746000	0.217094000
1	-10.288955000	0.946179000	-0.423804000
1	-8.095655000	0.158867000	-0.134402000
1	-8.278848000	0.325380000	-1.865930000
1	-6.063684000	1.016328000	-0.927506000
1	-6.841205000	2.328070000	-0.035528000
1	-5.280714000	2.587879000	-2.545367000
1	0.348739000	-2.094939000	-2.280843000
1	0.525559000	-1.057097000	-3.690196000
1	-2.052036000	-2.524814000	-2.882655000
1	-1.847489000	-1.528043000	-4.342985000
1	5.839684000	-1.040566000	4.234794000
1	4.341147000	-1.570948000	5.016210000
1	5.444968000	-2.769465000	2.399164000
1	3.939245000	-3.227814000	3.214587000
1	-8.314328000	-3.727621000	-1.367004000
1	0.570305000	8.097379000	-0.904562000
1	5.736701000	-2.667820000	4.918751000
6	-3.564323000	2.193495000	-2.566417000
1	-3.512077000	1.692717000	-1.610047000
1	-3.171022000	3.198071000	-2.640539000
1	-3.547569000	1.575093000	-3.453026000
1	-5.181813000	-1.392511000	7.778152000
1	6.277304000	-6.099038000	2.264358000
1	7.565989000	2.696165000	0.286021000
1	6.068651000	-5.110148000	-0.239967000
1	3.101573000	5.084094000	4.119758000

1	-6.171016000	1.313314000	2.971694000
1	5.069384000	-1.317367000	0.693587000
1	3.828585000	-0.135819000	0.696401000
1	-4.229315000	-3.579491000	0.536615000
1	-10.739742000	0.802861000	-2.919637000
1	4.320149000	2.270713000	-1.859069000
1	5.304325000	3.323881000	0.517249000
6	6.368092000	5.106476000	0.053289000
8	6.475205000	6.080616000	-0.656105000
8	7.465798000	4.535222000	0.652131000
1	8.247175000	5.044090000	0.360359000
8	-3.366824000	-4.001395000	0.742059000
8	-5.373508000	1.314655000	2.407218000

Int_2
$A\text{-}Int_2\text{-opt.gjf.log}$

Temperature 298.150 Kelvin. Pressure 1.00000 Atm.

Zero-point correction= 1.429553 (Hartree/Particle)
Thermal correction to Energy= 1.532878
Thermal correction to Enthalpy= 1.533822
Thermal correction to Gibbs Free Energy= 1.264435
Sum of electronic and zero-point Energies= -5896.868112
Sum of electronic and thermal Energies= -5896.764787
Sum of electronic and thermal Enthalpies= -5896.763843
Sum of electronic and thermal Free Energies= -5897.033230

6	-0.021513000	7.160178000	2.458958000
6	0.085076000	6.150561000	1.342768000
6	0.962136000	6.323497000	0.261244000
6	-0.744739000	5.022052000	1.329399000
6	1.010914000	5.406406000	-0.788306000
6	-0.718460000	4.099647000	0.285119000
6	0.168281000	4.283855000	-0.784586000
8	0.246067000	3.417421000	-1.827541000
6	-0.227649000	-4.192360000	3.264352000
6	2.744286000	-4.614698000	-2.093540000
6	6.566356000	4.501262000	-1.787192000
6	0.697092000	1.995674000	5.921131000
7	-0.497880000	-1.487537000	1.536987000
6	-0.605195000	-5.155442000	4.389639000
6	4.106847000	-4.255160000	-2.726082000
7	0.877602000	-2.182416000	-1.021668000
8	6.141526000	5.521151000	-2.292413000
8	0.094706000	1.123064000	6.513581000

7	0.959347000	1.068120000	1.605095000
8	0.261572000	-6.818920000	5.841342000
8	5.799457000	-5.967827000	-2.354083000
8	1.575852000	-6.212371000	4.117358000
8	5.556159000	-4.300902000	-0.856428000
6	-0.605518000	0.200075000	3.299787000
6	-0.696758000	-3.547766000	0.228307000
6	2.526135000	-1.365449000	-2.623323000
6	2.623471000	2.434877000	0.422055000
28	0.692318000	-0.400746000	0.170482000
6	-0.908830000	-1.026971000	2.763477000
6	0.078329000	-3.264122000	-0.886032000
6	2.776824000	-0.081135000	-2.086822000
6	1.784248000	2.167225000	1.504316000
6	-1.801362000	-2.067867000	3.455039000
6	0.173894000	-4.170133000	-2.120156000
6	3.558145000	0.951663000	-2.734561000
6	1.618048000	3.010694000	2.671345000
6	-1.391497000	-3.354878000	2.672940000
6	1.585139000	-3.752517000	-2.650409000
6	3.613343000	2.018714000	-1.866486000
6	0.678749000	2.389369000	3.461527000
6	-0.860040000	-2.764141000	1.376304000
6	1.691410000	-2.333214000	-2.107372000
6	2.844925000	1.635817000	-0.700184000
6	0.289851000	1.170539000	2.778936000
6	-3.281260000	-1.677603000	3.102409000
6	4.144936000	0.854402000	-4.117890000
6	2.366386000	4.277297000	2.950650000
6	-4.294571000	-2.640544000	3.668863000
6	-2.260447000	-3.347948000	-2.781593000
6	5.431050000	0.051269000	-4.087158000
6	3.717314000	4.016536000	3.632518000
8	-4.782506000	-2.586826000	4.781132000
8	-2.864600000	-2.386531000	-3.210511000
8	6.502918000	0.456054000	-3.678564000
6	-0.855748000	-3.713911000	-3.212364000
6	4.338370000	3.316927000	-2.057272000
8	5.264910000	-1.215885000	-4.534283000
6	-1.613258000	-2.107143000	4.976336000
6	-0.004048000	-5.662140000	-1.816591000
6	5.806449000	3.209146000	-1.614753000
6	0.144000000	2.845663000	4.792725000
6	0.534874000	-6.089702000	4.732676000
6	5.233610000	-4.952114000	-2.011223000
7	2.343865000	0.359701000	-0.872868000

8	7.830654000	4.403369000	-1.306260000
8	1.990725000	2.295913000	6.188900000
6	-10.488834000	3.747767000	-1.438857000
6	-9.895914000	3.743755000	-0.024770000
6	-9.081728000	2.480637000	0.301110000
6	-7.853004000	2.193417000	-0.578315000
6	-6.655662000	3.139060000	-0.385965000
16	-6.791387000	4.807900000	-1.137359000
6	-0.418670000	0.182646000	-2.879756000
8	0.943366000	0.806385000	-5.555989000
16	-1.115133000	0.647619000	-1.238733000
6	-1.263240000	0.522122000	-4.094285000
8	-1.234969000	0.469491000	-6.739602000
16	-0.351751000	0.053120000	-5.610196000
8	-0.158048000	-1.431179000	-5.523325000
6	-8.574905000	-1.228757000	-2.257214000
6	-7.145639000	-1.296844000	-1.761949000
6	-6.176953000	-0.377669000	-2.201179000
6	-6.745549000	-2.251929000	-0.819955000
6	-4.870563000	-0.394396000	-1.717398000
6	-5.439395000	-2.290262000	-0.323416000
6	-4.491749000	-1.356408000	-0.766342000
8	-3.242015000	-1.444579000	-0.246662000
6	4.384642000	-4.194575000	3.602209000
6	3.758142000	-4.153920000	2.200573000
6	3.261551000	-2.755531000	1.851002000
7	3.719262000	-2.233561000	0.684827000
8	2.505922000	-2.142683000	2.610828000
1	-0.531659000	8.072181000	2.116025000
1	-0.622046000	6.758269000	3.283671000
1	1.623485000	7.187759000	0.241605000
1	-1.429859000	4.854767000	2.157853000
1	1.698775000	5.539678000	-1.618615000
1	-1.357342000	3.222990000	0.302556000
1	-0.249628000	2.581643000	-1.599218000
1	-8.619824000	-0.999361000	-3.328384000
1	-9.140502000	-0.442901000	-1.737082000
1	-6.452408000	0.374315000	-2.937681000
1	-7.468577000	-2.981047000	-0.458463000
1	-4.132692000	0.313494000	-2.081362000
1	-5.144173000	-3.018990000	0.426482000
1	-2.612606000	-0.786235000	-0.658727000
1	0.183679000	-4.794884000	2.447907000
1	0.588165000	-3.528320000	3.569799000
1	2.547579000	-5.673603000	-2.287994000
1	2.792518000	-4.487642000	-1.005454000

1	-0.908004000	-4.649172000	5.309783000
1	-1.461183000	-5.780564000	4.098067000
1	4.125975000	-4.558318000	-3.776256000
1	4.277459000	-3.177097000	-2.670868000
1	-1.027668000	0.425170000	4.272183000
1	-1.209789000	-4.501433000	0.237674000
1	3.015909000	-1.599316000	-3.560482000
1	-2.246070000	-4.013817000	2.491955000
1	1.621113000	-3.773508000	-3.745077000
1	-3.385489000	-1.614977000	2.015953000
1	-3.487689000	-0.696646000	3.538942000
1	3.417465000	0.418417000	-4.809433000
1	4.403668000	1.853482000	-4.480538000
1	1.769823000	4.936705000	3.589241000
1	2.525423000	4.836308000	2.024268000
1	3.572478000	3.465733000	4.570485000
1	-0.490927000	-2.847795000	-3.770722000
1	-0.944815000	-4.530985000	-3.941836000
1	3.844387000	4.121700000	-1.503309000
1	4.309482000	3.616858000	-3.110845000
1	-0.567263000	-2.284342000	5.240922000
1	-2.234665000	-2.882284000	5.432741000
1	-1.910321000	-1.155498000	5.427627000
1	0.256387000	-6.262548000	-2.695999000
1	0.620826000	-5.985133000	-0.978300000
1	-1.046298000	-5.875178000	-1.566545000
1	5.878250000	2.904734000	-0.563113000
1	6.317124000	2.427607000	-2.192130000
1	-0.946316000	2.768169000	4.832741000
1	0.421811000	3.889455000	4.969657000
1	-9.701313000	3.824181000	-2.195479000
1	-11.172606000	4.592979000	-1.581757000
1	-9.269397000	4.635467000	0.109223000
1	-10.709657000	3.824404000	0.710445000
1	-8.754904000	2.526113000	1.350489000
1	-9.755220000	1.612527000	0.230350000
1	-7.498218000	1.182862000	-0.337763000
1	-8.132187000	2.168296000	-1.638775000
1	-5.774966000	2.679711000	-0.856922000
1	-6.425346000	3.236722000	0.682274000
1	-3.929183000	4.315010000	-2.234593000
1	-0.237980000	-0.887430000	-2.874803000
1	0.548507000	0.675993000	-2.976323000
1	-2.200393000	-0.040501000	-4.101714000
1	-1.476556000	1.592683000	-4.169514000
1	5.157908000	-3.424660000	3.710850000

1	3.623893000	-4.030173000	4.369029000
1	4.466958000	-4.514206000	1.448192000
1	2.888729000	-4.822608000	2.182206000
1	-9.098610000	-2.179501000	-2.091477000
1	0.929055000	7.492507000	2.895912000
1	4.858030000	-5.168154000	3.768965000
6	-3.314945000	3.443015000	-2.462550000
1	-3.386587000	2.717066000	-1.654598000
1	-2.269836000	3.728979000	-2.571065000
1	-3.663329000	2.981712000	-3.387603000
1	1.029185000	-7.403654000	5.991561000
1	6.260697000	-4.811188000	-0.413186000
1	8.257874000	5.267323000	-1.462655000
1	6.122864000	-1.672013000	-4.432485000
1	2.294314000	1.672365000	6.876253000
1	-5.241311000	-4.224156000	3.257379000
1	4.294076000	-2.781932000	0.058813000
1	3.321659000	-1.366685000	0.331072000
1	-3.683339000	-3.820358000	-1.646016000
1	-11.047498000	2.822214000	-1.626747000
1	3.136663000	3.390271000	0.441817000
1	4.361498000	3.386554000	3.007378000
6	4.455625000	5.295090000	3.948830000
8	4.019181000	6.422785000	3.826454000
8	5.704619000	5.049347000	4.414876000
1	6.110184000	5.917042000	4.605204000
8	-2.825419000	-4.214569000	-1.907301000
8	-4.605373000	-3.639303000	2.801322000

Int_3
A-Int_3-opt.gjf.log

Temperature 298.150 Kelvin. Pressure 1.00000 Atm.

Zero-point correction= 1.434499 (Hartree/Particle)
Thermal correction to Energy= 1.535642
Thermal correction to Enthalpy= 1.536586
Thermal correction to Gibbs Free Energy= 1.281283
Sum of electronic and zero-point Energies= -5896.898866
Sum of electronic and thermal Energies= -5896.797723
Sum of electronic and thermal Enthalpies= -5896.796779
Sum of electronic and thermal Free Energies= -5897.052082

6	1.477482000	7.616363000	-0.731850000
6	1.418888000	6.221715000	-1.302457000
6	2.346373000	5.757426000	-2.245551000

6	0.360231000	5.370311000	-0.958964000
6	2.221631000	4.494189000	-2.822903000
6	0.214703000	4.108462000	-1.528902000
6	1.151362000	3.660660000	-2.469961000
8	1.068767000	2.438358000	-3.061457000
6	-2.762643000	-1.343634000	4.465283000
6	1.156076000	-5.249313000	0.827201000
6	6.798432000	1.146664000	-1.123419000
6	2.096798000	4.464197000	3.853702000
7	-0.735217000	0.013850000	1.802182000
6	-3.931711000	-1.002159000	5.391273000
6	2.556341000	-5.455280000	0.249370000
7	0.016220000	-2.278442000	0.076192000
8	6.543769000	2.294647000	-1.450577000
8	1.292791000	4.288064000	4.748016000
7	1.455303000	1.571523000	0.568260000
8	-5.202348000	-1.736369000	7.250746000
8	3.255721000	-6.768762000	2.185321000
8	-3.653562000	-3.170703000	6.464462000
8	4.685759000	-6.446009000	0.471323000
6	-0.058952000	2.282372000	2.382591000
6	-1.814256000	-2.168527000	1.679210000
6	1.697387000	-3.046303000	-1.525634000
6	3.332560000	1.497596000	-1.029524000
28	0.577629000	-0.231998000	0.124905000
6	-0.834957000	1.165365000	2.540115000
6	-1.030922000	-2.821393000	0.731414000
6	2.410941000	-1.844788000	-1.767478000
6	2.588528000	2.104851000	-0.023546000
6	-1.889604000	1.041456000	3.650891000
6	-1.235910000	-4.298843000	0.330878000
6	3.638636000	-1.769261000	-2.513062000
6	2.898030000	3.377675000	0.578581000
6	-2.549735000	-0.342205000	3.312216000
6	0.191761000	-4.626257000	-0.215152000
6	4.098687000	-0.468289000	-2.405942000
6	1.916828000	3.610114000	1.517181000
6	-1.644220000	-0.886916000	2.206235000
6	0.678580000	-3.249660000	-0.634233000
6	3.155423000	0.222703000	-1.566049000
6	1.037128000	2.465747000	1.498620000
6	-2.891287000	2.233396000	3.526982000
6	4.368599000	-2.962414000	-3.063344000
6	4.143918000	4.168439000	0.320038000
6	-4.062086000	2.136777000	4.468707000
6	-3.642874000	-4.080758000	-0.841310000

6	4.920875000	-3.813437000	-1.924180000
6	5.297944000	3.604782000	1.169794000
8	-4.048460000	2.406476000	5.656089000
8	-4.372969000	-4.173206000	-1.810449000
8	5.470092000	-3.373165000	-0.924383000
6	-2.177056000	-4.441625000	-0.899168000
6	5.384782000	0.072087000	-2.967218000
8	4.729255000	-5.129104000	-2.107957000
6	-1.163116000	1.082167000	5.012237000
6	-1.696714000	-5.204285000	1.481383000
6	6.591180000	-0.065048000	-2.007153000
6	1.765970000	4.809115000	2.415131000
6	-4.212291000	-2.094429000	6.395948000
6	3.477057000	-6.289365000	1.095709000
7	2.132121000	-0.641341000	-1.202015000
8	7.350711000	0.836543000	0.062671000
8	3.432225000	4.333038000	4.047044000
6	-9.448156000	4.089507000	-2.875048000
6	-8.026120000	3.967787000	-2.301718000
6	-7.099233000	2.957968000	-3.003497000
6	-5.624819000	3.164611000	-2.602482000
6	-4.667219000	2.098940000	-3.159964000
16	-2.890240000	2.538683000	-2.907720000
6	-0.892150000	-0.709142000	-2.887467000
8	-0.949077000	-2.007431000	-5.756272000
16	-1.099438000	0.600893000	-1.602074000
6	-2.182886000	-1.137013000	-3.567935000
8	-3.251028000	-2.851228000	-5.253463000
16	-1.898425000	-2.518987000	-4.719130000
8	-1.298405000	-3.608039000	-3.880774000
6	-7.970125000	-1.031803000	-2.047396000
6	-6.789984000	-0.554372000	-1.238938000
6	-5.543868000	-1.181166000	-1.370570000
6	-6.882962000	0.548130000	-0.377650000
6	-4.417896000	-0.693881000	-0.709847000
6	-5.759969000	1.060665000	0.273300000
6	-4.510993000	0.467381000	0.064764000
8	-3.406073000	1.044099000	0.616553000
6	5.086054000	-3.077006000	3.989015000
6	4.338226000	-3.412898000	2.689080000
6	3.566265000	-2.197862000	2.179676000
7	4.007829000	-1.646154000	1.024583000
8	2.624051000	-1.730691000	2.829447000
1	1.065365000	8.349015000	-1.441680000
1	0.866242000	7.687256000	0.176012000
1	3.183973000	6.391315000	-2.530773000

1	-0.373157000	5.700423000	-0.225673000
1	2.952503000	4.130154000	-3.539553000
1	-0.607506000	3.463901000	-1.239536000
1	0.394260000	1.886407000	-2.586854000
1	-7.642773000	-1.436192000	-3.011579000
1	-8.665677000	-0.209381000	-2.253261000
1	-5.441163000	-2.048659000	-2.016301000
1	-7.841752000	1.046071000	-0.247851000
1	-3.452086000	-1.171046000	-0.819055000
1	-5.827083000	1.939935000	0.906502000
1	-2.616833000	0.758716000	0.099169000
1	-2.964328000	-2.330535000	4.040393000
1	-1.845894000	-1.459979000	5.051140000
1	0.759098000	-6.213359000	1.160110000
1	1.218564000	-4.604545000	1.712091000
1	-3.775793000	-0.073791000	5.949237000
1	-4.850254000	-0.843336000	4.811390000
1	2.512779000	-5.922483000	-0.742222000
1	3.054942000	-4.493015000	0.095306000
1	-0.246090000	3.101486000	3.067898000
1	-2.643354000	-2.737397000	2.073218000
1	2.056512000	-3.926953000	-2.046805000
1	-3.524650000	-0.157441000	2.844733000
1	0.137865000	-5.307023000	-1.072486000
1	-3.249486000	2.280878000	2.499189000
1	-2.365155000	3.160772000	3.771123000
1	3.740784000	-3.590852000	-3.701903000
1	5.226558000	-2.645040000	-3.668522000
1	3.996025000	5.227315000	0.554825000
1	4.417278000	4.126068000	-0.737765000
1	5.096297000	3.780727000	2.234664000
1	-1.781300000	-3.890742000	-1.764161000
1	-2.158277000	-5.491825000	-1.220060000
1	5.278626000	1.125363000	-3.246062000
1	5.619187000	-0.466711000	-3.891686000
1	-0.416418000	0.284121000	5.075664000
1	-1.855940000	0.983627000	5.851893000
1	-0.637108000	2.035730000	5.123811000
1	-1.662075000	-6.255283000	1.170602000
1	-1.064852000	-5.085495000	2.366122000
1	-2.724991000	-4.978754000	1.770101000
1	6.511052000	-0.968934000	-1.397102000
1	7.519027000	-0.154540000	-2.590108000
1	0.740802000	5.190628000	2.397255000
1	2.432994000	5.609769000	2.080791000
1	-9.411251000	4.279110000	-3.955726000

1	-9.971465000	4.937141000	-2.414221000
1	-7.556935000	4.960875000	-2.361129000
1	-8.073476000	3.723953000	-1.230813000
1	-7.410049000	1.933004000	-2.763368000
1	-7.194096000	3.066946000	-4.094084000
1	-5.308154000	4.158183000	-2.950545000
1	-5.537017000	3.173239000	-1.509378000
1	-4.838210000	1.953873000	-4.234054000
1	-4.854249000	1.138332000	-2.670251000
1	0.285286000	1.541171000	-7.030406000
1	-0.440893000	-1.567960000	-2.390723000
1	-0.183232000	-0.346217000	-3.635002000
1	-2.920721000	-1.499553000	-2.850743000
1	-2.628417000	-0.316593000	-4.137220000
1	5.909499000	-2.378780000	3.801281000
1	4.396390000	-2.610064000	4.699622000
1	5.027955000	-3.794341000	1.927220000
1	3.599353000	-4.195717000	2.891974000
1	-8.551485000	-1.821716000	-1.553730000
1	2.476909000	7.984701000	-0.466706000
1	5.494697000	-3.980039000	4.455132000
6	-0.336750000	1.365593000	-6.149709000
1	-1.275474000	1.911875000	-6.255412000
1	0.180804000	1.723131000	-5.259598000
1	-0.545777000	0.300226000	-6.052710000
1	-5.332819000	-2.487529000	7.860726000
1	5.265713000	-6.963482000	1.061769000
1	7.519508000	1.681926000	0.542459000
1	5.011269000	-5.595581000	-1.287921000
1	3.560738000	4.049549000	4.972534000
1	-5.876260000	1.615440000	4.536009000
1	4.624777000	-2.154252000	0.398255000
1	3.409067000	-0.954001000	0.585901000
1	-5.015988000	-3.337114000	0.212460000
1	-10.085669000	3.209706000	-2.721259000
1	4.180731000	2.058798000	-1.402403000
1	5.365215000	2.520689000	1.045807000
6	6.631057000	4.218131000	0.843214000
8	6.834592000	5.289913000	0.320348000
8	7.669056000	3.415159000	1.252861000
1	8.496894000	3.873511000	1.009450000
8	-4.098452000	-3.643400000	0.352702000
8	-5.178575000	1.671511000	3.854308000

TS_3
A-TS_3-opt.gjf.log

Temperature 298.150 Kelvin. Pressure 1.00000 Atm.

Zero-point correction= 1.434637 (Hartree/Particle)
Thermal correction to Energy= 1.534942
Thermal correction to Enthalpy= 1.535887
Thermal correction to Gibbs Free Energy= 1.283305
Sum of electronic and zero-point Energies= -5896.894708
Sum of electronic and thermal Energies= -5896.794403
Sum of electronic and thermal Enthalpies= -5896.793459
Sum of electronic and thermal Free Energies= -5897.046040

6	-3.727864000	-7.079834000	-0.495085000
6	-3.329599000	-5.777144000	-1.136349000
6	-4.060208000	-5.183466000	-2.175105000
6	-2.124039000	-5.171514000	-0.759352000
6	-3.592600000	-4.040483000	-2.827707000
6	-1.642537000	-4.035680000	-1.399311000
6	-2.371652000	-3.468361000	-2.450145000
8	-1.938831000	-2.362712000	-3.124268000
6	3.517279000	2.089577000	3.918938000
6	-0.369677000	5.316224000	-0.394479000
6	-6.722163000	-0.834216000	-0.335779000
6	-1.998707000	-3.093959000	5.137190000
7	1.117906000	0.301485000	1.846040000
6	4.682420000	1.865388000	4.884032000
6	-1.780011000	5.508060000	-0.950717000
7	0.456152000	2.131552000	-0.366742000
8	-6.575478000	-2.043727000	-0.407340000
8	-1.149169000	-2.768597000	5.943296000
7	-1.281887000	-1.250119000	1.198497000
8	6.194788000	2.926056000	6.366467000
8	-2.235974000	7.320106000	0.622137000
8	4.808176000	4.275901000	5.213655000
8	-3.786011000	6.752698000	-0.913746000
6	0.254447000	-1.607704000	3.092323000
6	2.383989000	2.251423000	1.120004000
6	-1.259282000	2.657116000	-2.030693000
6	-3.273110000	-1.377212000	-0.248564000
28	-0.320115000	0.324734000	0.290010000
6	1.135194000	-0.580476000	2.904110000
6	1.584159000	2.722514000	0.083587000
6	-2.111047000	1.534381000	-1.890288000
6	-2.493474000	-1.805160000	0.821335000
6	2.251651000	-0.257873000	3.910510000
6	1.889655000	4.035928000	-0.665425000

6	-3.395592000	1.401262000	-2.530049000
6	-2.858986000	-2.869947000	1.722300000
6	3.063832000	0.838692000	3.139036000
6	0.477775000	4.342621000	-1.256756000
6	-3.965968000	0.239978000	-2.046784000
6	-1.844553000	-2.948635000	2.652204000
6	2.138865000	1.172505000	1.964873000
6	-0.156498000	2.961363000	-1.276912000
6	-3.032879000	-0.293386000	-1.086383000
6	-0.886723000	-1.920849000	2.310871000
6	3.080765000	-1.554031000	4.178109000
6	-4.038708000	2.464585000	-3.374615000
6	-4.164134000	-3.607743000	1.697232000
6	4.298730000	-1.340687000	5.035468000
6	4.251012000	3.363654000	-1.594102000
6	-4.445121000	3.649305000	-2.505672000
6	-5.253126000	-2.779232000	2.402175000
8	4.308708000	-1.222813000	6.247442000
8	4.801323000	3.225556000	-0.518116000
8	-4.986226000	3.560114000	-1.412773000
6	2.828524000	3.791348000	-1.875728000
6	-5.313420000	-0.317604000	-2.409811000
8	-4.127738000	4.836281000	-3.046986000
6	1.594861000	0.244715000	5.213418000
6	2.458755000	5.147451000	0.230649000
6	-6.457405000	0.133155000	-1.469613000
6	-1.756021000	-3.859149000	3.849863000
6	5.198882000	3.156019000	5.474519000
6	-2.563922000	6.618882000	-0.309197000
7	-1.903678000	0.511487000	-1.019708000
8	-7.192928000	-0.225648000	0.767768000
8	-3.304674000	-2.766325000	5.292869000
6	7.670452000	-6.253124000	-2.902799000
6	6.337585000	-5.806185000	-2.279429000
6	5.606739000	-4.660909000	-3.001742000
6	4.161188000	-4.494466000	-2.487685000
6	3.490523000	-3.194328000	-2.953172000
16	1.655366000	-3.234224000	-2.673064000
6	0.939143000	-0.172127000	-3.079725000
8	1.614834000	0.379451000	-6.071838000
16	0.918370000	-1.324455000	-1.636501000
6	2.338304000	0.208473000	-3.532925000
8	3.775587000	1.414132000	-5.370814000
16	2.332775000	1.207859000	-5.054493000
8	1.605534000	2.468760000	-4.697062000
6	7.381209000	-0.882961000	-2.486020000

6	6.257124000	-1.109786000	-1.504637000
6	5.194584000	-0.202153000	-1.418507000
6	6.241175000	-2.219782000	-0.649009000
6	4.119652000	-0.417734000	-0.559148000
6	5.165517000	-2.462777000	0.203881000
6	4.080146000	-1.581882000	0.216391000
8	3.001905000	-1.914529000	0.984485000
6	-4.790900000	4.481889000	3.357917000
6	-3.934267000	4.526747000	2.076312000
6	-3.095574000	3.256099000	1.916571000
7	-3.385316000	2.464493000	0.860377000
8	-2.212161000	2.983036000	2.737450000
1	-3.513134000	-7.922267000	-1.169309000
1	-3.136762000	-7.253490000	0.411949000
1	-5.006476000	-5.622012000	-2.485300000
1	-1.545065000	-5.595177000	0.057946000
1	-4.162819000	-3.579418000	-3.629235000
1	-0.721872000	-3.572545000	-1.067194000
1	-1.215632000	-1.940003000	-2.613898000
1	7.015933000	-0.425249000	-3.411853000
1	7.888314000	-1.819086000	-2.744458000
1	5.182723000	0.667720000	-2.068352000
1	7.064104000	-2.930585000	-0.677295000
1	3.293190000	0.281744000	-0.516413000
1	5.131156000	-3.351653000	0.826723000
1	2.258595000	-1.311179000	0.790563000
1	3.837995000	2.850216000	3.203080000
1	2.671285000	2.531253000	4.454384000
1	0.131197000	6.288224000	-0.346677000
1	-0.438778000	4.940497000	0.633308000
1	4.425076000	1.205041000	5.717599000
1	5.521835000	1.379000000	4.371296000
1	-1.764626000	5.697032000	-2.030959000
1	-2.370029000	4.595265000	-0.820151000
1	0.396021000	-2.213105000	3.980521000
1	3.289497000	2.810726000	1.299360000
1	-1.567500000	3.404948000	-2.753558000
1	3.961354000	0.369845000	2.708892000
1	0.552835000	4.761083000	-2.267465000
1	3.373429000	-1.982947000	3.219804000
1	2.447448000	-2.270721000	4.708842000
1	-3.389998000	2.820135000	-4.180569000
1	-4.953998000	2.082994000	-3.843537000
1	-4.076585000	-4.582972000	2.188660000
1	-4.472966000	-3.812774000	0.667989000
1	-5.023850000	-2.693160000	3.472317000

1	2.387179000	3.077767000	-2.583069000
1	2.907061000	4.723766000	-2.452541000
1	-5.285400000	-1.412337000	-2.429060000
1	-5.561065000	-0.000578000	-3.428736000
1	0.968923000	1.121168000	5.016496000
1	2.334367000	0.504305000	5.975796000
1	0.949410000	-0.537950000	5.625240000
1	2.502840000	6.093128000	-0.323057000
1	1.846923000	5.298985000	1.123884000
1	3.472082000	4.903562000	0.552977000
1	-6.273732000	1.136741000	-1.076125000
1	-7.401980000	0.179486000	-2.030433000
1	-0.768612000	-4.322952000	3.933306000
1	-2.504038000	-4.654913000	3.770977000
1	7.540559000	-6.474068000	-3.970294000
1	8.022154000	-7.174814000	-2.421750000
1	5.668194000	-6.678688000	-2.255989000
1	6.492904000	-5.520824000	-1.229234000
1	6.160069000	-3.723381000	-2.870628000
1	5.582786000	-4.859303000	-4.083160000
1	3.573385000	-5.361103000	-2.821619000
1	4.150843000	-4.513173000	-1.391323000
1	3.657179000	-3.032549000	-4.024332000
1	3.906930000	-2.341963000	-2.415434000
1	-1.698485000	1.099997000	-4.773223000
1	0.379029000	0.706971000	-2.758675000
1	0.397897000	-0.648388000	-3.899708000
1	2.858678000	0.795260000	-2.775459000
1	2.940868000	-0.672466000	-3.759053000
1	-5.746379000	3.974926000	3.188529000
1	-4.246556000	3.937567000	4.137426000
1	-4.552411000	4.713467000	1.190819000
1	-3.212303000	5.347326000	2.151041000
1	8.131468000	-0.206233000	-2.055958000
1	-4.779644000	-7.194886000	-0.202807000
1	-4.980699000	5.486796000	3.750217000
6	-1.738084000	0.294688000	-5.505262000
1	-2.522153000	0.503591000	-6.236391000
1	-1.961840000	-0.634802000	-4.982179000
1	-0.766933000	0.223128000	-5.992744000
1	6.480134000	3.798289000	6.699666000
1	-4.278104000	7.453161000	-0.444842000
1	-7.400669000	-0.928132000	1.428561000
1	-4.310452000	5.533514000	-2.376097000
1	-3.368903000	-2.223959000	6.102234000
1	-4.027194000	2.750329000	0.128163000

1	-2.757153000	1.694721000	0.643082000
1	8.489886000	-5.528167000	-2.818632000
1	-4.187969000	-1.928309000	-0.427955000
1	-5.267364000	-1.758665000	2.010662000
6	-6.627726000	-3.373096000	2.268797000
8	-6.906889000	-4.521661000	2.010492000
8	-7.606277000	-2.440825000	2.521314000
1	-8.465193000	-2.893813000	2.413791000
1	5.826151000	2.902349000	-2.526148000
1	6.168491000	-1.100409000	4.925153000
8	5.435841000	-1.263625000	4.299650000
8	4.912211000	3.155508000	-2.757901000

PC
A-PC-opt.gjf.log

Temperature 298.150 Kelvin. Pressure 1.00000 Atm.

Zero-point correction= 1.431669 (Hartree/Particle)
Thermal correction to Energy= 1.533536
Thermal correction to Enthalpy= 1.534480
Thermal correction to Gibbs Free Energy= 1.273483
Sum of electronic and zero-point Energies= -5896.906056
Sum of electronic and thermal Energies= -5896.804189
Sum of electronic and thermal Enthalpies= -5896.803245
Sum of electronic and thermal Free Energies= -5897.064242

6	2.555644000	1.578238000	6.951659000
6	1.975909000	1.852531000	5.584600000
6	1.104692000	2.929000000	5.354757000
6	2.246092000	0.993645000	4.507499000
6	0.535680000	3.149188000	4.098390000
6	1.684004000	1.193900000	3.250424000
6	0.825012000	2.282644000	3.039499000
8	0.233871000	2.525595000	1.834556000
6	4.681206000	-3.965229000	0.084175000
6	0.147538000	-2.990491000	-4.087925000
6	-2.361287000	6.846654000	-2.273501000
6	4.882314000	3.093237000	3.456390000
7	2.847020000	-1.043576000	0.369830000
6	5.575247000	-5.021979000	0.734343000
6	-0.067575000	-2.047210000	-5.286786000
7	0.516641000	-1.369090000	-1.362906000
8	-1.864648000	7.563196000	-3.121823000
8	5.321884000	2.167175000	4.113134000
7	2.660433000	1.855260000	-0.015014000

8	7.047945000	-6.829825000	0.304176000
8	0.877226000	-2.635774000	-7.456426000
8	5.887847000	-6.047337000	-1.456742000
8	2.229194000	-1.847381000	-5.836484000
6	4.382150000	0.628258000	1.254873000
6	1.716832000	-3.131630000	-0.188068000
6	-1.042981000	-0.163175000	-2.820828000
6	1.357699000	3.672529000	-1.041051000
28	1.559257000	0.248675000	-0.633078000
6	3.971199000	-0.661653000	1.064086000
6	0.710998000	-2.663405000	-1.021553000
6	-0.671705000	1.175857000	-2.527243000
6	2.410164000	3.201244000	-0.259174000
6	4.681737000	-1.859233000	1.718813000
6	-0.356155000	-3.580265000	-1.644594000
6	-1.239635000	2.348162000	-3.157681000
6	3.368469000	4.019398000	0.438889000
6	3.916484000	-3.069208000	1.085770000
6	-0.759239000	-2.696935000	-2.863534000
6	-0.584524000	3.440315000	-2.626415000
6	4.224947000	3.146318000	1.079706000
6	2.743615000	-2.382461000	0.391333000
6	-0.468195000	-1.305885000	-2.325278000
6	0.402090000	2.914169000	-1.713386000
6	3.768197000	1.812442000	0.776402000
6	4.377857000	-1.768906000	3.232184000
6	-2.275059000	2.361813000	-4.253229000
6	3.410588000	5.519510000	0.426480000
6	4.901935000	-2.894317000	4.094947000
6	-2.853712000	-4.037375000	-1.220356000
6	-3.686683000	2.189574000	-3.713536000
6	4.054114000	6.067338000	-0.857327000
8	5.434305000	-3.923118000	3.726118000
8	-3.145451000	-5.118562000	-1.702689000
8	-4.297374000	3.072317000	-3.128311000
6	-1.519842000	-3.662818000	-0.604856000
6	-0.870065000	4.888041000	-2.897183000
8	-4.165364000	0.963721000	-3.924468000
6	6.199427000	-1.806761000	1.472659000
6	0.113704000	-4.999976000	-1.985021000
6	-2.039077000	5.393449000	-2.035300000
6	5.329327000	3.471309000	2.052412000
6	6.156939000	-5.984686000	-0.272168000
6	1.010418000	-2.221797000	-6.324249000
7	0.322074000	1.528928000	-1.667120000
8	-3.311448000	7.295066000	-1.417076000

8	3.880950000	3.888625000	3.879378000
6	-8.166689000	-2.339895000	5.776072000
6	-7.398347000	-1.048022000	5.471096000
6	-7.601171000	-0.554315000	4.033394000
6	-6.662426000	0.604836000	3.666830000
6	-7.046890000	1.254494000	2.339940000
16	-5.898676000	2.584946000	1.779595000
6	-4.108314000	1.258755000	-0.460281000
8	-6.999361000	-0.404167000	-0.304452000
16	-4.101331000	1.601212000	1.358115000
6	-4.378004000	-0.191445000	-0.870092000
8	-6.007049000	-1.963221000	-1.979945000
16	-6.063350000	-0.563615000	-1.449571000
8	-6.337139000	0.447123000	-2.538571000
6	-7.491173000	-3.607969000	0.792517000
6	-6.057977000	-3.706783000	1.255704000
6	-5.412087000	-2.549584000	1.718958000
6	-5.337218000	-4.905860000	1.283151000
6	-4.111371000	-2.584652000	2.214149000
6	-4.023199000	-4.956533000	1.757491000
6	-3.408023000	-3.794481000	2.229281000
8	-2.114761000	-3.896003000	2.685987000
6	6.340891000	-3.237376000	-3.637364000
6	5.162634000	-2.342242000	-4.021865000
6	4.650680000	-1.494571000	-2.855778000
7	3.431695000	-0.934467000	-3.046943000
8	5.329240000	-1.301287000	-1.843067000
1	1.968924000	2.079353000	7.730567000
1	2.533163000	0.503507000	7.170418000
1	0.862368000	3.605266000	6.171851000
1	2.909467000	0.149674000	4.664113000
1	-0.131910000	3.987699000	3.924304000
1	1.908061000	0.524144000	2.427501000
1	0.717068000	2.048521000	1.127886000
1	-7.614815000	-2.727143000	0.154902000
1	-8.162294000	-3.475409000	1.653839000
1	-5.927457000	-1.597354000	1.655677000
1	-5.801762000	-5.818688000	0.917092000
1	-3.640415000	-1.669922000	2.563933000
1	-3.462664000	-5.886612000	1.756252000
1	-1.781521000	-3.008614000	2.896617000
1	3.945175000	-4.491474000	-0.532309000
1	5.252937000	-3.343729000	-0.608437000
1	-0.017579000	-4.020215000	-4.422493000
1	1.192287000	-2.920794000	-3.764659000
1	6.398041000	-4.588039000	1.306726000

1	5.010460000	-5.614892000	1.466241000
1	-1.034328000	-2.226023000	-5.764387000
1	-0.035654000	-1.007141000	-4.945227000
1	5.254463000	0.773529000	1.881831000
1	1.752950000	-4.202553000	-0.017909000
1	-1.843975000	-0.294994000	-3.542165000
1	3.531290000	-3.724540000	1.877038000
1	-1.807041000	-2.818494000	-3.155110000
1	3.290971000	-1.735942000	3.388851000
1	4.764487000	-0.827895000	3.640626000
1	-2.245016000	3.325040000	-4.772629000
1	-2.073407000	1.573904000	-4.985943000
1	3.965379000	5.895594000	1.292326000
1	2.399648000	5.933764000	0.518993000
1	5.076579000	5.684638000	-0.971364000
1	-1.262846000	-4.393602000	0.166685000
1	-1.638941000	-2.697442000	-0.116973000
1	-1.125964000	5.041433000	-3.952722000
1	0.013833000	5.507486000	-2.711001000
1	6.415071000	-1.789063000	0.402366000
1	6.707459000	-2.653888000	1.937854000
1	6.618443000	-0.894923000	1.911917000
1	-0.682943000	-5.541948000	-2.504051000
1	1.015009000	-5.003948000	-2.603705000
1	0.339998000	-5.556566000	-1.069315000
1	-2.939749000	4.802205000	-2.246953000
1	-1.828003000	5.253063000	-0.968013000
1	5.555942000	4.542299000	2.029500000
1	6.250881000	2.922389000	1.837542000
1	-9.197665000	-2.289250000	5.404352000
1	-8.210199000	-2.532757000	6.855138000
1	-7.710431000	-0.253504000	6.163609000
1	-6.326749000	-1.209318000	5.655072000
1	-7.444245000	-1.384221000	3.332006000
1	-8.648467000	-0.241351000	3.908972000
1	-6.671491000	1.361730000	4.463318000
1	-5.633684000	0.225949000	3.602906000
1	-8.010283000	1.775308000	2.426795000
1	-7.127449000	0.529420000	1.528433000
1	-3.091787000	1.520278000	-0.774422000
1	-4.778823000	1.966168000	-0.952608000
1	-3.732711000	-0.456344000	-1.710581000
1	-4.164848000	-0.899027000	-0.069735000
1	7.103975000	-2.654218000	-3.114065000
1	6.031893000	-4.056521000	-2.981410000
1	5.461637000	-1.644291000	-4.817389000

1	4.335572000	-2.933172000	-4.434673000
1	-7.862917000	-4.478798000	0.236379000
1	3.596940000	1.895029000	7.094004000
1	6.782172000	-3.654695000	-4.548825000
1	7.366073000	-7.425029000	-0.401482000
1	2.887012000	-1.990240000	-6.543054000
1	-3.476420000	8.230202000	-1.643939000
1	-5.050359000	0.841612000	-3.447973000
1	3.472390000	3.474362000	4.667274000
1	5.024905000	-3.392556000	5.911182000
1	2.863119000	-1.188316000	-3.845091000
1	2.962327000	-0.488405000	-2.256634000
1	-4.558047000	-3.083181000	-1.579657000
1	-7.676727000	-3.206733000	5.315083000
1	1.280278000	4.750804000	-1.145814000
1	3.510558000	5.723146000	-1.745348000
6	4.115048000	7.576065000	-0.893093000
8	3.770279000	8.327824000	-0.003168000
8	4.620852000	8.026458000	-2.067413000
1	4.629880000	9.001508000	-2.015384000
8	-3.654798000	-2.971244000	-1.198650000
8	4.687987000	-2.627167000	5.406742000
6	-0.917759000	-0.816749000	1.796679000
1	-1.843631000	-0.950957000	1.240866000
1	-0.459158000	0.118106000	1.491188000
1	-1.130049000	-0.773898000	2.866831000
1	-0.229278000	-1.629829000	1.570193000

B

RC
B-RC-opt.gjf.log

Temperature 298.150 Kelvin. Pressure 1.00000 Atm.

Zero-point correction= 1.457577 (Hartree/Particle)
Thermal correction to Energy= 1.558640
Thermal correction to Enthalpy= 1.559584
Thermal correction to Gibbs Free Energy= 1.299594
Sum of electronic and zero-point Energies= -5857.145219
Sum of electronic and thermal Energies= -5857.044155
Sum of electronic and thermal Enthalpies= -5857.043211
Sum of electronic and thermal Free Energies= -5857.303202

6	1.455498000	-3.266028000	-6.516274000
6	1.522389000	-3.086569000	-5.017065000

6	1.768595000	-4.173665000	-4.163726000
6	1.402007000	-1.815177000	-4.436822000
6	1.890233000	-4.003235000	-2.785111000
6	1.533222000	-1.624541000	-3.062353000
6	1.772449000	-2.723152000	-2.228511000
8	1.881572000	-2.614223000	-0.876505000
6	0.164696000	5.218055000	-1.189020000
6	-0.613933000	2.273358000	5.069544000
6	4.313059000	-5.970563000	1.184218000
6	4.342045000	-0.066537000	-4.622167000
7	0.790163000	2.049067000	-0.688934000
6	-0.289010000	6.414374000	-2.026916000
6	0.062348000	1.456269000	6.186175000
7	-0.094387000	1.181928000	1.968241000
8	5.307370000	-6.311912000	1.796264000
8	3.530993000	0.456132000	-5.363501000
7	3.120004000	0.338373000	-0.481112000
8	-0.257937000	8.780432000	-2.171092000
8	0.357450000	2.404475000	8.407057000
8	1.255564000	7.842027000	-0.796626000
8	1.669579000	3.115026000	6.720741000
6	2.519108000	1.766575000	-2.394587000
6	-1.112216000	2.904603000	0.592907000
6	0.296056000	-0.537561000	3.654929000
6	4.170651000	-1.426037000	0.866975000
28	1.480650000	0.721787000	0.707025000
6	1.331807000	2.273891000	-1.939262000
6	-1.022172000	2.136864000	1.750630000
6	1.400793000	-1.205634000	3.066075000
6	4.164868000	-0.535223000	-0.206598000
6	0.393862000	3.142076000	-2.789338000
6	-2.043958000	2.244343000	2.898635000
6	1.972653000	-2.454540000	3.527527000
6	5.117582000	-0.525252000	-1.284579000
6	-0.408989000	3.859611000	-1.660957000
6	-1.327387000	1.403186000	4.005238000
6	3.091383000	-2.686237000	2.755920000
6	4.633699000	0.372412000	-2.218734000
6	-0.269874000	2.860791000	-0.516870000
6	-0.318452000	0.601446000	3.196259000
6	3.183340000	-1.578281000	1.834617000
6	3.387995000	0.880185000	-1.704980000
6	-0.545677000	2.148169000	-3.555503000
6	1.354306000	-3.388907000	4.535562000
6	6.366878000	-1.353637000	-1.352289000
7	-4.668846000	-0.048761000	3.758975000

6	-1.640847000	2.827501000	-4.372784000
6	-4.359325000	1.256463000	3.543576000
6	0.289180000	-4.223786000	3.833490000
6	7.469230000	-0.820390000	-0.424258000
8	-1.400317000	3.420934000	-5.428448000
8	-4.852441000	2.163646000	4.221431000
8	0.537037000	-5.213776000	3.164068000
6	-3.348406000	1.526600000	2.434194000
6	3.926511000	-3.929565000	2.664949000
7	-2.892807000	2.751296000	-3.853990000
8	-0.938562000	-3.718877000	3.984030000
6	1.131829000	4.039893000	-3.790010000
6	-2.356850000	3.704063000	3.268672000
6	3.415433000	-4.807717000	1.507122000
6	5.186280000	0.661608000	-3.591166000
6	0.331470000	7.715597000	-1.576532000
6	0.679246000	2.346043000	7.235849000
7	2.116252000	-0.712004000	2.018291000
8	3.881470000	-6.637509000	0.082895000
8	4.546958000	-1.399890000	-4.576667000
6	-9.538985000	-3.329751000	-2.576898000
6	-9.099964000	-2.505989000	-3.792749000
6	-8.542737000	-1.115451000	-3.445782000
6	-7.285839000	-1.074198000	-2.561764000
6	-6.023258000	-1.692516000	-3.187979000
16	-5.732526000	-3.485780000	-2.833526000
6	-0.947367000	-2.031233000	0.499552000
8	-3.852193000	-3.434595000	0.144704000
16	-1.154112000	-1.117281000	-1.081917000
6	-2.096371000	-1.914687000	1.498096000
8	-4.362745000	-2.720923000	2.488388000
16	-3.333338000	-3.235343000	1.531621000
8	-2.613519000	-4.456921000	2.040791000
6	-8.125428000	-0.037871000	1.455877000
6	-6.869940000	0.355341000	0.719783000
6	-5.899614000	-0.629163000	0.487627000
6	-6.647187000	1.632013000	0.190966000
6	-4.756195000	-0.360996000	-0.253428000
6	-5.485631000	1.927324000	-0.533892000
6	-4.533022000	0.927689000	-0.745600000
8	-3.377032000	1.238490000	-1.417417000
6	4.958006000	6.192165000	0.406287000
6	3.560301000	5.882171000	0.999129000
6	3.179837000	4.413910000	1.164627000
7	3.606218000	3.553577000	0.202720000
8	2.527405000	4.037185000	2.141386000

1	1.023154000	-4.234680000	-6.791158000
1	0.860703000	-2.478343000	-6.990800000
1	1.868619000	-5.171740000	-4.585351000
1	1.234052000	-0.948242000	-5.070370000
1	2.086250000	-4.845709000	-2.127356000
1	1.444549000	-0.633793000	-2.636017000
1	1.875460000	-1.672028000	-0.611381000
1	-7.884496000	-0.715142000	2.284399000
1	-8.801734000	-0.592407000	0.789066000
1	-6.025118000	-1.628404000	0.892487000
1	-7.382845000	2.416610000	0.353838000
1	-4.046397000	-1.154939000	-0.444723000
1	-5.299576000	2.928151000	-0.913188000
1	-2.703889000	0.535144000	-1.247896000
1	-0.153804000	5.385492000	-0.154226000
1	1.257945000	5.164837000	-1.158059000
1	-1.350826000	2.941219000	5.529202000
1	0.138864000	2.903661000	4.583720000
1	-0.037544000	6.295005000	-3.086212000
1	-1.379870000	6.524281000	-1.994536000
1	-0.659435000	0.803747000	6.685822000
1	0.853825000	0.832945000	5.757475000
1	2.811759000	2.024279000	-3.405510000
1	-1.923832000	3.623154000	0.540329000
1	-0.095817000	-0.964797000	4.572429000
1	-1.461272000	3.997270000	-1.934826000
1	-2.032025000	0.743394000	4.524921000
1	-0.973645000	1.445401000	-2.833328000
1	0.070610000	1.571247000	-4.252884000
1	2.107888000	-4.074308000	4.934158000
1	0.903968000	-2.842123000	5.369837000
1	6.747918000	-1.392283000	-2.377634000
1	6.150299000	-2.393788000	-1.078582000
1	7.727884000	0.214469000	-0.683407000
1	-3.846446000	2.145647000	1.682154000
1	-3.080421000	0.595248000	1.940353000
1	3.888194000	-4.502416000	3.598078000
1	4.982644000	-3.686478000	2.496705000
1	1.856609000	4.688245000	-3.288264000
1	0.432270000	4.648078000	-4.366168000
1	1.678281000	3.428184000	-4.515287000
1	-3.027855000	3.745874000	4.128800000
1	-1.446252000	4.271362000	3.481527000
1	-2.869554000	4.200005000	2.437646000
1	2.420087000	-5.195586000	1.756065000
1	3.281230000	-4.208271000	0.598976000

1	6.222652000	0.317890000	-3.667612000
1	5.158182000	1.728096000	-3.831558000
1	-8.669594000	-3.650995000	-1.993545000
1	-10.084397000	-4.229911000	-2.884396000
1	-8.350542000	-3.075328000	-4.359545000
1	-9.955066000	-2.369711000	-4.469721000
1	-8.327377000	-0.578435000	-4.381557000
1	-9.332990000	-0.541158000	-2.939864000
1	-7.059307000	-0.023757000	-2.337201000
1	-7.482138000	-1.540765000	-1.591588000
1	-5.130874000	-1.157760000	-2.855931000
1	-6.055354000	-1.619441000	-4.280800000
1	-5.214238000	-3.339148000	-1.592609000
1	-0.064055000	-1.576077000	0.949784000
1	-0.694418000	-3.069022000	0.266147000
1	-1.700327000	-1.881551000	2.515309000
1	-2.657055000	-0.999641000	1.328624000
1	5.651880000	5.357518000	0.551800000
1	4.924356000	6.430282000	-0.661344000
1	3.474303000	6.305189000	2.004140000
1	2.777098000	6.364155000	0.399663000
1	-8.713529000	0.786910000	1.879783000
1	2.462376000	-3.224136000	-6.951474000
1	5.357170000	7.049500000	0.958789000
6	-1.824514000	-2.406078000	-2.185927000
1	-2.064446000	-1.921662000	-3.132582000
1	-1.061889000	-3.165212000	-2.360484000
1	-2.716342000	-2.851940000	-1.755897000
1	0.208309000	9.576790000	-1.851438000
1	2.007439000	3.669308000	7.449691000
1	4.504200000	-7.375217000	-0.061976000
1	-1.566922000	-4.154385000	3.330022000
1	3.823217000	-1.827026000	-5.076990000
1	-3.646590000	3.181615000	-4.372919000
1	-3.111730000	2.232331000	-3.005593000
1	3.929699000	3.893323000	-0.691795000
1	3.220153000	2.612544000	0.210834000
1	-5.390070000	-0.237813000	4.443158000
1	-10.187430000	-2.753321000	-1.905151000
1	4.987524000	-2.141662000	0.894178000
1	7.121771000	-0.785078000	0.615182000
6	8.734566000	-1.643676000	-0.474250000
8	8.938261000	-2.600229000	-1.195025000
8	9.662714000	-1.184622000	0.400763000
1	10.445121000	-1.761899000	0.310632000
1	-4.408553000	-0.824244000	3.150278000

TS_1

B-TS_1-opt.gjf.log

Temperature 298.150 Kelvin. Pressure 1.00000 Atm.

Zero-point correction= 1.452407 (Hartree/Particle)
Thermal correction to Energy= 1.555103
Thermal correction to Enthalpy= 1.556047
Thermal correction to Gibbs Free Energy= 1.289874
Sum of electronic and zero-point Energies= -5857.073041
Sum of electronic and thermal Energies= -5856.970345
Sum of electronic and thermal Enthalpies= -5856.969401
Sum of electronic and thermal Free Energies= -5857.235574

6	-0.255085000	7.459564000	1.468618000
6	-0.116963000	6.280333000	0.539978000
6	0.646975000	6.354432000	-0.634557000
6	-0.800444000	5.086140000	0.795508000
6	0.727525000	5.279094000	-1.515851000
6	-0.732976000	3.999474000	-0.073055000
6	0.035982000	4.089239000	-1.241817000
8	0.138878000	3.070091000	-2.131618000
6	-0.103382000	-2.825363000	4.466301000
6	2.452944000	-5.049621000	-0.958607000
6	6.948528000	3.180313000	-3.316399000
6	1.888387000	3.719200000	4.867132000
7	-0.162002000	-0.739352000	1.922809000
6	-0.614081000	-3.318029000	5.822748000
6	3.687953000	-5.013143000	-1.883247000
7	0.765807000	-2.291066000	-0.451557000
8	6.712301000	3.940528000	-4.234709000
8	1.316089000	3.151178000	5.776203000
7	1.419340000	1.559571000	1.014622000
8	-0.030552000	-4.571916000	7.748258000
8	5.390210000	-6.602566000	-1.163297000
8	1.226225000	-4.915156000	5.913407000
8	5.440858000	-4.485822000	-0.389504000
6	0.073159000	1.399950000	3.074735000
6	-0.651922000	-3.071943000	1.364415000
6	2.195081000	-2.150327000	-2.427249000
6	2.985381000	2.338792000	-0.711588000
28	0.869729000	-0.259194000	0.159883000
6	-0.404039000	0.121181000	2.971403000
6	-0.006526000	-3.221615000	0.141515000
6	2.611514000	-0.795542000	-2.344827000

6	2.296722000	2.489646000	0.488920000
6	-1.350684000	-0.531561000	3.988769000
6	-0.073382000	-4.507573000	-0.694537000
6	3.470320000	-0.132828000	-3.293458000
6	2.381224000	3.633424000	1.363659000
6	-1.148897000	-2.039991000	3.637987000
6	1.213997000	-4.345299000	-1.563676000
6	3.712611000	1.136331000	-2.800307000
6	1.541653000	3.366613000	2.423945000
6	-0.651419000	-1.953318000	2.202166000
6	1.409355000	-2.835125000	-1.535653000
6	2.988888000	1.232837000	-1.560505000
6	0.962665000	2.065340000	2.186036000
6	-2.799825000	-0.079292000	3.592353000
6	4.039550000	-0.752204000	-4.543245000
6	3.258094000	4.831365000	1.161011000
7	-3.357731000	-5.288953000	-0.679820000
6	-3.875267000	-0.998108000	4.155965000
6	-2.631469000	-4.189096000	-1.046350000
6	5.277292000	-1.562760000	-4.205487000
6	4.679007000	4.589273000	1.692594000
8	-4.149644000	-1.060964000	5.356849000
8	-3.044837000	-3.052077000	-0.826012000
8	6.366269000	-1.103965000	-3.917875000
6	-1.283132000	-4.455126000	-1.689313000
6	4.608399000	2.193519000	-3.375800000
7	-4.472895000	-1.788343000	3.224712000
8	5.041504000	-2.896700000	-4.217161000
6	-1.073020000	-0.133064000	5.442683000
6	-0.148948000	-5.787422000	0.148580000
6	6.021729000	2.120678000	-2.775209000
6	1.242302000	4.251085000	3.604330000
6	0.304662000	-4.337758000	6.455822000
6	4.911753000	-5.487981000	-1.147446000
7	2.316651000	0.045327000	-1.315767000
8	8.132323000	3.194418000	-2.653749000
8	3.229950000	3.919226000	4.870424000
6	-10.638104000	3.747016000	-2.380036000
6	-10.246913000	4.060042000	-0.927616000
6	-9.853057000	2.824078000	-0.101946000
6	-8.758831000	1.907026000	-0.671134000
6	-7.306060000	2.390374000	-0.590571000
16	-6.858636000	3.646278000	-1.884761000
6	-0.837764000	-0.213093000	-2.789737000
8	-0.743791000	-0.871964000	-5.807242000
16	-1.246456000	0.456758000	-1.112594000

6	-1.945275000	-1.021800000	-3.447254000
8	-2.593638000	-2.531882000	-5.499711000
16	-1.360172000	-1.910631000	-4.927452000
8	-0.368428000	-2.918938000	-4.429364000
6	-8.776272000	-1.312561000	-2.558267000
6	-7.498641000	-1.178302000	-1.766945000
6	-6.275339000	-0.918776000	-2.396186000
6	-7.500365000	-1.278536000	-0.368110000
6	-5.098785000	-0.758896000	-1.665906000
6	-6.337719000	-1.098610000	0.380022000
6	-5.124535000	-0.840627000	-0.271442000
8	-4.004012000	-0.666958000	0.492183000
6	3.931679000	-3.740760000	4.052062000
6	4.180555000	-3.524511000	2.559768000
6	3.660795000	-2.184442000	2.037159000
7	3.949936000	-1.927201000	0.731764000
8	3.051717000	-1.396683000	2.761731000
1	-0.709774000	8.318396000	0.954858000
1	-0.908610000	7.212511000	2.313405000
1	1.191866000	7.269126000	-0.861494000
1	-1.396385000	4.995983000	1.701645000
1	1.328859000	5.335886000	-2.418986000
1	-1.238878000	3.070727000	0.163712000
1	-0.259059000	2.249466000	-1.732753000
1	-8.578137000	-1.329837000	-3.635082000
1	-9.459316000	-0.476333000	-2.362050000
1	-6.232912000	-0.843017000	-3.480299000
1	-8.434032000	-1.487031000	0.150834000
1	-4.159573000	-0.574480000	-2.165899000
1	-6.362876000	-1.150210000	1.463706000
1	-3.196230000	-0.634157000	-0.073642000
1	0.187272000	-3.705003000	3.882736000
1	0.811067000	-2.232632000	4.582898000
1	2.224269000	-6.098370000	-0.745424000
1	2.692606000	-4.570244000	-0.002453000
1	-0.767440000	-2.510450000	6.542080000
1	-1.595661000	-3.800697000	5.709619000
1	3.529831000	-5.660742000	-2.749944000
1	3.869820000	-3.995920000	-2.233784000
1	-0.200080000	1.953183000	3.966085000
1	-1.187792000	-3.935565000	1.743558000
1	2.540569000	-2.706749000	-3.289517000
1	-2.088276000	-2.599528000	3.682183000
1	1.052970000	-4.709397000	-2.583861000
1	-2.884055000	-0.029555000	2.504550000
1	-2.965873000	0.928947000	3.985266000

1	3.303901000	-1.386867000	-5.044850000
1	4.350945000	0.029734000	-5.242758000
1	2.831496000	5.705800000	1.663842000
1	3.302454000	5.098154000	0.100320000
1	4.645707000	4.337655000	2.760350000
1	-1.104696000	-3.669849000	-2.427609000
1	-1.296098000	-5.406941000	-2.235569000
1	4.190521000	3.191415000	-3.201256000
1	4.683986000	2.084515000	-4.463412000
1	-0.061950000	-0.411260000	5.754233000
1	-1.806983000	-0.594080000	6.108211000
1	-1.164139000	0.951156000	5.565234000
1	-0.036193000	-6.673771000	-0.487048000
1	0.624180000	-5.813490000	0.922015000
1	-1.119855000	-5.864750000	0.646674000
1	5.988641000	2.210794000	-1.682788000
1	6.463549000	1.139114000	-2.988999000
1	0.166244000	4.315766000	3.790146000
1	1.616184000	5.262652000	3.416459000
1	-9.744525000	3.627880000	-3.003717000
1	-11.235060000	4.556542000	-2.815370000
1	-9.429166000	4.792929000	-0.927463000
1	-11.088542000	4.546348000	-0.415595000
1	-9.558449000	3.143007000	0.908311000
1	-10.756042000	2.208803000	0.024238000
1	-8.786272000	0.973653000	-0.097710000
1	-8.990972000	1.628919000	-1.705548000
1	-6.642060000	1.536940000	-0.745257000
1	-7.096685000	2.812845000	0.397622000
1	-5.547268000	3.308923000	-1.981318000
1	0.031049000	-0.852345000	-2.652113000
1	-0.542858000	0.614191000	-3.441183000
1	-2.343229000	-1.774491000	-2.762579000
1	-2.766447000	-0.386298000	-3.790317000
1	4.380765000	-2.939179000	4.646841000
1	2.865303000	-3.780507000	4.285448000
1	5.252584000	-3.589877000	2.330821000
1	3.707622000	-4.326044000	1.977144000
1	-9.319951000	-2.231983000	-2.303959000
1	0.685365000	7.828594000	1.897928000
1	4.383529000	-4.694498000	4.345210000
6	-3.134787000	2.005016000	-1.898234000
1	-3.858836000	1.755498000	-1.135012000
1	-2.641401000	2.962696000	-1.814664000
1	-3.338928000	1.646441000	-2.898366000
1	0.587050000	-5.251470000	8.080430000

1	6.190901000	-4.855850000	0.113799000
1	8.677557000	3.889655000	-3.069345000
1	5.868746000	-3.336020000	-3.939121000
1	3.579029000	3.496494000	5.678267000
1	-5.172345000	-2.455301000	3.521175000
1	-4.249816000	-1.699707000	2.237483000
1	4.342805000	-2.657856000	0.151866000
1	3.458590000	-1.175173000	0.253640000
1	-4.307052000	-5.137018000	-0.364228000
1	-11.204639000	2.812697000	-2.480736000
1	3.590666000	3.184673000	-1.024307000
1	5.141116000	3.727806000	1.195035000
6	5.585693000	5.783140000	1.520782000
8	5.256576000	6.883836000	1.124279000
8	6.862670000	5.496879000	1.878461000
1	7.380384000	6.314416000	1.748198000
1	-3.122650000	-6.208931000	-1.022678000

Int_1
B-Int_1-opt.gjf.log

Temperature 298.150 Kelvin. Pressure 1.00000 Atm.

Zero-point correction= 1.450930 (Hartree/Particle)
Thermal correction to Energy= 1.554422
Thermal correction to Enthalpy= 1.555366
Thermal correction to Gibbs Free Energy= 1.288036
Sum of electronic and zero-point Energies= -5857.079844
Sum of electronic and thermal Energies= -5856.976353
Sum of electronic and thermal Enthalpies= -5856.975408
Sum of electronic and thermal Free Energies= -5857.242738

6	-0.555612000	7.442647000	1.505321000
6	-0.358198000	6.259283000	0.589224000
6	0.387307000	6.361220000	-0.595363000
6	-0.977685000	5.032491000	0.858222000
6	0.499977000	5.289168000	-1.477760000
6	-0.874768000	3.947109000	-0.010199000
6	-0.139368000	4.070215000	-1.198793000
8	-0.024823000	3.067846000	-2.102485000
6	0.246803000	-2.549161000	4.825856000
6	2.626511000	-5.126505000	-1.057220000
6	6.434925000	3.389839000	-3.990753000
6	1.718726000	3.798329000	4.924148000
7	0.017543000	-0.830820000	2.101540000
6	-0.249500000	-3.036401000	6.188465000

6	3.770558000	-5.001718000	-2.085570000
7	0.991614000	-2.382015000	-0.239835000
8	5.968291000	4.221687000	-4.743799000
8	1.163704000	3.201263000	5.824995000
7	1.402169000	1.542656000	1.126462000
8	0.420921000	-3.905708000	8.289031000
8	5.692747000	-6.461154000	-1.730618000
8	1.994648000	-3.849323000	6.680935000
8	5.541680000	-4.516416000	-0.600573000
6	0.007983000	1.371250000	3.154859000
6	-0.239987000	-3.228678000	1.677945000
6	2.204673000	-2.146337000	-2.342601000
6	2.872353000	2.381246000	-0.657387000
28	0.960578000	-0.301853000	0.317350000
6	-0.383350000	0.063198000	3.075081000
6	0.310681000	-3.344895000	0.406361000
6	2.548970000	-0.768749000	-2.279475000
6	2.223585000	2.506242000	0.570446000
6	-1.380483000	-0.633159000	4.010055000
6	0.154645000	-4.594619000	-0.465361000
6	3.208195000	-0.032903000	-3.325223000
6	2.246759000	3.670589000	1.421453000
6	-0.885329000	-2.104002000	3.870230000
6	1.330570000	-4.389486000	-1.475943000
6	3.438987000	1.242746000	-2.840053000
6	1.418636000	3.384093000	2.487470000
6	-0.341116000	-2.072716000	2.451480000
6	1.533062000	-2.881269000	-1.399679000
6	2.889522000	1.276148000	-1.510099000
6	0.906821000	2.051095000	2.279892000
6	-2.779844000	-0.495423000	3.307152000
6	3.548021000	-0.576456000	-4.689344000
6	3.049733000	4.914873000	1.193923000
7	-3.095439000	-5.432910000	-0.077602000
6	-3.755156000	-1.595604000	3.698366000
6	-2.436879000	-4.293259000	-0.439267000
6	4.815437000	-1.406592000	-4.628966000
6	4.465264000	4.793118000	1.778200000
8	-4.223472000	-1.697961000	4.836922000
8	-2.827552000	-3.185599000	-0.067138000
8	5.944390000	-0.965072000	-4.524024000
6	-1.183652000	-4.480004000	-1.282546000
6	4.157313000	2.363293000	-3.532157000
7	-4.035479000	-2.489869000	2.713898000
8	4.568689000	-2.738193000	-4.666783000
6	-1.449620000	-0.069300000	5.428840000

6	0.176795000	-5.913912000	0.316846000
6	5.675729000	2.280404000	-3.306556000
6	1.067248000	4.280503000	3.644327000
6	0.854218000	-3.625665000	7.035430000
6	5.083997000	-5.438947000	-1.493675000
7	2.345195000	0.038262000	-1.202303000
8	7.754296000	3.360212000	-3.675799000
8	3.045299000	4.079929000	4.952144000
6	-10.685630000	3.217334000	-2.486127000
6	-10.432214000	3.533347000	-1.004699000
6	-9.984477000	2.312915000	-0.180349000
6	-8.763089000	1.528261000	-0.686490000
6	-7.388979000	2.183099000	-0.511330000
16	-7.045357000	3.553454000	-1.717936000
6	-0.665149000	-0.542708000	-2.594036000
8	-0.001469000	-1.200195000	-5.508361000
16	-1.127124000	0.345284000	-1.045601000
6	-1.790745000	-0.873591000	-3.557907000
8	-2.322203000	-2.070850000	-5.851139000
16	-1.166838000	-1.929235000	-4.915292000
8	-0.776473000	-3.217700000	-4.255695000
6	-8.542671000	-1.727134000	-2.725394000
6	-7.279089000	-1.459602000	-1.944636000
6	-6.154115000	-0.910517000	-2.572885000
6	-7.210731000	-1.665205000	-0.558255000
6	-5.010301000	-0.558927000	-1.854431000
6	-6.077275000	-1.320658000	0.176171000
6	-4.965050000	-0.757229000	-0.468361000
8	-3.901677000	-0.390089000	0.293362000
6	4.230964000	-3.545745000	3.953762000
6	4.052354000	-3.499601000	2.433281000
6	3.767369000	-2.096007000	1.906328000
7	4.110732000	-1.877155000	0.606476000
8	3.271683000	-1.224511000	2.621981000
1	-1.037255000	8.275758000	0.974437000
1	-1.213023000	7.176944000	2.341492000
1	0.886209000	7.298928000	-0.834109000
1	-1.555961000	4.916487000	1.772890000
1	1.079906000	5.374356000	-2.392750000
1	-1.339597000	2.998633000	0.233329000
1	-0.422501000	2.227478000	-1.730825000
1	-8.344200000	-1.725299000	-3.803120000
1	-9.288093000	-0.941233000	-2.538417000
1	-6.170289000	-0.738487000	-3.646883000
1	-8.067204000	-2.092799000	-0.039894000
1	-4.159004000	-0.116958000	-2.357138000

1	-6.030789000	-1.480144000	1.248521000
1	-3.076462000	-0.256435000	-0.253355000
1	0.785873000	-3.374458000	4.348359000
1	0.979934000	-1.743462000	4.946182000
1	2.420829000	-6.191283000	-0.908969000
1	2.958595000	-4.728000000	-0.092140000
1	-0.733731000	-2.247335000	6.770132000
1	-1.012713000	-3.817300000	6.062806000
1	3.565436000	-5.613693000	-2.967527000
1	3.880780000	-3.961729000	-2.401770000
1	-0.354457000	1.943445000	4.001646000
1	-0.695840000	-4.116456000	2.102324000
1	2.470355000	-2.661101000	-3.257297000
1	-1.703269000	-2.824820000	3.958016000
1	1.044188000	-4.697839000	-2.487068000
1	-2.651940000	-0.487454000	2.225489000
1	-3.218633000	0.466555000	3.591552000
1	2.715237000	-1.164300000	-5.087900000
1	3.744012000	0.249584000	-5.379850000
1	2.548624000	5.777793000	1.644939000
1	3.111063000	5.141942000	0.124916000
1	4.414225000	4.571534000	2.851933000
1	-1.121501000	-3.635533000	-1.972970000
1	-1.267530000	-5.382632000	-1.900287000
1	3.788378000	3.334703000	-3.186429000
1	3.961805000	2.335292000	-4.610360000
1	-0.479148000	-0.108504000	5.932877000
1	-2.194235000	-0.617860000	6.012482000
1	-1.763987000	0.980036000	5.405313000
1	0.190428000	-6.768070000	-0.370436000
1	1.048815000	-5.984209000	0.973389000
1	-0.716306000	-6.011075000	0.940623000
1	5.914502000	2.305448000	-2.236163000
1	6.057783000	1.321614000	-3.680496000
1	-0.012996000	4.302689000	3.815634000
1	1.400149000	5.302765000	3.439473000
1	-9.742916000	3.142816000	-3.038769000
1	-11.292052000	3.994248000	-2.965313000
1	-9.692174000	4.339971000	-0.925106000
1	-11.353779000	3.922186000	-0.549873000
1	-9.798224000	2.626345000	0.857111000
1	-10.830051000	1.610513000	-0.139719000
1	-8.714504000	0.587186000	-0.124674000
1	-8.896418000	1.240584000	-1.735871000
1	-6.615948000	1.428908000	-0.672335000
1	-7.280052000	2.574885000	0.505452000

1	-5.687048000	3.378325000	-1.749791000
1	-0.199372000	-1.476310000	-2.298078000
1	0.094677000	0.043542000	-3.114319000
1	-2.580022000	-1.455430000	-3.074257000
1	-2.234510000	0.017990000	-4.013449000
1	4.861055000	-2.715269000	4.290587000
1	3.281663000	-3.478057000	4.489300000
1	4.929460000	-3.908941000	1.918878000
1	3.203486000	-4.129561000	2.130905000
1	-9.035972000	-2.679319000	-2.489595000
1	0.359487000	7.856792000	1.948055000
1	4.733268000	-4.477196000	4.236246000
6	-3.664294000	2.672526000	-1.658411000
1	-3.883779000	1.972739000	-0.866901000
1	-3.184288000	3.609440000	-1.418642000
1	-3.653375000	2.317276000	-2.678294000
1	1.178309000	-4.292361000	8.768977000
1	6.377995000	-4.852950000	-0.226490000
1	8.175027000	4.093539000	-4.164237000
1	5.428649000	-3.192179000	-4.572629000
1	3.404217000	3.682777000	5.768635000
1	-4.654406000	-3.258849000	2.933137000
1	-3.664778000	-2.428296000	1.767949000
1	4.385147000	-2.660054000	0.026767000
1	3.663440000	-1.098188000	0.124993000
1	-3.987897000	-5.335809000	0.388856000
1	-11.198290000	2.254560000	-2.605725000
1	3.398967000	3.263980000	-1.007982000
1	5.006440000	3.954367000	1.323680000
6	5.285811000	6.046851000	1.599271000
8	4.886978000	7.110061000	1.166235000
8	6.569631000	5.866088000	1.998778000
1	7.028006000	6.717505000	1.863064000
1	-2.886501000	-6.321470000	-0.508520000

TS_2
B-TS_2-opt.gjf.log

Temperature 298.150 Kelvin. Pressure 1.00000 Atm.

Zero-point correction= 1.450082 (Hartree/Particle)
Thermal correction to Energy= 1.552099
Thermal correction to Enthalpy= 1.553044
Thermal correction to Gibbs Free Energy= 1.289526
Sum of electronic and zero-point Energies= -5857.080881
Sum of electronic and thermal Energies= -5856.978864

Sum of electronic and thermal Enthalpies= -5856.977920
Sum of electronic and thermal Free Energies= -5857.241437

6	-0.362817000	7.294232000	2.250447000
6	-0.240270000	6.192488000	1.228231000
6	0.378082000	6.401164000	-0.013893000
6	-0.798771000	4.930664000	1.468058000
6	0.420110000	5.399273000	-0.983024000
6	-0.762900000	3.915240000	0.514773000
6	-0.163893000	4.146622000	-0.732994000
8	-0.126918000	3.213671000	-1.712634000
6	0.574721000	-4.073163000	3.887343000
6	3.204630000	-4.354724000	-2.492880000
6	5.669993000	5.161302000	-2.638377000
6	1.151891000	2.161211000	5.979628000
7	0.129487000	-1.661151000	1.822753000
6	0.176727000	-5.027375000	5.013493000
6	4.293403000	-3.733888000	-3.393091000
7	1.275378000	-2.262377000	-0.855075000
8	5.011691000	6.108676000	-3.020161000
8	0.729210000	1.233809000	6.640701000
7	1.251166000	1.023043000	1.705840000
8	1.008094000	-6.434726000	6.731769000
8	6.338930000	-5.043926000	-3.605803000
8	2.543117000	-5.432239000	5.424294000
8	6.083216000	-3.661961000	-1.845015000
6	-0.162764000	0.085893000	3.499288000
6	0.189776000	-3.817751000	0.669605000
6	2.345401000	-1.209073000	-2.778766000
6	2.561048000	2.562637000	0.304294000
28	1.134005000	-0.548133000	0.391619000
6	-0.386105000	-1.169107000	3.003819000
6	0.733894000	-3.456198000	-0.558063000
6	2.532241000	0.104824000	-2.267236000
6	1.918264000	2.214630000	1.493670000
6	-1.296134000	-2.233938000	3.631051000
6	0.707087000	-4.376181000	-1.782658000
6	3.021342000	1.229423000	-3.020716000
6	1.732323000	3.082162000	2.630963000
6	-0.614506000	-3.517263000	3.069109000
6	1.808016000	-3.707278000	-2.669094000
6	3.135955000	2.289007000	-2.138543000
6	0.927073000	2.389602000	3.513886000
6	-0.070646000	-2.980978000	1.754780000
6	1.817718000	-2.287262000	-2.117396000
6	2.687943000	1.802242000	-0.859196000

6	0.645413000	1.106532000	2.913055000
6	-2.689909000	-2.046840000	2.935833000
6	3.311362000	1.223018000	-4.500281000
6	2.291415000	4.463198000	2.791461000
7	-2.384090000	-5.664966000	-1.568566000
6	-3.551348000	-3.300099000	2.941669000
6	-1.901539000	-4.391421000	-1.651812000
6	4.677748000	0.621418000	-4.763593000
6	3.703745000	4.462490000	3.393024000
8	-3.996889000	-3.802514000	3.978832000
8	-2.424541000	-3.467694000	-1.023987000
8	5.743321000	1.183138000	-4.590046000
6	-0.668420000	-4.197915000	-2.524871000
6	3.621372000	3.675629000	-2.439593000
7	-3.778435000	-3.844484000	1.716635000
8	4.608211000	-0.663755000	-5.186997000
6	-1.448415000	-2.153750000	5.149719000
6	0.933919000	-5.858210000	-1.456893000
6	5.151672000	3.775962000	-2.342546000
6	0.416178000	2.856292000	4.851780000
6	1.377793000	-5.623775000	5.710196000
6	5.660755000	-4.233725000	-3.010765000
7	2.324828000	0.469459000	-0.972309000
8	7.009029000	5.248189000	-2.435190000
8	2.395038000	2.672789000	6.161976000
6	-10.708204000	3.552311000	-1.670420000
6	-10.382070000	3.645651000	-0.170345000
6	-9.981851000	2.296587000	0.455647000
6	-8.796087000	1.545783000	-0.174586000
6	-7.393038000	2.076513000	0.142241000
16	-6.930139000	3.606584000	-0.797735000
6	-0.718786000	-0.142641000	-2.524181000
8	-0.124280000	0.270942000	-5.523747000
16	-1.097951000	0.398390000	-0.806382000
6	-1.853184000	-0.094969000	-3.532160000
8	-2.437778000	-0.503409000	-6.077103000
16	-1.258078000	-0.641920000	-5.171663000
8	-0.825722000	-2.064624000	-4.977353000
6	-8.568318000	-1.282945000	-2.737414000
6	-7.283624000	-1.145751000	-1.955577000
6	-6.199201000	-0.423466000	-2.469291000
6	-7.148641000	-1.692010000	-0.669982000
6	-5.028542000	-0.234852000	-1.732625000
6	-5.985690000	-1.519366000	0.078438000
6	-4.913029000	-0.782264000	-0.446972000
8	-3.820829000	-0.605608000	0.338606000

6	4.602657000	-3.871250000	2.822290000
6	4.376832000	-3.629568000	1.323429000
6	3.877852000	-2.232923000	0.987602000
7	4.414618000	-1.647905000	-0.106365000
8	3.012378000	-1.672023000	1.674569000
1	-0.847451000	8.179276000	1.814900000
1	-0.991788000	6.970637000	3.088338000
1	0.826156000	7.368913000	-0.232610000
1	-1.267317000	4.728345000	2.429027000
1	0.894151000	5.569805000	-1.945530000
1	-1.172406000	2.935979000	0.735031000
1	-0.466498000	2.339631000	-1.358009000
1	-8.398272000	-1.134636000	-3.809646000
1	-9.306559000	-0.532623000	-2.421702000
1	-6.267317000	0.011505000	-3.464023000
1	-7.971063000	-2.265321000	-0.245713000
1	-4.208404000	0.342222000	-2.141633000
1	-5.879597000	-1.957440000	1.065926000
1	-3.014909000	-0.300416000	-0.176691000
1	1.232990000	-4.618089000	3.201952000
1	1.174396000	-3.245415000	4.282806000
1	3.149795000	-5.425085000	-2.716429000
1	3.505912000	-4.259095000	-1.443866000
1	-0.445393000	-4.546900000	5.774650000
1	-0.426809000	-5.858553000	4.624150000
1	4.115462000	-3.985168000	-4.441898000
1	4.288619000	-2.645879000	-3.290524000
1	-0.607735000	0.324543000	4.458966000
1	-0.146479000	-4.843119000	0.778470000
1	2.630162000	-1.364960000	-3.812060000
1	-1.330161000	-4.328426000	2.902112000
1	1.531352000	-3.724472000	-3.728961000
1	-2.551134000	-1.703985000	1.911655000
1	-3.238337000	-1.258528000	3.462265000
1	2.534484000	0.675790000	-5.043822000
1	3.337545000	2.249146000	-4.879426000
1	1.638392000	5.062916000	3.433949000
1	2.303239000	4.983784000	1.828869000
1	3.703717000	3.948178000	4.362793000
1	-0.732633000	-3.198511000	-2.960521000
1	-0.681378000	-4.904215000	-3.364480000
1	3.160794000	4.401266000	-1.761723000
1	3.314436000	3.974722000	-3.448725000
1	-0.485135000	-2.230432000	5.663473000
1	-2.120026000	-2.942731000	5.499476000
1	-1.896415000	-1.195653000	5.435973000

1	1.030166000	-6.444246000	-2.378575000
1	1.834088000	-6.010413000	-0.854530000
1	0.088779000	-6.266169000	-0.895437000
1	5.501549000	3.481901000	-1.345158000
1	5.620525000	3.076119000	-3.046495000
1	-0.648181000	2.634721000	4.974076000
1	0.556922000	3.937282000	4.952065000
1	-9.792380000	3.559160000	-2.272089000
1	-11.335938000	4.385536000	-2.004624000
1	-9.592714000	4.390851000	-0.009667000
1	-11.263230000	4.013421000	0.373446000
1	-9.777745000	2.441349000	1.526518000
1	-10.858641000	1.634519000	0.399891000
1	-8.813858000	0.513801000	0.199948000
1	-8.919403000	1.468950000	-1.261206000
1	-6.658937000	1.314756000	-0.128224000
1	-7.298142000	2.277936000	1.214718000
1	-5.521169000	3.349449000	-0.788281000
1	-0.348735000	-1.161860000	-2.473818000
1	0.099094000	0.475372000	-2.895773000
1	-2.668425000	-0.772717000	-3.264656000
1	-2.257309000	0.915154000	-3.659110000
1	5.232856000	-3.087029000	3.256852000
1	3.664118000	-3.908704000	3.379698000
1	5.288797000	-3.845906000	0.756144000
1	3.605938000	-4.320536000	0.953690000
1	-9.042873000	-2.264720000	-2.608889000
1	0.576754000	7.652801000	2.690412000
1	5.124609000	-4.827115000	2.938890000
6	-3.856129000	2.866775000	-0.703705000
1	-3.963800000	1.955028000	-0.132908000
1	-3.445477000	3.733087000	-0.202994000
1	-3.661389000	2.763666000	-1.762681000
1	1.830340000	-6.781485000	7.128107000
1	6.958171000	-4.037377000	-1.629816000
1	7.266477000	6.163144000	-2.658628000
1	5.526007000	-0.985507000	-5.278906000
1	2.825663000	2.135086000	6.853792000
1	-4.320547000	-4.697139000	1.673276000
1	-3.374846000	-3.492329000	0.850352000
1	4.991779000	-2.181674000	-0.744331000
1	3.920225000	-0.832372000	-0.466207000
1	-3.259441000	-5.813242000	-1.083838000
1	-11.224200000	2.609918000	-1.893357000
1	2.943748000	3.577165000	0.253793000
1	4.406221000	3.910682000	2.757147000

6	4.239657000	5.857552000	3.607428000
8	3.612518000	6.892792000	3.493954000
8	5.546703000	5.842945000	3.969608000
1	5.811653000	6.773483000	4.101771000
1	-2.072681000	-6.387468000	-2.200847000

Int_2
B-Int_2-opt.gjf.log

Temperature 298.150 Kelvin. Pressure 1.00000 Atm.

Zero-point correction= 1.454904 (Hartree/Particle)
Thermal correction to Energy= 1.558276
Thermal correction to Enthalpy= 1.559220
Thermal correction to Gibbs Free Energy= 1.291382
Sum of electronic and zero-point Energies= -5857.116571
Sum of electronic and thermal Energies= -5857.013199
Sum of electronic and thermal Enthalpies= -5857.012255
Sum of electronic and thermal Free Energies= -5857.280093

6	-0.855213000	7.080536000	2.652014000
6	-0.668436000	6.042750000	1.574171000
6	0.021590000	6.326699000	0.385719000
6	-1.238192000	4.770068000	1.703406000
6	0.136116000	5.380370000	-0.631562000
6	-1.138778000	3.812038000	0.696208000
6	-0.446739000	4.111064000	-0.486061000
8	-0.324233000	3.227030000	-1.506656000
6	0.932283000	-4.224779000	3.740987000
6	3.377922000	-4.079667000	-2.727630000
6	5.270835000	5.546263000	-2.577381000
6	1.071360000	1.980114000	6.063560000
7	0.286688000	-1.775272000	1.788304000
6	0.627491000	-5.245615000	4.837122000
6	4.404921000	-3.348938000	-3.618078000
7	1.384659000	-2.187618000	-0.944750000
8	4.538002000	6.480031000	-2.836699000
8	0.721781000	1.001869000	6.692488000
7	1.223887000	0.983250000	1.754901000
8	1.599202000	-6.642137000	6.488869000
8	6.513278000	-4.528627000	-3.944820000
8	3.025845000	-5.491124000	5.180617000
8	6.228740000	-3.250828000	-2.110917000
6	-0.092423000	-0.110537000	3.531164000
6	0.447112000	-3.874081000	0.539469000
6	2.324293000	-0.980516000	-2.846753000

6	2.388330000	2.662522000	0.387052000
28	1.179635000	-0.539667000	0.383900000
6	-0.238044000	-1.363363000	2.992756000
6	0.922953000	-3.427349000	-0.686769000
6	2.448922000	0.312194000	-2.283601000
6	1.787273000	2.227866000	1.569359000
6	-1.062929000	-2.507323000	3.595106000
6	0.907795000	-4.286225000	-1.953852000
6	2.840863000	1.502031000	-3.004732000
6	1.547209000	3.048815000	2.740397000
6	-0.311538000	-3.721674000	2.969932000
6	1.940210000	-3.511583000	-2.837378000
6	2.920499000	2.524181000	-2.083194000
6	0.813449000	2.271704000	3.607321000
6	0.167035000	-3.101443000	1.668874000
6	1.882842000	-2.122809000	-2.216964000
6	2.540175000	1.954030000	-0.808008000
6	0.627251000	0.982138000	2.969247000
6	-2.479867000	-2.384461000	2.931639000
6	3.061453000	1.575535000	-4.493446000
6	2.009958000	4.459339000	2.938751000
7	-2.115944000	-5.756377000	-1.746615000
6	-3.260194000	-3.690486000	2.917406000
6	-1.689361000	-4.460967000	-1.749747000
6	4.435439000	1.042181000	-4.851435000
6	3.423960000	4.534134000	3.532268000
8	-3.643158000	-4.252049000	3.948927000
8	-2.222871000	-3.608148000	-1.036176000
8	5.486840000	1.629157000	-4.677662000
6	-0.498266000	-4.150115000	-2.646645000
6	3.316816000	3.947657000	-2.335544000
7	-3.483788000	-4.209703000	1.681673000
8	4.389976000	-0.209381000	-5.366339000
6	-1.186646000	-2.493671000	5.118461000
6	1.225808000	-5.767053000	-1.710252000
6	4.843733000	4.120746000	-2.328491000
6	0.279198000	2.655724000	4.961337000
6	1.884915000	-5.775651000	5.487011000
6	5.808245000	-3.788049000	-3.293737000
7	2.263999000	0.610530000	-0.966757000
8	6.616857000	5.687155000	-2.484901000
8	2.271985000	2.578677000	6.259244000
6	-10.985847000	2.765964000	-1.241176000
6	-10.432626000	2.741718000	0.189153000
6	-9.485709000	1.563471000	0.473123000
6	-8.188441000	1.487760000	-0.349877000

6	-7.138763000	2.566822000	-0.038526000
16	-7.437230000	4.237386000	-0.731232000
6	-0.777131000	-0.107968000	-2.450074000
8	-0.241143000	0.480710000	-5.423545000
16	-1.140183000	0.316732000	-0.698528000
6	-1.929936000	-0.059004000	-3.436864000
8	-2.515818000	-0.388469000	-5.996040000
16	-1.324461000	-0.503426000	-5.103616000
8	-0.815624000	-1.908577000	-4.972688000
6	-8.522677000	-1.842907000	-2.578527000
6	-7.235918000	-1.636995000	-1.814552000
6	-6.234892000	-0.788559000	-2.305538000
6	-7.031421000	-2.219592000	-0.555063000
6	-5.083024000	-0.508751000	-1.569007000
6	-5.882497000	-1.960048000	0.191179000
6	-4.896754000	-1.095155000	-0.308795000
8	-3.811396000	-0.852170000	0.469463000
6	4.926067000	-3.700181000	2.595058000
6	4.629766000	-3.432077000	1.112329000
6	4.035329000	-2.061547000	0.834314000
7	4.507071000	-1.409324000	-0.250707000
8	3.151357000	-1.580138000	1.558187000
1	-1.414777000	7.945937000	2.269399000
1	-1.440921000	6.669888000	3.482941000
1	0.474564000	7.307331000	0.251240000
1	-1.776744000	4.517359000	2.614520000
1	0.667739000	5.607700000	-1.551032000
1	-1.581727000	2.829719000	0.819287000
1	-0.627780000	2.321745000	-1.200497000
1	-8.387609000	-1.635229000	-3.646103000
1	-9.306396000	-1.161448000	-2.217000000
1	-6.360579000	-0.318846000	-3.278850000
1	-7.788199000	-2.887479000	-0.146994000
1	-4.331120000	0.166376000	-1.959968000
1	-5.719868000	-2.428703000	1.156881000
1	-3.048387000	-0.464234000	-0.047838000
1	1.610975000	-4.696253000	3.021823000
1	1.482609000	-3.372745000	4.156080000
1	3.378985000	-5.138228000	-3.006363000
1	3.700014000	-4.020934000	-1.681905000
1	-0.011373000	-4.839715000	5.627180000
1	0.078268000	-6.104313000	4.427867000
1	4.214444000	-3.555237000	-4.674361000
1	4.341379000	-2.269703000	-3.457943000
1	-0.537132000	0.061968000	4.504614000
1	0.175549000	-4.921448000	0.611972000

1	2.570151000	-1.072602000	-3.897274000
1	-0.975761000	-4.571574000	2.785891000
1	1.634055000	-3.492895000	-3.888839000
1	-2.386154000	-2.002159000	1.915687000
1	-3.064500000	-1.648196000	3.492817000
1	2.276900000	1.025638000	-5.023697000
1	3.031293000	2.618905000	-4.821481000
1	1.321765000	4.992091000	3.602401000
1	1.978589000	5.007051000	1.992341000
1	3.466049000	3.991285000	4.485330000
1	-0.633098000	-3.135137000	-3.027043000
1	-0.495303000	-4.811420000	-3.521769000
1	2.869199000	4.611678000	-1.590357000
1	2.929311000	4.282775000	-3.304725000
1	-0.209173000	-2.523256000	5.609690000
1	-1.795156000	-3.339380000	5.449728000
1	-1.692820000	-1.580389000	5.450028000
1	1.330547000	-6.298431000	-2.663349000
1	2.148278000	-5.897839000	-1.137385000
1	0.418404000	-6.250192000	-1.152750000
1	5.270779000	3.796098000	-1.371281000
1	5.301930000	3.482851000	-3.095226000
1	-0.765222000	2.352409000	5.078399000
1	0.340537000	3.740152000	5.095331000
1	-10.191177000	2.968228000	-1.966874000
1	-11.752046000	3.541396000	-1.359090000
1	-9.918607000	3.690130000	0.396281000
1	-11.270071000	2.685125000	0.899617000
1	-9.217796000	1.572336000	1.540084000
1	-10.042836000	0.628305000	0.308440000
1	-7.714738000	0.520899000	-0.140168000
1	-8.407093000	1.488869000	-1.424490000
1	-6.180536000	2.245975000	-0.470055000
1	-6.985608000	2.642346000	1.045160000
1	-4.222235000	3.907112000	-1.087946000
1	-0.358010000	-1.109553000	-2.468347000
1	0.002653000	0.567990000	-2.802693000
1	-2.712244000	-0.783166000	-3.193095000
1	-2.378068000	0.937392000	-3.508578000
1	5.515056000	-2.886758000	3.033301000
1	4.013945000	-3.824998000	3.182828000
1	5.532079000	-3.575949000	0.507964000
1	3.889931000	-4.159844000	0.749776000
1	-8.921058000	-2.862054000	-2.487750000
1	0.065403000	7.487201000	3.090229000
1	5.519079000	-4.619077000	2.655848000

6	-3.775758000	3.062071000	-1.612661000
1	-3.599653000	2.244831000	-0.914771000
1	-2.819171000	3.353301000	-2.042437000
1	-4.453388000	2.728403000	-2.399388000
1	2.453167000	-6.941717000	6.855545000
1	7.128678000	-3.585805000	-1.935306000
1	6.813164000	6.626214000	-2.666655000
1	5.312598000	-0.490136000	-5.522589000
1	2.746127000	2.055597000	6.933719000
1	-3.980012000	-5.089025000	1.623090000
1	-3.132538000	-3.801783000	0.816604000
1	5.110225000	-1.877730000	-0.915521000
1	3.965639000	-0.606023000	-0.564567000
1	-2.969295000	-5.976728000	-1.249948000
1	-11.436424000	1.799155000	-1.498696000
1	2.695475000	3.702666000	0.367441000
1	4.157747000	4.051864000	2.875512000
6	3.865277000	5.955623000	3.787192000
8	3.168915000	6.948429000	3.705427000
8	5.171359000	6.017379000	4.146237000
1	5.374786000	6.959034000	4.306025000
1	-1.800675000	-6.414038000	-2.444582000

Int_3
B-Int_3-opt.gjf.log

Temperature 298.150 Kelvin. Pressure 1.00000 Atm.

Zero-point correction= 1.460180 (Hartree/Particle)
Thermal correction to Energy= 1.562258
Thermal correction to Enthalpy= 1.563202
Thermal correction to Gibbs Free Energy= 1.305776
Sum of electronic and zero-point Energies= -5857.134551
Sum of electronic and thermal Energies= -5857.032473
Sum of electronic and thermal Enthalpies= -5857.031529
Sum of electronic and thermal Free Energies= -5857.288955

6	-3.634976000	-7.103880000	0.261676000
6	-3.199767000	-5.878384000	-0.497080000
6	-3.957366000	-5.307467000	-1.528718000
6	-1.929137000	-5.340963000	-0.246915000
6	-3.459480000	-4.246883000	-2.289014000
6	-1.413305000	-4.292315000	-0.999520000
6	-2.180559000	-3.735734000	-2.031158000
8	-1.725932000	-2.702611000	-2.792280000
6	3.410975000	2.717694000	3.499937000

6	-0.736110000	5.284424000	-0.995418000
6	-6.769518000	-1.081650000	-0.308346000
6	-1.999224000	-2.463230000	5.397882000
7	1.032397000	0.624232000	1.710115000
6	4.592775000	2.650532000	4.467753000
6	-2.147919000	5.358103000	-1.575073000
7	0.292740000	2.169653000	-0.710736000
8	-6.559380000	-2.282102000	-0.242404000
8	-1.172907000	-1.984065000	6.148980000
7	-1.337760000	-1.073771000	1.271983000
8	6.121066000	3.944236000	5.732894000
8	-2.674789000	7.332306000	-0.236567000
8	4.584860000	5.084564000	4.544362000
8	-4.231702000	6.464388000	-1.617796000
6	0.211515000	-1.128152000	3.192291000
6	2.231786000	2.501890000	0.726681000
6	-1.471839000	2.448056000	-2.382604000
6	-3.306812000	-1.461068000	-0.160753000
28	-0.392865000	0.344245000	0.119811000
6	1.065757000	-0.109840000	2.873949000
6	1.390401000	2.852492000	-0.328635000
6	-2.269301000	1.303728000	-2.125703000
6	-2.516020000	-1.727552000	0.952872000
6	2.166922000	0.375906000	3.828657000
6	1.600940000	4.130114000	-1.168503000
6	-3.534530000	1.029345000	-2.754260000
6	-2.835091000	-2.690354000	1.975892000
6	2.965285000	1.365096000	2.905731000
6	0.170103000	4.293063000	-1.774306000
6	-4.057304000	-0.093370000	-2.138039000
6	-1.827768000	-2.603850000	2.914685000
6	2.031026000	1.522920000	1.701807000
6	-0.382858000	2.881723000	-1.674823000
6	-3.116139000	-0.466263000	-1.115682000
6	-0.915500000	-1.581888000	2.457478000
6	2.991807000	-0.855481000	4.323342000
6	-4.230045000	1.965335000	-3.702927000
6	-4.103461000	-3.487691000	2.036639000
6	4.313187000	-0.516736000	4.985907000
6	4.064840000	3.906618000	-2.036617000
6	-4.712210000	3.199498000	-2.946369000
6	-5.235139000	-2.635807000	2.638997000
8	4.398399000	-0.113381000	6.148517000
8	4.783804000	4.875467000	-2.283187000
8	-5.272499000	3.176687000	-1.860889000
6	2.577615000	3.904912000	-2.370959000

6	-5.380699000	-0.742807000	-2.430627000
8	-4.434770000	4.349360000	-3.582573000
6	1.494136000	1.072038000	5.031554000
6	2.087559000	5.325692000	-0.334627000
6	-6.540900000	-0.233807000	-1.541657000
6	-1.704885000	-3.361901000	4.211345000
6	5.062172000	4.021555000	4.888742000
6	-2.985645000	6.491693000	-1.050766000
7	-2.034953000	0.403188000	-1.135010000
8	-7.284011000	-0.379097000	0.716775000
8	-3.324395000	-2.209740000	5.523067000
6	7.673516000	-6.699785000	-2.292786000
6	6.453903000	-6.318780000	-1.437500000
6	5.539518000	-5.213963000	-1.997308000
6	4.297724000	-5.019683000	-1.106113000
6	3.495683000	-3.721533000	-1.333712000
16	2.126648000	-3.738571000	-2.560480000
6	0.943802000	-0.444715000	-2.966561000
8	2.171355000	-0.247963000	-5.899231000
16	0.826520000	-1.351474000	-1.360553000
6	2.348681000	0.036725000	-3.271218000
8	3.964517000	1.219551000	-4.951578000
16	2.527660000	0.809086000	-4.904185000
8	1.575904000	1.965171000	-4.905950000
6	7.605652000	-1.381861000	-2.323953000
6	6.466553000	-1.351393000	-1.330882000
6	5.362432000	-0.510281000	-1.525285000
6	6.510722000	-2.123527000	-0.160145000
6	4.311433000	-0.491781000	-0.611203000
6	5.455537000	-2.127260000	0.753481000
6	4.329120000	-1.341691000	0.493853000
8	3.229294000	-1.488494000	1.291708000
6	-4.355005000	4.752087000	3.189801000
6	-3.812362000	4.665517000	1.751778000
6	-3.087566000	3.340677000	1.506107000
7	-3.586295000	2.538545000	0.537923000
8	-2.098897000	3.031939000	2.181550000
1	-3.449886000	-8.009720000	-0.335280000
1	-3.039465000	-7.215440000	1.176010000
1	-4.949841000	-5.697258000	-1.745773000
1	-1.321193000	-5.761396000	0.551520000
1	-4.051595000	-3.800122000	-3.082593000
1	-0.424735000	-3.895902000	-0.798078000
1	-0.952070000	-2.288280000	-2.331941000
1	7.290468000	-1.047853000	-3.316703000
1	8.049002000	-2.379691000	-2.409942000

1	5.296360000	0.105479000	-2.419620000
1	7.364791000	-2.773421000	0.017658000
1	3.411943000	0.081956000	-0.793409000
1	5.452526000	-2.802843000	1.604772000
1	2.445933000	-1.130845000	0.827524000
1	3.710466000	3.378265000	2.682527000
1	2.565790000	3.218627000	3.980725000
1	-0.286375000	6.281535000	-1.022158000
1	-0.795031000	4.985816000	0.058116000
1	4.369601000	2.083132000	5.375752000
1	5.444497000	2.132670000	4.008871000
1	-2.127730000	5.448947000	-2.668289000
1	-2.697965000	4.435875000	-1.366799000
1	0.367908000	-1.607061000	4.152011000
1	3.094934000	3.137810000	0.863791000
1	-1.823190000	3.106808000	-3.169022000
1	3.861638000	0.852507000	2.530624000
1	0.219523000	4.627336000	-2.816402000
1	3.153585000	-1.523186000	3.478283000
1	2.398835000	-1.389437000	5.072031000
1	-3.594852000	2.282153000	-4.535055000
1	-5.117131000	1.487632000	-4.135827000
1	-3.972061000	-4.393381000	2.638272000
1	-4.394225000	-3.825453000	1.038124000
1	-5.020042000	-2.416356000	3.692864000
1	2.291928000	3.006133000	-2.926091000
1	2.447067000	4.749225000	-3.053661000
1	-5.308744000	-1.829802000	-2.322615000
1	-5.639913000	-0.555113000	-3.477952000
1	0.882675000	1.920056000	4.706013000
1	2.234824000	1.421617000	5.756088000
1	0.833585000	0.362661000	5.540846000
1	2.104857000	6.233643000	-0.947126000
1	1.453615000	5.500894000	0.538393000
1	3.107922000	5.165482000	0.025100000
1	-6.395461000	0.813070000	-1.261365000
1	-7.485910000	-0.283925000	-2.101137000
1	-0.695488000	-3.759744000	4.350931000
1	-2.409476000	-4.199550000	4.222875000
1	7.382457000	-6.832338000	-3.343072000
1	8.083813000	-7.659503000	-1.952142000
1	5.844576000	-7.223817000	-1.297470000
1	6.786712000	-6.026372000	-0.430890000
1	6.093920000	-4.267214000	-2.052744000
1	5.237560000	-5.462208000	-3.024591000
1	3.642755000	-5.898782000	-1.176392000

1	4.647057000	-4.981785000	-0.063529000
1	4.186158000	-2.926862000	-1.610037000
1	3.030262000	-3.435275000	-0.388896000
1	-0.575427000	-3.169068000	-6.789292000
1	0.249551000	0.395909000	-2.923348000
1	0.611467000	-1.124314000	-3.754036000
1	2.662980000	0.798240000	-2.555541000
1	3.058470000	-0.792486000	-3.237256000
1	-5.294506000	4.199497000	3.297201000
1	-3.621525000	4.317212000	3.877047000
1	-4.609075000	4.829322000	1.016700000
1	-3.064054000	5.451210000	1.598628000
1	8.378360000	-0.693590000	-1.956796000
1	-4.685684000	-7.166999000	0.572882000
1	-4.514392000	5.789586000	3.502727000
6	0.022936000	-3.016076000	-5.888419000
1	0.672234000	-3.879055000	-5.732485000
1	-0.634275000	-2.909690000	-5.025111000
1	0.635937000	-2.121187000	-5.995313000
1	6.365549000	4.861589000	5.960701000
1	-4.750602000	7.200497000	-1.241412000
1	-7.464142000	-1.013440000	1.450592000
1	-4.667666000	5.092951000	-2.980729000
1	-3.427304000	-1.579477000	6.261782000
1	-4.308602000	2.836791000	-0.108270000
1	-3.038184000	1.727290000	0.263207000
1	8.512338000	-5.992536000	-2.274838000
1	-4.190247000	-2.075152000	-0.279751000
1	-5.295788000	-1.669275000	2.131891000
6	-6.577754000	-3.309241000	2.568806000
8	-6.795537000	-4.492545000	2.442936000
8	-7.603414000	-2.404630000	2.706487000
1	-8.438058000	-2.909244000	2.648282000
7	5.413528000	-0.677926000	4.198760000
1	5.356011000	-0.972048000	3.233106000
1	6.318573000	-0.417728000	4.566974000
7	4.560570000	2.774668000	-1.466206000
1	3.956102000	2.055396000	-1.100733000
1	5.523528000	2.771706000	-1.158253000

TS_3
B-TS_3-opt.gjf.log

Temperature 298.150 Kelvin. Pressure 1.00000 Atm.

Zero-point correction= 1.459581 (Hartree/Particle)

Thermal correction to Energy= 1.560981
Thermal correction to Enthalpy= 1.561925
Thermal correction to Gibbs Free Energy= 1.305739
Sum of electronic and zero-point Energies= -5857.126808
Sum of electronic and thermal Energies= -5857.025408
Sum of electronic and thermal Enthalpies= -5857.024464
Sum of electronic and thermal Free Energies= -5857.280649

6	-4.315770000	-6.847395000	0.978715000
6	-3.893376000	-5.749126000	0.041699000
6	-4.683865000	-5.283667000	-1.017416000
6	-2.597127000	-5.232113000	0.162994000
6	-4.191949000	-4.344533000	-1.928643000
6	-2.090413000	-4.303817000	-0.736547000
6	-2.886476000	-3.855408000	-1.796656000
8	-2.434022000	-2.943284000	-2.708601000
6	3.695491000	2.649382000	3.413474000
6	-0.177547000	5.239323000	-1.310540000
6	-6.697649000	-0.491287000	-0.309494000
6	-2.057392000	-2.008083000	5.498875000
7	1.202309000	0.606252000	1.671775000
6	4.781749000	2.561042000	4.484813000
6	-1.573876000	5.381569000	-1.913798000
7	0.597953000	2.075130000	-0.808471000
8	-6.621162000	-1.698086000	-0.144462000
8	-1.180855000	-1.563132000	6.214083000
7	-1.286112000	-0.918948000	1.305225000
8	6.340894000	3.816388000	5.751798000
8	-2.006254000	7.415404000	-0.632397000
8	5.112023000	4.963205000	4.253174000
8	-3.550767000	6.658980000	-2.090519000
6	0.246596000	-1.015885000	3.229580000
6	2.518967000	2.384686000	0.660780000
6	-1.110845000	2.388916000	-2.532498000
6	-3.290963000	-1.192784000	-0.098927000
28	-0.269585000	0.426684000	0.143420000
6	1.160715000	-0.060443000	2.878022000
6	1.732876000	2.710568000	-0.444588000
6	-2.004155000	1.337567000	-2.212834000
6	-2.525890000	-1.467814000	1.030373000
6	2.206966000	0.473162000	3.870487000
6	2.061997000	3.901266000	-1.371645000
6	-3.283420000	1.131367000	-2.845588000
6	-2.938330000	-2.337369000	2.105346000
6	3.133542000	1.303174000	2.913306000
6	0.658126000	4.133722000	-2.010580000

6	-3.904506000	0.100791000	-2.167774000
6	-1.916406000	-2.312680000	3.029652000
6	2.243263000	1.459425000	1.669332000
6	0.001661000	2.774923000	-1.832482000
6	-3.004097000	-0.281872000	-1.109316000
6	-0.909674000	-1.412270000	2.511780000
6	2.860981000	-0.732298000	4.628987000
6	-3.884801000	2.056919000	-3.865815000
6	-4.288872000	-2.978247000	2.217603000
6	4.243569000	-0.498389000	5.209871000
6	4.510586000	3.445373000	-2.205060000
6	-4.265244000	3.378569000	-3.206094000
6	-5.312714000	-1.964998000	2.759808000
8	4.431167000	0.006026000	6.319463000
8	5.303955000	4.293615000	-2.615262000
8	-4.820833000	3.482163000	-2.122026000
6	3.025401000	3.503035000	-2.540688000
6	-5.273983000	-0.455710000	-2.436637000
8	-3.904527000	4.453133000	-3.925630000
6	1.476207000	1.372891000	4.893223000
6	2.641089000	5.114229000	-0.627736000
6	-6.397458000	0.218751000	-1.612292000
6	-1.862896000	-3.002126000	4.369124000
6	5.394834000	3.907246000	4.783989000
6	-2.339315000	6.591062000	-1.454705000
7	-1.848148000	0.484291000	-1.166799000
8	-7.114043000	0.346928000	0.656923000
8	-3.350699000	-1.621337000	5.616125000
6	6.790845000	-7.098067000	-2.360507000
6	5.681238000	-6.604267000	-1.413395000
6	4.719379000	-5.534284000	-1.966989000
6	3.634168000	-5.183548000	-0.926498000
6	2.913517000	-3.832198000	-1.100967000
16	1.387056000	-3.834335000	-2.142649000
6	0.774612000	-0.866017000	-2.979758000
8	2.260458000	-0.903659000	-5.748102000
16	0.474851000	-1.822999000	-1.432091000
6	2.237557000	-0.517820000	-3.138756000
8	4.156967000	0.341424000	-4.689537000
16	2.673857000	0.147254000	-4.768361000
8	1.910168000	1.425022000	-4.909557000
6	6.858642000	-1.815087000	-2.969277000
6	6.030245000	-1.836982000	-1.702854000
6	5.024846000	-0.880871000	-1.521337000
6	6.316830000	-2.706316000	-0.641072000
6	4.279683000	-0.828799000	-0.343181000

6	5.587527000	-2.661614000	0.547459000
6	4.548445000	-1.739029000	0.677937000
8	3.786665000	-1.792570000	1.826199000
6	-4.520238000	5.271946000	2.632618000
6	-3.745539000	5.014972000	1.326413000
6	-2.997666000	3.677517000	1.355055000
7	-3.246778000	2.820168000	0.340688000
8	-2.196677000	3.424907000	2.262011000
1	-4.192602000	-7.828481000	0.495105000
1	-3.663258000	-6.861307000	1.860112000
1	-5.698400000	-5.658003000	-1.136742000
1	-1.964328000	-5.563544000	0.983202000
1	-4.810063000	-3.979957000	-2.744061000
1	-1.087611000	-3.917323000	-0.613279000
1	-1.629922000	-2.507888000	-2.345620000
1	6.291289000	-1.424605000	-3.818861000
1	7.284602000	-2.792431000	-3.216057000
1	4.811950000	-0.191561000	-2.332253000
1	7.105266000	-3.447624000	-0.750779000
1	3.448379000	-0.137329000	-0.232137000
1	5.777151000	-3.366053000	1.351934000
1	2.954214000	-1.304339000	1.689071000
1	4.137518000	3.174893000	2.563194000
1	2.882695000	3.293500000	3.761992000
1	0.348003000	6.195681000	-1.395002000
1	-0.269802000	5.017429000	-0.240726000
1	4.420406000	2.141568000	5.427290000
1	5.590838000	1.891029000	4.167211000
1	-1.537308000	5.411559000	-3.009575000
1	-2.187718000	4.511622000	-1.661601000
1	0.353585000	-1.443111000	4.219983000
1	3.402622000	2.991399000	0.798343000
1	-1.395804000	3.023233000	-3.365050000
1	3.988494000	0.679389000	2.617216000
1	0.744796000	4.398674000	-3.070288000
1	2.896638000	-1.600009000	3.969435000
1	2.213352000	-0.994633000	5.470118000
1	-3.216961000	2.260158000	-4.708102000
1	-4.806449000	1.629354000	-4.279312000
1	-4.259447000	-3.852108000	2.877377000
1	-4.624952000	-3.344043000	1.243845000
1	-5.065598000	-1.698571000	3.795684000
1	2.690522000	2.569177000	-3.003718000
1	2.946382000	4.278873000	-3.307205000
1	-5.295768000	-1.533734000	-2.247379000
1	-5.503687000	-0.326707000	-3.499949000

1	0.975719000	2.210134000	4.395348000
1	2.169827000	1.769288000	5.641771000
1	0.711245000	0.786921000	5.412992000
1	2.741418000	5.966131000	-1.309371000
1	2.016618000	5.412141000	0.218507000
1	3.640281000	4.897044000	-0.238487000
1	-6.172672000	1.272631000	-1.427341000
1	-7.340121000	0.186909000	-2.177212000
1	-0.897870000	-3.490714000	4.533505000
1	-2.648890000	-3.761679000	4.431017000
1	6.388875000	-7.294263000	-3.362982000
1	7.203899000	-8.045082000	-1.989886000
1	5.080594000	-7.473947000	-1.108915000
1	6.136793000	-6.220121000	-0.489129000
1	5.282955000	-4.628659000	-2.222226000
1	4.259018000	-5.890709000	-2.898905000
1	2.907452000	-6.003624000	-0.847976000
1	4.128629000	-5.123778000	0.053105000
1	3.604851000	-3.100985000	-1.515059000
1	2.600417000	-3.470193000	-0.118831000
1	-1.048545000	-3.267514000	-6.722574000
1	0.157830000	0.029321000	-2.900113000
1	0.422661000	-1.463958000	-3.820742000
1	2.531978000	0.226221000	-2.395731000
1	2.859503000	-1.405212000	-3.015233000
1	-5.512289000	4.808593000	2.610878000
1	-3.959767000	4.842530000	3.469930000
1	-4.399160000	5.088223000	0.449456000
1	-2.967474000	5.778599000	1.209663000
1	7.672814000	-1.117418000	-2.732535000
1	-5.343225000	-6.841620000	1.364921000
1	-4.630249000	6.341805000	2.840037000
6	-0.452031000	-3.246322000	-5.808427000
1	0.040368000	-4.210786000	-5.674847000
1	-1.104170000	-3.062277000	-4.953812000
1	0.304943000	-2.464743000	-5.878230000
1	6.689573000	4.718110000	5.888090000
1	-4.031666000	7.432535000	-1.739654000
1	-7.351688000	-0.200655000	1.442329000
1	-4.075461000	5.253392000	-3.377719000
1	-3.382683000	-0.936203000	6.311141000
1	-3.868354000	3.041281000	-0.429832000
1	-2.667523000	1.989847000	0.243300000
1	7.646860000	-6.421409000	-2.476695000
1	-4.229738000	-1.725316000	-0.185365000
1	-5.268521000	-1.035496000	2.185976000

6	-6.722904000	-2.486247000	2.738050000
8	-7.074540000	-3.643432000	2.701327000
8	-7.639483000	-1.463940000	2.806007000
1	-8.525837000	-1.874397000	2.784344000
7	5.263123000	-0.890196000	4.399786000
1	5.079436000	-1.296056000	3.489834000
1	6.217224000	-0.742491000	4.698722000
7	4.916664000	2.397005000	-1.440178000
1	4.258821000	1.781461000	-0.986981000
1	5.883270000	2.352491000	-1.148343000

PC
B-PC-opt.gjf.log

Temperature 298.150 Kelvin. Pressure 1.00000 Atm.

Zero-point correction= 1.458937 (Hartree/Particle)
Thermal correction to Energy= 1.560332
Thermal correction to Enthalpy= 1.561276
Thermal correction to Gibbs Free Energy= 1.303775
Sum of electronic and zero-point Energies= -5857.167837
Sum of electronic and thermal Energies= -5857.066443
Sum of electronic and thermal Enthalpies= -5857.065498
Sum of electronic and thermal Free Energies= -5857.322999

6	-7.262727000	-1.155923000	4.895964000
6	-6.300073000	-1.615803000	3.825317000
6	-6.477902000	-2.838053000	3.157714000
6	-5.142281000	-0.882158000	3.533926000
6	-5.528524000	-3.322473000	2.261164000
6	-4.177175000	-1.350085000	2.637462000
6	-4.359546000	-2.589535000	2.012312000
8	-3.447979000	-3.136574000	1.159157000
6	4.416192000	4.324967000	-0.857548000
6	2.950106000	-1.337662000	-4.829024000
6	-4.845750000	-0.535913000	-3.228292000
6	-4.336199000	5.158515000	1.274439000
7	1.413955000	2.378794000	-0.457491000
6	5.467862000	5.125214000	-0.085643000
6	1.997864000	-2.228358000	-5.626569000
7	1.747133000	-0.155124000	-1.933000000
8	-5.504260000	0.321025000	-2.664231000
8	-4.432858000	5.720472000	0.200693000
7	-1.402364000	1.748303000	-0.072683000
8	7.382895000	6.506495000	-0.288532000
8	2.749508000	-1.136509000	-7.670778000

8	6.302424000	5.999385000	-2.198221000
8	1.240403000	-2.803269000	-7.796118000
6	-0.246224000	3.799925000	0.630900000
6	3.495329000	1.404741000	-1.272257000
6	0.556820000	-2.129310000	-2.741774000
6	-3.132362000	0.025774000	-0.422506000
28	0.167040000	0.798094000	-0.999270000
6	1.031584000	3.501516000	0.243857000
6	3.033057000	0.246150000	-1.887083000
6	-0.732958000	-1.864917000	-2.216033000
6	-2.687092000	1.243757000	0.092417000
6	2.231428000	4.366041000	0.667997000
6	3.994593000	-0.774350000	-2.515481000
6	-1.884074000	-2.724052000	-2.385825000
6	-3.501144000	2.184472000	0.811601000
6	3.434432000	3.564726000	0.059104000
6	2.993590000	-1.664141000	-3.317697000
6	-2.933498000	-2.115593000	-1.733806000
6	-2.694382000	3.281409000	1.073882000
6	2.749778000	2.385507000	-0.623808000
6	1.671185000	-1.340252000	-2.635361000
6	-2.406275000	-0.893276000	-1.170423000
6	-1.399273000	2.981969000	0.514834000
6	2.279589000	4.353920000	2.231096000
6	-1.912130000	-4.053156000	-3.097437000
6	-4.968933000	2.028925000	1.088273000
7	5.312444000	-3.858416000	-1.904432000
6	3.594451000	4.836623000	2.820762000
6	5.708235000	-2.571270000	-1.766518000
6	-1.369788000	-5.112666000	-2.145386000
6	-5.818426000	2.546381000	-0.119440000
6	-7.221249000	2.864735000	0.313644000
8	3.984191000	6.004295000	2.727955000
8	6.863885000	-2.196203000	-1.995075000
8	-2.043225000	-5.637680000	-1.273136000
6	4.620452000	-1.589749000	-1.335351000
6	-4.379108000	-2.516243000	-1.771002000
7	4.333879000	3.876995000	3.440350000
8	-0.069274000	-5.334340000	-2.334388000
8	-7.598284000	3.931602000	0.785865000
6	2.074529000	5.807328000	0.150932000
6	5.102947000	-0.105274000	-3.344511000
6	-5.078981000	-2.022637000	-3.075721000
6	-3.055285000	4.550327000	1.820182000
6	6.394255000	5.898118000	-0.990667000
6	2.065774000	-1.971915000	-7.112581000

7	-1.060887000	-0.763741000	-1.487442000
8	-3.774137000	-0.265252000	-3.990530000
8	-5.389066000	4.944226000	2.090713000
6	3.687810000	-2.189717000	8.822549000
6	2.575377000	-1.674375000	7.899089000
6	2.549935000	-2.300797000	6.496071000
6	1.220371000	-2.029485000	5.768901000
6	1.236402000	-2.521635000	4.319450000
16	-0.489189000	-2.594643000	3.628237000
6	0.285844000	-3.350558000	0.676082000
8	1.241890000	-6.186941000	-0.167616000
16	-0.338626000	-1.914980000	1.646886000
6	1.550424000	-3.963788000	1.256538000
8	3.478967000	-5.718006000	0.847821000
16	2.311396000	-5.161239000	0.123853000
8	2.661138000	-4.373362000	-1.101821000
6	5.573411000	-0.917260000	3.933624000
6	4.268215000	-0.284373000	3.510387000
6	3.767839000	-0.453782000	2.210275000
6	3.491552000	0.458402000	4.410880000
6	2.533281000	0.063554000	1.820296000
6	2.249220000	0.981185000	4.041884000
6	1.751179000	0.765260000	2.749743000
8	0.511081000	1.249133000	2.474787000
6	-4.082911000	4.435415000	-5.509526000
6	-2.819972000	3.754463000	-4.976315000
6	-3.018845000	3.219186000	-3.557275000
7	-2.287304000	2.087915000	-3.237986000
8	-3.799868000	3.727855000	-2.765005000
1	-7.306629000	-1.888979000	5.713080000
1	-6.924873000	-0.210789000	5.336298000
1	-7.367683000	-3.431838000	3.357633000
1	-4.975681000	0.073478000	4.026046000
1	-5.664541000	-4.274983000	1.757290000
1	-3.288209000	-0.762585000	2.426928000
1	-2.670168000	-2.550281000	1.063592000
1	5.718117000	-1.882527000	3.435257000
1	5.591032000	-1.097539000	5.014723000
1	4.344297000	-1.025426000	1.488304000
1	3.845094000	0.607851000	5.428915000
1	2.161085000	-0.088411000	0.812826000
1	1.639078000	1.529827000	4.753310000
1	0.272421000	1.097108000	1.535855000
1	4.940554000	3.605746000	-1.494190000
1	3.877151000	4.983162000	-1.544068000
1	3.954059000	-1.449013000	-5.250531000

1	2.665543000	-0.288664000	-4.975727000
1	5.017901000	5.834016000	0.617845000
1	6.080415000	4.460835000	0.538079000
1	2.209212000	-3.292733000	-5.457549000
1	0.957563000	-2.082379000	-5.315122000
1	-0.374178000	4.727569000	1.178712000
1	4.570153000	1.547978000	-1.273480000
1	0.683750000	-3.082792000	-3.238780000
1	4.031476000	3.138168000	0.877183000
1	3.233888000	-2.725948000	-3.202887000
1	2.054943000	3.343996000	2.578127000
1	1.490051000	5.009431000	2.613544000
1	-2.938223000	-4.323338000	-3.361013000
1	-1.311095000	-4.031656000	-4.011904000
1	-5.235856000	2.589376000	1.987551000
1	-5.225333000	0.982690000	1.281502000
1	-5.821840000	1.795842000	-0.911878000
1	-5.375119000	3.459383000	-0.520318000
1	5.097552000	-0.881034000	-0.651931000
1	3.816897000	-2.108078000	-0.802024000
1	-4.917006000	-2.113278000	-0.908389000
1	-4.476212000	-3.605158000	-1.712994000
1	1.977158000	5.829866000	-0.938820000
1	2.915508000	6.436029000	0.451635000
1	1.166812000	6.254559000	0.569372000
1	5.691817000	-0.846889000	-3.889828000
1	4.687287000	0.612525000	-4.057601000
1	5.801382000	0.427355000	-2.692688000
1	-4.664987000	-2.551989000	-3.938501000
1	-6.154851000	-2.212000000	-3.014340000
1	-2.265117000	5.294906000	1.694835000
1	-3.174291000	4.361060000	2.893281000
1	3.670668000	-3.286299000	8.868345000
1	3.533253000	-1.821012000	9.844669000
1	1.611399000	-1.881442000	8.386222000
1	2.635844000	-0.580856000	7.806205000
1	3.386024000	-1.918698000	5.896675000
1	2.694401000	-3.387970000	6.575433000
1	0.413828000	-2.525020000	6.328482000
1	0.996514000	-0.955924000	5.775858000
1	1.663340000	-3.527477000	4.257179000
1	1.826488000	-1.856362000	3.686784000
1	-2.216631000	-5.375584000	1.654929000
1	0.492246000	-2.911151000	-0.303451000
1	-0.500416000	-4.097998000	0.563163000
1	2.314922000	-3.205976000	1.452047000

1	1.343037000	-4.508707000	2.180480000
1	-4.876854000	3.699402000	-5.679288000
1	-4.453158000	5.155067000	-4.773693000
1	-2.490918000	2.946740000	-5.640726000
1	-1.989746000	4.473440000	-4.926544000
1	6.455020000	-0.304874000	3.698274000
1	-8.300841000	-0.997692000	4.576149000
1	-3.903547000	4.967625000	-6.449863000
6	-1.653054000	-6.245922000	1.987642000
1	-2.249844000	-7.144204000	1.818663000
1	-1.425775000	-6.152288000	3.050796000
1	-0.730552000	-6.315904000	1.412882000
1	7.928863000	6.991480000	-0.936547000
1	1.330291000	-2.574115000	-8.740811000
1	-3.454788000	0.653391000	-3.794836000
1	0.341571000	-5.784465000	-1.534853000
1	-6.220666000	5.010373000	1.562361000
1	5.213813000	4.139421000	3.863994000
1	3.998050000	2.930573000	3.571613000
1	-1.454174000	1.888460000	-3.782497000
1	-2.154044000	1.899729000	-2.242436000
1	4.704166000	-1.892323000	8.538037000
1	6.017125000	-4.541791000	-2.147944000
8	-8.066444000	1.824704000	0.180718000
1	-8.933107000	2.109286000	0.530741000
1	-4.175040000	-0.212614000	-0.246472000
1	4.386012000	-4.175353000	-1.602526000

C
RC
C-RC-opt.gjf.log

Temperature 298.150 Kelvin. Pressure 1.00000 Atm.

Zero-point correction= 1.543737 (Hartree/Particle)
Thermal correction to Energy= 1.645462
Thermal correction to Enthalpy= 1.646406
Thermal correction to Gibbs Free Energy= 1.389885
Sum of electronic and zero-point Energies= -5861.158006
Sum of electronic and thermal Energies= -5861.056281
Sum of electronic and thermal Enthalpies= -5861.055337
Sum of electronic and thermal Free Energies= -5861.311858

6	-1.376469000	7.552948000	-0.888252000
6	-0.944860000	6.200479000	-1.405283000

6	-0.039022000	6.089812000	-2.471697000
6	-1.407538000	5.014081000	-0.824492000
6	0.392146000	4.849355000	-2.938405000
6	-0.977059000	3.762280000	-1.268692000
6	-0.073330000	3.674315000	-2.334421000
8	0.378087000	2.484576000	-2.827670000
6	0.215549000	-2.145200000	4.286952000
6	3.733171000	-4.525407000	-0.508002000
6	7.732282000	0.360822000	-1.672468000
6	0.759046000	5.857093000	3.570410000
7	-0.735330000	-0.603391000	1.673713000
6	0.225835000	-2.474166000	5.786076000
6	4.741608000	-4.253080000	-1.640382000
7	1.128855000	-2.161313000	-0.041255000
8	7.669865000	-0.471797000	-0.775290000
8	1.764807000	6.358696000	4.040407000
7	1.018093000	1.765250000	1.008608000
8	1.937298000	-2.741763000	7.420357000
8	5.808421000	-2.366391000	-2.710673000
8	2.169061000	-3.910697000	5.511680000
8	5.731710000	-2.410963000	-0.460951000
6	-1.082992000	1.717089000	2.313573000
6	-0.382223000	-3.128159000	1.628630000
6	2.599071000	-1.936698000	-1.968498000
6	2.730210000	2.619535000	-0.497181000
28	0.843003000	-0.149173000	0.307147000
6	-1.138084000	0.242260000	2.557272000
6	0.605196000	-3.219844000	0.728160000
6	2.733609000	-0.575244000	-2.028155000
6	1.823145000	2.717444000	0.589594000
6	-1.676125000	-0.366846000	3.856324000
6	1.282215000	-4.558579000	0.389954000
6	3.548788000	0.127572000	-3.094968000
6	1.757692000	3.994131000	1.425662000
6	-1.171183000	-1.836550000	3.692645000
6	2.254652000	-4.146217000	-0.767117000
6	4.019803000	1.371523000	-2.310930000
6	0.469542000	3.749073000	2.242889000
6	-1.140484000	-1.953508000	2.136913000
6	1.982333000	-2.664777000	-0.925603000
6	2.922099000	1.498128000	-1.260097000
6	0.361660000	2.215739000	2.247236000
6	-3.238180000	-0.280982000	3.794855000
6	2.684908000	0.480501000	-4.313894000
6	1.742012000	5.308526000	0.599858000
7	0.374175000	-7.042570000	-2.061212000

6	-3.922552000	-1.101505000	4.879045000
6	0.875163000	-6.783707000	-0.831642000
6	3.500485000	1.092540000	-5.428258000
6	2.828525000	6.341875000	0.982864000
8	-3.928092000	-0.773119000	6.063897000
8	1.766766000	-7.467117000	-0.312615000
8	4.699889000	1.293714000	-5.410192000
6	0.237931000	-5.582666000	-0.130786000
6	5.348384000	1.120881000	-1.564677000
7	-4.544668000	-2.236673000	4.442786000
8	2.727818000	1.407589000	-6.492818000
6	-1.161836000	0.383033000	5.096772000
6	1.965659000	-5.096724000	1.666182000
6	6.560603000	0.866745000	-2.471135000
6	0.423391000	4.385076000	3.645862000
6	1.532627000	-3.124737000	6.187044000
6	5.440910000	-2.907813000	-1.681350000
7	2.267941000	0.310070000	-1.097943000
8	8.903894000	0.900024000	-2.041568000
8	-0.131308000	6.552462000	2.834577000
6	-10.998900000	1.259630000	-2.938284000
6	-10.745523000	2.080623000	-1.664193000
6	-10.206863000	1.249245000	-0.486282000
6	-8.939429000	0.414226000	-0.730769000
6	-7.608857000	1.169325000	-0.827853000
16	-7.351137000	1.993227000	-2.473551000
6	-0.878445000	-1.203127000	-2.766825000
8	-1.456965000	-3.405501000	-4.904438000
16	-1.613038000	0.176500000	-1.790275000
6	-1.450023000	-2.567475000	-2.408726000
8	-1.496411000	-5.108315000	-3.071170000
16	-0.891981000	-3.833633000	-3.594905000
8	0.597416000	-3.834755000	-3.544630000
6	-8.385321000	-3.230567000	-1.489341000
6	-7.123822000	-2.621352000	-0.936432000
6	-6.103841000	-2.214508000	-1.804726000
6	-6.958869000	-2.354353000	0.430442000
6	-4.964144000	-1.559554000	-1.339818000
6	-5.826857000	-1.698037000	0.913405000
6	-4.817997000	-1.296065000	0.026822000
8	-3.731602000	-0.649761000	0.542694000
6	4.419189000	-1.386384000	5.123762000
6	4.211402000	-1.868729000	3.687794000
6	3.533684000	-0.806043000	2.830844000
7	3.846550000	-0.813952000	1.509261000
8	2.757901000	0.020005000	3.325277000

1	-1.972624000	8.099918000	-1.630542000
1	-1.982306000	7.454835000	0.018662000
1	0.346492000	6.993411000	-2.939526000
1	-2.101888000	5.064020000	0.011337000
1	1.097941000	4.771450000	-3.760264000
1	-1.320037000	2.857787000	-0.778387000
1	0.067669000	1.747502000	-2.258786000
1	-8.232680000	-3.571075000	-2.519437000
1	-9.190742000	-2.484126000	-1.520381000
1	-6.200421000	-2.403330000	-2.871019000
1	-7.735454000	-2.652506000	1.131297000
1	-4.194170000	-1.248370000	-2.034116000
1	-5.726390000	-1.477410000	1.971609000
1	-3.050544000	-0.497720000	-0.152753000
1	0.610179000	-3.024293000	3.771056000
1	0.922344000	-1.336883000	4.071057000
1	3.735020000	-5.604395000	-0.328546000
1	4.097257000	-4.052552000	0.408140000
1	0.050644000	-1.606929000	6.423975000
1	-0.568000000	-3.200482000	6.013925000
1	5.566573000	-4.972888000	-1.534541000
1	4.308429000	-4.430396000	-2.628450000
1	-1.572786000	1.944060000	1.359781000
1	-1.623673000	2.252119000	3.099850000
1	-0.707166000	-4.059695000	2.088119000
1	3.040119000	-2.512120000	-2.770209000
1	4.372799000	-0.507351000	-3.423435000
1	2.626634000	3.991728000	2.102584000
1	-1.890914000	-2.553233000	4.103433000
1	1.950405000	-4.625105000	-1.704964000
1	4.103097000	2.263969000	-2.938177000
1	-0.369818000	4.145247000	1.659777000
1	-2.178362000	-2.066017000	1.785381000
1	0.929621000	1.793053000	3.088814000
1	-3.583407000	-0.576215000	2.802580000
1	-3.533961000	0.763124000	3.943188000
1	2.186427000	-0.413533000	-4.709191000
1	1.888369000	1.183820000	-4.043149000
1	0.765552000	5.773439000	0.722674000
1	1.821559000	5.089049000	-0.467376000
1	3.012144000	6.312633000	2.061010000
1	-0.350809000	-5.964876000	0.712748000
1	-0.457961000	-5.090803000	-0.814422000
1	5.209755000	0.256061000	-0.907868000
1	5.553467000	1.975930000	-0.910192000
1	-0.069095000	0.408041000	5.128666000

1	-1.541977000	-0.085857000	6.006387000
1	-1.519981000	1.417845000	5.099670000
1	2.478398000	-6.039356000	1.471178000
1	2.672119000	-4.369693000	2.079334000
1	1.207828000	-5.280905000	2.434929000
1	6.332908000	0.074797000	-3.192879000
1	6.845083000	1.758017000	-3.035301000
1	1.150473000	3.912716000	4.311974000
1	-0.577791000	4.255150000	4.073926000
1	-10.066077000	1.081910000	-3.485511000
1	-11.687473000	1.773648000	-3.617781000
1	-10.060384000	2.907256000	-1.892485000
1	-11.684109000	2.547673000	-1.336735000
1	-10.033669000	1.911121000	0.374363000
1	-11.000433000	0.552260000	-0.180337000
1	-8.825102000	-0.274321000	0.115906000
1	-9.061432000	-0.221094000	-1.615585000
1	-6.789154000	0.460049000	-0.703835000
1	-7.537838000	1.925903000	-0.039791000
1	-6.003636000	2.054845000	-2.411095000
1	0.193520000	-1.186318000	-2.573492000
1	-1.049682000	-0.976978000	-3.822897000
1	-1.133387000	-2.871728000	-1.408332000
1	-2.540823000	-2.588723000	-2.455035000
1	4.964516000	-0.435568000	5.136943000
1	3.464067000	-1.222299000	5.626926000
1	5.161650000	-2.168887000	3.229958000
1	3.563704000	-2.755762000	3.687751000
1	-8.786901000	-4.083968000	-0.927194000
1	-0.511552000	8.180578000	-0.637425000
1	5.004961000	-2.109358000	5.701635000
6	-2.691996000	0.955014000	-3.049272000
1	-3.223960000	1.766227000	-2.552733000
1	-2.078789000	1.368237000	-3.848815000
1	-3.412051000	0.248190000	-3.459667000
1	2.774023000	-3.212184000	7.602946000
1	6.343684000	-1.636519000	-0.583605000
1	9.602335000	0.485879000	-1.496895000
1	3.317866000	1.788863000	-7.171142000
1	0.316075000	7.374668000	2.516072000
1	-4.923532000	-2.871441000	5.133632000
1	-4.430788000	-2.582988000	3.500927000
1	4.359826000	-1.582282000	1.092484000
1	3.229448000	-0.302439000	0.881720000
1	0.801166000	-7.789010000	-2.594002000
1	-11.414899000	0.271567000	-2.704589000

1	3.341853000	3.487139000	-0.721356000
1	3.771603000	6.147078000	0.466287000
6	2.345493000	7.738763000	0.681775000
8	1.381503000	8.274683000	1.216198000
8	3.063488000	8.368099000	-0.262148000
1	2.668109000	9.253092000	-0.390286000
1	-0.265492000	-6.392352000	-2.529870000

TS_1
C-TS_1-opt.gjf.log

Temperature 298.150 Kelvin. Pressure 1.00000 Atm.

Zero-point correction= 1.538134 (Hartree/Particle)
Thermal correction to Energy= 1.641165
Thermal correction to Enthalpy= 1.642109
Thermal correction to Gibbs Free Energy= 1.380578
Sum of electronic and zero-point Energies= -5861.104596
Sum of electronic and thermal Energies= -5861.001565
Sum of electronic and thermal Enthalpies= -5861.000621
Sum of electronic and thermal Free Energies= -5861.262152

6	-0.722459000	7.655000000	1.060667000
6	-0.453043000	6.408748000	0.253849000
6	0.379361000	6.435091000	-0.875545000
6	-1.038430000	5.184226000	0.598348000
6	0.611322000	5.290738000	-1.637426000
6	-0.810049000	4.025250000	-0.143801000
6	0.010858000	4.074345000	-1.280714000
8	0.243507000	2.988930000	-2.062629000
6	0.437149000	-3.393080000	3.742520000
6	3.251112000	-4.534975000	-1.232232000
6	7.636310000	1.449723000	-2.843890000
6	1.629455000	4.557419000	4.860124000
7	-0.511089000	-1.183413000	1.705523000
6	0.505345000	-4.109605000	5.098416000
6	4.490836000	-4.046837000	-2.012307000
7	0.937061000	-2.313308000	-0.691954000
8	8.023751000	0.761295000	-1.920285000
8	2.736143000	4.847346000	5.278769000
7	1.238571000	1.268826000	1.360644000
8	2.325942000	-4.711961000	6.512801000
8	6.487027000	-5.321354000	-1.444767000
8	2.420900000	-5.421711000	4.379725000
8	5.989901000	-3.460345000	-0.274254000
6	-0.664820000	0.922168000	2.917029000

6	-0.386766000	-3.609015000	0.929146000
6	2.368089000	-1.697844000	-2.577870000
6	2.810870000	2.402473000	-0.114017000
28	0.724502000	-0.379706000	0.130522000
6	-0.796255000	-0.567399000	2.794608000
6	0.373139000	-3.495891000	-0.167738000
6	2.578090000	-0.366892000	-2.324894000
6	2.053280000	2.255565000	1.079639000
6	-1.351620000	-1.465244000	3.904192000
6	0.658325000	-4.663436000	-1.118527000
6	3.238748000	0.583060000	-3.304664000
6	2.179243000	3.276033000	2.209004000
6	-0.953655000	-2.870454000	3.339106000
6	1.904598000	-4.118683000	-1.876605000
6	3.775921000	1.677309000	-2.352141000
6	0.983348000	2.879969000	3.106888000
6	-0.993785000	-2.576258000	1.814510000
6	1.713254000	-2.617784000	-1.720681000
6	2.881286000	1.491117000	-1.133315000
6	0.780187000	1.396752000	2.753329000
6	-2.905492000	-1.257551000	3.901559000
6	2.197953000	1.131598000	-4.297352000
6	2.158902000	4.755565000	1.745162000
7	-2.388172000	-6.062797000	-1.291017000
6	-3.643786000	-2.371092000	4.631196000
6	-1.894876000	-4.822350000	-1.588960000
6	2.841283000	1.938902000	-5.397000000
6	3.368720000	5.609795000	2.194576000
8	-3.562391000	-2.543567000	5.846512000
8	-2.542646000	-3.803636000	-1.348792000
8	4.035701000	2.030104000	-5.617837000
6	-0.494854000	-4.779426000	-2.177280000
6	5.257978000	1.468846000	-1.980567000
7	-4.397732000	-3.178394000	3.833033000
8	1.917083000	2.568539000	-6.153424000
6	-0.778613000	-1.116243000	5.285751000
6	0.855280000	-6.007501000	-0.407513000
6	6.206391000	1.847654000	-3.118932000
6	1.156081000	3.142174000	4.616458000
6	1.834089000	-4.817007000	5.257318000
6	5.754575000	-4.374282000	-1.260395000
7	2.250738000	0.279411000	-1.166778000
8	8.479060000	1.947884000	-3.780087000
8	0.727338000	5.478224000	4.469404000
6	-10.815103000	2.660453000	-2.036964000
6	-10.386851000	3.105117000	-0.630325000

6	-9.799924000	1.975902000	0.233902000
6	-8.636024000	1.161487000	-0.353653000
6	-7.253128000	1.821857000	-0.397446000
16	-7.064159000	3.065500000	-1.765104000
6	-0.818848000	-0.118827000	-2.834591000
8	-0.460971000	-0.605904000	-5.738198000
16	-1.231374000	0.409356000	-1.113180000
6	-1.747859000	-1.135797000	-3.477965000
8	-2.071311000	-2.527148000	-5.701199000
16	-0.976791000	-1.799834000	-4.996579000
8	0.126823000	-2.698341000	-4.521820000
6	-8.466619000	-2.193924000	-1.906398000
6	-7.147926000	-1.883340000	-1.240430000
6	-6.027374000	-1.509147000	-1.992664000
6	-7.011925000	-1.909565000	0.155660000
6	-4.818907000	-1.166324000	-1.386805000
6	-5.817500000	-1.550558000	0.780218000
6	-4.709144000	-1.177014000	0.007627000
8	-3.559176000	-0.833917000	0.656511000
6	4.647406000	-2.844320000	4.313259000
6	4.281845000	-3.024459000	2.835811000
6	3.606046000	-1.794278000	2.243405000
7	3.949262000	-1.487636000	0.968335000
8	2.788160000	-1.133006000	2.893008000
1	-1.273637000	8.400450000	0.471497000
1	-1.328363000	7.426022000	1.944139000
1	0.858611000	7.368795000	-1.163401000
1	-1.680374000	5.127956000	1.475018000
1	1.253867000	5.318684000	-2.512910000
1	-1.262206000	3.082616000	0.145777000
1	-0.180587000	2.188553000	-1.650397000
1	-8.354120000	-2.262685000	-2.993519000
1	-9.207208000	-1.410004000	-1.703617000
1	-6.093805000	-1.484371000	-3.077873000
1	-7.862759000	-2.197470000	0.769890000
1	-3.959533000	-0.893876000	-1.984404000
1	-5.740640000	-1.540812000	1.863790000
1	-2.847617000	-0.582182000	0.010746000
1	0.748969000	-4.121580000	2.987410000
1	1.178320000	-2.588351000	3.697697000
1	3.298486000	-5.626425000	-1.170110000
1	3.287322000	-4.150829000	-0.205494000
1	0.351096000	-3.444565000	5.948742000
1	-0.275354000	-4.881904000	5.151004000
1	4.537979000	-4.534433000	-2.989678000
1	4.442587000	-2.965195000	-2.161091000

1	-1.278806000	1.392733000	2.138798000
1	-1.048756000	1.253896000	3.885516000
1	-0.771914000	-4.602252000	1.146172000
1	2.708628000	-2.088510000	-3.528686000
1	4.034598000	0.085431000	-3.864815000
1	3.120822000	3.062251000	2.738658000
1	-1.702667000	-3.628209000	3.592378000
1	1.899726000	-4.412139000	-2.931615000
1	3.645437000	2.682902000	-2.767305000
1	0.107848000	3.438634000	2.753547000
1	-2.048907000	-2.525173000	1.500433000
1	1.434337000	0.764374000	3.367934000
1	-3.268207000	-1.155644000	2.876165000
1	-3.130452000	-0.319123000	4.419514000
1	1.608728000	0.333014000	-4.768883000
1	1.472455000	1.768973000	-3.779109000
1	1.250245000	5.215369000	2.128985000
1	2.068331000	4.814683000	0.658980000
1	3.693744000	5.309066000	3.194471000
1	-0.455116000	-3.921069000	-2.852616000
1	-0.298517000	-5.674067000	-2.781898000
1	5.416377000	0.420096000	-1.700037000
1	5.499105000	2.064485000	-1.092913000
1	0.313230000	-1.178656000	5.298874000
1	-1.193330000	-1.788928000	6.039327000
1	-1.058359000	-0.097861000	5.575390000
1	1.200894000	-6.769867000	-1.115513000
1	1.578741000	-5.935373000	0.409626000
1	-0.089180000	-6.361132000	0.016326000
1	5.901799000	1.386360000	-4.065900000
1	6.178298000	2.927073000	-3.311818000
1	1.902963000	2.467189000	5.043134000
1	0.201444000	2.976360000	5.129328000
1	-9.947593000	2.587519000	-2.702848000
1	-11.513660000	3.375798000	-2.485483000
1	-9.668075000	3.930473000	-0.717512000
1	-11.253698000	3.515272000	-0.094518000
1	-9.490120000	2.387601000	1.205319000
1	-10.612545000	1.266761000	0.449014000
1	-8.512478000	0.268849000	0.270489000
1	-8.892083000	0.793899000	-1.353906000
1	-6.499853000	1.049304000	-0.570813000
1	-7.031639000	2.310912000	0.556873000
1	-5.723951000	2.920512000	-1.921425000
1	0.178078000	-0.547676000	-2.782425000
1	-0.764326000	0.772510000	-3.462663000

1	-1.951997000	-1.987423000	-2.822830000
1	-2.696427000	-0.685979000	-3.786020000
1	5.284521000	-1.964225000	4.457572000
1	3.754286000	-2.719225000	4.929980000
1	5.165344000	-3.293622000	2.247154000
1	3.572936000	-3.857676000	2.740549000
1	-8.906684000	-3.137920000	-1.558831000
1	0.191307000	8.146391000	1.419130000
1	5.202107000	-3.723290000	4.658876000
6	-3.339390000	1.923808000	-1.847869000
1	-3.927849000	1.640725000	-0.988567000
1	-2.827747000	2.874122000	-1.840321000
1	-3.551399000	1.450250000	-2.794957000
1	3.173113000	-5.198682000	6.528159000
1	6.798382000	-3.734005000	0.200018000
1	9.374916000	1.634413000	-3.550644000
1	2.397861000	3.043217000	-6.858412000
1	1.208381000	6.327208000	4.307572000
1	-4.866355000	-3.973229000	4.247158000
1	-4.413983000	-3.086337000	2.827232000
1	4.598045000	-2.063443000	0.445902000
1	3.420773000	-0.781566000	0.464705000
1	-3.359706000	-6.126257000	-1.014919000
1	-11.289090000	1.670819000	-2.040871000
1	3.400638000	3.306235000	-0.219768000
1	4.212082000	5.507021000	1.507241000
6	2.951732000	7.055096000	2.303578000
8	2.151968000	7.488663000	3.124444000
8	3.517078000	7.852537000	1.383470000
1	3.169838000	8.755802000	1.522814000
1	-1.956666000	-6.897810000	-1.660077000

Int_1
$C\text{-}Int_1$-opt.gjf.log

Temperature 298.150 Kelvin. Pressure 1.00000 Atm.

Zero-point correction= 1.537820 (Hartree/Particle)
Thermal correction to Energy= 1.641648
Thermal correction to Enthalpy= 1.642592
Thermal correction to Gibbs Free Energy= 1.378181
Sum of electronic and zero-point Energies= -5861.105963
Sum of electronic and thermal Energies= -5861.002135
Sum of electronic and thermal Enthalpies= -5861.001191
Sum of electronic and thermal Free Energies= -5861.265602

6	-0.872927000	7.636327000	0.941066000
6	-0.579230000	6.382322000	0.153342000
6	0.236864000	6.410712000	-0.988013000
6	-1.122698000	5.147118000	0.528193000
6	0.491776000	5.259618000	-1.732475000
6	-0.870172000	3.981285000	-0.196169000
6	-0.068253000	4.032132000	-1.347117000
8	0.183394000	2.940845000	-2.112876000
6	0.417689000	-3.280850000	3.853710000
6	3.354251000	-4.551077000	-1.087486000
6	7.653730000	1.526195000	-2.853692000
6	1.621332000	4.701285000	4.767174000
7	-0.470577000	-1.124363000	1.755065000
6	0.464040000	-3.969372000	5.225798000
6	4.588439000	-4.080516000	-1.886960000
7	1.024271000	-2.318549000	-0.593222000
8	8.056339000	0.872972000	-1.911180000
8	2.727513000	5.020630000	5.165179000
7	1.237100000	1.340209000	1.339233000
8	2.314906000	-4.436084000	6.655245000
8	6.567061000	-5.374791000	-1.303781000
8	2.387801000	-5.304555000	4.580557000
8	6.121984000	-3.466867000	-0.188689000
6	-0.649282000	1.005104000	2.923867000
6	-0.330702000	-3.569052000	1.034055000
6	2.460649000	-1.741044000	-2.486492000
6	2.792713000	2.455442000	-0.167167000
28	0.754833000	-0.350125000	0.163183000
6	-0.783980000	-0.486117000	2.823156000
6	0.455282000	-3.487012000	-0.047070000
6	2.640398000	-0.397639000	-2.278607000
6	2.038530000	2.329200000	1.031265000
6	-1.383915000	-1.361094000	3.928189000
6	0.760274000	-4.681380000	-0.957879000
6	3.279768000	0.535741000	-3.288459000
6	2.158012000	3.376199000	2.136148000
6	-0.966026000	-2.775887000	3.405469000
6	2.003797000	-4.147966000	-1.730812000
6	3.789148000	1.673609000	-2.372326000
6	0.980843000	2.979029000	3.057911000
6	-0.962137000	-2.513473000	1.874801000
6	1.809219000	-2.644174000	-1.608061000
6	2.892949000	1.509373000	-1.151571000
6	0.790115000	1.486952000	2.733316000
6	-2.936583000	-1.151698000	3.858522000
6	2.227455000	1.031801000	-4.296735000

6	2.104202000	4.844131000	1.638374000
7	-2.261929000	-6.125897000	-1.081467000
6	-3.703742000	-2.242091000	4.593591000
6	-1.791044000	-4.890908000	-1.434454000
6	2.854579000	1.818689000	-5.420241000
6	3.302026000	5.731334000	2.054928000
8	-3.687710000	-2.361716000	5.817721000
8	-2.461316000	-3.875765000	-1.246950000
8	4.046843000	1.923055000	-5.647215000
6	-0.387628000	-4.848096000	-2.015619000
6	5.274832000	1.513795000	-1.991396000
7	-4.404765000	-3.091838000	3.790945000
8	1.918316000	2.411412000	-6.191302000
6	-0.867609000	-0.989087000	5.325776000
6	0.971547000	-5.997009000	-0.198770000
6	6.215125000	1.882929000	-3.139757000
6	1.171495000	3.272533000	4.559303000
6	1.804297000	-4.645561000	5.420418000
6	5.856427000	-4.407360000	-1.141867000
7	2.295069000	0.280437000	-1.143510000
8	8.485205000	2.017533000	-3.803371000
8	0.696997000	5.595951000	4.368307000
6	-10.864457000	2.472435000	-2.206288000
6	-10.513276000	2.931055000	-0.782745000
6	-9.946811000	1.810910000	0.108324000
6	-8.741706000	1.020234000	-0.427463000
6	-7.374111000	1.710263000	-0.398427000
16	-7.165960000	3.013956000	-1.705039000
6	-0.739299000	-0.244922000	-2.834198000
8	-0.326280000	-0.813579000	-5.722077000
16	-1.188067000	0.319699000	-1.132344000
6	-1.658457000	-1.275525000	-3.471801000
8	-1.922080000	-2.745732000	-5.654051000
16	-0.850175000	-1.984757000	-4.949953000
8	0.250764000	-2.856214000	-4.420304000
6	-8.442673000	-2.344525000	-2.035229000
6	-7.138807000	-1.995702000	-1.356905000
6	-6.025838000	-1.577466000	-2.097877000
6	-7.011457000	-2.026451000	0.039902000
6	-4.831696000	-1.201461000	-1.481586000
6	-5.831640000	-1.638872000	0.674766000
6	-4.728305000	-1.228334000	-0.086391000
8	-3.592558000	-0.860483000	0.574996000
6	4.633487000	-2.763660000	4.283249000
6	4.237512000	-2.999640000	2.819477000
6	3.599698000	-1.778185000	2.171858000

7	4.043072000	-1.468187000	0.929263000
8	2.726639000	-1.120883000	2.750875000
1	-1.425657000	8.367193000	0.335513000
1	-1.486749000	7.411316000	1.820112000
1	0.683994000	7.352431000	-1.300587000
1	-1.750530000	5.087821000	1.414926000
1	1.120171000	5.291151000	-2.618092000
1	-1.289576000	3.030887000	0.116213000
1	-0.236528000	2.137706000	-1.696052000
1	-8.314592000	-2.420710000	-3.120234000
1	-9.201577000	-1.573180000	-1.850810000
1	-6.086759000	-1.543821000	-3.183201000
1	-7.857062000	-2.344337000	0.646499000
1	-3.978797000	-0.891130000	-2.072188000
1	-5.761630000	-1.638109000	1.758677000
1	-2.870197000	-0.610419000	-0.062296000
1	0.749670000	-4.024231000	3.122134000
1	1.155812000	-2.473809000	3.801888000
1	3.401852000	-5.641001000	-1.003683000
1	3.398497000	-4.146090000	-0.069362000
1	0.278893000	-3.290646000	6.058716000
1	-0.302515000	-4.755571000	5.271985000
1	4.622273000	-4.582933000	-2.857403000
1	4.545004000	-3.001134000	-2.051624000
1	-1.278027000	1.464402000	2.150388000
1	-1.019521000	1.349082000	3.893256000
1	-0.720021000	-4.556212000	1.270481000
1	2.809879000	-2.154807000	-3.424280000
1	4.087206000	0.039581000	-3.833046000
1	3.110233000	3.192462000	2.657884000
1	-1.717712000	-3.532287000	3.654531000
1	1.993476000	-4.461131000	-2.780074000
1	3.637098000	2.661078000	-2.822143000
1	0.092769000	3.519196000	2.707642000
1	-2.006807000	-2.466323000	1.526186000
1	1.455708000	0.870720000	3.353122000
1	-3.261559000	-1.080905000	2.817655000
1	-3.178853000	-0.198118000	4.339437000
1	1.653988000	0.208704000	-4.745118000
1	1.489201000	1.669413000	-3.796710000
1	1.190817000	5.295174000	2.021511000
1	2.001135000	4.876042000	0.552077000
1	3.644867000	5.458321000	3.056830000
1	-0.354973000	-4.012496000	-2.718983000
1	-0.177005000	-5.759645000	-2.589562000
1	5.460267000	0.478223000	-1.680556000

1	5.498826000	2.141369000	-1.121441000
1	0.223329000	-1.043510000	5.382301000
1	-1.305718000	-1.654948000	6.072191000
1	-1.165827000	0.030496000	5.591446000
1	1.321838000	-6.782126000	-0.879065000
1	1.696575000	-5.888661000	0.613010000
1	0.031672000	-6.342998000	0.241078000
1	5.921684000	1.388530000	-4.073489000
1	6.161964000	2.955626000	-3.362543000
1	1.937516000	2.620186000	4.987320000
1	0.228056000	3.099337000	5.090107000
1	-9.965541000	2.404544000	-2.828994000
1	-11.553595000	3.170685000	-2.694288000
1	-9.809014000	3.771192000	-0.834810000
1	-11.414222000	3.321870000	-0.290370000
1	-9.689398000	2.226242000	1.093397000
1	-10.756095000	1.087402000	0.284750000
1	-8.630519000	0.123426000	0.194084000
1	-8.941520000	0.657423000	-1.442275000
1	-6.597178000	0.962009000	-0.575150000
1	-7.197090000	2.163894000	0.582501000
1	-5.806586000	2.903141000	-1.798417000
1	0.256443000	-0.672693000	-2.762644000
1	-0.675892000	0.630426000	-3.483649000
1	-1.883247000	-2.107779000	-2.798613000
1	-2.597647000	-0.830357000	-3.814778000
1	5.275287000	-1.880572000	4.381580000
1	3.756152000	-2.619822000	4.918061000
1	5.104345000	-3.329646000	2.237314000
1	3.497439000	-3.809513000	2.776195000
1	-8.870734000	-3.293547000	-1.686360000
1	0.030505000	8.143411000	1.303840000
1	5.199062000	-3.632351000	4.637133000
6	-3.749060000	2.140964000	-1.670707000
1	-4.039778000	1.572839000	-0.801187000
1	-3.202132000	3.063378000	-1.546361000
1	-3.786514000	1.666706000	-2.639736000
1	3.172108000	-4.903900000	6.691645000
1	6.929108000	-3.741714000	0.287258000
1	9.387996000	1.731547000	-3.565405000
1	2.389388000	2.873045000	-6.911328000
1	1.158859000	6.450484000	4.181528000
1	-4.883113000	-3.877003000	4.212418000
1	-4.362243000	-3.045508000	2.782775000
1	4.732041000	-2.041548000	0.458544000
1	3.544597000	-0.769148000	0.386596000

1	-3.234558000	-6.195496000	-0.810677000
1	-11.323136000	1.475610000	-2.208909000
1	3.361556000	3.368758000	-0.301638000
1	4.139135000	5.630566000	1.359720000
6	2.857934000	7.170307000	2.138799000
8	2.058465000	7.604891000	2.959536000
8	3.398029000	7.959425000	1.196965000
1	3.034151000	8.858335000	1.321341000
1	-1.812265000	-6.968291000	-1.409856000

TS_2
C-TS_2-opt.gjf.log

Temperature 298.150 Kelvin. Pressure 1.00000 Atm.

Zero-point correction= 1.537180 (Hartree/Particle)
Thermal correction to Energy= 1.639608
Thermal correction to Enthalpy= 1.640552
Thermal correction to Gibbs Free Energy= 1.380416
Sum of electronic and zero-point Energies= -5861.105519
Sum of electronic and thermal Energies= -5861.003091
Sum of electronic and thermal Enthalpies= -5861.002147
Sum of electronic and thermal Free Energies= -5861.262283

6	-0.808452000	7.654081000	0.835035000
6	-0.519335000	6.390827000	0.061034000
6	0.311358000	6.399209000	-1.069961000
6	-1.084229000	5.166027000	0.438528000
6	0.561031000	5.238506000	-1.801394000
6	-0.838172000	3.991095000	-0.273193000
6	-0.019410000	4.021604000	-1.412983000
8	0.230234000	2.919873000	-2.164502000
6	0.372478000	-3.219299000	3.893397000
6	3.311429000	-4.588963000	-1.020986000
6	7.679836000	1.420340000	-2.825513000
6	1.619092000	4.750924000	4.708180000
7	-0.499998000	-1.092366000	1.756206000
6	0.409958000	-3.886181000	5.276845000
6	4.552589000	-4.139913000	-1.822032000
7	0.997005000	-2.329203000	-0.566092000
8	8.069282000	0.780871000	-1.868160000
8	2.722897000	5.064742000	5.116962000
7	1.230883000	1.349943000	1.317904000
8	2.268624000	-4.306082000	6.711447000
8	6.512076000	-5.450460000	-1.211772000
8	2.326428000	-5.247602000	4.668308000

8	6.091844000	-3.516455000	-0.132383000
6	-0.671358000	1.055478000	2.890608000
6	-0.369961000	-3.548718000	1.075115000
6	2.448648000	-1.790329000	-2.459029000
6	2.810350000	2.429907000	-0.189219000
28	0.738307000	-0.346052000	0.160900000
6	-0.815044000	-0.435851000	2.812808000
6	0.418352000	-3.486292000	-0.005447000
6	2.640553000	-0.446526000	-2.266843000
6	2.045854000	2.326391000	1.004434000
6	-1.420434000	-1.290142000	3.930847000
6	0.716068000	-4.694865000	-0.899515000
6	3.298937000	0.466348000	-3.283042000
6	2.168492000	3.385166000	2.097730000
6	-1.007144000	-2.715025000	3.432375000
6	1.966889000	-4.182393000	-1.674432000
6	3.812938000	1.610506000	-2.377488000
6	0.978642000	3.013393000	3.013543000
6	-0.997847000	-2.477391000	1.897694000
6	1.784357000	-2.675563000	-1.572267000
6	2.905991000	1.471192000	-1.161678000
6	0.774552000	1.519660000	2.706527000
6	-2.972168000	-1.075947000	3.855513000
6	2.261330000	0.959707000	-4.307636000
6	2.136736000	4.847389000	1.582004000
7	-2.323081000	-6.102458000	-1.007724000
6	-3.744425000	-2.145393000	4.615653000
6	-1.835789000	-4.880054000	-1.381863000
6	2.906948000	1.724107000	-5.436187000
6	3.339219000	5.726446000	2.002369000
8	-3.737953000	-2.229690000	5.842690000
8	-2.493854000	-3.853429000	-1.215038000
8	4.102221000	1.812170000	-5.653983000
6	-0.430076000	-4.864608000	-1.958888000
6	5.294040000	1.440985000	-1.983088000
7	-4.440197000	-3.017853000	3.832547000
8	1.984074000	2.316511000	-6.223425000
6	-0.904536000	-0.896102000	5.322673000
6	0.912615000	-6.002661000	-0.123210000
6	6.246744000	1.784704000	-3.129093000
6	1.158012000	3.323935000	4.512778000
6	1.749372000	-4.558588000	5.488287000
6	5.814844000	-4.470394000	-1.068836000
7	2.293129000	0.249749000	-1.143137000
8	8.522881000	1.886901000	-3.777458000
8	0.708739000	5.649165000	4.285953000

6	-10.801662000	2.512461000	-2.343414000
6	-10.468123000	2.977528000	-0.916450000
6	-9.973597000	1.845091000	0.001492000
6	-8.759239000	1.028794000	-0.472573000
6	-7.377249000	1.672695000	-0.315117000
16	-7.002468000	2.997242000	-1.557936000
6	-0.728617000	-0.271984000	-2.849375000
8	-0.292263000	-0.884965000	-5.724874000
16	-1.190356000	0.329737000	-1.162729000
6	-1.657145000	-1.296128000	-3.483852000
8	-1.916627000	-2.792874000	-5.648645000
16	-0.842206000	-2.037385000	-4.942665000
8	0.240107000	-2.916589000	-4.387995000
6	-8.406793000	-2.315158000	-2.106974000
6	-7.112268000	-1.967060000	-1.411795000
6	-5.996440000	-1.530678000	-2.137576000
6	-6.996597000	-2.019091000	-0.014753000
6	-4.810594000	-1.156230000	-1.505779000
6	-5.823723000	-1.635687000	0.635657000
6	-4.718282000	-1.205381000	-0.110624000
8	-3.590856000	-0.834225000	0.564027000
6	4.616037000	-2.741828000	4.320249000
6	4.203390000	-2.998456000	2.864380000
6	3.575031000	-1.780110000	2.200806000
7	4.022611000	-1.489302000	0.955128000
8	2.706187000	-1.108552000	2.769869000
1	-1.352849000	8.382645000	0.219112000
1	-1.429925000	7.441366000	1.711799000
1	0.775006000	7.332312000	-1.384269000
1	-1.724138000	5.122129000	1.317498000
1	1.201977000	5.254338000	-2.678396000
1	-1.274974000	3.049569000	0.041771000
1	-0.202788000	2.126002000	-1.743973000
1	-8.262637000	-2.404388000	-3.188947000
1	-9.164509000	-1.538100000	-1.942573000
1	-6.049003000	-1.481042000	-3.222700000
1	-7.845167000	-2.351768000	0.579557000
1	-3.955401000	-0.829346000	-2.083908000
1	-5.762309000	-1.651132000	1.719949000
1	-2.861852000	-0.589033000	-0.067670000
1	0.703349000	-3.976069000	3.175077000
1	1.115056000	-2.417142000	3.831610000
1	3.348993000	-5.678086000	-0.923029000
1	3.355776000	-4.171282000	-0.008006000
1	0.220192000	-3.193812000	6.097296000
1	-0.356839000	-4.671530000	5.329886000

1	4.584401000	-4.654495000	-2.786166000
1	4.520257000	-3.062323000	-1.999907000
1	-1.287903000	1.507537000	2.103266000
1	-1.048133000	1.418159000	3.850548000
1	-0.765820000	-4.530051000	1.324472000
1	2.799946000	-2.219622000	-3.388995000
1	4.105715000	-0.046003000	-3.813433000
1	3.113896000	3.196054000	2.629863000
1	-1.763063000	-3.463886000	3.691189000
1	1.957674000	-4.508726000	-2.719685000
1	3.674082000	2.593679000	-2.840746000
1	0.099847000	3.558435000	2.647720000
1	-2.041263000	-2.431049000	1.545657000
1	1.428748000	0.904510000	3.339636000
1	-3.296018000	-1.027956000	2.813028000
1	-3.210647000	-0.110497000	4.314030000
1	1.683104000	0.136394000	-4.749564000
1	1.525803000	1.612217000	-3.823185000
1	1.223774000	5.312703000	1.948754000
1	2.047019000	4.867449000	0.494305000
1	3.666634000	5.462567000	3.011820000
1	-0.385156000	-4.040699000	-2.675244000
1	-0.228684000	-5.787810000	-2.517362000
1	5.466723000	0.408035000	-1.656535000
1	5.517882000	2.078218000	-1.120164000
1	0.186074000	-0.954125000	5.381834000
1	-1.346346000	-1.546836000	6.080131000
1	-1.198962000	0.129082000	5.570280000
1	1.258472000	-6.799216000	-0.792353000
1	1.635458000	-5.890765000	0.690036000
1	-0.032060000	-6.334724000	0.317108000
1	5.956838000	1.278862000	-4.057743000
1	6.204654000	2.854353000	-3.368388000
1	1.913571000	2.669791000	4.956361000
1	0.207958000	3.166109000	5.036565000
1	-9.892670000	2.430901000	-2.950259000
1	-11.477732000	3.210557000	-2.849131000
1	-9.727667000	3.786740000	-0.958813000
1	-11.364974000	3.410685000	-0.452767000
1	-9.757684000	2.254061000	0.999205000
1	-10.808837000	1.142124000	0.135314000
1	-8.723511000	0.108152000	0.123711000
1	-8.892972000	0.707044000	-1.511861000
1	-6.614163000	0.901204000	-0.445343000
1	-7.268439000	2.091633000	0.691168000
1	-5.586928000	2.752488000	-1.579604000

1	0.258024000	-0.716474000	-2.758111000
1	-0.640273000	0.591115000	-3.512124000
1	-1.902187000	-2.115882000	-2.802457000
1	-2.585335000	-0.841170000	-3.843666000
1	5.269423000	-1.865054000	4.396758000
1	3.746068000	-2.575132000	4.959705000
1	5.060715000	-3.349321000	2.280439000
1	3.452750000	-3.799275000	2.842012000
1	-8.842634000	-3.258550000	-1.752467000
1	0.094680000	8.160134000	1.199929000
1	5.174162000	-3.610027000	4.686937000
6	-3.950284000	2.227583000	-1.473952000
1	-4.054437000	1.576222000	-0.618032000
1	-3.444963000	3.173536000	-1.329385000
1	-3.866962000	1.752674000	-2.441597000
1	3.125984000	-4.772733000	6.758254000
1	6.894253000	-3.793781000	0.350095000
1	9.421287000	1.597492000	-3.527329000
1	2.466466000	2.763215000	-6.945371000
1	1.181179000	6.496879000	4.094603000
1	-4.916479000	-3.793889000	4.272944000
1	-4.382239000	-3.005938000	2.824190000
1	4.709878000	-2.071917000	0.493438000
1	3.532442000	-0.791265000	0.403903000
1	-3.297588000	-6.154670000	-0.739707000
1	-11.265584000	1.518086000	-2.340180000
1	3.390964000	3.334754000	-0.330139000
1	4.183578000	5.607399000	1.318921000
6	2.910141000	7.171161000	2.062112000
8	2.107684000	7.625663000	2.869009000
8	3.467528000	7.941093000	1.114507000
1	3.112086000	8.845464000	1.222629000
1	-1.883877000	-6.956127000	-1.320767000

Int_2
C-Int_2-opt.gjf.log

Temperature 298.150 Kelvin. Pressure 1.00000 Atm.

Zero-point correction= 1.542407 (Hartree/Particle)
Thermal correction to Energy= 1.646139
Thermal correction to Enthalpy= 1.647083
Thermal correction to Gibbs Free Energy= 1.383073
Sum of electronic and zero-point Energies= -5861.141323
Sum of electronic and thermal Energies= -5861.037591
Sum of electronic and thermal Enthalpies= -5861.036646

Sum of electronic and thermal Free Energies= -5861.300657

6	-0.626736000	7.600580000	1.360675000
6	-0.379354000	6.381250000	0.505554000
6	0.443457000	6.437227000	-0.629899000
6	-0.981366000	5.153227000	0.807568000
6	0.651064000	5.318386000	-1.435895000
6	-0.779990000	4.020142000	0.018944000
6	0.033747000	4.098498000	-1.120849000
8	0.246752000	3.039072000	-1.944140000
6	0.391087000	-3.494694000	3.705979000
6	3.171896000	-4.591395000	-1.308617000
6	7.593383000	1.512499000	-2.901076000
6	1.833584000	4.388312000	5.020556000
7	-0.490638000	-1.206073000	1.753613000
6	0.453800000	-4.259089000	5.036449000
6	4.414778000	-4.120779000	-2.094832000
7	0.923068000	-2.313480000	-0.680326000
8	8.009808000	0.823642000	-1.990502000
8	2.959288000	4.649141000	5.406453000
7	1.273449000	1.227091000	1.431804000
8	2.315173000	-4.871162000	6.394825000
8	6.328772000	-5.526724000	-1.559749000
8	2.327223000	-5.599957000	4.265853000
8	6.006805000	-3.610522000	-0.416145000
6	-0.586410000	0.860564000	3.038478000
6	-0.447066000	-3.611050000	0.896730000
6	2.348587000	-1.684396000	-2.565428000
6	2.824583000	2.379854000	-0.050738000
28	0.707878000	-0.373635000	0.178209000
6	-0.761941000	-0.618856000	2.862058000
6	0.322708000	-3.492365000	-0.192227000
6	2.554318000	-0.353965000	-2.300371000
6	2.095543000	2.209064000	1.157610000
6	-1.356484000	-1.536987000	3.934229000
6	0.573570000	-4.640553000	-1.175457000
6	3.190252000	0.614828000	-3.278559000
6	2.268897000	3.191936000	2.312923000
6	-0.990346000	-2.931136000	3.324990000
6	1.833318000	-4.111090000	-1.922573000
6	3.743615000	1.695805000	-2.320085000
6	1.100339000	2.779795000	3.238647000
6	-1.021030000	-2.585525000	1.811494000
6	1.688641000	-2.610620000	-1.719790000
6	2.873995000	1.487407000	-1.086955000
6	0.861209000	1.312710000	2.841959000

6	-2.904400000	-1.284131000	3.919355000
6	2.120110000	1.179561000	-4.230476000
6	2.245143000	4.684443000	1.893181000
7	-2.488056000	-5.989219000	-1.391876000
6	-3.680389000	-2.390187000	4.620631000
6	-1.985372000	-4.742808000	-1.648854000
6	2.722412000	2.027448000	-5.321995000
6	3.476173000	5.517047000	2.325919000
8	-3.649855000	-2.560590000	5.838294000
8	-2.627200000	-3.728515000	-1.377560000
8	3.908540000	2.148061000	-5.569722000
6	-0.583856000	-4.692373000	-2.234755000
6	5.234816000	1.490857000	-1.985988000
7	-4.409725000	-3.192550000	3.793789000
8	1.767072000	2.662514000	-6.035458000
6	-0.793704000	-1.255184000	5.334907000
6	0.736088000	-6.008117000	-0.501899000
6	6.153210000	1.896925000	-3.139505000
6	1.330800000	2.988202000	4.748871000
6	1.777758000	-4.982685000	5.158716000
6	5.675762000	-4.523370000	-1.375311000
7	2.243553000	0.274604000	-1.128378000
8	8.408154000	2.024715000	-3.854162000
8	0.931175000	5.332767000	4.693390000
6	-10.781863000	2.865166000	-1.933431000
6	-10.415465000	3.229420000	-0.484729000
6	-9.987759000	2.022963000	0.367667000
6	-8.809551000	1.185841000	-0.154358000
6	-7.424371000	1.835689000	-0.028775000
16	-7.018667000	3.124569000	-1.242538000
6	-0.808723000	-0.166944000	-2.746198000
8	-0.590636000	-0.450137000	-5.745663000
16	-1.241353000	0.421640000	-1.048109000
6	-1.857717000	-0.989746000	-3.473612000
8	-2.267569000	-2.313795000	-5.726788000
16	-1.139105000	-1.643308000	-5.020022000
8	-0.054752000	-2.585561000	-4.587755000
6	-8.487422000	-2.015639000	-2.032492000
6	-7.188134000	-1.711367000	-1.325844000
6	-6.095146000	-1.180522000	-2.024069000
6	-7.042767000	-1.899427000	0.056389000
6	-4.897810000	-0.860405000	-1.383127000
6	-5.856160000	-1.578785000	0.716399000
6	-4.765254000	-1.072010000	-0.005003000
8	-3.612267000	-0.809260000	0.671975000
6	4.633512000	-3.095390000	4.112560000

6	4.215107000	-3.239438000	2.644096000
6	3.586762000	-1.971867000	2.080546000
7	3.985343000	-1.618541000	0.834364000
8	2.757973000	-1.319694000	2.726160000
1	-1.165985000	8.376741000	0.800787000
1	-1.237183000	7.347575000	2.234469000
1	0.935028000	7.373433000	-0.887371000
1	-1.619389000	5.074347000	1.685482000
1	1.288161000	5.369243000	-2.314443000
1	-1.251898000	3.076564000	0.272431000
1	-0.219647000	2.235285000	-1.581501000
1	-8.345334000	-2.052460000	-3.117956000
1	-9.238838000	-1.240591000	-1.832106000
1	-6.173168000	-1.016774000	-3.096445000
1	-7.874911000	-2.300300000	0.631620000
1	-4.061038000	-0.462618000	-1.944184000
1	-5.767276000	-1.714990000	1.790286000
1	-2.892602000	-0.519377000	0.047802000
1	0.683792000	-4.204249000	2.925605000
1	1.149035000	-2.704851000	3.679857000
1	3.184380000	-5.685066000	-1.279875000
1	3.235092000	-4.239339000	-0.271800000
1	0.310106000	-3.622682000	5.910034000
1	-0.334092000	-5.024996000	5.061361000
1	4.425517000	-4.579019000	-3.087552000
1	4.403832000	-3.034353000	-2.209655000
1	-1.216782000	1.376494000	2.302955000
1	-0.927717000	1.162147000	4.032244000
1	-0.866732000	-4.596295000	1.084020000
1	2.683629000	-2.062946000	-3.523535000
1	3.973651000	0.131005000	-3.867590000
1	3.226313000	2.954821000	2.802553000
1	-1.754497000	-3.681140000	3.554414000
1	1.811123000	-4.370549000	-2.986508000
1	3.598681000	2.707046000	-2.715898000
1	0.220796000	3.361688000	2.936087000
1	-2.072596000	-2.487883000	1.496244000
1	1.522849000	0.647477000	3.413007000
1	-3.255945000	-1.152757000	2.893031000
1	-3.107150000	-0.350403000	4.454224000
1	1.523719000	0.390758000	-4.708630000
1	1.406589000	1.797056000	-3.673977000
1	1.352858000	5.138614000	2.320154000
1	2.119172000	4.775660000	0.813017000
1	3.833374000	5.185436000	3.304712000
1	-0.536381000	-3.806558000	-2.873744000

1	-0.401602000	-5.562581000	-2.878338000
1	5.407562000	0.438552000	-1.728463000
1	5.492731000	2.072456000	-1.093808000
1	0.296391000	-1.341293000	5.358418000
1	-1.230285000	-1.949014000	6.056440000
1	-1.056547000	-0.244251000	5.663434000
1	1.052677000	-6.761624000	-1.232651000
1	1.469003000	-5.978255000	0.309484000
1	-0.214253000	-6.344919000	-0.077955000
1	5.833259000	1.445934000	-4.086354000
1	6.108342000	2.978603000	-3.315887000
1	2.082706000	2.289407000	5.125419000
1	0.392998000	2.818425000	5.290573000
1	-9.884826000	2.799616000	-2.558865000
1	-11.450293000	3.607458000	-2.382521000
1	-9.625705000	3.995315000	-0.486868000
1	-11.278195000	3.695613000	0.010069000
1	-9.758281000	2.361262000	1.388447000
1	-10.854735000	1.352216000	0.456147000
1	-8.765464000	0.254819000	0.424356000
1	-8.975972000	0.892661000	-1.196310000
1	-6.657121000	1.064840000	-0.207939000
1	-7.256249000	2.218506000	0.984615000
1	-2.339022000	3.341921000	-5.385864000
1	0.085637000	-0.773441000	-2.654347000
1	-0.558710000	0.703310000	-3.350322000
1	-2.186869000	-1.850978000	-2.884934000
1	-2.726618000	-0.390939000	-3.763386000
1	5.298719000	-2.235271000	4.250928000
1	3.766744000	-2.962470000	4.764144000
1	5.068058000	-3.546766000	2.030048000
1	3.462166000	-4.034545000	2.563000000
1	-8.937101000	-2.969710000	-1.727255000
1	0.293277000	8.065155000	1.738380000
1	5.179192000	-3.995363000	4.415874000
6	-1.938456000	2.624895000	-4.667177000
1	-2.709652000	2.369900000	-3.938362000
1	-1.094916000	3.064173000	-4.135041000
1	-1.611810000	1.721010000	-5.181190000
1	3.158863000	-5.364113000	6.383846000
1	6.803670000	-3.935854000	0.045225000
1	9.312701000	1.720612000	-3.647337000
1	2.217810000	3.170125000	-6.737290000
1	1.416439000	6.181320000	4.541541000
1	-4.884215000	-3.994091000	4.188101000
1	-4.362706000	-3.116052000	2.787629000

1	4.647042000	-2.181710000	0.314898000
1	3.489547000	-0.880206000	0.344028000
1	-3.461817000	-6.053628000	-1.123859000
1	-11.266956000	1.882098000	-1.981297000
1	3.413825000	3.284489000	-0.151120000
1	4.294096000	5.429088000	1.606523000
6	3.072011000	6.960656000	2.489813000
8	2.314838000	7.376519000	3.358943000
8	3.593937000	7.777692000	1.561515000
1	3.256096000	8.678139000	1.737292000
1	-2.063496000	-6.814081000	-1.790765000

Int_3
C-Int_3-opt.gjf.log
Temperature 298.150 Kelvin. Pressure 1.00000 Atm.

Zero-point correction= 1.542418 (Hartree/Particle)
Thermal correction to Energy= 1.646389
Thermal correction to Enthalpy= 1.647333
Thermal correction to Gibbs Free Energy= 1.381540
Sum of electronic and zero-point Energies= -5861.144119
Sum of electronic and thermal Energies= -5861.040148
Sum of electronic and thermal Enthalpies= -5861.039204
Sum of electronic and thermal Free Energies= -5861.304996

6	-0.349783000	7.607557000	1.461458000
6	-0.171705000	6.388904000	0.588675000
6	0.629745000	6.421274000	-0.562751000
6	-0.825014000	5.186597000	0.888244000
6	0.768050000	5.304383000	-1.386274000
6	-0.694170000	4.055795000	0.081537000
6	0.100263000	4.111029000	-1.073218000
8	0.246007000	3.053185000	-1.913874000
6	0.273771000	-3.513931000	3.712050000
6	2.892096000	-4.708140000	-1.392632000
6	7.530952000	1.247379000	-3.025475000
6	2.046907000	4.285022000	5.034115000
7	-0.558478000	-1.190808000	1.783486000
6	0.333563000	-4.285052000	5.039582000
6	4.133453000	-4.284307000	-2.207214000
7	0.759920000	-2.339868000	-0.686189000
8	7.944207000	0.551124000	-2.119102000
8	3.186506000	4.499380000	5.407572000
7	1.293721000	1.171796000	1.436391000
8	2.230501000	-4.890212000	6.352522000
8	6.014273000	-5.761299000	-1.749792000

8	2.149191000	-5.691326000	4.251051000
8	5.772558000	-3.869208000	-0.548067000
6	-0.549121000	0.872533000	3.076252000
6	-0.622465000	-3.591696000	0.917412000
6	2.166599000	-1.755399000	-2.599724000
6	2.857460000	2.271771000	-0.072648000
28	0.643270000	-0.400051000	0.187535000
6	-0.787621000	-0.597658000	2.898278000
6	0.123940000	-3.496356000	-0.190029000
6	2.434655000	-0.436809000	-2.329883000
6	2.148281000	2.122008000	1.150477000
6	-1.397699000	-1.496625000	3.978244000
6	0.299334000	-4.645594000	-1.187834000
6	3.086158000	0.513507000	-3.316220000
6	2.384800000	3.089274000	2.307982000
6	-1.093841000	-2.901081000	3.358533000
6	1.559887000	-4.163873000	-1.965660000
6	3.702731000	1.565450000	-2.364023000
6	1.218910000	2.718814000	3.254481000
6	-1.139426000	-2.549144000	1.846910000
6	1.486472000	-2.660317000	-1.746573000
6	2.852565000	1.383072000	-1.113321000
6	0.912347000	1.265285000	2.854525000
6	-2.935328000	-1.187774000	3.990825000
6	2.018288000	1.127715000	-4.239802000
6	2.413537000	4.584145000	1.897842000
7	-2.831291000	-5.844669000	-1.321033000
6	-3.738907000	-2.264892000	4.706127000
6	-2.274574000	-4.623329000	-1.587318000
6	2.629378000	1.956793000	-5.340731000
6	3.681971000	5.365351000	2.319009000
8	-3.690269000	-2.438719000	5.922754000
8	-2.857100000	-3.580095000	-1.292515000
8	3.813349000	2.029783000	-5.615999000
6	-0.889501000	-4.638752000	-2.213514000
6	5.191661000	1.300939000	-2.063771000
7	-4.514903000	-3.037617000	3.893813000
8	1.684647000	2.635004000	-6.028116000
6	-0.800980000	-1.239289000	5.369708000
6	0.420731000	-6.024905000	-0.529712000
6	6.099506000	1.678167000	-3.235226000
6	1.486032000	2.907434000	4.761065000
6	1.644898000	-5.033611000	5.141693000
6	5.395044000	-4.744921000	-1.524570000
7	2.176911000	0.194696000	-1.146938000
8	8.341266000	1.726388000	-3.999444000

8	1.177997000	5.266134000	4.724303000
6	-10.728098000	3.266438000	-1.676629000
6	-10.108930000	3.475013000	-0.286914000
6	-9.276262000	2.279605000	0.208951000
6	-8.005219000	1.951742000	-0.595615000
6	-6.905814000	3.017276000	-0.446211000
16	-5.355165000	2.558990000	-1.300927000
6	-0.920575000	-0.113637000	-2.710638000
8	-0.759235000	-0.399328000	-5.711541000
16	-1.290984000	0.483042000	-1.000688000
6	-2.015122000	-0.890353000	-3.421773000
8	-2.518359000	-2.185644000	-5.672205000
16	-1.349996000	-1.569250000	-4.981356000
8	-0.302379000	-2.561105000	-4.569817000
6	-8.610731000	-1.691229000	-1.864940000
6	-7.283240000	-1.455643000	-1.186831000
6	-6.186835000	-0.965246000	-1.901621000
6	-7.128845000	-1.636902000	0.197725000
6	-4.977267000	-0.659921000	-1.272457000
6	-5.930429000	-1.340319000	0.845100000
6	-4.835872000	-0.862542000	0.109714000
8	-3.671385000	-0.623973000	0.767386000
6	4.579138000	-3.294024000	4.012530000
6	4.092036000	-3.422949000	2.563533000
6	3.497372000	-2.130182000	2.019845000
7	3.872157000	-1.792256000	0.761952000
8	2.715387000	-1.445600000	2.689995000
1	-0.871944000	8.409365000	0.921646000
1	-0.950748000	7.368722000	2.345847000
1	1.159751000	7.336624000	-0.818912000
1	-1.448671000	5.126314000	1.777840000
1	1.389095000	5.336377000	-2.277067000
1	-1.206330000	3.132799000	0.332519000
1	-0.240182000	2.263202000	-1.544297000
1	-8.499186000	-1.715543000	-2.954170000
1	-9.318227000	-0.883450000	-1.629984000
1	-6.270790000	-0.804450000	-2.973566000
1	-7.968318000	-2.005149000	0.784058000
1	-4.132315000	-0.301894000	-1.845749000
1	-5.834253000	-1.470003000	1.919119000
1	-2.953647000	-0.352192000	0.130982000
1	0.527814000	-4.230499000	2.924458000
1	1.058162000	-2.750762000	3.674726000
1	2.857142000	-5.801570000	-1.374275000
1	2.998091000	-4.370107000	-0.354734000
1	0.212218000	-3.648334000	5.916083000

1	-0.469204000	-5.034840000	5.067705000
1	4.099513000	-4.733071000	-3.203637000
1	4.165960000	-3.197247000	-2.312658000
1	-1.171236000	1.416735000	2.354269000
1	-0.859390000	1.183915000	4.077119000
1	-1.075744000	-4.560972000	1.109682000
1	2.461344000	-2.138781000	-3.569008000
1	3.836452000	0.003710000	-3.926049000
1	3.341440000	2.809786000	2.776375000
1	-1.880165000	-3.623816000	3.600240000
1	1.497115000	-4.411459000	-3.030753000
1	3.589043000	2.584094000	-2.750970000
1	0.357991000	3.337614000	2.972282000
1	-2.192143000	-2.410893000	1.550821000
1	1.556718000	0.569633000	3.409096000
1	-3.299564000	-1.042401000	2.970936000
1	-3.094749000	-0.247453000	4.528836000
1	1.378819000	0.367289000	-4.708241000
1	1.343936000	1.770785000	-3.663569000
1	1.545257000	5.069442000	2.339823000
1	2.277086000	4.687773000	0.820077000
1	4.038613000	5.014887000	3.291255000
1	-0.817550000	-3.754027000	-2.851509000
1	-0.767470000	-5.514338000	-2.864061000
1	5.329151000	0.241328000	-1.814982000
1	5.491243000	1.867192000	-1.174763000
1	0.285538000	-1.364953000	5.374619000
1	-1.250253000	-1.918169000	6.097591000
1	-1.021181000	-0.220244000	5.704385000
1	0.684756000	-6.784578000	-1.274794000
1	1.175593000	-6.034148000	0.261787000
1	-0.531814000	-6.324313000	-0.083125000
1	5.746180000	1.239595000	-4.175981000
1	6.086583000	2.761086000	-3.409597000
1	2.217192000	2.177431000	5.118795000
1	0.552647000	2.769509000	5.319246000
1	-9.962905000	3.261021000	-2.461710000
1	-11.446070000	4.058123000	-1.918196000
1	-9.497072000	4.387711000	-0.287590000
1	-10.913213000	3.653275000	0.439785000
1	-8.993938000	2.447400000	1.258075000
1	-9.916807000	1.385443000	0.205638000
1	-7.610607000	0.995437000	-0.242111000
1	-8.240058000	1.814857000	-1.657737000
1	-6.702643000	3.201888000	0.614218000
1	-7.228196000	3.966707000	-0.895355000

1	-2.048860000	3.533834000	-5.447763000
1	-0.049014000	-0.754939000	-2.640476000
1	-0.649617000	0.749622000	-3.315693000
1	-2.369619000	-1.738912000	-2.829491000
1	-2.863102000	-0.255310000	-3.696219000
1	5.285932000	-2.463040000	4.119111000
1	3.747166000	-3.122804000	4.699669000
1	4.903388000	-3.770183000	1.915412000
1	3.301448000	-4.183453000	2.518531000
1	-9.088081000	-2.631748000	-1.559711000
1	0.593229000	8.035619000	1.825340000
1	5.098222000	-4.215653000	4.296915000
6	-2.018750000	2.738443000	-4.700771000
1	-3.017320000	2.595991000	-4.283995000
1	-1.333552000	3.016811000	-3.899918000
1	-1.679556000	1.810446000	-5.161130000
1	3.065281000	-5.397645000	6.326752000
1	6.568575000	-4.230585000	-0.112878000
1	9.239762000	1.394582000	-3.809487000
1	2.139087000	3.127630000	-6.738158000
1	1.693933000	6.096353000	4.572364000
1	-5.015282000	-3.818250000	4.297870000
1	-4.491144000	-2.958346000	2.887112000
1	4.495196000	-2.381014000	0.223532000
1	3.394434000	-1.032654000	0.286258000
1	-3.799319000	-5.861139000	-1.026179000
1	-11.248538000	2.301579000	-1.723992000
1	3.478844000	3.153683000	-0.181102000
1	4.486022000	5.249247000	1.588045000
6	3.335433000	6.822425000	2.494244000
8	2.618728000	7.264612000	3.384433000
8	3.857920000	7.620535000	1.549861000
1	3.556018000	8.532061000	1.733638000
1	-2.462402000	-6.686676000	-1.739003000

TS_3
C-TS_3-opt.gjf.log

Temperature 298.150 Kelvin. Pressure 1.00000 Atm.

Zero-point correction= 1.542438 (Hartree/Particle)
Thermal correction to Energy= 1.644420
Thermal correction to Enthalpy= 1.645364
Thermal correction to Gibbs Free Energy= 1.387690
Sum of electronic and zero-point Energies= -5861.142980
Sum of electronic and thermal Energies= -5861.040998

Sum of electronic and thermal Enthalpies= -5861.040054
Sum of electronic and thermal Free Energies= -5861.297728

6	2.652157000	-6.444041000	-3.868157000
6	2.171258000	-5.666660000	-2.673360000
6	2.531741000	-5.974573000	-1.353807000
6	1.190330000	-4.684136000	-2.867146000
6	1.910742000	-5.350128000	-0.269257000
6	0.560485000	-4.050948000	-1.800014000
6	0.901608000	-4.403142000	-0.487929000
8	0.265870000	-3.865422000	0.593895000
6	0.686821000	4.639501000	-1.229192000
6	0.466896000	3.773543000	3.573837000
6	5.372674000	-2.339205000	5.172187000
6	4.939836000	-0.961891000	-4.651848000
7	-0.583295000	1.809565000	-1.546401000
6	1.356201000	5.936778000	-1.688012000
6	1.574050000	3.115368000	4.423975000
7	-0.738433000	1.741178000	1.446762000
8	6.037165000	-1.375949000	4.844169000
8	6.128201000	-0.879299000	-4.396968000
7	1.732655000	-0.139041000	-1.143203000
8	3.245821000	7.272821000	-1.155109000
8	3.148191000	4.689351000	5.405453000
8	2.219428000	6.247890000	0.566185000
8	3.493626000	3.958115000	3.304366000
6	0.754357000	0.710215000	-3.251482000
6	-1.722703000	3.488707000	0.012635000
6	-0.059657000	0.510915000	3.457689000
6	2.746001000	-1.773128000	0.357549000
28	0.224978000	0.542054000	0.088068000
6	0.049477000	1.890102000	-2.668782000
6	-1.454801000	2.942683000	1.205892000
6	0.633636000	-0.535995000	2.902988000
6	2.628216000	-1.062982000	-0.862225000
6	0.002412000	3.260054000	-3.346208000
6	-1.904672000	3.562893000	2.543190000
6	1.111617000	-1.737767000	3.695306000
6	3.634859000	-1.305634000	-1.984435000
6	-0.451378000	4.130814000	-2.130455000
6	-0.835071000	2.935513000	3.482797000
6	2.267600000	-2.259855000	2.807488000
6	2.950909000	-0.591713000	-3.169904000
6	-1.318885000	3.089803000	-1.362850000
6	-0.539776000	1.636881000	2.760260000
6	2.005955000	-1.543010000	1.486533000

6	2.040499000	0.429986000	-2.470824000
6	-1.112869000	3.168934000	-4.447177000
6	-0.040466000	-2.743404000	3.874098000
6	3.955547000	-2.799436000	-2.246191000
7	-5.574046000	2.517249000	2.487430000
6	-1.607141000	4.541484000	-4.887021000
6	-4.509708000	3.301336000	2.171837000
6	0.265083000	-3.804430000	4.902295000
6	5.456105000	-3.176833000	-2.195470000
8	-0.882657000	5.362943000	-5.447352000
8	-4.582653000	4.210012000	1.340411000
8	1.181193000	-3.791321000	5.703373000
6	-3.271369000	3.000009000	3.018809000
6	3.662406000	-1.907259000	3.361112000
7	-2.907882000	4.806085000	-4.588253000
8	-0.640874000	-4.805944000	4.861670000
6	1.340958000	3.651637000	-3.993126000
6	-1.925077000	5.098141000	2.549654000
6	4.055919000	-2.760335000	4.567018000
6	3.899922000	0.041999000	-4.207463000
6	2.288295000	6.476744000	-0.627450000
6	2.796779000	3.994830000	4.476679000
7	1.036158000	-0.591347000	1.604185000
8	5.751502000	-3.175543000	6.168724000
8	4.394699000	-2.022215000	-5.278859000
6	-8.804961000	-4.348192000	-3.005852000
6	-7.388424000	-3.830842000	-3.420134000
6	-6.169362000	-4.187433000	-2.526138000
6	-5.990670000	-3.361513000	-1.233902000
6	-4.600667000	-3.494028000	-0.559220000
16	-3.256845000	-2.701739000	-1.588550000
6	-2.327537000	-1.607304000	1.341191000
8	-4.542681000	-2.501591000	3.298190000
16	-1.702039000	-1.479097000	-0.373557000
6	-3.517873000	-0.727671000	1.655463000
8	-5.278897000	-0.126528000	3.518331000
16	-4.124669000	-1.069456000	3.330175000
8	-2.978462000	-0.787832000	4.244593000
6	-7.775752000	0.303617000	-0.476456000
6	-6.463089000	0.411621000	-1.216035000
6	-5.329997000	0.876372000	-0.545679000
6	-6.333547000	0.095837000	-2.577235000
6	-4.090795000	0.955787000	-1.171384000
6	-5.098090000	0.168046000	-3.222810000
6	-3.965359000	0.557385000	-2.502394000
8	-2.739256000	0.515697000	-3.118463000

6	5.993213000	4.926661000	-0.836230000
6	5.023357000	4.381369000	0.220155000
6	4.067141000	3.318457000	-0.348106000
7	3.387536000	2.589604000	0.572617000
8	3.914117000	3.152107000	-1.563920000
1	2.186611000	-7.441449000	-3.867413000
1	2.309508000	-5.956709000	-4.788396000
1	3.301147000	-6.720274000	-1.167717000
1	0.895111000	-4.418478000	-3.879648000
1	2.185006000	-5.597998000	0.752157000
1	-0.211941000	-3.311096000	-1.978512000
1	-0.304957000	-3.121388000	0.284784000
1	-7.620030000	0.118596000	0.591483000
1	-8.411154000	-0.492516000	-0.879295000
1	-5.405424000	1.149657000	0.499903000
1	-7.203035000	-0.248010000	-3.133748000
1	-3.216650000	1.262127000	-0.608050000
1	-4.989628000	-0.129722000	-4.261583000
1	-2.057989000	0.578369000	-2.420033000
1	0.272951000	4.820679000	-0.233610000
1	1.445706000	3.860932000	-1.102504000
1	0.227946000	4.747228000	4.013809000
1	0.849815000	3.961292000	2.563638000
1	1.919665000	5.829351000	-2.616149000
1	0.599630000	6.712465000	-1.875617000
1	1.228168000	2.961805000	5.449715000
1	1.849364000	2.144536000	4.001696000
1	0.114023000	-0.177297000	-3.197175000
1	0.991523000	0.892470000	-4.303420000
1	-2.345240000	4.377589000	0.022311000
1	-0.216293000	0.500767000	4.530205000
1	1.459144000	-1.434046000	4.687075000
1	4.556598000	-0.766579000	-1.715649000
1	-1.069904000	4.977537000	-2.444947000
1	-1.220631000	2.768746000	4.495022000
1	2.201642000	-3.343010000	2.651806000
1	2.315622000	-1.326852000	-3.680808000
1	-2.257074000	2.967836000	-1.927883000
1	2.586996000	1.370226000	-2.314482000
1	-1.942417000	2.544202000	-4.104927000
1	-0.688435000	2.672639000	-5.326279000
1	-0.960632000	-2.231719000	4.192261000
1	-0.275510000	-3.238065000	2.926354000
1	3.562862000	-3.064725000	-3.227713000
1	3.417066000	-3.430731000	-1.540209000
1	6.067970000	-2.362693000	-2.593315000

1	-3.198276000	1.917680000	3.178273000
1	-3.474958000	3.421001000	4.013823000
1	3.683856000	-0.845113000	3.635811000
1	4.412114000	-2.032977000	2.572536000
1	2.163319000	3.642507000	-3.273517000
1	1.251469000	4.641766000	-4.444701000
1	1.595591000	2.954682000	-4.799021000
1	-2.052563000	5.466552000	3.575132000
1	-0.999028000	5.516208000	2.144492000
1	-2.762807000	5.465048000	1.956915000
1	3.292135000	-2.728250000	5.352136000
1	4.130033000	-3.819842000	4.290007000
1	4.424422000	0.897861000	-3.774352000
1	3.321786000	0.386589000	-5.072911000
1	-8.760587000	-4.961390000	-2.097497000
1	-9.252168000	-4.960613000	-3.795064000
1	-7.164437000	-4.215275000	-4.423155000
1	-7.415545000	-2.738418000	-3.528193000
1	-6.192398000	-5.258201000	-2.275568000
1	-5.272187000	-4.042060000	-3.144433000
1	-6.168684000	-2.302947000	-1.446663000
1	-6.739912000	-3.665899000	-0.488376000
1	-4.333487000	-4.544195000	-0.404326000
1	-4.632042000	-3.018799000	0.425675000
1	-2.315682000	-5.987381000	2.984694000
1	-1.484924000	-1.309018000	1.962181000
1	-2.548869000	-2.655175000	1.548745000
1	-3.245200000	0.327121000	1.598385000
1	-4.349977000	-0.909821000	0.978031000
1	6.836396000	4.244374000	-0.986973000
1	5.468117000	5.009561000	-1.792701000
1	5.561195000	3.956205000	1.076381000
1	4.387692000	5.178110000	0.624750000
1	-8.315108000	1.254011000	-0.583652000
1	3.721215000	-6.635589000	-4.027372000
1	6.391272000	5.921073000	-0.607303000
6	-2.688370000	-5.365046000	2.171512000
1	-3.317248000	-5.958583000	1.506480000
1	-1.837719000	-4.973329000	1.612532000
1	-3.269090000	-4.541014000	2.586699000
1	3.783841000	7.609512000	-0.412705000
1	4.243431000	4.577551000	3.389462000
1	6.597114000	-2.834511000	6.518133000
1	-0.409782000	-5.435917000	5.571070000
1	5.028470000	-2.778829000	-5.217248000
1	-3.295774000	5.703753000	-4.845672000

1	-3.513749000	4.133064000	-4.142048000
1	3.398248000	2.846848000	1.553265000
1	2.669489000	1.941753000	0.259650000
1	-6.417346000	2.658590000	1.945940000
1	-9.487084000	-3.511877000	-2.807522000
1	3.505686000	-2.543995000	0.411919000
1	5.778593000	-3.388316000	-1.172946000
6	5.708750000	-4.368395000	-3.083957000
8	5.625551000	-4.354332000	-4.305812000
8	6.014004000	-5.490793000	-2.411587000
1	6.139839000	-6.203102000	-3.068950000
1	-5.462805000	1.617560000	2.968386000

PC
C-PC-opt.gjf.log

Temperature 298.150 Kelvin. Pressure 1.00000 Atm.

Zero-point correction= 1.543809 (Hartree/Particle)
Thermal correction to Energy= 1.646787
Thermal correction to Enthalpy= 1.647732
Thermal correction to Gibbs Free Energy= 1.388881
Sum of electronic and zero-point Energies= -5861.172440
Sum of electronic and thermal Energies= -5861.069461
Sum of electronic and thermal Enthalpies= -5861.068517
Sum of electronic and thermal Free Energies= -5861.327367

6	0.298643000	7.663421000	-2.300156000
6	0.226618000	6.517020000	-1.322173000
6	-0.310663000	6.662188000	-0.033935000
6	0.788125000	5.276377000	-1.651852000
6	-0.264184000	5.623059000	0.896394000
6	0.829107000	4.220341000	-0.741701000
6	0.320606000	4.398240000	0.550406000
8	0.377025000	3.421391000	1.505085000
6	-1.500896000	-3.725329000	-2.991967000
6	-2.744802000	-3.984767000	2.284149000
6	-4.831734000	0.482046000	5.299572000
6	-2.699479000	3.887046000	-4.886648000
7	0.389418000	-1.451496000	-1.765531000
6	-2.175003000	-4.495294000	-4.133987000
6	-3.592863000	-3.285844000	3.366300000
7	-0.370043000	-2.244860000	0.991912000
8	-4.508984000	-0.641765000	4.962353000
8	-3.862905000	4.134569000	-5.149657000
7	-1.370213000	0.985572000	-1.227747000

8	-4.399861000	-5.141193000	-4.679107000
8	-5.942803000	-3.859102000	3.649929000
8	-3.734059000	-5.502321000	-2.560921000
8	-5.343882000	-2.476044000	1.980419000
6	0.089277000	0.511074000	-3.162149000
6	0.357390000	-3.800487000	-0.768410000
6	-1.047931000	-1.363413000	3.167962000
6	-2.490774000	2.305919000	0.481705000
28	-0.593633000	-0.506340000	-0.066401000
6	0.218370000	-0.962794000	-2.947583000
6	-0.095389000	-3.528240000	0.462471000
6	-1.323158000	-0.059892000	2.843635000
6	-2.106090000	2.012799000	-0.848372000
6	0.135851000	-1.987130000	-4.083048000
6	-0.316439000	-4.608849000	1.536458000
6	-1.763177000	0.973953000	3.858609000
6	-2.579228000	2.888140000	-2.010351000
6	-0.047180000	-3.301989000	-3.254899000
6	-1.219553000	-3.832725000	2.534171000
6	-2.667196000	1.873492000	2.985087000
6	-1.628809000	2.417981000	-3.135952000
6	0.657908000	-2.905527000	-1.919547000
6	-0.820905000	-2.397813000	2.233850000
6	-2.142245000	1.569738000	1.586333000
6	-1.291357000	0.984911000	-2.699644000
6	1.497103000	-1.973032000	-4.824992000
6	-0.558013000	1.729542000	4.440669000
6	-2.532686000	4.412616000	-1.736766000
7	3.253093000	-4.376247000	1.609433000
6	1.622727000	-2.987328000	-5.964072000
6	2.205243000	-5.212033000	1.417900000
6	-0.954938000	2.607313000	5.602338000
6	-3.830649000	5.193624000	-2.060864000
6	-3.471959000	6.623828000	-2.375626000
8	0.849681000	-3.926019000	-6.145774000
8	2.226867000	-6.136237000	0.597145000
8	-2.067688000	2.695273000	6.089330000
6	1.006315000	-4.914061000	2.307273000
6	-4.172011000	1.521204000	3.054541000
7	2.687711000	-2.764424000	-6.786313000
8	0.093470000	3.314863000	6.078063000
8	-2.923819000	6.995429000	-3.406582000
6	-0.994210000	-1.628025000	-5.067596000
6	-0.914000000	-5.906914000	0.977839000
6	-4.808698000	1.724862000	4.436933000
6	-2.170184000	2.505318000	-4.575750000

6	-3.487329000	-5.093467000	-3.680512000
6	-5.064156000	-3.272266000	3.056057000
7	-1.397444000	0.428441000	1.570150000
8	-5.295097000	0.754690000	6.539272000
8	-1.757466000	4.841872000	-4.758585000
6	10.534848000	2.292937000	-0.634080000
6	9.057857000	2.380169000	-1.049258000
6	8.054218000	1.799055000	-0.038490000
6	6.608674000	2.237057000	-0.344742000
6	5.577892000	1.564501000	0.569360000
16	3.956210000	2.478032000	0.529967000
6	2.210707000	0.310408000	2.059641000
8	2.241326000	-0.364034000	5.028466000
16	2.435339000	1.031867000	0.376569000
6	3.514867000	-0.090130000	2.724039000
8	4.555873000	-1.304234000	4.810357000
16	3.218047000	-1.121553000	4.189003000
8	2.639475000	-2.394652000	3.635304000
6	7.955621000	-2.325988000	0.407538000
6	6.697982000	-1.915834000	-0.319109000
6	5.436469000	-2.210808000	0.213172000
6	6.746996000	-1.252392000	-1.553129000
6	4.259108000	-1.855517000	-0.446592000
6	5.582357000	-0.870951000	-2.218966000
6	4.334270000	-1.161797000	-1.660336000
8	3.221805000	-0.739508000	-2.335272000
6	-6.195374000	-2.688097000	-2.862239000
6	-5.375335000	-2.702409000	-1.572216000
6	-4.293974000	-1.620617000	-1.580412000
7	-3.898598000	-1.165351000	-0.368568000
8	-3.800286000	-1.210929000	-2.639448000
1	0.987590000	8.436658000	-1.931990000
1	0.699418000	7.318085000	-3.259984000
1	-0.765842000	7.607754000	0.252586000
1	1.199699000	5.124612000	-2.647008000
1	-0.673950000	5.744670000	1.894855000
1	1.259691000	3.266830000	-1.027588000
1	0.726796000	2.599719000	1.104895000
1	7.808489000	-2.326918000	1.492769000
1	8.791232000	-1.655313000	0.178695000
1	5.376603000	-2.703207000	1.180157000
1	7.711752000	-1.007355000	-1.991349000
1	3.293141000	-2.094514000	-0.010700000
1	5.624357000	-0.327637000	-3.158197000
1	2.447528000	-0.754399000	-1.739582000
1	-1.510558000	-4.380178000	-2.116665000

1	-2.109040000	-2.855932000	-2.724921000
1	-3.003559000	-5.048281000	2.269672000
1	-2.988088000	-3.580934000	1.294187000
1	-2.356184000	-3.886576000	-5.019819000
1	-1.533488000	-5.327857000	-4.455514000
1	-3.463479000	-3.782441000	4.331621000
1	-3.287075000	-2.243021000	3.476373000
1	0.850916000	1.041299000	-2.579703000
1	0.236850000	0.756648000	-4.217962000
1	0.592012000	-4.839681000	-0.978029000
1	-1.150777000	-1.654124000	4.207877000
1	-2.304046000	0.499889000	4.677171000
1	-3.609592000	2.581313000	-2.251487000
1	0.485683000	-4.129470000	-3.728026000
1	-1.009508000	-4.116721000	3.572021000
1	-2.544342000	2.934357000	3.230358000
1	-0.715756000	3.022826000	-3.069524000
1	1.745828000	-2.978749000	-2.087201000
1	-2.061097000	0.292606000	-3.064324000
1	2.317342000	-2.159066000	-4.121877000
1	1.683817000	-0.969315000	-5.227884000
1	0.222085000	1.035549000	4.781707000
1	-0.090096000	2.364049000	3.678710000
1	-1.721059000	4.833904000	-2.326245000
1	-2.262022000	4.608680000	-0.698350000
1	-4.537676000	5.151918000	-1.228376000
1	-4.314327000	4.786948000	-2.951952000
1	1.243421000	-4.077101000	2.967899000
1	0.832098000	-5.794776000	2.938569000
1	-4.318095000	0.482071000	2.736681000
1	-4.693023000	2.149840000	2.324771000
1	-1.963263000	-1.558818000	-4.566576000
1	-1.050403000	-2.378069000	-5.857601000
1	-0.794307000	-0.662123000	-5.544811000
1	-1.230196000	-6.562935000	1.798197000
1	-1.776678000	-5.713846000	0.332325000
1	-0.157809000	-6.444621000	0.402141000
1	-4.295126000	2.507331000	5.005115000
1	-5.853974000	2.046464000	4.341449000
1	-2.990033000	1.797760000	-4.726174000
1	-1.367358000	2.257708000	-5.281145000
1	10.681667000	2.741048000	0.357035000
1	11.160167000	2.853609000	-1.340058000
1	8.814845000	3.441410000	-1.204726000
1	8.910982000	1.892808000	-2.023396000
1	8.117226000	0.703679000	-0.031564000

1	8.317036000	2.132915000	0.975587000
1	6.551872000	3.329921000	-0.238992000
1	6.356407000	2.008198000	-1.387895000
1	5.918521000	1.558302000	1.609584000
1	5.395199000	0.534979000	0.261936000
1	2.257693000	3.330792000	3.477938000
1	1.576397000	-0.561022000	1.862968000
1	1.652647000	1.000875000	2.691081000
1	4.149335000	-0.676525000	2.057675000
1	4.070410000	0.788221000	3.059229000
1	-6.811933000	-1.784051000	-2.926000000
1	-5.522217000	-2.688122000	-3.724089000
1	-6.012283000	-2.595104000	-0.687347000
1	-4.854231000	-3.662807000	-1.469241000
1	8.261408000	-3.340555000	0.116513000
1	-0.642431000	8.178881000	-2.531687000
1	-6.857231000	-3.555428000	-2.959183000
6	2.904818000	3.110880000	4.326972000
1	2.702328000	3.822399000	5.126742000
1	3.945521000	3.189219000	4.010697000
1	2.703887000	2.101304000	4.684725000
1	-5.206163000	-5.548940000	-4.307529000
1	-6.310347000	-2.502136000	1.846222000
1	-5.296294000	-0.086976000	7.034716000
1	-0.234554000	3.844576000	6.829979000
1	-2.215236000	5.706944000	-4.616294000
1	2.919879000	-3.478015000	-7.464450000
1	3.403617000	-2.091098000	-6.551895000
1	-4.251113000	-1.572610000	0.490188000
1	-3.063162000	-0.587437000	-0.315960000
1	4.079397000	-4.512917000	1.044025000
1	10.943363000	1.276086000	-0.594879000
8	-3.765142000	7.483379000	-1.386121000
1	-3.458575000	8.368660000	-1.664862000
1	-3.109031000	3.181748000	0.644379000
1	3.218603000	-3.615019000	2.292300000

D

RC

D-RC-opt.gjf.log

Temperature 298.150 Kelvin. Pressure 1.00000 Atm.

Zero-point correction= 1.543699 (Hartree/Particle)
Thermal correction to Energy= 1.645663

Thermal correction to Enthalpy= 1.646607
Thermal correction to Gibbs Free Energy= 1.385604
Sum of electronic and zero-point Energies= -5861.163979
Sum of electronic and thermal Energies= -5861.062015
Sum of electronic and thermal Enthalpies= -5861.061071
Sum of electronic and thermal Free Energies= -5861.322074

6	-1.058351000	7.387816000	1.981723000
6	-0.692112000	6.282341000	1.019031000
6	0.197750000	6.514090000	-0.041855000
6	-1.205803000	4.987921000	1.160945000
6	0.562908000	5.499686000	-0.924778000
6	-0.839298000	3.954166000	0.296446000
6	0.049495000	4.207676000	-0.755437000
8	0.449391000	3.244235000	-1.635783000
6	0.744063000	-3.812531000	3.408521000
6	2.916050000	-4.370239000	-1.901038000
6	7.813698000	1.228987000	-2.407951000
6	1.132209000	3.976900000	5.250120000
7	-0.443224000	-1.495096000	1.415609000
6	0.784076000	-4.553194000	4.751166000
6	4.267168000	-3.790241000	-2.368596000
7	0.731110000	-2.074178000	-1.243445000
8	8.063276000	0.333286000	-1.625355000
8	2.115072000	4.265022000	5.909816000
7	1.237748000	0.979527000	1.429156000
8	2.349401000	-5.612911000	6.187965000
8	6.265987000	-5.082091000	-1.847653000
8	2.702272000	-5.842504000	3.977706000
8	5.345729000	-3.777638000	-0.258252000
6	-0.789726000	0.482245000	2.770168000
6	0.144780000	-3.750254000	0.478191000
6	2.319067000	-1.304247000	-2.918531000
6	2.934023000	2.253128000	0.236465000
28	0.865115000	-0.485519000	0.037528000
6	-0.789982000	-0.996915000	2.547372000
6	-0.066144000	-3.298939000	-0.977996000
6	2.591883000	-0.052445000	-2.436954000
6	2.072619000	1.994811000	1.329782000
6	-1.191418000	-2.019244000	3.613003000
6	0.333623000	-4.329408000	-2.120286000
6	3.395064000	0.984968000	-3.200329000
6	2.078420000	2.910817000	2.555517000
6	-0.649821000	-3.342255000	2.962421000
6	1.683206000	-3.763296000	-2.617989000
6	3.978796000	1.822743000	-2.040554000

6	0.792387000	2.444345000	3.277279000
6	-0.699545000	-2.953691000	1.461711000
6	1.551914000	-2.295406000	-2.249489000
6	2.992404000	1.517272000	-0.920469000
6	0.650287000	0.997955000	2.783195000
6	-2.756858000	-2.008268000	3.651486000
6	2.478112000	1.814027000	-4.114389000
6	2.105782000	4.426228000	2.223753000
7	-1.476286000	-3.053139000	-1.254669000
6	-3.343689000	-3.210778000	4.378350000
6	-1.911070000	-3.427372000	-2.490291000
6	3.258047000	2.744613000	-5.008183000
6	3.176521000	5.254019000	2.979416000
8	-3.180616000	-3.409282000	5.581135000
8	-3.028776000	-3.201992000	-2.948563000
8	4.462934000	2.731418000	-5.185923000
6	-0.766359000	-4.121198000	-3.191712000
6	5.403116000	1.382145000	-1.647997000
7	-4.066914000	-4.062873000	3.597384000
8	2.447834000	3.626342000	-5.635466000
6	-0.623010000	-1.668481000	4.996838000
6	0.346194000	-5.796430000	-1.681774000
6	6.458229000	1.846204000	-2.653369000
6	0.778686000	2.573776000	4.812081000
6	2.038233000	-5.390727000	4.891080000
6	5.400735000	-4.300159000	-1.517860000
7	2.247492000	0.409292000	-1.194628000
8	8.764767000	1.789420000	-3.193900000
8	0.283482000	4.900473000	4.757636000
6	-10.983631000	2.318420000	-1.510060000
6	-10.542941000	2.639606000	-0.072929000
6	-9.904544000	1.450670000	0.668384000
6	-8.706392000	0.752237000	0.003068000
6	-7.350411000	1.465416000	0.053767000
16	-7.224563000	2.866469000	-1.159612000
6	-0.922077000	0.383856000	-3.079656000
8	-0.039509000	0.603860000	-5.965070000
16	-1.760145000	0.897273000	-1.530330000
6	-1.823496000	-0.168410000	-4.170350000
8	-1.825518000	-1.004772000	-6.666418000
16	-0.824151000	-0.626317000	-5.632371000
8	0.043264000	-1.759777000	-5.182705000
6	-8.486805000	-2.458929000	-1.711046000
6	-7.120552000	-2.148383000	-1.161447000
6	-6.156852000	-1.544756000	-1.979259000
6	-6.789584000	-2.376187000	0.181440000

6	-4.901341000	-1.187865000	-1.491685000
6	-5.543296000	-2.010867000	0.691772000
6	-4.592447000	-1.421968000	-0.148837000
8	-3.356962000	-1.128082000	0.379014000
6	4.624075000	-3.146064000	4.511213000
6	4.449039000	-3.079936000	2.996237000
6	3.681358000	-1.834268000	2.560257000
7	3.779949000	-1.511649000	1.246657000
8	3.014975000	-1.175096000	3.366123000
1	-1.672249000	8.159589000	1.498875000
1	-1.623321000	6.998485000	2.835117000
1	0.624061000	7.506600000	-0.172488000
1	-1.884508000	4.769478000	1.982234000
1	1.258077000	5.684546000	-1.738476000
1	-1.220032000	2.949539000	0.447091000
1	0.119513000	2.372167000	-1.336451000
1	-8.483198000	-2.415183000	-2.805805000
1	-9.220749000	-1.717520000	-1.369255000
1	-6.382830000	-1.361415000	-3.026660000
1	-7.520115000	-2.835018000	0.843973000
1	-4.153547000	-0.770743000	-2.154629000
1	-5.308363000	-2.168297000	1.739862000
1	-2.867944000	-0.527377000	-0.231762000
1	1.116269000	-4.520079000	2.663435000
1	1.448562000	-2.976147000	3.421062000
1	2.930815000	-5.452340000	-2.069778000
1	2.817805000	-4.214757000	-0.820954000
1	0.714366000	-3.890563000	5.615170000
1	-0.065429000	-5.247584000	4.830830000
1	4.468812000	-4.065384000	-3.406973000
1	4.248400000	-2.698209000	-2.296828000
1	-1.336075000	0.974721000	1.957518000
1	-1.287248000	0.730923000	3.711944000
1	1.208941000	-3.653320000	0.709053000
1	-0.134625000	-4.801923000	0.590940000
1	2.715539000	-1.572853000	-3.890671000
1	4.169970000	0.514914000	-3.811962000
1	2.957392000	2.645465000	3.164354000
1	-1.347987000	-4.168963000	3.135833000
1	1.800900000	-3.900407000	-3.698677000
1	3.983964000	2.895244000	-2.267762000
1	-0.042374000	3.029008000	2.874216000
1	-1.744258000	-3.075448000	1.149742000
1	1.262327000	0.331374000	3.404801000
1	-3.145767000	-1.935326000	2.633086000
1	-3.086169000	-1.109319000	4.184118000

1	1.855139000	1.179451000	-4.760188000
1	1.778027000	2.410927000	-3.518381000
1	1.124026000	4.837682000	2.451853000
1	2.240252000	4.582087000	1.151853000
1	3.307985000	4.878280000	3.996533000
1	-0.448019000	-3.444803000	-3.994936000
1	-1.103516000	-5.055139000	-3.651606000
1	5.430247000	0.288970000	-1.560406000
1	5.647216000	1.776619000	-0.655003000
1	0.469092000	-1.609358000	4.978058000
1	-0.941608000	-2.408542000	5.732723000
1	-1.003633000	-0.698353000	5.333529000
1	0.625512000	-6.434457000	-2.528071000
1	1.043866000	-5.998772000	-0.864936000
1	-0.654394000	-6.101229000	-1.353332000
1	6.160865000	1.611812000	-3.682726000
1	6.566979000	2.937630000	-2.635111000
1	1.503458000	1.893958000	5.268271000
1	-0.219075000	2.317645000	5.189428000
1	-10.128575000	2.332494000	-2.196086000
1	-11.712993000	3.048355000	-1.877888000
1	-9.855430000	3.495013000	-0.085805000
1	-11.414731000	2.961397000	0.512682000
1	-9.611144000	1.770627000	1.678537000
1	-10.685644000	0.688936000	0.805458000
1	-8.550238000	-0.202881000	0.519472000
1	-8.940552000	0.494145000	-1.036249000
1	-6.564828000	0.751175000	-0.203814000
1	-7.156703000	1.851125000	1.059832000
1	-5.876275000	2.934495000	-1.186178000
1	-0.201807000	-0.365893000	-2.753691000
1	-0.371495000	1.250390000	-3.453671000
1	-2.358580000	-1.066911000	-3.853313000
1	-2.545068000	0.574738000	-4.522054000
1	5.151098000	-2.259784000	4.880771000
1	3.656985000	-3.183035000	5.018205000
1	5.421169000	-3.102742000	2.488677000
1	3.891779000	-3.958416000	2.644830000
1	-8.898518000	-3.438137000	-1.433119000
1	-0.161143000	7.880313000	2.378439000
1	5.204702000	-4.029888000	4.796635000
6	-2.915488000	2.171321000	-2.143265000
1	-3.306496000	2.693412000	-1.270731000
1	-2.375722000	2.883223000	-2.767490000
1	-3.740570000	1.731885000	-2.701880000
1	3.148380000	-6.174913000	6.197477000

1	6.089005000	-4.156263000	0.248670000
1	9.602339000	1.327757000	-2.996870000
1	3.013023000	4.166777000	-6.220235000
1	0.744689000	5.775330000	4.764181000
1	-4.431830000	-4.909737000	4.013144000
1	-4.136838000	-3.958418000	2.595487000
1	4.224784000	-2.147886000	0.594958000
1	3.132307000	-0.823875000	0.867173000
1	-2.029916000	-2.409857000	-0.700868000
1	-11.426849000	1.317521000	-1.586166000
1	3.593888000	3.111070000	0.310095000
1	4.140569000	5.217390000	2.465660000
6	2.699530000	6.678457000	3.101628000
8	1.816373000	7.056289000	3.862902000
8	3.301060000	7.523483000	2.249005000
1	2.896753000	8.404770000	2.373337000

TS_1
D-TS_1-opt.gjf.log

Temperature 298.150 Kelvin. Pressure 1.00000 Atm.

Zero-point correction= 1.541060 (Hartree/Particle)
Thermal correction to Energy= 1.642589
Thermal correction to Enthalpy= 1.643533
Thermal correction to Gibbs Free Energy= 1.385307
Sum of electronic and zero-point Energies= -5861.127982
Sum of electronic and thermal Energies= -5861.026453
Sum of electronic and thermal Enthalpies= -5861.025509
Sum of electronic and thermal Free Energies= -5861.283735

6	-1.294461000	7.602445000	1.483966000
6	-0.923414000	6.431517000	0.606920000
6	-0.038323000	6.577567000	-0.472562000
6	-1.461274000	5.158787000	0.834450000
6	0.293006000	5.502322000	-1.295692000
6	-1.131686000	4.066939000	0.030641000
6	-0.252478000	4.234020000	-1.049831000
8	0.094912000	3.212733000	-1.877089000
6	1.014741000	-3.396920000	3.793620000
6	3.210657000	-4.374008000	-1.568428000
6	7.601264000	1.458356000	-2.712683000
6	1.031673000	4.400311000	4.912406000
7	-0.381852000	-1.435631000	1.597420000
6	1.138007000	-3.973261000	5.211500000
6	4.468416000	-3.827333000	-2.277908000

7	0.795544000	-2.232515000	-1.077668000
8	7.927942000	0.664072000	-1.852735000
8	2.026026000	4.755498000	5.519870000
7	1.115144000	1.164357000	1.303731000
8	2.903111000	-4.575911000	6.686124000
8	6.499613000	-5.028655000	-1.676051000
8	3.101297000	-5.250749000	4.548778000
8	5.854381000	-3.254981000	-0.444288000
6	-0.832094000	0.649406000	2.750177000
6	0.413978000	-3.704554000	0.876279000
6	2.328153000	-1.506469000	-2.821379000
6	2.705710000	2.404821000	-0.056346000
28	0.658765000	-0.407926000	-0.044783000
6	-0.750307000	-0.844417000	2.673435000
6	0.126261000	-3.477125000	-0.623373000
6	2.519147000	-0.201054000	-2.469279000
6	1.899437000	2.194183000	1.092975000
6	-1.086671000	-1.787448000	3.830074000
6	0.614163000	-4.601973000	-1.635035000
6	3.198485000	0.824667000	-3.354284000
6	1.918210000	3.186280000	2.257703000
6	-0.420170000	-3.115846000	3.319031000
6	1.883214000	-3.971260000	-2.261667000
6	3.743766000	1.815476000	-2.300682000
6	0.674005000	2.725910000	3.057027000
6	-0.505867000	-2.896772000	1.785081000
6	1.634306000	-2.487242000	-2.048084000
6	2.803857000	1.558490000	-1.130084000
6	0.579343000	1.242249000	2.673097000
6	-2.649016000	-1.904260000	3.836437000
6	2.167640000	1.484911000	-4.287497000
6	1.887293000	4.676595000	1.833634000
7	-1.302457000	-3.408842000	-0.877753000
6	-3.150301000	-3.076115000	4.670557000
6	-1.730258000	-4.011193000	-2.025691000
6	2.828860000	2.391206000	-5.295761000
6	2.960017000	5.578070000	2.495152000
8	-3.009111000	-3.134204000	5.890792000
8	-2.880167000	-3.980740000	-2.450466000
8	4.027499000	2.506260000	-5.479631000
6	-0.531337000	-4.662828000	-2.677979000
6	5.204074000	1.517508000	-1.905247000
7	-3.764230000	-4.066933000	3.964909000
8	1.917860000	3.084719000	-6.011119000
6	-0.586786000	-1.260671000	5.182813000
6	0.803091000	-5.984662000	-1.005200000

6	6.200169000	1.952601000	-2.981284000
6	0.705827000	2.961632000	4.578610000
6	2.471805000	-4.663995000	5.408471000
6	5.710442000	-4.130549000	-1.481478000
7	2.159352000	0.363875000	-1.271561000
8	8.493448000	2.002537000	-3.574069000
8	0.141595000	5.265190000	4.390100000
6	-11.010954000	1.895390000	-1.585494000
6	-10.513488000	2.280039000	-0.183694000
6	-9.734389000	1.164664000	0.536030000
6	-8.505470000	0.574883000	-0.176470000
6	-7.233472000	1.430356000	-0.214408000
16	-7.294047000	2.801352000	-1.467586000
6	-0.893423000	0.132912000	-2.906241000
8	-0.458572000	-0.104524000	-5.831767000
16	-1.310859000	0.511653000	-1.144707000
6	-1.862180000	-0.749997000	-3.675346000
8	-2.166886000	-1.927933000	-6.022680000
16	-1.067019000	-1.327144000	-5.218477000
8	-0.032185000	-2.320606000	-4.779572000
6	-8.270274000	-2.749935000	-1.669294000
6	-6.917737000	-2.395587000	-1.107474000
6	-5.970746000	-1.721805000	-1.888940000
6	-6.579818000	-2.687279000	0.221459000
6	-4.724961000	-1.362645000	-1.377593000
6	-5.342068000	-2.324904000	0.752471000
6	-4.403915000	-1.669639000	-0.052381000
8	-3.176250000	-1.384130000	0.489141000
6	4.989334000	-2.518383000	4.264842000
6	4.556967000	-2.705937000	2.809939000
6	3.779172000	-1.510427000	2.270700000
7	3.836973000	-1.335462000	0.928025000
8	3.120317000	-0.778367000	3.017100000
1	-1.933239000	8.318341000	0.948937000
1	-1.847182000	7.269547000	2.369183000
1	0.406797000	7.550925000	-0.669218000
1	-2.141215000	5.008886000	1.670256000
1	0.980363000	5.622955000	-2.128189000
1	-1.547601000	3.085834000	0.232761000
1	-0.285612000	2.364862000	-1.527270000
1	-8.257043000	-2.746019000	-2.764531000
1	-9.033391000	-2.026426000	-1.354010000
1	-6.201449000	-1.483166000	-2.924353000
1	-7.297521000	-3.204233000	0.854911000
1	-3.994799000	-0.866098000	-2.002206000
1	-5.100180000	-2.545141000	1.787839000

1	-2.652332000	-0.778152000	-0.107771000
1	1.441330000	-4.152024000	3.128340000
1	1.641905000	-2.505272000	3.692215000
1	3.285010000	-5.465451000	-1.543052000
1	3.198566000	-4.027348000	-0.528011000
1	1.011071000	-3.227860000	5.997030000
1	0.363648000	-4.736339000	5.378111000
1	4.574373000	-4.285692000	-3.264627000
1	4.391625000	-2.743894000	-2.399761000
1	-1.423740000	1.029087000	1.909437000
1	-1.321014000	0.960678000	3.676831000
1	1.464437000	-3.462276000	1.062998000
1	0.259819000	-4.758331000	1.124381000
1	2.703863000	-1.832606000	-3.783561000
1	3.988042000	0.371687000	-3.958830000
1	2.827347000	2.979612000	2.844417000
1	-1.035092000	-3.981460000	3.588809000
1	1.959289000	-4.212544000	-3.327466000
1	3.664788000	2.855977000	-2.633888000
1	-0.195983000	3.246608000	2.639837000
1	-1.532934000	-3.144222000	1.493475000
1	1.249707000	0.652267000	3.312807000
1	-3.017390000	-1.963508000	2.809251000
1	-3.064698000	-0.989204000	4.272072000
1	1.565894000	0.746912000	-4.835804000
1	1.449501000	2.078352000	-3.708410000
1	0.901852000	5.072278000	2.072616000
1	1.975276000	4.771563000	0.750388000
1	3.139270000	5.271969000	3.528188000
1	-0.315887000	-4.058460000	-3.568082000
1	-0.765703000	-5.680823000	-3.002606000
1	5.314930000	0.443510000	-1.710926000
1	5.439098000	2.026720000	-0.963721000
1	0.494842000	-1.097450000	5.176041000
1	-0.847962000	-1.961372000	5.977311000
1	-1.066819000	-0.307270000	5.426593000
1	1.131888000	-6.698982000	-1.768439000
1	1.538085000	-5.995818000	-0.195924000
1	-0.149109000	-6.347606000	-0.601333000
1	5.899638000	1.596127000	-3.974006000
1	6.229326000	3.045157000	-3.072768000
1	1.464739000	2.334764000	5.054810000
1	-0.271092000	2.703598000	5.005557000
1	-10.194940000	1.938840000	-2.316029000
1	-11.798793000	2.574563000	-1.929828000
1	-9.901541000	3.189000000	-0.252425000

1	-11.372651000	2.542271000	0.448745000
1	-9.426368000	1.524402000	1.528464000
1	-10.433442000	0.335096000	0.716821000
1	-8.224493000	-0.343470000	0.353718000
1	-8.762638000	0.264483000	-1.195950000
1	-6.390113000	0.792709000	-0.491069000
1	-7.032054000	1.862262000	0.771424000
1	-5.958260000	2.854752000	-1.696461000
1	0.075628000	-0.358218000	-2.881142000
1	-0.771729000	1.080170000	-3.434799000
1	-2.148740000	-1.647063000	-3.124341000
1	-2.768000000	-0.211967000	-3.969260000
1	5.568340000	-1.596188000	4.386090000
1	4.127210000	-2.459468000	4.933131000
1	5.423485000	-2.905137000	2.169840000
1	3.907797000	-3.588087000	2.732763000
1	-8.623592000	-3.737989000	-1.346022000
1	-0.416211000	8.155625000	1.841173000
1	5.621813000	-3.359387000	4.568841000
6	-3.524143000	1.956704000	-1.719149000
1	-3.994175000	1.730688000	-0.773615000
1	-3.019025000	2.903648000	-1.833589000
1	-3.872175000	1.439889000	-2.600620000
1	3.749187000	-5.061955000	6.736507000
1	6.659889000	-3.507305000	0.046492000
1	9.366382000	1.623084000	-3.355956000
1	2.411138000	3.626465000	-6.656589000
1	0.575431000	6.152034000	4.327014000
1	-4.077535000	-4.892874000	4.457636000
1	-3.821605000	-4.064851000	2.956974000
1	4.429885000	-1.912697000	0.344013000
1	3.302078000	-0.595170000	0.487093000
1	-1.901589000	-2.730946000	-0.416574000
1	-11.402227000	0.870633000	-1.614329000
1	3.301292000	3.310155000	-0.088831000
1	3.905268000	5.534416000	1.948099000
6	2.447008000	6.994748000	2.545909000
8	1.586519000	7.397632000	3.319650000
8	2.988710000	7.797116000	1.615893000
1	2.565243000	8.674167000	1.699808000

Int_1
D-Int_1-opt.gjf.log

Temperature 298.150 Kelvin. Pressure 1.00000 Atm.

Zero-point correction= 1.540157 (Hartree/Particle)
Thermal correction to Energy= 1.642662
Thermal correction to Enthalpy= 1.643606
Thermal correction to Gibbs Free Energy= 1.384236
Sum of electronic and zero-point Energies= -5861.133952
Sum of electronic and thermal Energies= -5861.031447
Sum of electronic and thermal Enthalpies= -5861.030502
Sum of electronic and thermal Free Energies= -5861.289873

6	-2.105859000	7.566722000	0.892473000
6	-1.607680000	6.377612000	0.107231000
6	-0.735296000	6.529479000	-0.981738000
6	-2.020181000	5.077970000	0.427834000
6	-0.296840000	5.434004000	-1.724574000
6	-1.583499000	3.967590000	-0.295547000
6	-0.718510000	4.139434000	-1.387061000
8	-0.274150000	3.099479000	-2.141170000
6	1.295721000	-2.852780000	4.164597000
6	3.847690000	-3.993248000	-1.115112000
6	6.564855000	0.504317000	-3.454889000
6	0.549806000	4.947563000	4.521120000
7	-0.170793000	-1.274869000	1.745599000
6	1.415923000	-3.269494000	5.637533000
6	4.994840000	-3.343281000	-1.913897000
7	1.126975000	-2.206772000	-0.831641000
8	6.245857000	-0.540494000	-2.919117000
8	1.502395000	5.454279000	5.086736000
7	1.020523000	1.431161000	1.224459000
8	3.171335000	-3.505625000	7.225140000
8	7.341508000	-3.810557000	-1.460008000
8	3.542282000	-4.363341000	5.178859000
8	6.180989000	-2.514120000	-0.033476000
6	-0.890963000	0.843258000	2.686042000
6	0.887678000	-3.504064000	1.266932000
6	2.557651000	-1.455085000	-2.646432000
6	2.466172000	2.714053000	-0.250803000
28	0.737879000	-0.296443000	0.019368000
6	-0.658780000	-0.636592000	2.744631000
6	0.629538000	-3.481666000	-0.255009000
6	2.598082000	-0.113406000	-2.408946000
6	1.680490000	2.521256000	0.915901000
6	-0.974191000	-1.513358000	3.959344000
6	1.308607000	-4.627595000	-1.126298000
6	3.176236000	0.895302000	-3.376138000
6	1.575190000	3.610132000	1.984941000
6	-0.135334000	-2.794161000	3.606367000

6	2.472969000	-3.882685000	-1.827301000
6	3.617348000	2.014778000	-2.408735000
6	0.381369000	3.087353000	2.822586000
6	-0.156309000	-2.721104000	2.057299000
6	2.001982000	-2.439677000	-1.773742000
6	2.672563000	1.783248000	-1.236771000
6	0.452375000	1.573094000	2.574604000
6	-2.500773000	-1.791509000	3.877868000
6	2.082071000	1.380584000	-4.345458000
6	1.382400000	5.042915000	1.427272000
7	-0.783197000	-3.643793000	-0.545915000
6	-3.023598000	-2.880389000	4.814764000
6	-1.085048000	-4.423676000	-1.622916000
6	2.644070000	2.283306000	-5.415720000
6	2.348035000	6.113194000	1.996441000
6	1.681160000	7.463134000	1.923179000
8	-2.378585000	-3.364848000	5.743487000
8	-2.212376000	-4.588575000	-2.077566000
8	3.817644000	2.573943000	-5.568085000
6	0.209218000	-4.997287000	-2.156844000
6	5.080744000	1.901013000	-1.919457000
7	-4.292546000	-3.283165000	4.529539000
8	1.673238000	2.755340000	-6.225765000
8	0.789125000	7.842068000	2.673061000
6	-0.628848000	-0.819047000	5.285789000
6	1.706922000	-5.874077000	-0.331677000
6	6.117934000	1.894031000	-3.052298000
6	0.374009000	3.459459000	4.317141000
6	2.809450000	-3.773051000	5.949914000
6	6.293343000	-3.283434000	-1.156928000
7	2.165680000	0.516281000	-1.266540000
8	7.407075000	0.545347000	-4.509952000
8	-0.423584000	5.668188000	3.932868000
6	-11.081829000	0.582856000	-1.764589000
6	-10.633528000	1.113847000	-0.395711000
6	-9.658309000	0.183741000	0.347783000
6	-8.342964000	-0.179745000	-0.361541000
6	-7.289423000	0.928741000	-0.479245000
16	-7.640807000	2.160651000	-1.825966000
6	-0.831239000	-0.228099000	-2.845146000
8	-0.376519000	-0.738905000	-5.712723000
16	-1.291282000	0.313843000	-1.138983000
6	-1.727708000	-1.270414000	-3.494075000
8	-1.921448000	-2.711070000	-5.708126000
16	-0.881802000	-1.938935000	-4.971997000
8	0.232465000	-2.787551000	-4.435560000

6	-7.848490000	-3.721491000	-1.424894000
6	-6.511639000	-3.224132000	-0.933458000
6	-5.821964000	-2.221330000	-1.630228000
6	-5.930433000	-3.720631000	0.240669000
6	-4.604463000	-1.718509000	-1.174424000
6	-4.708516000	-3.235866000	0.708766000
6	-4.044234000	-2.228279000	0.002546000
8	-2.844898000	-1.791358000	0.495433000
6	5.194701000	-1.527463000	4.586886000
6	4.787274000	-1.884893000	3.156261000
6	3.917222000	-0.807412000	2.518061000
7	3.998346000	-0.709778000	1.168388000
8	3.176319000	-0.090748000	3.199562000
1	-2.815258000	8.164976000	0.304276000
1	-2.627617000	7.243862000	1.800066000
1	-0.385842000	7.524426000	-1.250385000
1	-2.688106000	4.924269000	1.272657000
1	0.379886000	5.560261000	-2.564920000
1	-1.908575000	2.968127000	-0.026789000
1	-0.567683000	2.242869000	-1.728221000
1	-7.871227000	-3.789278000	-2.518566000
1	-8.658577000	-3.040291000	-1.130460000
1	-6.242903000	-1.823488000	-2.550912000
1	-6.435652000	-4.508187000	0.795529000
1	-4.075928000	-0.951455000	-1.729174000
1	-4.248706000	-3.650131000	1.601905000
1	-2.435724000	-1.087431000	-0.091717000
1	1.840194000	-3.611474000	3.596105000
1	1.821438000	-1.906485000	3.996621000
1	4.086909000	-5.050633000	-0.964393000
1	3.781271000	-3.534675000	-0.121712000
1	1.169632000	-2.469831000	6.336438000
1	0.722032000	-4.094061000	5.854127000
1	5.174916000	-3.889598000	-2.843359000
1	4.744792000	-2.310471000	-2.169756000
1	-1.499651000	1.085668000	1.807103000
1	-1.429530000	1.183181000	3.574163000
1	1.896028000	-3.120626000	1.451545000
1	0.847636000	-4.536057000	1.626805000
1	2.985225000	-1.817822000	-3.573130000
1	3.997182000	0.469497000	-3.953247000
1	2.495327000	3.560594000	2.588457000
1	-0.664535000	-3.691386000	3.937057000
1	2.592242000	-4.224010000	-2.861305000
1	3.471262000	3.006985000	-2.846895000
1	-0.536015000	3.470732000	2.360409000

1	-1.134661000	-3.100104000	1.746020000
1	1.166352000	1.116382000	3.272731000
1	-2.783332000	-2.041214000	2.849720000
1	-3.043489000	-0.865424000	4.112866000
1	1.560825000	0.545824000	-4.834124000
1	1.305192000	1.935907000	-3.804139000
1	0.358645000	5.348495000	1.635645000
1	1.463354000	5.047170000	0.339353000
1	3.292787000	6.124777000	1.446829000
1	2.558635000	5.923110000	3.051104000
1	0.363327000	-4.511057000	-3.126589000
1	0.120072000	-6.075267000	-2.321780000
1	5.195728000	0.989545000	-1.322191000
1	5.273523000	2.744352000	-1.248420000
1	0.419338000	-0.506575000	5.317483000
1	-0.833312000	-1.493421000	6.117912000
1	-1.245534000	0.075228000	5.426588000
1	2.164896000	-6.611544000	-1.000565000
1	2.416079000	-5.663950000	0.473625000
1	0.818920000	-6.338258000	0.112619000
1	5.733675000	2.391629000	-3.950337000
1	7.025758000	2.438077000	-2.762659000
1	1.188440000	2.958121000	4.847249000
1	-0.575436000	3.141581000	4.765252000
1	-10.277550000	0.666008000	-2.504140000
1	-11.942693000	1.142731000	-2.146877000
1	-10.189869000	2.110738000	-0.517006000
1	-11.514677000	1.252015000	0.245769000
1	-9.417216000	0.625734000	1.325388000
1	-10.186964000	-0.756922000	0.560640000
1	-7.866125000	-0.985387000	0.211436000
1	-8.540597000	-0.601593000	-1.354258000
1	-6.326748000	0.472251000	-0.719913000
1	-7.188611000	1.464162000	0.470585000
1	-6.346134000	2.464909000	-2.091330000
1	0.175580000	-0.631501000	-2.780886000
1	-0.790892000	0.656642000	-3.483144000
1	-1.935057000	-2.121851000	-2.841851000
1	-2.678650000	-0.845436000	-3.829572000
1	5.675434000	-0.543296000	4.621704000
1	4.327922000	-1.496747000	5.250955000
1	5.668149000	-2.073286000	2.533006000
1	4.209589000	-2.818537000	3.164376000
1	-8.097498000	-4.712094000	-1.022152000
1	-1.300072000	8.241630000	1.208999000
1	5.909604000	-2.264877000	4.967526000

6	-3.906784000	1.875022000	-2.130859000
1	-4.177188000	1.693404000	-1.102623000
1	-3.222412000	2.675072000	-2.365064000
1	-4.177709000	1.157432000	-2.890670000
1	4.064628000	-3.880486000	7.352123000
1	7.059236000	-2.492005000	0.391807000
1	7.659727000	-0.376437000	-4.711154000
1	2.106332000	3.316512000	-6.897337000
1	-0.083459000	6.586465000	3.791992000
1	-4.736534000	-3.962516000	5.132277000
1	-4.838387000	-2.871719000	3.785592000
1	4.632741000	-1.286921000	0.628716000
1	3.389676000	-0.069708000	0.671140000
1	-1.484596000	-3.002468000	-0.183200000
1	-11.357959000	-0.477707000	-1.709057000
8	2.119756000	8.225501000	0.909115000
1	1.600641000	9.053769000	0.915895000
1	2.958988000	3.672498000	-0.368177000

TS_2
D-TS_2-opt.gjf.log

Temperature 298.150 Kelvin. Pressure 1.00000 Atm.

Zero-point correction= 1.539573 (Hartree/Particle)
Thermal correction to Energy= 1.640729
Thermal correction to Enthalpy= 1.641674
Thermal correction to Gibbs Free Energy= 1.384595
Sum of electronic and zero-point Energies= -5861.129052
Sum of electronic and thermal Energies= -5861.027895
Sum of electronic and thermal Enthalpies= -5861.026951
Sum of electronic and thermal Free Energies= -5861.284030

6	-1.295801000	7.639348000	1.110104000
6	-0.910787000	6.438177000	0.280792000
6	-0.010060000	6.546494000	-0.790155000
6	-1.446025000	5.172093000	0.548807000
6	0.338375000	5.442190000	-1.566808000
6	-1.098600000	4.051568000	-0.207147000
6	-0.205038000	4.180956000	-1.281665000
8	0.158961000	3.130443000	-2.062218000
6	0.862834000	-3.213704000	3.964894000
6	3.250320000	-4.474505000	-1.280594000
6	7.723511000	1.352354000	-2.516803000
6	0.957613000	4.629055000	4.725585000
7	-0.416302000	-1.333396000	1.639130000

6	0.932008000	-3.722883000	5.412155000
6	4.535876000	-3.995862000	-1.989799000
7	0.855337000	-2.267555000	-0.951319000
8	8.019016000	0.609858000	-1.601122000
8	1.950449000	5.001158000	5.325269000
7	1.088921000	1.249561000	1.259709000
8	2.676576000	-4.199944000	6.958205000
8	6.472983000	-5.287505000	-1.278569000
8	2.905273000	-5.045675000	4.885713000
8	5.956751000	-3.378214000	-0.197254000
6	-0.892468000	0.803370000	2.681901000
6	0.369962000	-3.644005000	1.046333000
6	2.458514000	-1.641125000	-2.668788000
6	2.725143000	2.419587000	-0.109149000
28	0.668958000	-0.380190000	-0.019509000
6	-0.822368000	-0.692993000	2.672541000
6	0.145163000	-3.478352000	-0.471579000
6	2.638455000	-0.318752000	-2.377672000
6	1.884663000	2.264309000	1.024806000
6	-1.220537000	-1.582861000	3.851137000
6	0.650195000	-4.655877000	-1.412511000
6	3.349519000	0.660101000	-3.290580000
6	1.880660000	3.304881000	2.145715000
6	-0.550009000	-2.938644000	3.425221000
6	1.950143000	-4.073296000	-2.023808000
6	3.851910000	1.710182000	-2.273820000
6	0.619008000	2.881867000	2.937978000
6	-0.571361000	-2.783968000	1.881488000
6	1.724352000	-2.577384000	-1.878069000
6	2.868501000	1.514786000	-1.127529000
6	0.524053000	1.384171000	2.612391000
6	-2.783168000	-1.681626000	3.793470000
6	2.354382000	1.265266000	-4.296994000
6	1.862701000	4.775866000	1.657739000
7	-1.270977000	-3.394615000	-0.785591000
6	-3.333497000	-2.822321000	4.639893000
6	-1.667606000	-4.044987000	-1.918423000
6	3.053770000	2.112153000	-5.331116000
6	2.935700000	5.698017000	2.289008000
8	-3.229033000	-2.853721000	5.864840000
8	-2.799712000	-4.013585000	-2.388356000
8	4.258576000	2.216250000	-5.477303000
6	-0.459419000	-4.751353000	-2.491231000
6	5.297005000	1.442598000	-1.807040000
7	-3.944791000	-3.817796000	3.939238000
8	2.170680000	2.763213000	-6.117296000

6	-0.773595000	-1.005636000	5.201744000
6	0.796943000	-6.009472000	-0.712186000
6	6.332156000	1.822682000	-2.866917000
6	0.622970000	3.180256000	4.448969000
6	2.262604000	-4.393175000	5.686362000
6	5.752688000	-4.320608000	-1.163086000
7	2.236142000	0.307714000	-1.224753000
8	8.646511000	1.852053000	-3.372520000
8	0.079207000	5.478208000	4.159641000
6	-10.971795000	1.862658000	-1.957029000
6	-10.596140000	2.308045000	-0.533718000
6	-9.975714000	1.188200000	0.322538000
6	-8.728336000	0.478441000	-0.231960000
6	-7.392565000	1.221381000	-0.119642000
16	-7.196079000	2.616227000	-1.326660000
6	-0.738180000	-0.032199000	-2.963292000
8	-0.221564000	-0.370399000	-5.880462000
16	-1.237122000	0.451178000	-1.248376000
6	-1.719853000	-0.884090000	-3.750863000
8	-1.958993000	-2.168107000	-6.052543000
16	-0.880228000	-1.549020000	-5.233666000
8	0.116313000	-2.538727000	-4.704980000
6	-8.251630000	-2.793283000	-1.814522000
6	-6.919012000	-2.398555000	-1.230341000
6	-5.938375000	-1.796282000	-2.029285000
6	-6.631123000	-2.578275000	0.129852000
6	-4.706945000	-1.404279000	-1.506639000
6	-5.408781000	-2.180864000	0.672254000
6	-4.434055000	-1.604030000	-0.150139000
8	-3.224377000	-1.281579000	0.411238000
6	4.880644000	-2.398426000	4.388079000
6	4.440157000	-2.684760000	2.949831000
6	3.709735000	-1.513882000	2.304216000
7	3.890541000	-1.382589000	0.967473000
8	2.981748000	-0.761253000	2.960970000
1	-1.921407000	8.338573000	0.538782000
1	-1.866755000	7.337809000	1.994973000
1	0.434013000	7.513692000	-1.017438000
1	-2.136302000	5.049995000	1.380799000
1	1.037119000	5.535130000	-2.393390000
1	-1.508733000	3.075859000	0.029144000
1	-0.246452000	2.298129000	-1.698809000
1	-8.202644000	-2.838190000	-2.907898000
1	-9.028550000	-2.061733000	-1.558329000
1	-6.130377000	-1.641943000	-3.088243000
1	-7.375454000	-3.034386000	0.779040000

1	-3.952047000	-0.964770000	-2.146130000
1	-5.207295000	-2.311716000	1.731185000
1	-2.668311000	-0.733566000	-0.214440000
1	1.299584000	-4.007227000	3.352898000
1	1.507619000	-2.337805000	3.838352000
1	3.296297000	-5.564837000	-1.201657000
1	3.225642000	-4.076941000	-0.258680000
1	0.771710000	-2.943241000	6.156884000
1	0.156092000	-4.483591000	5.581707000
1	4.637297000	-4.494447000	-2.957503000
1	4.499899000	-2.915992000	-2.152461000
1	-1.461508000	1.148846000	1.811201000
1	-1.401133000	1.159072000	3.581405000
1	1.416447000	-3.410548000	1.263668000
1	0.188232000	-4.683213000	1.333837000
1	2.868445000	-2.015432000	-3.599075000
1	4.162258000	0.177624000	-3.838903000
1	2.776915000	3.122440000	2.759585000
1	-1.185444000	-3.785518000	3.706591000
1	2.050880000	-4.357388000	-3.076933000
1	3.782722000	2.730087000	-2.667289000
1	-0.240272000	3.388332000	2.482988000
1	-1.590095000	-3.028339000	1.558743000
1	1.176008000	0.815492000	3.290046000
1	-3.108975000	-1.767962000	2.753872000
1	-3.205692000	-0.749071000	4.183053000
1	1.770196000	0.497995000	-4.823544000
1	1.618246000	1.891813000	-3.778590000
1	0.878361000	5.187317000	1.872631000
1	1.957967000	4.823588000	0.571810000
1	3.104184000	5.433374000	3.335427000
1	-0.202886000	-4.198754000	-3.403793000
1	-0.702070000	-5.780850000	-2.770289000
1	5.404720000	0.381808000	-1.549133000
1	5.493969000	2.004658000	-0.887142000
1	0.309047000	-0.852405000	5.235283000
1	-1.075859000	-1.671854000	6.011208000
1	-1.253657000	-0.039049000	5.386129000
1	1.140501000	-6.763697000	-1.429078000
1	1.503930000	-5.991693000	0.121575000
1	-0.173744000	-6.340081000	-0.325000000
1	6.071802000	1.409318000	-3.848823000
1	6.360740000	2.908556000	-3.018335000
1	1.366764000	2.567525000	4.965727000
1	-0.364504000	2.949477000	4.866816000
1	-10.092705000	1.859732000	-2.611393000

1	-11.719400000	2.526171000	-2.405066000
1	-9.918952000	3.170230000	-0.585533000
1	-11.496097000	2.660515000	-0.011412000
1	-9.743238000	1.582934000	1.322260000
1	-10.749768000	0.421168000	0.471059000
1	-8.594662000	-0.454437000	0.331604000
1	-8.888344000	0.179948000	-1.274401000
1	-6.581336000	0.516381000	-0.322330000
1	-7.260498000	1.611125000	0.895511000
1	-5.774097000	2.480414000	-1.445590000
1	0.193102000	-0.583318000	-2.870449000
1	-0.527499000	0.879840000	-3.524536000
1	-2.075252000	-1.749832000	-3.189314000
1	-2.583755000	-0.308419000	-4.098546000
1	5.477540000	-1.481200000	4.442697000
1	4.024201000	-2.283699000	5.056280000
1	5.298704000	-2.974823000	2.334797000
1	3.754605000	-3.542399000	2.947568000
1	-8.616295000	-3.767589000	-1.463330000
1	-0.422723000	8.201688000	1.465680000
1	5.502637000	-3.230315000	4.735668000
6	-4.104697000	2.044752000	-1.438636000
1	-3.638006000	3.008455000	-1.284718000
1	-4.043235000	1.601704000	-2.422565000
1	-4.156855000	1.368341000	-0.597875000
1	3.522986000	-4.677625000	7.058988000
1	6.737194000	-3.649631000	0.323130000
1	9.511767000	1.490901000	-3.099643000
1	2.687699000	3.267169000	-6.774711000
1	0.521410000	6.357888000	4.062612000
1	-4.298505000	-4.621263000	4.441608000
1	-3.984570000	-3.832061000	2.930538000
1	4.530860000	-1.980411000	0.459545000
1	3.377710000	-0.675221000	0.451657000
1	-1.871222000	-2.680119000	-0.386263000
1	-11.367142000	0.839060000	-1.957115000
1	3.318466000	3.325011000	-0.169415000
1	3.885251000	5.626653000	1.752526000
6	2.431491000	7.118952000	2.279149000
8	1.556216000	7.553036000	3.018735000
8	3.001160000	7.886361000	1.336679000
1	2.583531000	8.769066000	1.382472000

Int_2

D-Int_2-opt.gjf.log

Temperature 298.150 Kelvin. Pressure 1.00000 Atm.

Zero-point correction= 1.545307 (Hartree/Particle)
Thermal correction to Energy= 1.647088
Thermal correction to Enthalpy= 1.648032
Thermal correction to Gibbs Free Energy= 1.389701
Sum of electronic and zero-point Energies= -5861.167705
Sum of electronic and thermal Energies= -5861.065923
Sum of electronic and thermal Enthalpies= -5861.064979
Sum of electronic and thermal Free Energies= -5861.323311

6	-1.256915000	7.778522000	1.159863000
6	-0.985643000	6.525624000	0.362237000
6	-0.186065000	6.553329000	-0.790498000
6	-1.539947000	5.295095000	0.736892000
6	0.046784000	5.403780000	-1.544161000
6	-1.311633000	4.131564000	0.001700000
6	-0.518429000	4.180857000	-1.154290000
8	-0.274883000	3.089366000	-1.924120000
6	1.563497000	-3.253764000	3.816809000
6	3.789654000	-3.734220000	-1.755416000
6	6.746569000	0.919039000	-3.594780000
6	1.218604000	4.452741000	4.865996000
7	0.043829000	-1.460300000	1.652199000
6	1.756845000	-3.859214000	5.216134000
6	4.791117000	-2.879104000	-2.543977000
7	0.981758000	-2.214307000	-1.140498000
8	7.934205000	0.956084000	-3.340317000
8	2.289377000	4.850703000	5.289237000
7	1.192947000	1.247390000	1.264777000
8	3.628110000	-3.975233000	6.685775000
8	7.165718000	-2.431784000	-2.572180000
8	3.928654000	-4.758232000	4.597846000
8	6.176210000	-2.806443000	-0.591896000
6	-0.575198000	0.555853000	2.863482000
6	1.014844000	-3.653912000	0.881463000
6	2.168078000	-1.255034000	-3.033908000
6	2.494829000	2.641280000	-0.240501000
28	0.727185000	-0.346682000	-0.045300000
6	-0.385154000	-0.926374000	2.737869000
6	0.636416000	-3.559984000	-0.612075000
6	2.249590000	0.057897000	-2.675921000
6	1.842433000	2.352309000	0.985699000
6	-0.720773000	-1.939962000	3.834190000
6	1.331652000	-4.596826000	-1.604248000
6	2.757467000	1.140630000	-3.602742000

6	1.877027000	3.335760000	2.154607000
6	0.107616000	-3.171669000	3.324936000
6	2.357257000	-3.715199000	-2.356113000
6	3.341649000	2.144492000	-2.584907000
6	0.753579000	2.764343000	3.054965000
6	0.026501000	-2.934170000	1.795717000
6	1.767486000	-2.325803000	-2.176779000
6	2.534488000	1.815689000	-1.335363000
6	0.762591000	1.276495000	2.672585000
6	-2.255359000	-2.162882000	3.678637000
6	1.591901000	1.730595000	-4.414809000
6	1.681474000	4.821665000	1.761103000
7	-0.777931000	-3.829627000	-0.802893000
6	-2.824534000	-3.407147000	4.355049000
6	-1.099716000	-4.613429000	-1.867728000
6	2.071298000	2.718976000	-5.449656000
6	2.795605000	5.786350000	2.233739000
8	-2.287946000	-3.981612000	5.302661000
8	-2.242840000	-4.912695000	-2.204912000
8	3.232463000	2.988182000	-5.699669000
6	0.183569000	-5.012040000	-2.559544000
6	4.834353000	1.880057000	-2.285119000
7	-4.004719000	-3.820368000	3.821473000
8	1.038667000	3.297871000	-6.097471000
6	-0.395315000	-1.446976000	5.250224000
6	1.904986000	-5.840230000	-0.918605000
6	5.782247000	2.078270000	-3.502223000
6	0.909275000	3.002763000	4.570984000
6	3.203667000	-4.249727000	5.432287000
6	6.158543000	-2.707979000	-1.941516000
7	1.978008000	0.572658000	-1.431547000
8	6.107541000	-0.220708000	-3.950202000
8	0.212358000	5.278558000	4.521183000
6	-11.168030000	1.682245000	0.155966000
6	-9.872547000	1.950447000	0.928484000
6	-8.771487000	0.908703000	0.686278000
6	-8.269369000	0.742544000	-0.757944000
6	-7.417401000	1.911646000	-1.275684000
16	-8.312032000	3.430721000	-1.728496000
6	-1.293305000	-0.097163000	-2.585905000
8	-1.648666000	-0.544531000	-5.510225000
16	-1.439820000	0.407494000	-0.810310000
6	-2.280063000	-1.168640000	-3.015847000
8	-3.023974000	-2.600418000	-5.111218000
16	-1.855563000	-1.773224000	-4.682974000
8	-0.597354000	-2.567252000	-4.498873000

6	-8.358384000	-2.921801000	0.238699000
6	-6.865575000	-2.694008000	0.291103000
6	-5.995695000	-3.493346000	-0.467808000
6	-6.306099000	-1.683799000	1.083639000
6	-4.617132000	-3.290427000	-0.453929000
6	-4.925479000	-1.460299000	1.107728000
6	-4.083393000	-2.260802000	0.329449000
8	-2.717790000	-2.097667000	0.373931000
6	5.579083000	-1.896688000	4.211089000
6	5.084365000	-2.147305000	2.785894000
6	4.139786000	-1.049417000	2.308691000
7	4.053097000	-0.886412000	0.962978000
8	3.478073000	-0.377216000	3.104139000
1	-1.982989000	8.428716000	0.652942000
1	-1.665794000	7.537408000	2.146806000
1	0.269987000	7.491339000	-1.100803000
1	-2.153863000	5.238341000	1.633437000
1	0.669307000	5.430805000	-2.434046000
1	-1.743092000	3.184944000	0.311675000
1	-0.604886000	2.271137000	-1.465738000
1	-8.715267000	-2.968431000	-0.796889000
1	-8.905911000	-2.122788000	0.745545000
1	-6.407472000	-4.281383000	-1.094617000
1	-6.953920000	-1.056416000	1.689003000
1	-3.946557000	-3.900645000	-1.053917000
1	-4.498197000	-0.669584000	1.718287000
1	-2.412038000	-1.192054000	0.050924000
1	2.112361000	-3.907139000	3.133294000
1	2.051075000	-2.273721000	3.760843000
1	4.150533000	-4.767334000	-1.735528000
1	3.746971000	-3.393611000	-0.715677000
1	1.442357000	-3.197389000	6.022673000
1	1.161585000	-4.778515000	5.307577000
1	4.931786000	-3.262658000	-3.559330000
1	4.412219000	-1.855896000	-2.664022000
1	-1.280938000	0.900401000	2.097910000
1	-0.992446000	0.804845000	3.842237000
1	2.029369000	-3.263619000	1.012032000
1	1.014996000	-4.706011000	1.181211000
1	2.502532000	-1.530346000	-4.027789000
1	3.511588000	0.747246000	-4.287487000
1	2.846396000	3.203026000	2.660268000
1	-0.399808000	-4.104683000	3.584774000
1	2.422121000	-3.994595000	-3.413687000
1	3.203890000	3.184865000	-2.894694000
1	-0.193838000	3.206651000	2.721081000

1	-0.974731000	-3.250585000	1.493373000
1	1.532648000	0.753734000	3.253107000
1	-2.522805000	-2.183645000	2.618802000
1	-2.777883000	-1.294110000	4.101567000
1	1.022755000	0.944358000	-4.927662000
1	0.877434000	2.238051000	-3.755865000
1	0.733450000	5.157191000	2.176647000
1	1.565637000	4.919547000	0.680492000
1	3.172848000	5.479348000	3.213060000
1	0.189121000	-4.441392000	-3.494742000
1	0.189282000	-6.077988000	-2.804560000
1	4.916416000	0.849789000	-1.917490000
1	5.151648000	2.526112000	-1.460662000
1	0.653483000	-1.151169000	5.349105000
1	-0.622489000	-2.235338000	5.969736000
1	-1.009991000	-0.578251000	5.509525000
1	2.371347000	-6.495222000	-1.663233000
1	2.655142000	-5.605863000	-0.158393000
1	1.102522000	-6.409566000	-0.435857000
1	5.201207000	2.141332000	-4.427718000
1	6.362271000	2.998022000	-3.403632000
1	1.733067000	2.405176000	4.970566000
1	-0.014598000	2.709693000	5.083180000
1	-11.021324000	1.824312000	-0.920331000
1	-11.961182000	2.370269000	0.471165000
1	-9.504722000	2.957781000	0.679166000
1	-10.085379000	1.974024000	2.006017000
1	-7.910356000	1.137825000	1.329886000
1	-9.154675000	-0.063018000	1.024423000
1	-7.629270000	-0.147721000	-0.794374000
1	-9.103188000	0.559054000	-1.445068000
1	-6.929877000	1.610037000	-2.216442000
1	-6.614079000	2.153184000	-0.570430000
1	-5.825084000	-1.409223000	-2.443097000
1	-0.280410000	-0.464989000	-2.735819000
1	-1.421461000	0.790548000	-3.209365000
1	-2.256881000	-2.039647000	-2.358314000
1	-3.302168000	-0.785049000	-3.050594000
1	6.108324000	-0.939887000	4.280600000
1	4.742473000	-1.865452000	4.913279000
1	5.923871000	-2.249377000	2.088603000
1	4.534129000	-3.096612000	2.751584000
1	-8.641415000	-3.866544000	0.721315000
1	-0.351023000	8.376133000	1.324662000
1	6.266358000	-2.691229000	4.521077000
6	-5.940156000	-1.193854000	-3.505034000

1	-6.948325000	-1.465647000	-3.821971000
1	-5.201625000	-1.761966000	-4.071629000
1	-5.781989000	-0.128574000	-3.681260000
1	4.558532000	-4.268409000	6.742829000
1	7.089614000	-2.616693000	-0.299126000
1	6.717326000	-0.979670000	-3.807231000
1	1.418196000	3.910207000	-6.756763000
1	0.594373000	6.182454000	4.393186000
1	-4.470021000	-4.622252000	4.224291000
1	-4.425994000	-3.368660000	3.019280000
1	4.641581000	-1.408140000	0.327158000
1	3.419280000	-0.194196000	0.580642000
1	-1.503758000	-3.254355000	-0.373248000
1	-11.535807000	0.658749000	0.301845000
1	3.002550000	3.595447000	-0.323840000
1	3.630718000	5.811243000	1.529296000
6	2.222904000	7.169602000	2.418081000
8	1.408850000	7.478892000	3.279284000
8	2.665279000	8.062375000	1.517146000
1	2.230009000	8.916187000	1.710009000

Int_3
D-Int_3-opt.gjf.log

Temperature 298.150 Kelvin. Pressure 1.00000 Atm.

Zero-point correction= 1.545738 (Hartree/Particle)
Thermal correction to Energy= 1.647563
Thermal correction to Enthalpy= 1.648508
Thermal correction to Gibbs Free Energy= 1.393414
Sum of electronic and zero-point Energies= -5861.181337
Sum of electronic and thermal Energies= -5861.079512
Sum of electronic and thermal Enthalpies= -5861.078568
Sum of electronic and thermal Free Energies= -5861.333661

6	0.127726000	8.048388000	-0.011225000
6	0.163893000	6.663247000	-0.606861000
6	1.012142000	6.349169000	-1.678921000
6	-0.674620000	5.651043000	-0.121860000
6	1.020466000	5.077902000	-2.251510000
6	-0.675219000	4.370309000	-0.674700000
6	0.170796000	4.080541000	-1.754524000
8	0.199520000	2.861260000	-2.353792000
6	0.689340000	-2.871251000	4.258505000
6	2.980909000	-4.432556000	-1.089432000
6	6.759055000	-0.572614000	-3.274941000

6	1.591635000	4.795973000	4.392694000
7	-0.478398000	-1.158291000	1.798387000
6	0.738687000	-3.292466000	5.735692000
6	4.141577000	-3.874434000	-1.924662000
7	0.479226000	-2.387630000	-0.796332000
8	7.929688000	-0.713906000	-2.981259000
8	2.704073000	5.037059000	4.826930000
7	1.132883000	1.253995000	1.156147000
8	2.514215000	-3.537762000	7.304143000
8	6.556028000	-3.819514000	-1.874076000
8	2.728995000	-4.633813000	5.350985000
8	5.450211000	-3.742794000	0.078261000
6	-0.798494000	1.073872000	2.701034000
6	0.167276000	-3.552788000	1.373648000
6	1.875490000	-1.894354000	-2.725764000
6	2.730596000	2.218072000	-0.401137000
28	0.491432000	-0.407502000	0.028321000
6	-0.845115000	-0.421653000	2.783178000
6	-0.128411000	-3.576616000	-0.140343000
6	2.165846000	-0.579256000	-2.526334000
6	1.983200000	2.190728000	0.802595000
6	-1.340774000	-1.207398000	3.999045000
6	0.404773000	-4.836101000	-0.960753000
6	2.892005000	0.274527000	-3.541215000
6	2.144595000	3.280333000	1.861468000
6	-0.716592000	-2.614450000	3.690030000
6	1.591553000	-4.243630000	-1.760589000
6	3.581119000	1.302410000	-2.616429000
6	0.897545000	3.024798000	2.742910000
6	-0.721875000	-2.578791000	2.139782000
6	1.258460000	-2.762461000	-1.774652000
6	2.677046000	1.268726000	-1.390547000
6	0.653912000	1.525192000	2.522912000
6	-2.887436000	-1.213002000	3.839690000
6	1.885685000	0.918908000	-4.510017000
6	2.244835000	4.722910000	1.305173000
7	-1.558451000	-3.603710000	-0.393835000
6	-3.654511000	-2.198405000	4.718000000
6	-1.972128000	-4.433582000	-1.387079000
6	2.577687000	1.656668000	-5.627664000
6	3.501760000	5.515633000	1.736663000
8	-3.185123000	-2.749871000	5.713309000
8	-3.131450000	-4.526619000	-1.788304000
8	3.778651000	1.692161000	-5.830848000
6	-0.769146000	-5.179705000	-1.914842000
6	4.998829000	0.858072000	-2.189864000

7	-4.934484000	-2.400947000	4.304856000
8	1.693942000	2.302598000	-6.417741000
6	-0.944143000	-0.567690000	5.336932000
6	0.737734000	-6.057371000	-0.098397000
6	6.017258000	0.740798000	-3.359963000
6	1.027802000	3.402808000	4.233055000
6	2.084112000	-3.898188000	6.074568000
6	5.495682000	-3.839478000	-1.271263000
7	1.924515000	0.130543000	-1.373284000
8	5.934402000	-1.618352000	-3.520996000
8	0.768352000	5.746572000	3.912111000
6	-10.547369000	3.741844000	-0.850518000
6	-9.074076000	3.693621000	-0.402729000
6	-8.173749000	2.699162000	-1.162608000
6	-6.689151000	2.856077000	-0.770831000
6	-5.722369000	1.957333000	-1.568098000
16	-3.963735000	2.432299000	-1.256308000
6	-1.295624000	-0.079673000	-2.731019000
8	-1.012846000	-0.885019000	-5.502742000
16	-1.518193000	0.583614000	-1.022503000
6	-2.313953000	-1.108600000	-3.196321000
8	-2.846957000	-2.577459000	-5.344180000
16	-1.661574000	-1.956984000	-4.685084000
8	-0.677604000	-2.958786000	-4.162276000
6	-8.828795000	-1.201354000	0.008092000
6	-7.330839000	-1.236799000	0.150402000
6	-6.577238000	-2.175379000	-0.568991000
6	-6.658560000	-0.356296000	1.006968000
6	-5.190652000	-2.237827000	-0.451193000
6	-5.268076000	-0.403404000	1.136970000
6	-4.539984000	-1.342304000	0.400562000
8	-3.171456000	-1.428049000	0.551437000
6	4.886353000	-2.191079000	4.697200000
6	4.371492000	-2.530942000	3.297463000
6	3.655078000	-1.346187000	2.654893000
7	3.663902000	-1.311701000	1.297203000
8	3.080165000	-0.491558000	3.335135000
1	-0.434194000	8.743314000	-0.650569000
1	-0.360809000	8.042079000	0.969020000
1	1.684293000	7.110842000	-2.068737000
1	-1.334425000	5.860968000	0.717253000
1	1.680740000	4.836659000	-3.079667000
1	-1.327067000	3.596118000	-0.281143000
1	-0.316416000	2.210793000	-1.815133000
1	-9.134596000	-1.336570000	-1.035542000
1	-9.245913000	-0.251821000	0.360665000

1	-7.084801000	-2.868660000	-1.235311000
1	-7.223070000	0.386812000	1.565078000
1	-4.606028000	-2.968694000	-1.003482000
1	-4.740819000	0.297996000	1.776425000
1	-2.696934000	-0.695654000	0.055517000
1	1.130978000	-3.700456000	3.699016000
1	1.342280000	-2.007042000	4.093351000
1	3.151470000	-5.500682000	-0.923487000
1	2.970159000	-3.956079000	-0.103508000
1	0.533241000	-2.475279000	6.426491000
1	-0.016996000	-4.067352000	5.926303000
1	4.252524000	-4.416567000	-2.868876000
1	3.947554000	-2.830496000	-2.202687000
1	-1.390738000	1.419454000	1.845337000
1	-1.219900000	1.521261000	3.604317000
1	1.227228000	-3.319699000	1.517101000
1	-0.020342000	-4.546743000	1.789677000
1	2.179284000	-2.345782000	-3.662442000
1	3.607254000	-0.323039000	-4.109976000
1	3.049255000	3.034258000	2.439442000
1	-1.381706000	-3.407012000	4.043175000
1	1.634685000	-4.656082000	-2.774215000
1	3.623596000	2.302217000	-3.059373000
1	0.061262000	3.585151000	2.305290000
1	-1.750926000	-2.784245000	1.835680000
1	1.292303000	0.949309000	3.205281000
1	-3.150147000	-1.398609000	2.794913000
1	-3.267418000	-0.207275000	4.065763000
1	1.211083000	0.171544000	-4.950565000
1	1.237604000	1.625921000	-3.979800000
1	1.361722000	5.270360000	1.629200000
1	2.189303000	4.716536000	0.215605000
1	3.783590000	5.250272000	2.759614000
1	-0.612086000	-4.796674000	-2.928134000
1	-0.971777000	-6.252984000	-1.977575000
1	4.900934000	-0.111872000	-1.687071000
1	5.378999000	1.556380000	-1.437772000
1	0.138374000	-0.430229000	5.418520000
1	-1.296382000	-1.193687000	6.158052000
1	-1.412344000	0.415972000	5.449925000
1	1.106472000	-6.870953000	-0.733236000
1	1.494882000	-5.854606000	0.663816000
1	-0.163446000	-6.419381000	0.409329000
1	5.496108000	0.798757000	-4.321038000
1	6.752185000	1.547463000	-3.324671000
1	1.706065000	2.714748000	4.744792000

1	0.043424000	3.343476000	4.711485000
1	-10.620692000	3.886935000	-1.935671000
1	-11.064551000	4.580494000	-0.368910000
1	-8.646677000	4.699407000	-0.524238000
1	-9.023986000	3.474661000	0.673462000
1	-8.502683000	1.667559000	-0.975666000
1	-8.281083000	2.868605000	-2.243829000
1	-6.405121000	3.907211000	-0.920620000
1	-6.562380000	2.649449000	0.299841000
1	-5.924272000	2.036176000	-2.641891000
1	-5.854580000	0.909415000	-1.277714000
1	-6.226954000	-2.880494000	-3.494361000
1	-0.304773000	-0.530083000	-2.745756000
1	-1.281172000	0.763945000	-3.423264000
1	-2.497824000	-1.887015000	-2.453042000
1	-3.266690000	-0.644020000	-3.465685000
1	5.592114000	-1.353404000	4.661131000
1	4.059931000	-1.898047000	5.349678000
1	5.183829000	-2.877547000	2.648803000
1	3.650925000	-3.356219000	3.363753000
1	-9.303954000	-2.002346000	0.589676000
1	1.123433000	8.487416000	0.132898000
1	5.399072000	-3.046206000	5.150604000
6	-5.702314000	-3.716366000	-3.958955000
1	-5.190329000	-4.291383000	-3.188413000
1	-4.952275000	-3.332863000	-4.648914000
1	-6.421024000	-4.347044000	-4.485715000
1	3.373448000	-3.980564000	7.447295000
1	6.372342000	-3.661562000	0.393152000
1	6.401318000	-2.449130000	-3.277009000
1	2.206484000	2.741632000	-7.123511000
1	1.316806000	6.545238000	3.710124000
1	-5.519110000	-3.043794000	4.821269000
1	-5.294121000	-2.004129000	3.445814000
1	4.177031000	-1.991266000	0.751316000
1	3.184691000	-0.560850000	0.813744000
1	-2.182214000	-2.862440000	-0.068918000
1	-11.107659000	2.832980000	-0.597273000
1	3.396456000	3.058646000	-0.558442000
1	4.349992000	5.314152000	1.077834000
6	3.195019000	6.992832000	1.758912000
8	2.404552000	7.530871000	2.523835000
8	3.861148000	7.697431000	0.828771000
1	3.593713000	8.633196000	0.921071000

TS_3

D-TS_3-opt.gjf.log

Temperature 298.150 Kelvin. Pressure 1.00000 Atm.

Zero-point correction= 1.546568 (Hartree/Particle)
Thermal correction to Energy= 1.647603
Thermal correction to Enthalpy= 1.648547
Thermal correction to Gibbs Free Energy= 1.396155
Sum of electronic and zero-point Energies= -5861.170563
Sum of electronic and thermal Energies= -5861.069528
Sum of electronic and thermal Enthalpies= -5861.068584
Sum of electronic and thermal Free Energies= -5861.320976

6	0.298442000	7.335189000	-3.176599000
6	0.296993000	6.296375000	-2.086481000
6	-0.047546000	6.598215000	-0.760021000
6	0.736128000	4.995481000	-2.362065000
6	0.065706000	5.648134000	0.255580000
6	0.839269000	4.028433000	-1.364323000
6	0.524026000	4.358290000	-0.041108000
8	0.652372000	3.465550000	0.983474000
6	-2.625162000	-3.719536000	-2.765699000
6	-2.134501000	-3.672234000	3.138827000
6	-3.865065000	1.023626000	5.908207000
6	-3.077915000	3.609016000	-4.853586000
7	-0.249581000	-1.728703000	-1.705645000
6	-3.411136000	-4.416129000	-3.889037000
6	-2.744501000	-2.858663000	4.296899000
7	0.078662000	-2.165995000	1.237197000
8	-3.514477000	-0.125660000	5.713648000
8	-4.231261000	3.891620000	-5.125421000
7	-1.426954000	0.914473000	-1.136508000
8	-5.747592000	-4.681644000	-4.275760000
8	-4.981957000	-3.329781000	5.136464000
8	-4.958246000	-5.408627000	-2.298689000
8	-4.747401000	-2.209699000	3.199612000
6	-0.325796000	0.163728000	-3.215197000
6	-0.792338000	-3.810290000	-0.401862000
6	-0.325935000	-1.042337000	3.365007000
6	-2.302941000	2.341307000	0.617169000
28	-0.403859000	-0.497362000	0.017671000
6	-0.382677000	-1.299715000	-2.909265000
6	0.213069000	-3.567659000	0.741073000
6	-0.721612000	0.200461000	2.967869000
6	-2.097031000	1.971218000	-0.734154000
6	-0.677642000	-2.382330000	-3.950364000
6	0.086112000	-4.484501000	2.034112000
6	-1.067095000	1.306521000	3.943398000

6	-2.712997000	2.775538000	-1.883569000
6	-1.117946000	-3.565165000	-3.015578000
6	-0.588017000	-3.553041000	3.058486000
6	-2.124655000	2.090812000	3.135763000
6	-1.912181000	2.214277000	-3.086148000
6	-0.331186000	-3.209685000	-1.726165000
6	-0.210541000	-2.181945000	2.515626000
6	-1.783456000	1.686625000	1.706088000
6	-1.575708000	0.800372000	-2.597148000
6	0.675702000	-2.688121000	-4.646037000
6	0.160200000	2.161602000	4.294129000
6	-2.630187000	4.311818000	-1.712032000
7	1.563481000	-3.865235000	0.292145000
6	0.639377000	-3.831050000	-5.663960000
6	2.351226000	-4.574255000	1.154236000
6	-0.136584000	3.104594000	5.434119000
6	-3.920793000	5.096185000	-2.063827000
6	-3.535562000	6.493241000	-2.479993000
8	-0.315966000	-4.589988000	-5.819815000
8	3.485086000	-4.970779000	0.920303000
8	-1.176514000	3.160299000	6.066345000
6	1.562431000	-4.726497000	2.435784000
6	-3.588683000	1.704378000	3.451775000
7	1.775060000	-3.941559000	-6.408066000
8	0.910974000	3.913211000	5.705583000
8	-3.097194000	6.800830000	-3.582000000
6	-1.705729000	-1.914423000	-4.994063000
6	-0.601545000	-5.829668000	1.788990000
6	-4.058223000	2.096640000	4.859859000
6	-2.607718000	2.226993000	-4.458026000
6	-4.755013000	-4.890878000	-3.380969000
6	-4.248411000	-2.854295000	4.298041000
7	-0.998510000	0.570876000	1.674856000
8	-4.170542000	1.481787000	7.140908000
8	-2.089626000	4.521787000	-4.804998000
6	10.487004000	1.756815000	-1.962622000
6	8.998572000	1.841703000	-2.364524000
6	7.978367000	1.366355000	-1.308345000
6	6.530139000	1.799067000	-1.644296000
6	5.499601000	1.264936000	-0.631224000
16	3.857343000	2.123048000	-0.806142000
6	2.320919000	0.519423000	1.652486000
8	3.042004000	0.328232000	4.682686000
16	2.082300000	0.747325000	-0.168514000
6	3.758179000	0.441303000	2.133530000
8	5.292309000	-0.552577000	4.021222000

16	3.838214000	-0.478996000	3.701622000
8	3.224609000	-1.810080000	3.391259000
6	7.919327000	-2.507295000	0.109488000
6	6.531280000	-2.266207000	-0.414616000
6	5.444737000	-2.198838000	0.460156000
6	6.287109000	-2.201439000	-1.794631000
6	4.145273000	-2.074350000	-0.021714000
6	4.989800000	-2.076594000	-2.290823000
6	3.919310000	-2.017945000	-1.397345000
8	2.636526000	-1.935329000	-1.906680000
6	-6.542089000	-2.627437000	-1.280535000
6	-5.358990000	-2.690468000	-0.316114000
6	-4.407700000	-1.512253000	-0.522416000
7	-3.716846000	-1.109804000	0.569359000
8	-4.255101000	-0.990256000	-1.633722000
1	1.039520000	8.117428000	-2.959448000
1	0.593192000	6.882321000	-4.130195000
1	-0.400739000	7.597082000	-0.512817000
1	0.995952000	4.724745000	-3.382595000
1	-0.194265000	5.889348000	1.282246000
1	1.167697000	3.024707000	-1.606344000
1	0.883737000	2.588135000	0.609082000
1	7.997594000	-2.296823000	1.180070000
1	8.676393000	-1.916784000	-0.419391000
1	5.602170000	-2.235032000	1.535053000
1	7.123396000	-2.246958000	-2.488878000
1	3.320102000	-2.020224000	0.676859000
1	4.794283000	-2.013600000	-3.357313000
1	2.133786000	-1.289322000	-1.362435000
1	-2.754380000	-4.346539000	-1.879148000
1	-3.087847000	-2.755931000	-2.533145000
1	-2.400673000	-4.724489000	3.280352000
1	-2.574743000	-3.356144000	2.186382000
1	-3.565674000	-3.783128000	-4.762059000
1	-2.862063000	-5.303191000	-4.232712000
1	-2.413283000	-3.255781000	5.259891000
1	-2.434503000	-1.813782000	4.235045000
1	0.563926000	0.616889000	-2.765133000
1	-0.292834000	0.338800000	-4.293233000
1	-1.764154000	-3.402724000	-0.106790000
1	-0.905067000	-4.886868000	-0.559379000
1	-0.229672000	-1.221584000	4.429940000
1	-1.470531000	0.885500000	4.863050000
1	-3.765360000	2.459719000	-1.968261000
1	-0.743052000	-4.509905000	-3.415564000
1	-0.182947000	-3.715511000	4.064323000

1	-2.015609000	3.173284000	3.266799000
1	-0.981088000	2.787634000	-3.160034000
1	0.689709000	-3.558548000	-1.902874000
1	-2.439606000	0.144847000	-2.779737000
1	1.444161000	-2.922324000	-3.898748000
1	1.031948000	-1.782892000	-5.155305000
1	1.019982000	1.536632000	4.572718000
1	0.485321000	2.756661000	3.432317000
1	-1.819074000	4.673635000	-2.340696000
1	-2.341452000	4.572494000	-0.692909000
1	-4.603326000	5.128668000	-1.210517000
1	-4.442020000	4.642279000	-2.908413000
1	1.937607000	-3.945365000	3.110410000
1	1.733175000	-5.698847000	2.904834000
1	-3.724285000	0.625512000	3.310286000
1	-4.228804000	2.201716000	2.715363000
1	-2.642439000	-1.596832000	-4.527090000
1	-1.911882000	-2.720249000	-5.699024000
1	-1.315883000	-1.066865000	-5.567824000
1	-0.648403000	-6.401171000	2.722811000
1	-1.620155000	-5.725717000	1.405982000
1	-0.029018000	-6.422284000	1.065999000
1	-3.551802000	2.998881000	5.216999000
1	-5.132289000	2.324692000	4.857820000
1	-3.482107000	1.570214000	-4.462337000
1	-1.906588000	1.861891000	-5.219510000
1	10.662935000	2.307562000	-1.029725000
1	11.111353000	2.217352000	-2.737986000
1	8.779613000	2.892858000	-2.602766000
1	8.834453000	1.283603000	-3.297035000
1	8.020078000	0.273700000	-1.208228000
1	8.249355000	1.778853000	-0.325902000
1	6.496645000	2.897397000	-1.671694000
1	6.258625000	1.451635000	-2.649949000
1	5.855982000	1.427474000	0.389984000
1	5.351004000	0.196482000	-0.775413000
1	2.659551000	3.724453000	2.579197000
1	1.816810000	-0.426230000	1.851737000
1	1.786225000	1.307238000	2.179653000
1	4.391120000	-0.107079000	1.442770000
1	4.191322000	1.428620000	2.297083000
1	-7.110488000	-1.701005000	-1.134653000
1	-6.187752000	-2.630362000	-2.314649000
1	-5.690177000	-2.733164000	0.727432000
1	-4.784202000	-3.607449000	-0.500034000
1	8.151483000	-3.569074000	-0.052186000

1	-0.647765000	7.857496000	-3.368303000
1	-7.238340000	-3.463741000	-1.155747000
6	3.347371000	3.742203000	3.424840000
1	3.075644000	4.559700000	4.091853000
1	4.365761000	3.883022000	3.059417000
1	3.277853000	2.799634000	3.967369000
1	-6.569735000	-5.023083000	-3.872850000
1	-5.720590000	-2.217302000	3.274874000
1	-4.039448000	0.739646000	7.762098000
1	0.650671000	4.477536000	6.458811000
1	-2.499810000	5.417855000	-4.721515000
1	1.876786000	-4.751377000	-7.005226000
1	2.607213000	-3.412687000	-6.187445000
1	-3.903813000	-1.477701000	1.494472000
1	-3.024793000	-0.376926000	0.468896000
1	1.954967000	-3.477980000	-0.556557000
1	10.864311000	0.738036000	-1.813923000
8	-3.660091000	7.397333000	-1.494945000
1	-3.332298000	8.252255000	-1.837057000
1	-2.911899000	3.216552000	0.812712000

PC
D-PC-opt.gjf.log
Temperature 298.150 Kelvin. Pressure 1.00000 Atm.

Zero-point correction= 1.545376 (Hartree/Particle)
Thermal correction to Energy= 1.647044
Thermal correction to Enthalpy= 1.647989
Thermal correction to Gibbs Free Energy= 1.394069
Sum of electronic and zero-point Energies= -5861.196741
Sum of electronic and thermal Energies= -5861.095073
Sum of electronic and thermal Enthalpies= -5861.094129
Sum of electronic and thermal Free Energies= -5861.348048

6	-1.011655000	7.593820000	-1.866506000
6	-0.578333000	6.152684000	-1.991291000
6	0.532158000	5.778231000	-2.761711000
6	-1.318042000	5.131536000	-1.377306000
6	0.891627000	4.439450000	-2.916865000
6	-0.970491000	3.786458000	-1.516082000
6	0.141066000	3.436798000	-2.291136000
8	0.531879000	2.144361000	-2.485522000
6	0.645576000	-1.830002000	4.296808000
6	2.551434000	-4.938187000	-0.352459000
6	6.091495000	-1.457918000	-1.092757000
6	-0.055678000	5.693981000	3.364434000

7	-0.948515000	-0.947976000	1.768364000
6	0.989066000	-1.813558000	5.794361000
6	3.916028000	-4.565983000	-0.937538000
7	0.511391000	-2.314540000	-0.376491000
8	5.422704000	-1.680401000	-0.089559000
8	0.736104000	6.318398000	4.047534000
7	0.773437000	1.447730000	1.125716000
8	2.965343000	-1.179575000	6.967381000
8	6.274941000	-4.795054000	-0.490516000
8	3.189311000	-2.746331000	5.367575000
8	4.856603000	-6.072095000	0.677412000
6	-1.449560000	1.368972000	2.236740000
6	-0.148182000	-3.335358000	1.753780000
6	2.272100000	-2.160770000	-2.034641000
6	2.670703000	2.257449000	-0.165612000
28	0.573829000	-0.408646000	0.301131000
6	-1.368777000	-0.074922000	2.613271000
6	-0.356863000	-3.350295000	0.223711000
6	2.619053000	-0.845765000	-1.889433000
6	1.632870000	2.393833000	0.781252000
6	-1.677699000	-0.632909000	4.007922000
6	-0.028439000	-4.696747000	-0.548797000
6	3.619796000	-0.154749000	-2.793078000
6	1.428363000	3.711217000	1.535080000
6	-0.845889000	-1.964650000	3.934362000
6	1.357152000	-4.392720000	-1.175488000
6	4.045498000	1.064724000	-1.940075000
6	0.023887000	3.482857000	2.145042000
6	-1.019142000	-2.282062000	2.426902000
6	1.362486000	-2.872691000	-1.208594000
6	2.889171000	1.151713000	-0.953483000
6	-0.030729000	1.954070000	2.253015000
6	-3.206733000	-0.897716000	4.017744000
6	2.927001000	0.282698000	-4.095704000
6	1.534356000	4.976596000	0.644130000
7	-1.751693000	-3.099015000	-0.136249000
6	-3.769246000	-1.640140000	5.228452000
6	-2.204802000	-3.796324000	-1.215551000
6	3.904194000	0.870514000	-5.080220000
6	2.398135000	6.129376000	1.216298000
6	1.904308000	7.436701000	0.650064000
8	-3.094819000	-2.045618000	6.173684000
8	-3.297530000	-3.632037000	-1.761232000
8	5.116472000	0.889607000	-4.964202000
6	-1.124975000	-4.758503000	-1.643329000
6	5.407615000	0.992319000	-1.168254000

7	-5.119420000	-1.810672000	5.168698000
8	3.275493000	1.393098000	-6.158079000
8	0.883611000	8.013243000	1.005956000
6	-1.322012000	0.340851000	5.141219000
6	-0.106704000	-5.961252000	0.312363000
6	6.405432000	-0.073737000	-1.627950000
6	-0.284925000	4.202670000	3.469741000
6	2.482816000	-1.973762000	5.988709000
6	5.122884000	-5.137386000	-0.243300000
7	2.150417000	0.010103000	-0.930593000
8	6.654822000	-2.428646000	-1.811366000
8	-0.771388000	6.253572000	2.370739000
6	-10.193503000	0.377565000	-2.001032000
6	-8.771141000	0.819124000	-1.619221000
6	-7.624220000	0.053401000	-2.305565000
6	-6.278398000	0.773517000	-2.098943000
6	-5.081168000	0.081878000	-2.760055000
16	-3.572494000	1.174057000	-2.646961000
6	-1.025508000	-0.649682000	-3.060646000
8	0.398723000	-1.368252000	-5.631484000
16	-2.080083000	0.034494000	-1.708981000
6	-1.732816000	-1.553401000	-4.053710000
8	-1.338856000	-3.096634000	-6.153015000
16	-0.519644000	-2.428058000	-5.105476000
8	0.182607000	-3.382141000	-4.187585000
6	-7.079413000	-3.637417000	-0.196192000
6	-6.536654000	-2.399843000	0.489472000
6	-5.243815000	-1.936683000	0.211863000
6	-7.313368000	-1.661899000	1.396647000
6	-4.750629000	-0.765382000	0.790645000
6	-6.834093000	-0.488824000	1.985911000
6	-5.552878000	-0.025523000	1.662346000
8	-5.132520000	1.151213000	2.233737000
6	6.057467000	0.113301000	4.745215000
6	5.321374000	-0.779438000	3.732763000
6	3.986113000	-0.191272000	3.266544000
7	3.655036000	-0.422876000	1.977342000
8	3.231456000	0.405735000	4.047653000
1	-1.619447000	7.895948000	-2.730815000
1	-1.629385000	7.737992000	-0.973392000
1	1.132144000	6.547110000	-3.243302000
1	-2.178925000	5.390433000	-0.765122000
1	1.759834000	4.152771000	-3.503025000
1	-1.553894000	3.008239000	-1.032876000
1	0.159306000	1.561998000	-1.796248000
1	-6.337272000	-4.057667000	-0.882079000

1	-7.979909000	-3.403133000	-0.777959000
1	-4.612935000	-2.490625000	-0.478847000
1	-8.319903000	-1.997009000	1.637712000
1	-3.749726000	-0.417580000	0.546382000
1	-7.450881000	0.092447000	2.665121000
1	-4.296633000	1.422611000	1.820630000
1	1.173895000	-2.693149000	3.884065000
1	1.088930000	-0.950125000	3.816089000
1	2.469303000	-6.027979000	-0.300268000
1	2.492939000	-4.566990000	0.677869000
1	0.660475000	-0.910511000	6.306731000
1	0.505358000	-2.663193000	6.296253000
1	3.990096000	-4.875104000	-1.989254000
1	4.060612000	-3.482978000	-0.931877000
1	-1.847396000	1.472284000	1.220124000
1	-2.089179000	1.921223000	2.932451000
1	-0.411719000	-4.300004000	2.196391000
1	2.745950000	-2.720287000	-2.832050000
1	4.443759000	-0.820909000	-3.054193000
1	2.177060000	3.755053000	2.342015000
1	-1.305968000	-2.737640000	4.555526000
1	1.425503000	-4.793358000	-2.192986000
1	4.068702000	1.962687000	-2.564383000
1	-0.715227000	3.795849000	1.397546000
1	-2.049400000	-2.634426000	2.285187000
1	0.470152000	1.641641000	3.183018000
1	-3.508946000	-1.461070000	3.127821000
1	-3.734832000	0.059945000	3.936936000
1	2.404460000	-0.551622000	-4.582599000
1	2.146191000	1.022139000	-3.883733000
1	0.526037000	5.349186000	0.471268000
1	1.917917000	4.718664000	-0.344020000
1	3.454530000	5.985368000	0.975594000
1	2.294591000	6.195145000	2.301305000
1	-0.759436000	-4.398233000	-2.614227000
1	-1.533203000	-5.762917000	-1.791349000
1	5.213306000	0.836215000	-0.103391000
1	5.889367000	1.971664000	-1.249802000
1	-0.278062000	0.660547000	5.096066000
1	-1.509491000	-0.133882000	6.105867000
1	-1.945869000	1.240136000	5.086007000
1	0.107437000	-6.843331000	-0.301717000
1	0.594415000	-5.959623000	1.150670000
1	-1.118187000	-6.078251000	0.718857000
1	6.502043000	-0.110756000	-2.716931000
1	7.403734000	0.171364000	-1.238330000

1	0.349995000	3.828588000	4.277642000
1	-1.331404000	4.021843000	3.745712000
1	-10.300388000	0.310181000	-3.091186000
1	-10.924887000	1.115113000	-1.647478000
1	-8.669568000	1.883068000	-1.878008000
1	-8.638516000	0.759041000	-0.530158000
1	-7.563278000	-0.970874000	-1.913977000
1	-7.830317000	-0.029380000	-3.382597000
1	-6.369081000	1.793767000	-2.498757000
1	-6.077192000	0.873502000	-1.026059000
1	-5.265352000	-0.112082000	-3.822041000
1	-4.841212000	-0.866981000	-2.274860000
1	-0.049373000	2.368268000	-4.970959000
1	-0.271294000	-1.203283000	-2.494854000
1	-0.531000000	0.177705000	-3.569764000
1	-2.331696000	-2.322319000	-3.560231000
1	-2.376458000	-0.975967000	-4.723776000
1	6.610034000	0.919191000	4.250157000
1	5.324500000	0.569299000	5.419423000
1	5.956489000	-1.022229000	2.873089000
1	5.052535000	-1.728889000	4.212642000
1	-7.354714000	-4.422807000	0.521438000
1	-0.185565000	8.313429000	-1.798181000
1	6.754149000	-0.468686000	5.358102000
6	-0.498795000	1.953921000	-5.872089000
1	-0.132963000	2.495799000	-6.746011000
1	-1.583424000	2.056601000	-5.809189000
1	-0.233776000	0.900038000	-5.950692000
1	3.927616000	-1.339804000	7.015249000
1	5.708327000	-6.359969000	1.061050000
1	6.489867000	-3.304997000	-1.362256000
1	3.966555000	1.732995000	-6.758372000
1	-0.334387000	7.103851000	2.115117000
1	-5.569261000	-2.365778000	5.883792000
1	-5.652974000	-1.571079000	4.342126000
1	4.285093000	-0.850167000	1.303957000
1	2.761127000	-0.086814000	1.631826000
1	-10.499082000	-0.586750000	-1.577890000
1	0.915782000	-3.161891000	1.930035000
1	-2.160761000	-2.194697000	0.067094000
8	2.669075000	7.913319000	-0.345994000
1	2.248017000	8.732019000	-0.674301000
1	3.331407000	3.105123000	-0.310138000

E

RC
E-RC-opt.gjf.log

Temperature 298.150 Kelvin. Pressure 1.00000 Atm.

Zero-point correction= 1.517619 (Hartree/Particle)
Thermal correction to Energy= 1.617365
Thermal correction to Enthalpy= 1.618309
Thermal correction to Gibbs Free Energy= 1.364101
Sum of electronic and zero-point Energies= -5784.754234
Sum of electronic and thermal Energies= -5784.654488
Sum of electronic and thermal Enthalpies= -5784.653544
Sum of electronic and thermal Free Energies= -5784.907752

6	1.170651000	-3.553766000	6.668317000
6	0.771446000	-3.539022000	5.212949000
6	-0.210143000	-4.412290000	4.717197000
6	1.348583000	-2.633983000	4.316125000
6	-0.616085000	-4.373189000	3.384377000
6	0.955602000	-2.580737000	2.978654000
6	-0.034770000	-3.449862000	2.507122000
8	-0.468126000	-3.434183000	1.213028000
6	-0.419380000	5.098808000	0.412153000
6	-3.276122000	1.985692000	-3.791893000
6	-7.831152000	-2.572713000	-0.297531000
6	-0.487524000	0.826554000	6.808000000
7	0.582630000	1.992609000	0.211232000
6	-0.253360000	6.559657000	0.891152000
6	-4.587470000	1.191619000	-3.679184000
7	-0.869138000	0.730233000	-2.026694000
8	-8.078822000	-1.388580000	-0.420846000
8	-1.326956000	0.992754000	7.669623000
7	-1.121305000	0.240912000	1.983178000
8	-2.053013000	6.688757000	2.422627000
8	-5.969960000	3.189383000	-3.728405000
8	-2.250346000	7.898524000	0.530729000
8	-6.488095000	1.527879000	-2.293910000
6	1.028930000	1.376008000	2.498217000
6	-0.214778000	3.118437000	-1.871526000
6	-2.497044000	-0.971086000	-2.621040000
6	-2.863983000	-1.496947000	2.158920000
28	-0.852162000	0.372684000	-0.022974000
6	1.062708000	2.331688000	1.351702000
6	-0.128853000	1.828230000	-2.709425000
6	-2.685328000	-1.597252000	-1.428070000
6	-1.975061000	-0.484524000	2.670531000

6	1.607018000	3.760460000	1.429182000
6	-0.707144000	1.882532000	-4.191711000
6	-3.454550000	-2.886986000	-1.275189000
6	-1.963235000	-0.135149000	4.157282000
6	0.919398000	4.384746000	0.159851000
6	-2.035567000	1.098729000	-4.062859000
6	-3.972632000	-2.745139000	0.167410000
6	-0.591972000	0.529440000	4.300629000
6	0.788672000	3.118896000	-0.727410000
6	-1.766856000	0.235853000	-2.843451000
6	-2.972302000	-1.765350000	0.776076000
6	-0.430628000	1.160911000	2.914822000
6	3.157870000	3.629354000	1.231376000
6	-2.541891000	-4.110334000	-1.456085000
6	-2.250993000	-1.332121000	5.052146000
7	1.254780000	1.428030000	-2.921680000
6	3.831968000	4.958386000	0.918176000
6	1.555113000	0.938412000	-4.155882000
6	-3.322075000	-5.400688000	-1.457417000
6	-3.571213000	-1.950613000	4.613290000
6	-3.650003000	-2.287943000	3.123819000
8	3.937579000	5.864444000	1.742683000
8	2.637495000	0.454053000	-4.478301000
8	-4.529922000	-5.513265000	-1.563272000
6	0.320283000	1.065298000	-5.018026000
6	-5.391813000	-2.133655000	0.203944000
7	4.315137000	5.077083000	-0.352005000
8	-2.506583000	-6.473507000	-1.347305000
8	-4.416314000	-3.209814000	2.812754000
6	1.315731000	4.442173000	2.775215000
6	-0.829047000	3.292680000	-4.775715000
6	-6.468133000	-3.180376000	-0.075913000
6	-0.444507000	1.518939000	5.465069000
6	-1.600381000	7.147491000	1.230353000
6	-5.732448000	2.088249000	-3.277432000
7	-2.291314000	-1.110422000	-0.190588000
8	-8.796423000	-3.522060000	-0.362770000
8	0.536470000	-0.050259000	6.948809000
6	10.954254000	-3.009191000	0.312529000
6	10.563026000	-2.120880000	1.504573000
6	9.947968000	-0.767638000	1.103406000
6	8.734718000	-0.786141000	0.158293000
6	7.377410000	-1.181750000	0.750667000
16	7.213364000	-3.012341000	1.021231000
6	0.750555000	-2.362385000	-1.967019000
8	-0.352716000	-4.333452000	-3.962341000

16	1.713880000	-1.793723000	-0.516263000
6	1.536511000	-2.630107000	-3.238774000
8	1.276975000	-3.551826000	-5.694906000
16	0.393114000	-3.180647000	-4.557350000
8	-0.489385000	-2.002708000	-4.830916000
6	8.386947000	-0.064392000	-3.407640000
6	7.054389000	0.210366000	-2.761226000
6	6.020376000	-0.731091000	-2.836640000
6	6.822576000	1.377717000	-2.019955000
6	4.796362000	-0.525266000	-2.201977000
6	5.611412000	1.591686000	-1.361540000
6	4.593160000	0.637504000	-1.453099000
8	3.403209000	0.896883000	-0.812476000
6	-4.607266000	5.210647000	0.387844000
6	-4.877412000	3.849501000	-0.245907000
6	-3.914716000	2.778049000	0.271408000
7	-4.194296000	1.506061000	-0.106740000
8	-2.939000000	3.063766000	0.970320000
1	1.766093000	-4.441198000	6.921070000
1	1.766545000	-2.671179000	6.922708000
1	-0.684846000	-5.120561000	5.392844000
1	2.103126000	-1.936126000	4.671535000
1	-1.396169000	-5.030841000	3.013201000
1	1.394065000	-1.852621000	2.306181000
1	-0.083943000	-2.657612000	0.755272000
1	8.323600000	-0.921895000	-4.086181000
1	9.146067000	-0.308077000	-2.653734000
1	6.165427000	-1.641095000	-3.413441000
1	7.606908000	2.127389000	-1.942237000
1	3.995042000	-1.245521000	-2.310400000
1	5.455066000	2.488617000	-0.770399000
1	2.883913000	0.060672000	-0.731866000
1	-0.979159000	5.139892000	-0.527597000
1	-1.049397000	4.529450000	1.099547000
1	-3.389830000	2.721020000	-4.593271000
1	-3.116638000	2.547154000	-2.862865000
1	0.387789000	6.619900000	1.771779000
1	0.191959000	7.165580000	0.097446000
1	-4.837815000	0.743990000	-4.650158000
1	-4.502233000	0.373791000	-2.963150000
1	1.451481000	0.413302000	2.194168000
1	1.613327000	1.750348000	3.342564000
1	-1.235791000	3.215296000	-1.491319000
1	0.003469000	3.987301000	-2.498952000
1	-2.928202000	-1.431434000	-3.502290000
1	-4.267461000	-2.950410000	-2.003603000

1	-2.748602000	0.624537000	4.319446000
1	1.590404000	5.096744000	-0.333205000
1	-2.226067000	0.489479000	-4.953223000
1	-3.975592000	-3.688837000	0.708168000
1	0.162567000	-0.257308000	4.407105000
1	1.777643000	2.962308000	-1.174585000
1	-0.959741000	2.126497000	2.882576000
1	3.371010000	2.888286000	0.457421000
1	3.593488000	3.253239000	2.163320000
1	-1.982784000	-4.065827000	-2.401968000
1	-1.793737000	-4.158442000	-0.657478000
1	-2.301643000	-1.027997000	6.103350000
1	-1.442010000	-2.061863000	4.955700000
1	-4.394865000	-1.249443000	4.820299000
1	-3.799163000	-2.872092000	5.157320000
1	-0.017747000	0.040994000	-5.212989000
1	0.566708000	1.530193000	-5.977582000
1	-5.457133000	-1.329780000	-0.539930000
1	-5.579687000	-1.680111000	1.179726000
1	0.243263000	4.544258000	2.962792000
1	1.786473000	5.426095000	2.810055000
1	1.745053000	3.860773000	3.597559000
1	-1.237471000	3.239149000	-5.791281000
1	-1.476350000	3.948997000	-4.188024000
1	0.159024000	3.762857000	-4.841233000
1	-6.218266000	-3.786222000	-0.956119000
1	-6.531996000	-3.891107000	0.756540000
1	-1.239282000	2.269488000	5.446186000
1	0.519758000	2.036268000	5.385758000
1	10.078122000	-3.525970000	-0.096496000
1	11.681084000	-3.774853000	0.604981000
1	9.876827000	-2.673119000	2.159386000
1	11.454215000	-1.909305000	2.110725000
1	9.681231000	-0.208239000	2.011562000
1	10.734370000	-0.182267000	0.605450000
1	8.597323000	0.232272000	-0.225101000
1	8.941666000	-1.409935000	-0.718913000
1	6.588060000	-0.891302000	0.053272000
1	7.209237000	-0.667618000	1.702425000
1	5.864321000	-3.047922000	1.067321000
1	0.025624000	-1.565174000	-2.124825000
1	0.211712000	-3.263324000	-1.665848000
1	2.046434000	-1.738167000	-3.610932000
1	2.263994000	-3.437250000	-3.111729000
1	-4.834195000	5.201959000	1.459217000
1	-3.553040000	5.470577000	0.276484000

1	-5.910617000	3.522413000	-0.073057000
1	-4.759037000	3.909712000	-1.336260000
1	8.790267000	0.777352000	-3.985843000
1	0.288837000	-3.563264000	7.321869000
1	-5.194703000	6.002387000	-0.089462000
6	2.814349000	-3.219098000	-0.217850000
1	3.585315000	-3.302528000	-0.982173000
1	3.283664000	-3.060671000	0.752470000
1	2.222439000	-4.133315000	-0.179764000
1	-2.951365000	7.050086000	2.547773000
1	-7.213912000	2.149223000	-2.087859000
1	-9.639201000	-3.056544000	-0.525198000
1	-3.072990000	-7.267564000	-1.394507000
1	0.424397000	-0.488405000	7.813992000
1	4.702589000	5.965801000	-0.641067000
1	4.141019000	4.383887000	-1.065368000
1	-4.991906000	1.290537000	-0.690794000
1	-3.504437000	0.780666000	0.066908000
1	1.905895000	1.275340000	-2.158505000
1	11.381032000	-2.423658000	-0.511548000

TS_1
E-TS_1-opt.gjf.log

Temperature 298.150 Kelvin. Pressure 1.00000 Atm.

Zero-point correction= 1.514271 (Hartree/Particle)
Thermal correction to Energy= 1.613737
Thermal correction to Enthalpy= 1.614681
Thermal correction to Gibbs Free Energy= 1.361471
Sum of electronic and zero-point Energies= -5784.720460
Sum of electronic and thermal Energies= -5784.620994
Sum of electronic and thermal Enthalpies= -5784.620050
Sum of electronic and thermal Free Energies= -5784.873260

6	1.613464000	-5.996750000	5.040453000
6	1.149738000	-5.367724000	3.748748000
6	0.157103000	-5.971654000	2.960488000
6	1.700341000	-4.164881000	3.290769000
6	-0.266276000	-5.404933000	1.759820000
6	1.291507000	-3.582853000	2.089899000
6	0.308509000	-4.208460000	1.308988000
8	-0.115420000	-3.701002000	0.121766000
6	-0.983176000	4.743346000	1.879020000
6	-3.564899000	2.825147000	-3.102229000
6	-7.572878000	-3.058839000	-0.978014000

6	-0.021847000	-1.559355000	6.613982000
7	0.393046000	1.987500000	0.803500000
6	-1.054665000	5.930270000	2.850834000
6	-4.800708000	1.938495000	-3.370052000
7	-0.964878000	1.323328000	-1.790341000
8	-7.949352000	-1.944698000	-0.669360000
8	-0.786018000	-1.790029000	7.529097000
7	-0.971358000	-0.478459000	1.940147000
8	-2.786583000	7.093089000	3.994535000
8	-6.864038000	3.230963000	-3.352735000
8	-3.109579000	6.703636000	1.802015000
8	-6.132315000	2.285776000	-1.441470000
6	1.043546000	0.735413000	2.764322000
6	-0.587423000	3.586246000	-0.854884000
6	-2.520941000	-0.236497000	-2.810473000
6	-2.568821000	-2.321699000	1.557781000
28	-0.671861000	0.244059000	-0.008262000
6	0.867657000	2.005014000	1.992938000
6	-0.365573000	2.656412000	-2.066163000
6	-2.616065000	-1.208129000	-1.865564000
6	-1.723567000	-1.467817000	2.355267000
6	1.209596000	3.399053000	2.519793000
6	-0.993635000	3.114926000	-3.453005000
6	-3.244943000	-2.554565000	-2.116749000
6	-1.638783000	-1.643116000	3.869896000
6	0.432940000	4.294421000	1.486400000
6	-2.234694000	2.194611000	-3.589463000
6	-3.697488000	-2.935729000	-0.696206000
6	-0.312822000	-0.939012000	4.179211000
6	0.435403000	3.356646000	0.250093000
6	-1.867311000	1.033335000	-2.683157000
6	-2.751335000	-2.114979000	0.174378000
6	-0.332180000	0.160174000	3.113074000
6	2.758575000	3.549994000	2.344394000
6	-2.207905000	-3.544053000	-2.677586000
6	-1.781156000	-3.092625000	4.310954000
7	1.045854000	2.519938000	-2.374194000
6	3.229092000	4.993487000	2.470079000
6	1.367844000	2.509931000	-3.701147000
6	-2.848568000	-4.846236000	-3.088464000
6	-3.076540000	-3.645407000	3.733446000
6	-3.232416000	-3.461919000	2.224460000
8	3.144673000	5.633198000	3.516878000
8	2.493313000	2.320613000	-4.148725000
8	-4.043554000	-5.063537000	-3.177412000
6	0.093109000	2.722059000	-4.487141000

6	-5.162266000	-2.522058000	-0.429091000
7	3.748876000	5.532716000	1.331924000
8	-1.920872000	-5.784480000	-3.376638000
8	-3.955469000	-4.281247000	1.644909000
6	0.819806000	3.577810000	3.994208000
6	-1.272509000	4.617198000	-3.555559000
6	-6.143255000	-3.539353000	-1.008873000
6	-0.148750000	-0.428999000	5.617089000
6	-2.413933000	6.596942000	2.794110000
6	-6.040258000	2.556068000	-2.776233000
7	-2.193749000	-1.114738000	-0.546161000
8	-8.434409000	-4.033586000	-1.357088000
8	1.064584000	-2.326735000	6.359323000
6	10.975685000	-2.419533000	-0.955764000
6	10.542027000	-2.068516000	0.475697000
6	9.739461000	-0.759711000	0.586932000
6	8.462560000	-0.629075000	-0.260466000
6	7.228718000	-1.415839000	0.196975000
16	7.309598000	-3.229495000	-0.199579000
6	0.776242000	-1.591866000	-2.355914000
8	0.173414000	-2.771632000	-5.014113000
16	1.288994000	-1.035616000	-0.668573000
6	1.618063000	-1.116832000	-3.529189000
8	1.684865000	-1.142092000	-6.172346000
16	0.692775000	-1.370171000	-5.086440000
8	-0.409611000	-0.351423000	-5.056882000
6	8.065361000	1.556490000	-3.150908000
6	6.749151000	1.528758000	-2.417745000
6	5.788982000	0.548126000	-2.696176000
6	6.458047000	2.462273000	-1.413144000
6	4.574474000	0.502927000	-2.013679000
6	5.252303000	2.425010000	-0.713578000
6	4.299427000	1.448628000	-1.021765000
8	3.102474000	1.479552000	-0.352018000
6	-4.914223000	4.115702000	2.854561000
6	-4.512408000	3.592576000	1.473713000
6	-3.717299000	2.293214000	1.545808000
7	-3.871929000	1.455896000	0.490636000
8	-2.956467000	2.049753000	2.488895000
1	2.228131000	-6.888646000	4.858609000
1	2.220799000	-5.296753000	5.625063000
1	-0.301533000	-6.898893000	3.297779000
1	2.454043000	-3.659736000	3.890232000
1	-1.047090000	-5.867237000	1.163469000
1	1.724377000	-2.647011000	1.753105000
1	0.291542000	-2.805517000	-0.017820000

1	8.003961000	1.013067000	-4.099805000
1	8.862912000	1.087775000	-2.560135000
1	5.983595000	-0.188944000	-3.471528000
1	7.187255000	3.232690000	-1.171735000
1	3.832814000	-0.245859000	-2.256619000
1	5.046803000	3.147593000	0.070318000
1	2.582002000	0.642136000	-0.513879000
1	-1.485502000	5.077309000	0.967218000
1	-1.568657000	3.902226000	2.263789000
1	-3.717585000	3.779075000	-3.615723000
1	-3.495322000	3.042373000	-2.029518000
1	-0.839156000	5.660049000	3.884806000
1	-0.318019000	6.695909000	2.566771000
1	-4.956446000	1.827060000	-4.446316000
1	-4.661172000	0.947627000	-2.930972000
1	1.593523000	0.007182000	2.158032000
1	1.616394000	0.915472000	3.677436000
1	-1.607581000	3.435947000	-0.488931000
1	-0.490317000	4.628382000	-1.170685000
1	-2.937141000	-0.451491000	-3.787148000
1	-4.080162000	-2.482032000	-2.817734000
1	-2.464192000	-1.053412000	4.306526000
1	1.012326000	5.193586000	1.249626000
1	-2.365744000	1.860094000	-4.623970000
1	-3.583230000	-3.997589000	-0.491797000
1	0.507149000	-1.629284000	3.948250000
1	1.424395000	3.457045000	-0.210829000
1	-0.992832000	0.977317000	3.435232000
1	3.063351000	3.116496000	1.388769000
1	3.256961000	2.972118000	3.130310000
1	-1.677056000	-3.141416000	-3.551474000
1	-1.435643000	-3.758379000	-1.930424000
1	-1.790694000	-3.163588000	5.403734000
1	-0.928544000	-3.671704000	3.948016000
1	-3.936633000	-3.138909000	4.198737000
1	-3.194812000	-4.714248000	3.933231000
1	-0.121549000	1.760180000	-4.968339000
1	0.235737000	3.474349000	-5.268321000
1	-5.344205000	-1.532679000	-0.867475000
1	-5.336117000	-2.430977000	0.645412000
1	-0.248624000	3.403902000	4.151820000
1	1.082543000	4.581257000	4.332233000
1	1.370376000	2.873023000	4.626037000
1	-1.697562000	4.849516000	-4.538412000
1	-1.966471000	4.982677000	-2.793985000
1	-0.337516000	5.180925000	-3.459023000

1	-5.890447000	-3.796206000	-2.045188000
1	-6.080144000	-4.482933000	-0.453858000
1	-0.999492000	0.189636000	5.914915000
1	0.761170000	0.179786000	5.688299000
1	10.137399000	-2.831009000	-1.529633000
1	11.776222000	-3.167632000	-0.958156000
1	9.968396000	-2.904496000	0.896949000
1	11.432413000	-1.963767000	1.110632000
1	9.481042000	-0.585039000	1.641399000
1	10.407068000	0.064710000	0.296881000
1	8.159512000	0.425160000	-0.242951000
1	8.671233000	-0.862704000	-1.311129000
1	6.351028000	-1.022106000	-0.321740000
1	7.076022000	-1.296985000	1.274747000
1	5.970432000	-3.419370000	-0.299096000
1	-0.239306000	-1.230038000	-2.490021000
1	0.740784000	-2.682713000	-2.353178000
1	1.852087000	-0.052671000	-3.481175000
1	2.552084000	-1.678568000	-3.622170000
1	-5.467451000	3.354218000	3.415893000
1	-4.038325000	4.394155000	3.445054000
1	-5.393110000	3.458671000	0.836283000
1	-3.880130000	4.338425000	0.974242000
1	8.395540000	2.580009000	-3.372221000
1	0.771977000	-6.313499000	5.670133000
1	-5.563714000	4.990529000	2.742440000
6	3.537635000	-2.616559000	-0.621562000
1	4.032861000	-1.964204000	0.081200000
1	3.026686000	-3.492955000	-0.254052000
1	3.811203000	-2.561562000	-1.664037000
1	-3.655258000	7.522554000	3.869162000
1	-6.937835000	2.722320000	-1.103664000
1	-9.330275000	-3.646403000	-1.327271000
1	-2.402048000	-6.584702000	-3.662394000
1	1.064562000	-3.054032000	7.010718000
1	4.034212000	6.503139000	1.339980000
1	3.750931000	5.044791000	0.448081000
1	-4.576608000	1.621732000	-0.217830000
1	-3.348946000	0.587937000	0.453925000
1	1.719836000	2.179938000	-1.694176000
1	11.327537000	-1.536288000	-1.503606000

Int_1
E-Int_1-opt.gjf.log
Temperature 298.150 Kelvin. Pressure 1.00000 Atm.

Zero-point correction= 1.513892 (Hartree/Particle)
Thermal correction to Energy= 1.614034
Thermal correction to Enthalpy= 1.614978
Thermal correction to Gibbs Free Energy= 1.362080
Sum of electronic and zero-point Energies= -5784.725657
Sum of electronic and thermal Energies= -5784.625515
Sum of electronic and thermal Enthalpies= -5784.624570
Sum of electronic and thermal Free Energies= -5784.877469

6	-2.196686000	5.620261000	5.337266000
6	-1.646750000	5.104523000	4.028297000
6	-0.661376000	5.811212000	3.320292000
6	-2.115367000	3.909406000	3.468527000
6	-0.166432000	5.350987000	2.101299000
6	-1.635612000	3.434380000	2.246513000
6	-0.660542000	4.161295000	1.547541000
8	-0.174109000	3.763332000	0.342066000
6	1.087746000	-4.787426000	1.799732000
6	4.144686000	-2.350159000	-2.793463000
6	6.631212000	2.781284000	-1.953903000
6	-0.411262000	1.239742000	6.684662000
7	-0.215191000	-2.021740000	0.713679000
6	1.088641000	-6.021064000	2.712938000
6	5.274846000	-1.311821000	-2.945571000
7	1.269117000	-1.193068000	-1.765430000
8	6.342917000	1.631871000	-2.231847000
8	0.280708000	1.479274000	7.653425000
7	0.898052000	0.454413000	2.038038000
8	2.686033000	-7.243911000	3.981795000
8	7.610766000	-1.914935000	-2.607952000
8	3.305230000	-6.628339000	1.908574000
8	6.272878000	-1.644544000	-0.818643000
6	-1.097765000	-0.897139000	2.664031000
6	0.982941000	-3.506953000	-0.911832000
6	2.728747000	0.549224000	-2.614046000
6	2.341156000	2.445034000	1.822940000
28	0.744428000	-0.184232000	0.042032000
6	-0.817685000	-2.115459000	1.840470000
6	0.837523000	-2.569865000	-2.128663000
6	2.676902000	1.484018000	-1.632768000
6	1.537750000	1.484691000	2.536960000
6	-1.205620000	-3.542348000	2.232792000
6	1.671900000	-2.940629000	-3.433293000
6	3.211854000	2.880005000	-1.791257000
6	1.357948000	1.572322000	4.051287000
6	-0.285244000	-4.361672000	1.256992000

6	2.806888000	-1.883455000	-3.428152000
6	3.576060000	3.217038000	-0.336081000
6	0.068026000	0.767026000	4.245872000
6	-0.164107000	-3.357265000	0.081021000
6	2.212902000	-0.787106000	-2.563415000
6	2.620248000	2.312513000	0.443718000
6	0.221079000	-0.275728000	3.134166000
6	-2.706007000	-3.675882000	1.849498000
6	2.120669000	3.819536000	-2.337740000
6	1.379957000	2.998110000	4.580919000
7	-0.530350000	-2.544795000	-2.608401000
6	-3.268501000	-5.097174000	1.870578000
6	-0.689892000	-2.534100000	-3.962812000
6	2.669357000	5.192944000	-2.635621000
6	2.649090000	3.677194000	4.083929000
6	2.841762000	3.618217000	2.569625000
8	-2.700517000	-6.059316000	2.385753000
8	-1.765507000	-2.412553000	-4.538936000
8	3.837858000	5.531151000	-2.570999000
6	0.679678000	-2.661799000	-4.593728000
6	5.033985000	2.855813000	0.041976000
7	-4.483839000	-5.211955000	1.268850000
8	1.691058000	6.046669000	-3.005957000
8	3.449950000	4.563261000	2.053538000
6	-1.011698000	-3.808031000	3.733658000
6	2.135107000	-4.398873000	-3.492421000
6	6.084710000	3.594637000	-0.800877000
6	-0.143185000	0.174095000	5.645515000
6	2.468923000	-6.639285000	2.791942000
6	6.512727000	-1.660026000	-2.164633000
7	2.184180000	1.306318000	-0.345176000
8	7.526150000	3.491357000	-2.675461000
8	-1.537052000	1.937659000	6.401921000
6	-11.008167000	1.824566000	-1.322532000
6	-10.604787000	1.424879000	0.103638000
6	-9.641596000	0.226647000	0.171243000
6	-8.303019000	0.348857000	-0.576104000
6	-7.259546000	1.302870000	0.019421000
16	-7.582351000	3.093927000	-0.356729000
6	-0.714993000	1.601808000	-2.292360000
8	-0.113301000	2.839918000	-4.899607000
16	-1.249525000	0.998831000	-0.629747000
6	-1.489372000	1.068464000	-3.487730000
8	-1.463581000	1.129676000	-6.136752000
16	-0.531583000	1.406260000	-5.009079000
8	0.636416000	0.466590000	-4.948283000

6	-7.772612000	-1.901917000	-3.500301000
6	-6.423851000	-1.837344000	-2.826832000
6	-5.753076000	-0.614414000	-2.678246000
6	-5.806642000	-2.986370000	-2.315630000
6	-4.522254000	-0.528425000	-2.029570000
6	-4.569865000	-2.922872000	-1.672424000
6	-3.928632000	-1.689534000	-1.520338000
8	-2.724035000	-1.677868000	-0.874767000
6	4.972532000	-4.044759000	3.136993000
6	4.598328000	-3.437806000	1.784030000
6	3.722222000	-2.199531000	1.954356000
7	3.859772000	-1.239242000	1.007563000
8	2.920403000	-2.102485000	2.890009000
1	-2.864005000	6.479028000	5.182612000
1	-2.779810000	4.847164000	5.850413000
1	-0.264452000	6.735057000	3.736072000
1	-2.861132000	3.325638000	4.002870000
1	0.612115000	5.890436000	1.570811000
1	-2.008977000	2.505690000	1.827428000
1	-0.509908000	2.850921000	0.130482000
1	-7.797484000	-1.288443000	-4.408402000
1	-8.567760000	-1.529862000	-2.840697000
1	-6.200940000	0.292752000	-3.077651000
1	-6.294406000	-3.951734000	-2.431131000
1	-4.011629000	0.422748000	-1.926246000
1	-4.080585000	-3.822837000	-1.310058000
1	-2.333446000	-0.753213000	-0.820358000
1	1.711704000	-5.052957000	0.942058000
1	1.589803000	-3.951488000	2.297806000
1	4.462879000	-3.280237000	-3.274216000
1	3.984827000	-2.575714000	-1.732771000
1	0.742495000	-5.812251000	3.725610000
1	0.416026000	-6.791572000	2.309973000
1	5.560235000	-1.207111000	-3.995344000
1	4.951596000	-0.333480000	-2.582233000
1	-1.644407000	-0.162000000	2.062329000
1	-1.717403000	-1.150933000	3.527556000
1	1.949691000	-3.313728000	-0.435970000
1	0.975684000	-4.545102000	-1.255254000
1	3.197674000	0.836103000	-3.547581000
1	4.066491000	2.901222000	-2.466772000
1	2.196681000	1.011253000	4.500092000
1	-0.807899000	-5.259272000	0.916534000
1	3.013655000	-1.523016000	-4.441483000
1	3.410904000	4.264507000	-0.099506000
1	-0.782115000	1.414200000	3.999116000

1	-1.090733000	-3.449230000	-0.492177000
1	0.901233000	-1.073419000	3.460985000
1	-2.881507000	-3.238196000	0.861270000
1	-3.305193000	-3.073927000	2.546528000
1	1.659209000	3.428159000	-3.255206000
1	1.304019000	3.928152000	-1.613918000
1	1.348452000	3.002053000	5.675583000
1	0.500265000	3.537084000	4.221515000
1	3.529551000	3.191349000	4.533319000
1	2.689602000	4.733017000	4.365763000
1	0.864739000	-1.698453000	-5.079892000
1	0.685041000	-3.443632000	-5.359117000
1	5.185010000	1.775241000	-0.062681000
1	5.172311000	3.109681000	1.094385000
1	0.015333000	-3.603377000	4.051115000
1	-1.263033000	-4.844840000	3.958854000
1	-1.676319000	-3.171428000	4.327816000
1	2.705169000	-4.570860000	-4.412350000
1	2.765603000	-4.685874000	-2.646408000
1	1.268898000	-5.070034000	-3.511463000
1	5.678771000	4.522832000	-1.219998000
1	6.948164000	3.886899000	-0.190457000
1	0.731390000	-0.398032000	5.966359000
1	-1.008451000	-0.500274000	5.629920000
1	-10.182547000	2.323714000	-1.842233000
1	-11.859441000	2.514530000	-1.315994000
1	-10.167939000	2.292104000	0.616024000
1	-11.505346000	1.159585000	0.674184000
1	-9.433008000	-0.005928000	1.225505000
1	-10.165415000	-0.650191000	-0.236559000
1	-7.832878000	-0.642882000	-0.582085000
1	-8.469541000	0.602604000	-1.629837000
1	-6.282770000	1.066466000	-0.408787000
1	-7.198589000	1.175250000	1.105185000
1	-6.287411000	3.491061000	-0.325873000
1	0.328243000	1.317712000	-2.402057000
1	-0.760381000	2.692335000	-2.289062000
1	-1.641534000	-0.012423000	-3.450711000
1	-2.462998000	1.554735000	-3.599780000
1	5.450828000	-3.294343000	3.777514000
1	4.082250000	-4.402384000	3.659672000
1	5.488367000	-3.194893000	1.193616000
1	4.026813000	-4.171498000	1.200661000
1	-8.034916000	-2.930708000	-3.780155000
1	-1.409441000	5.952766000	6.026206000
1	5.673009000	-4.879776000	3.028291000

6	-3.789284000	2.963992000	-0.635480000
1	-4.136808000	2.268227000	0.112125000
1	-3.043497000	3.697077000	-0.371342000
1	-4.067286000	2.825175000	-1.669627000
1	3.579364000	-7.637553000	3.942245000
1	7.114793000	-1.850854000	-0.369701000
1	7.836266000	2.911238000	-3.397358000
1	2.116437000	6.904453000	-3.197746000
1	-1.626554000	2.631321000	7.083149000
1	-4.933180000	-6.117111000	1.243740000
1	-4.936009000	-4.437148000	0.803579000
1	4.580685000	-1.283208000	0.296517000
1	3.284450000	-0.406860000	1.058198000
1	-1.305278000	-2.253386000	-2.015342000
1	-11.281375000	0.948233000	-1.923795000

TS_2

E-TS_2-opt.gjf.log

Temperature 298.150 Kelvin. Pressure 1.00000 Atm.

Zero-point correction= 1.513048 (Hartree/Particle)
Thermal correction to Energy= 1.611657
Thermal correction to Enthalpy= 1.612602
Thermal correction to Gibbs Free Energy= 1.362551
Sum of electronic and zero-point Energies= -5784.724593
Sum of electronic and thermal Energies= -5784.625983
Sum of electronic and thermal Enthalpies= -5784.625039
Sum of electronic and thermal Free Energies= -5784.875090

6	2.101762000	-5.826951000	5.123043000
6	1.535223000	-5.268921000	3.838725000
6	0.522600000	-5.940014000	3.134293000
6	2.008410000	-4.065236000	3.302406000
6	0.006056000	-5.437796000	1.941006000
6	1.505751000	-3.547374000	2.107109000
6	0.503803000	-4.239181000	1.409698000
8	-0.009963000	-3.795073000	0.233495000
6	-0.894788000	4.721722000	1.949281000
6	-4.065285000	2.566119000	-2.705975000
6	-6.771396000	-2.464283000	-1.961130000
6	0.406216000	-1.485065000	6.638682000
7	0.302474000	1.947142000	0.766297000
6	-0.846934000	5.921855000	2.904724000
6	-5.240853000	1.584408000	-2.887434000
7	-1.237556000	1.254559000	-1.719520000

8	-6.435956000	-1.319479000	-2.202805000
8	-0.288082000	-1.725434000	7.605548000
7	-0.898854000	-0.521373000	2.024769000
8	-2.389118000	7.177446000	4.208797000
8	-7.547314000	2.282257000	-2.530210000
8	-3.037648000	6.642580000	2.122227000
8	-6.223579000	1.899095000	-0.750950000
6	1.153925000	0.729729000	2.673440000
6	-0.849323000	3.527731000	-0.801581000
6	-2.780092000	-0.394967000	-2.605209000
6	-2.431292000	-2.438704000	1.769242000
28	-0.736891000	0.170493000	0.047683000
6	0.915284000	1.982803000	1.890902000
6	-0.747647000	2.621349000	-2.045411000
6	-2.761144000	-1.358914000	-1.651403000
6	-1.579156000	-1.536199000	2.501293000
6	1.354547000	3.381379000	2.328443000
6	-1.569227000	3.065189000	-3.334987000
6	-3.359946000	-2.724246000	-1.844747000
6	-1.390635000	-1.675898000	4.010496000
6	0.459448000	4.264500000	1.385450000
6	-2.749471000	2.058865000	-3.355294000
6	-3.722579000	-3.088968000	-0.395960000
6	-0.069628000	-0.926523000	4.218235000
6	0.296515000	3.304179000	0.178321000
6	-2.203159000	0.914371000	-2.522374000
6	-2.719278000	-2.251729000	0.398707000
6	-0.186578000	0.150515000	3.135723000
6	2.857020000	3.476390000	1.943359000
6	-2.318514000	-3.694723000	-2.432881000
6	-1.460882000	-3.115989000	4.495909000
7	0.616339000	2.551701000	-2.531268000
6	3.469226000	4.875481000	2.014841000
6	0.771823000	2.575710000	-3.885986000
6	-2.932377000	-5.032513000	-2.764306000
6	-2.760552000	-3.730718000	3.994171000
6	-2.970885000	-3.613791000	2.485451000
8	2.929856000	5.841317000	2.553139000
8	1.839790000	2.426751000	-4.469942000
8	-4.114441000	-5.318866000	-2.696372000
6	-0.593298000	2.777549000	-4.507060000
6	-5.158690000	-2.676149000	0.011771000
7	4.692688000	4.966263000	1.425547000
8	-1.997840000	-5.918361000	-3.170266000
8	-3.624663000	-4.515529000	1.948272000
6	1.175833000	3.602596000	3.838494000

6	-1.968560000	4.543374000	-3.350873000
6	-6.250738000	-3.339031000	-0.841562000
6	0.172294000	-0.380802000	5.631959000
6	-2.199972000	6.595163000	3.003123000
6	-6.462176000	1.964977000	-2.095438000
7	-2.247150000	-1.241728000	-0.365063000
8	-7.702422000	-3.110158000	-2.697093000
8	1.502311000	-2.217207000	6.327393000
6	10.904425000	-2.137265000	-1.607621000
6	10.640829000	-1.752393000	-0.143151000
6	9.881078000	-0.423446000	0.021598000
6	8.488788000	-0.316289000	-0.624344000
6	7.344892000	-1.052503000	0.084840000
16	7.299746000	-2.881788000	-0.220590000
6	0.604828000	-1.595673000	-2.375642000
8	-0.082503000	-2.738365000	-5.008283000
16	1.197288000	-1.070837000	-0.704920000
6	1.385280000	-1.066199000	-3.568807000
8	1.313720000	-1.044778000	-6.217830000
16	0.390064000	-1.319555000	-5.083036000
8	-0.739473000	-0.337462000	-4.974855000
6	7.742484000	1.730003000	-3.643207000
6	6.427292000	1.677707000	-2.907939000
6	5.708254000	0.477411000	-2.815445000
6	5.885428000	2.813442000	-2.292158000
6	4.496715000	0.401565000	-2.129818000
6	4.672426000	2.758641000	-1.605103000
6	3.977052000	1.548432000	-1.517957000
8	2.792595000	1.547379000	-0.835056000
6	-4.817779000	4.089081000	3.267295000
6	-4.465928000	3.515883000	1.893474000
6	-3.638359000	2.239280000	2.018384000
7	-3.825103000	1.313022000	1.046477000
8	-2.831884000	2.082102000	2.941810000
1	2.741388000	-6.700168000	4.936406000
1	2.715294000	-5.080652000	5.640120000
1	0.121199000	-6.869470000	3.532986000
1	2.773639000	-3.506503000	3.836111000
1	-0.792707000	-5.950739000	1.414106000
1	1.879042000	-2.609508000	1.710629000
1	0.360761000	-2.895659000	0.027358000
1	7.703444000	1.147052000	-4.570716000
1	8.557112000	1.311787000	-3.037947000
1	6.097699000	-0.418012000	-3.294417000
1	6.412812000	3.762228000	-2.361527000
1	3.942944000	-0.529196000	-2.082863000

1	4.240812000	3.651446000	-1.161225000
1	2.361180000	0.639911000	-0.828783000
1	-1.510779000	5.040188000	1.103973000
1	-1.426784000	3.888313000	2.419769000
1	-4.341614000	3.523121000	-3.158912000
1	-3.895923000	2.753111000	-1.639272000
1	-0.513816000	5.663486000	3.910385000
1	-0.140426000	6.676708000	2.531235000
1	-5.530036000	1.523509000	-3.939621000
1	-4.962103000	0.582505000	-2.552733000
1	1.664875000	-0.008441000	2.044738000
1	1.790057000	0.930845000	3.538793000
1	-1.820237000	3.358672000	-0.325085000
1	-0.803418000	4.574357000	-1.114818000
1	-3.267927000	-0.634414000	-3.542261000
1	-4.222087000	-2.686809000	-2.509939000
1	-2.204350000	-1.098678000	4.484098000
1	1.012600000	5.153183000	1.071068000
1	-2.971932000	1.734349000	-4.377355000
1	-3.600896000	-4.149293000	-0.192821000
1	0.753408000	-1.598869000	3.949193000
1	1.223186000	3.381416000	-0.397260000
1	-0.831980000	0.966298000	3.488061000
1	3.013819000	3.070583000	0.938567000
1	3.437256000	2.827569000	2.613849000
1	-1.850674000	-3.297186000	-3.344526000
1	-1.499523000	-3.861665000	-1.722927000
1	-1.416909000	-3.155240000	5.589423000
1	-0.606265000	-3.675987000	4.108748000
1	-3.616506000	-3.228121000	4.471546000
1	-2.837211000	-4.793197000	4.241468000
1	-0.821037000	1.836700000	-5.018920000
1	-0.568581000	3.579804000	-5.250592000
1	-5.262557000	-1.587095000	-0.055762000
1	-5.296490000	-2.958216000	1.057076000
1	0.143393000	3.424138000	4.154071000
1	1.464545000	4.621670000	4.097385000
1	1.819633000	2.923024000	4.407550000
1	-2.532320000	4.766073000	-4.263779000
1	-2.584482000	4.833450000	-2.495191000
1	-1.073995000	5.176544000	-3.353660000
1	-5.892369000	-4.270737000	-1.294826000
1	-7.120264000	-3.611081000	-0.230394000
1	-0.677992000	0.214552000	5.974987000
1	1.062399000	0.260657000	5.628729000
1	10.002856000	-2.549436000	-2.075006000

1	11.697583000	-2.888059000	-1.691841000
1	10.102809000	-2.565638000	0.361611000
1	11.599582000	-1.651204000	0.383693000
1	9.787492000	-0.192709000	1.092764000
1	10.507518000	0.372433000	-0.407058000
1	8.204172000	0.744729000	-0.632928000
1	8.523420000	-0.624764000	-1.675839000
1	6.394135000	-0.658941000	-0.284024000
1	7.391032000	-0.875306000	1.164697000
1	5.866795000	-2.970796000	-0.251651000
1	-0.423951000	-1.256145000	-2.459535000
1	0.593117000	-2.686752000	-2.406012000
1	1.587234000	0.005135000	-3.505179000
1	2.333889000	-1.592903000	-3.710600000
1	-5.315219000	3.331975000	3.885021000
1	-3.916051000	4.404071000	3.797880000
1	-5.364232000	3.328667000	1.295506000
1	-3.865870000	4.247064000	1.336229000
1	8.029877000	2.756722000	-3.905405000
1	1.317138000	-6.152078000	5.818478000
1	-5.495199000	4.946312000	3.189785000
6	4.162571000	-2.770255000	-0.292775000
1	4.132073000	-1.758911000	0.086767000
1	3.847609000	-3.563598000	0.372071000
1	3.995283000	-2.903302000	-1.353262000
1	-3.264912000	7.609634000	4.182007000
1	-7.055387000	2.129125000	-0.294749000
1	-7.993317000	-2.493093000	-3.395986000
1	-2.463742000	-6.749923000	-3.382352000
1	1.570146000	-2.932663000	6.988236000
1	5.180596000	5.851354000	1.444886000
1	5.133093000	4.183745000	0.962193000
1	-4.545655000	1.410959000	0.340581000
1	-3.278322000	0.460314000	1.060831000
1	1.380026000	2.213508000	-1.949136000
1	11.192774000	-1.255745000	-2.194053000

Int_2
E-Int_2-opt.gjf.log

Temperature 298.150 Kelvin. Pressure 1.00000 Atm.

Zero-point correction= 1.518324 (Hartree/Particle)
Thermal correction to Energy= 1.618164
Thermal correction to Enthalpy= 1.619108
Thermal correction to Gibbs Free Energy= 1.365831

Sum of electronic and zero-point Energies= -5784.762881
Sum of electronic and thermal Energies= -5784.663041
Sum of electronic and thermal Enthalpies= -5784.662097
Sum of electronic and thermal Free Energies= -5784.915374

6	2.136774000	-5.246316000	5.718306000
6	1.579912000	-4.822039000	4.379131000
6	0.558449000	-5.550571000	3.748441000
6	2.073579000	-3.691773000	3.716013000
6	0.051053000	-5.171592000	2.506766000
6	1.582537000	-3.299423000	2.469423000
6	0.568376000	-4.044996000	1.851624000
8	0.058628000	-3.722396000	0.631996000
6	-0.878264000	4.979390000	1.459626000
6	-4.088464000	2.289260000	-2.901938000
6	-6.725759000	-2.668049000	-1.631438000
6	0.482481000	-0.750946000	6.774920000
7	0.295258000	2.073674000	0.586059000
6	-0.796760000	6.279792000	2.267227000
6	-5.257245000	1.285135000	-2.974881000
7	-1.243021000	1.110033000	-1.809457000
8	-6.403869000	-1.549234000	-1.986704000
8	-0.204923000	-0.895119000	7.765610000
7	-0.864160000	-0.262855000	2.098451000
8	-2.157155000	7.939717000	3.277691000
8	-7.563425000	2.005191000	-2.662272000
8	-3.057115000	6.834216000	1.537141000
8	-6.224169000	1.784663000	-0.867064000
6	1.176546000	1.072776000	2.602837000
6	-0.865075000	3.471828000	-1.142910000
6	-2.771300000	-0.637765000	-2.509952000
6	-2.367272000	-2.219951000	2.046289000
28	-0.713458000	0.222065000	0.067280000
6	0.921329000	2.233350000	1.692456000
6	-0.766714000	2.437308000	-2.283267000
6	-2.728320000	-1.500633000	-1.463843000
6	-1.525966000	-1.235140000	2.678450000
6	1.366596000	3.671262000	1.965480000
6	-1.600295000	2.733934000	-3.607064000
6	-3.310541000	-2.885640000	-1.513429000
6	-1.326597000	-1.219212000	4.192720000
6	0.464778000	4.445653000	0.936853000
6	-2.774743000	1.726204000	-3.507918000
6	-3.659399000	-3.105669000	-0.032567000
6	-0.011544000	-0.440670000	4.312481000
6	0.286398000	3.355472000	-0.151363000

6	-2.211761000	0.679610000	-2.564628000
6	-2.662337000	-2.179339000	0.665367000
6	-0.151980000	0.524430000	3.131122000
6	2.865747000	3.720402000	1.557523000
6	-2.258642000	-3.896264000	-2.007450000
6	-1.381147000	-2.602264000	4.823800000
7	0.594498000	2.322923000	-2.769509000
6	3.473839000	5.119307000	1.453504000
6	0.738374000	2.190413000	-4.119011000
6	-2.852688000	-5.269469000	-2.196676000
6	-2.676713000	-3.277138000	4.394046000
6	-2.889663000	-3.321798000	2.882213000
8	2.952646000	6.136432000	1.909506000
8	1.801983000	1.977025000	-4.691048000
8	-4.027838000	-5.568597000	-2.082348000
6	-0.631883000	2.321542000	-4.748338000
6	-5.098318000	-2.670605000	0.341152000
7	4.675470000	5.146398000	0.815004000
8	-1.908506000	-6.176754000	-2.528424000
8	-3.530691000	-4.283608000	2.443030000
6	1.201959000	4.060505000	3.442711000
6	-2.007288000	4.199477000	-3.781456000
6	-6.187019000	-3.425189000	-0.437048000
6	0.236861000	0.244401000	5.663096000
6	-2.121987000	7.011017000	2.294461000
6	-6.473830000	1.730517000	-2.209921000
7	-2.206514000	-1.247170000	-0.201046000
8	-7.656556000	-3.390926000	-2.292324000
8	1.581192000	-1.504937000	6.533279000
6	10.979436000	-2.155541000	-1.257179000
6	10.638483000	-1.618250000	0.140395000
6	9.763525000	-0.353727000	0.123686000
6	8.383083000	-0.473039000	-0.542368000
6	7.350143000	-1.296795000	0.241587000
16	7.531748000	-3.104274000	0.175957000
6	0.614228000	-1.712601000	-2.181739000
8	-0.009036000	-3.229018000	-4.657353000
16	1.239164000	-1.070164000	-0.565895000
6	1.452082000	-1.401345000	-3.410623000
8	1.404221000	-1.694756000	-6.046624000
16	0.471981000	-1.827478000	-4.894050000
8	-0.657765000	-0.840910000	-4.909469000
6	7.810879000	1.471759000	-3.686412000
6	6.472753000	1.479780000	-2.988994000
6	5.803689000	0.278455000	-2.709261000
6	5.860792000	2.674910000	-2.590905000

6	4.577553000	0.258158000	-2.045168000
6	4.629083000	2.677180000	-1.934906000
6	3.986735000	1.466810000	-1.654232000
8	2.787038000	1.526935000	-1.004536000
6	-4.832749000	4.389214000	2.848988000
6	-4.443364000	3.701724000	1.538861000
6	-3.600420000	2.457165000	1.801423000
7	-3.807350000	1.410322000	0.965562000
8	-2.769180000	2.424523000	2.715741000
1	2.786142000	-6.127315000	5.624637000
1	2.738467000	-4.447025000	6.165466000
1	0.141954000	-6.425061000	4.244143000
1	2.849722000	-3.093106000	4.187025000
1	-0.756957000	-5.723870000	2.036934000
1	1.976531000	-2.419358000	1.971480000
1	0.435761000	-2.852548000	0.330925000
1	7.807395000	0.796129000	-4.549903000
1	8.609474000	1.127287000	-3.015898000
1	6.247177000	-0.664393000	-3.021300000
1	6.346766000	3.623333000	-2.808670000
1	4.066715000	-0.679242000	-1.847501000
1	4.140923000	3.609449000	-1.664375000
1	2.373579000	0.617585000	-0.879763000
1	-1.503171000	5.202721000	0.590481000
1	-1.413686000	4.212287000	2.028523000
1	-4.375380000	3.194061000	-3.446404000
1	-3.912736000	2.583205000	-1.860682000
1	-0.474728000	6.129468000	3.299561000
1	-0.060524000	6.965950000	1.825050000
1	-5.556405000	1.121345000	-4.013229000
1	-4.965906000	0.322488000	-2.548257000
1	1.696439000	0.279984000	2.052773000
1	1.812611000	1.372771000	3.439063000
1	-1.833419000	3.354983000	-0.645730000
1	-0.821725000	4.478804000	-1.567114000
1	-3.268493000	-0.976603000	-3.411021000
1	-4.177245000	-2.926357000	-2.172591000
1	-2.142376000	-0.603265000	4.610985000
1	1.020047000	5.287802000	0.515685000
1	-3.005982000	1.295213000	-4.487918000
1	-3.522732000	-4.138170000	0.276525000
1	0.816183000	-1.128505000	4.101266000
1	1.208336000	3.363385000	-0.739223000
1	-0.805368000	1.362492000	3.407216000
1	3.013732000	3.195488000	0.608056000
1	3.454260000	3.159988000	2.296925000

1	-1.799961000	-3.586505000	-2.956776000
1	-1.434617000	-3.978282000	-1.289198000
1	-1.333280000	-2.528758000	5.915360000
1	-0.522797000	-3.191249000	4.492421000
1	-3.535829000	-2.733648000	4.817993000
1	-2.743864000	-4.308125000	4.752278000
1	-0.859650000	1.331227000	-5.156137000
1	-0.613959000	3.038847000	-5.574405000
1	-5.214640000	-1.594538000	0.168427000
1	-5.227933000	-2.850514000	1.409763000
1	0.171506000	3.922976000	3.784337000
1	1.497828000	5.100336000	3.583791000
1	1.847873000	3.445271000	4.078559000
1	-2.579753000	4.318696000	-4.708216000
1	-2.617829000	4.578035000	-2.957099000
1	-1.116265000	4.832647000	-3.860846000
1	-5.819381000	-4.391731000	-0.801400000
1	-7.049267000	-3.648177000	0.203576000
1	-0.614200000	0.867715000	5.950031000
1	1.123870000	0.885926000	5.589787000
1	10.116049000	-2.653370000	-1.711700000
1	11.801948000	-2.878027000	-1.220505000
1	10.154493000	-2.409834000	0.732631000
1	11.566658000	-1.375707000	0.675588000
1	9.626275000	0.004838000	1.153969000
1	10.319749000	0.435624000	-0.402582000
1	7.960672000	0.534340000	-0.648480000
1	8.473598000	-0.873846000	-1.558221000
1	6.355240000	-1.119942000	-0.199090000
1	7.295938000	-0.969430000	1.286195000
1	2.623047000	-5.116090000	-2.241192000
1	-0.372224000	-1.285226000	-2.330113000
1	0.495844000	-2.790974000	-2.091491000
1	1.704274000	-0.341531000	-3.498758000
1	2.374362000	-1.989342000	-3.446787000
1	-5.337071000	3.684091000	3.520720000
1	-3.943398000	4.759596000	3.363869000
1	-5.324688000	3.447163000	0.940913000
1	-3.838774000	4.391363000	0.935843000
1	8.093811000	2.470772000	-4.043424000
1	1.347633000	-5.508963000	6.434687000
1	-5.515325000	5.229565000	2.682747000
6	1.555414000	-5.340890000	-2.272234000
1	1.090601000	-5.014514000	-1.341538000
1	1.414701000	-6.416568000	-2.388347000
1	1.095718000	-4.817003000	-3.110106000

1	-3.022914000	8.387584000	3.215459000
1	-7.052248000	2.054123000	-0.425902000
1	-7.959445000	-2.847341000	-3.045063000
1	-2.360223000	-7.034240000	-2.647285000
1	1.657288000	-2.148205000	7.263816000
1	5.145202000	6.033819000	0.697550000
1	5.077142000	4.328566000	0.377572000
1	-4.536205000	1.418697000	0.261597000
1	-3.249502000	0.571733000	1.071678000
1	1.364014000	2.048324000	-2.161437000
1	11.267433000	-1.332595000	-1.923452000

Int_3
E-Int_3-opt.gjf.log

Temperature 298.150 Kelvin. Pressure 1.00000 Atm.

Zero-point correction= 1.518970 (Hartree/Particle)
Thermal correction to Energy= 1.618704
Thermal correction to Enthalpy= 1.619648
Thermal correction to Gibbs Free Energy= 1.368914
Sum of electronic and zero-point Energies= -5784.772688
Sum of electronic and thermal Energies= -5784.672953
Sum of electronic and thermal Enthalpies= -5784.672009
Sum of electronic and thermal Free Energies= -5784.922743

6	1.629119000	-5.465473000	5.800932000
6	1.229742000	-4.952294000	4.438553000
6	0.216410000	-5.573478000	3.692090000
6	1.879509000	-3.851587000	3.864140000
6	-0.133165000	-5.121464000	2.421331000
6	1.546851000	-3.386519000	2.589972000
6	0.539909000	-4.029708000	1.857198000
8	0.180597000	-3.638560000	0.604162000
6	-0.820437000	5.024254000	1.388506000
6	-3.965443000	2.204803000	-2.918504000
6	-6.453116000	-2.879832000	-1.941331000
6	0.405725000	-0.659344000	6.765097000
7	0.357277000	2.145538000	0.562137000
6	-0.787716000	6.354350000	2.158189000
6	-5.085751000	1.148000000	-2.990039000
7	-1.060538000	1.152152000	-1.898647000
8	-6.140688000	-1.786075000	-2.374728000
8	-0.295143000	-0.782320000	7.749206000
7	-0.818277000	-0.171210000	2.054866000
8	-2.426851000	7.439758000	3.506721000

8	-7.417868000	1.746106000	-2.621984000
8	-3.024660000	6.922678000	1.400016000
8	-6.037257000	1.566734000	-0.854189000
6	1.200129000	1.181801000	2.617774000
6	-0.768954000	3.522050000	-1.210971000
6	-2.499652000	-0.658804000	-2.629083000
6	-2.306972000	-2.137534000	1.967761000
28	-0.600788000	0.297135000	0.030527000
6	0.964833000	2.323145000	1.677524000
6	-0.648787000	2.505894000	-2.363448000
6	-2.470795000	-1.509308000	-1.575000000
6	-1.497601000	-1.142329000	2.620678000
6	1.436623000	3.759561000	1.910319000
6	-1.522105000	2.789735000	-3.665545000
6	-3.016588000	-2.908061000	-1.628220000
6	-1.351904000	-1.105640000	4.140027000
6	0.535439000	4.517746000	0.871196000
6	-2.640206000	1.722144000	-3.568767000
6	-3.448824000	-3.098299000	-0.164542000
6	-0.029305000	-0.344471000	4.290485000
6	0.369415000	3.412107000	-0.203614000
6	-2.006292000	0.685475000	-2.660597000
6	-2.516097000	-2.134116000	0.569432000
6	-0.137424000	0.621672000	3.106343000
6	2.930450000	3.746206000	1.474719000
6	-1.917037000	-3.900620000	-2.047733000
6	-1.458984000	-2.474179000	4.793695000
7	0.706648000	2.452016000	-2.879932000
6	3.574627000	5.112227000	1.250833000
6	0.822484000	2.359881000	-4.233348000
6	-2.464370000	-5.292780000	-2.234472000
6	-2.747926000	-3.132767000	4.320759000
6	-2.886827000	-3.212568000	2.802164000
8	3.148325000	6.163114000	1.729770000
8	1.878151000	2.210811000	-4.843978000
8	-3.633250000	-5.625764000	-2.152525000
6	-0.561279000	2.449188000	-4.834545000
6	-4.917778000	-2.682028000	0.097484000
7	4.692731000	5.061717000	0.478521000
8	-1.483704000	-6.177066000	-2.520184000
8	-3.519555000	-4.175179000	2.354002000
6	1.308697000	4.207527000	3.372849000
6	-2.004942000	4.236698000	-3.798919000
6	-5.937247000	-3.531605000	-0.677636000
6	0.195300000	0.337020000	5.646696000
6	-2.186703000	6.920054000	2.282080000

6	-6.307461000	1.520084000	-2.194810000
7	-2.021366000	-1.217275000	-0.290457000
8	-7.348274000	-3.671831000	-2.571680000
8	1.489764000	-1.438590000	6.539381000
6	11.116654000	-2.342143000	-0.251000000
6	9.732150000	-2.094608000	0.376968000
6	8.550197000	-2.047253000	-0.610901000
6	7.193793000	-1.961916000	0.119891000
6	5.970554000	-2.020087000	-0.817379000
16	4.400089000	-2.185984000	0.131804000
6	0.958720000	-1.657254000	-2.081190000
8	0.434943000	-3.072832000	-4.594135000
16	1.454329000	-0.957684000	-0.447803000
6	1.759512000	-1.186262000	-3.283382000
8	1.832875000	-1.469320000	-5.919044000
16	0.860292000	-1.650006000	-4.807096000
8	-0.309054000	-0.713510000	-4.865215000
6	8.271077000	1.495321000	-2.754898000
6	6.836723000	1.515000000	-2.299213000
6	5.800058000	1.577163000	-3.241280000
6	6.502218000	1.495770000	-0.939364000
6	4.464539000	1.621843000	-2.849621000
6	5.167150000	1.529013000	-0.528079000
6	4.152692000	1.590041000	-1.488227000
8	2.832476000	1.672661000	-1.095026000
6	-4.793656000	4.515084000	2.837794000
6	-4.404383000	3.783742000	1.551884000
6	-3.530017000	2.569328000	1.860419000
7	-3.650337000	1.511842000	1.020278000
8	-2.738854000	2.574033000	2.810204000
1	2.255752000	-6.364566000	5.720069000
1	2.215790000	-4.716534000	6.345502000
1	-0.318434000	-6.419944000	4.117610000
1	2.652404000	-3.333314000	4.426667000
1	-0.937445000	-5.586652000	1.860522000
1	2.061191000	-2.532313000	2.159431000
1	0.618423000	-2.777951000	0.379118000
1	8.404001000	0.852183000	-3.632164000
1	8.941134000	1.134074000	-1.967225000
1	6.043454000	1.589954000	-4.301366000
1	7.290490000	1.435997000	-0.192698000
1	3.663887000	1.691579000	-3.580925000
1	4.904372000	1.479198000	0.524260000
1	2.466408000	0.774159000	-0.830298000
1	-1.462371000	5.202683000	0.521589000
1	-1.320766000	4.257607000	1.989273000

1	-4.307216000	3.107235000	-3.434412000
1	-3.778966000	2.482842000	-1.874799000
1	-0.346282000	6.270383000	3.150807000
1	-0.190991000	7.090723000	1.602276000
1	-5.398199000	0.989998000	-4.025266000
1	-4.740839000	0.191015000	-2.591894000
1	1.758447000	0.389560000	2.105597000
1	1.796270000	1.508998000	3.472720000
1	-1.742005000	3.387920000	-0.727298000
1	-0.732814000	4.533659000	-1.624937000
1	-2.954566000	-1.015544000	-3.545419000
1	-3.846404000	-2.978737000	-2.331008000
1	-2.169269000	-0.465143000	4.516661000
1	1.081078000	5.364833000	0.446707000
1	-2.874190000	1.304932000	-4.554014000
1	-3.312721000	-4.119279000	0.181381000
1	0.795180000	-1.042204000	4.096550000
1	1.300347000	3.402884000	-0.775239000
1	-0.804191000	1.454613000	3.365944000
1	3.043872000	3.148942000	0.566162000
1	3.517376000	3.229926000	2.246717000
1	-1.422555000	-3.595030000	-2.980343000
1	-1.126428000	-3.945474000	-1.290665000
1	-1.455866000	-2.380344000	5.884682000
1	-0.599599000	-3.085528000	4.510354000
1	-3.614018000	-2.561072000	4.689839000
1	-2.853232000	-4.152875000	4.699983000
1	-0.754428000	1.461404000	-5.264008000
1	-0.588584000	3.187849000	-5.641145000
1	-5.052072000	-1.625866000	-0.164463000
1	-5.105482000	-2.784696000	1.167233000
1	0.282985000	4.109349000	3.741038000
1	1.633249000	5.245007000	3.464558000
1	1.953339000	3.603214000	4.020308000
1	-2.606272000	4.345425000	-4.708527000
1	-2.611580000	4.568111000	-2.951786000
1	-1.148794000	4.915011000	-3.887370000
1	-5.509734000	-4.499356000	-0.965716000
1	-6.816014000	-3.758027000	-0.061545000
1	-0.655209000	0.969537000	5.914464000
1	1.090355000	0.969405000	5.594375000
1	11.096637000	-3.222631000	-0.905438000
1	11.861737000	-2.529126000	0.531731000
1	9.536105000	-2.897216000	1.102454000
1	9.753496000	-1.162308000	0.959095000
1	8.659476000	-1.194553000	-1.295308000

1	8.566893000	-2.951551000	-1.236267000
1	7.136134000	-2.792884000	0.836733000
1	7.145013000	-1.037399000	0.709484000
1	6.057703000	-2.864208000	-1.510003000
1	5.909702000	-1.101660000	-1.412522000
1	2.960240000	-4.921919000	-2.037142000
1	-0.082723000	-1.378316000	-2.227346000
1	1.001442000	-2.742629000	-2.011868000
1	1.874268000	-0.101077000	-3.312437000
1	2.749502000	-1.649965000	-3.322849000
1	-5.334139000	3.841954000	3.514130000
1	-3.898843000	4.855534000	3.364722000
1	-5.285085000	3.483618000	0.973140000
1	-3.820035000	4.456735000	0.910923000
1	8.611893000	2.500552000	-3.036780000
1	0.774816000	-5.736048000	6.435012000
1	-5.435430000	5.384052000	2.656817000
6	1.933816000	-5.258689000	-2.190847000
1	1.345608000	-5.026429000	-1.302771000
1	1.923626000	-6.336462000	-2.361132000
1	1.506157000	-4.745078000	-3.051936000
1	-3.339996000	7.787554000	3.494459000
1	-6.870931000	1.788690000	-0.397144000
1	-7.635244000	-3.195951000	-3.374792000
1	-1.906032000	-7.049735000	-2.636504000
1	1.545028000	-2.077561000	7.275499000
1	5.191937000	5.919054000	0.284689000
1	5.007134000	4.208385000	0.033771000
1	-4.367079000	1.464253000	0.304972000
1	-3.080657000	0.691027000	1.185789000
1	1.498308000	2.189145000	-2.290072000
1	11.482777000	-1.494260000	-0.843424000

TS_3
E-TS_3-opt.gjf.log

Temperature 298.150 Kelvin. Pressure 1.00000 Atm.

Zero-point correction= 1.520058 (Hartree/Particle)
Thermal correction to Energy= 1.618944
Thermal correction to Enthalpy= 1.619888
Thermal correction to Gibbs Free Energy= 1.373084
Sum of electronic and zero-point Energies= -5784.762991
Sum of electronic and thermal Energies= -5784.664106
Sum of electronic and thermal Enthalpies= -5784.663162
Sum of electronic and thermal Free Energies= -5784.909966

6	1.680861000	-2.309685000	7.636276000
6	1.373974000	-2.440237000	6.166937000
6	0.868650000	-3.625521000	5.609543000
6	1.677284000	-1.388639000	5.294252000
6	0.699963000	-3.767931000	4.232113000
6	1.515585000	-1.512932000	3.915649000
6	1.048941000	-2.716580000	3.375496000
8	0.922906000	-2.908940000	2.030560000
6	-2.758610000	4.536596000	-0.363543000
6	-3.081436000	-0.098083000	-4.104500000
6	-4.348730000	-5.251166000	-2.001395000
6	-1.477080000	1.497978000	6.598144000
7	-0.290026000	2.415556000	-0.016650000
6	-3.475049000	5.853691000	-0.022318000
6	-3.692928000	-1.512945000	-4.093941000
7	-0.435944000	0.429887000	-2.216932000
8	-4.144012000	-4.384476000	-2.832205000
8	-2.408152000	1.384991000	7.369674000
7	-1.142145000	0.281633000	1.866898000
8	-5.752334000	6.343196000	0.482877000
8	-6.060719000	-1.874482000	-4.537597000
8	-5.348163000	5.297654000	-1.467579000
8	-5.425413000	-1.086542000	-2.528010000
6	0.136573000	2.354234000	2.357999000
6	-1.314717000	2.748243000	-2.283494000
6	-0.937892000	-1.924008000	-2.582833000
6	-2.029156000	-2.003314000	2.117334000
28	-0.495147000	0.296262000	-0.100437000
6	-0.179553000	3.067401000	1.083697000
6	-0.447712000	1.700079000	-3.007026000
6	-1.092778000	-2.417594000	-1.328750000
6	-1.721584000	-0.669548000	2.561777000
6	-0.481253000	4.563972000	0.980818000
6	-0.887668000	1.275685000	-4.474901000
6	-1.362688000	-3.873806000	-1.059645000
6	-2.095507000	-0.199475000	3.965560000
6	-1.225718000	4.609469000	-0.402032000
6	-1.536622000	-0.106037000	-4.262243000
6	-2.194623000	-3.777303000	0.229870000
6	-1.135737000	0.981798000	4.146584000
6	-0.588270000	3.380603000	-1.102107000
6	-0.894163000	-0.543554000	-2.955315000
6	-1.755136000	-2.428119000	0.797962000
6	-1.085272000	1.500110000	2.708044000
6	0.895735000	5.275777000	0.902488000

6	-0.075513000	-4.689211000	-0.865791000
6	-2.062594000	-1.310592000	5.004071000
7	0.891486000	2.215674000	-3.226239000
6	0.833032000	6.788271000	0.673338000
6	1.437820000	1.991921000	-4.459156000
6	-0.376055000	-6.167836000	-0.831658000
6	-2.939538000	-2.451407000	4.504882000
6	-2.590022000	-2.950105000	3.102966000
8	-0.198694000	7.408803000	0.421454000
8	2.522817000	2.408323000	-4.841239000
8	-1.456193000	-6.684088000	-1.055683000
6	0.464570000	1.113815000	-5.213181000
6	-3.724151000	-3.750268000	-0.015226000
7	2.039816000	7.410611000	0.780383000
8	0.718933000	-6.898846000	-0.527415000
8	-2.834634000	-4.139758000	2.867175000
6	-1.266579000	5.078019000	2.199174000
6	-1.743664000	2.316497000	-5.200430000
6	-4.297827000	-5.089036000	-0.499095000
6	-1.555654000	2.030632000	5.184900000
6	-4.929581000	5.786495000	-0.434886000
6	-5.167964000	-1.524471000	-3.798260000
7	-1.173934000	-1.670279000	-0.160846000
8	-4.703816000	-6.506198000	-2.349558000
8	-0.214303000	1.129350000	6.919637000
6	10.767435000	0.238184000	0.755772000
6	9.370625000	0.514866000	1.352739000
6	8.164441000	0.032232000	0.519244000
6	6.843607000	0.044460000	1.326812000
6	5.622436000	-0.375084000	0.485643000
16	4.149143000	-0.740590000	1.560911000
6	2.089267000	-1.616227000	-0.893385000
8	2.368266000	-3.808944000	-3.099329000
16	2.111521000	-0.337664000	0.440685000
6	3.418811000	-1.932021000	-1.553801000
8	4.489152000	-2.691400000	-3.833897000
16	3.127567000	-2.525565000	-3.250523000
8	2.325211000	-1.439051000	-3.900400000
6	7.394844000	1.416722000	-3.282287000
6	6.127758000	1.685318000	-2.520090000
6	4.956613000	0.988810000	-2.825859000
6	6.070921000	2.709580000	-1.563040000
6	3.754280000	1.303970000	-2.200166000
6	4.873147000	3.032964000	-0.926238000
6	3.714728000	2.327140000	-1.252933000
8	2.525988000	2.680962000	-0.639518000

6	-6.647411000	2.709306000	0.213914000
6	-5.603900000	2.011509000	-0.657000000
6	-4.484882000	1.404759000	0.189700000
7	-3.896027000	0.295190000	-0.316260000
8	-4.114021000	1.927022000	1.247968000
1	2.469661000	-3.016242000	7.929482000
1	2.060643000	-1.305985000	7.859710000
1	0.610884000	-4.456909000	6.262037000
1	2.045324000	-0.448301000	5.696548000
1	0.302451000	-4.683312000	3.804577000
1	1.754916000	-0.684363000	3.259695000
1	1.108033000	-2.059322000	1.573615000
1	7.364357000	0.459483000	-3.810581000
1	8.284361000	1.436681000	-2.642287000
1	4.972964000	0.179859000	-3.551972000
1	6.976120000	3.257870000	-1.311318000
1	2.860474000	0.746778000	-2.450820000
1	4.823641000	3.814473000	-0.173636000
1	2.068105000	1.852482000	-0.372167000
1	-3.102841000	4.262257000	-1.364618000
1	-3.101002000	3.744192000	0.308393000
1	-3.517977000	0.458201000	-4.939769000
1	-3.352742000	0.436858000	-3.187554000
1	-3.417079000	6.113762000	1.034046000
1	-3.017376000	6.682219000	-0.579567000
1	-3.544764000	-2.000727000	-5.060714000
1	-3.222772000	-2.131666000	-3.327193000
1	1.006812000	1.702188000	2.231193000
1	0.353907000	3.061117000	3.162242000
1	-2.247177000	2.273671000	-1.962108000
1	-1.561864000	3.553690000	-2.980777000
1	-0.974442000	-2.634899000	-3.400835000
1	-1.922899000	-4.308722000	-1.885380000
1	-3.124970000	0.195334000	3.899669000
1	-0.943880000	5.515991000	-0.942214000
1	-1.284147000	-0.788426000	-5.082188000
1	-1.975843000	-4.581440000	0.928133000
1	-0.147127000	0.591881000	4.412692000
1	0.383021000	3.725393000	-1.465861000
1	-2.000381000	2.074350000	2.501449000
1	1.508056000	4.835101000	0.106047000
1	1.450742000	5.090577000	1.831761000
1	0.644404000	-4.500375000	-1.674356000
1	0.430101000	-4.416642000	0.067378000
1	-2.424804000	-0.944921000	5.970757000
1	-1.035938000	-1.657742000	5.143727000

1	-3.989847000	-2.121715000	4.469160000
1	-2.902763000	-3.322014000	5.165789000
1	0.860251000	0.093463000	-5.127196000
1	0.416464000	1.377874000	-6.272658000
1	-3.971189000	-2.965674000	-0.740462000
1	-4.207246000	-3.489910000	0.928104000
1	-2.201614000	4.528488000	2.341171000
1	-1.489705000	6.138026000	2.075938000
1	-0.674296000	4.970004000	3.114217000
1	-2.004444000	1.953546000	-6.200850000
1	-2.672604000	2.548941000	-4.673018000
1	-1.182504000	3.250111000	-5.322718000
1	-3.738631000	-5.936567000	-0.091215000
1	-5.332782000	-5.208256000	-0.151384000
1	-2.578885000	2.372723000	5.008453000
1	-0.886651000	2.897720000	5.118879000
1	10.890125000	-0.832748000	0.549511000
1	11.545720000	0.525661000	1.473147000
1	9.326440000	0.022530000	2.335216000
1	9.260049000	1.590073000	1.552107000
1	8.051569000	0.655920000	-0.377364000
1	8.350730000	-0.991514000	0.164285000
1	6.958714000	-0.633310000	2.184447000
1	6.667039000	1.046210000	1.740490000
1	5.851973000	-1.276671000	-0.089674000
1	5.357009000	0.417174000	-0.212112000
1	2.795856000	-4.325432000	0.898382000
1	1.416266000	-1.185458000	-1.633583000
1	1.633570000	-2.524406000	-0.503773000
1	4.046190000	-1.052113000	-1.653301000
1	3.976032000	-2.699871000	-1.016292000
1	-7.098075000	1.999647000	0.918323000
1	-6.176921000	3.498450000	0.806407000
1	-6.056290000	1.243849000	-1.294685000
1	-5.138649000	2.744182000	-1.329213000
1	7.498012000	2.220356000	-4.024541000
1	0.847708000	-2.488529000	8.328183000
1	-7.461968000	3.158229000	-0.364625000
6	3.357725000	-5.005486000	0.257861000
1	3.111535000	-6.033130000	0.522277000
1	4.426032000	-4.830720000	0.393926000
1	3.085368000	-4.830100000	-0.782720000
1	-6.661593000	6.266390000	0.133266000
1	-6.389815000	-1.144062000	-2.387940000
1	-4.734468000	-6.533437000	-3.325275000
1	0.450465000	-7.837605000	-0.544138000

1	-0.241301000	0.756681000	7.821626000
1	2.099049000	8.385178000	0.516852000
1	2.902130000	6.888267000	0.847392000
1	-4.276114000	-0.203731000	-1.112000000
1	-3.115539000	-0.114133000	0.182220000
1	1.452206000	2.612128000	-2.482542000
1	10.979245000	0.774861000	-0.176695000

PC
E-PC-opt.gjf.log

Temperature 298.150 Kelvin. Pressure 1.00000 Atm.

Zero-point correction= 1.517724 (Hartree/Particle)
Thermal correction to Energy= 1.618089
Thermal correction to Enthalpy= 1.619034
Thermal correction to Gibbs Free Energy= 1.366016
Sum of electronic and zero-point Energies= -5784.780052
Sum of electronic and thermal Energies= -5784.679687
Sum of electronic and thermal Enthalpies= -5784.678743
Sum of electronic and thermal Free Energies= -5784.931760

6	1.059462000	-2.638489000	7.420895000
6	0.606592000	-2.570492000	5.982317000
6	-0.502112000	-3.298494000	5.523227000
6	1.328933000	-1.821420000	5.043907000
6	-0.874658000	-3.285961000	4.179666000
6	0.978284000	-1.807160000	3.692660000
6	-0.127109000	-2.544382000	3.257321000
8	-0.515434000	-2.587696000	1.950895000
6	-0.399231000	4.691720000	-0.914685000
6	-2.752535000	0.759440000	-4.467380000
6	-6.731620000	-2.116609000	-1.466348000
6	0.193549000	2.464071000	6.275579000
7	0.945195000	1.894999000	-0.478473000
6	-0.587163000	6.191446000	-0.631611000
6	-4.132556000	0.144722000	-4.156492000
7	-0.574862000	0.074002000	-2.180598000
8	-6.119541000	-1.413651000	-2.248770000
8	-0.534715000	2.924078000	7.131402000
7	-0.858187000	0.900035000	1.751359000
8	-2.480144000	7.370361000	0.217991000
8	-6.151593000	1.332547000	-4.825849000
8	-2.772368000	6.204248000	-1.684343000
8	-5.035789000	2.004685000	-2.989807000
6	1.413366000	1.923352000	1.884813000

6	0.125507000	2.358872000	-2.793658000
6	-2.296316000	-1.624311000	-2.277102000
6	-2.797345000	-0.486146000	2.399733000
28	-0.619918000	0.389643000	-0.188315000
6	1.419699000	2.539490000	0.526041000
6	0.258387000	0.857521000	-3.123852000
6	-2.611553000	-1.744009000	-0.959334000
6	-1.754937000	0.478935000	2.622012000
6	1.866309000	3.972226000	0.213402000
6	-0.178642000	0.386396000	-4.575372000
6	-3.540080000	-2.804154000	-0.430072000
6	-1.597036000	1.156837000	3.980729000
6	1.048902000	4.206526000	-1.108343000
6	-1.572139000	-0.236800000	-4.309593000
6	-4.097512000	-2.099194000	0.817905000
6	-0.150376000	1.652176000	3.907104000
6	1.075780000	2.764539000	-1.677376000
6	-1.452312000	-0.631276000	-2.850057000
6	-2.972712000	-1.120860000	1.147709000
6	-0.028568000	1.937682000	2.408615000
6	3.391953000	3.884600000	-0.056842000
6	-2.756670000	-4.079055000	-0.073763000
6	-1.962413000	0.239965000	5.139867000
7	1.643263000	0.400595000	-3.033035000
6	4.070866000	5.155363000	-0.566508000
6	2.004249000	-0.538000000	-3.955005000
6	-3.663454000	-5.188973000	0.388612000
6	-3.387689000	-0.247053000	4.919749000
6	-3.648305000	-0.864830000	3.544554000
8	3.483753000	6.208493000	-0.809020000
8	3.072797000	-1.148471000	-3.959552000
8	-4.880749000	-5.195842000	0.342443000
6	0.855917000	-0.717698000	-4.919561000
6	-5.395830000	-1.296802000	0.554044000
7	5.416003000	5.015546000	-0.727457000
8	-2.960134000	-6.237927000	0.874710000
8	-4.600611000	-1.652721000	3.477163000
6	1.607243000	4.948769000	1.370078000
6	-0.135233000	1.479614000	-5.646201000
6	-6.554699000	-2.161601000	0.035714000
6	0.193632000	2.843381000	4.811821000
6	-2.047355000	6.564250000	-0.775459000
6	-5.216618000	1.185330000	-4.068460000
7	-2.202951000	-0.900205000	0.060843000
8	-7.700202000	-2.967578000	-1.874008000
8	1.115078000	1.506654000	6.539408000

6	10.158544000	-2.672876000	0.099387000
6	8.792093000	-2.209208000	0.625944000
6	7.570849000	-2.684414000	-0.181407000
6	6.265768000	-2.425576000	0.593023000
6	4.996719000	-2.849208000	-0.152502000
16	3.521544000	-2.679532000	0.973704000
6	1.023200000	-2.811764000	-0.946354000
8	-0.334503000	-5.310499000	-2.047554000
16	2.081470000	-1.578181000	-0.078433000
6	1.740410000	-3.655693000	-1.983821000
8	1.363938000	-5.440576000	-3.883980000
16	0.535737000	-4.564086000	-3.011991000
8	-0.217066000	-3.504118000	-3.756520000
6	7.158352000	-0.234459000	-3.659379000
6	6.680971000	0.352339000	-2.347623000
6	5.345035000	0.223850000	-1.945217000
6	7.565109000	1.010562000	-1.478306000
6	4.913333000	0.694931000	-0.703265000
6	7.148158000	1.490376000	-0.234889000
6	5.820431000	1.303704000	0.167883000
8	5.468060000	1.728365000	1.427811000
6	-5.555948000	5.218435000	0.776305000
6	-4.953838000	4.228800000	-0.229958000
6	-3.748089000	3.458782000	0.319037000
7	-3.521196000	2.250184000	-0.251416000
8	-3.016953000	3.924085000	1.201692000
1	1.596256000	-3.576825000	7.617899000
1	1.753303000	-1.821417000	7.649958000
1	-1.089029000	-3.881294000	6.229557000
1	2.183237000	-1.233880000	5.371094000
1	-1.744766000	-3.832579000	3.828811000
1	1.557507000	-1.235229000	2.974431000
1	-0.134667000	-1.840082000	1.452162000
1	6.362959000	-0.812310000	-4.140214000
1	8.013679000	-0.903350000	-3.503128000
1	4.629404000	-0.269371000	-2.599555000
1	8.607765000	1.130234000	-1.764561000
1	3.880315000	0.550272000	-0.398919000
1	7.846576000	1.964500000	0.448337000
1	4.606466000	1.346267000	1.660808000
1	-0.945929000	4.496610000	-1.841099000
1	-0.906867000	4.109159000	-0.136922000
1	-2.762659000	1.141632000	-5.493673000
1	-2.585776000	1.617679000	-3.807118000
1	-0.236934000	6.495527000	0.353469000
1	-0.028925000	6.781083000	-1.371637000

1	-4.419877000	-0.577423000	-4.924751000
1	-4.116769000	-0.373035000	-3.195080000
1	1.752939000	0.883629000	1.829584000
1	2.065226000	2.471729000	2.570901000
1	0.363124000	2.973049000	-3.666397000
1	-2.744782000	-2.329733000	-2.966099000
1	-4.308789000	-3.056355000	-1.161301000
1	-2.271929000	2.030696000	3.991378000
1	1.579589000	4.898394000	-1.767521000
1	-1.752112000	-1.106698000	-4.950507000
1	-4.285723000	-2.790469000	1.635875000
1	0.515493000	0.816754000	4.151112000
1	2.083245000	2.595264000	-2.078570000
1	-0.489283000	2.912756000	2.185496000
1	3.614285000	3.092997000	-0.781410000
1	3.905060000	3.578334000	0.862640000
1	-2.173562000	-4.449971000	-0.928336000
1	-2.021685000	-3.874138000	0.711693000
1	-1.882919000	0.770664000	6.094756000
1	-1.270192000	-0.607496000	5.171445000
1	-4.086880000	0.597416000	5.021193000
1	-3.691079000	-0.994014000	5.659517000
1	0.468216000	-1.727655000	-4.732300000
1	1.204230000	-0.676722000	-5.955861000
1	-5.193140000	-0.495514000	-0.165354000
1	-5.683856000	-0.829690000	1.497296000
1	0.563094000	4.934564000	1.693245000
1	1.874827000	5.961604000	1.064939000
1	2.225232000	4.689508000	2.237109000
1	-0.442199000	1.063581000	-6.612437000
1	-0.788625000	2.327161000	-5.424539000
1	0.886459000	1.861065000	-5.757902000
1	-6.428476000	-3.212425000	0.321363000
1	-7.511708000	-1.840584000	0.466708000
1	-0.518209000	3.661511000	4.673802000
1	1.196398000	3.214619000	4.565584000
1	10.154059000	-3.753024000	-0.094967000
1	10.936732000	-2.481479000	0.848983000
1	8.684331000	-2.581696000	1.655034000
1	8.767270000	-1.113133000	0.696345000
1	7.535538000	-2.171909000	-1.151765000
1	7.663001000	-3.759848000	-0.391318000
1	6.319441000	-2.963105000	1.550892000
1	6.190632000	-1.359398000	0.835144000
1	5.048289000	-3.893839000	-0.476500000
1	4.819681000	-2.223314000	-1.029500000

1	0.221566000	-5.044188000	1.761573000
1	0.271661000	-2.173578000	-1.419027000
1	0.524877000	-3.434609000	-0.203197000
1	2.334272000	-3.045468000	-2.668679000
1	2.386663000	-4.401817000	-1.512241000
1	-6.131637000	4.704255000	1.553244000
1	-4.751142000	5.773066000	1.269285000
1	-5.712815000	3.524011000	-0.590167000
1	-4.581589000	4.777771000	-1.103842000
1	7.480283000	0.540294000	-4.369122000
1	0.245410000	-2.587054000	8.155470000
1	-6.209706000	5.937977000	0.271759000
6	0.672246000	-5.894519000	1.251574000
1	0.307596000	-6.821385000	1.697103000
1	1.756921000	-5.840585000	1.356107000
1	0.401208000	-5.861204000	0.196674000
1	-3.422637000	7.559093000	0.042584000
1	-5.757461000	2.661757000	-3.003552000
1	-7.764917000	-2.881104000	-2.844634000
1	-3.607069000	-6.923339000	1.129927000
1	1.035049000	1.281326000	7.485983000
1	5.932587000	5.774305000	-1.151111000
1	5.875837000	4.118172000	-0.636781000
1	-4.038298000	1.938534000	-1.065212000
1	-2.682606000	1.732135000	-0.000285000
1	10.487856000	-2.173485000	-0.819577000
1	-0.916511000	2.542595000	-2.521417000
1	2.111147000	0.395347000	-2.133886000

ii) Reactant, transition state and product ($RC_{uncatalyzed}$, $TS_{uncatalyzed}$ and $PC_{uncatalyzed}$) optimized for uncatalyzed reaction.

$RC_{uncatalyzed}$
RC-uncatalyzed-opt.gjf.log

Temperature 298.150 Kelvin. Pressure 1.00000 Atm.

Zero-point correction= 0.258282 (Hartree/Particle)
Thermal correction to Energy= 0.280198
Thermal correction to Enthalpy= 0.281142
Thermal correction to Gibbs Free Energy= 0.196120
Sum of electronic and zero-point Energies= -1736.493572
Sum of electronic and thermal Energies= -1736.471656
Sum of electronic and thermal Enthalpies= -1736.470712
Sum of electronic and thermal Free Energies= -1736.555733

6	3.048676000	1.423431000	-1.393069000
6	3.838931000	0.822545000	-0.226151000
6	3.088050000	0.850239000	1.115119000
6	1.786480000	0.035522000	1.200294000
6	1.937109000	-1.487549000	1.077039000
16	1.996909000	-2.169999000	-0.647984000
6	-2.222789000	1.267634000	0.409528000
8	-1.282638000	-1.535058000	1.271409000
16	-2.011043000	3.017499000	-0.100807000
6	-1.742390000	0.256407000	-0.623815000
8	-1.357475000	-2.301829000	-1.103722000
16	-1.989723000	-1.450260000	-0.041261000
8	-3.465782000	-1.632940000	0.059677000
1	2.139457000	0.846392000	-1.594281000
1	3.644983000	1.431376000	-2.313198000
1	4.125007000	-0.207710000	-0.471830000
1	4.777683000	1.380188000	-0.103723000
1	3.764855000	0.498206000	1.907123000
1	2.851996000	1.896645000	1.359506000
1	1.334619000	0.230359000	2.183218000
1	1.055501000	0.390161000	0.467306000
1	1.093155000	-1.977908000	1.562405000
1	2.860828000	-1.825269000	1.559193000
1	0.654133000	-2.193986000	-0.865268000
1	-3.295028000	1.139002000	0.585567000
1	-1.703816000	1.110980000	1.359691000
1	-2.286063000	0.355399000	-1.567325000
1	-0.673337000	0.357015000	-0.826365000
6	-0.187973000	3.151463000	-0.068534000
1	0.272619000	2.551583000	-0.850870000
1	0.061052000	4.198454000	-0.237995000
1	0.200591000	2.845663000	0.903063000
1	2.754078000	2.458088000	-1.176761000

$TS_{uncatalyzed}$
TS-uncatalyzed-opt.gjf.log

Temperature 298.150 Kelvin. Pressure 1.00000 Atm.

Zero-point correction= 0.268074 (Hartree/Particle)
Thermal correction to Energy= 0.287760
Thermal correction to Enthalpy= 0.288705
Thermal correction to Gibbs Free Energy= 0.215997
Sum of electronic and zero-point Energies= -1736.365041
Sum of electronic and thermal Energies= -1736.345354
Sum of electronic and thermal Enthalpies= -1736.344410

Sum of electronic and thermal Free Energies= -1736.417118

6	5.280247000	-0.058942000	1.002095000
6	4.833949000	0.091966000	-0.456327000
6	3.773669000	-0.927046000	-0.898008000
6	2.415824000	-0.846885000	-0.185969000
6	1.559973000	0.390035000	-0.534968000
16	2.099076000	1.960587000	0.198072000
6	-3.543516000	0.268238000	0.222900000
8	-0.868994000	-0.921067000	1.195429000
16	-3.932290000	1.969746000	-0.296984000
6	-2.590404000	-0.462526000	-0.731292000
8	-0.720663000	-2.320254000	-0.869305000
16	-1.615013000	-1.714331000	0.159819000
8	-2.598979000	-2.665615000	0.751453000
1	4.452295000	0.116397000	1.697759000
1	6.074481000	0.655656000	1.246713000
1	4.453055000	1.114152000	-0.618009000
1	5.707115000	-0.004446000	-1.115877000
1	3.607654000	-0.829548000	-1.980127000
1	4.177967000	-1.937981000	-0.739837000
1	1.798542000	-1.703087000	-0.483767000
1	2.534249000	-0.912214000	0.901793000
1	0.561109000	0.193163000	-0.120984000
1	1.475376000	0.506236000	-1.621055000
1	0.144710000	2.664243000	0.337331000
1	-4.486242000	-0.283428000	0.318273000
1	-3.105189000	0.317626000	1.225368000
1	-3.128713000	-0.958731000	-1.543141000
1	-1.857245000	0.217585000	-1.169984000
6	-0.985314000	2.565053000	0.271430000
1	-1.213534000	2.565229000	-0.791799000
1	-1.430744000	3.412512000	0.788555000
1	-1.217418000	1.615625000	0.745030000
1	5.666669000	-1.068891000	1.189460000

$PC_{uncatalyzed}$
PC-uncatalyzed-opt.gjf.log

Temperature 298.150 Kelvin. Pressure 1.00000 Atm.

Zero-point correction= 0.258162 (Hartree/Particle)
Thermal correction to Energy= 0.281268
Thermal correction to Enthalpy= 0.282212
Thermal correction to Gibbs Free Energy= 0.192449
Sum of electronic and zero-point Energies= -1736.496894

Sum of electronic and thermal Energies= -1736.473788
Sum of electronic and thermal Enthalpies= -1736.472844
Sum of electronic and thermal Free Energies= -1736.562607

6	2.949810000	-2.163925000	0.740960000
6	3.906958000	-1.159510000	0.088707000
6	3.618153000	-0.898331000	-1.399124000
6	2.246145000	-0.292106000	-1.739425000
6	2.029429000	1.160063000	-1.293196000
16	1.687231000	1.372601000	0.530364000
6	-1.411917000	1.324998000	0.648143000
8	-2.625280000	-1.569471000	0.678355000
16	-0.038328000	2.554609000	0.602966000
6	-1.510945000	0.461507000	-0.598202000
8	-2.896795000	-1.424267000	-1.800715000
16	-2.915738000	-0.694437000	-0.499721000
8	-4.120543000	0.166963000	-0.308481000
1	1.920827000	-1.790566000	0.754254000
1	3.238321000	-2.365387000	1.779121000
1	3.889053000	-0.214326000	0.649713000
1	4.935874000	-1.533867000	0.174614000
1	4.398139000	-0.239086000	-1.805643000
1	3.709010000	-1.850885000	-1.939718000
1	2.140754000	-0.292816000	-2.833141000
1	1.436600000	-0.924930000	-1.356532000
1	1.166319000	1.591760000	-1.805619000
1	2.904783000	1.772597000	-1.533253000
1	0.514196000	-2.359251000	3.177994000
1	-2.313343000	1.937400000	0.760758000
1	-1.301765000	0.705808000	1.540448000
1	-1.655538000	1.068128000	-1.496842000
1	-0.614556000	-0.150951000	-0.726452000
6	-0.037596000	-1.430722000	3.026258000
1	0.650162000	-0.659424000	2.679669000
1	-0.490428000	-1.119911000	3.969108000
1	-0.815771000	-1.578827000	2.277756000
1	2.957423000	-3.117467000	0.198004000

(iii) Complexes considered in proposed thermodynamic cycle of the first catalytic step.

a) CH_3-SCoM, CH_3-radical, SCoM-radical and SCoM-anion.

CH_3-SCoM
CH_3-SCoM-opt.gjf.log

Temperature 298.150 Kelvin. Pressure 1.00000 Atm.

Zero-point correction= 0.108695 (Hartree/Particle)
Thermal correction to Energy= 0.118183
Thermal correction to Enthalpy= 0.119127
Thermal correction to Gibbs Free Energy= 0.072518
Sum of electronic and zero-point Energies= -1140.607933
Sum of electronic and thermal Energies= -1140.598445
Sum of electronic and thermal Enthalpies= -1140.597500
Sum of electronic and thermal Free Energies= -1140.644109

6	0.918499000	0.618548000	0.351404000
8	-1.707355000	-0.713251000	1.354849000
16	2.684533000	0.607887000	-0.157341000
6	-0.013515000	-0.139142000	-0.584022000
8	-2.519606000	-0.820068000	-1.009192000
16	-1.739357000	-0.061826000	0.010914000
8	-2.060436000	1.395807000	0.060059000
1	0.627270000	1.672459000	0.386004000
1	0.842162000	0.216142000	1.366228000
1	0.003187000	0.285393000	-1.591941000
1	0.242623000	-1.200744000	-0.646099000
6	3.082054000	-1.158896000	0.091823000
1	2.551251000	-1.792165000	-0.618230000
1	4.154250000	-1.276946000	-0.063281000
1	2.833392000	-1.464094000	1.109188000

CH_3-radical
CH_3-radical-opt.gjf.log

Temperature 298.150 Kelvin. Pressure 1.00000 Atm.

Zero-point correction= 0.029460 (Hartree/Particle)
Thermal correction to Energy= 0.032552
Thermal correction to Enthalpy= 0.033497
Thermal correction to Gibbs Free Energy= 0.009647
Sum of electronic and zero-point Energies= -39.819671
Sum of electronic and thermal Energies= -39.816578
Sum of electronic and thermal Enthalpies= -39.815634
Sum of electronic and thermal Free Energies= -39.839484

6	-0.000001000	-0.000022000	-0.000199000
1	0.922247000	-0.564634000	0.000399000
1	-0.950191000	-0.516232000	0.000399000
1	0.027950000	1.080996000	0.000399000

SCoM-radical
SCoM-radical-opt.gjf.log

Temperature 298.150 Kelvin. Pressure 1.00000 Atm.

Zero-point correction= 0.068871 (Hartree/Particle)
Thermal correction to Energy= 0.076456
Thermal correction to Enthalpy= 0.077401
Thermal correction to Gibbs Free Energy= 0.034821
Sum of electronic and zero-point Energies= -1100.681361
Sum of electronic and thermal Energies= -1100.673775
Sum of electronic and thermal Enthalpies= -1100.672831
Sum of electronic and thermal Free Energies= -1100.715411

6	1.357671000	-0.420942000	0.000108000
8	-1.425645000	-0.826895000	-1.249453000
16	3.090058000	0.116913000	-0.000029000
6	0.322200000	0.694781000	0.000220000
8	-2.270614000	1.171085000	-0.000601000
16	-1.362269000	-0.010374000	-0.000011000
8	-1.426346000	-0.826194000	1.249813000
1	1.215187000	-1.070379000	0.874863000
1	1.215173000	-1.070101000	-0.874885000
1	0.413225000	1.324319000	0.889832000
1	0.413403000	1.324544000	-0.889207000

SCoM-anion

SCoM-anion-opt.gjf.log

Temperature 298.150 Kelvin. Pressure 1.00000 Atm.

Zero-point correction= 0.070035 (Hartree/Particle)
Thermal correction to Energy= 0.077423
Thermal correction to Enthalpy= 0.078368
Thermal correction to Gibbs Free Energy= 0.036923
Sum of electronic and zero-point Energies= -1100.801029
Sum of electronic and thermal Energies= -1100.793641
Sum of electronic and thermal Enthalpies= -1100.792697
Sum of electronic and thermal Free Energies= -1100.834141

6	1.391729000	-0.504391000	0.000149000
8	-1.522555000	-0.811286000	-1.248945000
16	3.141533000	0.136692000	-0.000030000
6	0.347541000	0.608435000	-0.000828000
8	-2.216381000	1.238993000	-0.000941000

16	-1.373016000	0.001002000	-0.000019000
8	-1.521822000	-0.808609000	1.250745000
1	1.226968000	-1.133025000	0.881320000
1	1.227433000	-1.133996000	-0.880440000
1	0.449947000	1.244277000	0.884717000
1	0.449826000	1.242595000	-0.887601000

b) Ni^{I}, Ni^{II} and Ni^{II}-SCoM for **A**.

NiI-A

NiI-A-opt.gjf.log

Temperature 298.150 Kelvin. Pressure 1.00000 Atm.

Zero-point correction= 0.839162 (Hartree/Particle)
Thermal correction to Energy= 0.900511
Thermal correction to Enthalpy= 0.901455
Thermal correction to Gibbs Free Energy= 0.727098
Sum of electronic and zero-point Energies= -3218.519819
Sum of electronic and thermal Energies= -3218.458469
Sum of electronic and thermal Enthalpies= -3218.457525
Sum of electronic and thermal Free Energies= -3218.631882

6	5.042923000	-1.311170000	1.039976000
6	1.909202000	4.495215000	1.424308000
6	-7.563337000	0.927317000	-1.122287000
6	-0.802230000	-6.076067000	-0.924777000
7	1.859058000	-1.072866000	-0.104385000
6	6.417113000	-1.985054000	1.018140000
6	0.735404000	5.190423000	2.138020000
7	1.073110000	1.749147000	-0.139582000
8	-8.290252000	0.988778000	-0.149743000
8	0.328271000	-6.347544000	-1.281907000
7	-0.944301000	-1.802361000	0.217870000
8	8.455868000	-2.248473000	2.197317000
8	1.110424000	6.790018000	3.930098000
8	7.045846000	-0.672849000	2.972571000
8	1.447859000	4.597848000	4.321219000
6	0.981037000	-3.337349000	0.117004000
6	3.316292000	0.849693000	-0.468777000
6	-0.838986000	3.273633000	0.000335000
6	-3.247293000	-0.938545000	0.308487000
28	0.065562000	-0.034831000	0.036353000
6	2.000444000	-2.443589000	-0.064340000
6	2.389918000	1.884308000	-0.407802000

6	-1.895527000	2.328708000	0.076287000
6	-2.317483000	-1.976046000	0.309949000
6	3.440977000	-2.875216000	-0.394448000
6	2.755035000	3.342409000	-0.728165000
6	-3.299720000	2.660821000	0.150501000
6	-2.640262000	-3.381039000	0.397979000
6	4.207576000	-1.522696000	-0.242772000
6	1.577811000	4.080397000	-0.029305000
6	-3.988305000	1.468097000	0.242400000
6	-1.434768000	-4.050237000	0.376783000
6	3.067235000	-0.510570000	-0.286184000
6	0.502045000	3.004317000	-0.065414000
6	-2.987570000	0.428476000	0.226554000
6	-0.401712000	-3.047790000	0.247070000
6	3.432818000	-3.305519000	-1.883615000
6	-3.886222000	4.048132000	0.094560000
6	-4.019045000	-3.958518000	0.536592000
6	4.778266000	-3.681349000	-2.468270000
6	2.931827000	4.868428000	-2.788032000
6	-3.964746000	4.524518000	-1.343941000
6	-4.542591000	-3.865640000	1.978660000
8	5.871662000	-3.525811000	-1.958347000
8	4.041954000	5.292416000	-3.045594000
8	-4.809581000	4.189734000	-2.152657000
6	2.618776000	3.480001000	-2.287024000
6	-5.474925000	1.267654000	0.281744000
8	-2.946777000	5.355066000	-1.665067000
6	3.917585000	-4.048221000	0.476546000
6	4.164119000	3.755759000	-0.286441000
6	-6.068042000	1.127766000	-1.129649000
6	-1.198299000	-5.538591000	0.437329000
6	7.297133000	-1.544592000	2.164469000
6	1.104060000	5.642440000	3.529109000
7	-1.720201000	0.981606000	0.125594000
8	-8.039368000	0.671440000	-2.365540000
8	-1.871108000	-6.191916000	-1.746069000
1	5.200413000	-0.235112000	1.166643000
1	4.475292000	-1.632041000	1.921417000
1	2.765264000	5.179817000	1.414111000
1	2.207832000	3.612312000	2.000376000
1	6.353974000	-3.075526000	1.044864000
1	6.946217000	-1.753580000	0.084486000
1	0.406125000	6.072116000	1.580723000
1	-0.109279000	4.497072000	2.212564000
1	1.240387000	-4.389487000	0.088776000
1	4.348176000	1.121866000	-0.667111000

1	-1.122860000	4.320983000	-0.012119000
1	4.878627000	-1.364838000	-1.093398000
1	1.273244000	4.974708000	-0.582349000
1	3.032825000	-2.492071000	-2.504681000
1	2.750536000	-4.151310000	-2.028208000
1	-4.907653000	4.043523000	0.486949000
1	-3.295000000	4.757801000	0.681551000
1	-4.029693000	-5.006187000	0.217656000
1	-4.722796000	-3.440354000	-0.125491000
1	-3.881914000	-4.404523000	2.669391000
1	3.327800000	2.793159000	-2.756459000
1	1.603275000	3.200943000	-2.581666000
1	-5.964640000	2.111381000	0.782146000
1	-5.733435000	0.380340000	0.870743000
1	3.901849000	-3.791578000	1.539599000
1	4.927437000	-4.358338000	0.201018000
1	3.261811000	-4.914084000	0.337795000
1	4.312600000	4.834547000	-0.395023000
1	4.349770000	3.480721000	0.755228000
1	4.923360000	3.265640000	-0.904155000
1	-5.846571000	2.027475000	-1.717976000
1	-5.606158000	0.290691000	-1.666905000
1	-2.104716000	-6.054637000	0.768565000
1	-0.390133000	-5.786746000	1.131734000
1	8.974578000	-1.902655000	2.948866000
1	1.681431000	4.967213000	5.194250000
1	-9.006748000	0.568173000	-2.281685000
1	-3.030983000	5.559267000	-2.616729000
1	-1.534370000	-6.485354000	-2.614376000
1	5.530833000	-4.424027000	-4.029432000
1	2.120631000	6.526897000	-3.181189000
1	-4.292909000	-1.227421000	0.375549000
1	-4.544174000	-2.824760000	2.324895000
6	-5.940247000	-4.416427000	2.137402000
8	-6.625954000	-4.891721000	1.254223000
8	-6.369767000	-4.322156000	3.419100000
1	-7.274104000	-4.689931000	3.442763000
8	1.822488000	5.641049000	-2.896591000
8	4.633028000	-4.226030000	-3.699491000

NiII-A
NiII-A-opt.gjf.log

Temperature 298.150 Kelvin. Pressure 1.00000 Atm.

Zero-point correction= 0.844882 (Hartree/Particle)
Thermal correction to Energy= 0.904082
Thermal correction to Enthalpy= 0.905026
Thermal correction to Gibbs Free Energy= 0.742070
Sum of electronic and zero-point Energies= -3218.437295
Sum of electronic and thermal Energies= -3218.378095
Sum of electronic and thermal Enthalpies= -3218.377151
Sum of electronic and thermal Free Energies= -3218.540106

28	-0.215413000	-0.357028000	-0.423306000
7	0.617096000	1.382855000	-0.563139000
7	1.547156000	-1.202226000	-0.185335000
7	-1.053891000	-2.110492000	-0.344917000
7	-1.965041000	0.475538000	-0.498079000
6	0.029952000	2.624746000	-0.334971000
6	1.001232000	3.665970000	-0.531366000
6	1.912713000	1.629571000	-0.893714000
6	2.164671000	3.044400000	-0.932204000
6	-3.158771000	-0.148306000	-0.685664000
6	-2.265498000	1.788082000	-0.144164000
6	-3.686579000	1.954500000	-0.043178000
6	-4.243347000	0.741721000	-0.401922000
6	-1.316151000	2.798372000	-0.061099000
6	-2.311507000	-2.423407000	-0.856249000
6	-0.522084000	-3.224442000	0.193298000
6	-1.526291000	-4.362562000	0.197939000
6	-2.437120000	-3.932106000	-0.994766000
6	-3.299496000	-1.514969000	-1.037060000
6	1.758371000	-2.403917000	0.397430000
6	2.765400000	-0.634329000	-0.517614000
6	3.230461000	-2.607242000	0.740980000
6	3.901781000	-1.604983000	-0.249221000
6	0.779590000	-3.349053000	0.655156000
6	2.922037000	0.640803000	-0.962843000
1	4.115115000	-2.156319000	-1.176031000
6	5.212951000	-0.968615000	0.257794000
1	5.736502000	-1.677039000	0.905970000
1	4.984273000	-0.092332000	0.873139000
6	6.198661000	-0.573455000	-0.864783000
1	6.467459000	-1.461933000	-1.442383000
1	5.772819000	0.172476000	-1.537883000
6	7.432701000	0.019337000	-0.231763000
8	7.571938000	1.192467000	0.088080000
8	8.372935000	-0.901761000	0.025608000
1	3.913887000	0.970240000	-1.234629000
6	3.407718000	-2.208853000	2.225435000

1	2.754208000	-2.824146000	2.852032000
1	4.435334000	-2.362609000	2.562737000
1	3.137838000	-1.158785000	2.377411000
1	-1.031632000	-5.316233000	-0.016387000
6	-2.224986000	-4.506539000	1.571670000
1	-2.962379000	-5.313950000	1.500539000
1	-1.480409000	-4.840385000	2.301847000
6	-2.904825000	-3.242454000	2.113863000
1	-2.172038000	-2.434978000	2.249137000
1	-3.643435000	-2.846241000	1.415148000
6	-3.570006000	-3.485795000	3.460168000
8	-3.253897000	-4.381230000	4.217756000
8	-4.548176000	-2.625870000	3.801633000
6	-3.865761000	-4.480129000	-0.965949000
1	-4.412982000	-4.188338000	-1.866562000
1	-3.846029000	-5.574700000	-0.933414000
1	-4.427223000	-4.122559000	-0.099862000
6	-1.715036000	-4.396958000	-2.311359000
1	-1.864349000	-5.474192000	-2.429817000
1	-0.643977000	-4.195687000	-2.230391000
1	9.109535000	-0.458009000	0.490904000
1	-4.742074000	-1.983100000	3.081282000
1	-1.664131000	3.801903000	0.158605000
6	3.696492000	-4.069089000	0.501658000
1	3.373397000	-4.399206000	-0.489009000
1	3.257536000	-4.727871000	1.255373000
6	5.199073000	-4.201469000	0.617677000
8	5.813955000	-4.332373000	1.656719000
1	1.061409000	-4.283579000	1.124407000
6	3.472595000	3.688870000	-1.304267000
1	3.752801000	3.448584000	-2.334705000
1	3.378282000	4.777884000	-1.237639000
6	0.814762000	5.129381000	-0.259993000
1	-0.241108000	5.413929000	-0.301976000
1	1.316466000	5.730225000	-1.026664000
6	4.611123000	3.279227000	-0.376890000
8	4.593481000	3.403026000	0.832936000
8	5.632446000	2.737908000	-1.061994000
1	6.318908000	2.368007000	-0.447954000
6	1.389867000	5.500764000	1.117861000
1	0.854756000	4.973871000	1.917425000
1	2.435391000	5.176120000	1.201102000
6	1.321267000	6.984030000	1.395826000
8	0.906947000	7.831607000	0.630287000
8	1.790279000	7.279401000	2.629632000
1	1.722278000	8.247621000	2.738207000

6	-4.391315000	3.230323000	0.313730000
1	-3.833623000	3.777978000	1.080998000
1	-5.370167000	3.014455000	0.754841000
6	-5.673171000	0.305342000	-0.322779000
1	-6.343577000	1.157728000	-0.157293000
1	-6.027867000	-0.177403000	-1.241060000
6	-4.587159000	4.141563000	-0.909105000
1	-5.165323000	3.629724000	-1.688457000
1	-3.624919000	4.392547000	-1.371495000
6	-5.298596000	5.430502000	-0.564636000
8	-5.718126000	5.737632000	0.532804000
8	-5.423882000	6.230479000	-1.647921000
1	-5.889223000	7.038337000	-1.356672000
6	-5.901265000	-0.662479000	0.830302000
8	-5.146404000	-0.801025000	1.779432000
8	-7.042353000	-1.352390000	0.694803000
1	-7.145769000	-1.929929000	1.476470000
1	-4.273974000	-1.862398000	-1.360874000
8	5.812874000	-4.105589000	-0.585660000
1	6.775790000	-4.153587000	-0.423853000
6	-2.201158000	-3.661243000	-3.537150000
8	-1.721746000	-2.629719000	-3.962163000
8	-3.271806000	-4.258650000	-4.110630000
1	-3.541002000	-3.700991000	-4.866741000

NiII-SCoM-A
NiII-SCoM-A-opt.gjf.log

Temperature 298.150 Kelvin. Pressure 1.00000 Atm.

Zero-point correction= 0.914356 (Hartree/Particle)
Thermal correction to Energy= 0.982875
Thermal correction to Enthalpy= 0.983819
Thermal correction to Gibbs Free Energy= 0.798745
Sum of electronic and zero-point Energies= -4319.287511
Sum of electronic and thermal Energies= -4319.218992
Sum of electronic and thermal Enthalpies= -4319.218048
Sum of electronic and thermal Free Energies= -4319.403123

6	-5.412655000	-0.841081000	1.162476000
6	0.282614000	-4.389863000	2.536661000
6	6.527326000	3.519989000	-0.312827000
6	-2.308813000	6.125109000	-1.655187000
7	-2.504631000	0.260876000	0.042975000
6	-6.930161000	-1.078288000	1.021935000
6	1.540237000	-4.274158000	3.418229000

7	-0.373383000	-1.673860000	0.772312000
8	6.598156000	4.534416000	-0.979013000
8	-1.630419000	7.013758000	-2.132931000
7	-0.572350000	2.463787000	0.302538000
8	-7.521016000	-0.132555000	3.112487000
8	1.918342000	-5.785940000	5.286362000
8	-8.132268000	-2.273658000	2.767151000
8	0.343181000	-4.187502000	5.465466000
6	-2.969544000	2.639290000	-0.256880000
6	-2.676722000	-2.176348000	0.154076000
6	2.013542000	-1.833400000	1.293134000
6	1.795692000	2.993570000	0.714681000
28	-0.377668000	0.391464000	0.253659000
6	-3.328656000	1.319854000	-0.270672000
6	-1.360892000	-2.528809000	0.444392000
6	2.390601000	-0.464254000	1.232129000
6	0.472719000	3.357395000	0.441551000
6	-4.685304000	0.802959000	-0.777047000
6	-0.801331000	-3.945538000	0.231732000
6	3.743693000	0.033788000	1.377239000
6	-0.010586000	4.701442000	0.271378000
6	-4.686933000	-0.643846000	-0.189693000
6	0.493783000	-3.868699000	1.095974000
6	3.681273000	1.402371000	1.219447000
6	-1.373214000	4.594836000	0.047363000
6	-3.195050000	-0.887154000	0.010899000
6	0.773664000	-2.374120000	1.066916000
6	2.295509000	1.721191000	0.968488000
6	-1.689638000	3.190234000	0.041587000
6	-4.536511000	0.730009000	-2.340255000
6	4.986797000	-0.815267000	1.457377000
6	0.786991000	5.971120000	0.369122000
6	-5.737525000	0.123593000	-3.025224000
6	0.435524000	-5.003407000	-1.865531000
6	5.168855000	-1.441655000	0.075348000
8	-6.776130000	0.704085000	-3.279165000
8	1.071818000	-5.839241000	-1.252998000
8	5.580275000	-0.820315000	-0.891081000
6	-0.467919000	-3.935960000	-1.284198000
6	4.820998000	2.376694000	1.197997000
8	4.741311000	-2.703242000	0.037991000
6	-5.869985000	1.702808000	-0.407307000
6	-1.748516000	-5.105609000	0.548604000
6	5.353187000	2.577367000	-0.244572000
6	-2.335983000	5.732021000	-0.191708000
6	-7.596172000	-1.260708000	2.364480000

6	1.321217000	-4.847865000	4.795551000
7	1.540201000	0.561549000	0.988562000
8	7.551406000	3.110061000	0.479340000
8	-3.101489000	5.326442000	-2.405922000
6	1.764137000	-0.942156000	-2.221320000
8	4.396664000	-2.301349000	-3.979229000
16	0.195921000	0.051003000	-2.187373000
6	1.802812000	-1.870321000	-3.436987000
8	2.789583000	-4.186302000	-4.319986000
16	3.219818000	-3.030552000	-3.449696000
8	3.379725000	-3.495133000	-2.022200000
1	-4.976128000	-1.718460000	1.653729000
1	-5.220604000	0.011532000	1.822293000
1	-0.010897000	-5.444594000	2.489254000
1	-0.540848000	-3.845341000	3.013416000
1	-7.407551000	-0.232330000	0.520358000
1	-7.119761000	-1.977967000	0.430610000
1	2.383031000	-4.804706000	2.966754000
1	1.816883000	-3.219851000	3.526644000
1	2.817846000	-2.540122000	1.451195000
1	-5.096239000	-1.367535000	-0.902024000
1	1.307154000	-4.433916000	0.637063000
1	-3.640275000	0.154422000	-2.588379000
1	-4.415612000	1.748580000	-2.718620000
1	5.859544000	-0.196015000	1.682060000
1	4.904957000	-1.599788000	2.216041000
1	0.310675000	6.747148000	-0.239056000
1	1.794628000	5.831643000	-0.038941000
1	-1.398861000	-3.928055000	-1.862865000
1	0.020381000	-2.987888000	-1.535998000
1	5.641623000	2.014456000	1.825980000
1	4.508995000	3.342567000	1.614092000
1	-5.973109000	1.807014000	0.676395000
1	-6.806733000	1.320410000	-0.819692000
1	-5.731300000	2.704412000	-0.826509000
1	-1.224630000	-6.057901000	0.414953000
1	-2.137810000	-5.058731000	1.569692000
1	-2.604801000	-5.102478000	-0.134817000
1	5.657709000	1.598827000	-0.636543000
1	4.565689000	2.973959000	-0.889724000
1	-2.050771000	6.613805000	0.388265000
1	-3.354015000	5.450075000	0.092780000
1	1.821702000	-1.541890000	-1.312692000
1	2.635114000	-0.278736000	-2.225941000
1	0.913867000	-2.501876000	-3.474020000
1	1.867549000	-1.313795000	-4.376943000

1	-7.946965000	-0.328922000	3.968816000
1	0.255584000	-4.621771000	6.335353000
1	8.263353000	3.770295000	0.378656000
1	4.443845000	-2.963657000	-0.890702000
1	-2.968190000	5.580325000	-3.339482000
1	-6.388243000	-1.508125000	-3.717792000
1	1.386877000	-4.978308000	-3.577720000
1	2.518701000	3.801494000	0.742261000
6	0.922893000	6.518306000	1.819078000
1	1.401834000	7.502346000	1.785820000
1	-0.063506000	6.615762000	2.279912000
6	1.804505000	5.615454000	2.648679000
8	3.012660000	5.531147000	2.525112000
8	1.107160000	4.859239000	3.522366000
1	1.743256000	4.249371000	3.944824000
1	-3.375288000	-2.988796000	-0.019724000
1	-3.727286000	3.353546000	-0.558141000
8	0.466579000	-4.858789000	-3.206708000
8	-5.558801000	-1.188930000	-3.311746000

c) Ni^{I}, Ni^{II} and Ni^{II}-SCoM for **B**.

NiI-B
NiI-B-opt.gjf.log

Temperature 298.150 Kelvin. Pressure 1.00000 Atm.

Zero-point correction= 0.865192 (Hartree/Particle)
Thermal correction to Energy= 0.926020
Thermal correction to Enthalpy= 0.926964
Thermal correction to Gibbs Free Energy= 0.760345
Sum of electronic and zero-point Energies= -3178.778640
Sum of electronic and thermal Energies= -3178.717812
Sum of electronic and thermal Enthalpies= -3178.716868
Sum of electronic and thermal Free Energies= -3178.883487

6	-4.440021000	0.976920000	1.888113000
6	-0.401488000	-4.919696000	1.077413000
6	7.412895000	0.050540000	0.485257000
6	-0.319603000	5.962691000	0.598321000
7	-2.005157000	0.638388000	-0.267022000
6	-5.841557000	1.421734000	2.312055000
6	1.087758000	-5.260769000	1.133823000
7	-0.789416000	-1.999530000	-0.452183000
8	8.228770000	0.140112000	-0.411614000
8	-1.472880000	6.058925000	0.967321000

7	0.546698000	1.874023000	-0.804704000
8	-7.208810000	2.024734000	4.151629000
8	2.807015000	-6.298200000	2.450328000
8	-5.164094000	1.216823000	4.642326000
8	0.748725000	-6.158326000	3.337152000
6	-1.628908000	3.018532000	-0.667048000
6	-3.081246000	-1.503436000	0.205330000
6	1.274445000	-3.136194000	-1.099673000
6	2.952637000	1.424959000	-1.048453000
28	-0.124266000	-0.056295000	-0.657217000
6	-2.441102000	1.938412000	-0.464191000
6	-2.051853000	-2.368094000	-0.146068000
6	2.147671000	-2.020698000	-1.184988000
6	1.863306000	2.289105000	-0.923948000
6	-3.974613000	1.969394000	-0.512856000
6	-2.277801000	-3.864728000	-0.391640000
6	3.587196000	-2.117772000	-1.285198000
6	1.932384000	3.730326000	-0.939507000
6	-4.296177000	0.727357000	0.369416000
6	-0.812944000	-4.387360000	-0.323104000
6	4.073517000	-0.829457000	-1.231077000
6	0.627962000	4.175092000	-0.851237000
6	-3.056486000	-0.114703000	0.104139000
6	-0.035711000	-3.126594000	-0.694452000
6	2.920190000	0.033813000	-1.111437000
6	-0.209576000	3.001981000	-0.761738000
6	-4.352478000	1.639973000	-2.001941000
6	4.370144000	-3.402326000	-1.263140000
6	3.189108000	4.549817000	-0.962113000
7	-5.195633000	-3.815795000	-1.978491000
6	-5.811196000	1.244655000	-2.198528000
6	-3.997225000	-3.243411000	-2.262435000
6	4.302288000	-4.061907000	0.112169000
6	3.791107000	4.691979000	0.445447000
8	-6.744607000	2.034375000	-2.026714000
8	-3.918792000	-2.132466000	-2.797315000
8	4.483064000	-3.480812000	1.167139000
6	-2.760423000	-4.045287000	-1.878648000
6	5.508243000	-0.400985000	-1.139349000
7	-6.013787000	-0.047292000	-2.568863000
8	3.994273000	-5.371564000	0.045449000
6	-4.601768000	3.299351000	-0.090613000
6	-3.256700000	-4.536634000	0.577311000
6	5.954045000	-0.300138000	0.329281000
6	0.138922000	5.599295000	-0.800434000
6	-5.993405000	1.529150000	3.811808000

6	1.634466000	-5.954359000	2.351627000
7	1.758874000	-0.725222000	-1.071866000
8	7.741728000	0.255569000	1.784372000
8	0.728067000	6.145115000	1.440422000
1	-4.207960000	0.043365000	2.412728000
1	-3.691452000	1.704843000	2.222342000
1	-0.994620000	-5.809896000	1.312203000
1	-0.631102000	-4.168688000	1.842055000
1	-6.126596000	2.384159000	1.877763000
1	-6.596610000	0.706141000	1.957787000
1	1.382220000	-5.882924000	0.280718000
1	1.681191000	-4.346869000	1.038366000
1	-2.104072000	3.990228000	-0.745786000
1	-4.041073000	-1.943877000	0.454938000
1	1.724888000	-4.109059000	-1.268410000
1	-5.195669000	0.205440000	0.024029000
1	-0.643343000	-5.192583000	-1.049201000
1	-3.697105000	0.843449000	-2.366847000
1	-4.162047000	2.531795000	-2.608364000
1	4.022708000	-4.117658000	-2.015531000
1	5.428643000	-3.203782000	-1.470970000
1	2.993618000	5.551275000	-1.361984000
1	3.937098000	4.102279000	-1.627065000
1	3.063036000	5.159698000	1.119877000
1	-1.961037000	-3.709551000	-2.544667000
1	-2.928148000	-5.114813000	-2.058550000
1	5.667269000	0.564938000	-1.632806000
1	6.160892000	-1.113391000	-1.656584000
1	-4.304711000	3.581293000	0.924173000
1	-5.690809000	3.250301000	-0.159217000
1	-4.275798000	4.101573000	-0.761889000
1	-3.305302000	-5.616625000	0.391446000
1	-2.961648000	-4.381216000	1.618176000
1	-4.266589000	-4.132989000	0.464678000
1	5.356006000	0.447111000	0.864990000
1	5.766909000	-1.252819000	0.842367000
1	-0.712532000	5.759083000	-1.468918000
1	0.940025000	6.285624000	-1.094086000
1	-7.237599000	2.057628000	5.127095000
1	1.224101000	-6.588030000	4.075331000
1	8.695842000	0.462352000	1.804612000
1	3.820834000	-5.704507000	0.962995000
1	0.355767000	6.315046000	2.326839000
1	-6.969954000	-0.351689000	-2.696897000
1	-5.264380000	-0.731985000	-2.681089000
1	-6.047915000	-3.313604000	-2.186686000

1	3.932832000	1.889459000	-1.120364000
1	4.011710000	3.707482000	0.875695000
6	5.054018000	5.517837000	0.469321000
8	5.542288000	6.108165000	-0.474194000
8	5.610402000	5.543561000	1.705439000
1	6.411428000	6.098577000	1.642915000
1	-5.271898000	-4.730607000	-1.560227000

NiII-B

NiII-B-opt.gjf.log

Temperature 298.150 Kelvin. Pressure 1.00000 Atm.

Zero-point correction= 0.868728 (Hartree/Particle)
Thermal correction to Energy= 0.929024
Thermal correction to Enthalpy= 0.929968
Thermal correction to Gibbs Free Energy= 0.765686
Sum of electronic and zero-point Energies= -3178.683341
Sum of electronic and thermal Energies= -3178.623046
Sum of electronic and thermal Enthalpies= -3178.622102
Sum of electronic and thermal Free Energies= -3178.786383

28	-0.218224000	-0.342829000	-0.411780000
7	0.635928000	1.385790000	-0.561671000
7	1.532740000	-1.208624000	-0.165396000
7	-1.080305000	-2.084288000	-0.319989000
7	-1.956412000	0.511157000	-0.495248000
6	0.063105000	2.636346000	-0.344452000
6	1.046591000	3.664000000	-0.548321000
6	1.935254000	1.613972000	-0.891829000
6	2.203489000	3.025233000	-0.941977000
6	-3.156303000	-0.100651000	-0.679513000
6	-2.242450000	1.829224000	-0.148602000
6	-3.662065000	2.011451000	-0.047177000
6	-4.231003000	0.802965000	-0.399690000
6	-1.281464000	2.828450000	-0.072629000
6	-2.338770000	-2.387221000	-0.833450000
6	-0.564617000	-3.198584000	0.233632000
6	-1.585983000	-4.321050000	0.252121000
6	-2.485687000	-3.895514000	-0.951613000
6	-3.315055000	-1.467489000	-1.021244000
6	1.728340000	-2.408157000	0.426447000
6	2.759161000	-0.659103000	-0.500813000
6	3.197840000	-2.635966000	0.765147000
6	3.881719000	-1.645234000	-0.228878000
6	0.735219000	-3.336015000	0.695976000

6	2.932383000	0.611730000	-0.952346000
1	4.083036000	-2.200176000	-1.157330000
6	5.205173000	-1.029529000	0.272651000
1	5.717273000	-1.751111000	0.914052000
1	4.990791000	-0.149871000	0.888510000
6	6.192890000	-0.645361000	-0.852754000
1	6.456567000	-1.538187000	-1.426305000
1	5.773268000	0.101847000	-1.528672000
6	7.431359000	-0.058857000	-0.222312000
8	7.571475000	1.111410000	0.109181000
8	8.376070000	-0.979352000	0.017495000
1	3.928914000	0.927214000	-1.223571000
6	3.384833000	-2.232237000	2.247304000
1	2.717059000	-2.830195000	2.876205000
1	4.408678000	-2.413834000	2.578558000
1	3.137242000	-1.175660000	2.394481000
1	-1.103767000	-5.284511000	0.053202000
6	-2.289811000	-4.438401000	1.625412000
1	-3.039713000	-5.234945000	1.561118000
1	-1.552914000	-4.776398000	2.361463000
6	-2.951970000	-3.159369000	2.155096000
1	-2.205815000	-2.364748000	2.293102000
1	-3.676872000	-2.753713000	1.447353000
6	-3.632796000	-3.388315000	3.496472000
8	-3.333233000	-4.284540000	4.259998000
8	-4.605817000	-2.518443000	3.826013000
6	-3.925279000	-4.414938000	-0.915306000
1	-4.466160000	-4.121197000	-1.820305000
1	-3.929852000	-5.509478000	-0.865932000
1	-4.484474000	-4.033459000	-0.057853000
6	-1.774908000	-4.401014000	-2.255196000
1	-1.884517000	-5.490682000	-2.303332000
1	-0.706662000	-4.174141000	-2.194251000
6	-2.282213000	-3.725583000	-3.522474000
8	-1.879181000	-2.617257000	-3.869295000
7	-3.230094000	-4.405869000	-4.224537000
1	-3.585709000	-4.001482000	-5.080529000
1	-3.521779000	-5.340465000	-3.980342000
1	9.114071000	-0.537865000	0.482671000
1	-4.785291000	-1.871500000	3.105056000
1	-1.617246000	3.837734000	0.139765000
6	3.638518000	-4.104370000	0.526742000
1	3.239405000	-4.446674000	-0.433983000
1	3.222526000	-4.746343000	1.309186000
6	5.154661000	-4.268712000	0.580555000
8	5.785584000	-4.207274000	1.635033000

7	5.773793000	-4.423653000	-0.622558000
1	6.774578000	-4.572631000	-0.630411000
1	5.263577000	-4.599961000	-1.475466000
1	1.006037000	-4.269109000	1.174716000
6	3.518696000	3.652629000	-1.317716000
1	3.799064000	3.398334000	-2.344763000
1	3.435039000	4.743119000	-1.262677000
6	0.877267000	5.132021000	-0.290696000
1	-0.175301000	5.428313000	-0.334850000
1	1.385342000	5.719904000	-1.063257000
6	4.650065000	3.240807000	-0.382862000
8	4.634093000	3.386459000	0.824623000
8	5.662239000	2.670761000	-1.057732000
1	6.340019000	2.295153000	-0.437147000
6	1.457533000	5.510000000	1.083188000
1	0.916450000	4.997619000	1.888135000
1	2.499033000	5.173213000	1.169124000
6	1.407839000	6.996574000	1.346564000
8	1.007469000	7.842374000	0.571619000
8	1.877023000	7.297843000	2.578985000
1	1.821931000	8.267938000	2.677525000
6	-4.353133000	3.296156000	0.303663000
1	-3.787252000	3.843677000	1.065009000
1	-5.332403000	3.092476000	0.749853000
6	-5.664226000	0.372119000	-0.317634000
1	-6.318835000	1.222285000	-0.089207000
1	-6.038607000	-0.061159000	-1.251517000
6	-4.544987000	4.200424000	-0.924907000
1	-5.130972000	3.688128000	-1.698139000
1	-3.582197000	4.438762000	-1.392768000
6	-5.243254000	5.498373000	-0.587710000
8	-5.660210000	5.815954000	0.507761000
8	-5.360314000	6.293676000	-1.675489000
1	-5.817859000	7.107541000	-1.388685000
6	-5.860790000	-0.645783000	0.796932000
8	-5.204004000	-0.674911000	1.825924000
8	-6.842535000	-1.519598000	0.531403000
1	-6.932689000	-2.120303000	1.297285000
1	-4.292614000	-1.802767000	-1.348387000

NiII-SCoM-B

NiII-SCoM-B-opt.gjf.log

Temperature 298.150 Kelvin. Pressure 1.00000 Atm.

Zero-point correction= 0.940166 (Hartree/Particle)

Thermal correction to Energy= 1.009376
Thermal correction to Enthalpy= 1.010320
Thermal correction to Gibbs Free Energy= 0.824855
Sum of electronic and zero-point Energies= -4279.529468
Sum of electronic and thermal Energies= -4279.460259
Sum of electronic and thermal Enthalpies= -4279.459315
Sum of electronic and thermal Free Energies= -4279.644780

6	-3.916532000	2.557509000	2.255147000
6	-0.830472000	-3.913035000	2.790369000
6	7.514890000	-0.573526000	0.727705000
6	0.843920000	6.388853000	-0.155976000
7	-1.748507000	1.382603000	0.137566000
6	-5.206214000	3.291125000	2.628864000
6	0.584372000	-4.489323000	2.853262000
7	-0.963853000	-1.459455000	0.530161000
8	8.277439000	-0.824604000	-0.185000000
8	-0.250593000	6.754846000	0.222206000
7	0.951929000	2.040249000	-0.750125000
8	-6.339568000	4.472969000	4.342551000
8	2.236337000	-5.446625000	4.312392000
8	-4.332467000	3.603434000	4.880388000
8	0.291556000	-4.790102000	5.220167000
6	-1.017006000	3.524920000	-0.775681000
6	-3.082501000	-0.418912000	1.123113000
6	0.885954000	-3.002250000	0.165338000
6	3.225384000	1.122468000	-0.967272000
28	-0.059067000	0.257348000	-0.453652000
6	-1.979113000	2.665295000	-0.319593000
6	-2.232101000	-1.510977000	0.974719000
6	1.904462000	-2.094386000	-0.240901000
6	2.303960000	2.177129000	-0.995293000
6	-3.491125000	2.925551000	-0.324427000
6	-2.681475000	-2.958915000	1.215314000
6	3.305037000	-2.439030000	-0.358318000
6	2.599334000	3.553557000	-1.303922000
6	-3.931471000	1.968451000	0.825874000
6	-1.298491000	-3.678475000	1.327801000
6	3.980274000	-1.270608000	-0.642030000
6	1.390525000	4.227355000	-1.258838000
6	-2.858889000	0.894207000	0.703637000
6	-0.383111000	-2.698734000	0.595122000
6	2.982361000	-0.226191000	-0.707581000
6	0.380653000	3.258688000	-0.907969000
6	-4.001660000	2.338728000	-1.691457000
6	3.886208000	-3.790287000	-0.046317000

6	3.967443000	4.127050000	-1.529289000
7	-5.764755000	-2.914039000	-0.142742000
6	-5.504299000	2.092572000	-1.721943000
6	-4.513705000	-2.639737000	-0.610324000
6	3.824293000	-4.080896000	1.451144000
6	4.675654000	4.424983000	-0.197483000
8	-6.327378000	3.014656000	-1.716734000
8	-4.309320000	-1.707484000	-1.391793000
8	4.131138000	-3.285815000	2.322369000
6	-3.387685000	-3.509962000	-0.075153000
6	5.466166000	-1.071457000	-0.694828000
7	-5.881501000	0.787844000	-1.740516000
8	3.365829000	-5.316539000	1.721212000
6	-3.895842000	4.393952000	-0.193485000
6	-3.611085000	-3.130122000	2.424646000
6	6.012895000	-0.707401000	0.696049000
6	1.144821000	5.698254000	-1.471805000
6	-5.207614000	3.782661000	4.057487000
6	1.120057000	-4.957459000	4.178940000
7	1.733889000	-0.768540000	-0.451916000
8	7.952064000	-0.133757000	1.933811000
8	1.958576000	6.533482000	0.603925000
6	-0.974957000	-1.931556000	-2.721149000
8	-0.054211000	-4.880232000	-3.370028000
16	-0.789556000	-0.097300000	-2.821016000
6	-1.231847000	-2.601335000	-4.062843000
8	-1.664710000	-4.904246000	-5.283614000
16	-1.372191000	-4.413685000	-3.901664000
8	-2.503132000	-4.649980000	-2.945015000
1	-3.775169000	1.735651000	2.966058000
1	-3.051635000	3.218972000	2.381840000
1	-1.529567000	-4.597735000	3.281141000
1	-0.861209000	-2.969566000	3.347680000
1	-5.401294000	4.151227000	1.981360000
1	-6.075569000	2.630791000	2.506433000
1	0.695575000	-5.339393000	2.169724000
1	1.304883000	-3.744326000	2.504630000
1	-1.338082000	4.524494000	-1.047985000
1	-4.076275000	-0.620590000	1.508338000
1	1.181601000	-4.043512000	0.228406000
1	-4.922568000	1.540873000	0.643042000
1	-1.320624000	-4.650961000	0.821870000
1	-3.453663000	1.418296000	-1.917704000
1	-3.765369000	3.059991000	-2.480629000
1	3.378305000	-4.595137000	-0.586404000
1	4.943118000	-3.824832000	-0.337145000

1	3.909711000	5.053452000	-2.111357000
1	4.586222000	3.440458000	-2.118530000
1	4.076835000	5.125770000	0.397660000
1	-2.670865000	-3.619445000	-0.891716000
1	-3.758886000	-4.515787000	0.157909000
1	5.736536000	-0.285302000	-1.408931000
1	5.967282000	-1.980853000	-1.044303000
1	-3.512764000	4.841464000	0.728982000
1	-4.983338000	4.493817000	-0.231934000
1	-3.491569000	4.972485000	-1.031539000
1	-3.798757000	-4.193097000	2.616497000
1	-3.190675000	-2.685012000	3.330646000
1	-4.579127000	-2.655383000	2.239922000
1	5.571884000	0.231759000	1.051969000
1	5.714061000	-1.470584000	1.426918000
1	0.289119000	5.873458000	-2.130562000
1	2.025769000	6.168870000	-1.920358000
1	-1.809355000	-2.147899000	-2.054885000
1	-0.069252000	-2.350694000	-2.279465000
1	-2.168066000	-2.255187000	-4.513689000
1	-0.417567000	-2.416680000	-4.771473000
1	-6.271067000	4.752945000	5.275412000
1	0.756357000	-5.110692000	6.018013000
1	8.925822000	-0.086353000	1.879476000
1	3.207955000	-5.391006000	2.697285000
1	1.676344000	6.926594000	1.451851000
1	-6.873916000	0.591916000	-1.727832000
1	-5.240477000	-0.003716000	-1.671566000
1	-6.548525000	-2.422809000	-0.552286000
1	4.258326000	1.387756000	-1.177062000
1	4.766078000	3.515019000	0.407983000
6	6.051386000	5.018509000	-0.377874000
8	6.556220000	5.351810000	-1.431856000
8	6.693832000	5.158237000	0.808157000
1	7.565233000	5.552713000	0.611752000
1	-5.960400000	-3.766674000	0.360920000

d) Ni^{I}, Ni^{II} and Ni^{II}-SCoM for **C**.

NiI-C
NiI-C-opt.gjf.log

Temperature 298.150 Kelvin. Pressure 1.00000 Atm.

Zero-point correction= 0.948754 (Hartree/Particle)

Thermal correction to Energy= 1.011288
Thermal correction to Enthalpy= 1.012232
Thermal correction to Gibbs Free Energy= 0.837793
Sum of electronic and zero-point Energies= -3182.767516
Sum of electronic and thermal Energies= -3182.704982
Sum of electronic and thermal Enthalpies= -3182.704038
Sum of electronic and thermal Free Energies= -3182.878477

6	-4.447103000	0.771047000	1.324081000
6	-1.468392000	-4.339402000	1.636295000
6	6.659595000	-2.402424000	1.972885000
6	1.871936000	5.976140000	1.273618000
7	-2.117894000	0.812319000	-0.836244000
6	-5.687274000	1.289828000	2.054200000
6	-0.116402000	-4.773437000	2.232646000
7	-1.150926000	-1.974399000	-0.550586000
8	6.236967000	-1.929278000	3.009064000
8	2.656512000	5.967483000	2.205371000
7	0.687114000	1.608370000	-0.313418000
8	-6.797399000	1.362421000	4.145581000
8	0.013645000	-5.903778000	4.380853000
8	-4.972080000	0.046204000	4.020359000
8	-0.472392000	-3.704071000	4.317703000
6	-1.238857000	3.089125000	-0.827432000
6	-3.519494000	-1.305827000	-0.571033000
6	0.852586000	-3.379310000	-0.569646000
6	3.010789000	0.906376000	-0.501328000
28	-0.234140000	-0.164131000	-0.648079000
6	-2.337950000	2.076906000	-0.723862000
6	-2.540654000	-2.217063000	-0.487806000
6	1.821815000	-2.415403000	-0.769417000
6	1.978861000	1.851284000	-0.255899000
6	-3.813901000	2.470512000	-0.574979000
6	-2.800826000	-3.729145000	-0.493797000
6	3.273358000	-2.754352000	-1.069569000
6	2.322406000	3.262477000	0.209515000
6	-4.418209000	1.084004000	-0.184954000
6	-1.461185000	-4.242079000	0.093026000
6	3.991739000	-1.432726000	-0.713649000
6	0.959036000	3.975889000	0.088503000
6	-3.437200000	0.137840000	-0.928021000
6	-0.518426000	-3.140460000	-0.370933000
6	2.829911000	-0.442571000	-0.672237000
6	-0.054660000	2.812774000	0.099172000
6	-4.299789000	2.927896000	-1.994263000
6	3.414332000	-3.148726000	-2.548676000

6	3.481426000	3.966520000	-0.552759000
7	-3.238282000	-2.484066000	-3.666928000
6	-5.798923000	3.208198000	-2.051485000
6	-3.825095000	-3.401492000	-2.839538000
6	4.801095000	-3.637082000	-2.904898000
6	4.696198000	4.337080000	0.330580000
8	-6.299561000	4.207805000	-1.540054000
8	-5.043553000	-3.564480000	-2.802186000
8	5.654682000	-3.993358000	-2.116780000
6	-2.859382000	-4.212311000	-1.986684000
6	4.713206000	-1.482799000	0.649335000
7	-6.550631000	2.262845000	-2.679390000
8	4.985834000	-3.664668000	-4.242351000
6	-4.024327000	3.606886000	0.438478000
6	-4.069571000	-4.168837000	0.241850000
6	5.996654000	-2.312608000	0.619102000
6	0.680239000	5.053233000	1.159554000
6	-5.745928000	0.817538000	3.490242000
6	-0.173902000	-4.891104000	3.737128000
7	1.619017000	-1.077014000	-0.698644000
8	7.820321000	-3.097173000	1.910409000
8	2.043298000	6.732687000	0.173415000
1	-4.421034000	-0.315983000	1.449561000
1	-3.538097000	1.139104000	1.813136000
1	-2.233636000	-5.063504000	1.937265000
1	-1.756448000	-3.370945000	2.060048000
1	-5.756484000	2.381263000	2.061070000
1	-6.602771000	0.940210000	1.557528000
1	0.191640000	-5.745053000	1.836587000
1	0.655635000	-4.039823000	1.975640000
1	-0.867752000	3.096580000	-1.861574000
1	-1.626190000	4.089871000	-0.618744000
1	-4.539473000	-1.680719000	-0.572924000
1	1.179235000	-4.414033000	-0.535807000
1	3.619104000	-3.590814000	-0.455022000
1	2.593293000	3.184826000	1.273477000
1	-5.429073000	0.964968000	-0.589780000
1	-1.182994000	-5.219074000	-0.320400000
1	4.715479000	-1.140928000	-1.484935000
1	0.917610000	4.457258000	-0.896990000
1	-3.690755000	0.170541000	-2.003504000
1	-0.434979000	2.645053000	1.118423000
1	-4.015975000	2.183857000	-2.746072000
1	-3.789027000	3.861343000	-2.251574000
1	2.715793000	-3.959655000	-2.793811000
1	3.150053000	-2.311163000	-3.204386000

1	3.091119000	4.879182000	-1.007141000
1	3.821577000	3.339636000	-1.383455000
1	4.356682000	4.548115000	1.350723000
1	-1.854356000	-4.173757000	-2.420096000
1	-3.190005000	-5.255802000	-2.001970000
1	4.027296000	-1.886118000	1.404711000
1	4.953469000	-0.463608000	0.969749000
1	-3.584699000	3.365251000	1.410408000
1	-5.087754000	3.814224000	0.565015000
1	-3.567362000	4.535778000	0.083919000
1	-4.087003000	-5.259750000	0.348386000
1	-4.135315000	-3.719814000	1.236893000
1	-4.957714000	-3.884107000	-0.328166000
1	5.817179000	-3.330114000	0.255931000
1	6.721937000	-1.887616000	-0.086675000
1	0.525551000	4.590549000	2.137818000
1	-0.217584000	5.620807000	0.890417000
1	-6.781354000	1.009643000	5.056105000
1	-0.503811000	-3.855476000	5.281833000
1	8.187799000	-3.119685000	2.814905000
1	5.880572000	-4.022442000	-4.403575000
1	2.991427000	7.013402000	0.147333000
1	-7.548736000	2.408272000	-2.754288000
1	-6.148599000	1.469928000	-3.156557000
1	-3.850412000	-1.809771000	-4.109215000
1	4.033193000	1.266223000	-0.446626000
1	5.426769000	3.526326000	0.381177000
6	5.366780000	5.612096000	-0.125071000
8	4.796996000	6.681159000	-0.300326000
8	6.689922000	5.480756000	-0.306177000
1	7.041230000	6.355838000	-0.564974000
1	-2.289714000	-2.179648000	-3.489769000

NiII-C
NiII-C-opt.gjf.log

Temperature 298.150 Kelvin. Pressure 1.00000 Atm.

Zero-point correction= 0.951884 (Hartree/Particle)
Thermal correction to Energy= 1.013469
Thermal correction to Enthalpy= 1.014413
Thermal correction to Gibbs Free Energy= 0.846797
Sum of electronic and zero-point Energies= -3182.648424
Sum of electronic and thermal Energies= -3182.586838
Sum of electronic and thermal Enthalpies= -3182.585894
Sum of electronic and thermal Free Energies= -3182.753510

28	0.156874000	-0.126407000	0.364126000
7	-0.967190000	1.446804000	0.336753000
7	-1.367636000	-1.312524000	0.535851000
7	1.406944000	-1.593946000	0.903308000
7	1.616453000	0.873048000	-0.293071000
6	-0.548592000	2.735723000	0.093035000
6	-1.707556000	3.712288000	0.177820000
6	-2.298351000	1.454361000	0.649613000
6	-2.795885000	2.866678000	0.873705000
6	2.816978000	0.137969000	-0.717020000
6	1.736000000	2.156269000	-0.529243000
6	3.086268000	2.522432000	-1.120316000
6	3.663283000	1.140979000	-1.532567000
6	0.715181000	3.102109000	-0.281545000
6	2.675829000	-1.499725000	1.129610000
6	0.900463000	-2.892669000	1.446588000
6	2.181233000	-3.755640000	1.567928000
6	3.228505000	-2.662307000	1.955013000
6	3.523613000	-0.444161000	0.499888000
6	-1.276805000	-2.708713000	0.278749000
6	-2.651467000	-0.965699000	0.556735000
6	-2.581039000	-3.167712000	-0.356652000
6	-3.562064000	-2.164503000	0.340084000
6	-0.229858000	-3.454775000	0.642155000
6	-3.109213000	0.350256000	0.737650000
1	-3.786037000	-2.578543000	1.335680000
6	-4.889677000	-1.862248000	-0.382196000
1	-5.160508000	-2.701574000	-1.025620000
1	-4.772497000	-1.000761000	-1.047245000
6	-6.048043000	-1.621962000	0.588632000
1	-6.232264000	-2.523053000	1.187540000
1	-5.837123000	-0.824238000	1.311195000
6	-7.333314000	-1.270543000	-0.126941000
8	-7.472455000	-1.184707000	-1.329652000
8	-8.341701000	-1.057845000	0.748062000
1	-4.167120000	0.516650000	0.892565000
6	-2.499729000	-2.934809000	-1.881993000
1	-1.677774000	-3.527670000	-2.296509000
1	-3.421615000	-3.244070000	-2.376736000
1	-2.307730000	-1.879638000	-2.105714000
1	0.510967000	-2.671355000	2.450575000
1	2.069979000	-4.475857000	2.385735000
6	2.539018000	-4.552268000	0.295271000
1	3.469270000	-5.098894000	0.483888000
1	1.782442000	-5.324407000	0.130549000

6	2.687668000	-3.747279000	-1.005812000
1	1.747851000	-3.234076000	-1.250398000
1	3.445187000	-2.964402000	-0.922515000
6	3.028067000	-4.643303000	-2.191050000
8	2.707560000	-5.812639000	-2.253025000
8	3.687962000	-4.066605000	-3.211042000
6	4.696154000	-3.019288000	1.679860000
1	5.363501000	-2.227772000	2.035254000
1	4.955170000	-3.937227000	2.217305000
1	4.907318000	-3.184063000	0.621457000
6	3.081866000	-2.343147000	3.483047000
1	3.487159000	-3.195307000	4.037943000
1	2.027693000	-2.247046000	3.754984000
6	3.734539000	-1.027176000	3.888466000
8	3.271122000	0.050081000	3.506658000
7	4.842397000	-1.112600000	4.661538000
1	5.283392000	-0.259142000	4.979487000
1	5.194591000	-1.990533000	5.013939000
1	3.763031000	0.326381000	1.239537000
1	4.468607000	-0.896299000	0.184029000
1	2.485124000	-0.693455000	-1.339670000
1	4.718121000	1.053022000	-1.259265000
6	3.531616000	0.888848000	-3.040565000
1	4.121670000	1.621829000	-3.605661000
1	2.487632000	1.023455000	-3.359336000
6	3.955684000	-0.507335000	-3.467055000
8	4.336493000	-1.374798000	-2.694679000
8	3.870553000	-0.795811000	-4.771114000
1	2.936115000	3.171052000	-1.992816000
1	-3.780290000	3.006137000	0.419573000
1	-1.424662000	4.586096000	0.773854000
6	-2.109804000	4.180450000	-1.238195000
1	-2.480565000	3.321278000	-1.811138000
1	-1.218605000	4.537040000	-1.764458000
6	-3.160600000	5.290462000	-1.229914000
1	-4.069844000	4.995849000	-0.696572000
1	-2.794607000	6.173955000	-0.692488000
6	-3.542965000	5.715168000	-2.629140000
8	-3.081430000	5.264737000	-3.658848000
8	-4.485103000	6.683968000	-2.610120000
6	-2.910304000	3.135590000	2.384118000
1	-3.545696000	2.381627000	2.864053000
1	-1.930439000	3.062263000	2.871598000
6	-3.500898000	4.496815000	2.685396000
8	-3.928733000	5.278937000	1.860404000
8	-3.507672000	4.741831000	4.010010000

1	-9.137725000	-0.842121000	0.224669000
1	-3.910072000	5.622035000	4.144706000
1	-4.683922000	6.912159000	-3.538770000
1	3.549659000	-0.035083000	-5.287815000
1	3.935683000	-3.130888000	-3.024705000
1	0.917471000	4.147664000	-0.482107000
6	3.945313000	3.286004000	-0.090903000
1	3.368360000	4.123821000	0.315008000
1	4.175181000	2.633369000	0.758664000
6	5.242807000	3.821743000	-0.696234000
1	5.854032000	3.019772000	-1.129098000
1	5.037559000	4.517717000	-1.519432000
6	6.095425000	4.541397000	0.326635000
8	5.831757000	4.662607000	1.505270000
8	7.215945000	5.043835000	-0.235270000
1	7.720921000	5.491057000	0.471287000
6	-2.896781000	-4.645437000	-0.025059000
1	-2.700167000	-4.822308000	1.037531000
1	-2.226729000	-5.293558000	-0.599657000
6	-4.323652000	-5.046352000	-0.388814000
8	-4.699652000	-5.157568000	-1.553791000
7	-5.173036000	-5.208972000	0.665011000
1	-6.108533000	-5.543414000	0.471136000
1	-4.841248000	-5.280646000	1.615932000
1	-0.236574000	-4.519177000	0.441192000

NiII-SCoM-C

NiII-SCoM-C-opt.gjf.log

Temperature 298.150 Kelvin. Pressure 1.00000 Atm.

Zero-point correction= 1.023474 (Hartree/Particle)
Thermal correction to Energy= 1.094049
Thermal correction to Enthalpy= 1.094993
Thermal correction to Gibbs Free Energy= 0.906095
Sum of electronic and zero-point Energies= -4283.538336
Sum of electronic and thermal Energies= -4283.467762
Sum of electronic and thermal Enthalpies= -4283.466818
Sum of electronic and thermal Free Energies= -4283.655716

6	-4.622631000	2.147588000	1.637140000
6	-1.123985000	-3.926553000	2.880299000
6	6.729429000	-2.227057000	2.464836000
6	2.380218000	6.573653000	0.802545000
7	-1.959114000	1.414912000	0.183554000
6	-6.008577000	2.772689000	1.809160000

6	0.245277000	-4.590135000	3.124492000
7	-1.142215000	-1.495124000	0.589036000
8	6.364001000	-1.741908000	3.517355000
8	3.233579000	6.680124000	1.665638000
7	0.894509000	2.094433000	0.065820000
8	-7.533563000	3.702752000	3.366373000
8	0.609321000	-5.961818000	5.099640000
8	-5.559038000	2.994564000	4.189992000
8	0.511917000	-3.721294000	5.314105000
6	-1.048138000	3.575277000	-0.511286000
6	-3.379114000	-0.574924000	0.928473000
6	0.794457000	-2.971757000	0.421097000
6	3.151126000	1.205262000	-0.137567000
28	-0.135787000	0.294942000	-0.063036000
6	-2.161584000	2.603278000	-0.260603000
6	-2.494842000	-1.586909000	0.959478000
6	1.783342000	-2.078317000	0.083737000
6	2.193048000	2.250366000	0.005663000
6	-3.620914000	2.847510000	-0.679996000
6	-2.872587000	-3.053834000	1.164708000
6	3.174993000	-2.501904000	-0.351211000
6	2.626430000	3.701176000	0.189718000
6	-4.311984000	1.741680000	0.182841000
6	-1.471092000	-3.705440000	1.388867000
6	3.990248000	-1.205886000	-0.118492000
6	1.283446000	4.458715000	0.046629000
6	-3.223606000	0.657393000	0.095862000
6	-0.550400000	-2.669651000	0.753352000
6	2.892277000	-0.146325000	-0.090267000
6	0.219311000	3.372941000	0.328318000
6	-3.679783000	2.469103000	-2.211365000
6	3.154909000	-2.929304000	-1.829560000
6	3.744422000	4.196644000	-0.771299000
7	-5.754728000	-2.901368000	-0.502082000
6	-5.094688000	2.133579000	-2.669321000
6	-4.463082000	-2.717513000	-0.890086000
6	4.455212000	-3.563510000	-2.258042000
6	5.042951000	4.649030000	-0.060887000
8	-5.941885000	3.010171000	-2.854361000
8	-4.165953000	-1.861408000	-1.730664000
8	5.349215000	-3.935083000	-1.519223000
6	-3.418833000	-3.595964000	-0.211799000
6	4.782696000	-1.216327000	1.204419000
7	-5.366727000	0.812448000	-2.852017000
8	4.520929000	-3.702673000	-3.598930000
6	-4.125145000	4.279992000	-0.484938000

6	-3.904840000	-3.284470000	2.273415000
6	6.018855000	-2.114035000	1.138013000
6	1.143014000	5.714992000	0.934010000
6	-6.303173000	3.150249000	3.242825000
6	0.475456000	-4.867181000	4.590374000
7	1.667039000	-0.719844000	0.066238000
8	7.862910000	-2.961389000	2.359126000
8	2.498861000	7.120491000	-0.421414000
6	-0.034770000	-1.569792000	-2.816095000
8	0.716781000	-4.336808000	-3.736901000
16	-0.534299000	0.183432000	-2.501975000
6	-1.138708000	-2.458910000	-3.377858000
8	-1.689325000	-4.954901000	-4.067179000
16	-0.689383000	-4.223217000	-3.234151000
8	-0.796386000	-4.543130000	-1.770168000
1	-4.573118000	1.257801000	2.273909000
1	-3.856260000	2.826588000	2.026493000
1	-1.896606000	-4.566703000	3.319847000
1	-1.159351000	-2.969828000	3.413813000
1	-6.142539000	3.669894000	1.195814000
1	-6.791862000	2.076655000	1.480795000
1	0.318540000	-5.541261000	2.590341000
1	1.047105000	-3.933987000	2.771156000
1	-0.788434000	3.503413000	-1.578404000
1	-1.412235000	4.596137000	-0.350103000
1	-4.404906000	-0.804486000	1.201686000
1	1.050834000	-4.023301000	0.387825000
1	3.540561000	-3.338248000	0.250361000
1	2.981311000	3.791806000	1.227328000
1	-5.232498000	1.384016000	-0.287633000
1	-1.383678000	-4.657527000	0.854669000
1	4.683952000	-1.008472000	-0.944393000
1	1.184202000	4.768636000	-1.001167000
1	-3.211676000	0.260417000	-0.930095000
1	-0.070988000	3.387574000	1.390380000
1	-2.994078000	1.645619000	-2.435390000
1	-3.346120000	3.334364000	-2.791695000
1	2.347657000	-3.641311000	-2.048832000
1	2.950239000	-2.071076000	-2.478934000
1	3.353562000	5.035282000	-1.350607000
1	3.987926000	3.416434000	-1.499503000
1	4.798122000	5.057473000	0.925659000
1	-2.584746000	-3.699539000	-0.909916000
1	-3.821378000	-4.602134000	-0.038090000
1	4.125469000	-1.547478000	2.017854000
1	5.090085000	-0.194594000	1.453493000

1	-4.059660000	4.602135000	0.558779000
1	-5.161816000	4.354698000	-0.823683000
1	-3.538471000	4.980901000	-1.088445000
1	-4.087116000	-4.355671000	2.417751000
1	-3.583034000	-2.855107000	3.226171000
1	-4.860147000	-2.820532000	2.010775000
1	5.768226000	-3.124007000	0.794387000
1	6.736865000	-1.737551000	0.398178000
1	1.046332000	5.433573000	1.985996000
1	0.253614000	6.281045000	0.635639000
1	0.288086000	-1.989182000	-1.866525000
1	0.824625000	-1.591481000	-3.490953000
1	-2.077885000	-2.330477000	-2.833051000
1	-1.321357000	-2.260958000	-4.439193000
1	-7.654998000	3.918922000	4.310894000
1	0.651514000	-3.977170000	6.245974000
1	8.259641000	-3.000258000	3.250491000
1	5.363606000	-4.153226000	-3.800155000
1	3.453899000	7.336691000	-0.563483000
1	-6.322973000	0.568912000	-3.076239000
1	-4.759298000	0.050931000	-2.560665000
1	-6.479371000	-2.393649000	-0.992744000
1	4.191987000	1.501500000	-0.218887000
1	5.739283000	3.819300000	0.080640000
6	5.724851000	5.781079000	-0.792474000
8	5.195941000	6.845378000	-1.086266000
8	7.008909000	5.527695000	-1.088698000
1	7.370521000	6.318920000	-1.535105000
1	-6.032250000	-3.695547000	0.055823000

e) Ni^{I}, Ni^{II} and Ni^{II}-SCoM for **D**.

NiI-D-opt
NiI-D-opt.gjf.log

Temperature 298.150 Kelvin. Pressure 1.00000 Atm.

Zero-point correction= 0.951577 (Hartree/Particle)
Thermal correction to Energy= 1.012685
Thermal correction to Enthalpy= 1.013629
Thermal correction to Gibbs Free Energy= 0.844487
Sum of electronic and zero-point Energies= -3182.786635
Sum of electronic and thermal Energies= -3182.725527
Sum of electronic and thermal Enthalpies= -3182.724582
Sum of electronic and thermal Free Energies= -3182.893724

6	-3.807211000	0.836761000	1.804904000
6	-2.262194000	-4.502674000	0.508994000
6	5.719552000	-2.719952000	3.112165000
6	2.025963000	6.094356000	0.523281000
7	-2.230100000	0.903120000	-1.023870000
6	-4.677355000	1.480062000	2.888943000
6	-1.137472000	-5.133782000	1.350216000
7	-1.206578000	-1.877712000	-1.169131000
8	5.084928000	-2.216184000	4.017547000
8	2.702602000	6.229304000	1.527102000
7	0.680325000	1.577229000	-0.463327000
8	-5.089970000	1.537831000	5.220472000
8	-1.605879000	-6.609644000	3.224404000
8	-3.818261000	-0.163290000	4.467217000
8	-2.045181000	-4.407227000	3.415256000
6	-1.188698000	3.080930000	-1.162490000
6	-3.455977000	-1.203316000	-0.427909000
6	0.616160000	-3.440527000	-0.790962000
6	2.962014000	0.727087000	-0.248952000
28	-0.251092000	-0.157970000	-0.880036000
6	-2.314133000	2.164513000	-0.798975000
6	-2.643227000	-1.932649000	-1.520572000
6	1.651520000	-2.540474000	-0.678214000
6	1.976867000	1.746102000	-0.272729000
6	-3.617792000	2.620064000	-0.135042000
6	-2.952151000	-3.464145000	-1.766979000
6	3.114139000	-2.962134000	-0.629979000
6	2.371580000	3.192065000	0.021936000
6	-4.174884000	1.235108000	0.364260000
6	-1.843109000	-4.158371000	-0.939767000
6	3.809210000	-1.673440000	-0.132929000
6	1.085292000	3.950707000	-0.368156000
6	-3.544567000	0.293479000	-0.696135000
6	-0.747619000	-3.099481000	-0.970189000
6	2.729056000	-0.622240000	-0.367021000
6	0.003205000	2.868490000	-0.223211000
6	-4.516461000	3.211197000	-1.275026000
6	3.574033000	-3.399016000	-2.029953000
6	3.654330000	3.692718000	-0.702348000
7	-2.847039000	-1.342542000	-2.841360000
6	-5.940919000	3.515120000	-0.817827000
6	-2.736996000	-2.225655000	-3.880061000
6	4.973100000	-3.973720000	-2.045318000
6	4.785719000	4.159110000	0.243904000
8	-6.199843000	4.412700000	-0.018545000

8	-2.675807000	-1.924889000	-5.065288000
8	5.595332000	-4.359831000	-1.075321000
6	-2.682969000	-3.624176000	-3.287178000
6	4.194735000	-1.723134000	1.360291000
7	-6.905338000	2.701310000	-1.328832000
8	5.464294000	-4.043672000	-3.301345000
6	-3.367234000	3.689424000	0.940668000
6	-4.389059000	-3.892157000	-1.459040000
6	5.399579000	-2.621399000	1.639761000
6	0.788741000	5.229888000	0.445110000
6	-4.459251000	0.844197000	4.245145000
6	-1.606109000	-5.496265000	2.739568000
7	1.516797000	-1.187715000	-0.621097000
8	6.830328000	-3.463853000	3.328772000
8	2.366495000	6.614588000	-0.670321000
1	-3.936232000	-0.243513000	1.909686000
1	-2.745193000	1.026372000	2.003055000
1	-3.102109000	-5.205476000	0.467852000
1	-2.622141000	-3.603472000	1.018840000
1	-4.517382000	2.555073000	2.995243000
1	-5.742684000	1.363094000	2.645240000
1	-0.758909000	-6.045334000	0.879694000
1	-0.304670000	-4.428359000	1.445494000
1	-0.861705000	2.871576000	-2.187060000
1	-1.509751000	4.125512000	-1.116037000
1	-2.966438000	-1.409780000	0.525860000
1	-4.478473000	-1.587252000	-0.373816000
1	0.862010000	-4.497320000	-0.764499000
1	3.259135000	-3.800392000	0.058138000
1	2.518295000	3.273631000	1.109948000
1	-5.266273000	1.205975000	0.273055000
1	-1.512799000	-5.085629000	-1.424269000
1	4.706033000	-1.441116000	-0.720712000
1	1.159549000	4.222387000	-1.429346000
1	-4.148125000	0.390259000	-1.609393000
1	-0.365749000	2.862248000	0.815021000
1	-4.523517000	2.533728000	-2.135341000
1	-4.075622000	4.155492000	-1.611521000
1	2.904541000	-4.173807000	-2.425871000
1	3.522149000	-2.563756000	-2.737855000
1	3.379896000	4.523907000	-1.354610000
1	4.042120000	2.909640000	-1.361560000
1	4.351281000	4.580692000	1.156980000
1	-1.679383000	-4.016979000	-3.489918000
1	-3.395596000	-4.287157000	-3.786269000
1	3.331443000	-2.066890000	1.943436000

1	4.414313000	-0.708377000	1.709365000
1	-2.689896000	3.327045000	1.719103000
1	-4.306929000	4.006218000	1.393810000
1	-2.915210000	4.581757000	0.495633000
1	-4.520589000	-4.954786000	-1.692330000
1	-4.666610000	-3.744205000	-0.412760000
1	-5.093431000	-3.324202000	-2.077907000
1	5.250930000	-3.635038000	1.252288000
1	6.293477000	-2.252088000	1.120971000
1	0.500538000	4.974926000	1.468420000
1	-0.030800000	5.785724000	-0.023783000
1	-4.924847000	1.067130000	6.060159000
1	-2.334796000	-4.711811000	4.296610000
1	6.971129000	-3.490502000	4.294756000
1	6.348309000	-4.455523000	-3.243568000
1	3.329982000	6.836698000	-0.635905000
1	-7.870242000	2.888120000	-1.089321000
1	-6.719842000	2.024374000	-2.054068000
1	-2.563777000	-0.381356000	-2.995637000
1	3.982060000	1.035266000	-0.041727000
1	5.440157000	3.333448000	0.533109000
6	5.601069000	5.282071000	-0.353542000
8	5.140946000	6.330573000	-0.786422000
8	6.920884000	5.039449000	-0.352178000
1	7.366667000	5.825879000	-0.725008000

NiII-D

NiII-D-opt.gjf.log

Temperature 298.150 Kelvin. Pressure 1.00000 Atm.

Zero-point correction= 0.954530 (Hartree/Particle)
Thermal correction to Energy= 1.014856
Thermal correction to Enthalpy= 1.015800
Thermal correction to Gibbs Free Energy= 0.851141
Sum of electronic and zero-point Energies= -3182.657729
Sum of electronic and thermal Energies= -3182.597403
Sum of electronic and thermal Enthalpies= -3182.596458
Sum of electronic and thermal Free Energies= -3182.761118

28	-0.039333000	-0.248308000	-0.282631000
7	1.328327000	1.136072000	-0.373248000
7	1.313972000	-1.684098000	-0.346114000
7	-1.571640000	-1.501340000	-0.637856000
7	-1.305393000	1.074132000	0.217337000
6	1.109739000	2.496544000	-0.397733000

6	2.410765000	3.276699000	-0.474270000
6	2.674568000	0.913245000	-0.449054000
6	3.432673000	2.173939000	-0.804898000
6	-2.616217000	0.648506000	0.733057000
6	-1.236468000	2.383586000	0.181115000
6	-2.492886000	3.066401000	0.690958000
6	-3.247816000	1.900799000	1.376582000
6	-0.097735000	3.108365000	-0.220571000
6	-2.811734000	-1.204528000	-0.867903000
6	-1.373238000	-2.949428000	-0.915210000
6	-2.796273000	-3.547738000	-0.859365000
6	-3.612693000	-2.365166000	-1.464955000
6	-3.433381000	0.068204000	-0.411126000
6	1.068925000	-3.144345000	-0.448187000
6	2.620031000	-1.517578000	-0.182584000
6	2.244668000	-3.834738000	0.312419000
6	3.383134000	-2.819343000	-0.003718000
6	-0.327838000	-3.535952000	0.009716000
6	3.295949000	-0.288612000	-0.263764000
1	-0.401348000	-4.627937000	-0.011301000
1	-0.490933000	-3.206740000	1.038212000
1	3.782197000	-3.066921000	-0.998127000
6	4.569431000	-2.799844000	0.980925000
1	4.660676000	-3.788481000	1.439923000
1	4.378678000	-2.102321000	1.803263000
6	5.920086000	-2.489905000	0.327605000
1	6.126633000	-3.200722000	-0.483906000
1	5.961726000	-1.498738000	-0.135140000
6	7.062314000	-2.584403000	1.316771000
8	6.971834000	-2.965497000	2.465705000
8	8.228401000	-2.193443000	0.758393000
1	4.376329000	-0.294354000	-0.253458000
6	1.978414000	-4.011392000	1.807001000
1	1.121663000	-4.669947000	1.976630000
1	2.837426000	-4.466522000	2.306318000
1	1.775442000	-3.049482000	2.291470000
6	2.455793000	-5.145000000	-0.461702000
7	1.319185000	-3.629271000	-1.813631000
6	2.045929000	-4.788925000	-1.892204000
8	2.306561000	-5.398346000	-2.917740000
1	-1.009742000	-3.044247000	-1.942132000
1	-2.848119000	-4.419795000	-1.520752000
6	-3.257231000	-4.016710000	0.536512000
1	-4.252567000	-4.462290000	0.431752000
1	-2.608636000	-4.836166000	0.860000000
6	-3.309719000	-2.978236000	1.668015000

1	-2.330752000	-2.513274000	1.836173000
1	-3.989832000	-2.152774000	1.443936000
6	-3.726310000	-3.613174000	2.992506000
8	-3.614721000	-4.799359000	3.225306000
8	-4.205897000	-2.779893000	3.930554000
6	-5.116512000	-2.369900000	-1.155442000
1	-5.624400000	-1.540716000	-1.658172000
1	-5.557582000	-3.301007000	-1.525590000
1	-5.339122000	-2.301522000	-0.089402000
6	-3.445531000	-2.373038000	-3.023778000
1	-4.022205000	-3.216827000	-3.415476000
1	-2.401068000	-2.535621000	-3.301632000
6	-3.841939000	-1.050695000	-3.669416000
8	-3.200697000	-0.022226000	-3.440737000
7	-4.924580000	-1.072091000	-4.480411000
1	-5.194088000	-0.222026000	-4.958672000
1	-5.421940000	-1.921948000	-4.702541000
1	-3.510801000	0.765281000	-1.250633000
1	-4.451030000	-0.138728000	-0.068030000
1	-2.448440000	-0.136937000	1.469196000
1	3.483902000	-5.515835000	-0.432990000
1	1.804969000	-5.946900000	-0.091288000
1	0.939103000	-3.193412000	-2.643192000
1	-4.319448000	1.933799000	1.164405000
6	-3.056579000	1.908892000	2.898306000
1	-3.462378000	2.832002000	3.331718000
1	-1.986273000	1.894647000	3.150167000
6	-3.707424000	0.723636000	3.589664000
8	-4.316891000	-0.156362000	2.999324000
8	-3.575841000	0.640387000	4.918327000
1	-2.207199000	3.838688000	1.416590000
1	4.343646000	2.260539000	-0.206951000
1	2.352104000	4.034219000	-1.263271000
6	2.683768000	3.978426000	0.875116000
1	2.823417000	3.218265000	1.654071000
1	1.800437000	4.554706000	1.166806000
6	3.893725000	4.911411000	0.839659000
1	4.810430000	4.391362000	0.545093000
1	3.762899000	5.696564000	0.084881000
6	4.129626000	5.577313000	2.175809000
8	3.456324000	5.422389000	3.175439000
8	5.206933000	6.392144000	2.136712000
6	3.824967000	2.120217000	-2.292125000
1	4.390164000	1.205747000	-2.508898000
1	2.934506000	2.085964000	-2.931583000
6	4.678907000	3.297938000	-2.711282000

8	5.127184000	4.145958000	-1.966159000
8	4.902052000	3.288023000	-4.039611000
1	8.923560000	-2.292139000	1.437560000
1	5.470495000	4.054781000	-4.248828000
1	5.301732000	6.789421000	3.023933000
1	-3.070778000	1.391341000	5.278687000
1	-4.282688000	-1.850141000	3.609116000
1	-0.145038000	4.190671000	-0.224684000
6	-3.264687000	3.751815000	-0.455462000
1	-2.581223000	4.397726000	-1.016999000
1	-3.629183000	3.003139000	-1.166591000
6	-4.437019000	4.590606000	0.053814000
1	-5.150545000	3.988196000	0.630074000
1	-4.093278000	5.378991000	0.735334000
6	-5.202739000	5.246014000	-1.075447000
8	-4.968238000	5.110493000	-2.258682000
8	-6.206591000	6.016576000	-0.604367000
1	-6.661238000	6.407027000	-1.375764000

NiII-SCoM-D
NiII-SCoM-D-opt.gjf.log

Temperature 298.150 Kelvin. Pressure 1.00000 Atm.

Zero-point correction= 1.025758 (Hartree/Particle)
Thermal correction to Energy= 1.095258
Thermal correction to Enthalpy= 1.096202
Thermal correction to Gibbs Free Energy= 0.908316
Sum of electronic and zero-point Energies= -4283.556785
Sum of electronic and thermal Energies= -4283.487286
Sum of electronic and thermal Enthalpies= -4283.486342
Sum of electronic and thermal Free Energies= -4283.674227

6	-4.856495000	0.396487000	1.646060000
6	-1.163273000	-4.060641000	2.482774000
6	6.401376000	0.534809000	3.671180000
6	0.083151000	6.836769000	0.129379000
7	-2.457975000	0.477672000	-0.509888000
6	-6.222662000	0.829380000	2.185950000
6	0.018618000	-4.049098000	3.471224000
7	-0.748568000	-1.975468000	-0.000511000
8	5.739476000	1.165351000	4.472029000
8	0.726748000	7.289553000	1.059440000
7	0.083320000	2.057061000	-0.138591000
8	-7.476795000	1.184342000	4.164202000
8	-0.261793000	-4.864616000	5.745577000

8	-5.489649000	0.149148000	4.405291000
8	-1.147238000	-2.874710000	5.167454000
6	-2.095348000	2.813061000	-1.088458000
6	-3.189214000	-1.782001000	0.344150000
6	1.402930000	-2.712052000	0.852437000
6	2.511125000	1.906160000	0.048032000
28	-0.302746000	0.018625000	-0.413183000
6	-2.954578000	1.644367000	-0.703324000
6	-2.053479000	-2.563615000	-0.355471000
6	2.150319000	-1.585605000	0.610737000
6	1.275245000	2.599568000	-0.069009000
6	-4.485209000	1.695881000	-0.620814000
6	-1.921079000	-4.106362000	-0.005480000
6	3.667498000	-1.581959000	0.685608000
6	1.239709000	4.125134000	-0.027287000
6	-4.770548000	0.332986000	0.109750000
6	-0.737883000	-4.121165000	0.996560000
6	4.003230000	-0.070185000	0.650030000
6	-0.224138000	4.419333000	-0.427678000
6	-3.562690000	-0.500552000	-0.389875000
6	0.014242000	-2.859312000	0.598476000
6	2.663987000	0.556529000	0.268008000
6	-0.953495000	3.098472000	-0.104965000
6	-4.999450000	1.660123000	-2.101496000
6	4.261568000	-2.383986000	-0.487125000
6	2.298452000	4.841258000	-0.913872000
7	-2.219621000	-2.547179000	-1.806419000
6	-6.500831000	1.404781000	-2.202255000
6	-1.897490000	-3.731491000	-2.423197000
6	5.716372000	-2.722880000	-0.270385000
6	3.285804000	5.740437000	-0.131960000
8	-7.337331000	2.198635000	-1.774330000
8	-1.893673000	-3.908867000	-3.635437000
8	6.266852000	-2.832421000	0.812293000
6	-1.499776000	-4.731131000	-1.358485000
6	4.508951000	0.484259000	1.997311000
7	-6.851156000	0.219260000	-2.769889000
8	6.369224000	-2.935919000	-1.429821000
6	-4.991893000	2.965662000	0.079845000
6	-3.211055000	-4.770517000	0.483514000
6	5.932018000	0.033873000	2.327185000
6	-0.856550000	5.658123000	0.243282000
6	-6.318810000	0.670940000	3.687383000
6	-0.452953000	-4.009275000	4.904931000
7	1.659225000	-0.361077000	0.270108000
8	7.690001000	0.189959000	3.906824000

8	0.221977000	7.262636000	-1.139395000
6	1.201007000	-1.484290000	-2.753763000
8	3.648052000	-3.309735000	-3.471143000
16	-0.064393000	-0.134169000	-2.832206000
6	1.173718000	-2.466340000	-3.915034000
8	2.083679000	-4.824338000	-4.702997000
16	2.269009000	-3.882601000	-3.560353000
8	1.787313000	-4.438183000	-2.249295000
1	-4.665716000	-0.601668000	2.048896000
1	-4.065843000	1.035455000	2.056847000
1	-1.787123000	-4.934640000	2.699828000
1	-1.781535000	-3.173782000	2.657489000
1	-6.476096000	1.864083000	1.943465000
1	-7.021654000	0.219281000	1.742278000
1	0.639065000	-4.940756000	3.348223000
1	0.645527000	-3.168729000	3.292962000
1	-1.652706000	2.598440000	-2.069866000
1	-2.705920000	3.713727000	-1.191447000
1	-2.881192000	-1.580001000	1.373946000
1	-4.093771000	-2.396624000	0.378477000
1	1.933051000	-3.601180000	1.175162000
1	4.001409000	-2.046778000	1.618635000
1	1.392813000	4.417957000	1.022218000
1	-5.696289000	-0.117823000	-0.264016000
1	-0.115098000	-5.011840000	0.853948000
1	4.747365000	0.155002000	-0.124841000
1	-0.252199000	4.564938000	-1.515021000
1	-3.790682000	-0.810284000	-1.417739000
1	-1.369254000	3.135811000	0.914288000
1	-4.441055000	0.914009000	-2.676316000
1	-4.800400000	2.633161000	-2.562870000
1	3.726210000	-3.332627000	-0.624916000
1	4.136391000	-1.875729000	-1.445680000
1	1.773029000	5.455727000	-1.647273000
1	2.865682000	4.104645000	-1.491635000
1	2.786305000	6.153265000	0.751291000
1	-0.409080000	-4.825463000	-1.459128000
1	-1.938909000	-5.715912000	-1.540896000
1	3.826075000	0.170420000	2.796510000
1	4.475336000	1.578661000	1.979054000
1	-4.582752000	3.060606000	1.090026000
1	-6.081421000	2.963820000	0.123691000
1	-4.700510000	3.858280000	-0.482811000
1	-3.028575000	-5.830905000	0.691021000
1	-3.612950000	-4.315792000	1.392674000
1	-3.983248000	-4.713071000	-0.292416000

1	6.025662000	-1.057958000	2.316816000
1	6.641128000	0.387222000	1.566997000
1	-1.026762000	5.473489000	1.307347000
1	-1.815486000	5.888853000	-0.233928000
1	1.015177000	-2.047330000	-1.840694000
1	2.194753000	-1.036883000	-2.669077000
1	0.174522000	-2.879298000	-4.067920000
1	1.521232000	-2.019856000	-4.852348000
1	-7.479383000	1.035698000	5.129438000
1	-1.430025000	-2.921022000	6.100803000
1	7.918917000	0.535929000	4.790759000
1	7.280337000	-3.201119000	-1.198197000
1	1.084069000	7.744442000	-1.202650000
1	-7.833594000	0.014523000	-2.896239000
1	-6.178400000	-0.402717000	-3.193655000
1	-1.954058000	-1.692069000	-2.307597000
1	3.408081000	2.515077000	0.088526000
1	4.162800000	5.183428000	0.205703000
6	3.713425000	6.945309000	-0.936681000
8	2.949851000	7.757908000	-1.442236000
8	5.045123000	7.071146000	-1.041154000
1	5.225613000	7.884283000	-1.553311000

f) Ni^{I}, Ni^{II} and Ni^{II}-SCoM for **E**.

NiI-E
NiI-E-opt.gjf.log

Temperature 298.150 Kelvin. Pressure 1.00000 Atm.

Zero-point correction= 0.924258 (Hartree/Particle)
Thermal correction to Energy= 0.983147
Thermal correction to Enthalpy= 0.984091
Thermal correction to Gibbs Free Energy= 0.822719
Sum of electronic and zero-point Energies= -3106.377016
Sum of electronic and thermal Energies= -3106.318127
Sum of electronic and thermal Enthalpies= -3106.317183
Sum of electronic and thermal Free Energies= -3106.478555

28	0.361405000	0.067914000	-0.368578000
7	-1.549380000	-0.750134000	-0.414384000
7	-0.368227000	1.941033000	-0.171702000
7	2.347886000	0.755257000	-0.653367000
7	1.052523000	-1.802201000	-0.197448000
6	-1.900037000	-2.052751000	-0.542593000

6	-3.415628000	-2.229137000	-0.527954000
6	-2.679518000	0.024880000	-0.377855000
6	-3.919909000	-0.785279000	-0.700389000
6	2.476833000	-2.032664000	0.102361000
6	0.409722000	-2.935471000	-0.253029000
6	1.263116000	-4.144514000	0.080629000
6	2.677706000	-3.562720000	0.274632000
6	-0.986104000	-3.125085000	-0.565128000
6	3.393732000	0.058278000	-0.937637000
6	2.739599000	2.176615000	-0.519292000
6	4.254766000	2.127665000	-0.204421000
6	4.677581000	0.894337000	-1.067282000
6	3.347808000	-1.437307000	-1.013925000
6	0.430772000	3.178170000	-0.121646000
6	-1.638049000	2.266556000	-0.006452000
6	-0.430919000	4.231751000	0.652779000
6	-1.855718000	3.757773000	0.232252000
6	1.837785000	2.941879000	0.439517000
6	-2.732048000	1.372178000	-0.127842000
1	2.296826000	3.919388000	0.629406000
1	1.750184000	2.423862000	1.397086000
1	-2.064558000	4.192246000	-0.757304000
6	-3.010390000	4.168681000	1.165399000
1	-2.744885000	5.098241000	1.678685000
1	-3.156913000	3.419720000	1.951020000
6	-4.330926000	4.426620000	0.432679000
1	-4.201423000	5.209687000	-0.326888000
1	-4.696119000	3.551035000	-0.114000000
6	-5.429689000	4.880142000	1.368081000
8	-5.300071000	5.125252000	2.550244000
8	-6.611651000	4.995927000	0.720722000
1	-3.724302000	1.800690000	-0.063829000
6	-0.209111000	4.202493000	2.165168000
1	0.825277000	4.454936000	2.416497000
1	-0.850938000	4.929354000	2.670664000
1	-0.430123000	3.211324000	2.577245000
6	-0.072269000	5.564829000	-0.021035000
7	0.503704000	3.819976000	-1.445475000
6	0.263287000	5.164159000	-1.460250000
8	0.309120000	5.892465000	-2.443314000
1	2.623037000	2.643282000	-1.504432000
1	4.744104000	3.034224000	-0.579813000
6	4.593443000	2.036190000	1.296925000
1	5.682098000	2.020379000	1.414267000
1	4.263700000	2.964195000	1.776355000
6	4.006403000	0.858831000	2.087454000

1	2.920500000	0.783044000	1.958800000
1	4.408719000	-0.102804000	1.747372000
6	4.302528000	0.970606000	3.573434000
8	4.951909000	1.851297000	4.090155000
8	3.785079000	-0.016953000	4.349413000
6	5.971434000	0.195955000	-0.622584000
1	6.264785000	-0.578610000	-1.338035000
1	6.785961000	0.927697000	-0.577651000
1	5.886963000	-0.285405000	0.351922000
6	4.864093000	1.370258000	-2.548949000
1	5.759142000	2.000884000	-2.588123000
1	4.015641000	1.984384000	-2.862029000
6	4.940344000	0.224932000	-3.550726000
8	3.944602000	-0.433838000	-3.850734000
7	6.165152000	-0.028547000	-4.079816000
1	6.261788000	-0.778402000	-4.751586000
1	6.983127000	0.514267000	-3.846494000
1	2.955474000	-1.743814000	-1.989247000
1	4.357224000	-1.838518000	-0.924096000
1	2.707802000	-1.510282000	1.035380000
1	-0.876926000	6.305348000	-0.003135000
1	0.812263000	6.026824000	0.436048000
1	0.716149000	3.310065000	-2.292807000
1	3.348550000	-3.938728000	-0.505973000
6	3.252620000	-3.949205000	1.642680000
1	3.319376000	-5.042143000	1.730202000
1	2.566274000	-3.624157000	2.439140000
6	4.613001000	-3.352118000	1.962089000
8	5.185900000	-2.516997000	1.293761000
8	5.188888000	-3.782130000	3.107866000
1	0.895165000	-4.569790000	1.027353000
6	1.075248000	-5.205640000	-1.002676000
6	-1.429011000	-4.489995000	-0.895880000
6	-0.395210000	-5.624810000	-0.991361000
1	1.353691000	-4.783067000	-1.977241000
1	1.726575000	-6.069840000	-0.824611000
1	-0.584435000	-6.268981000	-0.120059000
1	-0.663129000	-6.218815000	-1.871192000
8	-2.603738000	-4.808189000	-1.120048000
1	-4.739329000	-0.549093000	-0.015023000
1	-3.729419000	-2.883718000	-1.341842000
6	-3.864584000	-2.859176000	0.810341000
1	-3.750277000	-2.119770000	1.612977000
1	-3.213328000	-3.699328000	1.058148000
6	-5.308405000	-3.353770000	0.755629000
1	-5.997095000	-2.569817000	0.420936000

1	-5.403058000	-4.158819000	0.015698000
6	-5.794998000	-3.880188000	2.083413000
8	-5.164798000	-3.896961000	3.122614000
8	-7.062587000	-4.350670000	1.996663000
6	-4.385580000	-0.476513000	-2.133498000
1	-4.524318000	0.605158000	-2.262860000
1	-3.629670000	-0.779485000	-2.866254000
6	-5.705265000	-1.132033000	-2.476848000
8	-6.537864000	-1.513373000	-1.677681000
8	-5.883573000	-1.218498000	-3.812180000
1	-7.269338000	5.301381000	1.374991000
1	-6.765334000	-1.612112000	-3.961312000
1	-7.308339000	-4.669799000	2.886188000
1	4.634093000	-4.449770000	3.547772000
1	3.277392000	-0.646892000	3.810081000

NiII-E
NiII-E-opt.gjf.log

Temperature 298.150 Kelvin. Pressure 1.00000 Atm.

Zero-point correction= 0.926980 (Hartree/Particle)
Thermal correction to Energy= 0.985101
Thermal correction to Enthalpy= 0.986045
Thermal correction to Gibbs Free Energy= 0.826917
Sum of electronic and zero-point Energies= -3106.236734
Sum of electronic and thermal Energies= -3106.178614
Sum of electronic and thermal Enthalpies= -3106.177669
Sum of electronic and thermal Free Energies= -3106.336797

28	-0.340477000	0.056289000	0.360210000
7	1.569291000	-0.701765000	0.403834000
7	0.331404000	1.969549000	0.183028000
7	-2.324482000	0.660319000	0.658680000
7	-0.989453000	-1.813768000	0.100155000
6	1.941682000	-2.004347000	0.544329000
6	3.456473000	-2.144749000	0.537104000
6	2.684553000	0.097563000	0.354103000
6	3.933371000	-0.688413000	0.686572000
6	-2.393834000	-2.049081000	-0.272416000
6	-0.322795000	-2.928591000	0.171563000
6	-1.134569000	-4.141909000	-0.225746000
6	-2.537555000	-3.572461000	-0.541854000
6	1.053254000	-3.088811000	0.553764000
6	-3.331310000	-0.076972000	0.985907000
6	-2.722663000	2.088684000	0.683953000

6	-4.264786000	2.043643000	0.558282000
6	-4.570221000	0.742158000	1.375265000
6	-3.317252000	-1.567308000	0.851619000
6	-0.501195000	3.191643000	0.139846000
6	1.588660000	2.305842000	-0.035419000
6	0.309141000	4.222230000	-0.714704000
6	1.761481000	3.786854000	-0.338704000
6	-1.933104000	2.906416000	-0.330286000
6	2.705545000	1.438747000	0.080107000
1	-2.437251000	3.868343000	-0.472007000
1	-1.905997000	2.399950000	-1.298595000
1	2.002756000	4.252976000	0.628363000
6	2.872105000	4.176721000	-1.332918000
1	2.559587000	5.069911000	-1.881703000
1	3.011065000	3.394084000	-2.086124000
6	4.209652000	4.508604000	-0.663333000
1	4.087067000	5.333600000	0.051226000
1	4.617914000	3.677184000	-0.079070000
6	5.260321000	4.927869000	-1.669106000
8	5.075486000	5.084357000	-2.858570000
8	6.458241000	5.120645000	-1.076043000
1	3.684869000	1.890124000	-0.005668000
6	0.017844000	4.128889000	-2.212265000
1	-1.030317000	4.360649000	-2.422505000
1	0.624842000	4.843180000	-2.774363000
1	0.232535000	3.125773000	-2.598205000
6	-0.036737000	5.576014000	-0.076784000
7	-0.508222000	3.873647000	1.440148000
6	-0.308365000	5.229173000	1.388260000
8	-0.343559000	5.990609000	2.342072000
1	-2.484674000	2.481320000	1.677910000
1	-4.701357000	2.913636000	1.061386000
6	-4.810066000	2.035083000	-0.883343000
1	-5.904292000	2.034449000	-0.823486000
1	-4.543131000	2.975828000	-1.374873000
6	-4.367764000	0.872152000	-1.792272000
1	-3.299873000	0.945226000	-2.028109000
1	-4.505848000	-0.093337000	-1.308740000
6	-5.123230000	0.902127000	-3.115586000
8	-5.272675000	1.919819000	-3.759774000
8	-5.627801000	-0.263955000	-3.558226000
6	-5.923905000	0.074640000	1.087804000
1	-6.094642000	-0.766134000	1.767642000
1	-6.728650000	0.798885000	1.250948000
1	-6.015893000	-0.301558000	0.067551000
6	-4.522011000	1.088949000	2.903947000

1	-5.427908000	1.655023000	3.143265000
1	-3.667369000	1.732164000	3.127538000
6	-4.356036000	-0.136273000	3.794658000
8	-3.300195000	-0.773390000	3.799457000
7	-5.423249000	-0.484783000	4.551216000
1	-5.337821000	-1.268630000	5.185409000
1	-6.267396000	0.067540000	4.588621000
1	-3.022082000	-2.014373000	1.806798000
1	-4.331136000	-1.910503000	0.635119000
1	-2.602267000	-1.464218000	-1.171851000
1	0.757791000	6.322541000	-0.158694000
1	-0.944364000	6.011195000	-0.513395000
1	-0.713220000	3.410022000	2.315275000
1	-3.279775000	-3.999625000	0.140308000
6	-2.949723000	-3.898997000	-1.984028000
1	-3.033746000	-4.985219000	-2.115384000
1	-2.163773000	-3.569380000	-2.680037000
6	-4.231311000	-3.243525000	-2.463322000
8	-4.840308000	-2.377240000	-1.852249000
8	-4.681499000	-3.611127000	-3.669290000
1	-0.682392000	-4.561781000	-1.136178000
6	-1.002611000	-5.193851000	0.878326000
6	1.501486000	-4.453747000	0.912845000
6	0.473709000	-5.594526000	0.966587000
1	-1.353183000	-4.774097000	1.829950000
1	-1.625766000	-6.067541000	0.658278000
1	0.725255000	-6.252289000	0.122338000
1	0.689631000	-6.168679000	1.872938000
8	2.667695000	-4.736667000	1.190459000
1	4.746458000	-0.448638000	-0.004085000
1	3.779111000	-2.781452000	1.361446000
6	3.911233000	-2.789483000	-0.795057000
1	3.771703000	-2.068223000	-1.609801000
1	3.277982000	-3.648617000	-1.022835000
6	5.366178000	-3.250808000	-0.747709000
1	6.044276000	-2.445114000	-0.447212000
1	5.491196000	-4.034255000	0.010352000
6	5.831868000	-3.801837000	-2.074429000
8	5.169282000	-3.862787000	-3.091493000
8	7.111246000	-4.234182000	-2.008562000
6	4.391354000	-0.341695000	2.114823000
1	4.506824000	0.743985000	2.226899000
1	3.644880000	-0.650043000	2.855189000
6	5.726241000	-0.966876000	2.463103000
8	6.528153000	-1.408272000	1.664823000
8	5.943518000	-0.946449000	3.792334000

1	7.085358000	5.400623000	-1.770723000
1	6.830275000	-1.325196000	3.951378000
1	7.346367000	-4.574343000	-2.893357000
1	-4.119924000	-4.297454000	-4.072015000
1	-5.458699000	-1.005322000	-2.932843000

NiII-SCoM-E
NiII-SCoM-E-opt.gjf.log

Temperature 298.150 Kelvin. Pressure 1.00000 Atm.

Zero-point correction= 0.999147 (Hartree/Particle)
Thermal correction to Energy= 1.066487
Thermal correction to Enthalpy= 1.067431
Thermal correction to Gibbs Free Energy= 0.884390
Sum of electronic and zero-point Energies= -4207.148799
Sum of electronic and thermal Energies= -4207.081459
Sum of electronic and thermal Enthalpies= -4207.080515
Sum of electronic and thermal Free Energies= -4207.263557

6	-4.348845000	-0.714704000	2.120408000
6	1.020408000	-3.581744000	2.733584000
6	5.535153000	2.928546000	3.553482000
6	-3.131614000	6.373205000	-1.173622000
7	-2.502281000	-0.392149000	-0.480740000
6	-5.698241000	-0.682781000	2.868262000
6	2.292655000	-3.046430000	3.392714000
7	0.068009000	-1.847474000	0.081140000
8	4.724057000	2.853841000	4.455885000
8	-2.835527000	7.353112000	-0.520261000
7	-0.823994000	2.134010000	-0.485299000
8	-5.145944000	1.159146000	4.250577000
8	1.280882000	-3.504195000	5.557969000
8	-5.751426000	-0.742202000	5.299890000
8	3.359558000	-2.638008000	5.465513000
6	-3.093389000	1.739675000	-1.451563000
6	-2.185998000	-2.487386000	0.862119000
6	2.421220000	-1.559912000	0.570853000
6	1.437484000	3.114683000	-0.311445000
28	-0.350837000	0.096811000	-0.569569000
6	-3.423772000	0.452525000	-0.763842000
6	-0.946482000	-2.920996000	0.043073000
6	2.599916000	-0.250717000	0.244090000
6	0.003643000	3.149260000	-0.464134000
6	-4.833954000	0.007484000	-0.361306000
6	-0.198782000	-4.239704000	0.522926000

6	3.964266000	0.386516000	0.142450000
6	-0.719329000	4.488343000	-0.581763000
6	-4.474922000	-1.147753000	0.648122000
6	1.088794000	-3.672839000	1.190440000
6	3.620774000	1.872379000	0.337497000
6	-2.103913000	4.070714000	-1.089401000
6	-3.132324000	-1.624972000	0.041836000
6	1.189197000	-2.287461000	0.576672000
6	2.145205000	1.915944000	-0.048974000
6	-2.213868000	2.630052000	-0.565801000
6	-5.501766000	-0.562141000	-1.660529000
6	4.593895000	0.117144000	-1.234875000
6	0.070765000	5.463137000	-1.448375000
7	-1.291197000	-3.191586000	-1.347427000
6	-6.789232000	-1.330907000	-1.374323000
6	-0.686244000	-4.301317000	-1.882009000
6	6.034448000	0.562936000	-1.287806000
6	1.427762000	5.675389000	-0.791585000
6	2.169116000	4.397028000	-0.388870000
8	-7.798854000	-0.787083000	-0.929381000
8	-0.774219000	-4.643523000	-3.055494000
8	6.740051000	0.820751000	-0.329012000
6	0.132436000	-4.970976000	-0.802103000
6	3.809088000	2.331280000	1.801166000
7	-6.732392000	-2.668541000	-1.613170000
8	6.490050000	0.626902000	-2.556677000
8	3.371698000	4.532616000	-0.134860000
6	-5.677347000	1.169412000	0.189214000
6	-1.038385000	-5.165533000	1.410221000
6	5.274383000	2.647466000	2.094856000
6	-3.269074000	4.967418000	-0.637965000
6	-5.550797000	-0.134889000	4.268317000
6	2.221448000	-3.103952000	4.901263000
7	1.608789000	0.679091000	-0.036938000
8	6.825301000	3.283924000	3.768535000
8	-3.336730000	6.417204000	-2.509874000
6	1.496182000	-1.058405000	-2.938917000
8	4.279020000	-2.317787000	-3.350156000
16	-0.190722000	-0.290765000	-2.956978000
6	1.657527000	-2.300321000	-3.808315000
8	3.209408000	-4.402241000	-4.232093000
16	3.117765000	-3.260143000	-3.278471000
8	2.817348000	-3.684389000	-1.867995000
1	-3.709077000	-1.429576000	2.647203000
1	-3.848207000	0.256148000	2.196935000
1	0.826141000	-4.580962000	3.134039000

1	0.170567000	-2.957236000	3.034537000
1	-6.425267000	-0.061216000	2.340034000
1	-6.110817000	-1.691859000	2.946086000
1	3.176150000	-3.615012000	3.074243000
1	2.493507000	-2.006824000	3.110840000
1	-2.544490000	1.522155000	-2.375092000
1	-4.004779000	2.279877000	-1.718977000
1	-1.834943000	-1.971609000	1.761088000
1	-2.753174000	-3.366285000	1.180039000
1	3.312852000	-2.137383000	0.784435000
1	4.638540000	0.008444000	0.915000000
1	-0.811741000	4.914495000	0.430977000
1	-5.208404000	-1.958804000	0.589857000
1	1.964738000	-4.271404000	0.917269000
1	4.205765000	2.522826000	-0.310298000
1	-2.083211000	4.038045000	-2.186930000
1	-3.377528000	-2.229307000	-0.840762000
1	-2.631193000	2.639371000	0.453768000
1	-4.790533000	-1.188784000	-2.208128000
1	-5.758593000	0.276924000	-2.315606000
1	4.562002000	-0.943671000	-1.516481000
1	4.036921000	0.630172000	-2.026154000
1	-0.458843000	6.415556000	-1.552441000
1	0.189332000	5.039109000	-2.454142000
1	1.302975000	6.260312000	0.131784000
1	2.112795000	6.246505000	-1.426017000
1	1.174332000	-4.820696000	-1.111376000
1	-0.062752000	-6.046752000	-0.763564000
1	3.449430000	1.545118000	2.476621000
1	3.206250000	3.220986000	1.994975000
1	-5.230672000	1.616266000	1.081865000
1	-6.686888000	0.827417000	0.419556000
1	-5.777954000	1.957586000	-0.563807000
1	-0.450265000	-6.048086000	1.685233000
1	-1.382210000	-4.691953000	2.333382000
1	-1.919336000	-5.517053000	0.860860000
1	5.935164000	1.831864000	1.776540000
1	5.591788000	3.522195000	1.513854000
1	-3.302493000	5.027426000	0.453156000
1	-4.216139000	4.546999000	-0.994997000
1	1.698569000	-1.348594000	-1.911127000
1	2.239499000	-0.309881000	-3.222431000
1	0.806157000	-2.977616000	-3.717135000
1	1.796895000	-2.055990000	-4.866227000
1	-5.055560000	1.445221000	5.179869000
1	3.244039000	-2.696382000	6.433359000

1	6.919030000	3.440242000	4.727751000
1	7.432337000	0.878981000	-2.510371000
1	-3.188966000	7.339436000	-2.795682000
1	-7.566166000	-3.222468000	-1.468057000
1	-5.934430000	-3.114415000	-2.041504000
1	-1.421255000	-2.392179000	-1.972470000

iv) Complexes considered in proposed thermodynamic cycle for the third step of catalytic cycle

g) SCoB-radical, SCoM-anion, SCoM-radical and CoMS-SCoB.

SCoB-radical
SCoB-radical-opt.gjf.log

Temperature 298.150 Kelvin. Pressure 1.00000 Atm.

Zero-point correction= 0.152122 (Hartree/Particle)
Thermal correction to Energy= 0.159779
Thermal correction to Enthalpy= 0.160723
Thermal correction to Gibbs Free Energy= 0.119101
Sum of electronic and zero-point Energies= -595.200026
Sum of electronic and thermal Energies= -595.192370
Sum of electronic and thermal Enthalpies= -595.191426
Sum of electronic and thermal Free Energies= -595.233047

6	2.017774000	-1.114250000	-0.536584000
6	1.550203000	-0.301251000	0.675548000
6	1.131743000	1.138940000	0.342308000
6	-0.085914000	1.301747000	-0.583987000
6	-1.419433000	0.847005000	0.032421000
16	-1.729007000	-0.946718000	0.070172000
1	1.211852000	-1.251221000	-1.265286000
1	2.361596000	-2.109725000	-0.233340000
1	0.722260000	-0.827968000	1.175556000
1	2.358586000	-0.260096000	1.418056000
1	0.930152000	1.679844000	1.277779000
1	1.985018000	1.648120000	-0.128110000
1	-0.192709000	2.368927000	-0.821303000
1	0.073141000	0.784151000	-1.536890000
1	-2.250813000	1.230962000	-0.579401000
1	-1.551281000	1.262823000	1.037373000
1	2.850078000	-0.611471000	-1.045415000

SCoM-anion
SCoM-anion-opt.gjf.log

Temperature 298.150 Kelvin. Pressure 1.00000 Atm.

Zero-point correction= 0.070035 (Hartree/Particle)
Thermal correction to Energy= 0.077423
Thermal correction to Enthalpy= 0.078368
Thermal correction to Gibbs Free Energy= 0.036923
Sum of electronic and zero-point Energies= -1100.801029
Sum of electronic and thermal Energies= -1100.793641
Sum of electronic and thermal Enthalpies= -1100.792697
Sum of electronic and thermal Free Energies= -1100.834141

6	1.391729000	-0.504391000	0.000149000
8	-1.522555000	-0.811286000	-1.248945000
16	3.141533000	0.136692000	-0.000030000
6	0.347541000	0.608435000	-0.000828000
8	-2.216381000	1.238993000	-0.000941000
16	-1.373016000	0.001002000	-0.000019000
8	-1.521822000	-0.808609000	1.250745000
1	1.226968000	-1.133025000	0.881320000
1	1.227433000	-1.133996000	-0.880440000
1	0.449947000	1.244277000	0.884717000
1	0.449826000	1.242595000	-0.887601000

SCoM-radical

SCoM-radical-opt.gjf.log

Temperature 298.150 Kelvin. Pressure 1.00000 Atm.

Zero-point correction= 0.068871 (Hartree/Particle)
Thermal correction to Energy= 0.076456
Thermal correction to Enthalpy= 0.077401
Thermal correction to Gibbs Free Energy= 0.034821
Sum of electronic and zero-point Energies= -1100.681361
Sum of electronic and thermal Energies= -1100.673775
Sum of electronic and thermal Enthalpies= -1100.672831
Sum of electronic and thermal Free Energies= -1100.715411

6	1.357671000	-0.420942000	0.000108000
8	-1.425645000	-0.826895000	-1.249453000
16	3.090058000	0.116913000	-0.000029000
6	0.322200000	0.694781000	0.000220000
8	-2.270614000	1.171085000	-0.000601000
16	-1.362269000	-0.010374000	-0.000011000
8	-1.426346000	-0.826194000	1.249813000
1	1.215187000	-1.070379000	0.874863000
1	1.215173000	-1.070101000	-0.874885000

1	0.413225000	1.324319000	0.889832000
1	0.413403000	1.324544000	-0.889207000

CoMS-SCoB
CoMS-SCoB-opt.gjf.log

Temperature 298.150 Kelvin. Pressure 1.00000 Atm.

Zero-point correction= 0.225622 (Hartree/Particle)
Thermal correction to Energy= 0.242047
Thermal correction to Enthalpy= 0.242991
Thermal correction to Gibbs Free Energy= 0.178245
Sum of electronic and zero-point Energies= -1695.995149
Sum of electronic and thermal Energies= -1695.978724
Sum of electronic and thermal Enthalpies= -1695.977780
Sum of electronic and thermal Free Energies= -1696.042526

16	-1.309502000	-2.672622000	0.541748000
6	-2.727184000	-1.581161000	0.081783000
1	-3.026394000	-1.820469000	-0.943524000
1	-3.527200000	-1.922876000	0.751367000
6	-2.476017000	-0.084244000	0.248239000
1	-1.664927000	0.221227000	-0.424851000
1	-2.138457000	0.121121000	1.272493000
6	-3.727679000	0.747237000	-0.065419000
1	-4.545019000	0.446325000	0.605842000
1	-4.068284000	0.524488000	-1.087019000
6	-3.485218000	2.255849000	0.071457000
1	-2.666263000	2.551491000	-0.599384000
1	-3.140704000	2.474743000	1.091962000
6	-4.732861000	3.088634000	-0.240600000
1	-5.556658000	2.831857000	0.437092000
1	-4.534555000	4.161617000	-0.136112000
1	-5.078827000	2.909369000	-1.266279000
16	0.209645000	-2.129172000	-0.791830000
6	1.135079000	-0.816466000	0.125718000
1	0.453697000	0.002131000	0.362861000
1	1.523762000	-1.241486000	1.053659000
6	2.276883000	-0.313133000	-0.746367000
1	1.912217000	0.120025000	-1.682790000
1	2.993182000	-1.106507000	-0.980770000
16	3.209345000	1.003166000	0.110027000
8	4.277944000	1.390944000	-0.855851000
8	2.210468000	2.083324000	0.366156000
8	3.728414000	0.366321000	1.356880000

b) Ni^{II}-SCoM, Ni^{II}, Ni^{I} and for **A**.

NiII-SCoM-A
NiII-SCoM-A-opt.gjf.log

Temperature 298.150 Kelvin. Pressure 1.00000 Atm.

Zero-point correction= 0.914356 (Hartree/Particle)
Thermal correction to Energy= 0.982875
Thermal correction to Enthalpy= 0.983819
Thermal correction to Gibbs Free Energy= 0.798745
Sum of electronic and zero-point Energies= -4319.287511
Sum of electronic and thermal Energies= -4319.218992
Sum of electronic and thermal Enthalpies= -4319.218048
Sum of electronic and thermal Free Energies= -4319.403123

6	-5.412655000	-0.841081000	1.162476000
6	0.282614000	-4.389863000	2.536661000
6	6.527326000	3.519989000	-0.312827000
6	-2.308813000	6.125109000	-1.655187000
7	-2.504631000	0.260876000	0.042975000
6	-6.930161000	-1.078288000	1.021935000
6	1.540237000	-4.274158000	3.418229000
7	-0.373383000	-1.673860000	0.772312000
8	6.598156000	4.534416000	-0.979013000
8	-1.630419000	7.013758000	-2.132931000
7	-0.572350000	2.463787000	0.302538000
8	-7.521016000	-0.132555000	3.112487000
8	1.918342000	-5.785940000	5.286362000
8	-8.132268000	-2.273658000	2.767151000
8	0.343181000	-4.187502000	5.465466000
6	-2.969544000	2.639290000	-0.256880000
6	-2.676722000	-2.176348000	0.154076000
6	2.013542000	-1.833400000	1.293134000
6	1.795692000	2.993570000	0.714681000
28	-0.377668000	0.391464000	0.253659000
6	-3.328656000	1.319854000	-0.270672000
6	-1.360892000	-2.528809000	0.444392000
6	2.390601000	-0.464254000	1.232129000
6	0.472719000	3.357395000	0.441551000
6	-4.685304000	0.802959000	-0.777047000
6	-0.801331000	-3.945538000	0.231732000
6	3.743693000	0.033788000	1.377239000
6	-0.010586000	4.701442000	0.271378000
6	-4.686933000	-0.643846000	-0.189693000
6	0.493783000	-3.868699000	1.095974000

6	3.681273000	1.402371000	1.219447000
6	-1.373214000	4.594836000	0.047363000
6	-3.195050000	-0.887154000	0.010899000
6	0.773664000	-2.374120000	1.066916000
6	2.295509000	1.721191000	0.968488000
6	-1.689638000	3.190234000	0.041587000
6	-4.536511000	0.730009000	-2.340255000
6	4.986797000	-0.815267000	1.457377000
6	0.786991000	5.971120000	0.369122000
6	-5.737525000	0.123593000	-3.025224000
6	0.435524000	-5.003407000	-1.865531000
6	5.168855000	-1.441655000	0.075348000
8	-6.776130000	0.704085000	-3.279165000
8	1.071818000	-5.839241000	-1.252998000
8	5.580275000	-0.820315000	-0.891081000
6	-0.467919000	-3.935960000	-1.284198000
6	4.820998000	2.376694000	1.197997000
8	4.741311000	-2.703242000	0.037991000
6	-5.869985000	1.702808000	-0.407307000
6	-1.748516000	-5.105609000	0.548604000
6	5.353187000	2.577367000	-0.244572000
6	-2.335983000	5.732021000	-0.191708000
6	-7.596172000	-1.260708000	2.364480000
6	1.321217000	-4.847865000	4.795551000
7	1.540201000	0.561549000	0.988562000
8	7.551406000	3.110061000	0.479340000
8	-3.101489000	5.326442000	-2.405922000
6	1.764137000	-0.942156000	-2.221320000
8	4.396664000	-2.301349000	-3.979229000
16	0.195921000	0.051003000	-2.187373000
6	1.802812000	-1.870321000	-3.436987000
8	2.789583000	-4.186302000	-4.319986000
16	3.219818000	-3.030552000	-3.449696000
8	3.379725000	-3.495133000	-2.022200000
1	-4.976128000	-1.718460000	1.653729000
1	-5.220604000	0.011532000	1.822293000
1	-0.010897000	-5.444594000	2.489254000
1	-0.540848000	-3.845341000	3.013416000
1	-7.407551000	-0.232330000	0.520358000
1	-7.119761000	-1.977967000	0.430610000
1	2.383031000	-4.804706000	2.966754000
1	1.816883000	-3.219851000	3.526644000
1	2.817846000	-2.540122000	1.451195000
1	-5.096239000	-1.367535000	-0.902024000
1	1.307154000	-4.433916000	0.637063000
1	-3.640275000	0.154422000	-2.588379000

1	-4.415612000	1.748580000	-2.718620000
1	5.859544000	-0.196015000	1.682060000
1	4.904957000	-1.599788000	2.216041000
1	0.310675000	6.747148000	-0.239056000
1	1.794628000	5.831643000	-0.038941000
1	-1.398861000	-3.928055000	-1.862865000
1	0.020381000	-2.987888000	-1.535998000
1	5.641623000	2.014456000	1.825980000
1	4.508995000	3.342567000	1.614092000
1	-5.973109000	1.807014000	0.676395000
1	-6.806733000	1.320410000	-0.819692000
1	-5.731300000	2.704412000	-0.826509000
1	-1.224630000	-6.057901000	0.414953000
1	-2.137810000	-5.058731000	1.569692000
1	-2.604801000	-5.102478000	-0.134817000
1	5.657709000	1.598827000	-0.636543000
1	4.565689000	2.973959000	-0.889724000
1	-2.050771000	6.613805000	0.388265000
1	-3.354015000	5.450075000	0.092780000
1	1.821702000	-1.541890000	-1.312692000
1	2.635114000	-0.278736000	-2.225941000
1	0.913867000	-2.501876000	-3.474020000
1	1.867549000	-1.313795000	-4.376943000
1	-7.946965000	-0.328922000	3.968816000
1	0.255584000	-4.621771000	6.335353000
1	8.263353000	3.770295000	0.378656000
1	4.443845000	-2.963657000	-0.890702000
1	-2.968190000	5.580325000	-3.339482000
1	-6.388243000	-1.508125000	-3.717792000
1	1.386877000	-4.978308000	-3.577720000
1	2.518701000	3.801494000	0.742261000
6	0.922893000	6.518306000	1.819078000
1	1.401834000	7.502346000	1.785820000
1	-0.063506000	6.615762000	2.279912000
6	1.804505000	5.615454000	2.648679000
8	3.012660000	5.531147000	2.525112000
8	1.107160000	4.859239000	3.522366000
1	1.743256000	4.249371000	3.944824000
1	-3.375288000	-2.988796000	-0.019724000
1	-3.727286000	3.353546000	-0.558141000
8	0.466579000	-4.858789000	-3.206708000
8	-5.558801000	-1.188930000	-3.311746000

NiII-A

NiII-A-opt.gjf.log

Temperature 298.150 Kelvin. Pressure 1.00000 Atm.

Zero-point correction= 0.844882 (Hartree/Particle)
Thermal correction to Energy= 0.904082
Thermal correction to Enthalpy= 0.905026
Thermal correction to Gibbs Free Energy= 0.742070
Sum of electronic and zero-point Energies= -3218.437295
Sum of electronic and thermal Energies= -3218.378095
Sum of electronic and thermal Enthalpies= -3218.377151
Sum of electronic and thermal Free Energies= -3218.540106

28	-0.215413000	-0.357028000	-0.423306000
7	0.617096000	1.382855000	-0.563139000
7	1.547156000	-1.202226000	-0.185335000
7	-1.053891000	-2.110492000	-0.344917000
7	-1.965041000	0.475538000	-0.498079000
6	0.029952000	2.624746000	-0.334971000
6	1.001232000	3.665970000	-0.531366000
6	1.912713000	1.629571000	-0.893714000
6	2.164671000	3.044400000	-0.932204000
6	-3.158771000	-0.148306000	-0.685664000
6	-2.265498000	1.788082000	-0.144164000
6	-3.686579000	1.954500000	-0.043178000
6	-4.243347000	0.741721000	-0.401922000
6	-1.316151000	2.798372000	-0.061099000
6	-2.311507000	-2.423407000	-0.856249000
6	-0.522084000	-3.224442000	0.193298000
6	-1.526291000	-4.362562000	0.197939000
6	-2.437120000	-3.932106000	-0.994766000
6	-3.299496000	-1.514969000	-1.037060000
6	1.758371000	-2.403917000	0.397430000
6	2.765400000	-0.634329000	-0.517614000
6	3.230461000	-2.607242000	0.740980000
6	3.901781000	-1.604983000	-0.249221000
6	0.779590000	-3.349053000	0.655156000
6	2.922037000	0.640803000	-0.962843000
1	4.115115000	-2.156319000	-1.176031000
6	5.212951000	-0.968615000	0.257794000
1	5.736502000	-1.677039000	0.905970000
1	4.984273000	-0.092332000	0.873139000
6	6.198661000	-0.573455000	-0.864783000
1	6.467459000	-1.461933000	-1.442383000
1	5.772819000	0.172476000	-1.537883000
6	7.432701000	0.019337000	-0.231763000
8	7.571938000	1.192467000	0.088080000
8	8.372935000	-0.901761000	0.025608000

1	3.913887000	0.970240000	-1.234629000
6	3.407718000	-2.208853000	2.225435000
1	2.754208000	-2.824146000	2.852032000
1	4.435334000	-2.362609000	2.562737000
1	3.137838000	-1.158785000	2.377411000
1	-1.031632000	-5.316233000	-0.016387000
6	-2.224986000	-4.506539000	1.571670000
1	-2.962379000	-5.313950000	1.500539000
1	-1.480409000	-4.840385000	2.301847000
6	-2.904825000	-3.242454000	2.113863000
1	-2.172038000	-2.434978000	2.249137000
1	-3.643435000	-2.846241000	1.415148000
6	-3.570006000	-3.485795000	3.460168000
8	-3.253897000	-4.381230000	4.217756000
8	-4.548176000	-2.625870000	3.801633000
6	-3.865761000	-4.480129000	-0.965949000
1	-4.412982000	-4.188338000	-1.866562000
1	-3.846029000	-5.574700000	-0.933414000
1	-4.427223000	-4.122559000	-0.099862000
6	-1.715036000	-4.396958000	-2.311359000
1	-1.864349000	-5.474192000	-2.429817000
1	-0.643977000	-4.195687000	-2.230391000
1	9.109535000	-0.458009000	0.490904000
1	-4.742074000	-1.983100000	3.081282000
1	-1.664131000	3.801903000	0.158605000
6	3.696492000	-4.069089000	0.501658000
1	3.373397000	-4.399206000	-0.489009000
1	3.257536000	-4.727871000	1.255373000
6	5.199073000	-4.201469000	0.617677000
8	5.813955000	-4.332373000	1.656719000
1	1.061409000	-4.283579000	1.124407000
6	3.472595000	3.688870000	-1.304267000
1	3.752801000	3.448584000	-2.334705000
1	3.378282000	4.777884000	-1.237639000
6	0.814762000	5.129381000	-0.259993000
1	-0.241108000	5.413929000	-0.301976000
1	1.316466000	5.730225000	-1.026664000
6	4.611123000	3.279227000	-0.376890000
8	4.593481000	3.403026000	0.832936000
8	5.632446000	2.737908000	-1.061994000
1	6.318908000	2.368007000	-0.447954000
6	1.389867000	5.500764000	1.117861000
1	0.854756000	4.973871000	1.917425000
1	2.435391000	5.176120000	1.201102000
6	1.321267000	6.984030000	1.395826000
8	0.906947000	7.831607000	0.630287000

8	1.790279000	7.279401000	2.629632000
1	1.722278000	8.247621000	2.738207000
6	-4.391315000	3.230323000	0.313730000
1	-3.833623000	3.777978000	1.080998000
1	-5.370167000	3.014455000	0.754841000
6	-5.673171000	0.305342000	-0.322779000
1	-6.343577000	1.157728000	-0.157293000
1	-6.027867000	-0.177403000	-1.241060000
6	-4.587159000	4.141563000	-0.909105000
1	-5.165323000	3.629724000	-1.688457000
1	-3.624919000	4.392547000	-1.371495000
6	-5.298596000	5.430502000	-0.564636000
8	-5.718126000	5.737632000	0.532804000
8	-5.423882000	6.230479000	-1.647921000
1	-5.889223000	7.038337000	-1.356672000
6	-5.901265000	-0.662479000	0.830302000
8	-5.146404000	-0.801025000	1.779432000
8	-7.042353000	-1.352390000	0.694803000
1	-7.145769000	-1.929929000	1.476470000
1	-4.273974000	-1.862398000	-1.360874000
8	5.812874000	-4.105589000	-0.585660000
1	6.775790000	-4.153587000	-0.423853000
6	-2.201158000	-3.661243000	-3.537150000
8	-1.721746000	-2.629719000	-3.962163000
8	-3.271806000	-4.258650000	-4.110630000
1	-3.541002000	-3.700991000	-4.866741000

NiI-A

NiI-A-opt.gjf.log

Temperature 298.150 Kelvin. Pressure 1.00000 Atm.

Zero-point correction= 0.839162 (Hartree/Particle)
Thermal correction to Energy= 0.900511
Thermal correction to Enthalpy= 0.901455
Thermal correction to Gibbs Free Energy= 0.727098
Sum of electronic and zero-point Energies= -3218.519819
Sum of electronic and thermal Energies= -3218.458469
Sum of electronic and thermal Enthalpies= -3218.457525
Sum of electronic and thermal Free Energies= -3218.631882

6	5.042923000	-1.311170000	1.039976000
6	1.909202000	4.495215000	1.424308000
6	-7.563337000	0.927317000	-1.122287000
6	-0.802230000	-6.076067000	-0.924777000
7	1.859058000	-1.072866000	-0.104385000

6	6.417113000	-1.985054000	1.018140000
6	0.735404000	5.190423000	2.138020000
7	1.073110000	1.749147000	-0.139582000
8	-8.290252000	0.988778000	-0.149743000
8	0.328271000	-6.347544000	-1.281907000
7	-0.944301000	-1.802361000	0.217870000
8	8.455868000	-2.248473000	2.197317000
8	1.110424000	6.790018000	3.930098000
8	7.045846000	-0.672849000	2.972571000
8	1.447859000	4.597848000	4.321219000
6	0.981037000	-3.337349000	0.117004000
6	3.316292000	0.849693000	-0.468777000
6	-0.838986000	3.273633000	0.000335000
6	-3.247293000	-0.938545000	0.308487000
28	0.065562000	-0.034831000	0.036353000
6	2.000444000	-2.443589000	-0.064340000
6	2.389918000	1.884308000	-0.407802000
6	-1.895527000	2.328708000	0.076287000
6	-2.317483000	-1.976046000	0.309949000
6	3.440977000	-2.875216000	-0.394448000
6	2.755035000	3.342409000	-0.728165000
6	-3.299720000	2.660821000	0.150501000
6	-2.640262000	-3.381039000	0.397979000
6	4.207576000	-1.522696000	-0.242772000
6	1.577811000	4.080397000	-0.029305000
6	-3.988305000	1.468097000	0.242400000
6	-1.434768000	-4.050237000	0.376783000
6	3.067235000	-0.510570000	-0.286184000
6	0.502045000	3.004317000	-0.065414000
6	-2.987570000	0.428476000	0.226554000
6	-0.401712000	-3.047790000	0.247070000
6	3.432818000	-3.305519000	-1.883615000
6	-3.886222000	4.048132000	0.094560000
6	-4.019045000	-3.958518000	0.536592000
6	4.778266000	-3.681349000	-2.468270000
6	2.931827000	4.868428000	-2.788032000
6	-3.964746000	4.524518000	-1.343941000
6	-4.542591000	-3.865640000	1.978660000
8	5.871662000	-3.525811000	-1.958347000
8	4.041954000	5.292416000	-3.045594000
8	-4.809581000	4.189734000	-2.152657000
6	2.618776000	3.480001000	-2.287024000
6	-5.474925000	1.267654000	0.281744000
8	-2.946777000	5.355066000	-1.665067000
6	3.917585000	-4.048221000	0.476546000
6	4.164119000	3.755759000	-0.286441000

6	-6.068042000	1.127766000	-1.129649000
6	-1.198299000	-5.538591000	0.437329000
6	7.297133000	-1.544592000	2.164469000
6	1.104060000	5.642440000	3.529109000
7	-1.720201000	0.981606000	0.125594000
8	-8.039368000	0.671440000	-2.365540000
8	-1.871108000	-6.191916000	-1.746069000
1	5.200413000	-0.235112000	1.166643000
1	4.475292000	-1.632041000	1.921417000
1	2.765264000	5.179817000	1.414111000
1	2.207832000	3.612312000	2.000376000
1	6.353974000	-3.075526000	1.044864000
1	6.946217000	-1.753580000	0.084486000
1	0.406125000	6.072116000	1.580723000
1	-0.109279000	4.497072000	2.212564000
1	1.240387000	-4.389487000	0.088776000
1	4.348176000	1.121866000	-0.667111000
1	-1.122860000	4.320983000	-0.012119000
1	4.878627000	-1.364838000	-1.093398000
1	1.273244000	4.974708000	-0.582349000
1	3.032825000	-2.492071000	-2.504681000
1	2.750536000	-4.151310000	-2.028208000
1	-4.907653000	4.043523000	0.486949000
1	-3.295000000	4.757801000	0.681551000
1	-4.029693000	-5.006187000	0.217656000
1	-4.722796000	-3.440354000	-0.125491000
1	-3.881914000	-4.404523000	2.669391000
1	3.327800000	2.793159000	-2.756459000
1	1.603275000	3.200943000	-2.581666000
1	-5.964640000	2.111381000	0.782146000
1	-5.733435000	0.380340000	0.870743000
1	3.901849000	-3.791578000	1.539599000
1	4.927437000	-4.358338000	0.201018000
1	3.261811000	-4.914084000	0.337795000
1	4.312600000	4.834547000	-0.395023000
1	4.349770000	3.480721000	0.755228000
1	4.923360000	3.265640000	-0.904155000
1	-5.846571000	2.027475000	-1.717976000
1	-5.606158000	0.290691000	-1.666905000
1	-2.104716000	-6.054637000	0.768565000
1	-0.390133000	-5.786746000	1.131734000
1	8.974578000	-1.902655000	2.948866000
1	1.681431000	4.967213000	5.194250000
1	-9.006748000	0.568173000	-2.281685000
1	-3.030983000	5.559267000	-2.616729000
1	-1.534370000	-6.485354000	-2.614376000

1	5.530833000	-4.424027000	-4.029432000
1	2.120631000	6.526897000	-3.181189000
1	-4.292909000	-1.227421000	0.375549000
1	-4.544174000	-2.824760000	2.324895000
6	-5.940247000	-4.416427000	2.137402000
8	-6.625954000	-4.891721000	1.254223000
8	-6.369767000	-4.322156000	3.419100000
1	-7.274104000	-4.689931000	3.442763000
8	1.822488000	5.641049000	-2.896591000
8	4.633028000	-4.226030000	-3.699491000

c) Ni^{II}-SCoM, Ni^{II}, Ni^{I} and for **B**.

NiII-SCoM-B
NiII-SCoM-B.gjf.log

Temperature 298.150 Kelvin. Pressure 1.00000 Atm.

Zero-point correction= 0.940166 (Hartree/Particle)
Thermal correction to Energy= 1.009376
Thermal correction to Enthalpy= 1.010320
Thermal correction to Gibbs Free Energy= 0.824855
Sum of electronic and zero-point Energies= -4279.529468
Sum of electronic and thermal Energies= -4279.460259
Sum of electronic and thermal Enthalpies= -4279.459315
Sum of electronic and thermal Free Energies= -4279.644780

6	-3.916532000	2.557509000	2.255147000
6	-0.830472000	-3.913035000	2.790369000
6	7.514890000	-0.573526000	0.727705000
6	0.843920000	6.388853000	-0.155976000
7	-1.748507000	1.382603000	0.137566000
6	-5.206214000	3.291125000	2.628864000
6	0.584372000	-4.489323000	2.853262000
7	-0.963853000	-1.459455000	0.530161000
8	8.277439000	-0.824604000	-0.185000000
8	-0.250593000	6.754846000	0.222206000
7	0.951929000	2.040249000	-0.750125000
8	-6.339568000	4.472969000	4.342551000
8	2.236337000	-5.446625000	4.312392000
8	-4.332467000	3.603434000	4.880388000
8	0.291556000	-4.790102000	5.220167000
6	-1.017006000	3.524920000	-0.775681000
6	-3.082501000	-0.418912000	1.123113000
6	0.885954000	-3.002250000	0.165338000

6	3.225384000	1.122468000	-0.967272000
28	-0.059067000	0.257348000	-0.453652000
6	-1.979113000	2.665295000	-0.319593000
6	-2.232101000	-1.510977000	0.974719000
6	1.904462000	-2.094386000	-0.240901000
6	2.303960000	2.177129000	-0.995293000
6	-3.491125000	2.925551000	-0.324427000
6	-2.681475000	-2.958915000	1.215314000
6	3.305037000	-2.439030000	-0.358318000
6	2.599334000	3.553557000	-1.303922000
6	-3.931471000	1.968451000	0.825874000
6	-1.298491000	-3.678475000	1.327801000
6	3.980274000	-1.270608000	-0.642030000
6	1.390525000	4.227355000	-1.258838000
6	-2.858889000	0.894207000	0.703637000
6	-0.383111000	-2.698734000	0.595122000
6	2.982361000	-0.226191000	-0.707581000
6	0.380653000	3.258688000	-0.907969000
6	-4.001660000	2.338728000	-1.691457000
6	3.886208000	-3.790287000	-0.046317000
6	3.967443000	4.127050000	-1.529289000
7	-5.764755000	-2.914039000	-0.142742000
6	-5.504299000	2.092572000	-1.721943000
6	-4.513705000	-2.639737000	-0.610324000
6	3.824293000	-4.080896000	1.451144000
6	4.675654000	4.424983000	-0.197483000
8	-6.327378000	3.014656000	-1.716734000
8	-4.309320000	-1.707484000	-1.391793000
8	4.131138000	-3.285815000	2.322369000
6	-3.387685000	-3.509962000	-0.075153000
6	5.466166000	-1.071457000	-0.694828000
7	-5.881501000	0.787844000	-1.740516000
8	3.365829000	-5.316539000	1.721212000
6	-3.895842000	4.393952000	-0.193485000
6	-3.611085000	-3.130122000	2.424646000
6	6.012895000	-0.707401000	0.696049000
6	1.144821000	5.698254000	-1.471805000
6	-5.207614000	3.782661000	4.057487000
6	1.120057000	-4.957459000	4.178940000
7	1.733889000	-0.768540000	-0.451916000
8	7.952064000	-0.133757000	1.933811000
8	1.958576000	6.533482000	0.603925000
6	-0.974957000	-1.931556000	-2.721149000
8	-0.054211000	-4.880232000	-3.370028000
16	-0.789556000	-0.097300000	-2.821016000
6	-1.231847000	-2.601335000	-4.062843000

8	-1.664710000	-4.904246000	-5.283614000
16	-1.372191000	-4.413685000	-3.901664000
8	-2.503132000	-4.649980000	-2.945015000
1	-3.775169000	1.735651000	2.966058000
1	-3.051635000	3.218972000	2.381840000
1	-1.529567000	-4.597735000	3.281141000
1	-0.861209000	-2.969566000	3.347680000
1	-5.401294000	4.151227000	1.981360000
1	-6.075569000	2.630791000	2.506433000
1	0.695575000	-5.339393000	2.169724000
1	1.304883000	-3.744326000	2.504630000
1	-1.338082000	4.524494000	-1.047985000
1	-4.076275000	-0.620590000	1.508338000
1	1.181601000	-4.043512000	0.228406000
1	-4.922568000	1.540873000	0.643042000
1	-1.320624000	-4.650961000	0.821870000
1	-3.453663000	1.418296000	-1.917704000
1	-3.765369000	3.059991000	-2.480629000
1	3.378305000	-4.595137000	-0.586404000
1	4.943118000	-3.824832000	-0.337145000
1	3.909711000	5.053452000	-2.111357000
1	4.586222000	3.440458000	-2.118530000
1	4.076835000	5.125770000	0.397660000
1	-2.670865000	-3.619445000	-0.891716000
1	-3.758886000	-4.515787000	0.157909000
1	5.736536000	-0.285302000	-1.408931000
1	5.967282000	-1.980853000	-1.044303000
1	-3.512764000	4.841464000	0.728982000
1	-4.983338000	4.493817000	-0.231934000
1	-3.491569000	4.972485000	-1.031539000
1	-3.798757000	-4.193097000	2.616497000
1	-3.190675000	-2.685012000	3.330646000
1	-4.579127000	-2.655383000	2.239922000
1	5.571884000	0.231759000	1.051969000
1	5.714061000	-1.470584000	1.426918000
1	0.289119000	5.873458000	-2.130562000
1	2.025769000	6.168870000	-1.920358000
1	-1.809355000	-2.147899000	-2.054885000
1	-0.069252000	-2.350694000	-2.279465000
1	-2.168066000	-2.255187000	-4.513689000
1	-0.417567000	-2.416680000	-4.771473000
1	-6.271067000	4.752945000	5.275412000
1	0.756357000	-5.110692000	6.018013000
1	8.925822000	-0.086353000	1.879476000
1	3.207955000	-5.391006000	2.697285000
1	1.676344000	6.926594000	1.451851000

1	-6.873916000	0.591916000	-1.727832000
1	-5.240477000	-0.003716000	-1.671566000
1	-6.548525000	-2.422809000	-0.552286000
1	4.258326000	1.387756000	-1.177062000
1	4.766078000	3.515019000	0.407983000
6	6.051386000	5.018509000	-0.377874000
8	6.556220000	5.351810000	-1.431856000
8	6.693832000	5.158237000	0.808157000
1	7.565233000	5.552713000	0.611752000
1	-5.960400000	-3.766674000	0.360920000

NiII-B

NiII-B-opt.gjf.log

Temperature 298.150 Kelvin. Pressure 1.00000 Atm.

Zero-point correction= 0.868728 (Hartree/Particle)
Thermal correction to Energy= 0.929024
Thermal correction to Enthalpy= 0.929968
Thermal correction to Gibbs Free Energy= 0.765686
Sum of electronic and zero-point Energies= -3178.683341
Sum of electronic and thermal Energies= -3178.623046
Sum of electronic and thermal Enthalpies= -3178.622102
Sum of electronic and thermal Free Energies= -3178.786383

28	-0.218224000	-0.342829000	-0.411780000
7	0.635928000	1.385790000	-0.561671000
7	1.532740000	-1.208624000	-0.165396000
7	-1.080305000	-2.084288000	-0.319989000
7	-1.956412000	0.511157000	-0.495248000
6	0.063105000	2.636346000	-0.344452000
6	1.046591000	3.664000000	-0.548321000
6	1.935254000	1.613972000	-0.891829000
6	2.203489000	3.025233000	-0.941977000
6	-3.156303000	-0.100651000	-0.679513000
6	-2.242450000	1.829224000	-0.148602000
6	-3.662065000	2.011451000	-0.047177000
6	-4.231003000	0.802965000	-0.399690000
6	-1.281464000	2.828450000	-0.072629000
6	-2.338770000	-2.387221000	-0.833450000
6	-0.564617000	-3.198584000	0.233632000
6	-1.585983000	-4.321050000	0.252121000
6	-2.485687000	-3.895514000	-0.951613000
6	-3.315055000	-1.467489000	-1.021244000
6	1.728340000	-2.408157000	0.426447000
6	2.759161000	-0.659103000	-0.500813000

6	3.197840000	-2.635966000	0.765147000
6	3.881719000	-1.645234000	-0.228878000
6	0.735219000	-3.336015000	0.695976000
6	2.932383000	0.611730000	-0.952346000
1	4.083036000	-2.200176000	-1.157330000
6	5.205173000	-1.029529000	0.272651000
1	5.717273000	-1.751111000	0.914052000
1	4.990791000	-0.149871000	0.888510000
6	6.192890000	-0.645361000	-0.852754000
1	6.456567000	-1.538187000	-1.426305000
1	5.773268000	0.101847000	-1.528672000
6	7.431359000	-0.058857000	-0.222312000
8	7.571475000	1.111410000	0.109181000
8	8.376070000	-0.979352000	0.017495000
1	3.928914000	0.927214000	-1.223571000
6	3.384833000	-2.232237000	2.247304000
1	2.717059000	-2.830195000	2.876205000
1	4.408678000	-2.413834000	2.578558000
1	3.137242000	-1.175660000	2.394481000
1	-1.103767000	-5.284511000	0.053202000
6	-2.289811000	-4.438401000	1.625412000
1	-3.039713000	-5.234945000	1.561118000
1	-1.552914000	-4.776398000	2.361463000
6	-2.951970000	-3.159369000	2.155096000
1	-2.205815000	-2.364748000	2.293102000
1	-3.676872000	-2.753713000	1.447353000
6	-3.632796000	-3.388315000	3.496472000
8	-3.333233000	-4.284540000	4.259998000
8	-4.605817000	-2.518443000	3.826013000
6	-3.925279000	-4.414938000	-0.915306000
1	-4.466160000	-4.121197000	-1.820305000
1	-3.929852000	-5.509478000	-0.865932000
1	-4.484474000	-4.033459000	-0.057853000
6	-1.774908000	-4.401014000	-2.255196000
1	-1.884517000	-5.490682000	-2.303332000
1	-0.706662000	-4.174141000	-2.194251000
6	-2.282213000	-3.725583000	-3.522474000
8	-1.879181000	-2.617257000	-3.869295000
7	-3.230094000	-4.405869000	-4.224537000
1	-3.585709000	-4.001482000	-5.080529000
1	-3.521779000	-5.340465000	-3.980342000
1	9.114071000	-0.537865000	0.482671000
1	-4.785291000	-1.871500000	3.105056000
1	-1.617246000	3.837734000	0.139765000
6	3.638518000	-4.104370000	0.526742000
1	3.239405000	-4.446674000	-0.433983000

1	3.222526000	-4.746343000	1.309186000
6	5.154661000	-4.268712000	0.580555000
8	5.785584000	-4.207274000	1.635033000
7	5.773793000	-4.423653000	-0.622558000
1	6.774578000	-4.572631000	-0.630411000
1	5.263577000	-4.599961000	-1.475466000
1	1.006037000	-4.269109000	1.174716000
6	3.518696000	3.652629000	-1.317716000
1	3.799064000	3.398334000	-2.344763000
1	3.435039000	4.743119000	-1.262677000
6	0.877267000	5.132021000	-0.290696000
1	-0.175301000	5.428313000	-0.334850000
1	1.385342000	5.719904000	-1.063257000
6	4.650065000	3.240807000	-0.382862000
8	4.634093000	3.386459000	0.824623000
8	5.662239000	2.670761000	-1.057732000
1	6.340019000	2.295153000	-0.437147000
6	1.457533000	5.510000000	1.083188000
1	0.916450000	4.997619000	1.888135000
1	2.499033000	5.173213000	1.169124000
6	1.407839000	6.996574000	1.346564000
8	1.007469000	7.842374000	0.571619000
8	1.877023000	7.297843000	2.578985000
1	1.821931000	8.267938000	2.677525000
6	-4.353133000	3.296156000	0.303663000
1	-3.787252000	3.843677000	1.065009000
1	-5.332403000	3.092476000	0.749853000
6	-5.664226000	0.372119000	-0.317634000
1	-6.318835000	1.222285000	-0.089207000
1	-6.038607000	-0.061159000	-1.251517000
6	-4.544987000	4.200424000	-0.924907000
1	-5.130972000	3.688128000	-1.698139000
1	-3.582197000	4.438762000	-1.392768000
6	-5.243254000	5.498373000	-0.587710000
8	-5.660210000	5.815954000	0.507761000
8	-5.360314000	6.293676000	-1.675489000
1	-5.817859000	7.107541000	-1.388685000
6	-5.860790000	-0.645783000	0.796932000
8	-5.204004000	-0.674911000	1.825924000
8	-6.842535000	-1.519598000	0.531403000
1	-6.932689000	-2.120303000	1.297285000
1	-4.292614000	-1.802767000	-1.348387000

NiI-B

NiI-B-opt.gjf.log

Temperature 298.150 Kelvin. Pressure 1.00000 Atm.

Zero-point correction= 0.865192 (Hartree/Particle)
Thermal correction to Energy= 0.926020
Thermal correction to Enthalpy= 0.926964
Thermal correction to Gibbs Free Energy= 0.760345
Sum of electronic and zero-point Energies= -3178.778640
Sum of electronic and thermal Energies= -3178.717812
Sum of electronic and thermal Enthalpies= -3178.716868
Sum of electronic and thermal Free Energies= -3178.883487

6	-4.440021000	0.976920000	1.888113000
6	-0.401488000	-4.919696000	1.077413000
6	7.412895000	0.050540000	0.485257000
6	-0.319603000	5.962691000	0.598321000
7	-2.005157000	0.638388000	-0.267022000
6	-5.841557000	1.421734000	2.312055000
6	1.087758000	-5.260769000	1.133823000
7	-0.789416000	-1.999530000	-0.452183000
8	8.228770000	0.140112000	-0.411614000
8	-1.472880000	6.058925000	0.967321000
7	0.546698000	1.874023000	-0.804704000
8	-7.208810000	2.024734000	4.151629000
8	2.807015000	-6.298200000	2.450328000
8	-5.164094000	1.216823000	4.642326000
8	0.748725000	-6.158326000	3.337152000
6	-1.628908000	3.018532000	-0.667048000
6	-3.081246000	-1.503436000	0.205330000
6	1.274445000	-3.136194000	-1.099673000
6	2.952637000	1.424959000	-1.048453000
28	-0.124266000	-0.056295000	-0.657217000
6	-2.441102000	1.938412000	-0.464191000
6	-2.051853000	-2.368094000	-0.146068000
6	2.147671000	-2.020698000	-1.184988000
6	1.863306000	2.289105000	-0.923948000
6	-3.974613000	1.969394000	-0.512856000
6	-2.277801000	-3.864728000	-0.391640000
6	3.587196000	-2.117772000	-1.285198000
6	1.932384000	3.730326000	-0.939507000
6	-4.296177000	0.727357000	0.369416000
6	-0.812944000	-4.387360000	-0.323104000
6	4.073517000	-0.829457000	-1.231077000
6	0.627962000	4.175092000	-0.851237000
6	-3.056486000	-0.114703000	0.104139000
6	-0.035711000	-3.126594000	-0.694452000
6	2.920190000	0.033813000	-1.111437000

6	-0.209576000	3.001981000	-0.761738000
6	-4.352478000	1.639973000	-2.001941000
6	4.370144000	-3.402326000	-1.263140000
6	3.189108000	4.549817000	-0.962113000
7	-5.195633000	-3.815795000	-1.978491000
6	-5.811196000	1.244655000	-2.198528000
6	-3.997225000	-3.243411000	-2.262435000
6	4.302288000	-4.061907000	0.112169000
6	3.791107000	4.691979000	0.445447000
8	-6.744607000	2.034375000	-2.026714000
8	-3.918792000	-2.132466000	-2.797315000
8	4.483064000	-3.480812000	1.167139000
6	-2.760423000	-4.045287000	-1.878648000
6	5.508243000	-0.400985000	-1.139349000
7	-6.013787000	-0.047292000	-2.568863000
8	3.994273000	-5.371564000	0.045449000
6	-4.601768000	3.299351000	-0.090613000
6	-3.256700000	-4.536634000	0.577311000
6	5.954045000	-0.300138000	0.329281000
6	0.138922000	5.599295000	-0.800434000
6	-5.993405000	1.529150000	3.811808000
6	1.634466000	-5.954359000	2.351627000
7	1.758874000	-0.725222000	-1.071866000
8	7.741728000	0.255569000	1.784372000
8	0.728067000	6.145115000	1.440422000
1	-4.207960000	0.043365000	2.412728000
1	-3.691452000	1.704843000	2.222342000
1	-0.994620000	-5.809896000	1.312203000
1	-0.631102000	-4.168688000	1.842055000
1	-6.126596000	2.384159000	1.877763000
1	-6.596610000	0.706141000	1.957787000
1	1.382220000	-5.882924000	0.280718000
1	1.681191000	-4.346869000	1.038366000
1	-2.104072000	3.990228000	-0.745786000
1	-4.041073000	-1.943877000	0.454938000
1	1.724888000	-4.109059000	-1.268410000
1	-5.195669000	0.205440000	0.024029000
1	-0.643343000	-5.192583000	-1.049201000
1	-3.697105000	0.843449000	-2.366847000
1	-4.162047000	2.531795000	-2.608364000
1	4.022708000	-4.117658000	-2.015531000
1	5.428643000	-3.203782000	-1.470970000
1	2.993618000	5.551275000	-1.361984000
1	3.937098000	4.102279000	-1.627065000
1	3.063036000	5.159698000	1.119877000
1	-1.961037000	-3.709551000	-2.544667000

1	-2.928148000	-5.114813000	-2.058550000
1	5.667269000	0.564938000	-1.632806000
1	6.160892000	-1.113391000	-1.656584000
1	-4.304711000	3.581293000	0.924173000
1	-5.690809000	3.250301000	-0.159217000
1	-4.275798000	4.101573000	-0.761889000
1	-3.305302000	-5.616625000	0.391446000
1	-2.961648000	-4.381216000	1.618176000
1	-4.266589000	-4.132989000	0.464678000
1	5.356006000	0.447111000	0.864990000
1	5.766909000	-1.252819000	0.842367000
1	-0.712532000	5.759083000	-1.468918000
1	0.940025000	6.285624000	-1.094086000
1	-7.237599000	2.057628000	5.127095000
1	1.224101000	-6.588030000	4.075331000
1	8.695842000	0.462352000	1.804612000
1	3.820834000	-5.704507000	0.962995000
1	0.355767000	6.315046000	2.326839000
1	-6.969954000	-0.351689000	-2.696897000
1	-5.264380000	-0.731985000	-2.681089000
1	-6.047915000	-3.313604000	-2.186686000
1	3.932832000	1.889459000	-1.120364000
1	4.011710000	3.707482000	0.875695000
6	5.054018000	5.517837000	0.469321000
8	5.542288000	6.108165000	-0.474194000
8	5.610402000	5.543561000	1.705439000
1	6.411428000	6.098577000	1.642915000
1	-5.271898000	-4.730607000	-1.560227000

d) Ni^{II}-SCoM, Ni^{II}, Ni^{I} and for **C**.

NiII-SCoM-C
NiII-SCoM-C-opt.gjf.log

Temperature 298.150 Kelvin. Pressure 1.00000 Atm.

Zero-point correction= 1.023474 (Hartree/Particle)
Thermal correction to Energy= 1.094049
Thermal correction to Enthalpy= 1.094993
Thermal correction to Gibbs Free Energy= 0.906095
Sum of electronic and zero-point Energies= -4283.538336
Sum of electronic and thermal Energies= -4283.467762
Sum of electronic and thermal Enthalpies= -4283.466818
Sum of electronic and thermal Free Energies= -4283.655716

6	-4.622631000	2.147588000	1.637140000

6	-1.123985000	-3.926553000	2.880299000
6	6.729429000	-2.227057000	2.464836000
6	2.380218000	6.573653000	0.802545000
7	-1.959114000	1.414912000	0.183554000
6	-6.008577000	2.772689000	1.809160000
6	0.245277000	-4.590135000	3.124492000
7	-1.142215000	-1.495124000	0.589036000
8	6.364001000	-1.741908000	3.517355000
8	3.233579000	6.680124000	1.665638000
7	0.894509000	2.094433000	0.065820000
8	-7.533563000	3.702752000	3.366373000
8	0.609321000	-5.961818000	5.099640000
8	-5.559038000	2.994564000	4.189992000
8	0.511917000	-3.721294000	5.314105000
6	-1.048138000	3.575277000	-0.511286000
6	-3.379114000	-0.574924000	0.928473000
6	0.794457000	-2.971757000	0.421097000
6	3.151126000	1.205262000	-0.137567000
28	-0.135787000	0.294942000	-0.063036000
6	-2.161584000	2.603278000	-0.260603000
6	-2.494842000	-1.586909000	0.959478000
6	1.783342000	-2.078317000	0.083737000
6	2.193048000	2.250366000	0.005663000
6	-3.620914000	2.847510000	-0.679996000
6	-2.872587000	-3.053834000	1.164708000
6	3.174993000	-2.501904000	-0.351211000
6	2.626430000	3.701176000	0.189718000
6	-4.311984000	1.741680000	0.182841000
6	-1.471092000	-3.705440000	1.388867000
6	3.990248000	-1.205886000	-0.118492000
6	1.283446000	4.458715000	0.046629000
6	-3.223606000	0.657393000	0.095862000
6	-0.550400000	-2.669651000	0.753352000
6	2.892277000	-0.146325000	-0.090267000
6	0.219311000	3.372941000	0.328318000
6	-3.679783000	2.469103000	-2.211365000
6	3.154909000	-2.929304000	-1.829560000
6	3.744422000	4.196644000	-0.771299000
7	-5.754728000	-2.901368000	-0.502082000
6	-5.094688000	2.133579000	-2.669321000
6	-4.463082000	-2.717513000	-0.890086000
6	4.455212000	-3.563510000	-2.258042000
6	5.042951000	4.649030000	-0.060887000
8	-5.941885000	3.010171000	-2.854361000
8	-4.165953000	-1.861408000	-1.730664000
8	5.349215000	-3.935083000	-1.519223000

6	-3.418833000	-3.595964000	-0.211799000
6	4.782696000	-1.216327000	1.204419000
7	-5.366727000	0.812448000	-2.852017000
8	4.520929000	-3.702673000	-3.598930000
6	-4.125145000	4.279992000	-0.484938000
6	-3.904840000	-3.284470000	2.273415000
6	6.018855000	-2.114035000	1.138013000
6	1.143014000	5.714992000	0.934010000
6	-6.303173000	3.150249000	3.242825000
6	0.475456000	-4.867181000	4.590374000
7	1.667039000	-0.719844000	0.066238000
8	7.862910000	-2.961389000	2.359126000
8	2.498861000	7.120491000	-0.421414000
6	-0.034770000	-1.569792000	-2.816095000
8	0.716781000	-4.336808000	-3.736901000
16	-0.534299000	0.183432000	-2.501975000
6	-1.138708000	-2.458910000	-3.377858000
8	-1.689325000	-4.954901000	-4.067179000
16	-0.689383000	-4.223217000	-3.234151000
8	-0.796386000	-4.543130000	-1.770168000
1	-4.573118000	1.257801000	2.273909000
1	-3.856260000	2.826588000	2.026493000
1	-1.896606000	-4.566703000	3.319847000
1	-1.159351000	-2.969828000	3.413813000
1	-6.142539000	3.669894000	1.195814000
1	-6.791862000	2.076655000	1.480795000
1	0.318540000	-5.541261000	2.590341000
1	1.047105000	-3.933987000	2.771156000
1	-0.788434000	3.503413000	-1.578404000
1	-1.412235000	4.596137000	-0.350103000
1	-4.404906000	-0.804486000	1.201686000
1	1.050834000	-4.023301000	0.387825000
1	3.540561000	-3.338248000	0.250361000
1	2.981311000	3.791806000	1.227328000
1	-5.232498000	1.384016000	-0.287633000
1	-1.383678000	-4.657527000	0.854669000
1	4.683952000	-1.008472000	-0.944393000
1	1.184202000	4.768636000	-1.001167000
1	-3.211676000	0.260417000	-0.930095000
1	-0.070988000	3.387574000	1.390380000
1	-2.994078000	1.645619000	-2.435390000
1	-3.346120000	3.334364000	-2.791695000
1	2.347657000	-3.641311000	-2.048832000
1	2.950239000	-2.071076000	-2.478934000
1	3.353562000	5.035282000	-1.350607000
1	3.987926000	3.416434000	-1.499503000

1	4.798122000	5.057473000	0.925659000
1	-2.584746000	-3.699539000	-0.909916000
1	-3.821378000	-4.602134000	-0.038090000
1	4.125469000	-1.547478000	2.017854000
1	5.090085000	-0.194594000	1.453493000
1	-4.059660000	4.602135000	0.558779000
1	-5.161816000	4.354698000	-0.823683000
1	-3.538471000	4.980901000	-1.088445000
1	-4.087116000	-4.355671000	2.417751000
1	-3.583034000	-2.855107000	3.226171000
1	-4.860147000	-2.820532000	2.010775000
1	5.768226000	-3.124007000	0.794387000
1	6.736865000	-1.737551000	0.398178000
1	1.046332000	5.433573000	1.985996000
1	0.253614000	6.281045000	0.635639000
1	0.288086000	-1.989182000	-1.866525000
1	0.824625000	-1.591481000	-3.490953000
1	-2.077885000	-2.330477000	-2.833051000
1	-1.321357000	-2.260958000	-4.439193000
1	-7.654998000	3.918922000	4.310894000
1	0.651514000	-3.977170000	6.245974000
1	8.259641000	-3.000258000	3.250491000
1	5.363606000	-4.153226000	-3.800155000
1	3.453899000	7.336691000	-0.563483000
1	-6.322973000	0.568912000	-3.076239000
1	-4.759298000	0.050931000	-2.560665000
1	-6.479371000	-2.393649000	-0.992744000
1	4.191987000	1.501500000	-0.218887000
1	5.739283000	3.819300000	0.080640000
6	5.724851000	5.781079000	-0.792474000
8	5.195941000	6.845378000	-1.086266000
8	7.008909000	5.527695000	-1.088698000
1	7.370521000	6.318920000	-1.535105000
1	-6.032250000	-3.695547000	0.055823000

NiII-C

NiII-C-opt.gjf.log

Temperature 298.150 Kelvin. Pressure 1.00000 Atm.

Zero-point correction= 0.951884 (Hartree/Particle)
Thermal correction to Energy= 1.013469
Thermal correction to Enthalpy= 1.014413
Thermal correction to Gibbs Free Energy= 0.846797
Sum of electronic and zero-point Energies= -3182.648424
Sum of electronic and thermal Energies= -3182.586838

Sum of electronic and thermal Enthalpies= -3182.585894
Sum of electronic and thermal Free Energies= -3182.753510

28	0.156874000	-0.126407000	0.364126000
7	-0.967190000	1.446804000	0.336753000
7	-1.367636000	-1.312524000	0.535851000
7	1.406944000	-1.593946000	0.903308000
7	1.616453000	0.873048000	-0.293071000
6	-0.548592000	2.735723000	0.093035000
6	-1.707556000	3.712288000	0.177820000
6	-2.298351000	1.454361000	0.649613000
6	-2.795885000	2.866678000	0.873705000
6	2.816978000	0.137969000	-0.717020000
6	1.736000000	2.156269000	-0.529243000
6	3.086268000	2.522432000	-1.120316000
6	3.663283000	1.140979000	-1.532567000
6	0.715181000	3.102109000	-0.281545000
6	2.675829000	-1.499725000	1.129610000
6	0.900463000	-2.892669000	1.446588000
6	2.181233000	-3.755640000	1.567928000
6	3.228505000	-2.662307000	1.955013000
6	3.523613000	-0.444161000	0.499888000
6	-1.276805000	-2.708713000	0.278749000
6	-2.651467000	-0.965699000	0.556735000
6	-2.581039000	-3.167712000	-0.356652000
6	-3.562064000	-2.164503000	0.340084000
6	-0.229858000	-3.454775000	0.642155000
6	-3.109213000	0.350256000	0.737650000
1	-3.786037000	-2.578543000	1.335680000
6	-4.889677000	-1.862248000	-0.382196000
1	-5.160508000	-2.701574000	-1.025620000
1	-4.772497000	-1.000761000	-1.047245000
6	-6.048043000	-1.621962000	0.588632000
1	-6.232264000	-2.523053000	1.187540000
1	-5.837123000	-0.824238000	1.311195000
6	-7.333314000	-1.270543000	-0.126941000
8	-7.472455000	-1.184707000	-1.329652000
8	-8.341701000	-1.057845000	0.748062000
1	-4.167120000	0.516650000	0.892565000
6	-2.499729000	-2.934809000	-1.881993000
1	-1.677774000	-3.527670000	-2.296509000
1	-3.421615000	-3.244070000	-2.376736000
1	-2.307730000	-1.879638000	-2.105714000
1	0.510967000	-2.671355000	2.450575000
1	2.069979000	-4.475857000	2.385735000
6	2.539018000	-4.552268000	0.295271000

1	3.469270000	-5.098894000	0.483888000
1	1.782442000	-5.324407000	0.130549000
6	2.687668000	-3.747279000	-1.005812000
1	1.747851000	-3.234076000	-1.250398000
1	3.445187000	-2.964402000	-0.922515000
6	3.028067000	-4.643303000	-2.191050000
8	2.707560000	-5.812639000	-2.253025000
8	3.687962000	-4.066605000	-3.211042000
6	4.696154000	-3.019288000	1.679860000
1	5.363501000	-2.227772000	2.035254000
1	4.955170000	-3.937227000	2.217305000
1	4.907318000	-3.184063000	0.621457000
6	3.081866000	-2.343147000	3.483047000
1	3.487159000	-3.195307000	4.037943000
1	2.027693000	-2.247046000	3.754984000
6	3.734539000	-1.027176000	3.888466000
8	3.271122000	0.050081000	3.506658000
7	4.842397000	-1.112600000	4.661538000
1	5.283392000	-0.259142000	4.979487000
1	5.194591000	-1.990533000	5.013939000
1	3.763031000	0.326381000	1.239537000
1	4.468607000	-0.896299000	0.184029000
1	2.485124000	-0.693455000	-1.339670000
1	4.718121000	1.053022000	-1.259265000
6	3.531616000	0.888848000	-3.040565000
1	4.121670000	1.621829000	-3.605661000
1	2.487632000	1.023455000	-3.359336000
6	3.955684000	-0.507335000	-3.467055000
8	4.336493000	-1.374798000	-2.694679000
8	3.870553000	-0.795811000	-4.771114000
1	2.936115000	3.171052000	-1.992816000
1	-3.780290000	3.006137000	0.419573000
1	-1.424662000	4.586096000	0.773854000
6	-2.109804000	4.180450000	-1.238195000
1	-2.480565000	3.321278000	-1.811138000
1	-1.218605000	4.537040000	-1.764458000
6	-3.160600000	5.290462000	-1.229914000
1	-4.069844000	4.995849000	-0.696572000
1	-2.794607000	6.173955000	-0.692488000
6	-3.542965000	5.715168000	-2.629140000
8	-3.081430000	5.264737000	-3.658848000
8	-4.485103000	6.683968000	-2.610120000
6	-2.910304000	3.135590000	2.384118000
1	-3.545696000	2.381627000	2.864053000
1	-1.930439000	3.062263000	2.871598000
6	-3.500898000	4.496815000	2.685396000

8	-3.928733000	5.278937000	1.860404000
8	-3.507672000	4.741831000	4.010010000
1	-9.137725000	-0.842121000	0.224669000
1	-3.910072000	5.622035000	4.144706000
1	-4.683922000	6.912159000	-3.538770000
1	3.549659000	-0.035083000	-5.287815000
1	3.935683000	-3.130888000	-3.024705000
1	0.917471000	4.147664000	-0.482107000
6	3.945313000	3.286004000	-0.090903000
1	3.368360000	4.123821000	0.315008000
1	4.175181000	2.633369000	0.758664000
6	5.242807000	3.821743000	-0.696234000
1	5.854032000	3.019772000	-1.129098000
1	5.037559000	4.517717000	-1.519432000
6	6.095425000	4.541397000	0.326635000
8	5.831757000	4.662607000	1.505270000
8	7.215945000	5.043835000	-0.235270000
1	7.720921000	5.491057000	0.471287000
6	-2.896781000	-4.645437000	-0.025059000
1	-2.700167000	-4.822308000	1.037531000
1	-2.226729000	-5.293558000	-0.599657000
6	-4.323652000	-5.046352000	-0.388814000
8	-4.699652000	-5.157568000	-1.553791000
7	-5.173036000	-5.208972000	0.665011000
1	-6.108533000	-5.543414000	0.471136000
1	-4.841248000	-5.280646000	1.615932000
1	-0.236574000	-4.519177000	0.441192000

NiI-C
NiI-C-opt.gjf.log

Temperature 298.150 Kelvin. Pressure 1.00000 Atm.

Zero-point correction= 0.948754 (Hartree/Particle)
Thermal correction to Energy= 1.011288
Thermal correction to Enthalpy= 1.012232
Thermal correction to Gibbs Free Energy= 0.837793
Sum of electronic and zero-point Energies= -3182.767516
Sum of electronic and thermal Energies= -3182.704982
Sum of electronic and thermal Enthalpies= -3182.704038
Sum of electronic and thermal Free Energies= -3182.878477

6	-4.447103000	0.771047000	1.324081000
6	-1.468392000	-4.339402000	1.636295000
6	6.659595000	-2.402424000	1.972885000
6	1.871936000	5.976140000	1.273618000

7	-2.117894000	0.812319000	-0.836244000
6	-5.687274000	1.289828000	2.054200000
6	-0.116402000	-4.773437000	2.232646000
7	-1.150926000	-1.974399000	-0.550586000
8	6.236967000	-1.929278000	3.009064000
8	2.656512000	5.967483000	2.205371000
7	0.687114000	1.608370000	-0.313418000
8	-6.797399000	1.362421000	4.145581000
8	0.013645000	-5.903778000	4.380853000
8	-4.972080000	0.046204000	4.020359000
8	-0.472392000	-3.704071000	4.317703000
6	-1.238857000	3.089125000	-0.827432000
6	-3.519494000	-1.305827000	-0.571033000
6	0.852586000	-3.379310000	-0.569646000
6	3.010789000	0.906376000	-0.501328000
28	-0.234140000	-0.164131000	-0.648079000
6	-2.337950000	2.076906000	-0.723862000
6	-2.540654000	-2.217063000	-0.487806000
6	1.821815000	-2.415403000	-0.769417000
6	1.978861000	1.851284000	-0.255899000
6	-3.813901000	2.470512000	-0.574979000
6	-2.800826000	-3.729145000	-0.493797000
6	3.273358000	-2.754352000	-1.069569000
6	2.322406000	3.262477000	0.209515000
6	-4.418209000	1.084004000	-0.184954000
6	-1.461185000	-4.242079000	0.093026000
6	3.991739000	-1.432726000	-0.713649000
6	0.959036000	3.975889000	0.088503000
6	-3.437200000	0.137840000	-0.928021000
6	-0.518426000	-3.140460000	-0.370933000
6	2.829911000	-0.442571000	-0.672237000
6	-0.054660000	2.812774000	0.099172000
6	-4.299789000	2.927896000	-1.994263000
6	3.414332000	-3.148726000	-2.548676000
6	3.481426000	3.966520000	-0.552759000
7	-3.238282000	-2.484066000	-3.666928000
6	-5.798923000	3.208198000	-2.051485000
6	-3.825095000	-3.401492000	-2.839538000
6	4.801095000	-3.637082000	-2.904898000
6	4.696198000	4.337080000	0.330580000
8	-6.299561000	4.207805000	-1.540054000
8	-5.043553000	-3.564480000	-2.802186000
8	5.654682000	-3.993358000	-2.116780000
6	-2.859382000	-4.212311000	-1.986684000
6	4.713206000	-1.482799000	0.649335000
7	-6.550631000	2.262845000	-2.679390000

8	4.985834000	-3.664668000	-4.242351000
6	-4.024327000	3.606886000	0.438478000
6	-4.069571000	-4.168837000	0.241850000
6	5.996654000	-2.312608000	0.619102000
6	0.680239000	5.053233000	1.159554000
6	-5.745928000	0.817538000	3.490242000
6	-0.173902000	-4.891104000	3.737128000
7	1.619017000	-1.077014000	-0.698644000
8	7.820321000	-3.097173000	1.910409000
8	2.043298000	6.732687000	0.173415000
1	-4.421034000	-0.315983000	1.449561000
1	-3.538097000	1.139104000	1.813136000
1	-2.233636000	-5.063504000	1.937265000
1	-1.756448000	-3.370945000	2.060048000
1	-5.756484000	2.381263000	2.061070000
1	-6.602771000	0.940210000	1.557528000
1	0.191640000	-5.745053000	1.836587000
1	0.655635000	-4.039823000	1.975640000
1	-0.867752000	3.096580000	-1.861574000
1	-1.626190000	4.089871000	-0.618744000
1	-4.539473000	-1.680719000	-0.572924000
1	1.179235000	-4.414033000	-0.535807000
1	3.619104000	-3.590814000	-0.455022000
1	2.593293000	3.184826000	1.273477000
1	-5.429073000	0.964968000	-0.589780000
1	-1.182994000	-5.219074000	-0.320400000
1	4.715479000	-1.140928000	-1.484935000
1	0.917610000	4.457258000	-0.896990000
1	-3.690755000	0.170541000	-2.003504000
1	-0.434979000	2.645053000	1.118423000
1	-4.015975000	2.183857000	-2.746072000
1	-3.789027000	3.861343000	-2.251574000
1	2.715793000	-3.959655000	-2.793811000
1	3.150053000	-2.311163000	-3.204386000
1	3.091119000	4.879182000	-1.007141000
1	3.821577000	3.339636000	-1.383455000
1	4.356682000	4.548115000	1.350723000
1	-1.854356000	-4.173757000	-2.420096000
1	-3.190005000	-5.255802000	-2.001970000
1	4.027296000	-1.886118000	1.404711000
1	4.953469000	-0.463608000	0.969749000
1	-3.584699000	3.365251000	1.410408000
1	-5.087754000	3.814224000	0.565015000
1	-3.567362000	4.535778000	0.083919000
1	-4.087003000	-5.259750000	0.348386000
1	-4.135315000	-3.719814000	1.236893000

1	-4.957714000	-3.884107000	-0.328166000
1	5.817179000	-3.330114000	0.255931000
1	6.721937000	-1.887616000	-0.086675000
1	0.525551000	4.590549000	2.137818000
1	-0.217584000	5.620807000	0.890417000
1	-6.781354000	1.009643000	5.056105000
1	-0.503811000	-3.855476000	5.281833000
1	8.187799000	-3.119685000	2.814905000
1	5.880572000	-4.022442000	-4.403575000
1	2.991427000	7.013402000	0.147333000
1	-7.548736000	2.408272000	-2.754288000
1	-6.148599000	1.469928000	-3.156557000
1	-3.850412000	-1.809771000	-4.109215000
1	4.033193000	1.266223000	-0.446626000
1	5.426769000	3.526326000	0.381177000
6	5.366780000	5.612096000	-0.125071000
8	4.796996000	6.681159000	-0.300326000
8	6.689922000	5.480756000	-0.306177000
1	7.041230000	6.355838000	-0.564974000
1	-2.289714000	-2.179648000	-3.489769000

e) Ni^{II}-SCoM, Ni^{II}, Ni^{I} and for **D**.

NiII-SCoM-D
NiII-SCoM-D-opt.gjf.log

Temperature 298.150 Kelvin. Pressure 1.00000 Atm.

Zero-point correction= 1.025758 (Hartree/Particle)
Thermal correction to Energy= 1.095258
Thermal correction to Enthalpy= 1.096202
Thermal correction to Gibbs Free Energy= 0.908313
Sum of electronic and zero-point Energies= -4283.556785
Sum of electronic and thermal Energies= -4283.487286
Sum of electronic and thermal Enthalpies= -4283.486342
Sum of electronic and thermal Free Energies= -4283.674230

6	-4.856495000	0.396487000	1.646060000
6	-1.163273000	-4.060641000	2.482774000
6	6.401376000	0.534809000	3.671180000
6	0.083151000	6.836769000	0.129379000
7	-2.457975000	0.477672000	-0.509888000
6	-6.222662000	0.829380000	2.185950000
6	0.018618000	-4.049098000	3.471224000
7	-0.748568000	-1.975468000	-0.000511000
8	5.739476000	1.165351000	4.472029000

8	0.726748000	7.289553000	1.059440000
7	0.083320000	2.057061000	-0.138591000
8	-7.476795000	1.184342000	4.164202000
8	-0.261793000	-4.864616000	5.745577000
8	-5.489649000	0.149148000	4.405291000
8	-1.147238000	-2.874710000	5.167454000
6	-2.095348000	2.813061000	-1.088458000
6	-3.189214000	-1.782001000	0.344150000
6	1.402930000	-2.712052000	0.852437000
6	2.511125000	1.906160000	0.048032000
28	-0.302746000	0.018625000	-0.413183000
6	-2.954578000	1.644367000	-0.703324000
6	-2.053479000	-2.563615000	-0.355471000
6	2.150319000	-1.585605000	0.610737000
6	1.275245000	2.599568000	-0.069009000
6	-4.485209000	1.695881000	-0.620814000
6	-1.921079000	-4.106362000	-0.005480000
6	3.667498000	-1.581959000	0.685608000
6	1.239709000	4.125134000	-0.027287000
6	-4.770548000	0.332986000	0.109750000
6	-0.737883000	-4.121165000	0.996560000
6	4.003230000	-0.070185000	0.650030000
6	-0.224138000	4.419333000	-0.427678000
6	-3.562690000	-0.500552000	-0.389875000
6	0.014242000	-2.859312000	0.598476000
6	2.663987000	0.556529000	0.268008000
6	-0.953495000	3.098472000	-0.104965000
6	-4.999450000	1.660123000	-2.101496000
6	4.261568000	-2.383986000	-0.487125000
6	2.298452000	4.841258000	-0.913872000
7	-2.219621000	-2.547179000	-1.806419000
6	-6.500831000	1.404781000	-2.202255000
6	-1.897490000	-3.731491000	-2.423197000
6	5.716372000	-2.722880000	-0.270385000
6	3.285804000	5.740437000	-0.131960000
8	-7.337331000	2.198635000	-1.774330000
8	-1.893673000	-3.908867000	-3.635437000
8	6.266852000	-2.832421000	0.812293000
6	-1.499776000	-4.731131000	-1.358485000
6	4.508951000	0.484259000	1.997311000
7	-6.851156000	0.219260000	-2.769889000
8	6.369224000	-2.935919000	-1.429821000
6	-4.991893000	2.965662000	0.079845000
6	-3.211055000	-4.770517000	0.483514000
6	5.932018000	0.033873000	2.327185000
6	-0.856550000	5.658123000	0.243282000

6	-6.318810000	0.670940000	3.687383000
6	-0.452953000	-4.009275000	4.904931000
7	1.659225000	-0.361077000	0.270108000
8	7.690001000	0.189959000	3.906824000
8	0.221977000	7.262636000	-1.139395000
6	1.201007000	-1.484290000	-2.753763000
8	3.648052000	-3.309735000	-3.471143000
16	-0.064393000	-0.134169000	-2.832206000
6	1.173718000	-2.466340000	-3.915034000
8	2.083679000	-4.824338000	-4.702997000
16	2.269009000	-3.882601000	-3.560353000
8	1.787313000	-4.438183000	-2.249295000
1	-4.665716000	-0.601668000	2.048896000
1	-4.065843000	1.035455000	2.056847000
1	-1.787123000	-4.934640000	2.699828000
1	-1.781535000	-3.173782000	2.657489000
1	-6.476096000	1.864083000	1.943465000
1	-7.021654000	0.219281000	1.742278000
1	0.639065000	-4.940756000	3.348223000
1	0.645527000	-3.168729000	3.292962000
1	-1.652706000	2.598440000	-2.069866000
1	-2.705920000	3.713727000	-1.191447000
1	-2.881192000	-1.580001000	1.373946000
1	-4.093771000	-2.396624000	0.378477000
1	1.933051000	-3.601180000	1.175162000
1	4.001409000	-2.046778000	1.618635000
1	1.392813000	4.417957000	1.022218000
1	-5.696289000	-0.117823000	-0.264016000
1	-0.115098000	-5.011840000	0.853948000
1	4.747365000	0.155002000	-0.124841000
1	-0.252199000	4.564938000	-1.515021000
1	-3.790682000	-0.810284000	-1.417739000
1	-1.369254000	3.135811000	0.914288000
1	-4.441055000	0.914009000	-2.676316000
1	-4.800400000	2.633161000	-2.562870000
1	3.726210000	-3.332627000	-0.624916000
1	4.136391000	-1.875729000	-1.445680000
1	1.773029000	5.455727000	-1.647273000
1	2.865682000	4.104645000	-1.491635000
1	2.786305000	6.153265000	0.751291000
1	-0.409080000	-4.825463000	-1.459128000
1	-1.938909000	-5.715912000	-1.540896000
1	3.826075000	0.170420000	2.796510000
1	4.475336000	1.578661000	1.979054000
1	-4.582752000	3.060606000	1.090026000
1	-6.081421000	2.963820000	0.123691000

1	-4.700510000	3.858280000	-0.482811000
1	-3.028575000	-5.830905000	0.691021000
1	-3.612950000	-4.315792000	1.392674000
1	-3.983248000	-4.713071000	-0.292416000
1	6.025662000	-1.057958000	2.316816000
1	6.641128000	0.387222000	1.566997000
1	-1.026762000	5.473489000	1.307347000
1	-1.815486000	5.888853000	-0.233928000
1	1.015177000	-2.047330000	-1.840694000
1	2.194753000	-1.036883000	-2.669077000
1	0.174522000	-2.879298000	-4.067920000
1	1.521232000	-2.019856000	-4.852348000
1	-7.479383000	1.035698000	5.129438000
1	-1.430025000	-2.921022000	6.100803000
1	7.918917000	0.535929000	4.790759000
1	7.280337000	-3.201119000	-1.198197000
1	1.084069000	7.744442000	-1.202650000
1	-7.833594000	0.014523000	-2.896239000
1	-6.178400000	-0.402717000	-3.193655000
1	-1.954058000	-1.692069000	-2.307597000
1	3.408081000	2.515077000	0.088526000
1	4.162800000	5.183428000	0.205703000
6	3.713425000	6.945309000	-0.936681000
8	2.949851000	7.757908000	-1.442236000
8	5.045123000	7.071146000	-1.041154000
1	5.225613000	7.884283000	-1.553311000

NiII-D

NiII-D-opt.gjf.log

Temperature 298.150 Kelvin. Pressure 1.00000 Atm.

Zero-point correction= 0.954530 (Hartree/Particle)
Thermal correction to Energy= 1.014856
Thermal correction to Enthalpy= 1.015800
Thermal correction to Gibbs Free Energy= 0.851141
Sum of electronic and zero-point Energies= -3182.657729
Sum of electronic and thermal Energies= -3182.597403
Sum of electronic and thermal Enthalpies= -3182.596458
Sum of electronic and thermal Free Energies= -3182.761118

28	-0.039333000	-0.248308000	-0.282631000
7	1.328327000	1.136072000	-0.373248000
7	1.313972000	-1.684098000	-0.346114000
7	-1.571640000	-1.501340000	-0.637856000
7	-1.305393000	1.074132000	0.217337000

6	1.109739000	2.496544000	-0.397733000
6	2.410765000	3.276699000	-0.474270000
6	2.674568000	0.913245000	-0.449054000
6	3.432673000	2.173939000	-0.804898000
6	-2.616217000	0.648506000	0.733057000
6	-1.236468000	2.383586000	0.181115000
6	-2.492886000	3.066401000	0.690958000
6	-3.247816000	1.900799000	1.376582000
6	-0.097735000	3.108365000	-0.220571000
6	-2.811734000	-1.204528000	-0.867903000
6	-1.373238000	-2.949428000	-0.915210000
6	-2.796273000	-3.547738000	-0.859365000
6	-3.612693000	-2.365166000	-1.464955000
6	-3.433381000	0.068204000	-0.411126000
6	1.068925000	-3.144345000	-0.448187000
6	2.620031000	-1.517578000	-0.182584000
6	2.244668000	-3.834738000	0.312419000
6	3.383134000	-2.819343000	-0.003718000
6	-0.327838000	-3.535952000	0.009716000
6	3.295949000	-0.288612000	-0.263764000
1	-0.401348000	-4.627937000	-0.011301000
1	-0.490933000	-3.206740000	1.038212000
1	3.782197000	-3.066921000	-0.998127000
6	4.569431000	-2.799844000	0.980925000
1	4.660676000	-3.788481000	1.439923000
1	4.378678000	-2.102321000	1.803263000
6	5.920086000	-2.489905000	0.327605000
1	6.126633000	-3.200722000	-0.483906000
1	5.961726000	-1.498738000	-0.135140000
6	7.062314000	-2.584403000	1.316771000
8	6.971834000	-2.965497000	2.465705000
8	8.228401000	-2.193443000	0.758393000
1	4.376329000	-0.294354000	-0.253458000
6	1.978414000	-4.011392000	1.807001000
1	1.121663000	-4.669947000	1.976630000
1	2.837426000	-4.466522000	2.306318000
1	1.775442000	-3.049482000	2.291470000
6	2.455793000	-5.145000000	-0.461702000
7	1.319185000	-3.629271000	-1.813631000
6	2.045929000	-4.788925000	-1.892204000
8	2.306561000	-5.398346000	-2.917740000
1	-1.009742000	-3.044247000	-1.942132000
1	-2.848119000	-4.419795000	-1.520752000
6	-3.257231000	-4.016710000	0.536512000
1	-4.252567000	-4.462290000	0.431752000
1	-2.608636000	-4.836166000	0.860000000

6	-3.309719000	-2.978236000	1.668015000
1	-2.330752000	-2.513274000	1.836173000
1	-3.989832000	-2.152774000	1.443936000
6	-3.726310000	-3.613174000	2.992506000
8	-3.614721000	-4.799359000	3.225306000
8	-4.205897000	-2.779893000	3.930554000
6	-5.116512000	-2.369900000	-1.155442000
1	-5.624400000	-1.540716000	-1.658172000
1	-5.557582000	-3.301007000	-1.525590000
1	-5.339122000	-2.301522000	-0.089402000
6	-3.445531000	-2.373038000	-3.023778000
1	-4.022205000	-3.216827000	-3.415476000
1	-2.401068000	-2.535621000	-3.301632000
6	-3.841939000	-1.050695000	-3.669416000
8	-3.200697000	-0.022226000	-3.440737000
7	-4.924580000	-1.072091000	-4.480411000
1	-5.194088000	-0.222026000	-4.958672000
1	-5.421940000	-1.921948000	-4.702541000
1	-3.510801000	0.765281000	-1.250633000
1	-4.451030000	-0.138728000	-0.068030000
1	-2.448440000	-0.136937000	1.469196000
1	3.483902000	-5.515835000	-0.432990000
1	1.804969000	-5.946900000	-0.091288000
1	0.939103000	-3.193412000	-2.643192000
1	-4.319448000	1.933799000	1.164405000
6	-3.056579000	1.908892000	2.898306000
1	-3.462378000	2.832002000	3.331718000
1	-1.986273000	1.894647000	3.150167000
6	-3.707424000	0.723636000	3.589664000
8	-4.316891000	-0.156362000	2.999324000
8	-3.575841000	0.640387000	4.918327000
1	-2.207199000	3.838688000	1.416590000
1	4.343646000	2.260539000	-0.206951000
1	2.352104000	4.034219000	-1.263271000
6	2.683768000	3.978426000	0.875116000
1	2.823417000	3.218265000	1.654071000
1	1.800437000	4.554706000	1.166806000
6	3.893725000	4.911411000	0.839659000
1	4.810430000	4.391362000	0.545093000
1	3.762899000	5.696564000	0.084881000
6	4.129626000	5.577313000	2.175809000
8	3.456324000	5.422389000	3.175439000
8	5.206933000	6.392144000	2.136712000
6	3.824967000	2.120217000	-2.292125000
1	4.390164000	1.205747000	-2.508898000
1	2.934506000	2.085964000	-2.931583000

6	4.678907000	3.297938000	-2.711282000
8	5.127184000	4.145958000	-1.966159000
8	4.902052000	3.288023000	-4.039611000
1	8.923560000	-2.292139000	1.437560000
1	5.470495000	4.054781000	-4.248828000
1	5.301732000	6.789421000	3.023933000
1	-3.070778000	1.391341000	5.278687000
1	-4.282688000	-1.850141000	3.609116000
1	-0.145038000	4.190671000	-0.224684000
6	-3.264687000	3.751815000	-0.455462000
1	-2.581223000	4.397726000	-1.016999000
1	-3.629183000	3.003139000	-1.166591000
6	-4.437019000	4.590606000	0.053814000
1	-5.150545000	3.988196000	0.630074000
1	-4.093278000	5.378991000	0.735334000
6	-5.202739000	5.246014000	-1.075447000
8	-4.968238000	5.110493000	-2.258682000
8	-6.206591000	6.016576000	-0.604367000
1	-6.661238000	6.407027000	-1.375764000

NiI-D

NiI-D-opt.gjf.log

Temperature 298.150 Kelvin. Pressure 1.00000 Atm.

Zero-point correction= 0.951577 (Hartree/Particle)
Thermal correction to Energy= 1.012685
Thermal correction to Enthalpy= 1.013629
Thermal correction to Gibbs Free Energy= 0.844487
Sum of electronic and zero-point Energies= -3182.786635
Sum of electronic and thermal Energies= -3182.725527
Sum of electronic and thermal Enthalpies= -3182.724582
Sum of electronic and thermal Free Energies= -3182.893724

6	-3.807211000	0.836761000	1.804904000
6	-2.262194000	-4.502674000	0.508994000
6	5.719552000	-2.719952000	3.112165000
6	2.025963000	6.094356000	0.523281000
7	-2.230100000	0.903120000	-1.023870000
6	-4.677355000	1.480062000	2.888943000
6	-1.137472000	-5.133782000	1.350216000
7	-1.206578000	-1.877712000	-1.169131000
8	5.084928000	-2.216184000	4.017547000
8	2.702602000	6.229304000	1.527102000
7	0.680325000	1.577229000	-0.463327000
8	-5.089970000	1.537831000	5.220472000

8	-1.605879000	-6.609644000	3.224404000
8	-3.818261000	-0.163290000	4.467217000
8	-2.045181000	-4.407227000	3.415256000
6	-1.188698000	3.080930000	-1.162490000
6	-3.455977000	-1.203316000	-0.427909000
6	0.616160000	-3.440527000	-0.790962000
6	2.962014000	0.727087000	-0.248952000
28	-0.251092000	-0.157970000	-0.880036000
6	-2.314133000	2.164513000	-0.798975000
6	-2.643227000	-1.932649000	-1.520572000
6	1.651520000	-2.540474000	-0.678214000
6	1.976867000	1.746102000	-0.272729000
6	-3.617792000	2.620064000	-0.135042000
6	-2.952151000	-3.464145000	-1.766979000
6	3.114139000	-2.962134000	-0.629979000
6	2.371580000	3.192065000	0.021936000
6	-4.174884000	1.235108000	0.364260000
6	-1.843109000	-4.158371000	-0.939767000
6	3.809210000	-1.673440000	-0.132929000
6	1.085292000	3.950707000	-0.368156000
6	-3.544567000	0.293479000	-0.696135000
6	-0.747619000	-3.099481000	-0.970189000
6	2.729056000	-0.622240000	-0.367021000
6	0.003205000	2.868490000	-0.223211000
6	-4.516461000	3.211197000	-1.275026000
6	3.574033000	-3.399016000	-2.029953000
6	3.654330000	3.692718000	-0.702348000
7	-2.847039000	-1.342542000	-2.841360000
6	-5.940919000	3.515120000	-0.817827000
6	-2.736996000	-2.225655000	-3.880061000
6	4.973100000	-3.973720000	-2.045318000
6	4.785719000	4.159110000	0.243904000
8	-6.199843000	4.412700000	-0.018545000
8	-2.675807000	-1.924889000	-5.065288000
8	5.595332000	-4.359831000	-1.075321000
6	-2.682969000	-3.624176000	-3.287178000
6	4.194735000	-1.723134000	1.360291000
7	-6.905338000	2.701310000	-1.328832000
8	5.464294000	-4.043672000	-3.301345000
6	-3.367234000	3.689424000	0.940668000
6	-4.389059000	-3.892157000	-1.459040000
6	5.399579000	-2.621399000	1.639761000
6	0.788741000	5.229888000	0.445110000
6	-4.459251000	0.844197000	4.245145000
6	-1.606109000	-5.496265000	2.739568000
7	1.516797000	-1.187715000	-0.621097000

8	6.830328000	-3.463853000	3.328772000
8	2.366495000	6.614588000	-0.670321000
1	-3.936232000	-0.243513000	1.909686000
1	-2.745193000	1.026372000	2.003055000
1	-3.102109000	-5.205476000	0.467852000
1	-2.622141000	-3.603472000	1.018840000
1	-4.517382000	2.555073000	2.995243000
1	-5.742684000	1.363094000	2.645240000
1	-0.758909000	-6.045334000	0.879694000
1	-0.304670000	-4.428359000	1.445494000
1	-0.861705000	2.871576000	-2.187060000
1	-1.509751000	4.125512000	-1.116037000
1	-2.966438000	-1.409780000	0.525860000
1	-4.478473000	-1.587252000	-0.373816000
1	0.862010000	-4.497320000	-0.764499000
1	3.259135000	-3.800392000	0.058138000
1	2.518295000	3.273631000	1.109948000
1	-5.266273000	1.205975000	0.273055000
1	-1.512799000	-5.085629000	-1.424269000
1	4.706033000	-1.441116000	-0.720712000
1	1.159549000	4.222387000	-1.429346000
1	-4.148125000	0.390259000	-1.609393000
1	-0.365749000	2.862248000	0.815021000
1	-4.523517000	2.533728000	-2.135341000
1	-4.075622000	4.155492000	-1.611521000
1	2.904541000	-4.173807000	-2.425871000
1	3.522149000	-2.563756000	-2.737855000
1	3.379896000	4.523907000	-1.354610000
1	4.042120000	2.909640000	-1.361560000
1	4.351281000	4.580692000	1.156980000
1	-1.679383000	-4.016979000	-3.489918000
1	-3.395596000	-4.287157000	-3.786269000
1	3.331443000	-2.066890000	1.943436000
1	4.414313000	-0.708377000	1.709365000
1	-2.689896000	3.327045000	1.719103000
1	-4.306929000	4.006218000	1.393810000
1	-2.915210000	4.581757000	0.495633000
1	-4.520589000	-4.954786000	-1.692330000
1	-4.666610000	-3.744205000	-0.412760000
1	-5.093431000	-3.324202000	-2.077907000
1	5.250930000	-3.635038000	1.252288000
1	6.293477000	-2.252088000	1.120971000
1	0.500538000	4.974926000	1.468420000
1	-0.030800000	5.785724000	-0.023783000
1	-4.924847000	1.067130000	6.060159000
1	-2.334796000	-4.711811000	4.296610000

1	6.971129000	-3.490502000	4.294756000
1	6.348309000	-4.455523000	-3.243568000
1	3.329982000	6.836698000	-0.635905000
1	-7.870242000	2.888120000	-1.089321000
1	-6.719842000	2.024374000	-2.054068000
1	-2.563777000	-0.381356000	-2.995637000
1	3.982060000	1.035266000	-0.041727000
1	5.440157000	3.333448000	0.533109000
6	5.601069000	5.282071000	-0.353542000
8	5.140946000	6.330573000	-0.786422000
8	6.920884000	5.039449000	-0.352178000
1	7.366667000	5.825879000	-0.725008000

f) Ni^{II}-SCoM, Ni^{II}, Ni^{I} and for **E**.

NiII-SCoM-E

NiII-SCoM-E.gjf.log

Temperature 298.150 Kelvin. Pressure 1.00000 Atm.

Zero-point correction= 0.999147 (Hartree/Particle)
Thermal correction to Energy= 1.066487
Thermal correction to Enthalpy= 1.067431
Thermal correction to Gibbs Free Energy= 0.884390
Sum of electronic and zero-point Energies= -4207.148799
Sum of electronic and thermal Energies= -4207.081459
Sum of electronic and thermal Enthalpies= -4207.080515
Sum of electronic and thermal Free Energies= -4207.263557

6	-4.348845000	-0.714704000	2.120408000
6	1.020408000	-3.581744000	2.733584000
6	5.535153000	2.928546000	3.553482000
6	-3.131614000	6.373205000	-1.173622000
7	-2.502281000	-0.392149000	-0.480740000
6	-5.698241000	-0.682781000	2.868262000
6	2.292655000	-3.046430000	3.392714000
7	0.068009000	-1.847474000	0.081140000
8	4.724057000	2.853841000	4.455885000
8	-2.835527000	7.353112000	-0.520261000
7	-0.823994000	2.134010000	-0.485299000
8	-5.145944000	1.159146000	4.250577000
8	1.280882000	-3.504195000	5.557969000
8	-5.751426000	-0.742202000	5.299890000
8	3.359558000	-2.638008000	5.465513000
6	-3.093389000	1.739675000	-1.451563000
6	-2.185998000	-2.487386000	0.862119000

6	2.421220000	-1.559912000	0.570853000
6	1.437484000	3.114683000	-0.311445000
28	-0.350837000	0.096811000	-0.569569000
6	-3.423772000	0.452525000	-0.763842000
6	-0.946482000	-2.920996000	0.043073000
6	2.599916000	-0.250717000	0.244090000
6	0.003643000	3.149260000	-0.464134000
6	-4.833954000	0.007484000	-0.361306000
6	-0.198782000	-4.239704000	0.522926000
6	3.964266000	0.386516000	0.142450000
6	-0.719329000	4.488343000	-0.581763000
6	-4.474922000	-1.147753000	0.648122000
6	1.088794000	-3.672839000	1.190440000
6	3.620774000	1.872379000	0.337497000
6	-2.103913000	4.070714000	-1.089401000
6	-3.132324000	-1.624972000	0.041836000
6	1.189197000	-2.287461000	0.576672000
6	2.145205000	1.915944000	-0.048974000
6	-2.213868000	2.630052000	-0.565801000
6	-5.501766000	-0.562141000	-1.660529000
6	4.593895000	0.117144000	-1.234875000
6	0.070765000	5.463137000	-1.448375000
7	-1.291197000	-3.191586000	-1.347427000
6	-6.789232000	-1.330907000	-1.374323000
6	-0.686244000	-4.301317000	-1.882009000
6	6.034448000	0.562936000	-1.287806000
6	1.427762000	5.675389000	-0.791585000
6	2.169116000	4.397028000	-0.388870000
8	-7.798854000	-0.787083000	-0.929381000
8	-0.774219000	-4.643523000	-3.055494000
8	6.740051000	0.820751000	-0.329012000
6	0.132436000	-4.970976000	-0.802103000
6	3.809088000	2.331280000	1.801166000
7	-6.732392000	-2.668541000	-1.613170000
8	6.490050000	0.626902000	-2.556677000
8	3.371698000	4.532616000	-0.134860000
6	-5.677347000	1.169412000	0.189214000
6	-1.038385000	-5.165533000	1.410221000
6	5.274383000	2.647466000	2.094856000
6	-3.269074000	4.967418000	-0.637965000
6	-5.550797000	-0.134889000	4.268317000
6	2.221448000	-3.103952000	4.901263000
7	1.608789000	0.679091000	-0.036938000
8	6.825301000	3.283924000	3.768535000
8	-3.336730000	6.417204000	-2.509874000
6	1.496182000	-1.058405000	-2.938917000

8	4.279020000	-2.317787000	-3.350156000
16	-0.190722000	-0.290765000	-2.956978000
6	1.657527000	-2.300321000	-3.808315000
8	3.209408000	-4.402241000	-4.232093000
16	3.117765000	-3.260143000	-3.278471000
8	2.817348000	-3.684389000	-1.867995000
1	-3.709077000	-1.429576000	2.647203000
1	-3.848207000	0.256148000	2.196935000
1	0.826141000	-4.580962000	3.134039000
1	0.170567000	-2.957236000	3.034537000
1	-6.425267000	-0.061216000	2.340034000
1	-6.110817000	-1.691859000	2.946086000
1	3.176150000	-3.615012000	3.074243000
1	2.493507000	-2.006824000	3.110840000
1	-2.544490000	1.522155000	-2.375092000
1	-4.004779000	2.279877000	-1.718977000
1	-1.834943000	-1.971609000	1.761088000
1	-2.753174000	-3.366285000	1.180039000
1	3.312852000	-2.137383000	0.784435000
1	4.638540000	0.008444000	0.915000000
1	-0.811741000	4.914495000	0.430977000
1	-5.208404000	-1.958804000	0.589857000
1	1.964738000	-4.271404000	0.917269000
1	4.205765000	2.522826000	-0.310298000
1	-2.083211000	4.038045000	-2.186930000
1	-3.377528000	-2.229307000	-0.840762000
1	-2.631193000	2.639371000	0.453768000
1	-4.790533000	-1.188784000	-2.208128000
1	-5.758593000	0.276924000	-2.315606000
1	4.562002000	-0.943671000	-1.516481000
1	4.036921000	0.630172000	-2.026154000
1	-0.458843000	6.415556000	-1.552441000
1	0.189332000	5.039109000	-2.454142000
1	1.302975000	6.260312000	0.131784000
1	2.112795000	6.246505000	-1.426017000
1	1.174332000	-4.820696000	-1.111376000
1	-0.062752000	-6.046752000	-0.763564000
1	3.449430000	1.545118000	2.476621000
1	3.206250000	3.220986000	1.994975000
1	-5.230672000	1.616266000	1.081865000
1	-6.686888000	0.827417000	0.419556000
1	-5.777954000	1.957586000	-0.563807000
1	-0.450265000	-6.048086000	1.685233000
1	-1.382210000	-4.691953000	2.333382000
1	-1.919336000	-5.517053000	0.860860000
1	5.935164000	1.831864000	1.776540000

1	5.591788000	3.522195000	1.513854000
1	-3.302493000	5.027426000	0.453156000
1	-4.216139000	4.546999000	-0.994997000
1	1.698569000	-1.348594000	-1.911127000
1	2.239499000	-0.309881000	-3.222431000
1	0.806157000	-2.977616000	-3.717135000
1	1.796895000	-2.055990000	-4.866227000
1	-5.055560000	1.445221000	5.179869000
1	3.244039000	-2.696382000	6.433359000
1	6.919030000	3.440242000	4.727751000
1	7.432337000	0.878981000	-2.510371000
1	-3.188966000	7.339436000	-2.795682000
1	-7.566166000	-3.222468000	-1.468057000
1	-5.934430000	-3.114415000	-2.041504000
1	-1.421255000	-2.392179000	-1.972470000

NiII-E
NiII-E.gjf.log

Temperature 298.150 Kelvin. Pressure 1.00000 Atm.

Zero-point correction= 0.926980 (Hartree/Particle)
Thermal correction to Energy= 0.985101
Thermal correction to Enthalpy= 0.986045
Thermal correction to Gibbs Free Energy= 0.826917
Sum of electronic and zero-point Energies= -3106.236734
Sum of electronic and thermal Energies= -3106.178614
Sum of electronic and thermal Enthalpies= -3106.177669
Sum of electronic and thermal Free Energies= -3106.336797

28	-0.340477000	0.056289000	0.360210000
7	1.569291000	-0.701765000	0.403834000
7	0.331404000	1.969549000	0.183028000
7	-2.324482000	0.660319000	0.658680000
7	-0.989453000	-1.813768000	0.100155000
6	1.941682000	-2.004347000	0.544329000
6	3.456473000	-2.144749000	0.537104000
6	2.684553000	0.097563000	0.354103000
6	3.933371000	-0.688413000	0.686572000
6	-2.393834000	-2.049081000	-0.272416000
6	-0.322795000	-2.928591000	0.171563000
6	-1.134569000	-4.141909000	-0.225746000
6	-2.537555000	-3.572461000	-0.541854000
6	1.053254000	-3.088811000	0.553764000
6	-3.331310000	-0.076972000	0.985907000
6	-2.722663000	2.088684000	0.683953000

6	-4.264786000	2.043643000	0.558282000
6	-4.570221000	0.742158000	1.375265000
6	-3.317252000	-1.567308000	0.851619000
6	-0.501195000	3.191643000	0.139846000
6	1.588660000	2.305842000	-0.035419000
6	0.309141000	4.222230000	-0.714704000
6	1.761481000	3.786854000	-0.338704000
6	-1.933104000	2.906416000	-0.330286000
6	2.705545000	1.438747000	0.080107000
1	-2.437251000	3.868343000	-0.472007000
1	-1.905997000	2.399950000	-1.298595000
1	2.002756000	4.252976000	0.628363000
6	2.872105000	4.176721000	-1.332918000
1	2.559587000	5.069911000	-1.881703000
1	3.011065000	3.394084000	-2.086124000
6	4.209652000	4.508604000	-0.663333000
1	4.087067000	5.333600000	0.051226000
1	4.617914000	3.677184000	-0.079070000
6	5.260321000	4.927869000	-1.669106000
8	5.075486000	5.084357000	-2.858570000
8	6.458241000	5.120645000	-1.076043000
1	3.684869000	1.890124000	-0.005668000
6	0.017844000	4.128889000	-2.212265000
1	-1.030317000	4.360649000	-2.422505000
1	0.624842000	4.843180000	-2.774363000
1	0.232535000	3.125773000	-2.598205000
6	-0.036737000	5.576014000	-0.076784000
7	-0.508222000	3.873647000	1.440148000
6	-0.308365000	5.229173000	1.388260000
8	-0.343559000	5.990609000	2.342072000
1	-2.484674000	2.481320000	1.677910000
1	-4.701357000	2.913636000	1.061386000
6	-4.810066000	2.035083000	-0.883343000
1	-5.904292000	2.034449000	-0.823486000
1	-4.543131000	2.975828000	-1.374873000
6	-4.367764000	0.872152000	-1.792272000
1	-3.299873000	0.945226000	-2.028109000
1	-4.505848000	-0.093337000	-1.308740000
6	-5.123230000	0.902127000	-3.115586000
8	-5.272675000	1.919819000	-3.759774000
8	-5.627801000	-0.263955000	-3.558226000
6	-5.923905000	0.074640000	1.087804000
1	-6.094642000	-0.766134000	1.767642000
1	-6.728650000	0.798885000	1.250948000
1	-6.015893000	-0.301558000	0.067551000
6	-4.522011000	1.088949000	2.903947000

1	-5.427908000	1.655023000	3.143265000
1	-3.667369000	1.732164000	3.127538000
6	-4.356036000	-0.136273000	3.794658000
8	-3.300195000	-0.773390000	3.799457000
7	-5.423249000	-0.484783000	4.551216000
1	-5.337821000	-1.268630000	5.185409000
1	-6.267396000	0.067540000	4.588621000
1	-3.022082000	-2.014373000	1.806798000
1	-4.331136000	-1.910503000	0.635119000
1	-2.602267000	-1.464218000	-1.171851000
1	0.757791000	6.322541000	-0.158694000
1	-0.944364000	6.011195000	-0.513395000
1	-0.713220000	3.410022000	2.315275000
1	-3.279775000	-3.999625000	0.140308000
6	-2.949723000	-3.898997000	-1.984028000
1	-3.033746000	-4.985219000	-2.115384000
1	-2.163773000	-3.569380000	-2.680037000
6	-4.231311000	-3.243525000	-2.463322000
8	-4.840308000	-2.377240000	-1.852249000
8	-4.681499000	-3.611127000	-3.669290000
1	-0.682392000	-4.561781000	-1.136178000
6	-1.002611000	-5.193851000	0.878326000
6	1.501486000	-4.453747000	0.912845000
6	0.473709000	-5.594526000	0.966587000
1	-1.353183000	-4.774097000	1.829950000
1	-1.625766000	-6.067541000	0.658278000
1	0.725255000	-6.252289000	0.122338000
1	0.689631000	-6.168679000	1.872938000
8	2.667695000	-4.736667000	1.190459000
1	4.746458000	-0.448638000	-0.004085000
1	3.779111000	-2.781452000	1.361446000
6	3.911233000	-2.789483000	-0.795057000
1	3.771703000	-2.068223000	-1.609801000
1	3.277982000	-3.648617000	-1.022835000
6	5.366178000	-3.250808000	-0.747709000
1	6.044276000	-2.445114000	-0.447212000
1	5.491196000	-4.034255000	0.010352000
6	5.831868000	-3.801837000	-2.074429000
8	5.169282000	-3.862787000	-3.091493000
8	7.111246000	-4.234182000	-2.008562000
6	4.391354000	-0.341695000	2.114823000
1	4.506824000	0.743985000	2.226899000
1	3.644880000	-0.650043000	2.855189000
6	5.726241000	-0.966876000	2.463103000
8	6.528153000	-1.408272000	1.664823000
8	5.943518000	-0.946449000	3.792334000

1	7.085358000	5.400623000	-1.770723000
1	6.830275000	-1.325196000	3.951378000
1	7.346367000	-4.574343000	-2.893357000
1	-4.119924000	-4.297454000	-4.072015000
1	-5.458699000	-1.005322000	-2.932843000

NiI-E

NiI-E-opt.gjf.log

Temperature 298.150 Kelvin. Pressure 1.00000 Atm.

Zero-point correction= 0.924258 (Hartree/Particle)
Thermal correction to Energy= 0.983147
Thermal correction to Enthalpy= 0.984091
Thermal correction to Gibbs Free Energy= 0.822719
Sum of electronic and zero-point Energies= -3106.377016
Sum of electronic and thermal Energies= -3106.318127
Sum of electronic and thermal Enthalpies= -3106.317183
Sum of electronic and thermal Free Energies= -3106.478555

28	0.361405000	0.067914000	-0.368578000
7	-1.549380000	-0.750134000	-0.414384000
7	-0.368227000	1.941033000	-0.171702000
7	2.347886000	0.755257000	-0.653367000
7	1.052523000	-1.802201000	-0.197448000
6	-1.900037000	-2.052751000	-0.542593000
6	-3.415628000	-2.229137000	-0.527954000
6	-2.679518000	0.024880000	-0.377855000
6	-3.919909000	-0.785279000	-0.700389000
6	2.476833000	-2.032664000	0.102361000
6	0.409722000	-2.935471000	-0.253029000
6	1.263116000	-4.144514000	0.080629000
6	2.677706000	-3.562720000	0.274632000
6	-0.986104000	-3.125085000	-0.565128000
6	3.393732000	0.058278000	-0.937637000
6	2.739599000	2.176615000	-0.519292000
6	4.254766000	2.127665000	-0.204421000
6	4.677581000	0.894337000	-1.067282000
6	3.347808000	-1.437307000	-1.013925000
6	0.430772000	3.178170000	-0.121646000
6	-1.638049000	2.266556000	-0.006452000
6	-0.430919000	4.231751000	0.652779000
6	-1.855718000	3.757773000	0.232252000
6	1.837785000	2.941879000	0.439517000
6	-2.732048000	1.372178000	-0.127842000
1	2.296826000	3.919388000	0.629406000

1	1.750184000	2.423862000	1.397086000
1	-2.064558000	4.192246000	-0.757304000
6	-3.010390000	4.168681000	1.165399000
1	-2.744885000	5.098241000	1.678685000
1	-3.156913000	3.419720000	1.951020000
6	-4.330926000	4.426620000	0.432679000
1	-4.201423000	5.209687000	-0.326888000
1	-4.696119000	3.551035000	-0.114000000
6	-5.429689000	4.880142000	1.368081000
8	-5.300071000	5.125252000	2.550244000
8	-6.611651000	4.995927000	0.720722000
1	-3.724302000	1.800690000	-0.063829000
6	-0.209111000	4.202493000	2.165168000
1	0.825277000	4.454936000	2.416497000
1	-0.850938000	4.929354000	2.670664000
1	-0.430123000	3.211324000	2.577245000
6	-0.072269000	5.564829000	-0.021035000
7	0.503704000	3.819976000	-1.445475000
6	0.263287000	5.164159000	-1.460250000
8	0.309120000	5.892465000	-2.443314000
1	2.623037000	2.643282000	-1.504432000
1	4.744104000	3.034224000	-0.579813000
6	4.593443000	2.036190000	1.296925000
1	5.682098000	2.020379000	1.414267000
1	4.263700000	2.964195000	1.776355000
6	4.006403000	0.858831000	2.087454000
1	2.920500000	0.783044000	1.958800000
1	4.408719000	-0.102804000	1.747372000
6	4.302528000	0.970606000	3.573434000
8	4.951909000	1.851297000	4.090155000
8	3.785079000	-0.016953000	4.349413000
6	5.971434000	0.195955000	-0.622584000
1	6.264785000	-0.578610000	-1.338035000
1	6.785961000	0.927697000	-0.577651000
1	5.886963000	-0.285405000	0.351922000
6	4.864093000	1.370258000	-2.548949000
1	5.759142000	2.000884000	-2.588123000
1	4.015641000	1.984384000	-2.862029000
6	4.940344000	0.224932000	-3.550726000
8	3.944602000	-0.433838000	-3.850734000
7	6.165152000	-0.028547000	-4.079816000
1	6.261788000	-0.778402000	-4.751586000
1	6.983127000	0.514267000	-3.846494000
1	2.955474000	-1.743814000	-1.989247000
1	4.357224000	-1.838518000	-0.924096000
1	2.707802000	-1.510282000	1.035380000

1	-0.876926000	6.305348000	-0.003135000
1	0.812263000	6.026824000	0.436048000
1	0.716149000	3.310065000	-2.292807000
1	3.348550000	-3.938728000	-0.505973000
6	3.252620000	-3.949205000	1.642680000
1	3.319376000	-5.042143000	1.730202000
1	2.566274000	-3.624157000	2.439140000
6	4.613001000	-3.352118000	1.962089000
8	5.185900000	-2.516997000	1.293761000
8	5.188888000	-3.782130000	3.107866000
1	0.895165000	-4.569790000	1.027353000
6	1.075248000	-5.205640000	-1.002676000
6	-1.429011000	-4.489995000	-0.895880000
6	-0.395210000	-5.624810000	-0.991361000
1	1.353691000	-4.783067000	-1.977241000
1	1.726575000	-6.069840000	-0.824611000
1	-0.584435000	-6.268981000	-0.120059000
1	-0.663129000	-6.218815000	-1.871192000
8	-2.603738000	-4.808189000	-1.120048000
1	-4.739329000	-0.549093000	-0.015023000
1	-3.729419000	-2.883718000	-1.341842000
6	-3.864584000	-2.859176000	0.810341000
1	-3.750277000	-2.119770000	1.612977000
1	-3.213328000	-3.699328000	1.058148000
6	-5.308405000	-3.353770000	0.755629000
1	-5.997095000	-2.569817000	0.420936000
1	-5.403058000	-4.158819000	0.015698000
6	-5.794998000	-3.880188000	2.083413000
8	-5.164798000	-3.896961000	3.122614000
8	-7.062587000	-4.350670000	1.996663000
6	-4.385580000	-0.476513000	-2.133498000
1	-4.524318000	0.605158000	-2.262860000
1	-3.629670000	-0.779485000	-2.866254000
6	-5.705265000	-1.132033000	-2.476848000
8	-6.537864000	-1.513373000	-1.677681000
8	-5.883573000	-1.218498000	-3.812180000
1	-7.269338000	5.301381000	1.374991000
1	-6.765334000	-1.612112000	-3.961312000
1	-7.308339000	-4.669799000	2.886188000
1	4.634093000	-4.449770000	3.547772000
1	3.277392000	-0.646892000	3.810081000

v) Reactive mode composition factor analysis of TS_1 for **A-E.**

A-TS_1-KED

2 6 0 0.01 0 0.00015

3	6	0	0.02	0	0.00061
4	6	0.01	0	0	0.00015
5	6	0.01	0.02	-0.01	0.00092
6	6	0.02	0	0	0.00061
7	6	0.02	0.01	0	0.00077
8	8	0.04	0.02	-0.01	0.00430
9	6	0	0.01	-0.01	0.00031
10	6	0	0.02	0	0.00061
11	6	0	0	0	0.00000
12	6	0	0	0	0.00000
13	7	-0.01	0.01	-0.04	0.00323
14	6	0	0	-0.01	0.00015
15	6	0	0.01	0	0.00015
16	7	-0.03	0.04	-0.01	0.00466
17	8	0	0	0	0.00000
18	8	0	0	0	0.00000
19	7	-0.03	-0.01	-0.03	0.00340
20	8	0	0	-0.01	0.00020
21	8	-0.01	0	0	0.00020
22	8	0	0	0	0.00000
23	8	0	0	0	0.00000
24	6	-0.01	0	-0.02	0.00077
25	6	0	0.01	-0.01	0.00031
26	6	-0.02	0.01	-0.01	0.00092
27	6	-0.01	0	0	0.00015
28	28	0.02	-0.03	0.02	0.01277
29	6	0	0	-0.03	0.00138
30	6	-0.01	0.02	-0.01	0.00092
31	6	-0.03	0.01	-0.01	0.00169
32	6	-0.01	-0.01	-0.01	0.00046
33	6	0	0	-0.01	0.00015
34	6	0	0.01	0	0.00015
35	6	-0.01	0	0	0.00015
36	6	0	-0.01	0	0.00015
37	6	0.01	0.01	-0.01	0.00046
38	6	0	0.02	0	0.00061
39	6	-0.01	0	0	0.00015
40	6	0	-0.01	-0.01	0.00031
41	6	0	0.01	-0.02	0.00077
42	6	-0.02	0.02	0	0.00123
43	6	-0.02	0	-0.01	0.00077
44	6	-0.01	0	-0.02	0.00077
45	6	0	0	0	0.00000
46	6	0	0	0	0.00000
47	6	0	-0.01	0	0.00015
48	6	0	0	0	0.00000

49	6	0	0	0	0.00000
50	6	0	0	0	0.00000
51	6	0	0	0	0.00000
52	8	0	0	0	0.00000
53	8	0	0.01	0	0.00020
54	8	0	0	0	0.00000
55	6	0	0	0	0.00000
56	6	0	0	0.01	0.00015
57	8	0	0	0	0.00000
58	6	0.01	0.01	-0.01	0.00046
59	6	0	0.02	0	0.00061
60	6	0	0	0	0.00000
61	6	0	-0.01	0	0.00015
62	6	0	0	-0.01	0.00015
63	6	0	0	0	0.00000
64	7	-0.05	0.01	-0.02	0.00538
65	8	0	0	0	0.00000
66	8	0	0	0	0.00000
68	6	0	0	0	0.00000
69	6	0	0	0	0.00000
70	6	0	0	0	0.00000
71	6	0	-0.01	0.01	0.00031
72	16	0.01	-0.01	0	0.00082
73	6	-0.07	0.06	0	0.01306
74	8	0	0.01	0.01	0.00041
75	16	-0.12	0.12	-0.04	0.12476
76	6	-0.03	-0.01	0	0.00154
77	8	0.01	0	0	0.00020
78	16	0	0	0	0.00000
79	8	0.01	0.01	0.01	0.00061
81	6	0	0	0	0.00000
82	6	0	-0.01	0	0.00015
83	6	-0.01	0	0	0.00015
84	6	0	-0.02	0.01	0.00077
85	6	-0.01	0	0.01	0.00031
86	6	-0.01	-0.01	0.01	0.00046
87	8	-0.01	-0.02	0.01	0.00123
89	6	0	0	0	0.00000
90	6	0	0	0.01	0.00015
91	7	-0.02	0.01	0	0.00090
92	8	0.01	0	0.02	0.00102
93	1	0	0	0	0.00000
94	1	0	0	0	0.00000
95	1	-0.01	0.02	0	0.00006
96	1	0.02	-0.01	0	0.00006
97	1	0.01	0.02	-0.01	0.00008

98	1	0.04	-0.01	0.02	0.00027
99	1	0.13	0	0.03	0.00228
100	1	0	0	0	0.00000
101	1	0	0	0	0.00000
102	1	0	-0.02	0	0.00005
103	1	-0.01	0.01	0	0.00003
104	1	0	-0.04	0.01	0.00022
105	1	-0.01	0	0.01	0.00003
106	1	-0.01	-0.06	0.02	0.00052
107	1	0	0	-0.01	0.00001
108	1	0.01	0	-0.01	0.00003
109	1	-0.01	0.02	0.01	0.00008
110	1	0	0.02	0	0.00005
111	1	0	0.01	-0.01	0.00003
112	1	0	0.01	-0.01	0.00003
113	1	-0.01	0.01	0	0.00003
114	1	0	0.01	0	0.00001
115	1	0	0	-0.01	0.00001
116	1	0.01	0	0	0.00001
117	1	-0.01	0.01	0	0.00003
118	1	0.01	0.01	-0.01	0.00004
119	1	0	0.02	0	0.00005
120	1	0	0	0	0.00000
121	1	0	0	0	0.00000
122	1	0	0	0	0.00000
123	1	0	0	0	0.00000
124	1	0	-0.01	0	0.00001
125	1	0	-0.01	0	0.00001
126	1	0	0	0	0.00000
127	1	0	0	-0.01	0.00001
128	1	0	0	0.01	0.00001
129	1	0	0	0.01	0.00001
130	1	0	0	0.01	0.00001
131	1	0.01	0.01	-0.02	0.00008
132	1	0.01	0	-0.01	0.00003
133	1	0.01	0	-0.01	0.00003
134	1	0.01	0.01	0.01	0.00004
135	1	0	0.02	0.01	0.00006
136	1	0	0.01	0	0.00001
137	1	0	0	0	0.00000
138	1	0	0	0	0.00000
139	1	0	-0.01	0	0.00001
140	1	0	-0.01	-0.01	0.00003
141	1	0	0	0	0.00000
142	1	0	0	0	0.00000
143	1	0	0	0	0.00000

144	1	0	0	0	0.00000
145	1	0	0	0	0.00000
146	1	0	0	0	0.00000
147	1	-0.01	0	0	0.00001
148	1	0	0	0	0.00000
149	1	0	-0.01	0.01	0.00003
150	1	0	0	0.01	0.00001
151	1	0.02	0.02	0.06	0.00056
152	1	-0.06	0.1	0.1	0.00302
153	1	-0.06	0.04	-0.02	0.00072
154	1	0	-0.01	0.01	0.00003
155	1	-0.05	-0.05	-0.02	0.00069
156	1	0	0	0	0.00000
157	1	0	0	0	0.00000
158	1	0	0.01	0	0.00001
159	1	0.01	0	0	0.00001
163	6	0.5	-0.46	0.15	0.74361
164	1	0.2	-0.12	-0.02	0.00701
165	1	0.26	-0.31	0	0.02095
166	1	0.26	-0.26	0.1	0.01859
167	1	0	0	0	0.00000
168	1	0	0	0	0.00000
169	1	0	0	0	0.00000
170	1	0	0	0	0.00000
171	1	0	0	0	0.00000
172	1	0	0	0	0.00000
173	1	-0.04	0.01	-0.01	0.00023
174	1	-0.06	-0.01	0.01	0.00049
175	1	0	0.01	0	0.00001
177	1	0	-0.01	0.01	0.00003
178	1	0	0	0	0.00000
179	6	0	0	0	0.00000
180	8	0	0	0	0.00000
181	8	0	0	0	0.00000
182	1	0	0	0	0.00000
183	8	0	0	0	0.00000
184	8	0	0	0	0.00000

B-TS$_1$-KED

2	6	0	0.01	0	0.00015
3	6	0	0.02	0	0.00062
4	6	0.01	0	0	0.00015
5	6	0.01	0.02	-0.01	0.00093
6	6	0.03	0	0	0.00139
7	6	0.03	0.01	0	0.00155
8	8	0.05	0.02	-0.01	0.00620

9	6	0	0	-0.01	0.00015
10	6	0	0.02	0	0.00062
11	6	0	0	0	0.00000
12	6	0	0	0	0.00000
13	7	-0.02	0.01	-0.04	0.00379
14	6	0	0	-0.01	0.00015
15	6	0	0.01	0	0.00015
16	7	-0.04	0.04	-0.01	0.00596
17	8	0	0	0	0.00000
18	8	0	0	0	0.00000
19	7	-0.03	-0.01	-0.02	0.00253
20	8	0	0	0	0.00000
21	8	-0.01	0	0	0.00021
22	8	0	0	0	0.00000
23	8	0	0	0	0.00000
24	6	-0.01	0	-0.01	0.00031
25	6	0	0.01	0	0.00015
26	6	-0.02	0.01	0	0.00077
27	6	-0.01	0	0	0.00015
28	28	0.03	-0.03	0.01	0.01439
29	6	-0.01	0	-0.02	0.00077
30	6	-0.01	0.02	-0.01	0.00093
31	6	-0.02	0.01	0	0.00077
32	6	-0.02	-0.01	-0.01	0.00093
33	6	0	0	-0.01	0.00015
34	6	0	0.01	0	0.00015
35	6	-0.01	0	0	0.00015
36	6	0	-0.01	0	0.00015
37	6	0	0.01	-0.01	0.00031
38	6	0	0.02	0.01	0.00077
39	6	-0.01	0	0	0.00015
40	6	0	-0.01	-0.01	0.00031
41	6	0	0.01	-0.01	0.00031
42	6	-0.02	0.02	0	0.00124
43	6	-0.02	0	0	0.00062
44	6	-0.01	0	-0.02	0.00077
45	6	0	0	0	0.00000
46	6	0	0	0.01	0.00015
47	6	0	-0.01	0	0.00015
48	7	0	0.01	0	0.00018
49	6	0	0	0	0.00000
50	6	0	0.01	0	0.00015
51	6	0	0	0	0.00000
52	6	0	0	0	0.00000
53	8	0	0	0	0.00000
54	8	0.01	0.01	0	0.00041

55	8	0	0	0	0.00000
56	6	0	0	0	0.00000
57	6	0	0	0.01	0.00015
58	7	0	0	0.01	0.00018
59	8	0	0	0	0.00000
60	6	0.01	0	-0.01	0.00031
61	6	0	0.01	0	0.00015
62	6	0	0	0	0.00000
63	6	0	-0.01	0	0.00015
64	6	0	0	-0.01	0.00015
65	6	0	0	0	0.00000
66	7	-0.05	0.01	-0.01	0.00488
67	8	0	0	0	0.00000
68	8	0	0	0	0.00000
70	6	0	0	0	0.00000
71	6	0	0	0	0.00000
72	6	0	0	0	0.00000
73	6	0	-0.01	0.01	0.00031
74	16	0.01	-0.01	0	0.00083
75	6	-0.06	0.05	0	0.00945
76	8	0	0.01	0.01	0.00041
77	16	-0.12	0.12	-0.04	0.12579
78	6	-0.02	-0.01	0	0.00077
79	8	0.01	0	0	0.00021
80	16	0.01	0	0	0.00041
81	8	0.01	0.01	0	0.00041
83	6	0	0	0	0.00000
84	6	0	-0.01	0	0.00015
85	6	-0.01	0	0	0.00015
86	6	0	-0.02	0.01	0.00077
87	6	-0.01	0	0.01	0.00031
88	6	-0.01	-0.01	0.01	0.00046
89	8	-0.01	-0.02	0.01	0.00139
91	6	0	0	0	0.00000
92	6	0	0	0.01	0.00015
93	7	-0.02	0.01	0.01	0.00108
94	8	0.01	0	0.02	0.00103
95	1	0	0	0	0.00000
96	1	0	0	0	0.00000
97	1	-0.01	0.02	0	0.00006
98	1	0.02	-0.01	0	0.00006
99	1	0.01	0.02	0	0.00006
100	1	0.05	-0.01	0.01	0.00035
101	1	0.16	-0.01	0.02	0.00337
102	1	0	0	0	0.00000
103	1	0	0	0	0.00000

104	1	0	-0.02	0	0.00005
105	1	-0.01	0.01	0	0.00003
106	1	0	-0.05	0.01	0.00034
107	1	-0.01	0	0.01	0.00003
108	1	-0.01	-0.1	0.02	0.00136
109	1	0	0	-0.01	0.00001
110	1	0	0	-0.01	0.00001
111	1	0	0.02	0.01	0.00006
112	1	0	0.02	0	0.00005
113	1	0	0	-0.01	0.00001
114	1	0	0	-0.01	0.00001
115	1	-0.01	0.01	0	0.00003
116	1	0	0.01	0	0.00001
117	1	0	-0.01	-0.01	0.00003
118	1	0.01	0	0	0.00001
119	1	0	0	0.01	0.00001
120	1	0.01	0	-0.01	0.00003
121	1	0	0.02	0.01	0.00006
122	1	-0.01	0	0	0.00001
123	1	0	0	0	0.00000
124	1	0	0	0	0.00000
125	1	0	0	0	0.00000
126	1	0	-0.01	0	0.00001
127	1	0	-0.01	0	0.00001
128	1	0	0	0	0.00000
129	1	0	0	-0.01	0.00001
130	1	0	0	0.01	0.00001
131	1	0	0	0.01	0.00001
132	1	0	0	0.01	0.00001
133	1	0.01	0.01	-0.02	0.00008
134	1	0.01	0	-0.01	0.00003
135	1	0.01	0	-0.01	0.00003
136	1	0.01	0.01	0	0.00003
137	1	0	0.02	0.01	0.00006
138	1	0	0.01	0	0.00001
139	1	0	0	0	0.00000
140	1	0	0	0	0.00000
141	1	0	-0.01	0	0.00001
142	1	0	-0.01	-0.01	0.00003
143	1	0	0	0	0.00000
144	1	0	0	0	0.00000
145	1	0	0	0	0.00000
146	1	0	0	0	0.00000
147	1	0	0	0	0.00000
148	1	0	0	0	0.00000
149	1	-0.01	0	0	0.00001

150	1	0	0	0	0.00000
151	1	-0.01	-0.01	0.01	0.00004
152	1	0	0	0	0.00000
153	1	0.01	0.02	0.05	0.00039
154	1	-0.05	0.08	0.11	0.00271
155	1	-0.04	0.03	-0.02	0.00037
156	1	0.01	-0.01	0.01	0.00004
157	1	-0.04	-0.04	-0.02	0.00046
158	1	0	0	0	0.00000
159	1	0	0	0	0.00000
160	1	0	0.01	0	0.00001
161	1	0	0	0	0.00000
165	6	0.5	-0.46	0.13	0.74109
166	1	0.21	-0.14	-0.01	0.00823
167	1	0.26	-0.31	0	0.02113
168	1	0.25	-0.26	0.09	0.01784
169	1	0	0	0	0.00000
170	1	0	0	0	0.00000
171	1	0	0	0	0.00000
172	1	0	0	0	0.00000
173	1	0	0	0	0.00000
174	1	0	-0.01	0.01	0.00003
175	1	0	-0.01	0.01	0.00003
176	1	-0.05	0.01	-0.01	0.00035
177	1	-0.07	-0.01	0.02	0.00070
178	1	0	0.01	0.01	0.00003
180	1	0	-0.01	0.01	0.00003
181	1	0	0	0	0.00000
182	6	0	0	0	0.00000
183	8	0	0	0	0.00000
184	8	0	0	0	0.00000
185	1	0	0	0	0.00000
186	1	0	0.01	0.02	0.00006

C-TS$_1$-KED

2	6	0	0	0	0.00000
3	6	0	0.01	0	0.00014
4	6	0.01	0	0	0.00014
5	6	0	0.01	-0.01	0.00028
6	6	0.02	0	0	0.00055
7	6	0.02	0.01	0	0.00069
8	8	0.03	0.01	-0.01	0.00202
9	6	0	0.01	-0.01	0.00028
10	6	-0.01	0.02	0	0.00069
11	6	-0.01	0	0	0.00014

12	6	0	-0.01	0	0.00014
13	7	-0.02	0.01	-0.03	0.00225
14	6	0	0.01	-0.01	0.00028
15	6	-0.01	0.01	0	0.00028
16	7	-0.03	0.02	-0.01	0.00225
17	8	0	0	0	0.00000
18	8	0	-0.01	0	0.00018
19	7	-0.02	0	-0.02	0.00129
20	8	0	0	0	0.00000
21	8	-0.01	0	0	0.00018
22	8	0	0	0	0.00000
23	8	0	0	0	0.00000
24	6	-0.01	0.01	-0.01	0.00041
25	6	-0.01	0.01	0	0.00028
26	6	-0.02	0.01	0	0.00069
27	6	-0.01	0	0	0.00014
28	28	0.02	-0.01	0.02	0.00606
29	6	-0.01	0.01	-0.02	0.00083
30	6	-0.01	0.01	-0.01	0.00041
31	6	-0.02	0.01	0	0.00069
32	6	-0.01	0	-0.01	0.00028
33	6	0	0.01	-0.01	0.00028
34	6	-0.01	0.01	0	0.00028
35	6	-0.01	0	0	0.00014
36	6	0	-0.01	0	0.00014
37	6	0	0.01	-0.01	0.00028
38	6	-0.01	0.02	0	0.00069
39	6	-0.01	0	0	0.00014
40	6	-0.01	0	0	0.00014
41	6	-0.01	0.01	-0.01	0.00041
42	6	-0.02	0.02	0	0.00110
43	6	-0.02	0	0	0.00055
44	6	-0.01	0	-0.01	0.00028
45	6	0	0	0	0.00000
46	6	0	0	-0.01	0.00014
47	6	0	0	0	0.00000
48	7	0	0	0	0.00000
49	6	0.01	0	0	0.00014
50	6	0	0	0	0.00000
51	6	0	0	0	0.00000
52	6	0	0	0	0.00000
53	8	0.01	0	0	0.00018
54	8	0	0	-0.01	0.00018
55	8	0	0	0	0.00000
56	6	0	0	0	0.00000
57	6	-0.01	0	0	0.00014

58	7	0	0	0	0.00000
59	8	0	0	-0.01	0.00018
60	6	0.01	0.01	-0.01	0.00041
61	6	0	0.01	0	0.00014
62	6	-0.01	0	0	0.00014
63	6	0	-0.01	0	0.00014
64	6	0	0	0	0.00000
65	6	-0.01	0.01	0	0.00028
66	7	-0.04	0.01	-0.01	0.00289
67	8	-0.01	0	0	0.00018
68	8	0	0	0	0.00000
70	6	0	0	0	0.00000
71	6	0	0	0	0.00000
72	6	0	0	0	0.00000
73	6	0	-0.01	0.01	0.00028
74	16	0.02	-0.02	0	0.00294
75	6	-0.05	0.03	0.01	0.00482
76	8	0	0.01	0.01	0.00037
77	16	-0.12	0.09	-0.03	0.08607
78	6	-0.02	0	0	0.00055
79	8	0	0.01	0	0.00018
80	16	0	0.01	0.01	0.00074
81	8	0	0.01	0.01	0.00037
83	6	0	0	0	0.00000
84	6	0	-0.01	0.01	0.00028
85	6	-0.01	0	0	0.00014
86	6	0	-0.02	0.01	0.00069
87	6	-0.01	0	0.01	0.00028
88	6	-0.01	-0.01	0.01	0.00041
89	8	-0.01	-0.02	0.02	0.00165
91	6	0.01	0.01	0	0.00028
92	6	-0.01	0	0.01	0.00028
93	7	-0.03	0.01	0	0.00161
94	8	0	0	0.02	0.00073
95	1	0	0	0	0.00000
96	1	0	0	0	0.00000
97	1	-0.01	0.02	0	0.00006
98	1	0.02	-0.01	0	0.00006
99	1	0	0.02	-0.01	0.00006
100	1	0.03	0	0.01	0.00011
101	1	0.07	0.01	0.01	0.00059
102	1	0	0	0	0.00000
103	1	0	0	0	0.00000
104	1	0.01	-0.02	0.01	0.00007
105	1	-0.01	0.01	0	0.00002
106	1	0.01	-0.04	0.01	0.00021

107	1	-0.01	0	0.01	0.00002
108	1	-0.01	-0.03	0.02	0.00016
109	1	0	0.01	-0.01	0.00002
110	1	0	0.01	-0.01	0.00002
111	1	-0.01	0.02	0.01	0.00007
112	1	-0.01	0.02	0	0.00006
113	1	0	0.01	-0.01	0.00002
114	1	0	0.01	-0.01	0.00002
115	1	-0.01	0.01	0	0.00002
116	1	0	0.01	0	0.00001
117	1	-0.01	0.01	-0.01	0.00003
118	1	-0.01	0	-0.01	0.00002
119	1	0	0	0	0.00000
120	1	0	0.01	0	0.00001
121	1	-0.01	0	0	0.00001
122	1	-0.01	-0.01	0	0.00002
123	1	0	0.01	-0.01	0.00002
124	1	-0.01	0.01	0	0.00002
125	1	0	0	0	0.00000
126	1	0	0	0	0.00000
127	1	-0.01	0	0	0.00001
128	1	-0.01	-0.01	-0.02	0.00007
129	1	0	0.01	0	0.00001
130	1	0	0	0	0.00000
131	1	0	0	-0.01	0.00001
132	1	0	0.01	-0.01	0.00002
133	1	0	0	0	0.00000
134	1	0	0	0	0.00000
135	1	0	0	0	0.00000
136	1	0	0	0	0.00000
137	1	0	0	0	0.00000
138	1	-0.01	0	0	0.00001
139	1	-0.01	-0.01	0	0.00002
140	1	0.01	0.01	-0.02	0.00007
141	1	0.01	0.01	-0.01	0.00003
142	1	0.01	0.01	-0.01	0.00003
143	1	0	0.01	0.01	0.00002
144	1	-0.01	0.02	0.01	0.00007
145	1	0	0.01	0	0.00001
146	1	-0.01	0	0	0.00001
147	1	-0.01	0	0	0.00001
148	1	0	-0.01	-0.01	0.00002
149	1	0	-0.01	0	0.00001
150	1	0	0	0	0.00000
151	1	0	0	0	0.00000
152	1	0.01	0	0	0.00001

153	1	0.01	0	0	0.00001
154	1	0	0	0	0.00000
155	1	0	0	0	0.00000
156	1	-0.01	0	0	0.00001
157	1	0	0	0	0.00000
158	1	0	-0.02	0.01	0.00006
159	1	0	-0.01	0.01	0.00002
160	1	0.01	0	0.03	0.00011
161	1	-0.06	0.03	0.1	0.00166
162	1	-0.01	0.01	-0.01	0.00003
163	1	-0.01	0	0.01	0.00002
164	1	-0.02	-0.02	-0.01	0.00010
165	1	0	0	0.01	0.00001
166	1	0	0	0	0.00000
167	1	0.01	0.02	0	0.00006
168	1	0.01	0	-0.01	0.00002
172	6	0.62	-0.45	0.11	0.82470
173	1	0.18	-0.01	-0.03	0.00383
174	1	0.31	-0.27	-0.08	0.02012
175	1	0.22	-0.23	0.08	0.01236
176	1	0	0	0	0.00000
177	1	-0.01	0	0	0.00001
178	1	-0.01	0	0	0.00001
179	1	0	0	-0.01	0.00001
180	1	0	0	0	0.00000
181	1	0.01	0	0	0.00001
182	1	0.01	0	0	0.00001
183	1	-0.04	0	-0.01	0.00020
184	1	-0.07	-0.02	0	0.00061
185	1	0	0	0	0.00000
187	1	0.01	-0.01	0.01	0.00003
188	1	0	0	0	0.00000
189	6	0	0	0	0.00000
190	8	0	0	0	0.00000
191	8	0	0	0	0.00000
192	1	0	0	0	0.00000
193	1	0	0	0.01	0.00001

D-TS$_1$-KED

2	6	0	0	0	0.00000
3	6	0	0.01	-0.01	0.00029
4	6	0.02	0	0	0.00058
5	6	0	0.01	-0.01	0.00029
6	6	0.03	0	0.01	0.00146
7	6	0.02	0.01	0	0.00073
8	8	0.03	0.01	0	0.00194

9	6	0	0.01	-0.01	0.00029
10	6	-0.01	0.02	0	0.00073
11	6	-0.01	0	0	0.00015
12	6	0	0	0	0.00000
13	7	-0.01	0.01	-0.02	0.00102
14	6	0	0	-0.01	0.00015
15	6	-0.01	0.01	0	0.00029
16	7	-0.03	0.02	0	0.00221
17	8	0	0	0	0.00000
18	8	0	0	0	0.00000
19	7	-0.03	0	-0.02	0.00221
20	8	0	0	0	0.00000
21	8	-0.01	0	0	0.00019
22	8	0	0	0	0.00000
23	8	0	0	0	0.00000
24	6	-0.02	0.01	-0.01	0.00087
25	6	0	0.01	-0.01	0.00029
26	6	-0.02	0.01	0	0.00073
27	6	-0.01	0	0	0.00015
28	28	0.01	-0.01	0.02	0.00427
29	6	-0.01	0.01	-0.01	0.00044
30	6	-0.01	0.01	0	0.00029
31	6	-0.02	0.01	0	0.00073
32	6	-0.01	0	-0.01	0.00029
33	6	0	0.01	-0.01	0.00029
34	6	-0.01	0.01	0	0.00029
35	6	-0.01	0	0	0.00015
36	6	-0.01	0	0	0.00015
37	6	0	0.01	-0.01	0.00029
38	6	-0.01	0.02	0.01	0.00087
39	6	-0.01	0	0	0.00015
40	6	-0.01	0	0	0.00015
41	6	0	0.01	-0.01	0.00029
42	6	-0.02	0.02	0	0.00116
43	6	-0.02	0	0	0.00058
44	6	-0.02	0	-0.01	0.00073
45	6	0	0	0	0.00000
46	6	0	0.01	-0.01	0.00029
47	6	0	0	0	0.00000
48	7	-0.01	0	0	0.00017
49	6	0	0	0	0.00000
50	6	-0.01	0	0	0.00015
51	6	0	0	0	0.00000
52	6	0	0	0	0.00000
53	8	0	0	0	0.00000
54	8	-0.01	0	0	0.00019

55	8	0	0	0	0.00000
56	6	0	0.01	0	0.00015
57	6	-0.01	0	0	0.00015
58	7	0	0	0	0.00000
59	8	0	0.01	-0.01	0.00039
60	6	0	0.01	-0.01	0.00029
61	6	0	0.01	0	0.00015
62	6	-0.01	0	0	0.00015
63	6	0	0	0	0.00000
64	6	0	0	0	0.00000
65	6	-0.01	0	0	0.00015
66	7	-0.04	0.02	-0.01	0.00357
67	8	-0.01	0	0	0.00019
68	8	0	0	0	0.00000
70	6	0.01	0	0	0.00015
71	6	0	0	0	0.00000
72	6	0	0	0	0.00000
73	6	0.01	-0.01	0.01	0.00044
74	16	0.03	-0.02	0	0.00506
75	6	-0.06	0.04	0	0.00757
76	8	0	0.01	0.01	0.00039
77	16	-0.12	0.08	-0.02	0.08247
78	6	-0.02	0	-0.01	0.00073
79	8	0.01	0	0	0.00019
80	16	0	0	0	0.00000
81	8	0	0.01	0.01	0.00039
83	6	0	0	0	0.00000
84	6	0	-0.01	0	0.00015
85	6	-0.01	0	0.01	0.00029
86	6	0	-0.02	0	0.00058
87	6	-0.01	0	0.01	0.00029
88	6	-0.01	-0.01	0.01	0.00044
89	8	-0.01	-0.02	0.01	0.00116
91	6	0.01	0	0	0.00015
92	6	0	0	0	0.00000
93	7	-0.01	0	0	0.00017
94	8	0	0	0	0.00000
95	1	0	0	0	0.00000
96	1	0	0	0	0.00000
97	1	-0.01	0.01	-0.01	0.00004
98	1	0.02	-0.01	0.01	0.00007
99	1	0	0.02	-0.01	0.00006
100	1	0.04	0	0.02	0.00024
101	1	0.06	0.01	0.01	0.00046
102	1	0	0	0	0.00000
103	1	0	0	0	0.00000

104	1	0.01	-0.02	0	0.00006
105	1	-0.01	0.01	0	0.00002
106	1	0.01	-0.04	0	0.00021
107	1	-0.01	0	0.01	0.00002
108	1	-0.01	-0.01	0.02	0.00007
109	1	0	0.01	-0.01	0.00002
110	1	0	0.01	0	0.00001
111	1	-0.01	0.02	0.01	0.00007
112	1	-0.01	0.02	0	0.00006
113	1	0	0	-0.01	0.00001
114	1	0	0	-0.01	0.00001
115	1	-0.01	0.01	0	0.00002
116	1	0	0.01	0	0.00001
117	1	-0.02	0.01	0	0.00006
118	1	-0.01	0	0	0.00001
119	1	0	0.02	-0.01	0.00006
120	1	0	0.01	0	0.00001
121	1	-0.01	0.01	0.01	0.00004
122	1	-0.01	0	0	0.00001
123	1	-0.01	-0.01	0	0.00002
124	1	0	0.01	-0.01	0.00002
125	1	-0.01	0.02	0.01	0.00007
126	1	0	0	0	0.00000
127	1	0	0.01	0	0.00001
128	1	0	0	0	0.00000
129	1	-0.01	0	-0.02	0.00006
130	1	0	0	0	0.00000
131	1	0	0	0	0.00000
132	1	0	0.01	-0.01	0.00002
133	1	0	0.01	-0.01	0.00002
134	1	0	0	0	0.00000
135	1	0	0	0	0.00000
136	1	0	0	0	0.00000
137	1	-0.01	0	0	0.00001
138	1	0	0	0	0.00000
139	1	-0.01	0	0	0.00001
140	1	-0.01	-0.01	0	0.00002
141	1	0	0.01	-0.01	0.00002
142	1	0	0.01	-0.01	0.00002
143	1	0	0.01	-0.01	0.00002
144	1	0	0.01	0	0.00001
145	1	0	0.01	0	0.00001
146	1	0	0.01	0	0.00001
147	1	-0.01	0	0	0.00001
148	1	-0.01	0	0	0.00001
149	1	0	-0.01	-0.01	0.00002

150	1	0	0	0	0.00000
151	1	0	0	0	0.00000
152	1	0	0	0	0.00000
153	1	0.01	0	-0.01	0.00002
154	1	0.01	0.01	0	0.00002
155	1	0	0	0	0.00000
156	1	0	0	0	0.00000
157	1	0	-0.01	0	0.00001
158	1	0.01	0	0	0.00001
159	1	0.01	-0.02	0.02	0.00011
160	1	0	-0.01	0.01	0.00002
161	1	0.02	-0.02	0.02	0.00015
162	1	-0.06	0.05	0.08	0.00152
163	1	-0.05	0.02	-0.02	0.00040
164	1	-0.01	0	0	0.00001
165	1	-0.03	-0.03	-0.03	0.00033
166	1	0	0	0	0.00000
167	1	0	0	0	0.00000
168	1	0.01	0.01	0	0.00002
169	1	0.01	0	-0.01	0.00002
173	6	0.63	-0.4	0.08	0.82020
174	1	0.23	-0.02	-0.02	0.00652
175	1	0.3	-0.24	-0.07	0.01850
176	1	0.29	-0.22	0.09	0.01706
177	1	0	0	0	0.00000
178	1	0	0	0	0.00000
179	1	-0.01	0	0	0.00001
180	1	0	0	-0.01	0.00001
181	1	0	0	0	0.00000
182	1	0.01	0	0	0.00001
183	1	0.01	0	0	0.00001
184	1	-0.02	-0.01	0	0.00006
185	1	-0.05	-0.03	0	0.00041
186	1	-0.02	-0.02	0.01	0.00011
188	1	0.01	-0.01	0.01	0.00004
189	1	0	0	0	0.00000
190	6	0	0	0	0.00000
191	8	0	0	0	0.00000
192	8	0	0	0	0.00000
193	1	0	0	0	0.00000

E-TS$_1$-KED

2	6	0	0	0	0.00000
3	6	0	0.01	0	0.00014
4	6	0.01	0	0	0.00014
5	6	0	0.01	0	0.00014

6	6	0.02	0	-0.01	0.00071
7	6	0.01	0.01	-0.01	0.00043
8	8	0.02	0.01	0	0.00095
9	6	0	0.01	0	0.00014
10	6	-0.01	0.01	-0.01	0.00043
11	6	-0.01	0	0	0.00014
12	6	0	0	0	0.00000
13	7	-0.02	0.02	0.01	0.00149
14	6	0	0.01	0	0.00014
15	6	-0.01	0.01	0	0.00028
16	7	-0.03	0.02	-0.01	0.00232
17	8	0	0	0	0.00000
18	8	0	-0.01	0	0.00019
19	7	-0.02	0.01	0.02	0.00149
20	8	0	0	0	0.00000
21	8	-0.01	0.01	0	0.00038
22	8	0	0	0	0.00000
23	8	-0.01	0	0	0.00019
24	6	-0.01	0.01	0.01	0.00043
25	6	0	0.01	0	0.00014
26	6	-0.02	0.01	0	0.00071
27	6	-0.01	0	0	0.00014
28	28	0.01	-0.02	0	0.00348
29	6	-0.01	0.01	0.01	0.00043
30	6	-0.01	0.01	0	0.00028
31	6	-0.02	0.01	0	0.00071
32	6	-0.01	0	0.01	0.00028
33	6	0	0.01	0	0.00014
34	6	-0.01	0.01	-0.01	0.00043
35	6	-0.01	0	0	0.00014
36	6	-0.01	0	0.01	0.00028
37	6	0	0.01	0	0.00014
38	6	-0.01	0.01	-0.01	0.00043
39	6	-0.01	0	0	0.00014
40	6	-0.01	0	0	0.00014
41	6	0	0.01	0	0.00014
42	6	-0.02	0.01	-0.01	0.00085
43	6	-0.02	0	0	0.00057
44	6	-0.01	0.01	0.01	0.00043
45	6	0	0	0	0.00000
46	6	0	0.01	0	0.00014
47	6	0	-0.01	0	0.00014
48	7	-0.01	0	0	0.00017
49	6	0	0	0	0.00000
50	6	-0.01	0	0	0.00014
51	6	0	0.01	0	0.00014

52	6	0	-0.01	0	0.00014
53	6	0	0	0	0.00000
54	8	0	0	0	0.00000
55	8	-0.01	0	0.01	0.00038
56	8	0	0	0	0.00000
57	6	-0.01	0.01	0	0.00028
58	6	-0.01	0	0	0.00014
59	7	0	0	0	0.00000
60	8	0	0.01	0	0.00019
61	8	0	0	0	0.00000
62	6	0	0.01	0	0.00014
63	6	-0.01	0.01	-0.01	0.00043
64	6	-0.01	0	0	0.00014
65	6	0	0	0	0.00000
66	6	0	0	0	0.00000
67	6	-0.01	0.01	0	0.00028
68	7	-0.04	0.01	0	0.00282
69	8	-0.01	0	0	0.00019
70	8	0	0	0	0.00000
72	6	0.01	0	0	0.00014
73	6	0.01	0	0	0.00014
74	6	0.01	0	0	0.00014
75	6	0.01	-0.02	0	0.00071
76	16	0.03	-0.01	0.01	0.00418
77	6	-0.05	0.02	-0.01	0.00427
78	8	0	0	-0.01	0.00019
79	16	-0.11	0.08	-0.01	0.07071
80	6	-0.02	0	0	0.00057
81	8	0	0	0	0.00000
82	16	0	0	0	0.00000
83	8	0	0	-0.01	0.00019
85	6	0	0	0	0.00000
86	6	0.01	-0.02	0.01	0.00085
87	6	-0.01	0	-0.01	0.00028
88	6	0.01	-0.02	0.01	0.00085
89	6	-0.01	0	-0.01	0.00028
90	6	-0.01	-0.01	0	0.00028
91	8	-0.01	-0.02	0	0.00095
93	6	0.01	0.01	0	0.00028
94	6	-0.01	0	-0.01	0.00028
95	7	-0.02	0	-0.01	0.00083
96	8	0	-0.01	-0.01	0.00038
97	1	0	0	0	0.00000
98	1	0	0	0	0.00000
99	1	-0.01	0.02	0	0.00006
100	1	0.02	-0.01	-0.01	0.00007

101	1	0	0.02	0	0.00005
102	1	0.03	-0.01	-0.02	0.00017
103	1	0.05	0.01	-0.02	0.00036
104	1	0	0	0	0.00000
105	1	0	0	0	0.00000
106	1	0.01	-0.02	0.02	0.00011
107	1	-0.02	0.01	-0.01	0.00007
108	1	0.02	-0.04	0.03	0.00034
109	1	-0.02	0	-0.02	0.00009
110	1	-0.01	-0.02	-0.02	0.00011
111	1	0	0.01	0	0.00001
112	1	0	0.01	0	0.00001
113	1	-0.01	0.01	-0.01	0.00004
114	1	-0.01	0.01	-0.01	0.00004
115	1	0	0.01	0	0.00001
116	1	0	0.01	0.01	0.00002
117	1	-0.01	0.01	0	0.00002
118	1	-0.01	0.01	0	0.00002
119	1	-0.02	0.01	0	0.00006
120	1	-0.01	0.01	0	0.00002
121	1	-0.01	0.02	0	0.00006
122	1	0	0.01	0	0.00001
123	1	-0.01	0	0	0.00001
124	1	-0.01	0	0	0.00001
125	1	-0.01	-0.01	0.01	0.00004
126	1	0	0.01	0	0.00001
127	1	-0.01	0.01	-0.01	0.00004
128	1	0	0	0	0.00000
129	1	0	0	0	0.00000
130	1	0	0	0	0.00000
131	1	-0.01	0.01	0.02	0.00007
132	1	0	0	0	0.00000
133	1	0	0	0	0.00000
134	1	0	0.01	0	0.00001
135	1	0	0.01	0	0.00001
136	1	0	-0.01	0	0.00001
137	1	0	0	0	0.00000
138	1	0	-0.01	0	0.00001
139	1	0.01	-0.01	0	0.00002
140	1	-0.01	0.01	0	0.00002
141	1	0	0	0	0.00000
142	1	-0.01	0	0	0.00001
143	1	-0.01	-0.01	0	0.00002
144	1	0	0.01	0	0.00001
145	1	0	0.01	0	0.00001
146	1	0.01	0.01	0	0.00002

147	1	0	0.01	-0.01	0.00002
148	1	-0.01	0.01	-0.01	0.00004
149	1	-0.01	0.01	-0.01	0.00004
150	1	-0.01	0	0	0.00001
151	1	-0.01	-0.01	0	0.00002
152	1	0	0	0.01	0.00001
153	1	0	0	0	0.00000
154	1	0	0	0	0.00000
155	1	0	0	0	0.00000
156	1	0.01	0	0.01	0.00002
157	1	0.01	0.01	0	0.00002
158	1	0.01	0	0	0.00001
159	1	0	0	0	0.00000
160	1	0	0	0.01	0.00001
161	1	0.01	0	0	0.00001
162	1	0.01	-0.03	-0.01	0.00013
163	1	0	-0.01	0	0.00001
164	1	0.03	-0.01	-0.03	0.00023
165	1	-0.06	-0.01	-0.08	0.00120
166	1	-0.03	0.02	0.01	0.00017
167	1	-0.01	0	0	0.00001
168	1	-0.02	-0.01	0.03	0.00017
169	1	0	0	-0.01	0.00001
170	1	0	0	0.01	0.00001
171	1	0.01	0.02	-0.01	0.00007
172	1	0.01	0.01	0.01	0.00004
176	6	0.64	-0.42	0.08	0.84290
177	1	0.2	-0.01	0	0.00475
178	1	0.31	-0.18	0.15	0.01790
179	1	0.28	-0.25	0	0.01671
180	1	-0.01	0	0	0.00001
181	1	-0.01	0	0	0.00001
182	1	-0.01	0	0	0.00001
183	1	0	0.01	0	0.00001
184	1	0	0	0	0.00000
185	1	0.01	0	0	0.00001
186	1	0.01	0	0	0.00001
187	1	-0.03	0	0.01	0.00012
188	1	-0.06	-0.02	0.01	0.00049
189	1	-0.02	-0.02	0	0.00009

vi) Reactants and transition states (TS1) of charged carboxylic groups for **A**-**E**

A-RC-charged
A-RC-charged-opt.gjf.log

Temperature 298.150 Kelvin. Pressure 1.00000 Atm.

Zero-point correction= 1.326294 (Hartree/Particle)
Thermal correction to Energy= 1.425543
Thermal correction to Enthalpy= 1.426487
Thermal correction to Gibbs Free Energy= 1.168216
Sum of electronic and zero-point Energies= -5892.308090
Sum of electronic and thermal Energies= -5892.208841
Sum of electronic and thermal Enthalpies= -5892.207897
Sum of electronic and thermal Free Energies= -5892.466168

6	1.828499000	4.344167000	-4.015015000
6	1.823616000	2.833001000	-3.956915000
6	1.956041000	2.048047000	-5.112444000
6	1.760151000	2.164355000	-2.725816000
6	2.059753000	0.655662000	-5.043852000
6	1.854559000	0.776678000	-2.639742000
6	2.031234000	0.014709000	-3.798950000
8	2.156526000	-1.349848000	-3.779616000
6	-1.530590000	3.162090000	4.180675000
6	0.228286000	-4.076115000	3.633067000
6	6.537294000	-3.374627000	-4.249409000
6	3.558564000	5.808598000	-1.243689000
7	0.249429000	1.672964000	1.751453000
6	-2.529941000	4.204280000	4.703452000
6	1.154843000	-5.229807000	3.206704000
7	0.177626000	-1.267948000	1.754045000
8	7.322813000	-4.096893000	-3.567293000
8	2.381223000	6.258251000	-1.165249000
7	2.851404000	1.588192000	0.446673000
8	-2.955327000	5.539685000	6.650311000
8	1.934549000	-6.654882000	4.999501000
8	-2.236790000	3.421773000	6.976300000
8	3.115594000	-4.798571000	4.497577000
6	1.517633000	3.597917000	0.954324000
6	-1.417452000	0.238363000	2.806060000
6	1.143857000	-3.193983000	0.618828000
6	4.490621000	0.115228000	-0.636807000
28	1.478482000	0.177965000	1.040996000
6	0.377021000	3.015186000	1.447901000
6	-0.905223000	-1.030871000	2.519806000
6	2.212538000	-2.564276000	-0.080785000
6	4.081794000	1.362548000	-0.161339000
6	-0.970755000	3.734093000	1.636836000
6	-1.607116000	-2.325115000	2.979580000

6	3.143639000	-3.246416000	-0.950537000
6	4.761597000	2.625953000	-0.387818000
6	-1.655858000	2.788896000	2.677070000
6	-0.595534000	-3.419468000	2.491493000
6	4.149904000	-2.331440000	-1.251476000
6	3.899589000	3.612233000	0.055696000
6	-0.917038000	1.477088000	2.395253000
6	0.302071000	-2.628072000	1.545478000
6	3.788438000	-1.097282000	-0.581999000
6	2.713596000	2.937960000	0.545875000
6	-1.698202000	3.614150000	0.261395000
6	3.071252000	-4.690574000	-1.365789000
6	6.138078000	2.849779000	-0.963876000
6	-3.168688000	4.092190000	0.075703000
6	-3.772830000	-3.748411000	2.691516000
6	2.020514000	-4.977860000	-2.495636000
6	7.237951000	2.984192000	0.109485000
8	-3.749961000	4.726925000	0.996081000
8	-4.627890000	-3.579104000	3.609647000
8	2.476159000	-5.160432000	-3.658198000
6	-2.971899000	-2.489553000	2.247075000
6	5.414517000	-2.617041000	-2.025139000
8	0.812115000	-5.008221000	-2.131853000
6	-0.842257000	5.209032000	2.027099000
6	-1.858080000	-2.280722000	4.500082000
6	5.252713000	-2.848698000	-3.541804000
6	4.126450000	5.102933000	0.034441000
6	-2.561256000	4.402816000	6.250858000
6	2.145011000	-5.610811000	4.329888000
7	2.595621000	-1.267709000	0.112527000
8	6.674194000	-3.067738000	-5.469657000
8	4.333269000	5.862403000	-2.241898000
6	-9.326998000	0.900838000	-4.331070000
6	-8.834249000	2.203308000	-3.689950000
6	-8.288001000	2.047230000	-2.260419000
6	-7.118239000	1.072122000	-2.048197000
6	-5.767355000	1.456819000	-2.658550000
16	-5.572418000	1.117424000	-4.478172000
6	-0.833856000	-1.369118000	-1.992296000
8	-4.044068000	-1.448127000	-3.214435000
16	-1.513711000	0.160643000	-1.243704000
6	-1.777487000	-2.540029000	-2.272404000
8	-3.440361000	-3.752210000	-3.925167000
16	-2.935755000	-2.367118000	-3.673995000
8	-2.160079000	-1.792905000	-4.814022000
6	-8.379400000	-2.576457000	-0.317383000

6	-7.214708000	-1.728977000	0.130945000
6	-5.995048000	-1.770063000	-0.556943000
6	-7.333999000	-0.830913000	1.199327000
6	-4.930792000	-0.946416000	-0.205985000
6	-6.278071000	0.003919000	1.566523000
6	-5.068506000	-0.049541000	0.860921000
8	-4.074467000	0.787285000	1.260102000
6	4.244960000	-1.029035000	6.705453000
6	3.243428000	-1.894432000	5.935763000
6	2.966368000	-1.255654000	4.567173000
7	3.039770000	-2.091310000	3.510487000
8	2.733177000	-0.041572000	4.487344000
1	1.361537000	4.714203000	-4.938287000
1	1.314715000	4.785598000	-3.155470000
1	1.997944000	2.534363000	-6.087106000
1	1.652602000	2.729893000	-1.804817000
1	2.180628000	0.053893000	-5.941110000
1	1.803026000	0.295524000	-1.669697000
1	2.404163000	-1.656530000	-2.884888000
1	-8.038799000	-3.514793000	-0.770764000
1	-8.988589000	-2.058495000	-1.074338000
1	-5.863575000	-2.434777000	-1.406827000
1	-8.268742000	-0.781126000	1.756804000
1	-4.011351000	-0.980188000	-0.777908000
1	-6.365033000	0.693315000	2.401569000
1	-3.228587000	0.552260000	0.830865000
1	-1.677274000	2.252775000	4.774519000
1	-0.500118000	3.485564000	4.390465000
1	-0.465077000	-4.463908000	4.390718000
1	0.832549000	-3.305390000	4.121560000
1	-2.375146000	5.182213000	4.239717000
1	-3.543534000	3.894474000	4.398351000
1	0.551329000	-6.100478000	2.917483000
1	1.740581000	-4.917951000	2.337370000
1	1.493545000	4.663606000	0.748374000
1	-2.352293000	0.270820000	3.356522000
1	1.001392000	-4.245603000	0.398770000
1	-2.719945000	2.685986000	2.441740000
1	-1.143346000	-4.212335000	1.972155000
1	-1.666962000	2.565028000	-0.048556000
1	-1.093931000	4.146596000	-0.487717000
1	4.056833000	-5.021457000	-1.711860000
1	2.794910000	-5.304785000	-0.496127000
1	6.120931000	3.763165000	-1.569628000
1	6.417515000	2.039343000	-1.648134000
1	6.946806000	3.749520000	0.842661000

1	-3.576830000	-1.600544000	2.443587000
1	-2.789878000	-2.541326000	1.165642000
1	5.893789000	-3.520746000	-1.625334000
1	6.140039000	-1.808026000	-1.864417000
1	-0.297640000	5.340347000	2.968953000
1	-1.839444000	5.644612000	2.110814000
1	-0.297131000	5.757598000	1.248346000
1	-2.307030000	-3.212304000	4.856201000
1	-0.934227000	-2.090152000	5.056408000
1	-2.563670000	-1.477477000	4.737101000
1	4.453250000	-3.587618000	-3.701325000
1	4.914996000	-1.930544000	-4.037140000
1	5.203874000	5.303319000	0.083600000
1	3.657911000	5.558843000	0.916343000
1	-8.484177000	0.225665000	-4.509557000
1	-9.805066000	1.096989000	-5.300278000
1	-8.058884000	2.637015000	-4.334867000
1	-9.657348000	2.934044000	-3.666093000
1	-7.984693000	3.038202000	-1.891914000
1	-9.116789000	1.721526000	-1.611903000
1	-6.952902000	0.995339000	-0.966978000
1	-7.392498000	0.061862000	-2.372806000
1	-4.981545000	0.863279000	-2.189463000
1	-5.521333000	2.506674000	-2.470422000
1	-4.846874000	-0.024510000	-4.294585000
1	-0.092560000	-1.716556000	-1.266056000
1	-0.293414000	-1.093349000	-2.900309000
1	-1.158836000	-3.414610000	-2.512685000
1	-2.387198000	-2.783542000	-1.396608000
1	5.162580000	-0.901282000	6.115681000
1	3.829872000	-0.029830000	6.868836000
1	3.581772000	-2.927388000	5.818126000
1	2.286136000	-1.931913000	6.474678000
1	-9.049022000	-2.831296000	0.514782000
1	2.851410000	4.738981000	-3.963298000
1	4.549284000	-1.445852000	7.671586000
6	-1.986381000	1.140478000	-2.708491000
1	-2.426357000	2.065611000	-2.328338000
1	-1.092699000	1.366600000	-3.292983000
1	-2.711674000	0.600387000	-3.309338000
1	3.067065000	-3.108675000	3.679213000
1	2.725107000	-1.740761000	2.605914000
1	-10.051500000	0.362332000	-3.707308000
1	5.432613000	0.098377000	-1.180147000
1	7.299518000	2.047746000	0.685750000
6	8.672847000	3.332029000	-0.392696000

8	8.893302000	3.286394000	-1.634079000
8	9.506983000	3.625353000	0.515353000
8	-3.490421000	-4.840315000	2.119252000
8	-3.664481000	3.801805000	-1.058146000

A-TS$_1$-charged

A-TS$_1$-charged-opt.gjf.log

Temperature 298.150 Kelvin. Pressure 1.00000 Atm.

Zero-point correction= 1.324750 (Hartree/Particle)
Thermal correction to Energy= 1.423339
Thermal correction to Enthalpy= 1.424283
Thermal correction to Gibbs Free Energy= 1.171047
Sum of electronic and zero-point Energies= -5892.260703
Sum of electronic and thermal Energies= -5892.162114
Sum of electronic and thermal Enthalpies= -5892.161169
Sum of electronic and thermal Free Energies= -5892.414405

6	1.164541000	7.450929000	0.122034000
6	1.223451000	6.156314000	-0.645918000
6	2.019945000	6.002942000	-1.789216000
6	0.467445000	5.054602000	-0.227995000
6	2.063132000	4.795192000	-2.487425000
6	0.504653000	3.839686000	-0.902918000
6	1.304482000	3.701580000	-2.045435000
8	1.381727000	2.539127000	-2.744356000
6	-2.728397000	-3.061998000	3.684025000
6	2.514832000	-5.269989000	-0.029738000
6	7.987295000	2.549204000	-2.494766000
6	0.410572000	3.963281000	5.063533000
7	-0.663124000	-0.790725000	1.740695000
6	-4.076572000	-3.188093000	4.407977000
6	3.956288000	-5.425035000	-0.548758000
7	0.872458000	-2.444436000	-0.193378000
8	7.666185000	3.081205000	-3.597042000
8	-0.658351000	3.509672000	5.561427000
7	1.335628000	1.361795000	1.339744000
8	-5.400706000	-4.831982000	5.552020000
8	5.301431000	-7.057919000	0.623463000
8	-3.161847000	-4.908404000	5.841822000
8	5.207291000	-4.948421000	1.422881000
6	-0.615197000	1.379188000	2.856339000
6	-1.170885000	-3.052341000	1.007874000
6	2.821729000	-2.461333000	-1.683692000
6	3.382239000	2.013012000	0.142866000

28	0.979981000	-0.463485000	0.416160000
6	-1.143913000	0.130149000	2.641708000
6	-0.118295000	-3.303150000	0.126247000
6	3.264028000	-1.115648000	-1.538532000
6	2.393381000	2.232506000	1.102158000
6	-2.320760000	-0.425526000	3.469999000
6	0.032039000	-4.642198000	-0.617685000
6	4.338968000	-0.506709000	-2.284219000
6	2.248373000	3.421566000	1.919356000
6	-2.570065000	-1.814842000	2.777595000
6	1.542216000	-4.573177000	-1.018825000
6	4.519251000	0.763181000	-1.751172000
6	1.078749000	3.254400000	2.640788000
6	-1.401065000	-1.913502000	1.788661000
6	1.790572000	-3.071506000	-1.015041000
6	3.545576000	0.902069000	-0.690891000
6	0.548735000	1.955831000	2.276257000
6	-3.545715000	0.529689000	3.353135000
6	5.058180000	-1.126693000	-3.454219000
6	3.105027000	4.661590000	1.892916000
6	-4.863634000	0.077211000	4.036187000
6	-2.346870000	-4.496587000	-1.797205000
6	6.120672000	-2.217648000	-3.078838000
6	3.914878000	4.962840000	3.167952000
8	-4.906516000	0.127838000	5.298909000
8	-2.864325000	-3.420451000	-2.210559000
8	7.285179000	-1.808579000	-2.813254000
6	-0.805710000	-4.631119000	-1.938224000
6	5.489372000	1.812719000	-2.233049000
8	5.722883000	-3.416822000	-3.087921000
6	-1.849088000	-0.534810000	4.936708000
6	-0.351202000	-5.856388000	0.240376000
6	6.960504000	1.582682000	-1.832708000
6	0.394205000	4.265367000	3.524808000
6	-4.218140000	-4.427679000	5.339827000
6	4.909705000	-5.861791000	0.585363000
7	2.777802000	-0.251927000	-0.600975000
8	9.088394000	2.691377000	-1.885634000
8	1.484511000	4.217322000	5.677221000
6	-9.372700000	4.274026000	-3.786234000
6	-8.940752000	4.776860000	-2.399475000
6	-8.685761000	3.660735000	-1.372036000
6	-7.692660000	2.549583000	-1.749883000
6	-6.195255000	2.868060000	-1.689381000
16	-5.577988000	3.848126000	-3.144621000
6	-0.056797000	-0.574799000	-3.045449000

8	-0.166656000	-0.596455000	-6.213333000
16	-0.568598000	0.369586000	-1.533139000
6	-1.220241000	-1.158609000	-3.840304000
8	-1.993156000	-2.288875000	-6.092665000
16	-0.723808000	-1.779625000	-5.475694000
8	0.290581000	-2.854793000	-5.245508000
6	-8.214747000	-0.890003000	-2.745821000
6	-6.901566000	-0.802680000	-2.008554000
6	-5.686025000	-0.839455000	-2.700981000
6	-6.848273000	-0.631964000	-0.614897000
6	-4.464998000	-0.688985000	-2.044024000
6	-5.643731000	-0.454959000	0.063953000
6	-4.437753000	-0.465895000	-0.660630000
8	-3.292250000	-0.262073000	0.030358000
6	3.610166000	-3.343948000	5.331110000
6	3.554477000	-3.639546000	3.829396000
6	3.300200000	-2.390846000	2.984914000
7	3.811452000	-2.434435000	1.736303000
8	2.660198000	-1.437029000	3.447431000
1	0.793634000	8.273588000	-0.510464000
1	0.470390000	7.360855000	0.965854000
1	2.630821000	6.837042000	-2.132987000
1	-0.126478000	5.126710000	0.679292000
1	2.697654000	4.671003000	-3.360992000
1	-0.021801000	2.979038000	-0.507609000
1	0.880895000	1.840911000	-2.249069000
1	-8.052968000	-1.110595000	-3.808000000
1	-8.770876000	0.056701000	-2.696464000
1	-5.683484000	-0.999923000	-3.777824000
1	-7.776501000	-0.625162000	-0.043047000
1	-3.535291000	-0.784470000	-2.586373000
1	-5.620682000	-0.329619000	1.148745000
1	-2.488081000	-0.286257000	-0.546231000
1	-2.598665000	-3.956188000	3.061245000
1	-1.921645000	-3.110189000	4.421662000
1	2.140561000	-6.272798000	0.210746000
1	2.525270000	-4.703847000	0.908615000
1	-4.263675000	-2.294489000	5.017234000
1	-4.895317000	-3.202354000	3.677328000
1	3.969986000	-6.167227000	-1.359013000
1	4.321233000	-4.479879000	-0.960166000
1	-1.081225000	1.982929000	3.629614000
1	-1.898747000	-3.849637000	1.102309000
1	3.424463000	-3.076848000	-2.343867000
1	-3.483722000	-1.729406000	2.175207000
1	1.696055000	-5.006816000	-2.015398000

1	-3.730538000	0.699118000	2.289565000
1	-3.256945000	1.488439000	3.805175000
1	4.324068000	-1.592928000	-4.124713000
1	5.571333000	-0.334200000	-4.012709000
1	2.462370000	5.531338000	1.723978000
1	3.781394000	4.637031000	1.028676000
1	3.252989000	4.841130000	4.038491000
1	-0.427365000	-3.833523000	-2.585890000
1	-0.598343000	-5.587496000	-2.443002000
1	5.167621000	2.805755000	-1.891325000
1	5.465073000	1.862361000	-3.329440000
1	-0.954289000	-1.164053000	5.012800000
1	-2.635325000	-0.939704000	5.576462000
1	-1.587658000	0.460740000	5.312564000
1	-0.037567000	-6.784166000	-0.257346000
1	0.118162000	-5.819258000	1.228988000
1	-1.435534000	-5.904423000	0.350654000
1	7.073593000	1.641676000	-0.741470000
1	7.233857000	0.554182000	-2.112685000
1	-0.656835000	4.358613000	3.217205000
1	0.873561000	5.240617000	3.371789000
1	-8.500912000	3.936917000	-4.358961000
1	-9.859205000	5.068112000	-4.365989000
1	-8.042848000	5.398752000	-2.511820000
1	-9.718671000	5.437026000	-1.989136000
1	-8.365432000	4.113602000	-0.422872000
1	-9.652064000	3.175603000	-1.166357000
1	-7.836308000	1.728941000	-1.038929000
1	-7.935813000	2.135586000	-2.735392000
1	-5.637528000	1.928493000	-1.686442000
1	-5.951645000	3.411788000	-0.771457000
1	-4.301835000	3.372275000	-3.129073000
1	0.610857000	-1.364604000	-2.699717000
1	0.521650000	0.104594000	-3.679355000
1	-1.707996000	-1.984601000	-3.307646000
1	-1.965457000	-0.391577000	-4.067136000
1	4.351771000	-2.567097000	5.552331000
1	2.643857000	-2.999794000	5.709299000
1	4.455848000	-4.150791000	3.478312000
1	2.726479000	-4.332773000	3.618680000
1	-8.893096000	-1.661089000	-2.357411000
1	2.118334000	7.788380000	0.547943000
1	3.908275000	-4.258837000	5.854389000
6	-1.907183000	1.844784000	-2.704331000
1	-2.906389000	1.421796000	-2.712298000
1	-1.810784000	2.741270000	-2.100606000

1	-1.446386000	1.925386000	-3.685909000
1	4.292976000	-3.287340000	1.417221000
1	3.503961000	-1.738330000	1.059009000
1	-10.054764000	3.415611000	-3.741544000
1	4.107241000	2.812165000	0.015076000
1	4.723559000	4.231182000	3.299623000
6	4.525353000	6.396421000	3.227686000
8	3.945330000	7.316752000	2.577436000
8	5.542583000	6.536924000	3.967539000
8	-2.967874000	-5.485453000	-1.309196000
8	-5.813792000	-0.283106000	3.277019000

B-RC-charged

B-RC-charged-opt.gjf.log

Temperature 298.150 Kelvin. Pressure 1.00000 Atm.

Zero-point correction= 1.376994 (Hartree/Particle)
Thermal correction to Energy= 1.476847
Thermal correction to Enthalpy= 1.477791
Thermal correction to Gibbs Free Energy= 1.223115
Sum of electronic and zero-point Energies= -5853.795494
Sum of electronic and thermal Energies= -5853.695641
Sum of electronic and thermal Enthalpies= -5853.694697
Sum of electronic and thermal Free Energies= -5853.949373

6	1.400239000	-1.386597000	-6.504884000
6	1.272522000	-1.771492000	-5.054222000
6	1.235995000	-3.111125000	-4.644925000
6	1.197807000	-0.782451000	-4.063595000
6	1.168994000	-3.458916000	-3.292630000
6	1.091902000	-1.111837000	-2.716315000
6	1.117499000	-2.454591000	-2.318275000
8	1.087516000	-2.836219000	-1.009396000
6	0.317267000	5.357459000	-0.320891000
6	0.498668000	1.708458000	5.044369000
6	4.832950000	-6.938005000	1.266048000
6	4.045006000	0.883910000	-5.388057000
7	0.940536000	2.126416000	-0.465369000
6	-0.166536000	6.696769000	-0.886507000
6	1.675371000	1.051797000	5.775806000
7	0.343763000	0.761719000	2.024780000
8	5.668184000	-6.924067000	2.216437000
8	3.038447000	1.607393000	-5.635747000
7	3.186872000	0.306324000	-0.914787000
8	0.000627000	9.067606000	-0.645997000

8	2.814256000	2.156412000	7.596364000
8	0.698264000	7.803318000	1.093760000
8	3.152302000	2.918388000	5.500210000
6	2.439121000	2.126379000	-2.399136000
6	-0.813035000	2.705540000	1.139483000
6	0.902653000	-1.213350000	3.349985000
6	4.216629000	-1.794234000	-0.157675000
28	1.717458000	0.489059000	0.507550000
6	1.340993000	2.568243000	-1.712301000
6	-0.599189000	1.722714000	2.102111000
6	1.828191000	-1.848539000	2.476257000
6	4.136282000	-0.702221000	-1.025810000
6	0.305035000	3.555367000	-2.273169000
6	-1.451116000	1.589378000	3.385883000
6	2.478114000	-3.109242000	2.743404000
6	4.931197000	-0.506238000	-2.226566000
6	-0.321090000	4.088060000	-0.949250000
6	-0.453006000	0.768656000	4.258974000
6	3.474236000	-3.253845000	1.788736000
6	4.424615000	0.621499000	-2.845166000
6	-0.078824000	2.892269000	-0.033212000
6	0.310174000	0.010270000	3.183406000
6	3.394626000	-2.085852000	0.936855000
6	3.330619000	1.086057000	-2.021056000
6	-0.746281000	2.660511000	-3.028546000
6	2.118164000	-4.063953000	3.848268000
6	6.100260000	-1.330361000	-2.702944000
7	-4.491637000	-0.667203000	3.956272000
6	-2.010346000	3.386352000	-3.462361000
6	-3.552103000	0.278077000	4.240328000
6	0.775365000	-4.829664000	3.574584000
6	7.465033000	-0.782771000	-2.240936000
8	-2.040047000	4.187306000	-4.402384000
8	-3.455304000	0.746601000	5.379590000
8	0.869475000	-6.010156000	3.146655000
6	-2.698630000	0.729225000	3.051711000
6	4.455561000	-4.392601000	1.687848000
7	-3.137956000	3.079948000	-2.755881000
8	-0.284996000	-4.180080000	3.807099000
6	0.883971000	4.600232000	-3.229301000
6	-1.895810000	2.948140000	3.951436000
6	3.876569000	-5.712869000	1.140126000
6	4.894617000	1.264204000	-4.125328000
6	0.227972000	7.967141000	-0.065816000
6	2.631140000	2.127528000	6.352957000
7	2.357732000	-1.257691000	1.361302000

8	4.664180000	-7.867422000	0.425535000
8	4.453758000	-0.111526000	-6.048888000
6	-9.897351000	-2.373292000	-3.713442000
6	-9.074544000	-1.311647000	-4.452898000
6	-8.504980000	-0.194666000	-3.564366000
6	-7.545426000	-0.598298000	-2.432542000
6	-6.224341000	-1.255778000	-2.865492000
16	-6.233661000	-3.109813000	-2.958289000
6	-1.503405000	-2.710597000	0.641615000
8	-4.464880000	-3.074589000	-0.135763000
16	-1.655085000	-1.089189000	-0.231953000
6	-2.517211000	-3.018718000	1.740914000
8	-5.102165000	-2.979816000	2.268092000
16	-4.183879000	-3.598495000	1.246811000
8	-4.167862000	-5.086117000	1.295812000
6	-8.956238000	0.064234000	1.005733000
6	-7.535319000	0.529133000	0.792179000
6	-6.488461000	-0.400330000	0.836062000
6	-7.223649000	1.847772000	0.428483000
6	-5.182977000	-0.044046000	0.510340000
6	-5.916752000	2.225065000	0.106685000
6	-4.891709000	1.271305000	0.142310000
8	-3.618591000	1.673366000	-0.170981000
6	3.968488000	6.541555000	2.450292000
6	3.996897000	5.141875000	3.073788000
6	3.586466000	4.051608000	2.077703000
7	3.099267000	2.925117000	2.637409000
8	3.726799000	4.224340000	0.859151000
1	0.940194000	-2.144644000	-7.155864000
1	0.900488000	-0.430486000	-6.697592000
1	1.282401000	-3.901555000	-5.393602000
1	1.282083000	0.261928000	-4.349851000
1	1.179030000	-4.498432000	-2.975218000
1	1.046542000	-0.335191000	-1.964858000
1	1.297969000	-2.072874000	-0.428486000
1	-8.975944000	-0.868873000	1.580443000
1	-9.440191000	-0.150323000	0.041185000
1	-6.667760000	-1.432974000	1.120820000
1	-8.014989000	2.594439000	0.384569000
1	-4.404270000	-0.795340000	0.513628000
1	-5.676744000	3.249542000	-0.164233000
1	-2.978869000	0.951824000	0.019346000
1	0.077721000	5.355625000	0.748197000
1	1.410799000	5.280420000	-0.367366000
1	-0.096790000	2.272813000	5.775679000
1	0.912387000	2.443065000	4.345757000

1	0.169456000	6.854327000	-1.916778000
1	-1.268241000	6.686902000	-0.942947000
1	1.317975000	0.398216000	6.582024000
1	2.234224000	0.428807000	5.066516000
1	2.604926000	2.524231000	-3.394391000
1	-1.610290000	3.419152000	1.320030000
1	0.661319000	-1.757335000	4.256583000
1	-1.396380000	4.278754000	-1.060721000
1	-0.965731000	0.101773000	4.959003000
1	-1.000940000	1.809243000	-2.388323000
1	-0.265842000	2.262695000	-3.928260000
1	2.921970000	-4.796562000	3.979318000
1	2.001484000	-3.514118000	4.793730000
1	6.091820000	-1.367239000	-3.797964000
1	6.006911000	-2.371752000	-2.370371000
1	7.535583000	0.283294000	-2.503865000
1	-3.352438000	1.292375000	2.375912000
1	-2.382937000	-0.154626000	2.488464000
1	4.866117000	-4.621383000	2.679533000
1	5.317983000	-4.096350000	1.075526000
1	1.667944000	5.185192000	-2.739918000
1	0.103680000	5.275023000	-3.589821000
1	1.325113000	4.110358000	-4.104636000
1	-2.343402000	2.820406000	4.938827000
1	-1.060632000	3.650457000	4.016849000
1	-2.652331000	3.399542000	3.297418000
1	2.956440000	-5.946060000	1.696674000
1	3.577942000	-5.594571000	0.090081000
1	5.933465000	0.971119000	-4.313468000
1	4.869391000	2.356685000	-4.015206000
1	-9.236788000	-3.024326000	-3.130365000
1	-10.448082000	-3.007380000	-4.419540000
1	-8.253258000	-1.812634000	-4.984718000
1	-9.695153000	-0.841184000	-5.229416000
1	-7.986660000	0.529264000	-4.210568000
1	-9.347396000	0.351191000	-3.112321000
1	-7.284651000	0.316077000	-1.888248000
1	-8.052595000	-1.241524000	-1.704880000
1	-5.434795000	-1.002136000	-2.155652000
1	-5.910461000	-0.889690000	-3.848923000
1	-5.612855000	-3.321566000	-1.765821000
1	-0.522557000	-2.679236000	1.123433000
1	-1.462828000	-3.490025000	-0.122097000
1	-2.095643000	-3.794737000	2.392240000
1	-2.675664000	-2.138887000	2.364722000
1	4.641197000	6.590045000	1.588729000

1	2.963838000	6.809771000	2.102305000
1	5.015037000	4.903990000	3.416110000
1	3.364606000	5.077835000	3.966241000
1	-9.605530000	0.780348000	1.526712000
1	2.440444000	-1.246021000	-6.826055000
1	4.289393000	7.276180000	3.196592000
6	-2.360337000	-1.563320000	-1.845732000
1	-2.563739000	-0.634257000	-2.381582000
1	-1.631310000	-2.141452000	-2.412879000
1	-3.278153000	-2.122850000	-1.698162000
1	-3.961869000	3.636098000	-2.944445000
1	-3.123132000	2.559914000	-1.881795000
1	3.033618000	2.817734000	3.662670000
1	2.766480000	2.178169000	2.027977000
1	-4.989972000	-1.044571000	4.751770000
1	-10.621375000	-1.950808000	-3.005425000
1	4.988168000	-2.526154000	-0.383663000
1	7.523951000	-0.808839000	-1.142957000
6	8.732450000	-1.489915000	-2.814003000
8	8.563107000	-2.429818000	-3.638105000
8	9.836522000	-1.033316000	-2.395350000
1	-4.530439000	-1.237314000	3.112308000

B-TS$_1$-charged
B-TS$_1$-charged-opt.gjf.log

Temperature 298.150 Kelvin. Pressure 1.00000 Atm.

Zero-point correction= 1.376782 (Hartree/Particle)
Thermal correction to Energy= 1.476119
Thermal correction to Enthalpy= 1.477064
Thermal correction to Gibbs Free Energy= 1.224715
Sum of electronic and zero-point Energies= -5853.737166
Sum of electronic and thermal Energies= -5853.637829
Sum of electronic and thermal Enthalpies= -5853.636884
Sum of electronic and thermal Free Energies= -5853.889233

6	1.344902000	7.477229000	0.645610000
6	1.294826000	6.223339000	-0.186450000
6	1.968329000	6.109148000	-1.411031000
6	0.530293000	5.130325000	0.237114000
6	1.876550000	4.952097000	-2.186050000
6	0.433710000	3.965339000	-0.515165000
6	1.101701000	3.869477000	-1.744118000
8	1.027664000	2.762878000	-2.528742000
6	-1.887283000	-3.562119000	3.884913000

6	3.024924000	-5.056281000	-0.929201000
6	7.515673000	3.394463000	-3.017967000
6	0.556036000	3.403027000	5.447153000
7	-0.227482000	-1.053996000	1.874322000
6	-3.220962000	-3.917039000	4.555841000
6	4.394450000	-4.916776000	-1.611434000
7	1.176791000	-2.392081000	-0.388007000
8	6.970780000	4.052942000	-3.950364000
8	-0.526964000	2.920339000	5.890032000
7	1.523325000	1.288394000	1.491394000
8	-4.091586000	-5.403250000	6.222210000
8	5.990408000	-6.603300000	-0.944218000
8	-2.502028000	-6.205882000	4.827755000
8	5.769925000	-4.753605000	0.332795000
6	-0.340125000	1.058787000	3.091176000
6	-0.543435000	-3.329402000	1.071063000
6	2.812591000	-2.025946000	-2.172707000
6	3.402019000	2.188194000	0.196964000
28	1.236425000	-0.474495000	0.457354000
6	-0.763736000	-0.217682000	2.820743000
6	0.356566000	-3.381963000	0.009637000
6	3.197287000	-0.684820000	-1.889330000
6	2.505809000	2.239669000	1.267223000
6	-1.912013000	-0.894344000	3.594151000
6	0.467567000	-4.608837000	-0.909190000
6	4.111099000	0.102338000	-2.684226000
6	2.351010000	3.338952000	2.203385000
6	-1.940114000	-2.328823000	2.952824000
6	1.838955000	-4.327518000	-1.608936000
6	4.318599000	1.286221000	-1.992206000
6	1.230601000	3.045003000	2.960475000
6	-0.816370000	-2.254583000	1.920544000
6	1.964840000	-2.816623000	-1.436253000
6	3.512662000	1.204125000	-0.791392000
6	0.757422000	1.753048000	2.511713000
6	-3.226161000	-0.107649000	3.276419000
6	4.662437000	-0.287950000	-4.029635000
6	3.160880000	4.605692000	2.279903000
7	-2.580043000	-5.964875000	-1.602011000
6	-4.482642000	-0.757361000	3.832799000
6	-2.056431000	-4.707099000	-1.526589000
6	5.789316000	-1.378535000	-3.982710000
6	4.017025000	4.775516000	3.548520000
8	-4.837639000	-0.648337000	5.008566000
8	-2.744874000	-3.781778000	-1.083321000
8	6.963472000	-0.968712000	-3.776928000

6	-0.633982000	-4.554201000	-2.024208000
6	5.145827000	2.459662000	-2.449322000
7	-5.218847000	-1.470444000	2.924010000
8	5.419527000	-2.573808000	-4.161824000
6	-1.617486000	-0.863154000	5.105343000
6	0.376059000	-5.944047000	-0.155545000
6	6.671703000	2.269709000	-2.345668000
6	0.537737000	3.924034000	3.968761000
6	-3.259327000	-5.300684000	5.276027000
6	5.491244000	-5.480724000	-0.676809000
7	2.826608000	-0.001823000	-0.767513000
8	8.700124000	3.528551000	-2.593981000
8	1.645789000	3.522206000	6.068539000
6	-9.315452000	5.137034000	-3.511654000
6	-9.084995000	5.659036000	-2.085261000
6	-9.075793000	4.576520000	-0.994263000
6	-8.104894000	3.395426000	-1.155978000
6	-6.604829000	3.672433000	-0.977924000
16	-5.769093000	4.369105000	-2.483495000
6	-0.555374000	-0.346686000	-2.706573000
8	-1.934131000	-0.703318000	-5.606025000
16	-0.646827000	0.548616000	-1.092669000
6	-1.841346000	-1.109850000	-2.986741000
8	-3.258639000	-2.590214000	-4.645175000
16	-1.950802000	-1.848736000	-4.642160000
8	-0.770278000	-2.758235000	-4.783101000
6	-8.298603000	-0.149922000	-3.176407000
6	-7.036703000	-0.404338000	-2.377538000
6	-6.034367000	-1.258205000	-2.854847000
6	-6.920750000	0.054716000	-1.058706000
6	-4.981258000	-1.681103000	-2.044032000
6	-5.857225000	-0.324393000	-0.241296000
6	-4.894113000	-1.221098000	-0.723329000
8	-3.911042000	-1.613238000	0.140820000
6	3.522307000	-3.974811000	4.354951000
6	3.763961000	-3.976009000	2.842524000
6	3.577415000	-2.604829000	2.195169000
7	4.248661000	-2.416684000	1.039455000
8	2.841208000	-1.754024000	2.712169000
1	1.044822000	8.353981000	0.050194000
1	0.636320000	7.405140000	1.479640000
1	2.581832000	6.936827000	-1.764602000
1	0.029213000	5.170388000	1.200920000
1	2.414626000	4.859964000	-3.125574000
1	-0.102007000	3.108858000	-0.124961000
1	0.583286000	2.036962000	-2.008999000

1	-8.130652000	-0.215669000	-4.256327000
1	-8.760093000	0.816027000	-2.945989000
1	-6.068599000	-1.623961000	-3.878781000
1	-7.693001000	0.699147000	-0.649123000
1	-4.239680000	-2.358309000	-2.448067000
1	-5.783557000	0.037749000	0.778869000
1	-3.328937000	-2.289152000	-0.275015000
1	-1.590486000	-4.437441000	3.299067000
1	-1.101585000	-3.437261000	4.640048000
1	2.800834000	-6.129011000	-0.864981000
1	3.106500000	-4.687641000	0.097338000
1	-3.546108000	-3.143653000	5.259145000
1	-4.000732000	-3.948184000	3.776727000
1	4.391187000	-5.460331000	-2.565150000
1	4.615033000	-3.869350000	-1.831320000
1	-0.837032000	1.586971000	3.899761000
1	-1.154888000	-4.210304000	1.226459000
1	3.331154000	-2.488235000	-3.007471000
1	-2.860630000	-2.441995000	2.368775000
1	1.810328000	-4.613498000	-2.667903000
1	-3.305272000	-0.001461000	2.189626000
1	-3.147637000	0.892388000	3.713881000
1	3.849001000	-0.673115000	-4.658768000
1	5.073456000	0.605037000	-4.514919000
1	2.485646000	5.465902000	2.245083000
1	3.797332000	4.708782000	1.392035000
1	3.405757000	4.511901000	4.424812000
1	-0.555694000	-3.615058000	-2.574318000
1	-0.419749000	-5.354299000	-2.743670000
1	4.849969000	3.364468000	-1.902720000
1	4.926387000	2.676001000	-3.502793000
1	-0.670256000	-1.368390000	5.320054000
1	-2.412376000	-1.342433000	5.682141000
1	-1.520112000	0.171466000	5.450856000
1	0.556307000	-6.788952000	-0.833374000
1	1.105128000	-5.995605000	0.656479000
1	-0.613670000	-6.083069000	0.290096000
1	6.976689000	2.178643000	-1.294122000
1	6.933885000	1.313301000	-2.822651000
1	-0.512959000	4.053862000	3.672265000
1	1.010744000	4.913365000	3.953854000
1	-8.377020000	4.762456000	-3.936283000
1	-9.677448000	5.933947000	-4.172072000
1	-8.140827000	6.219760000	-2.063707000
1	-9.871391000	6.381488000	-1.824759000
1	-8.884293000	5.055010000	-0.022905000

1	-10.090275000	4.154950000	-0.932033000
1	-8.373219000	2.667831000	-0.378690000
1	-8.270935000	2.889988000	-2.114481000
1	-6.093852000	2.730986000	-0.761771000
1	-6.437060000	4.350217000	-0.134760000
1	-4.602232000	3.671649000	-2.359560000
1	0.298970000	-1.024019000	-2.650538000
1	-0.365864000	0.379290000	-3.503803000
1	-1.969552000	-1.920596000	-2.268905000
1	-2.711117000	-0.454965000	-2.914879000
1	4.139441000	-3.218817000	4.853844000
1	2.476377000	-3.763593000	4.593597000
1	4.749297000	-4.376728000	2.582424000
1	3.040464000	-4.648174000	2.358163000
1	-8.996284000	-0.945280000	-2.882914000
1	2.312077000	7.728551000	1.099860000
1	3.798274000	-4.958718000	4.749343000
6	-2.365758000	1.920577000	-1.850316000
1	-3.282595000	1.466024000	-1.484451000
1	-2.121436000	2.871499000	-1.386995000
1	-2.273193000	1.922499000	-2.934355000
1	-5.920647000	-2.094932000	3.300379000
1	-4.812268000	-1.715355000	2.025673000
1	4.790575000	-3.193512000	0.629555000
1	3.956513000	-1.633783000	0.457909000
1	-3.456754000	-6.134207000	-1.126170000
1	-10.021654000	4.299334000	-3.571706000
1	4.062108000	3.043909000	0.088774000
1	4.859303000	4.070400000	3.550082000
6	4.574064000	6.215765000	3.769292000
8	3.970075000	7.177280000	3.208251000
8	5.574711000	6.306695000	4.536864000
1	-1.981858000	-6.759690000	-1.774609000

C-RC-charged

C-RC-charged-opt.gjf.log

Temperature 298.150 Kelvin. Pressure 1.00000 Atm.

Zero-point correction= 1.464608 (Hartree/Particle)
Thermal correction to Energy= 1.565068
Thermal correction to Enthalpy= 1.566012
Thermal correction to Gibbs Free Energy= 1.309484
Sum of electronic and zero-point Energies= -5857.902535
Sum of electronic and thermal Energies= -5857.802075
Sum of electronic and thermal Enthalpies= -5857.801131

Sum of electronic and thermal Free Energies= -5858.057659

6	-0.309999000	7.134019000	0.434174000
6	-0.152451000	5.817959000	-0.286954000
6	0.360644000	5.755465000	-1.591909000
6	-0.450035000	4.613224000	0.361218000
6	0.587648000	4.534622000	-2.229589000
6	-0.231436000	3.385807000	-0.258922000
6	0.292635000	3.344367000	-1.554900000
8	0.510245000	2.165474000	-2.212645000
6	0.025559000	-3.587715000	3.357984000
6	3.476750000	-4.392330000	-1.005764000
6	8.056059000	2.825558000	-3.254778000
6	0.146407000	3.437700000	5.238381000
7	-0.155291000	-0.839947000	1.479962000
6	-0.300530000	-4.413730000	4.605208000
6	4.895286000	-3.939309000	-1.379351000
7	1.373227000	-1.970640000	-0.778626000
8	8.600309000	1.999438000	-2.470265000
8	-0.324184000	2.545344000	6.004750000
7	1.863565000	1.422637000	1.391192000
8	0.363719000	-6.331608000	5.863986000
8	6.738784000	-5.351605000	-0.722384000
8	1.266503000	-6.085439000	3.805143000
8	5.823564000	-3.982903000	0.821584000
6	-0.103403000	1.133131000	2.908505000
6	-0.369820000	-3.165040000	0.485773000
6	2.988667000	-1.450304000	-2.539377000
6	3.412082000	2.619790000	-0.049389000
28	1.443225000	-0.169084000	0.191170000
6	-0.379865000	-0.308529000	2.628803000
6	0.596932000	-3.098238000	-0.443416000
6	3.193422000	-0.099043000	-2.304747000
6	2.694174000	2.418511000	1.165365000
6	-1.085331000	-1.202169000	3.651392000
6	0.924729000	-4.275927000	-1.386422000
6	3.874949000	0.838367000	-3.286305000
6	2.814463000	3.403884000	2.337367000
6	-1.055842000	-2.586990000	2.913818000
6	2.330949000	-3.838138000	-1.888785000
6	4.240910000	2.048209000	-2.391929000
6	1.573723000	3.022498000	3.169140000
6	-0.880795000	-2.128675000	1.427567000
6	2.237774000	-2.328012000	-1.739649000
6	3.454252000	1.762699000	-1.118275000
6	1.356866000	1.551106000	2.771499000

6	-2.542164000	-0.636863000	3.811450000
6	2.894819000	1.191763000	-4.436247000
6	2.882368000	4.878097000	1.884324000
7	-0.907523000	-5.274921000	-4.591123000
6	-3.480692000	-1.613438000	4.501773000
6	0.100298000	-5.270791000	-3.668708000
6	3.472559000	2.286951000	-5.375173000
6	2.688828000	5.922066000	2.991600000
6	3.857633000	6.014543000	4.019154000
8	-3.445181000	-1.862115000	5.706558000
8	1.045395000	-6.061927000	-3.739817000
8	4.369578000	1.913773000	-6.180992000
6	-0.078787000	-4.228686000	-2.563553000
6	5.762239000	2.206579000	-2.162722000
7	-4.391329000	-2.207550000	3.668965000
8	3.010679000	3.456838000	-5.231164000
8	3.543789000	6.362167000	5.193638000
6	-0.364101000	-1.143092000	5.012015000
6	0.853147000	-5.639754000	-0.682497000
6	6.502363000	2.783120000	-3.375483000
6	1.591414000	3.246936000	4.694061000
6	0.531575000	-5.727195000	4.766930000
6	5.921135000	-4.475448000	-0.350186000
7	2.831191000	0.537075000	-1.160916000
8	8.639770000	3.674872000	-3.988534000
8	-0.452817000	4.475151000	4.834973000
6	-10.977925000	3.261522000	-2.324397000
6	-10.536112000	3.800328000	-0.954037000
6	-10.166343000	2.703540000	0.061130000
6	-9.104734000	1.672234000	-0.356713000
6	-7.643853000	2.136395000	-0.391572000
16	-7.237136000	3.136005000	-1.903605000
6	-1.367077000	-0.369638000	-2.401356000
8	-2.437751000	-0.535017000	-5.561231000
16	-2.080116000	0.442166000	-0.918253000
6	-2.321911000	-1.359323000	-3.057988000
8	-2.628332000	-2.983671000	-5.101620000
16	-1.901393000	-1.698514000	-4.799795000
8	-0.420241000	-1.842139000	-4.860519000
6	-9.336560000	-1.823268000	-1.581515000
6	-7.996896000	-1.605262000	-0.930217000
6	-6.863456000	-1.327958000	-1.702529000
6	-7.850694000	-1.623131000	0.464791000
6	-5.622733000	-1.086020000	-1.114072000
6	-6.622160000	-1.373582000	1.071893000
6	-5.495214000	-1.113395000	0.279060000

8	-4.304119000	-0.915432000	0.909397000
6	3.500278000	-3.557125000	4.997841000
6	3.732376000	-3.568689000	3.486332000
6	3.434310000	-2.211533000	2.856011000
7	4.019965000	-1.983216000	1.660967000
8	2.688116000	-1.397417000	3.422275000
1	-0.773633000	7.897673000	-0.204606000
1	-0.926808000	7.024512000	1.332695000
1	0.614192000	6.678814000	-2.110292000
1	-0.826433000	4.632961000	1.381756000
1	1.037183000	4.472473000	-3.217857000
1	-0.418919000	2.456197000	0.259725000
1	0.763068000	1.466111000	-1.571165000
1	-9.225850000	-2.036503000	-2.650559000
1	-9.970227000	-0.929962000	-1.496990000
1	-6.943087000	-1.305408000	-2.786870000
1	-8.717077000	-1.831361000	1.089690000
1	-4.751385000	-0.892519000	-1.726151000
1	-6.515380000	-1.381554000	2.152184000
1	-3.600619000	-0.652181000	0.260462000
1	0.182615000	-4.305091000	2.548601000
1	0.979086000	-3.070223000	3.473200000
1	3.442999000	-5.488387000	-1.050806000
1	3.285520000	-4.111616000	0.035727000
1	-0.181164000	-3.836246000	5.528346000
1	-1.362583000	-4.711606000	4.588705000
1	5.152203000	-4.291469000	-2.386545000
1	4.939604000	-2.843913000	-1.385816000
1	-0.705933000	1.729106000	2.210045000
1	-0.429928000	1.389594000	3.917598000
1	-0.915192000	-4.103263000	0.554352000
1	3.477335000	-1.880736000	-3.408284000
1	4.766815000	0.379548000	-3.728294000
1	3.731516000	3.173274000	2.903431000
1	-2.024011000	-3.092814000	3.005079000
1	2.502373000	-4.140130000	-2.925886000
1	3.862184000	2.973463000	-2.843327000
1	0.737233000	3.607900000	2.767084000
1	-1.888249000	-1.858838000	1.064450000
1	1.960859000	0.886477000	3.402669000
1	-2.950271000	-0.365577000	2.833595000
1	-2.493996000	0.273102000	4.418235000
1	2.699881000	0.277556000	-5.012417000
1	1.954338000	1.536291000	-3.999854000
1	2.123736000	5.026381000	1.106472000
1	3.861618000	5.063281000	1.429253000

1	1.739222000	5.774297000	3.517327000
1	2.620988000	6.911985000	2.507962000
1	-1.099937000	-4.321098000	-2.170848000
1	-0.027115000	-3.249607000	-3.055643000
1	6.201472000	1.235756000	-1.900607000
1	5.930750000	2.857785000	-1.294436000
1	0.667210000	-1.488858000	4.914680000
1	-0.893660000	-1.757637000	5.743396000
1	-0.342013000	-0.114419000	5.391506000
1	1.256709000	-6.421712000	-1.329203000
1	1.394814000	-5.635914000	0.267234000
1	-0.190022000	-5.898210000	-0.462252000
1	6.244579000	2.206266000	-4.276228000
1	6.142278000	3.800257000	-3.582635000
1	2.161425000	4.149109000	4.936319000
1	2.076956000	2.397457000	5.192058000
1	-10.110629000	2.988490000	-2.936170000
1	-11.554791000	4.007163000	-2.883114000
1	-9.694741000	4.491520000	-1.089929000
1	-11.349249000	4.395152000	-0.515268000
1	-9.847796000	3.176702000	1.000921000
1	-11.085104000	2.145940000	0.296354000
1	-9.134881000	0.851373000	0.370234000
1	-9.365257000	1.223125000	-1.322202000
1	-6.993302000	1.259641000	-0.410086000
1	-7.405233000	2.727570000	0.498138000
1	-5.903528000	2.927726000	-1.880430000
1	-0.454459000	-0.862384000	-2.055328000
1	-1.071504000	0.435204000	-3.078804000
1	-2.326468000	-2.309912000	-2.520257000
1	-3.344944000	-0.980108000	-3.106413000
1	3.977966000	-2.687194000	5.463940000
1	2.434349000	-3.518681000	5.232287000
1	4.749096000	-3.879430000	3.219902000
1	3.054897000	-4.308253000	3.037214000
1	-9.908149000	-2.650057000	-1.139572000
1	0.660299000	7.532781000	0.757148000
1	3.923045000	-4.457974000	5.455466000
6	-3.100225000	1.713528000	-1.735994000
1	-3.805303000	1.277060000	-2.445305000
1	-3.650872000	2.238644000	-0.955254000
1	-2.446656000	2.418948000	-2.248577000
1	-4.893800000	-3.011105000	4.022523000
1	-4.296957000	-2.105094000	2.665267000
1	4.644736000	-2.688259000	1.228478000
1	3.695858000	-1.186107000	1.120916000

1	-0.715126000	-5.809151000	-5.429554000
1	-11.585812000	2.353244000	-2.226974000
8	5.017011000	5.776353000	3.580291000
1	3.982163000	3.537673000	-0.135576000
1	-1.516886000	-4.457405000	-4.707741000

C-TS$_1$-charged

C-TS$_1$-charged-opt.gjf.log

Temperature 298.150 Kelvin. Pressure 1.00000 Atm.

Zero-point correction= 1.460665 (Hartree/Particle)
Thermal correction to Energy= 1.561390
Thermal correction to Enthalpy= 1.562334
Thermal correction to Gibbs Free Energy= 1.306963
Sum of electronic and zero-point Energies= -5857.868187
Sum of electronic and thermal Energies= -5857.767463
Sum of electronic and thermal Enthalpies= -5857.766519
Sum of electronic and thermal Free Energies= -5858.021890

6	-0.300294000	7.512662000	1.676376000
6	-0.138618000	6.386761000	0.691969000
6	0.740109000	6.458767000	-0.400449000
6	-0.897958000	5.220255000	0.839766000
6	0.868737000	5.407875000	-1.309365000
6	-0.775021000	4.150548000	-0.042311000
6	0.114487000	4.242465000	-1.120004000
8	0.272916000	3.224313000	-2.008620000
6	0.551023000	-4.467587000	2.530111000
6	3.953519000	-3.663833000	-2.086464000
6	7.371288000	4.275725000	-1.207485000
6	0.157821000	0.749701000	6.451975000
7	-0.395038000	-1.640719000	1.234836000
6	0.526372000	-5.588688000	3.573168000
6	5.225717000	-2.902155000	-2.489943000
7	1.355487000	-1.864106000	-1.191684000
8	7.582785000	4.061821000	0.019006000
8	0.626866000	0.452802000	7.582633000
7	1.047442000	0.961119000	1.777652000
8	1.799906000	-7.396124000	4.475417000
8	7.242662000	-4.193416000	-2.208721000
8	2.546275000	-6.469944000	2.552698000
8	6.468944000	-2.995414000	-0.455977000
6	-0.794577000	-0.066691000	3.052479000
6	-0.013887000	-3.675782000	-0.236847000
6	2.886055000	-0.617426000	-2.639189000

6	2.430025000	2.706872000	0.810868000
28	0.895723000	-0.319655000	0.112464000
6	-0.773473000	-1.431207000	2.444485000
6	0.856125000	-3.179270000	-1.126991000
6	2.906149000	0.622954000	-2.017724000
6	1.704514000	2.088092000	1.872784000
6	-1.277031000	-2.671707000	3.188697000
6	1.380921000	-3.996006000	-2.316382000
6	3.640706000	1.820126000	-2.594887000
6	1.713290000	2.678830000	3.285903000
6	-0.805095000	-3.804570000	2.215567000
6	2.632074000	-3.157493000	-2.719814000
6	3.663973000	2.807999000	-1.405080000
6	0.729620000	1.759090000	4.051865000
6	-0.767582000	-3.021879000	0.867651000
6	2.266397000	-1.787020000	-2.163943000
6	2.744587000	2.139535000	-0.394217000
6	0.608139000	0.534080000	3.118425000
6	-2.840094000	-2.527470000	3.237874000
6	2.933766000	2.372957000	-3.856377000
6	1.365578000	4.179124000	3.328246000
7	-1.170516000	-5.804865000	-3.626530000
6	-3.560425000	-3.845754000	3.453156000
6	-0.999109000	-4.495369000	-3.248010000
6	3.674656000	3.629138000	-4.399690000
6	1.290855000	4.804542000	4.729939000
8	-3.525473000	-4.484464000	4.504442000
8	-1.926694000	-3.861139000	-2.747599000
8	4.688033000	3.404642000	-5.116295000
6	0.377390000	-3.912790000	-3.517638000
6	5.081542000	3.071577000	-0.844785000
7	-4.285132000	-4.279586000	2.374556000
8	3.216040000	4.757064000	-4.053720000
6	-0.730008000	-2.748107000	4.623726000
6	1.668184000	-5.462652000	-1.967036000
6	5.963757000	3.913652000	-1.771717000
6	1.181588000	1.412109000	5.477606000
6	1.746387000	-6.565403000	3.525782000
6	6.431149000	-3.408973000	-1.661305000
7	2.338537000	0.890753000	-0.817036000
8	8.177050000	4.782461000	-2.040456000
8	-1.035844000	0.586844000	6.060005000
6	-10.653479000	3.206058000	-1.589223000
6	-10.107278000	3.473814000	-0.177322000
6	-9.536603000	2.231508000	0.527821000
6	-8.449072000	1.427843000	-0.204138000

6	-7.027307000	2.001105000	-0.232238000
16	-6.793253000	3.348302000	-1.488828000
6	-0.699208000	0.272204000	-2.960848000
8	-1.449483000	0.556259000	-6.047636000
16	-1.115887000	0.559047000	-1.178521000
6	-1.685665000	-0.624764000	-3.691008000
8	-2.304830000	-1.789952000	-5.968990000
16	-1.293951000	-0.809795000	-5.460059000
8	0.110548000	-1.324172000	-5.522065000
6	-8.484744000	-1.713963000	-2.018643000
6	-7.113114000	-1.567784000	-1.405017000
6	-5.995080000	-1.249894000	-2.185369000
6	-6.916784000	-1.721800000	-0.023917000
6	-4.733526000	-1.071383000	-1.618312000
6	-5.669572000	-1.520014000	0.564560000
6	-4.564636000	-1.177674000	-0.232525000
8	-3.374379000	-0.979885000	0.385275000
6	4.762973000	-3.440091000	3.690842000
6	4.410858000	-3.468942000	2.194852000
6	3.718025000	-2.194008000	1.736614000
7	4.273706000	-1.573544000	0.673913000
8	2.707825000	-1.770540000	2.321137000
1	-0.877377000	8.343736000	1.239795000
1	-0.826829000	7.175766000	2.575203000
1	1.348609000	7.351796000	-0.534517000
1	-1.583784000	5.136840000	1.680434000
1	1.560839000	5.444299000	-2.148265000
1	-1.336089000	3.235424000	0.113886000
1	-0.078893000	2.393366000	-1.606448000
1	-8.439007000	-1.656295000	-3.111540000
1	-9.167788000	-0.927443000	-1.675401000
1	-6.103659000	-1.142007000	-3.262374000
1	-7.761574000	-1.986848000	0.609635000
1	-3.876393000	-0.851009000	-2.238643000
1	-5.537621000	-1.616475000	1.637858000
1	-2.673732000	-0.640459000	-0.237357000
1	0.921461000	-4.922834000	1.606931000
1	1.295317000	-3.703709000	2.779477000
1	4.094085000	-4.714813000	-2.366791000
1	3.853326000	-3.635407000	-0.996432000
1	0.452675000	-5.208077000	4.597525000
1	-0.376969000	-6.205767000	3.431901000
1	5.415078000	-3.039121000	-3.562537000
1	5.096122000	-1.832864000	-2.300212000
1	-1.423504000	0.585858000	2.431340000
1	-1.202187000	-0.076215000	4.064436000

1	-0.325429000	-4.705774000	-0.387348000
1	3.467176000	-0.713652000	-3.549729000
1	4.656785000	1.528128000	-2.887833000
1	2.731159000	2.548163000	3.689982000
1	-1.557762000	-4.599552000	2.145861000
1	2.756811000	-3.120498000	-3.809160000
1	3.229936000	3.766357000	-1.708003000
1	-0.248929000	2.255940000	4.106750000
1	-1.807410000	-2.935355000	0.504183000
1	1.306467000	-0.248665000	3.439964000
1	-3.191380000	-2.050436000	2.321445000
1	-3.095594000	-1.863966000	4.070840000
1	2.933555000	1.589644000	-4.625023000
1	1.901770000	2.620892000	-3.600516000
1	0.403299000	4.321378000	2.825319000
1	2.098699000	4.753671000	2.750837000
1	0.503859000	4.324455000	5.322268000
1	0.247028000	-2.871533000	-3.820381000
1	0.834779000	-4.432160000	-4.369749000
1	5.568677000	2.107579000	-0.635124000
1	5.008785000	3.581514000	0.121862000
1	0.360000000	-2.829024000	4.628455000
1	-1.152044000	-3.619823000	5.130487000
1	-0.999710000	-1.852346000	5.193763000
1	2.139517000	-5.978502000	-2.813592000
1	2.325116000	-5.549367000	-1.098385000
1	0.745216000	-5.999054000	-1.726776000
1	6.086299000	3.431484000	-2.749207000
1	5.441251000	4.854569000	-2.001888000
1	1.531747000	2.323016000	5.979676000
1	2.063383000	0.756247000	5.429162000
1	-9.836198000	3.183666000	-2.319682000
1	-11.351203000	3.991307000	-1.902884000
1	-9.343987000	4.260828000	-0.233671000
1	-10.909890000	3.874797000	0.457412000
1	-9.155672000	2.523019000	1.517062000
1	-10.375269000	1.544223000	0.715016000
1	-8.361826000	0.461422000	0.305203000
1	-8.766814000	1.199278000	-1.228029000
1	-6.330363000	1.202444000	-0.498210000
1	-6.746957000	2.384692000	0.753970000
1	-5.463396000	3.145512000	-1.683596000
1	0.298002000	-0.165936000	-2.981120000
1	-0.649818000	1.248083000	-3.451612000
1	-1.720533000	-1.628470000	-3.259792000
1	-2.691029000	-0.197600000	-3.681645000

1	5.417326000	-2.594415000	3.935813000
1	3.860497000	-3.369969000	4.304933000
1	5.303570000	-3.649567000	1.587272000
1	3.713451000	-4.301408000	2.026110000
1	-8.949715000	-2.673099000	-1.755019000
1	0.640894000	7.933163000	2.053870000
1	5.293854000	-4.368008000	3.929255000
6	-2.986101000	1.955929000	-1.727679000
1	-3.452000000	2.053853000	-0.752891000
1	-2.482268000	2.843415000	-2.091419000
1	-3.579260000	1.424241000	-2.461643000
1	-4.647452000	-5.223641000	2.398002000
1	-4.183853000	-3.842231000	1.468642000
1	5.073642000	-1.998490000	0.174049000
1	3.762063000	-0.802651000	0.256100000
1	-2.004422000	-6.271996000	-3.294510000
1	-11.163985000	2.238711000	-1.677316000
1	2.841561000	3.689835000	1.007896000
1	2.249135000	4.640650000	5.245674000
6	1.019294000	6.336614000	4.643601000
8	1.947623000	7.029430000	4.139091000
8	-0.102097000	6.744630000	5.061303000
1	-0.368701000	-6.386515000	-3.824033000

D-RC-charged
D-RC-charged-opt.gjf.log

Temperature   298.150 Kelvin. Pressure   1.00000 Atm.

Zero-point correction=   1.464971 (Hartree/Particle)
Thermal correction to Energy=   1.565229
Thermal correction to Enthalpy=   1.566173
Thermal correction to Gibbs Free Energy=   1.308140
Sum of electronic and zero-point Energies=   -5857.920261
Sum of electronic and thermal Energies=   -5857.820002
Sum of electronic and thermal Enthalpies=   -5857.819058
Sum of electronic and thermal Free Energies=   -5858.077092

6	-0.107820000	6.444757000	3.482145000
6	0.065757000	5.590663000	2.251615000
6	0.814602000	6.040205000	1.152578000
6	-0.462843000	4.296461000	2.197523000
6	1.045323000	5.226011000	0.043953000
6	-0.233223000	3.463172000	1.103606000
6	0.526393000	3.926068000	0.024295000
8	0.786357000	3.161030000	-1.077496000

6	0.578466000	-4.757549000	2.125175000
6	2.921408000	-3.637595000	-3.149568000
6	8.280618000	2.638219000	-1.239618000
6	-0.909134000	1.071749000	5.783444000
7	-0.415734000	-1.764878000	0.939581000
6	0.486169000	-5.863571000	3.186912000
6	4.345116000	-3.130670000	-3.421247000
7	0.881370000	-1.491527000	-1.709208000
8	8.549345000	1.495435000	-0.775133000
8	-1.364361000	-0.036589000	6.193689000
7	1.389191000	0.516597000	1.802692000
8	1.451166000	-7.926450000	3.898824000
8	6.061738000	-4.814715000	-3.638095000
8	1.911270000	-7.250971000	1.790106000
8	5.405011000	-4.188759000	-1.568590000
6	-0.733038000	-0.292842000	2.834337000
6	0.155898000	-3.611532000	-0.640951000
6	2.633836000	-0.369564000	-2.982618000
6	3.177623000	2.036057000	1.146615000
28	1.065047000	-0.410906000	0.012845000
6	-0.753045000	-1.628984000	2.170363000
6	0.010237000	-2.677486000	-1.865144000
6	2.988977000	0.642015000	-2.112171000
6	2.275016000	1.456276000	2.078153000
6	-1.186041000	-2.915048000	2.877563000
6	0.361996000	-3.282840000	-3.292241000
6	3.916441000	1.784616000	-2.491729000
6	2.251111000	1.894721000	3.548073000
6	-0.729363000	-4.002950000	1.835894000
6	1.764052000	-2.698385000	-3.570124000
6	4.300831000	2.375161000	-1.113669000
6	0.857191000	1.387055000	3.965949000
6	-0.704546000	-3.155890000	0.532027000
6	1.744242000	-1.437346000	-2.713068000
6	3.308818000	1.698378000	-0.178075000
6	0.710926000	0.155691000	3.067046000
6	-2.750694000	-2.812638000	2.982344000
6	3.189574000	2.791658000	-3.417512000
6	2.479304000	3.393471000	3.847747000
7	-1.392110000	-2.289160000	-2.046226000
6	-3.425050000	-4.125652000	3.335675000
6	-1.867485000	-2.304294000	-3.313294000
6	4.081304000	4.025979000	-3.728251000
6	3.888283000	3.800049000	4.285653000
8	-3.422998000	-4.614590000	4.462950000
8	-3.028613000	-2.049756000	-3.645106000

8	5.027862000	3.834664000	-4.541775000
6	-0.705928000	-2.649871000	-4.219297000
6	5.774020000	2.112028000	-0.720488000
7	-4.085615000	-4.736647000	2.293783000
8	3.795462000	5.098135000	-3.121282000
6	-0.578717000	-3.033346000	4.285099000
6	0.239421000	-4.807624000	-3.378779000
6	6.779167000	2.978444000	-1.487275000
6	0.612489000	1.149965000	5.459271000
6	1.379075000	-7.122895000	2.927368000
6	5.375656000	-4.130541000	-2.840788000
7	2.581434000	0.722088000	-0.813732000
8	9.105356000	3.542922000	-1.558137000
8	-1.564794000	2.130125000	5.577426000
6	-10.716725000	3.481081000	-0.399874000
6	-10.097826000	3.338092000	0.998655000
6	-9.420548000	1.978785000	1.252813000
6	-8.290575000	1.546348000	0.301895000
6	-6.941379000	2.261570000	0.442580000
16	-6.929114000	3.950896000	-0.331921000
6	-1.268224000	1.256824000	-2.923135000
8	-1.299547000	2.705484000	-5.755498000
16	-1.791892000	1.203892000	-1.164748000
6	-2.416896000	1.161311000	-3.917832000
8	-3.097160000	1.114867000	-6.450692000
16	-1.855627000	1.324451000	-5.643753000
8	-0.832178000	0.251318000	-5.835610000
6	-8.825913000	-1.334737000	-1.926280000
6	-7.397812000	-1.352481000	-1.450224000
6	-6.440790000	-0.537391000	-2.068981000
6	-6.995919000	-2.106577000	-0.339751000
6	-5.131467000	-0.462861000	-1.600644000
6	-5.691425000	-2.038826000	0.150290000
6	-4.753662000	-1.210083000	-0.477555000
8	-3.486354000	-1.174667000	0.029644000
6	4.368308000	-4.929054000	2.980653000
6	4.135777000	-4.523406000	1.525074000
6	3.678595000	-3.075003000	1.378272000
7	3.850943000	-2.548086000	0.146565000
8	3.162080000	-2.462840000	2.326182000
1	-0.654633000	7.373135000	3.258898000
1	-0.670305000	5.908020000	4.254133000
1	1.254112000	7.035824000	1.178951000
1	-1.019686000	3.910215000	3.048880000
1	1.661767000	5.548025000	-0.791740000
1	-0.586794000	2.439496000	1.102422000

1	0.751711000	2.212371000	-0.843841000
1	-8.887787000	-1.003056000	-2.969395000
1	-9.426744000	-0.627943000	-1.335976000
1	-6.720259000	0.051757000	-2.939651000
1	-7.716808000	-2.750550000	0.160403000
1	-4.397704000	0.148888000	-2.109992000
1	-5.393132000	-2.619945000	1.017568000
1	-2.983143000	-0.421684000	-0.375092000
1	0.879461000	-5.266196000	1.204261000
1	1.382761000	-4.054486000	2.359686000
1	2.801510000	-4.589729000	-3.683045000
1	2.832580000	-3.861788000	-2.081322000
1	0.727983000	-5.495840000	4.189399000
1	-0.550995000	-6.230829000	3.260041000
1	4.500207000	-3.002840000	-4.500348000
1	4.488431000	-2.156724000	-2.941719000
1	-1.231408000	0.442607000	2.191790000
1	-1.254350000	-0.332712000	3.791982000
1	1.212201000	-3.638643000	-0.361613000
1	-0.156184000	-4.627934000	-0.898995000
1	3.130290000	-0.384428000	-3.947785000
1	4.797577000	1.414196000	-3.028333000
1	3.012676000	1.296700000	4.080467000
1	-1.506579000	-4.769352000	1.729673000
1	1.886451000	-2.454028000	-4.633543000
1	4.125936000	3.457741000	-1.105950000
1	0.129733000	2.135792000	3.636165000
1	-1.743587000	-3.141873000	0.174609000
1	1.265599000	-0.685843000	3.514216000
1	-3.152015000	-2.419346000	2.043943000
1	-2.992167000	-2.093299000	3.771876000
1	2.938345000	2.275797000	-4.353916000
1	2.263903000	3.111487000	-2.933565000
1	1.797380000	3.692944000	4.651896000
1	2.173572000	3.986474000	2.980990000
1	4.243906000	3.111087000	5.069209000
1	-0.378188000	-1.706710000	-4.674640000
1	-1.023772000	-3.310062000	-5.032265000
1	6.015633000	1.053534000	-0.880276000
1	5.893851000	2.287599000	0.357112000
1	0.512070000	-3.086191000	4.232859000
1	-0.949728000	-3.934756000	4.778532000
1	-0.847138000	-2.169417000	4.904198000
1	0.484225000	-5.144351000	-4.393406000
1	0.899298000	-5.331408000	-2.683932000
1	-0.791440000	-5.117206000	-3.164675000

1	6.586680000	2.909765000	-2.568286000
1	6.620108000	4.038438000	-1.245108000
1	1.028658000	2.000828000	6.016576000
1	1.129417000	0.241004000	5.793084000
1	-9.941082000	3.621398000	-1.161521000
1	-11.391536000	4.343294000	-0.452188000
1	-9.383822000	4.154248000	1.165767000
1	-10.884268000	3.462681000	1.756365000
1	-9.035472000	1.960274000	2.282299000
1	-10.200010000	1.203615000	1.205618000
1	-8.080774000	0.488021000	0.497611000
1	-8.622215000	1.591800000	-0.742823000
1	-6.175936000	1.673221000	-0.068665000
1	-6.661883000	2.355135000	1.496505000
1	-5.587453000	4.076481000	-0.416380000
1	-0.578738000	0.415509000	-3.028391000
1	-0.698907000	2.181725000	-3.055495000
1	-2.919693000	0.195243000	-3.849805000
1	-3.147609000	1.963094000	-3.774242000
1	5.020628000	-4.214889000	3.496769000
1	3.425717000	-4.989081000	3.530431000
1	5.025660000	-4.685266000	0.906096000
1	3.352140000	-5.171772000	1.110530000
1	-9.340048000	-2.302891000	-1.863447000
1	0.858873000	6.725566000	3.919829000
1	4.850081000	-5.912507000	3.001869000
6	-2.799468000	2.719530000	-1.054478000
1	-3.033004000	2.869044000	-0.001086000
1	-2.212222000	3.568106000	-1.404990000
1	-3.726016000	2.639475000	-1.623788000
1	-4.357952000	-5.701528000	2.437079000
1	-3.867734000	-4.474933000	1.341305000
1	4.374218000	-3.067170000	-0.580066000
1	3.464056000	-1.629311000	-0.049435000
1	-1.975766000	-1.955619000	-1.291105000
1	-11.280416000	2.583092000	-0.682555000
1	3.815287000	2.831630000	1.515792000
1	4.613684000	3.697694000	3.466900000
6	4.019280000	5.247732000	4.863728000
8	2.956757000	5.898369000	5.078894000
8	5.200192000	5.627641000	5.096499000

D-TS$_1$-charged

D-TS$_1$-charged-opt.gjf.log

Temperature 298.150 Kelvin. Pressure 1.00000 Atm.

Zero-point correction= 1.462582 (Hartree/Particle)
Thermal correction to Energy= 1.562143
Thermal correction to Enthalpy= 1.563087
Thermal correction to Gibbs Free Energy= 1.309537
Sum of electronic and zero-point Energies= -5857.894988
Sum of electronic and thermal Energies= -5857.795428
Sum of electronic and thermal Enthalpies= -5857.794483
Sum of electronic and thermal Free Energies= -5858.048034

6	0.633605000	-6.817162000	3.555002000
6	0.359891000	-5.995266000	2.322467000
6	-0.472223000	-6.461162000	1.294107000
6	0.928502000	-4.725005000	2.176752000
6	-0.736080000	-5.687903000	0.164001000
6	0.672398000	-3.936814000	1.055738000
6	-0.164167000	-4.414327000	0.038381000
8	-0.446305000	-3.691218000	-1.080653000
6	-0.617241000	4.472745000	2.742317000
6	-3.215672000	3.952032000	-2.522556000
6	-7.908287000	-2.984209000	-1.556588000
6	1.167988000	-1.677796000	5.571164000
7	0.451646000	1.752645000	1.108693000
6	-0.527320000	5.446672000	3.925006000
6	-4.597913000	3.377562000	-2.868006000
7	-0.927321000	1.808197000	-1.550801000
8	-8.265851000	-1.950418000	-0.926674000
8	1.645857000	-0.593638000	6.019050000
7	-1.134815000	-0.764563000	1.655371000
8	-1.699181000	7.217998000	5.017933000
8	-6.409988000	4.959376000	-2.678657000
8	-2.243349000	6.796824000	2.863648000
8	-5.730149000	3.854002000	-0.828497000
6	0.930463000	0.050556000	2.766892000
6	-0.291862000	3.769640000	-0.173307000
6	-2.640854000	0.740663000	-2.910344000
6	-2.826752000	-2.296717000	0.811135000
28	-0.802080000	0.360870000	-0.077916000
6	0.863812000	1.463017000	2.287793000
6	-0.150735000	3.064665000	-1.541875000
6	-2.840391000	-0.433059000	-2.211895000
6	-1.976599000	-1.763432000	1.818014000
6	1.294513000	2.663596000	3.131566000
6	-0.634906000	3.861357000	-2.828085000
6	-3.652220000	-1.592336000	-2.748502000
6	-1.967101000	-2.312647000	3.249631000
6	0.714277000	3.854583000	2.282216000

6	-2.003413000	3.210031000	-3.138361000
6	-3.965585000	-2.398906000	-1.465122000
6	-0.589101000	-1.807640000	3.726854000
6	0.655381000	3.196713000	0.875068000
6	-1.840733000	1.835796000	-2.501948000
6	-2.994625000	-1.797006000	-0.455991000
6	-0.489116000	-0.488630000	2.955567000
6	2.865056000	2.621978000	3.101925000
6	-2.848761000	-2.400003000	-3.791385000
6	-2.183109000	-3.827091000	3.440857000
7	1.257671000	2.812954000	-1.845573000
6	3.514435000	3.919100000	3.549439000
6	1.642557000	3.030796000	-3.128529000
6	-3.746050000	-3.414349000	-4.569882000
6	-3.603581000	-4.285554000	3.785307000
8	3.581768000	4.279094000	4.722239000
8	2.778019000	2.852912000	-3.571778000
8	-4.813089000	-2.940547000	-5.053389000
6	0.418373000	3.476740000	-3.897511000
6	-5.442780000	-2.302595000	-1.014220000
7	4.063406000	4.670878000	2.537011000
8	-3.313449000	-4.593278000	-4.666524000
6	0.796982000	2.566829000	4.582386000
6	-0.642789000	5.383792000	-2.662232000
6	-6.390913000	-3.143428000	-1.875582000
6	-0.353368000	-1.712374000	5.237811000
6	-1.603267000	6.583671000	3.930736000
6	-5.690297000	4.130776000	-2.071556000
7	-2.372293000	-0.678788000	-0.955618000
8	-8.655025000	-3.908302000	-1.990398000
8	1.803326000	-2.742007000	5.333203000
6	10.642616000	-3.145776000	-1.213969000
6	10.139345000	-3.068193000	0.236097000
6	9.466563000	-1.733461000	0.603619000
6	8.245228000	-1.289365000	-0.219913000
6	6.922502000	-2.024620000	0.030190000
16	6.810125000	-3.673721000	-0.819121000
6	0.696330000	-0.901628000	-2.862280000
8	0.800249000	-1.763084000	-5.869076000
16	1.118470000	-0.912395000	-1.057302000
6	1.834171000	-0.491517000	-3.783797000
8	2.586263000	-0.059406000	-6.263782000
16	1.327578000	-0.406945000	-5.533012000
8	0.286008000	0.666726000	-5.609177000
6	8.318193000	1.546450000	-2.509167000
6	6.950128000	1.487368000	-1.878203000

6	5.960424000	0.646083000	-2.402826000
6	6.638567000	2.216034000	-0.721869000
6	4.707303000	0.531150000	-1.805290000
6	5.390449000	2.109875000	-0.107587000
6	4.413015000	1.265080000	-0.650752000
8	3.195826000	1.215048000	-0.038323000
6	-4.590249000	4.244533000	3.588149000
6	-4.211171000	4.032172000	2.118902000
6	-3.668684000	2.637954000	1.824262000
7	-3.888713000	2.202998000	0.562119000
8	-3.046771000	1.986988000	2.678467000
1	1.227500000	-7.712884000	3.316242000
1	1.210616000	-6.233643000	4.281953000
1	-0.943577000	-7.437734000	1.389385000
1	1.535451000	-4.314593000	2.981566000
1	-1.397630000	-6.042911000	-0.621269000
1	1.085106000	-2.937599000	0.979040000
1	-0.099000000	-2.774708000	-0.965546000
1	8.280901000	1.233951000	-3.559101000
1	9.019983000	0.870479000	-2.001123000
1	6.165438000	0.070744000	-3.302920000
1	7.386117000	2.878289000	-0.288795000
1	3.948347000	-0.107209000	-2.233691000
1	5.166565000	2.679261000	0.789285000
1	2.605273000	0.504862000	-0.427777000
1	-1.009864000	5.068719000	1.913175000
1	-1.363668000	3.694341000	2.930870000
1	-3.193688000	4.993854000	-2.866328000
1	-3.109094000	3.975565000	-1.432524000
1	-0.583223000	4.937779000	4.892165000
1	0.454666000	5.949277000	3.919362000
1	-4.778769000	3.465249000	-3.946987000
1	-4.636879000	2.316996000	-2.600762000
1	1.444888000	-0.566452000	2.020505000
1	1.471459000	-0.011677000	3.712207000
1	-1.332600000	3.683070000	0.150053000
1	-0.054643000	4.832782000	-0.273762000
1	-3.173285000	0.851557000	-3.848717000
1	-4.560724000	-1.244693000	-3.249789000
1	-2.746852000	-1.760421000	3.804977000
1	1.438441000	4.677043000	2.244761000
1	-2.162817000	3.127040000	-4.220786000
1	-3.716612000	-3.458777000	-1.605377000
1	0.159800000	-2.496007000	3.323825000
1	1.669143000	3.278303000	0.460607000
1	-1.089405000	0.278279000	3.471706000

1	3.202051000	2.352181000	2.097087000
1	3.197620000	1.831689000	3.782703000
1	-2.415080000	-1.704784000	-4.524184000
1	-2.018676000	-2.921609000	-3.301319000
1	-1.542423000	-4.166638000	4.261417000
1	-1.825590000	-4.357864000	2.553837000
1	-4.008636000	-3.632160000	4.576766000
1	0.118037000	2.608969000	-4.499424000
1	0.657293000	4.293060000	-4.586472000
1	-5.767144000	-1.254588000	-1.033071000
1	-5.520785000	-2.623782000	0.033006000
1	-0.295491000	2.584499000	4.619552000
1	1.180634000	3.409818000	5.162285000
1	1.134025000	1.639271000	5.059407000
1	-0.977446000	5.858190000	-3.592525000
1	-1.298868000	5.724275000	-1.857946000
1	0.370780000	5.746100000	-2.449317000
1	-6.234608000	-2.908417000	-2.938456000
1	-6.131018000	-4.207582000	-1.781286000
1	-0.777523000	-2.607531000	5.711803000
1	-0.864832000	-0.833399000	5.651042000
1	9.810408000	-3.322907000	-1.905162000
1	11.360868000	-3.963403000	-1.345199000
1	9.452238000	-3.903253000	0.425551000
1	10.985951000	-3.211036000	0.922477000
1	9.179686000	-1.760582000	1.664671000
1	10.226695000	-0.942781000	0.514347000
1	8.047840000	-0.238841000	0.025894000
1	8.473601000	-1.305320000	-1.292377000
1	6.103174000	-1.419928000	-0.365004000
1	6.757365000	-2.168111000	1.102727000
1	5.461638000	-3.668675000	-0.978397000
1	-0.137289000	-0.209079000	-2.970181000
1	0.340099000	-1.898506000	-3.134180000
1	2.227110000	0.496397000	-3.538492000
1	2.653587000	-1.214649000	-3.758014000
1	-5.244823000	3.445414000	3.956436000
1	-3.703806000	4.287804000	4.225905000
1	-5.053248000	4.241467000	1.450912000
1	-3.426099000	4.758873000	1.869496000
1	8.772664000	2.545559000	-2.480380000
1	-0.270263000	-7.162961000	4.073038000
1	-5.130351000	5.194435000	3.663714000
6	2.937294000	-2.567110000	-1.222551000
1	3.364991000	-2.432129000	-0.235811000
1	2.355743000	-3.469176000	-1.363076000

1	3.570515000	-2.273626000	-2.050107000
1	4.318678000	5.623384000	2.767181000
1	3.786247000	4.504054000	1.578724000
1	-4.523429000	2.723570000	-0.068526000
1	-3.511893000	1.304766000	0.277459000
1	1.863335000	2.295100000	-1.219018000
1	11.120062000	-2.211263000	-1.534571000
1	-3.404496000	-3.174724000	1.075294000
1	-4.288269000	-4.181050000	2.934613000
6	-3.698103000	-5.752784000	4.322108000
8	-2.693202000	-6.206104000	4.943199000
8	-4.797439000	-6.340109000	4.129721000

E-RC-charged
E-RC-charged-opt.gjf.log

Temperature 298.150 Kelvin. Pressure 1.00000 Atm.

Zero-point correction= 1.452876 (Hartree/Particle)
Thermal correction to Energy= 1.550413
Thermal correction to Enthalpy= 1.551357
Thermal correction to Gibbs Free Energy= 1.303167
Sum of electronic and zero-point Energies= -5782.086120
Sum of electronic and thermal Energies= -5781.988583
Sum of electronic and thermal Enthalpies= -5781.987639
Sum of electronic and thermal Free Energies= -5782.235829

6	0.801969000	-4.313287000	6.312671000
6	0.462243000	-4.229309000	4.843252000
6	-0.359014000	-5.176097000	4.211496000
6	0.933299000	-3.159003000	4.072461000
6	-0.707597000	-5.057907000	2.867142000
6	0.589995000	-3.022127000	2.725959000
6	-0.233986000	-3.975170000	2.114651000
8	-0.609197000	-3.905186000	0.804616000
6	-0.461641000	5.343683000	0.750571000
6	-3.259784000	2.485497000	-3.772852000
6	-7.929580000	-3.381773000	-0.074096000
6	-0.096158000	-0.277604000	6.756410000
7	0.342637000	2.105012000	0.450386000
6	-0.112894000	6.764305000	1.240434000
6	-4.641213000	1.814839000	-3.814111000
7	-0.986117000	0.993946000	-1.953568000
8	-8.253297000	-2.292757000	0.476557000
8	-0.860639000	-0.463656000	7.739560000
7	-1.303306000	0.097078000	1.988073000

8	-1.696468000	6.714449000	3.038138000
8	-6.422730000	3.355695000	-4.331248000
8	-1.395831000	8.671188000	1.939382000
8	-5.774274000	3.124492000	-2.179226000
6	0.753537000	1.322161000	2.691561000
6	-0.302258000	3.356846000	-1.612043000
6	-2.654504000	-0.589126000	-2.740621000
6	-2.894171000	-1.797953000	1.930846000
28	-1.036234000	0.457242000	0.012163000
6	0.775794000	2.370344000	1.628997000
6	-0.196879000	2.112116000	-2.518776000
6	-2.842812000	-1.370890000	-1.628490000
6	-2.097957000	-0.777416000	2.569660000
6	1.367527000	3.775555000	1.803655000
6	-0.682306000	2.271547000	-4.023711000
6	-3.580755000	-2.684517000	-1.661160000
6	-2.099188000	-0.611927000	4.088918000
6	0.796040000	4.485356000	0.521035000
6	-2.049629000	1.549537000	-4.011649000
6	-3.976530000	-2.859215000	-0.183331000
6	-0.751168000	0.069833000	4.315900000
6	0.633548000	3.263631000	-0.417125000
6	-1.879599000	0.596063000	-2.837846000
6	-3.035211000	-1.888479000	0.526794000
6	-0.680529000	0.931690000	3.050849000
6	2.922920000	3.546651000	1.703708000
6	-2.682919000	-3.827997000	-2.182253000
6	-2.376142000	-1.890344000	4.861706000
7	1.201635000	1.712794000	-2.685649000
6	3.719935000	4.826030000	1.513249000
6	1.578367000	1.346827000	-3.936299000
6	-3.485207000	-5.144575000	-2.432176000
6	-3.647488000	-2.515908000	4.300660000
6	-3.548505000	-2.799391000	2.800052000
8	3.984634000	5.603474000	2.428001000
8	2.703246000	0.955567000	-4.254493000
8	-4.548231000	-5.019597000	-3.101774000
6	0.365145000	1.463424000	-4.830467000
6	-5.466869000	-2.513721000	0.081158000
7	4.163323000	5.052374000	0.233339000
8	-2.984675000	-6.204015000	-1.967921000
8	-4.044174000	-3.858943000	2.399048000
6	1.037476000	4.431410000	3.155933000
6	-0.696371000	3.715357000	-4.535453000
6	-6.412714000	-3.653767000	-0.303750000
6	-0.551639000	0.750956000	5.672110000

6	-1.181640000	7.446156000	2.143915000
6	-5.724389000	2.847831000	-3.422279000
7	-2.427830000	-1.050918000	-0.352037000
8	-8.707694000	-4.304480000	-0.452604000
8	1.016062000	-0.840119000	6.528949000
6	10.654865000	-3.204515000	0.139924000
6	10.242369000	-2.479790000	1.430439000
6	9.590875000	-1.104230000	1.198706000
6	8.348386000	-1.041103000	0.293935000
6	7.030410000	-1.574826000	0.867581000
16	6.929935000	-3.430287000	0.847743000
6	0.736453000	-2.182806000	-2.391928000
8	0.632258000	-4.125800000	-4.868307000
16	1.365311000	-1.810551000	-0.705049000
6	1.814112000	-2.222435000	-3.464439000
8	2.317331000	-2.666690000	-6.001549000
16	1.143727000	-2.740225000	-5.080263000
8	0.067259000	-1.752634000	-5.401518000
6	8.245602000	0.390293000	-3.081201000
6	6.887703000	0.553783000	-2.450964000
6	5.932309000	-0.463148000	-2.578677000
6	6.561437000	1.657066000	-1.651065000
6	4.696882000	-0.391360000	-1.938898000
6	5.332246000	1.742496000	-0.996571000
6	4.390941000	0.716226000	-1.141269000
8	3.185718000	0.855159000	-0.506846000
6	-4.549012000	5.560465000	1.462488000
6	-4.196316000	4.669556000	0.268463000
6	-3.667756000	3.298134000	0.686473000
7	-3.927864000	2.306587000	-0.194916000
8	-3.031552000	3.141293000	1.738548000
1	1.438577000	-5.180125000	6.541083000
1	1.316695000	-3.399764000	6.628987000
1	-0.757144000	-6.009264000	4.788306000
1	1.508743000	-2.378009000	4.561480000
1	-1.373593000	-5.769791000	2.389669000
1	0.906931000	-2.146502000	2.167604000
1	-0.342688000	-3.041973000	0.428580000
1	8.213523000	-0.363550000	-3.876549000
1	8.973954000	0.029840000	-2.341027000
1	6.150978000	-1.330492000	-3.197084000
1	7.282699000	2.462508000	-1.527542000
1	3.962054000	-1.173670000	-2.078135000
1	5.099055000	2.600405000	-0.375038000
1	2.655728000	0.022318000	-0.580748000
1	-0.999263000	5.434263000	-0.201082000

1	-1.158265000	4.859506000	1.439300000
1	-3.249525000	3.271143000	-4.538990000
1	-3.142943000	2.983604000	-2.803702000
1	0.809353000	6.729900000	1.837112000
1	0.108967000	7.414245000	0.383883000
1	-4.833154000	1.419557000	-4.819764000
1	-4.671674000	0.980290000	-3.106471000
1	1.274162000	0.427673000	2.327407000
1	1.271921000	1.664415000	3.591314000
1	-1.341746000	3.459342000	-1.288705000
1	-0.025918000	4.254931000	-2.172682000
1	-3.152394000	-0.910189000	-3.650080000
1	-4.452028000	-2.643438000	-2.321352000
1	-2.899839000	0.113862000	4.326073000
1	1.550683000	5.149816000	0.084850000
1	-2.214762000	1.003407000	-4.948851000
1	-3.789640000	-3.874108000	0.163848000
1	0.031338000	-0.690329000	4.222571000
1	1.636687000	3.076968000	-0.824087000
1	-1.288725000	1.842242000	3.158554000
1	3.137819000	2.837934000	0.899393000
1	3.259811000	3.089151000	2.639632000
1	-2.232341000	-3.520816000	-3.137145000
1	-1.868191000	-4.018676000	-1.474701000
1	-2.455641000	-1.669117000	5.931860000
1	-1.540058000	-2.583531000	4.736227000
1	-4.496935000	-1.830567000	4.452070000
1	-3.901503000	-3.463343000	4.786362000
1	0.045569000	0.434019000	-5.038592000
1	0.626255000	1.924745000	-5.788276000
1	-5.725980000	-1.603153000	-0.477409000
1	-5.618967000	-2.275181000	1.136463000
1	0.000089000	4.767836000	3.220509000
1	1.683170000	5.297947000	3.319132000
1	1.230750000	3.730616000	3.975743000
1	-1.042437000	3.737349000	-5.575650000
1	-1.347502000	4.370872000	-3.952642000
1	0.316859000	4.135852000	-4.513159000
1	-6.253822000	-3.954371000	-1.347551000
1	-6.145724000	-4.542840000	0.288120000
1	-1.466547000	1.267257000	5.988249000
1	0.243300000	1.505988000	5.583456000
1	9.784133000	-3.646335000	-0.358017000
1	11.364445000	-4.014294000	0.344542000
1	9.570647000	-3.125206000	2.010582000
1	11.130059000	-2.322920000	2.059032000

1	9.338706000	-0.659579000	2.172076000
1	10.351611000	-0.447155000	0.751695000
1	8.159571000	0.014257000	0.063035000
1	8.546592000	-1.523628000	-0.670532000
1	6.204282000	-1.208228000	0.254538000
1	6.885771000	-1.224214000	1.894277000
1	5.585105000	-3.520299000	0.926294000
1	0.005546000	-1.397133000	-2.590539000
1	0.202727000	-3.135479000	-2.339342000
1	2.273139000	-1.244211000	-3.618225000
1	2.594896000	-2.953221000	-3.231320000
1	-5.203696000	5.023891000	2.161108000
1	-3.657957000	5.881529000	2.009177000
1	-5.041585000	4.540355000	-0.415257000
1	-3.398871000	5.143581000	-0.322967000
1	8.688894000	1.293673000	-3.520394000
1	-0.085155000	-4.386416000	6.954912000
1	-5.098593000	6.441281000	1.113492000
6	2.454712000	-3.250384000	-0.409092000
1	3.380575000	-3.195265000	-0.980718000
1	2.686415000	-3.254550000	0.655588000
1	1.916693000	-4.165640000	-0.654118000
1	4.525271000	5.976063000	0.031126000
1	3.758070000	4.549240000	-0.544623000
1	-4.557273000	2.468820000	-1.000112000
1	-3.512582000	1.393291000	-0.039373000
1	1.805940000	1.502649000	-1.899157000
1	11.112951000	-2.516637000	-0.581930000

E-TS$_1$-charged

E-TS$_1$-charged-opt.gjf.log

Temperature 298.150 Kelvin. Pressure 1.00000 Atm.

Zero-point correction= 1.449905 (Hartree/Particle)
Thermal correction to Energy= 1.547046
Thermal correction to Enthalpy= 1.547990
Thermal correction to Gibbs Free Energy= 1.301900
Sum of electronic and zero-point Energies= -5782.061941
Sum of electronic and thermal Energies= -5781.964801
Sum of electronic and thermal Enthalpies= -5781.963857
Sum of electronic and thermal Free Energies= -5782.209947

6	1.217453000	-5.895291000	5.058174000
6	0.854775000	-5.392498000	3.682078000
6	0.013967000	-6.108168000	2.815665000

6	1.347733000	-4.158645000	3.238922000
6	-0.330183000	-5.612440000	1.558477000
6	1.016472000	-3.649985000	1.983671000
6	0.174679000	-4.376147000	1.130158000
8	-0.178354000	-3.932148000	-0.106406000
6	-0.833593000	4.811029000	1.994717000
6	-3.542960000	3.134468000	-2.959544000
6	-7.735895000	-3.603070000	-0.609268000
6	0.185946000	-1.947233000	6.431573000
7	0.343633000	1.924683000	0.852427000
6	-0.792312000	5.992882000	2.972209000
6	-4.878359000	2.395023000	-3.133301000
7	-1.052299000	1.385124000	-1.737551000
8	-8.020908000	-2.739338000	0.266444000
8	-0.589905000	-2.357466000	7.334876000
7	-1.089218000	-0.541424000	1.878292000
8	-2.266221000	7.647709000	3.875271000
8	-6.815184000	3.831491000	-3.110277000
8	-2.576181000	7.042210000	1.717257000
8	-5.971596000	3.109183000	-1.142403000
6	0.922909000	0.603056000	2.789499000
6	-0.548670000	3.611247000	-0.760419000
6	-2.712028000	-0.033568000	-2.805836000
6	-2.626226000	-2.415666000	1.401050000
28	-0.818744000	0.237937000	-0.023879000
6	0.809295000	1.892718000	2.045564000
6	-0.382210000	2.684172000	-1.984145000
6	-2.812192000	-1.077885000	-1.925072000
6	-1.838653000	-1.555223000	2.246880000
6	1.230229000	3.253457000	2.604856000
6	-0.971675000	3.197590000	-3.366262000
6	-3.520809000	-2.364173000	-2.249149000
6	-1.800894000	-1.751321000	3.761298000
6	0.526150000	4.220964000	1.586189000
6	-2.289172000	2.396822000	-3.493403000
6	-3.814142000	-2.927781000	-0.847602000
6	-0.472137000	-1.082660000	4.121495000
6	0.463003000	3.303443000	0.334602000
6	-1.991662000	1.180475000	-2.630675000
6	-2.847075000	-2.144784000	0.034015000
6	-0.472507000	0.052623000	3.092411000
6	2.792535000	3.281945000	2.456313000
6	-2.628413000	-3.303447000	-3.090446000
6	-2.012830000	-3.186391000	4.213919000
7	1.028669000	2.486918000	-2.297494000
6	3.402009000	4.651447000	2.705303000

6	1.357146000	2.506594000	-3.616688000
6	-3.432683000	-4.540059000	-3.606090000
6	-3.270876000	-3.721376000	3.539035000
6	-3.204510000	-3.633914000	2.014318000
8	3.563724000	5.123365000	3.828456000
8	2.490812000	2.343948000	-4.066018000
8	-4.520774000	-4.276537000	-4.190004000
6	0.078101000	2.712630000	-4.397677000
6	-5.288654000	-2.715291000	-0.409706000
7	3.796350000	5.326917000	1.577797000
8	-2.915237000	-5.670888000	-3.407563000
8	-3.659054000	-4.585612000	1.372596000
6	0.837538000	3.434335000	4.077126000
6	-1.106921000	4.719577000	-3.469458000
6	-6.238972000	-3.748238000	-1.018801000
6	-0.277682000	-0.711711000	5.593036000
6	-2.004837000	6.974862000	2.841106000
6	-6.003241000	3.178202000	-2.414976000
7	-2.318632000	-1.086976000	-0.634508000
8	-8.533821000	-4.394911000	-1.187888000
8	1.311804000	-2.424477000	6.106017000
6	10.815166000	-2.683703000	-0.770367000
6	10.340302000	-2.327180000	0.646871000
6	9.569363000	-0.998306000	0.740725000
6	8.291101000	-0.846699000	-0.101668000
6	7.052782000	-1.624992000	0.361163000
16	7.063454000	-3.417441000	-0.130233000
6	0.722276000	-1.449371000	-2.523187000
8	0.877004000	-2.746664000	-5.359506000
16	1.147320000	-1.100153000	-0.753821000
6	1.797742000	-1.068893000	-3.528125000
8	2.425542000	-0.920853000	-6.078269000
16	1.242012000	-1.303046000	-5.248831000
8	0.072237000	-0.383798000	-5.422815000
6	8.131433000	1.470140000	-2.924565000
6	6.775823000	1.456597000	-2.266016000
6	5.886579000	0.398490000	-2.495948000
6	6.380563000	2.455068000	-1.365832000
6	4.650458000	0.331224000	-1.857188000
6	5.145701000	2.404878000	-0.718724000
6	4.269555000	1.340406000	-0.965733000
8	3.060237000	1.345580000	-0.333060000
6	-4.774924000	4.566280000	2.985147000
6	-4.393086000	4.064413000	1.589491000
6	-3.760107000	2.676073000	1.594894000
7	-3.959125000	1.961990000	0.462430000

8	-3.087733000	2.265769000	2.554862000
1	1.880635000	-6.771582000	5.014618000
1	1.727556000	-5.103585000	5.617650000
1	-0.401825000	-7.061285000	3.138598000
1	1.934494000	-3.551092000	3.921862000
1	-1.013132000	-6.148866000	0.907328000
1	1.373993000	-2.674415000	1.673288000
1	0.131058000	-3.000878000	-0.221037000
1	8.110566000	0.933327000	-3.880073000
1	8.882168000	0.969749000	-2.297068000
1	6.159846000	-0.391626000	-3.191764000
1	7.049206000	3.290033000	-1.165162000
1	3.971139000	-0.485696000	-2.054802000
1	4.855693000	3.187242000	-0.024423000
1	2.529268000	0.514862000	-0.518394000
1	-1.302006000	5.214105000	1.091835000
1	-1.504527000	4.026717000	2.360213000
1	-3.624397000	4.096488000	-3.480162000
1	-3.404296000	3.359554000	-1.896397000
1	-0.730839000	5.677591000	4.017460000
1	0.115670000	6.591692000	2.785058000
1	-5.108026000	2.284576000	-4.200541000
1	-4.813214000	1.395675000	-2.691950000
1	1.464528000	-0.128568000	2.178530000
1	1.474698000	0.737719000	3.723379000
1	-1.572103000	3.513726000	-0.388439000
1	-0.393573000	4.651579000	-1.059585000
1	-3.248804000	-0.136817000	-3.742671000
1	-4.432444000	-2.190330000	-2.827584000
1	-2.621744000	-1.133317000	4.170564000
1	1.181386000	5.070800000	1.362067000
1	-2.475209000	2.105563000	-4.534392000
1	-3.583950000	-3.990405000	-0.788293000
1	0.339364000	-1.765648000	3.849119000
1	1.455757000	3.352811000	-0.129601000
1	-1.123179000	0.875020000	3.425664000
1	3.069425000	2.907130000	1.467892000
1	3.222109000	2.596629000	3.194562000
1	-2.246779000	-2.752001000	-3.961028000
1	-1.765731000	-3.630518000	-2.498924000
1	-2.080615000	-3.222728000	5.306474000
1	-1.153106000	-3.793893000	3.922044000
1	-4.145506000	-3.134258000	3.862154000
1	-3.468219000	-4.768037000	3.789858000
1	-0.164945000	1.729316000	-4.821768000
1	0.226064000	3.409664000	-5.228173000

1	-5.600559000	-1.700079000	-0.692775000
1	-5.371954000	-2.765562000	0.678280000
1	-0.242928000	3.340119000	4.211602000
1	1.154173000	4.415575000	4.435275000
1	1.325518000	2.680994000	4.705202000
1	-1.509903000	4.990322000	-4.452421000
1	-1.765554000	5.145209000	-2.709024000
1	-0.122767000	5.194213000	-3.370881000
1	-6.159265000	-3.756041000	-2.113562000
1	-5.909536000	-4.750962000	-0.705765000
1	-1.197451000	-0.282271000	6.009485000
1	0.511769000	0.050487000	5.668888000
1	9.983348000	-3.060173000	-1.376888000
1	11.588118000	-3.460534000	-0.750023000
1	9.728417000	-3.148893000	1.042035000
1	11.211176000	-2.251501000	1.313096000
1	9.316323000	-0.808048000	1.793743000
1	10.256609000	-0.192365000	0.442792000
1	8.002611000	0.211519000	-0.076034000
1	8.490889000	-1.073726000	-1.155670000
1	6.167490000	-1.186156000	-0.104065000
1	6.938803000	-1.557990000	1.447886000
1	5.719970000	-3.542536000	-0.273773000
1	-0.188174000	-0.889546000	-2.732789000
1	0.487631000	-2.512732000	-2.613286000
1	2.091793000	-0.021480000	-3.443456000
1	2.689342000	-1.692486000	-3.424466000
1	-5.368981000	3.822541000	3.530321000
1	-3.890122000	4.804356000	3.581049000
1	-5.252307000	4.065199000	0.911040000
1	-3.661021000	4.766775000	1.168489000
1	8.514507000	2.479687000	-3.123708000
1	0.353972000	-6.180777000	5.672849000
1	-5.378898000	5.474178000	2.881853000
6	3.117454000	-2.665669000	-0.662703000
1	3.684970000	-2.152252000	0.103680000
1	2.606280000	-3.568954000	-0.360613000
1	3.542875000	-2.662486000	-1.657686000
1	4.031003000	6.305700000	1.687424000
1	3.454742000	5.046325000	0.668522000
1	-4.655296000	2.276256000	-0.238994000
1	-3.552256000	1.035490000	0.392840000
1	1.694032000	2.132926000	-1.617981000
1	11.216765000	-1.809581000	-1.298272000

vii) Optimized structures of experimentally well-defined synthetic F430 complexes *viz.* **Coenzyme-F430**, **Pentamethyl ester F430M** and **F430 model complex.**

a) Optimized reduced (doublet) and oxidized (triplet and singlet) state of **Coenzyme-F430** in aqueous medium.

Coenzyme-F430

Coenzyme-F430-reduced-state-doublet-H_2O
reduced-state-doublet.gjf.log

28	0.364930000	0.070707000	-0.385368000
7	-1.551689000	-0.754864000	-0.424897000
7	-0.373992000	1.952268000	-0.187924000
7	2.349942000	0.760275000	-0.655178000
7	1.057548000	-1.809041000	-0.215050000
6	-1.900324000	-2.058100000	-0.548403000
6	-3.417037000	-2.233656000	-0.532307000
6	-2.681641000	0.021647000	-0.388045000
6	-3.922537000	-0.790146000	-0.706808000
6	2.481247000	-2.033154000	0.094227000
6	0.414575000	-2.941016000	-0.253974000
6	1.267660000	-4.145148000	0.097411000
6	2.684893000	-3.562289000	0.272205000
6	-0.984921000	-3.130859000	-0.565251000
6	3.397236000	0.066144000	-0.939999000
6	2.736203000	2.182723000	-0.517951000
6	4.252801000	2.137399000	-0.206784000
6	4.676491000	0.906894000	-1.074221000
6	3.357727000	-1.430961000	-1.014614000
6	0.425006000	3.188208000	-0.116308000
6	-1.642955000	2.269749000	-0.015270000
6	-0.441220000	4.229170000	0.670949000
6	-1.865142000	3.757488000	0.242535000
6	1.831625000	2.941960000	0.444752000
6	-2.734825000	1.369427000	-0.140638000
1	2.292134000	3.916201000	0.646362000
1	1.739862000	2.414789000	1.397109000
1	-2.073687000	4.203545000	-0.741636000
6	-3.022537000	4.153067000	1.178764000
1	-2.759291000	5.076770000	1.703544000
1	-3.168698000	3.393453000	1.954256000
6	-4.341638000	4.416374000	0.445433000
1	-4.214990000	5.216451000	-0.296326000
1	-4.696029000	3.549415000	-0.121919000
6	-5.450509000	4.837625000	1.383347000
8	-5.336088000	5.030159000	2.577889000

8	-6.621321000	4.988743000	0.725959000
1	-3.728062000	1.795890000	-0.077662000
6	-0.220647000	4.180720000	2.183071000
1	0.813841000	4.428833000	2.437619000
1	-0.862185000	4.902353000	2.696344000
1	-0.442907000	3.184713000	2.582549000
6	-0.084012000	5.572427000	0.016652000
7	0.500099000	3.850051000	-1.430934000
6	0.264230000	5.192936000	-1.423887000
8	0.322404000	5.938734000	-2.395854000
1	2.618217000	2.651728000	-1.501638000
1	4.738562000	3.045774000	-0.581350000
6	4.598453000	2.041303000	1.292947000
1	5.687881000	2.028406000	1.401987000
1	4.267314000	2.966899000	1.776308000
6	4.020947000	0.858604000	2.082737000
1	2.932608000	0.789493000	1.976983000
1	4.410927000	-0.101789000	1.724925000
6	4.351051000	0.948943000	3.562194000
8	5.043707000	1.803823000	4.070651000
8	3.822542000	-0.023277000	4.346014000
6	5.975365000	0.211940000	-0.638988000
1	6.270553000	-0.553779000	-1.363144000
1	6.785555000	0.947849000	-0.590771000
1	5.895435000	-0.278825000	0.331503000
6	4.852385000	1.386896000	-2.556622000
1	5.757652000	2.001477000	-2.603808000
1	4.011054000	2.017503000	-2.855488000
6	4.895683000	0.247019000	-3.566136000
8	3.876185000	-0.374346000	-3.876523000
7	6.111501000	-0.046011000	-4.088639000
1	6.190175000	-0.795842000	-4.763095000
1	6.946297000	0.467608000	-3.847239000
1	2.977328000	-1.741561000	-1.993485000
1	4.368131000	-1.826634000	-0.914002000
1	2.702332000	-1.510714000	1.029581000
1	-0.892248000	6.308849000	0.039614000
1	0.795880000	6.031694000	0.485134000
1	0.739972000	3.358793000	-2.282091000
1	3.345848000	-3.943106000	-0.514364000
6	3.273424000	-3.941018000	1.636657000
1	3.334527000	-5.033218000	1.732835000
1	2.600448000	-3.604309000	2.439099000
6	4.641625000	-3.354080000	1.933658000
8	5.227108000	-2.549071000	1.237585000
8	5.217068000	-3.758179000	3.087975000

1	0.904570000	-4.549543000	1.054808000
6	1.072791000	-5.227692000	-0.962647000
6	-1.427110000	-4.497391000	-0.885647000
6	-0.399400000	-5.638988000	-0.941287000
1	1.352300000	-4.828502000	-1.946747000
1	1.719199000	-6.090951000	-0.765665000
1	-0.592299000	-6.251794000	-0.048213000
1	-0.667633000	-6.261708000	-1.801010000
8	-2.600738000	-4.812933000	-1.128735000
1	-4.740382000	-0.553145000	-0.020052000
1	-3.732523000	-2.889047000	-1.344519000
6	-3.863826000	-2.859919000	0.808365000
1	-3.738347000	-2.120485000	1.609422000
1	-3.218659000	-3.705929000	1.052724000
6	-5.312809000	-3.340089000	0.763302000
1	-5.994210000	-2.549509000	0.429860000
1	-5.420542000	-4.145810000	0.025688000
6	-5.799987000	-3.859199000	2.093887000
8	-5.160558000	-3.890597000	3.128010000
8	-7.075965000	-4.303267000	2.015552000
6	-4.389554000	-0.482063000	-2.139470000
1	-4.530973000	0.599295000	-2.267633000
1	-3.632724000	-0.782477000	-2.872429000
6	-5.707397000	-1.139122000	-2.486703000
8	-6.536694000	-1.532812000	-1.688932000
8	-5.887322000	-1.211648000	-3.821430000
1	-7.290061000	5.269074000	1.380607000
1	-6.768902000	-1.603284000	-3.978373000
1	-7.326928000	-4.621478000	2.904293000
1	4.653473000	-4.401348000	3.553427000
1	3.277321000	-0.635889000	3.822628000

Triplet

Coenzyme-F430- oxidized-state-triplet-H2O

oxidized-state-triplet.gjf.log

28	-0.338772000	0.063033000	0.365935000
7	1.580946000	-0.710143000	0.399093000
7	0.344890000	1.982238000	0.187932000
7	-2.331204000	0.677017000	0.649086000
7	-0.991645000	-1.823335000	0.144273000
6	1.949815000	-2.010181000	0.547839000
6	3.465627000	-2.153156000	0.530480000
6	2.693805000	0.090272000	0.339614000
6	3.946624000	-0.696007000	0.662714000
6	-2.394472000	-2.054858000	-0.232852000
6	-0.324779000	-2.935435000	0.210935000

6	-1.140034000	-4.150221000	-0.175389000
6	-2.546516000	-3.581557000	-0.478409000
6	1.058975000	-3.094016000	0.578471000
6	-3.343655000	-0.052863000	0.971168000
6	-2.722179000	2.106614000	0.651034000
6	-4.262438000	2.067067000	0.500380000
6	-4.588821000	0.773837000	1.323272000
6	-3.324701000	-1.546223000	0.874421000
6	-0.486137000	3.201748000	0.127954000
6	1.601619000	2.307962000	-0.035541000
6	0.333910000	4.227753000	-0.723889000
6	1.782167000	3.789366000	-0.336638000
6	-1.912778000	2.912610000	-0.358060000
6	2.715296000	1.432478000	0.070287000
1	-2.413398000	3.873483000	-0.518016000
1	-1.869129000	2.394766000	-1.319622000
1	2.016207000	4.253203000	0.633168000
6	2.902529000	4.178290000	-1.319855000
1	2.596613000	5.072224000	-1.871358000
1	3.049015000	3.394041000	-2.069895000
6	4.231425000	4.506544000	-0.631703000
1	4.102411000	5.338800000	0.073061000
1	4.620379000	3.676856000	-0.032044000
6	5.305997000	4.906904000	-1.617953000
8	5.153560000	5.042721000	-2.815851000
8	6.489468000	5.110239000	-1.000235000
1	3.696057000	1.880056000	-0.019826000
6	0.054283000	4.130399000	-2.223418000
1	-0.991901000	4.362983000	-2.442036000
1	0.667262000	4.842605000	-2.781966000
1	0.270022000	3.125486000	-2.603491000
6	-0.014298000	5.584482000	-0.093829000
7	-0.509114000	3.892698000	1.425350000
6	-0.304129000	5.243022000	1.368212000
8	-0.347425000	6.013694000	2.318513000
1	-2.500980000	2.508083000	1.645139000
1	-4.702926000	2.943390000	0.988247000
6	-4.787718000	2.050277000	-0.948922000
1	-5.882504000	2.051967000	-0.901424000
1	-4.511486000	2.988507000	-1.440323000
6	-4.334728000	0.879720000	-1.845174000
1	-3.273531000	0.972312000	-2.102164000
1	-4.441536000	-0.079493000	-1.342414000
6	-5.116138000	0.864132000	-3.151162000
8	-5.289723000	1.859555000	-3.827646000
8	-5.627694000	-0.315186000	-3.546720000

6	-5.937151000	0.107034000	1.010015000
1	-6.126519000	-0.728157000	1.691354000
1	-6.743652000	0.834311000	1.149024000
1	-6.004545000	-0.278002000	-0.008769000
6	-4.572164000	1.137815000	2.848692000
1	-5.463755000	1.738413000	3.054591000
1	-3.700985000	1.752188000	3.088437000
6	-4.481551000	-0.079492000	3.760513000
8	-3.438778000	-0.734920000	3.841496000
7	-5.598934000	-0.398410000	4.453453000
1	-5.579696000	-1.191885000	5.080925000
1	-6.444378000	0.151393000	4.407456000
1	-3.031969000	-1.968036000	1.841755000
1	-4.336331000	-1.898848000	0.663944000
1	-2.588985000	-1.484356000	-1.144576000
1	0.783942000	6.327821000	-0.169647000
1	-0.916131000	6.020186000	-0.541769000
1	-0.726966000	3.431330000	2.298892000
1	-3.278343000	-3.996899000	0.221916000
6	-2.978517000	-3.930721000	-1.909362000
1	-3.078944000	-5.017705000	-2.017455000
1	-2.194980000	-3.627402000	-2.619495000
6	-4.250917000	-3.268165000	-2.397945000
8	-4.844965000	-2.373264000	-1.811091000
8	-4.713614000	-3.661237000	-3.591205000
1	-0.697374000	-4.572226000	-1.089195000
6	-0.997514000	-5.197716000	0.930680000
6	1.506798000	-4.453462000	0.943230000
6	0.480181000	-5.594722000	1.008116000
1	-1.340990000	-4.775337000	1.883611000
1	-1.620044000	-6.073502000	0.717932000
1	0.725133000	-6.253199000	0.162333000
1	0.703781000	-6.167193000	1.913950000
8	2.676045000	-4.739746000	1.217932000
1	4.750785000	-0.461367000	-0.040059000
1	3.792344000	-2.781028000	1.359959000
6	3.911439000	-2.812846000	-0.796654000
1	3.764601000	-2.101279000	-1.618406000
1	3.278494000	-3.676496000	-1.008177000
6	5.368478000	-3.267649000	-0.752029000
1	6.042438000	-2.454199000	-0.463119000
1	5.501072000	-4.041104000	0.015157000
6	5.838585000	-3.831985000	-2.070826000
8	5.179653000	-3.909864000	-3.089918000
8	7.120356000	-4.256835000	-1.997830000
6	4.420678000	-0.338251000	2.082676000

1	4.541973000	0.748069000	2.181399000
1	3.678699000	-0.634323000	2.832289000
6	5.755120000	-0.962615000	2.429492000
8	6.558200000	-1.406828000	1.632200000
8	5.976168000	-0.939249000	3.758157000
1	7.135414000	5.374607000	-1.683783000
1	6.864184000	-1.315293000	3.917048000
1	7.361253000	-4.606310000	-2.877630000
1	-4.163939000	-4.367867000	-3.975838000
1	-5.437256000	-1.040703000	-2.905504000

Singlet

Coenzyme-F430- oxidized-state-singlet-H2O

oxidized-state-singlet.gjf.log

28	-0.334283000	-0.023390000	0.295591000
7	1.549438000	-0.572855000	0.341284000
7	0.121903000	1.899427000	0.333119000
7	-2.249212000	0.300889000	0.744932000
7	-0.787184000	-1.781685000	-0.256003000
6	2.041387000	-1.844699000	0.383351000
6	3.551178000	-1.849858000	0.546185000
6	2.593591000	0.315326000	0.480145000
6	3.851273000	-0.390210000	0.924101000
6	-2.164163000	-2.062246000	-0.704602000
6	-0.051632000	-2.852417000	-0.330145000
6	-0.737807000	-4.013896000	-1.005362000
6	-2.147176000	-3.474295000	-1.340671000
6	1.283958000	-2.991509000	0.150780000
6	-3.146516000	-0.588686000	1.023963000
6	-2.767370000	1.645282000	1.106957000
6	-4.298231000	1.446201000	1.201705000
6	-4.352525000	-0.008626000	1.766589000
6	-3.082509000	-1.983173000	0.508171000
6	-0.804572000	3.052057000	0.460762000
6	1.325981000	2.385493000	0.097068000
6	-0.180422000	4.205525000	-0.386204000
6	1.338519000	3.869975000	-0.227160000
6	-2.247122000	2.689762000	0.137819000
6	2.527073000	1.656822000	0.263861000
1	-2.854098000	3.596494000	0.220920000
1	-2.319757000	2.326901000	-0.889511000
1	1.717835000	4.379725000	0.670606000
6	2.210430000	4.321456000	-1.415331000
1	1.894389000	5.337291000	-1.673728000
1	2.005450000	3.702531000	-2.294741000
6	3.720688000	4.371899000	-1.165194000

1	3.949675000	4.798331000	-0.179154000
1	4.185318000	3.382128000	-1.185629000
6	4.437856000	5.218883000	-2.196983000
8	3.909887000	5.900209000	-3.053200000
8	5.777074000	5.134673000	-2.048662000
1	3.450848000	2.214496000	0.321491000
6	-0.668806000	4.218676000	-1.835391000
1	-1.747069000	4.397022000	-1.876639000
1	-0.187370000	5.019202000	-2.402454000
1	-0.458490000	3.269212000	-2.339941000
6	-0.538519000	5.473295000	0.406635000
7	-0.704688000	3.627894000	1.810462000
6	-0.600704000	4.990468000	1.854600000
8	-0.567016000	5.671526000	2.870890000
1	-2.397131000	1.887633000	2.106729000
1	-4.710126000	2.150581000	1.932279000
6	-5.076303000	1.656542000	-0.114367000
1	-6.142698000	1.538204000	0.106940000
1	-4.958010000	2.696981000	-0.431553000
6	-4.723466000	0.746240000	-1.301616000
1	-3.686934000	0.893645000	-1.628265000
1	-4.801844000	-0.311159000	-1.046556000
6	-5.608048000	1.025327000	-2.510276000
8	-6.112229000	2.108922000	-2.733970000
8	-5.797531000	0.005405000	-3.363820000
6	-5.681426000	-0.752687000	1.576084000
1	-5.646443000	-1.740769000	2.045547000
1	-6.483698000	-0.185212000	2.058421000
1	-5.957140000	-0.891210000	0.529601000
6	-4.042772000	0.031119000	3.304378000
1	-4.920303000	0.447518000	3.808206000
1	-3.200285000	0.694841000	3.513160000
6	-3.644925000	-1.328436000	3.865680000
8	-2.578000000	-1.857640000	3.537880000
7	-4.518962000	-1.912460000	4.714771000
1	-4.283556000	-2.806579000	5.125861000
1	-5.378546000	-1.467882000	5.002511000
1	-2.750426000	-2.662449000	1.300436000
1	-4.088278000	-2.296787000	0.215142000
1	-2.452347000	-1.292699000	-1.422002000
1	0.185908000	6.284300000	0.294384000
1	-1.522156000	5.866409000	0.121301000
1	-0.771125000	3.079240000	2.657833000
1	-2.917108000	-4.099262000	-0.878792000
6	-2.387872000	-3.438006000	-2.854623000
1	-2.350389000	-4.452242000	-3.271483000

1	-1.587912000	-2.872229000	-3.353232000
6	-3.697238000	-2.792314000	-3.260876000
8	-4.482426000	-2.276168000	-2.476520000
8	-3.981266000	-2.758754000	-4.567317000
1	-0.185859000	-4.231540000	-1.930746000
6	-0.607797000	-5.240238000	-0.096366000
6	1.808210000	-4.358395000	0.346370000
6	0.885735000	-5.551900000	0.062166000
1	-1.068464000	-5.032072000	0.877518000
1	-1.133369000	-6.097020000	-0.530724000
1	1.264245000	-5.998157000	-0.868970000
1	1.068358000	-6.288362000	0.850712000
8	2.952703000	-4.597493000	0.736796000
1	4.728212000	0.002093000	0.403485000
1	3.837898000	-2.559181000	1.323124000
6	4.224393000	-2.266778000	-0.784134000
1	4.105050000	-1.458406000	-1.515792000
1	3.720291000	-3.143171000	-1.194999000
6	5.705191000	-2.590129000	-0.597077000
1	6.242911000	-1.775264000	-0.100823000
1	5.824024000	-3.459201000	0.062068000
6	6.397943000	-2.894293000	-1.903536000
8	5.886273000	-2.864092000	-3.006192000
8	7.697438000	-3.213471000	-1.710830000
6	4.043061000	-0.178702000	2.436647000
1	4.043595000	0.891960000	2.676316000
1	3.216734000	-0.620310000	3.005020000
6	5.350398000	-0.752311000	2.939826000
8	6.280285000	-1.111475000	2.244162000
8	5.377941000	-0.796580000	4.285446000
1	6.184031000	5.708054000	-2.726910000
1	6.251687000	-1.143296000	4.552720000
1	8.085804000	-3.395466000	-2.588498000
1	-3.285888000	-3.187630000	-5.098627000
1	-5.373287000	-0.830552000	-3.049940000

b) Optimized reduced (doublet) and oxidized (triplet and singlet) state of **Pentamethyl ester F430M** in solvent THF.

Pentamethyl ester F430M

Pentamethyl-ester-F430M- reduced-state-doublet-THF
reduced-state-doublet.gjf.log

Temperature 298.150 Kelvin. Pressure 1.00000 Atm.

Zero-point correction= 1.066319 (Hartree/Particle)
Thermal correction to Energy= 1.133149
Thermal correction to Enthalpy= 1.134094
Thermal correction to Gibbs Free Energy= 0.955537
Sum of electronic and zero-point Energies= -3302.795609
Sum of electronic and thermal Energies= -3302.728779
Sum of electronic and thermal Enthalpies= -3302.727835
Sum of electronic and thermal Free Energies= -3302.906391

28	0.536555000	0.230238000	-0.567518000
7	-1.383914000	-0.579470000	-0.537312000
7	-0.166837000	2.093916000	-0.265713000
7	2.519330000	0.918306000	-0.918023000
7	1.219496000	-1.644167000	-0.488044000
6	-1.745811000	-1.875753000	-0.694696000
6	-3.259451000	-2.051212000	-0.636878000
6	-2.503818000	0.199421000	-0.410111000
6	-3.772251000	-0.598592000	-0.636973000
6	2.653222000	-1.885118000	-0.247917000
6	0.570288000	-2.773318000	-0.532186000
6	1.429091000	-3.990189000	-0.245040000
6	2.851615000	-3.416017000	-0.086179000
6	-0.837873000	-2.947696000	-0.800643000
6	3.547287000	0.222652000	-1.264846000
6	2.938827000	2.322820000	-0.704489000
6	4.459162000	2.228288000	-0.424602000
6	4.838901000	1.051227000	-1.383480000
6	3.481008000	-1.270700000	-1.389093000
6	0.650121000	3.318501000	-0.190111000
6	-1.425734000	2.424342000	-0.036838000
6	-0.174567000	4.349004000	0.652763000
6	-1.617004000	3.908701000	0.258271000
6	2.069779000	3.039833000	0.319520000
6	-2.531691000	1.541568000	-0.129240000
1	2.544448000	4.000906000	0.550651000
1	2.001772000	2.469856000	1.249050000
1	-1.846966000	4.378353000	-0.710311000
6	-2.740354000	4.304026000	1.234771000
1	-2.449897000	5.214631000	1.768153000
1	-2.874274000	3.532761000	2.001029000
6	-4.075858000	4.597333000	0.542856000
1	-3.955241000	5.411752000	-0.184623000
1	-4.454565000	3.747309000	-0.033487000
6	-5.153108000	5.017891000	1.521074000
8	-4.981876000	5.257612000	2.700810000

8	-6.350105000	5.108449000	0.909439000
1	-3.514952000	1.973911000	0.006034000
6	0.091154000	4.255136000	2.155339000
1	1.135266000	4.485881000	2.386319000
1	-0.527691000	4.967450000	2.708043000
1	-0.129590000	3.250193000	2.532723000
6	0.179781000	5.704638000	0.023408000
7	0.691542000	4.014147000	-1.488012000
6	0.471598000	5.359877000	-1.439044000
8	0.503293000	6.129550000	-2.392052000
1	2.812193000	2.855537000	-1.653924000
1	4.958212000	3.151393000	-0.741928000
6	4.823155000	2.005967000	1.059400000
1	5.914933000	1.975609000	1.150268000
1	4.502699000	2.883788000	1.630949000
6	4.240587000	0.750509000	1.717086000
1	3.146937000	0.728848000	1.648771000
1	4.577716000	-0.163150000	1.224549000
6	4.590633000	0.611824000	3.179931000
8	5.029953000	1.481316000	3.905693000
8	4.328905000	-0.647912000	3.595241000
6	6.142683000	0.315165000	-1.037434000
1	6.399313000	-0.412165000	-1.814061000
1	6.964609000	1.037639000	-0.979407000
1	6.095838000	-0.229260000	-0.094216000
6	4.979115000	1.619456000	-2.837270000
1	5.879816000	2.241877000	-2.870269000
1	4.128493000	2.260442000	-3.082942000
6	5.005196000	0.534765000	-3.906543000
8	3.994463000	-0.109481000	-4.191956000
7	6.199466000	0.318818000	-4.513151000
1	6.265670000	-0.395931000	-5.225784000
1	7.031116000	0.841123000	-4.280692000
1	3.041441000	-1.545512000	-2.353536000
1	4.488457000	-1.685590000	-1.355720000
1	2.916399000	-1.377882000	0.682910000
1	-0.616403000	6.451188000	0.094202000
1	1.081592000	6.139185000	0.473456000
1	0.884034000	3.539473000	-2.360308000
1	3.503264000	-3.796353000	-0.880610000
6	3.457508000	-3.783202000	1.274147000
1	3.520063000	-4.872014000	1.395662000
1	2.809437000	-3.427864000	2.086570000
6	4.836795000	-3.207018000	1.518341000
8	5.469720000	-2.520744000	0.734904000
8	5.297031000	-3.547603000	2.733912000

1	1.089271000	-4.428563000	0.705989000
6	1.203122000	-5.033226000	-1.338790000
6	-1.290200000	-4.289604000	-1.201131000
6	-0.271924000	-5.436208000	-1.304479000
1	1.465736000	-4.601202000	-2.313744000
1	1.846965000	-5.907597000	-1.186129000
1	-0.453598000	-6.070596000	-0.423843000
1	-0.558434000	-6.034546000	-2.175254000
8	-2.463265000	-4.574934000	-1.478046000
1	-4.487019000	-0.417626000	0.173258000
1	-3.593832000	-2.607334000	-1.514105000
6	-3.699428000	-2.827340000	0.623944000
1	-3.551855000	-2.197519000	1.510011000
1	-3.076028000	-3.712814000	0.753907000
6	-5.161036000	-3.263666000	0.528399000
1	-5.835088000	-2.416102000	0.351878000
1	-5.295796000	-3.936171000	-0.328711000
6	-5.642408000	-3.986741000	1.766450000
8	-4.984862000	-4.197838000	2.767429000
8	-6.924824000	-4.381111000	1.625048000
6	-4.462971000	-0.191108000	-1.963141000
1	-4.707562000	0.874817000	-1.935448000
1	-3.799625000	-0.382698000	-2.810985000
6	-5.752757000	-0.967450000	-2.104977000
8	-6.743836000	-0.776901000	-1.423710000
8	-5.657602000	-1.950160000	-3.019190000
6	-7.449715000	5.526274000	1.741886000
1	-7.260023000	6.520100000	2.156481000
1	-8.321593000	5.544971000	1.088191000
1	-7.600006000	4.817544000	2.560753000
6	-6.813925000	-2.804811000	-3.142350000
1	-7.690729000	-2.220112000	-3.431454000
1	-6.560516000	-3.528289000	-3.916459000
1	-7.012795000	-3.310232000	-2.193539000
6	-7.491112000	-5.083117000	2.747780000
1	-8.514513000	-5.322572000	2.458814000
1	-6.927210000	-5.997433000	2.952367000
1	-7.483539000	-4.450046000	3.639380000
6	6.598708000	-3.028931000	3.075136000
1	6.802076000	-3.391270000	4.082420000
1	7.352098000	-3.397080000	2.374098000
1	6.582511000	-1.937168000	3.054690000
6	4.562782000	-0.925307000	4.988858000
1	3.887419000	-0.334376000	5.613536000
1	4.364339000	-1.990142000	5.106693000
1	5.597398000	-0.696125000	5.256530000

Triplet

Pentamethyl-ester-F430M-oxidized-state-triplet -THF

oxidized-state-triplet.gjf.log

Temperature 298.150 Kelvin. Pressure 1.00000 Atm.

Zero-point correction= 1.068518 (Hartree/Particle)
Thermal correction to Energy= 1.135145
Thermal correction to Enthalpy= 1.136089
Thermal correction to Gibbs Free Energy= 0.956729
Sum of electronic and zero-point Energies= -3302.654971
Sum of electronic and thermal Energies= -3302.588345
Sum of electronic and thermal Enthalpies= -3302.587400
Sum of electronic and thermal Free Energies= -3302.766761

28	0.493989000	0.144375000	-0.576703000
7	-1.435235000	-0.594089000	-0.494032000
7	-0.152113000	2.069186000	-0.348310000
7	2.482289000	0.755000000	-0.939155000
7	1.129139000	-1.754449000	-0.408463000
6	-1.834895000	-1.891084000	-0.610017000
6	-3.347652000	-2.014193000	-0.519716000
6	-2.532765000	0.222062000	-0.375981000
6	-3.819697000	-0.549767000	-0.556453000
6	2.559493000	-2.026825000	-0.175902000
6	0.439822000	-2.858212000	-0.437044000
6	1.263465000	-4.090521000	-0.128409000
6	2.706055000	-3.560153000	0.005743000
6	-0.969460000	-2.990027000	-0.700566000
6	3.476305000	0.014321000	-1.297202000
6	2.933752000	2.165189000	-0.829945000
6	4.460047000	2.045708000	-0.609466000
6	4.774368000	0.806724000	-1.510615000
6	3.381448000	-1.477630000	-1.349457000
6	0.704506000	3.273818000	-0.318729000
6	-1.397389000	2.427708000	-0.104257000
6	-0.085461000	4.335336000	0.518285000
6	-1.544684000	3.919392000	0.156550000
6	2.120611000	2.962125000	0.178822000
6	-2.525110000	1.570514000	-0.143966000
1	2.633994000	3.914539000	0.350955000
1	2.057300000	2.442276000	1.137706000
1	-1.776769000	4.359329000	-0.825139000
6	-2.646089000	4.364725000	1.136394000
1	-2.321308000	5.278415000	1.643058000

1	-2.789052000	3.617446000	1.924100000
6	-3.982610000	4.679749000	0.455977000
1	-3.852125000	5.478651000	-0.286366000
1	-4.392984000	3.830647000	-0.099986000
6	-5.032078000	5.141786000	1.446962000
8	-4.832513000	5.378859000	2.622595000
8	-6.231985000	5.269263000	0.851722000
1	-3.493592000	2.031911000	-0.007535000
6	0.203026000	4.264598000	2.017991000
1	1.254224000	4.482538000	2.227158000
1	-0.393917000	4.999197000	2.564613000
1	-0.029210000	3.272467000	2.421570000
6	0.284850000	5.670684000	-0.143934000
7	0.738323000	3.935982000	-1.628557000
6	0.557327000	5.292814000	-1.600820000
8	0.607764000	6.039087000	-2.567817000
1	2.779552000	2.633039000	-1.807947000
1	4.962355000	2.934702000	-1.006869000
6	4.890659000	1.916282000	0.865916000
1	5.982371000	1.846303000	0.905658000
1	4.627629000	2.852907000	1.372289000
6	4.298748000	0.739202000	1.675141000
1	3.208226000	0.752168000	1.666649000
1	4.629857000	-0.218427000	1.265384000
6	4.735030000	0.809860000	3.120979000
8	4.027397000	1.133876000	4.053892000
8	6.039465000	0.488218000	3.252002000
6	6.068537000	0.052615000	-1.167863000
1	6.282151000	-0.714708000	-1.918528000
1	6.907452000	0.756581000	-1.170386000
1	6.037900000	-0.443271000	-0.197199000
6	4.873875000	1.287574000	-2.999506000
1	5.817955000	1.831068000	-3.108539000
1	4.065914000	1.984889000	-3.235525000
6	4.744228000	0.152163000	-4.007222000
8	3.666908000	-0.424508000	-4.173239000
7	5.866265000	-0.187681000	-4.684217000
1	5.815547000	-0.918834000	-5.381772000
1	6.738143000	0.309814000	-4.578609000
1	2.929706000	-1.783362000	-2.298245000
1	4.381232000	-1.907475000	-1.306694000
1	2.851316000	-1.501458000	0.737354000
1	-0.498727000	6.430437000	-0.079575000
1	1.197328000	6.099763000	0.288552000
1	0.933401000	3.450955000	-2.494571000
1	3.328790000	-3.966477000	-0.798138000

6	3.330401000	-3.933030000	1.355341000
1	3.407896000	-5.022174000	1.461602000
1	2.689132000	-3.595582000	2.180091000
6	4.706198000	-3.336100000	1.579401000
8	5.327562000	-2.668711000	0.768927000
8	5.161656000	-3.613890000	2.807694000
1	0.912296000	-4.485575000	0.836571000
6	0.992477000	-5.154556000	-1.192786000
6	-1.472547000	-4.335603000	-1.062684000
6	-0.493343000	-5.513396000	-1.135612000
1	1.261269000	-4.760474000	-2.181491000
1	1.610745000	-6.041575000	-1.016672000
1	-0.688616000	-6.111894000	-0.233413000
1	-0.803193000	-6.129786000	-1.984851000
8	-2.654221000	-4.573224000	-1.323834000
1	-4.513654000	-0.323358000	0.259514000
1	-3.711854000	-2.587616000	-1.373600000
6	-3.778719000	-2.740697000	0.775655000
1	-3.591801000	-2.089091000	1.637793000
1	-3.176886000	-3.638661000	0.919567000
6	-5.253382000	-3.139168000	0.728294000
1	-5.910346000	-2.280124000	0.545894000
1	-5.429110000	-3.833263000	-0.103722000
6	-5.710931000	-3.814527000	2.002976000
8	-5.018426000	-4.021763000	2.980932000
8	-7.008060000	-4.167680000	1.920399000
6	-4.518572000	-0.163857000	-1.884182000
1	-4.739483000	0.907523000	-1.885344000
1	-3.875415000	-0.395204000	-2.737460000
6	-5.829905000	-0.915016000	-1.980272000
8	-6.786000000	-0.705993000	-1.257021000
8	-5.790066000	-1.887305000	-2.905935000
6	-7.307148000	5.727041000	1.696803000
1	-7.077997000	6.715038000	2.104927000
1	-8.185542000	5.773042000	1.053518000
1	-7.470452000	5.025829000	2.519504000
6	-7.554289000	-4.825340000	3.080669000
1	-8.596092000	-5.032667000	2.836845000
1	-7.015464000	-5.755153000	3.282186000
1	-7.487864000	-4.173991000	3.956299000
6	-6.973418000	-2.710920000	-3.001625000
1	-7.841478000	-2.099023000	-3.258137000
1	-6.762532000	-3.432191000	-3.790097000
1	-7.155460000	-3.219730000	-2.051464000
6	6.575023000	0.568148000	4.589596000
1	6.044910000	-0.120266000	5.252648000

1	7.623296000	0.284467000	4.501559000
1	6.482099000	1.586718000	4.974676000
6	6.452072000	-3.053916000	3.141831000
1	7.223650000	-3.462961000	2.484555000
1	6.426778000	-1.966173000	3.043338000
1	6.635215000	-3.345663000	4.175440000

Singlet

Pentamethyl-ester-F430M-oxidized-state-singlet -THF

Temperature 298.150 Kelvin. Pressure 1.00000 Atm.

Zero-point correction= 1.070256 (Hartree/Particle)
Thermal correction to Energy= 1.136255
Thermal correction to Enthalpy= 1.137199
Thermal correction to Gibbs Free Energy= 0.961056
Sum of electronic and zero-point Energies= -3302.659314
Sum of electronic and thermal Energies= -3302.593315
Sum of electronic and thermal Enthalpies= -3302.592370
Sum of electronic and thermal Free Energies= -3302.768514

oxidized-state-singlet.gjf.log

28	-0.458303000	0.085656000	0.496855000
7	1.330541000	-0.694399000	0.466565000
7	0.255643000	1.935694000	0.469496000
7	-2.284338000	0.723093000	0.982959000
7	-1.169646000	-1.623173000	0.022164000
6	1.690347000	-2.004787000	0.327784000
6	3.192444000	-2.178306000	0.459693000
6	2.474758000	0.042439000	0.720458000
6	3.618105000	-0.854419000	1.108394000
6	-2.630840000	-1.777743000	-0.166428000
6	-0.561074000	-2.752795000	-0.230001000
6	-1.469725000	-3.802186000	-0.828161000
6	-2.883006000	-3.215932000	-0.671868000
6	0.817143000	-3.041038000	0.001488000
6	-3.274290000	-0.003563000	1.392096000
6	-2.672229000	2.156510000	1.080797000
6	-4.216230000	2.135806000	1.089312000
6	-4.464507000	0.820481000	1.896226000
6	-3.308471000	-1.474444000	1.168583000
6	-0.518925000	3.204289000	0.429758000
6	1.524759000	2.258505000	0.306699000
6	0.306391000	4.187288000	-0.454403000
6	1.751989000	3.699807000	-0.113231000
6	-1.969240000	2.987182000	0.027520000

6	2.600818000	1.387077000	0.583695000
1	-2.457121000	3.963909000	-0.050115000
1	-2.016825000	2.505173000	-0.951323000
1	2.100661000	4.235643000	0.781835000
6	2.783084000	3.950842000	-1.230965000
1	2.604536000	4.962831000	-1.608404000
1	2.605857000	3.273206000	-2.072715000
6	4.256769000	3.883401000	-0.817120000
1	4.417918000	4.381964000	0.148758000
1	4.617918000	2.858784000	-0.698269000
6	5.159370000	4.567376000	-1.829068000
8	4.789815000	5.296515000	-2.728753000
8	6.449756000	4.271835000	-1.590866000
1	3.584803000	1.814590000	0.710844000
6	-0.046647000	4.099616000	-1.939351000
1	-1.088303000	4.389378000	-2.105640000
1	0.575580000	4.775821000	-2.530684000
1	0.092986000	3.083505000	-2.324908000
6	0.036809000	5.560204000	0.181187000
7	-0.437524000	3.890736000	1.728172000
6	-0.179704000	5.233912000	1.658678000
8	-0.134004000	6.000900000	2.608986000
1	-2.347686000	2.516187000	2.060614000
1	-4.584106000	2.996704000	1.658135000
6	-4.880618000	2.200825000	-0.301616000
1	-5.967339000	2.202374000	-0.164542000
1	-4.640373000	3.168681000	-0.754421000
6	-4.509732000	1.089642000	-1.286685000
1	-3.429896000	1.049794000	-1.464941000
1	-4.785578000	0.100667000	-0.915385000
6	-5.161912000	1.239472000	-2.642863000
8	-5.763426000	2.212338000	-3.049349000
8	-4.967981000	0.116973000	-3.368506000
6	-5.843924000	0.172967000	1.699097000
1	-5.979244000	-0.671248000	2.382687000
1	-6.621413000	0.909287000	1.927499000
1	-6.010409000	-0.196856000	0.686673000
6	-4.291065000	1.126670000	3.422817000
1	-5.160951000	1.706792000	3.746734000
1	-3.404917000	1.742469000	3.598599000
6	-4.091138000	-0.132194000	4.257376000
8	-3.085318000	-0.830971000	4.109925000
7	-5.067913000	-0.433491000	5.143132000
1	-4.959207000	-1.246204000	5.735960000
1	-5.877490000	0.153209000	5.282452000
1	-2.798925000	-1.999473000	1.982069000

1	-4.340329000	-1.820723000	1.139695000
1	-2.949520000	-1.052018000	-0.914591000
1	0.854110000	6.274926000	0.054046000
1	-0.873524000	6.022680000	-0.220233000
1	-0.606697000	3.436026000	2.615583000
1	-3.429594000	-3.779149000	0.092199000
6	-3.678513000	-3.258950000	-1.981608000
1	-3.730564000	-4.282998000	-2.369618000
1	-3.174210000	-2.666447000	-2.756924000
6	-5.091909000	-2.727442000	-1.857845000
8	-5.561955000	-2.200367000	-0.863206000
8	-5.780018000	-2.902786000	-2.993267000
1	-1.205384000	-3.873790000	-1.894631000
6	-1.208158000	-5.163808000	-0.186558000
6	1.271361000	-4.451297000	-0.088552000
6	0.251894000	-5.538247000	-0.433611000
1	-1.416967000	-5.106848000	0.889578000
1	-1.879108000	-5.919159000	-0.609703000
1	0.397640000	-5.761575000	-1.501114000
1	0.549297000	-6.437228000	0.113996000
8	2.437643000	-4.802106000	0.093196000
1	4.565102000	-0.482235000	0.711282000
1	3.419147000	-3.046883000	1.075916000
6	3.835877000	-2.370281000	-0.935385000
1	3.827803000	-1.413151000	-1.470784000
1	3.242797000	-3.069916000	-1.527100000
6	5.264756000	-2.898918000	-0.822873000
1	5.865108000	-2.300003000	-0.128628000
1	5.258875000	-3.912303000	-0.403824000
6	5.972300000	-2.937256000	-2.158795000
8	5.521238000	-2.527722000	-3.211944000
8	7.196633000	-3.487735000	-2.040317000
6	3.727910000	-0.937462000	2.640776000
1	3.836616000	0.065457000	3.072052000
1	2.821405000	-1.367551000	3.081322000
6	4.928433000	-1.756661000	3.075240000
8	5.842772000	-2.099212000	2.349491000
8	4.866017000	-2.042085000	4.384451000
6	7.409322000	4.895570000	-2.468747000
1	7.339998000	5.984299000	-2.398155000
1	8.385076000	4.550952000	-2.127216000
1	7.234267000	4.589076000	-3.503374000
6	7.968447000	-3.567181000	-3.254335000
1	8.912445000	-4.033089000	-2.971918000
1	7.448720000	-4.175798000	-3.999352000
1	8.141875000	-2.568380000	-3.664200000

6	5.979554000	-2.789754000	4.919242000
1	6.910264000	-2.232094000	4.787162000
1	5.758803000	-2.923709000	5.977672000
1	6.062103000	-3.757132000	4.417263000
6	-5.487241000	0.124969000	-4.713441000
1	-4.961792000	0.868951000	-5.318040000
1	-5.308419000	-0.879399000	-5.095541000
1	-6.556515000	0.349937000	-4.709622000
6	-7.136826000	-2.408647000	-2.985667000
1	-7.727619000	-2.936828000	-2.233293000
1	-7.143114000	-1.338184000	-2.770761000
1	-7.523087000	-2.604326000	-3.985184000

c) Optimized reduced (doublet) and oxidized (triplet and singlet) state of **Pentamethyl ester F430M** in solvent DMF.

Pentamethyl ester F430M

Pentamethyl-ester-F430M-reduced-state-doublet-DMF
reduced-state-doublet.gjf.log

Temperature 298.150 Kelvin. Pressure 1.00000 Atm.

Zero-point correction= 1.066192 (Hartree/Particle)
Thermal correction to Energy= 1.133022
Thermal correction to Enthalpy= 1.133966
Thermal correction to Gibbs Free Energy= 0.955282
Sum of electronic and zero-point Energies= -3302.801944
Sum of electronic and thermal Energies= -3302.735114
Sum of electronic and thermal Enthalpies= -3302.734170
Sum of electronic and thermal Free Energies= -3302.912854

28	0.537868000	0.228828000	-0.571189000
7	-1.382974000	-0.580726000	-0.540379000
7	-0.165742000	2.094198000	-0.273467000
7	2.518388000	0.914039000	-0.922729000
7	1.219688000	-1.646138000	-0.484002000
6	-1.745175000	-1.877386000	-0.694093000
6	-3.259279000	-2.051732000	-0.636358000
6	-2.503085000	0.199119000	-0.414215000
6	-3.771555000	-0.598910000	-0.640364000
6	2.653490000	-1.885935000	-0.240191000
6	0.570792000	-2.775565000	-0.522711000
6	1.428628000	-3.990447000	-0.225598000
6	2.851614000	-3.415995000	-0.071630000

6	-0.837335000	-2.950315000	-0.794197000
6	3.546002000	0.217388000	-1.268755000
6	2.937710000	2.320401000	-0.718449000
6	4.459568000	2.228655000	-0.445374000
6	4.835695000	1.046088000	-1.398804000
6	3.482372000	-1.276893000	-1.383074000
6	0.651499000	3.319423000	-0.197028000
6	-1.423938000	2.424052000	-0.041257000
6	-0.171798000	4.347178000	0.650623000
6	-1.615245000	3.907095000	0.259198000
6	2.072861000	3.040700000	0.306969000
6	-2.530420000	1.541073000	-0.134007000
1	2.548778000	4.001831000	0.534531000
1	2.008220000	2.472652000	1.237916000
1	-1.849008000	4.379662000	-0.706951000
6	-2.736745000	4.296505000	1.240462000
1	-2.443731000	5.202310000	1.780490000
1	-2.871159000	3.519142000	2.000374000
6	-4.071974000	4.596909000	0.551179000
1	-3.953465000	5.424088000	-0.161807000
1	-4.445416000	3.755148000	-0.040844000
6	-5.154284000	4.993565000	1.533431000
8	-4.992623000	5.187211000	2.723449000
8	-6.343753000	5.119111000	0.914568000
1	-3.513558000	1.973813000	0.000952000
6	0.098954000	4.250495000	2.152104000
1	1.143949000	4.480245000	2.379660000
1	-0.517434000	4.962574000	2.707775000
1	-0.121190000	3.245120000	2.528713000
6	0.179865000	5.704669000	0.023622000
7	0.687290000	4.018347000	-1.493678000
6	0.470902000	5.363584000	-1.439165000
8	0.504863000	6.137013000	-2.390301000
1	2.806485000	2.847672000	-1.670137000
1	4.956318000	3.149981000	-0.770717000
6	4.831170000	2.015059000	1.038038000
1	5.923403000	1.986116000	1.122869000
1	4.512626000	2.896173000	1.605568000
6	4.253265000	0.763028000	1.706491000
1	3.159274000	0.740356000	1.645126000
1	4.588241000	-0.153523000	1.217685000
6	4.611394000	0.632805000	3.168258000
8	5.056670000	1.506816000	3.885773000
8	4.349488000	-0.622624000	3.593293000
6	6.141587000	0.312776000	-1.055077000
1	6.395170000	-0.417517000	-1.829921000

1	6.962838000	1.036274000	-1.004375000
1	6.099252000	-0.226964000	-0.108853000
6	4.967517000	1.604768000	-2.857676000
1	5.878810000	2.210258000	-2.905064000
1	4.125643000	2.259899000	-3.095453000
6	4.959168000	0.514837000	-3.921679000
8	3.925827000	-0.096409000	-4.204758000
7	6.144882000	0.254521000	-4.524464000
1	6.190029000	-0.467505000	-5.231552000
1	6.993305000	0.750608000	-4.294358000
1	3.046521000	-1.559930000	-2.346945000
1	4.490423000	-1.689699000	-1.343812000
1	2.915280000	-1.373830000	0.688476000
1	-0.617306000	6.449819000	0.097763000
1	1.081883000	6.139063000	0.473242000
1	0.891499000	3.549284000	-2.366393000
1	3.501008000	-3.799941000	-0.866102000
6	3.460609000	-3.778365000	1.288528000
1	3.523195000	-4.866810000	1.412714000
1	2.814667000	-3.420550000	2.101462000
6	4.841018000	-3.202260000	1.526756000
8	5.473867000	-2.523089000	0.736647000
8	5.301970000	-3.533795000	2.743936000
1	1.088361000	-4.419973000	0.729212000
6	1.202806000	-5.043091000	-1.310013000
6	-1.288724000	-4.293487000	-1.190162000
6	-0.272981000	-5.443045000	-1.275252000
1	1.468338000	-4.621113000	-2.288553000
1	1.844323000	-5.917095000	-1.147250000
1	-0.458131000	-6.065170000	-0.386560000
1	-0.557364000	-6.052606000	-2.138940000
8	-2.460679000	-4.577136000	-1.476521000
1	-4.486704000	-0.415633000	0.168906000
1	-3.594062000	-2.609988000	-1.511981000
6	-3.699373000	-2.824108000	0.626653000
1	-3.546295000	-2.193024000	1.510945000
1	-3.079233000	-3.711994000	0.756127000
6	-5.163371000	-3.253784000	0.536968000
1	-5.833995000	-2.403580000	0.361295000
1	-5.304900000	-3.926965000	-0.318668000
6	-5.643658000	-3.974023000	1.777216000
8	-4.976833000	-4.203330000	2.768616000
8	-6.933957000	-4.343257000	1.649907000
6	-4.460405000	-0.193777000	-1.968176000
1	-4.706378000	0.871846000	-1.941903000
1	-3.795301000	-0.384913000	-2.814738000

6	-5.748827000	-0.971791000	-2.113051000
8	-6.741140000	-0.783778000	-1.432214000
8	-5.651930000	-1.951651000	-3.029400000
6	-7.448874000	5.516247000	1.750642000
1	-7.255202000	6.492960000	2.202247000
1	-8.313581000	5.566238000	1.089253000
1	-7.613769000	4.779476000	2.541392000
6	-6.808556000	-2.805883000	-3.159568000
1	-7.683978000	-2.219606000	-3.449289000
1	-6.553178000	-3.526099000	-3.935946000
1	-7.009929000	-3.315233000	-2.213446000
6	-7.500899000	-5.043421000	2.774446000
1	-8.531896000	-5.260704000	2.495651000
1	-6.952566000	-5.970112000	2.964632000
1	-7.470823000	-4.417123000	3.670212000
6	6.606575000	-3.017439000	3.079683000
1	6.807669000	-3.368841000	4.091170000
1	7.357989000	-3.398169000	2.383345000
1	6.595831000	-1.926007000	3.046481000
6	4.592917000	-0.893524000	4.987029000
1	3.925362000	-0.295667000	5.613411000
1	4.389809000	-1.956520000	5.112363000
1	5.631010000	-0.669138000	5.244725000

Triplet

Pentamethyl-ester-F430M-oxidized-state-triplet-DMF

oxidized-state-triplet.gjf.log

Temperature 298.150 Kelvin. Pressure 1.00000 Atm.

Zero-point correction= 1.067636 (Hartree/Particle)
Thermal correction to Energy= 1.134368
Thermal correction to Enthalpy= 1.135312
Thermal correction to Gibbs Free Energy= 0.955567
Sum of electronic and zero-point Energies= -3302.669811
Sum of electronic and thermal Energies= -3302.603078
Sum of electronic and thermal Enthalpies= -3302.602134
Sum of electronic and thermal Free Energies= -3302.781880

28	0.505218000	0.210979000	-0.576911000
7	-1.354061000	-0.681588000	-0.537246000
7	-0.291613000	2.063048000	-0.234987000
7	2.440386000	1.002358000	-0.893580000
7	1.295029000	-1.637997000	-0.506340000
6	-1.651772000	-2.001352000	-0.693692000
6	-3.153497000	-2.236142000	-0.613559000

6	-2.515332000	0.043727000	-0.423451000
6	-3.726617000	-0.812454000	-0.713091000
6	2.743217000	-1.797849000	-0.281287000
6	0.695179000	-2.791415000	-0.549716000
6	1.618454000	-3.959320000	-0.276895000
6	3.016162000	-3.318465000	-0.147834000
6	-0.704277000	-3.029988000	-0.797735000
6	3.491121000	0.366609000	-1.289193000
6	2.782098000	2.430135000	-0.672024000
6	4.311454000	2.410466000	-0.439389000
6	4.723495000	1.273426000	-1.434282000
6	3.514481000	-1.125105000	-1.426551000
6	0.470437000	3.322458000	-0.108635000
6	-1.563225000	2.309419000	0.006787000
6	-0.407563000	4.260382000	0.785695000
6	-1.826863000	3.763536000	0.368534000
6	1.902442000	3.082808000	0.384037000
6	-2.620688000	1.373586000	-0.120209000
1	2.340987000	4.054506000	0.636027000
1	1.870661000	2.485868000	1.299018000
1	-2.079034000	4.254790000	-0.583263000
6	-2.972090000	4.052604000	1.356639000
1	-2.723119000	4.946833000	1.935614000
1	-3.070962000	3.238783000	2.082789000
6	-4.317285000	4.319566000	0.672812000
1	-4.232492000	5.175516000	-0.010218000
1	-4.657003000	3.484509000	0.051737000
6	-5.413584000	4.637769000	1.668844000
8	-5.253057000	4.812550000	2.861782000
8	-6.610009000	4.716515000	1.057974000
1	-3.626300000	1.751913000	0.003227000
6	-0.132811000	4.105999000	2.281478000
1	0.897194000	4.384355000	2.522072000
1	-0.788563000	4.755708000	2.867014000
1	-0.296174000	3.073749000	2.611166000
6	-0.130071000	5.662695000	0.224213000
7	0.466057000	4.076369000	-1.370166000
6	0.181872000	5.408341000	-1.251128000
8	0.180947000	6.223800000	-2.164075000
1	2.600597000	2.960074000	-1.612501000
1	4.746741000	3.363534000	-0.758908000
6	4.738673000	2.186479000	1.026822000
1	5.832690000	2.216116000	1.075599000
1	4.392535000	3.037738000	1.622763000
6	4.252567000	0.891663000	1.686245000
1	3.159026000	0.819634000	1.680819000

1	4.608453000	0.004168000	1.159673000
6	4.687052000	0.744207000	3.126824000
8	5.126701000	1.622876000	3.841239000
8	4.501353000	-0.530210000	3.532060000
6	6.076608000	0.603899000	-1.148164000
1	6.349812000	-0.082981000	-1.955391000
1	6.854189000	1.373132000	-1.094388000
1	6.092001000	0.033301000	-0.219395000
6	4.780713000	1.873324000	-2.881127000
1	5.668405000	2.510960000	-2.940545000
1	3.910453000	2.505935000	-3.073775000
6	4.766950000	0.808474000	-3.970330000
8	3.757244000	0.131100000	-4.181078000
7	5.912548000	0.647939000	-4.672304000
1	5.943460000	-0.039964000	-5.413574000
1	6.732974000	1.214292000	-4.513556000
1	3.082288000	-1.414679000	-2.389311000
1	4.545540000	-1.475786000	-1.409247000
1	2.985803000	-1.290614000	0.654943000
1	-0.967765000	6.357279000	0.330830000
1	0.745150000	6.123786000	0.698630000
1	0.698651000	3.665920000	-2.265085000
1	3.658577000	-3.653373000	-0.969042000
6	3.680643000	-3.678174000	1.187013000
1	3.801209000	-4.764111000	1.281841000
1	3.041396000	-3.371744000	2.025806000
6	5.035522000	-3.033796000	1.397195000
8	5.587473000	-2.276928000	0.616346000
8	5.569105000	-3.395833000	2.572611000
1	1.314060000	-4.399765000	0.684273000
6	1.416870000	-5.019967000	-1.359180000
6	-1.105932000	-4.411996000	-1.143259000
6	-0.033145000	-5.500176000	-1.282054000
1	1.633421000	-4.585008000	-2.343450000
1	2.108695000	-5.856705000	-1.213131000
1	-0.156902000	-6.145576000	-0.400140000
1	-0.313051000	-6.109069000	-2.147378000
8	-2.274735000	-4.758061000	-1.338483000
1	-4.517906000	-0.632798000	0.019619000
1	-3.474257000	-2.877043000	-1.435286000
6	-3.514377000	-2.927713000	0.722738000
1	-3.382438000	-2.213334000	1.544492000
1	-2.827581000	-3.755556000	0.908009000
6	-4.945634000	-3.461591000	0.716144000
1	-5.668461000	-2.684922000	0.441913000
1	-5.053749000	-4.243995000	-0.045354000

6	-5.353772000	-4.040665000	2.051836000
8	-4.675480000	-4.042517000	3.062257000
8	-6.594164000	-4.564617000	1.991832000
6	-4.281094000	-0.480180000	-2.111109000
1	-4.493163000	0.594161000	-2.184787000
1	-3.550437000	-0.713390000	-2.892011000
6	-5.580067000	-1.209694000	-2.392982000
8	-6.389966000	-1.543396000	-1.547489000
8	-5.748247000	-1.417843000	-3.707502000
6	-7.729978000	5.040478000	1.906558000
1	-7.582373000	6.015678000	2.378178000
1	-8.598807000	5.061665000	1.249157000
1	-7.854218000	4.279386000	2.681439000
6	-6.985050000	-2.057070000	-4.090402000
1	-7.838955000	-1.448392000	-3.782203000
1	-6.945527000	-2.141589000	-5.175834000
1	-7.058393000	-3.045299000	-3.629367000
6	-7.092781000	-5.139511000	3.215534000
1	-8.090582000	-5.509821000	2.980706000
1	-6.447984000	-5.959075000	3.544232000
1	-7.141339000	-4.382040000	4.002591000
6	6.856432000	-2.818841000	2.879802000
1	7.117864000	-3.199802000	3.866334000
1	7.597490000	-3.127767000	2.138446000
1	6.783088000	-1.729555000	2.893440000
6	4.816928000	-0.819672000	4.908392000
1	4.149570000	-0.267221000	5.575242000
1	4.667947000	-1.893365000	5.016925000
1	5.853572000	-0.552241000	5.127464000

Singlet

Pentamethyl-ester-F430M-oxidized-state-singlet-DMF

oxidized-state-singlet.gjf.log

Temperature 298.150 Kelvin. Pressure 1.00000 Atm.

Zero-point correction= 1.070197 (Hartree/Particle)
Thermal correction to Energy= 1.136174
Thermal correction to Enthalpy= 1.137118
Thermal correction to Gibbs Free Energy= 0.961122
Sum of electronic and zero-point Energies= -3302.669796
Sum of electronic and thermal Energies= -3302.603819
Sum of electronic and thermal Enthalpies= -3302.602875
Sum of electronic and thermal Free Energies= -3302.778871

28	-0.458038000	0.085525000	0.497005000

7	1.332752000	-0.691488000	0.462879000
7	0.253099000	1.937633000	0.475294000
7	-2.284448000	0.718169000	0.985228000
7	-1.165876000	-1.623827000	0.018091000
6	1.694843000	-2.000090000	0.320603000
6	3.197232000	-2.171411000	0.455040000
6	2.475128000	0.047112000	0.721276000
6	3.619313000	-0.848997000	1.109245000
6	-2.626743000	-1.779675000	-0.171806000
6	-0.555723000	-2.750926000	-0.239346000
6	-1.462576000	-3.799589000	-0.841047000
6	-2.876778000	-3.216504000	-0.681854000
6	0.823848000	-3.037442000	-0.009384000
6	-3.273500000	-0.011125000	1.391254000
6	-2.673915000	2.150878000	1.088388000
6	-4.217856000	2.128239000	1.099036000
6	-4.463916000	0.808903000	1.899901000
6	-3.305458000	-1.481318000	1.163769000
6	-0.523642000	3.204606000	0.436082000
6	1.521147000	2.262172000	0.311417000
6	0.299335000	4.189223000	-0.448241000
6	1.746217000	3.703535000	-0.109515000
6	-1.973988000	2.985511000	0.036014000
6	2.598812000	1.391990000	0.588561000
1	-2.464038000	3.961281000	-0.038807000
1	-2.021418000	2.505442000	-0.943705000
1	2.096974000	4.240192000	0.784129000
6	2.774328000	3.954365000	-1.230213000
1	2.595131000	4.966884000	-1.605946000
1	2.594035000	3.277078000	-2.071518000
6	4.248702000	3.884435000	-0.819652000
1	4.413347000	4.385176000	0.144414000
1	4.606512000	2.858994000	-0.698101000
6	5.152497000	4.560967000	-1.834547000
8	4.785654000	5.281978000	-2.742588000
8	6.442929000	4.269778000	-1.590576000
1	3.581260000	1.821445000	0.720694000
6	-0.056279000	4.102472000	-1.932615000
1	-1.098797000	4.390374000	-2.096262000
1	0.563650000	4.780700000	-2.524112000
1	0.084317000	3.086981000	-2.319206000
6	0.028335000	5.561162000	0.188816000
7	-0.441454000	3.890984000	1.735486000
6	-0.187553000	5.233104000	1.665580000
8	-0.143845000	6.002030000	2.616273000
1	-2.348665000	2.507335000	2.068990000

1	-4.586043000	2.986012000	1.672034000
6	-4.884386000	2.198425000	-0.290715000
1	-5.970812000	2.198235000	-0.151304000
1	-4.645007000	3.168421000	-0.739332000
6	-4.514034000	1.091886000	-1.281392000
1	-3.434635000	1.054382000	-1.462448000
1	-4.787560000	0.100767000	-0.913977000
6	-5.168641000	1.245868000	-2.635680000
8	-5.770477000	2.220738000	-3.039068000
8	-4.977271000	0.126118000	-3.364779000
6	-5.843028000	0.161364000	1.701065000
1	-5.976645000	-0.687230000	2.379436000
1	-6.620505000	0.895655000	1.935615000
1	-6.010491000	-0.201791000	0.686372000
6	-4.287560000	1.107417000	3.427971000
1	-5.155584000	1.687946000	3.755655000
1	-3.399479000	1.719820000	3.604961000
6	-4.091034000	-0.155567000	4.256773000
8	-3.077375000	-0.846825000	4.120732000
7	-5.078551000	-0.469386000	5.125120000
1	-4.979702000	-1.291201000	5.707002000
1	-5.899779000	0.104963000	5.247553000
1	-2.795854000	-2.008567000	1.975862000
1	-4.336803000	-1.828970000	1.133232000
1	-2.945359000	-1.051700000	-0.917839000
1	0.844769000	6.276917000	0.061676000
1	-0.883049000	6.022273000	-0.211710000
1	-0.615198000	3.435972000	2.622012000
1	-3.421784000	-3.783381000	0.080534000
6	-3.672431000	-3.256542000	-1.991493000
1	-3.721958000	-4.279454000	-2.382753000
1	-3.169703000	-2.660193000	-2.764764000
6	-5.087136000	-2.729872000	-1.865550000
8	-5.560884000	-2.211808000	-0.867837000
8	-5.773500000	-2.898345000	-3.003525000
1	-1.198603000	-3.866863000	-1.907776000
6	-1.198546000	-5.162691000	-0.203970000
6	1.279700000	-4.445520000	-0.103841000
6	0.262231000	-5.532985000	-0.452502000
1	-1.407364000	-5.109835000	0.872309000
1	-1.867646000	-5.917852000	-0.630134000
1	0.407895000	-5.751715000	-1.520982000
1	0.561246000	-6.433578000	0.091743000
8	2.447276000	-4.796627000	0.076540000
1	4.566372000	-0.473123000	0.715850000
1	3.423647000	-3.041361000	1.069323000

6	3.844416000	-2.358353000	-0.938715000
1	3.833983000	-1.399945000	-1.471874000
1	3.255269000	-3.059301000	-1.532714000
6	5.274927000	-2.881589000	-0.822743000
1	5.872995000	-2.277209000	-0.131627000
1	5.271258000	-3.892640000	-0.397402000
6	5.984716000	-2.930046000	-2.156794000
8	5.523336000	-2.560330000	-3.220513000
8	7.224343000	-3.442119000	-2.025073000
6	3.723954000	-0.936260000	2.641608000
1	3.832579000	0.065450000	3.075459000
1	2.814762000	-1.365449000	3.077546000
6	4.919837000	-1.758773000	3.080725000
8	5.832072000	-2.113665000	2.357531000
8	4.857278000	-2.033663000	4.392351000
6	7.404527000	4.886153000	-2.471200000
1	7.333848000	5.975452000	-2.411446000
1	8.379670000	4.546485000	-2.123087000
1	7.233963000	4.568918000	-3.503353000
6	7.998732000	-3.531994000	-3.237035000
1	8.955677000	-3.962679000	-2.942698000
1	7.497228000	-4.175509000	-3.964994000
1	8.143687000	-2.539934000	-3.673326000
6	5.966135000	-2.783993000	4.932708000
1	6.900381000	-2.233421000	4.795902000
1	5.745360000	-2.907778000	5.992350000
1	6.042012000	-3.756303000	4.439242000
6	-5.497617000	0.137060000	-4.709085000
1	-4.974846000	0.884575000	-5.311632000
1	-5.315721000	-0.865317000	-5.094749000
1	-6.567811000	0.357605000	-4.703564000
6	-7.131735000	-2.408260000	-2.993737000
1	-7.722787000	-2.946577000	-2.248752000
1	-7.141908000	-1.340258000	-2.767299000
1	-7.515179000	-2.593927000	-3.996224000

d) Optimized reduced (doublet) and oxidized (triplet and singlet) state of **Pentamethyl ester F430M** in solvent CH_3CN.

Pentamethyl ester F430M

Pentamethyl-ester-F430M-reduced-state-doublet-CH3CN
reduced-state-doublet.gjf.log

Temperature 298.150 Kelvin. Pressure 1.00000 Atm.

Zero-point correction= 1.066191 (Hartree/Particle)
Thermal correction to Energy= 1.133021
Thermal correction to Enthalpy= 1.133966
Thermal correction to Gibbs Free Energy= 0.955287
Sum of electronic and zero-point Energies= -3302.801877
Sum of electronic and thermal Energies= -3302.735047
Sum of electronic and thermal Enthalpies= -3302.734102
Sum of electronic and thermal Free Energies= -3302.912781

28	0.537878000	0.228823000	-0.571053000
7	-1.382972000	-0.580739000	-0.540147000
7	-0.165655000	2.094132000	-0.273191000
7	2.518416000	0.914044000	-0.922646000
7	1.219651000	-1.646128000	-0.483921000
6	-1.745194000	-1.877357000	-0.694057000
6	-3.259313000	-2.051700000	-0.636344000
6	-2.503049000	0.199104000	-0.413792000
6	-3.771559000	-0.598850000	-0.639994000
6	2.653443000	-1.885936000	-0.240148000
6	0.570748000	-2.775552000	-0.522675000
6	1.428565000	-3.990431000	-0.225480000
6	2.851568000	-3.416009000	-0.071599000
6	-0.837360000	-2.950288000	-0.794239000
6	3.545962000	0.217351000	-1.268778000
6	2.937790000	2.320388000	-0.718472000
6	4.459657000	2.228597000	-0.445465000
6	4.835699000	1.046002000	-1.398903000
6	3.482300000	-1.276925000	-1.383065000
6	0.651574000	3.319378000	-0.197021000
6	-1.423835000	2.424010000	-0.040883000
6	-0.171649000	4.347254000	0.650558000
6	-1.615117000	3.907102000	0.259313000
6	2.072986000	3.040773000	0.306921000
6	-2.530326000	1.541029000	-0.133408000
1	2.548872000	4.001955000	0.534340000
1	2.008435000	2.472835000	1.237943000
1	-1.848921000	4.379476000	-0.706923000
6	-2.736571000	4.296732000	1.240547000
1	-2.443529000	5.202649000	1.780375000
1	-2.870968000	3.519536000	2.000631000
6	-4.071821000	4.597002000	0.551250000
1	-3.953347000	5.424083000	-0.161859000
1	-4.445252000	3.755149000	-0.040652000
6	-5.154119000	4.993783000	1.533470000
8	-4.992427000	5.187710000	2.723429000

8	-6.343614000	5.119167000	0.914603000
1	-3.513439000	1.973768000	0.001742000
6	0.099236000	4.250789000	2.152027000
1	1.144249000	4.480578000	2.379460000
1	-0.517110000	4.962938000	2.707655000
1	-0.120868000	3.245466000	2.528798000
6	0.179973000	5.704647000	0.023315000
7	0.687235000	4.018098000	-1.493780000
6	0.470690000	5.363327000	-1.439486000
8	0.504714000	6.136634000	-2.390706000
1	2.806544000	2.847617000	-1.670182000
1	4.956435000	3.149903000	-0.770828000
6	4.831293000	2.015009000	1.037949000
1	5.923530000	1.986002000	1.122743000
1	4.512814000	2.896164000	1.605451000
6	4.253304000	0.763046000	1.706439000
1	3.159309000	0.740479000	1.645014000
1	4.588224000	-0.153552000	1.217697000
6	4.611231000	0.632936000	3.168267000
8	5.055874000	1.507187000	3.885890000
8	4.349885000	-0.622631000	3.593208000
6	6.141557000	0.312625000	-1.055148000
1	6.395119000	-0.417674000	-1.829990000
1	6.962838000	1.036088000	-1.004412000
1	6.099165000	-0.227109000	-0.108921000
6	4.967548000	1.604651000	-2.857775000
1	5.878876000	2.210095000	-2.905174000
1	4.125711000	2.259824000	-3.095567000
6	4.959115000	0.514692000	-3.921759000
8	3.925760000	-0.096557000	-4.204727000
7	6.144816000	0.254251000	-4.524546000
1	6.189800000	-0.467535000	-5.231888000
1	6.993144000	0.750726000	-4.294932000
1	3.046440000	-1.559958000	-2.346935000
1	4.490346000	-1.689744000	-1.343805000
1	2.915292000	-1.373825000	0.688499000
1	-0.617147000	6.449847000	0.097487000
1	1.082093000	6.139058000	0.472715000
1	0.891369000	3.548884000	-2.366430000
1	3.500910000	-3.799953000	-0.866114000
6	3.460676000	-3.778388000	1.288515000
1	3.523308000	-4.866832000	1.412674000
1	2.814788000	-3.420620000	2.101515000
6	4.841114000	-3.202294000	1.526632000
8	5.474007000	-2.523311000	0.736404000
8	5.302073000	-3.533667000	2.743868000

1	1.088306000	-4.419799000	0.729411000
6	1.202693000	-5.043242000	-1.309707000
6	-1.288743000	-4.293404000	-1.190406000
6	-0.273124000	-5.443097000	-1.274909000
1	1.468251000	-4.621454000	-2.288323000
1	1.844150000	-5.917265000	-1.146791000
1	-0.458376000	-6.064794000	-0.385931000
1	-0.557472000	-6.053031000	-2.138341000
8	-2.460612000	-4.576871000	-1.477277000
1	-4.486655000	-0.415734000	0.169354000
1	-3.594083000	-2.609711000	-1.512125000
6	-3.699430000	-2.824367000	0.626463000
1	-3.546310000	-2.193524000	1.510922000
1	-3.079334000	-3.712313000	0.755730000
6	-5.163437000	-3.253985000	0.536706000
1	-5.834069000	-2.403737000	0.361301000
1	-5.305000000	-3.926922000	-0.319117000
6	-5.643657000	-3.974597000	1.776771000
8	-4.976786000	-4.204219000	2.768055000
8	-6.933969000	-4.343811000	1.649399000
6	-4.460510000	-0.193386000	-1.967681000
1	-4.706361000	0.872258000	-1.941145000
1	-3.795510000	-0.384415000	-2.814345000
6	-5.749066000	-0.971188000	-2.112523000
8	-6.741293000	-0.783025000	-1.431618000
8	-5.652369000	-1.951083000	-3.028882000
6	-7.448730000	5.516466000	1.750600000
1	-7.255027000	6.493234000	2.202081000
1	-8.313420000	5.566398000	1.089178000
1	-7.613675000	4.779806000	2.541451000
6	-6.809120000	-2.805119000	-3.159030000
1	-7.684502000	-2.218701000	-3.448601000
1	-6.553919000	-3.525289000	-3.935518000
1	-7.010492000	-3.314570000	-2.212954000
6	-7.500859000	-5.044380000	2.773702000
1	-8.531744000	-5.261940000	2.494690000
1	-6.952256000	-5.970930000	2.963817000
1	-7.471146000	-4.418241000	3.669600000
6	6.606776000	-3.017446000	3.079442000
1	6.807913000	-3.368794000	4.090944000
1	7.358076000	-3.398339000	2.383062000
1	6.596184000	-1.926012000	3.046142000
6	4.593088000	-0.893462000	4.987013000
1	3.924887000	-0.296139000	5.613221000
1	4.390719000	-1.956620000	5.112168000
1	5.630938000	-0.668324000	5.245036000

Triplet

Pentamethyl-ester-F430M-oxidized-state-triplet-CH3CN

oxidized-state-triplet.gjf.log

Temperature 298.150 Kelvin. Pressure 1.00000 Atm.

Zero-point correction= 1.068379 (Hartree/Particle)
Thermal correction to Energy= 1.135004
Thermal correction to Enthalpy= 1.135948
Thermal correction to Gibbs Free Energy= 0.956857
Sum of electronic and zero-point Energies= -3302.667148
Sum of electronic and thermal Energies= -3302.600523
Sum of electronic and thermal Enthalpies= -3302.599579
Sum of electronic and thermal Free Energies= -3302.778669

28	0.494063000	0.149163000	-0.577744000
7	-1.437959000	-0.591930000	-0.492893000
7	-0.155041000	2.075420000	-0.349735000
7	2.484201000	0.759978000	-0.935953000
7	1.128052000	-1.753296000	-0.418241000
6	-1.837132000	-1.887413000	-0.613073000
6	-3.350130000	-2.011558000	-0.520266000
6	-2.534610000	0.224584000	-0.369903000
6	-3.822921000	-0.547020000	-0.546396000
6	2.557468000	-2.025676000	-0.181553000
6	0.438802000	-2.856051000	-0.447499000
6	1.261420000	-4.089273000	-0.140178000
6	2.704032000	-3.559536000	-0.004250000
6	-0.971769000	-2.986628000	-0.710596000
6	3.479715000	0.020243000	-1.290354000
6	2.933727000	2.170386000	-0.824841000
6	4.459139000	2.052144000	-0.598444000
6	4.778221000	0.812919000	-1.497722000
6	3.384281000	-1.471421000	-1.349479000
6	0.701718000	3.279152000	-0.318255000
6	-1.399198000	2.431877000	-0.102232000
6	-0.088822000	4.340967000	0.518045000
6	-1.547972000	3.923797000	0.157789000
6	2.116932000	2.966963000	0.181395000
6	-2.526566000	1.572708000	-0.137658000
1	2.630138000	3.918998000	0.355655000
1	2.050091000	2.446069000	1.139326000
1	-1.781582000	4.362864000	-0.823845000
6	-2.649130000	4.368393000	1.138317000
1	-2.324479000	5.282184000	1.644948000

1	-2.791770000	3.620097000	1.925092000
6	-3.985244000	4.682210000	0.456631000
1	-3.855835000	5.485703000	-0.280814000
1	-4.389734000	3.834417000	-0.105588000
6	-5.040819000	5.133385000	1.445234000
8	-4.851034000	5.356948000	2.625674000
8	-6.236844000	5.268556000	0.843552000
1	-3.494950000	2.033400000	0.001759000
6	0.200514000	4.272067000	2.017682000
1	1.251915000	4.489871000	2.225642000
1	-0.395937000	5.007819000	2.563399000
1	-0.031817000	3.280349000	2.422087000
6	0.280925000	5.675873000	-0.145412000
7	0.737338000	3.941698000	-1.628696000
6	0.555072000	5.296458000	-1.601197000
8	0.605333000	6.044281000	-2.568936000
1	2.783096000	2.637805000	-1.803512000
1	4.962616000	2.941401000	-0.993323000
6	4.884416000	1.922057000	0.878491000
1	5.975960000	1.852343000	0.921046000
1	4.619681000	2.858155000	1.384796000
6	4.290402000	0.744066000	1.685199000
1	3.199958000	0.763252000	1.685622000
1	4.611573000	-0.213307000	1.266866000
6	4.741237000	0.798820000	3.127072000
8	4.042110000	1.104024000	4.073360000
8	6.048842000	0.484113000	3.239858000
6	6.071498000	0.060471000	-1.148254000
1	6.287391000	-0.710784000	-1.894018000
1	6.910259000	0.764542000	-1.152094000
1	6.037924000	-0.430358000	-0.175076000
6	4.882226000	1.292630000	-2.986787000
1	5.819491000	1.848688000	-3.090043000
1	4.066107000	1.977844000	-3.229542000
6	4.778238000	0.154262000	-3.993926000
8	3.705500000	-0.423830000	-4.187861000
7	5.917395000	-0.185769000	-4.640491000
1	5.891224000	-0.929143000	-5.326314000
1	6.790155000	0.301453000	-4.499503000
1	2.936061000	-1.773062000	-2.301318000
1	4.383598000	-1.902159000	-1.304841000
1	2.844559000	-1.503092000	0.734921000
1	-0.503435000	6.434954000	-0.082189000
1	1.193022000	6.105479000	0.287297000
1	0.935491000	3.455347000	-2.493418000
1	3.326949000	-3.963930000	-0.808895000

6	3.326984000	-3.936061000	1.344885000
1	3.402692000	-5.025654000	1.448249000
1	2.685753000	-3.599130000	2.169691000
6	4.703838000	-3.343057000	1.570827000
8	5.333445000	-2.685815000	0.758445000
8	5.152351000	-3.613056000	2.804080000
1	0.909549000	-4.485962000	0.823776000
6	0.990959000	-5.150690000	-1.206999000
6	-1.473773000	-4.328841000	-1.078954000
6	-0.495469000	-5.507387000	-1.152754000
1	1.261640000	-4.754925000	-2.194450000
1	1.607451000	-6.039005000	-1.031468000
1	-0.692397000	-6.106926000	-0.251537000
1	-0.804441000	-6.122230000	-2.003559000
8	-2.655711000	-4.566702000	-1.345074000
1	-4.510965000	-0.324902000	0.275773000
1	-3.715555000	-2.578913000	-1.377567000
6	-3.779157000	-2.747396000	0.770131000
1	-3.587184000	-2.103248000	1.636757000
1	-3.179799000	-3.648556000	0.903975000
6	-5.255468000	-3.139998000	0.723924000
1	-5.909889000	-2.276525000	0.554517000
1	-5.436822000	-3.822963000	-0.116235000
6	-5.712994000	-3.831509000	1.989609000
8	-5.014653000	-4.074766000	2.955514000
8	-7.017992000	-4.156214000	1.914820000
6	-4.530200000	-0.153546000	-1.867736000
1	-4.753263000	0.917232000	-1.860102000
1	-3.890249000	-0.377331000	-2.725340000
6	-5.839834000	-0.906486000	-1.964411000
8	-6.800418000	-0.695577000	-1.246801000
8	-5.795478000	-1.884614000	-2.883996000
6	-7.318434000	5.715273000	1.686156000
1	-7.093299000	6.698469000	2.107919000
1	-8.192167000	5.768875000	1.037156000
1	-7.487892000	5.003719000	2.498681000
6	-7.565083000	-4.829767000	3.065796000
1	-8.613306000	-5.010816000	2.828858000
1	-7.043138000	-5.774628000	3.239759000
1	-7.476739000	-4.200092000	3.955195000
6	-6.977096000	-2.710408000	-2.980599000
1	-7.845009000	-2.101237000	-3.243971000
1	-6.762110000	-3.435121000	-3.764764000
1	-7.162287000	-3.215467000	-2.029051000
6	6.599258000	0.546773000	4.572129000
1	6.081807000	-0.155795000	5.230290000

1	7.648145000	0.271230000	4.467681000
1	6.504703000	1.558743000	4.973900000
6	6.443985000	-3.056854000	3.139189000
1	7.217080000	-3.477698000	2.491164000
1	6.425628000	-1.970239000	3.028659000
1	6.619957000	-3.337576000	4.177099000

Singlet
Pentamethyl-ester-F430M-oxidized-state-singlet-CH3CN
oxidized-state-singlet.gjf.log

Temperature 298.150 Kelvin. Pressure 1.00000 Atm.

Zero-point correction= 1.070199 (Hartree/Particle)
Thermal correction to Energy= 1.136176
Thermal correction to Enthalpy= 1.137120
Thermal correction to Gibbs Free Energy= 0.961127
Sum of electronic and zero-point Energies= -3302.669681
Sum of electronic and thermal Energies= -3302.603704
Sum of electronic and thermal Enthalpies= -3302.602760
Sum of electronic and thermal Free Energies= -3302.778753

28	-0.458072000	0.085531000	0.497048000
7	1.332653000	-0.691707000	0.463021000
7	0.253217000	1.937577000	0.474945000
7	-2.284437000	0.718490000	0.985201000
7	-1.166108000	-1.623894000	0.018670000
6	1.694596000	-2.000430000	0.321073000
6	3.196991000	-2.171828000	0.455262000
6	2.475121000	0.046841000	0.720947000
6	3.619351000	-0.849220000	1.108882000
6	-2.626975000	-1.779578000	-0.171225000
6	-0.556096000	-2.751118000	-0.238552000
6	-1.463071000	-3.799789000	-0.840064000
6	-2.877209000	-3.216499000	-0.680971000
6	0.823450000	-3.037758000	-0.008509000
6	-3.273556000	-0.010611000	1.391422000
6	-2.673817000	2.151257000	1.087952000
6	-4.217779000	2.128729000	1.098542000
6	-4.463953000	0.809653000	1.899783000
6	-3.305651000	-1.480853000	1.164301000
6	-0.523398000	3.204623000	0.435557000
6	1.521307000	2.261941000	0.310881000
6	0.299674000	4.189026000	-0.448930000
6	1.746488000	3.703286000	-0.110086000
6	-1.973755000	2.985600000	0.035453000

6	2.598930000	1.391693000	0.587839000
1	-2.463689000	3.961416000	-0.039568000
1	-2.021191000	2.505365000	-0.944184000
1	2.097114000	4.239902000	0.783640000
6	2.774730000	3.954185000	-1.230616000
1	2.595545000	4.966713000	-1.606336000
1	2.594573000	3.276938000	-2.071982000
6	4.249055000	3.884267000	-0.819858000
1	4.413544000	4.384544000	0.144473000
1	4.606968000	2.858800000	-0.698749000
6	5.152955000	4.561325000	-1.834328000
8	4.786129000	5.282156000	-2.742515000
8	6.443410000	4.270810000	-1.589771000
1	3.581488000	1.821021000	0.719534000
6	-0.055815000	4.102044000	-1.933315000
1	-1.098290000	4.389997000	-2.097141000
1	0.564242000	4.780117000	-2.524859000
1	0.084754000	3.086474000	-2.319716000
6	0.028769000	5.561103000	0.187875000
7	-0.441196000	3.891208000	1.734818000
6	-0.187179000	5.233325000	1.664699000
8	-0.143455000	6.002377000	2.615265000
1	-2.348606000	2.507953000	2.068482000
1	-4.585906000	2.986695000	1.671295000
6	-4.884256000	2.198596000	-0.291257000
1	-5.970686000	2.198397000	-0.151908000
1	-4.644858000	3.168491000	-0.740080000
6	-4.513846000	1.091800000	-1.281662000
1	-3.434424000	1.054153000	-1.462503000
1	-4.787583000	0.100822000	-0.914051000
6	-5.168181000	1.245589000	-2.636111000
8	-5.769564000	2.220548000	-3.039923000
8	-4.977075000	0.125523000	-3.364829000
6	-5.843075000	0.162137000	1.701046000
1	-5.976799000	-0.686247000	2.379667000
1	-6.620532000	0.896539000	1.935321000
1	-6.010486000	-0.201338000	0.686464000
6	-4.287655000	1.108545000	3.427790000
1	-5.155723000	1.689094000	3.755326000
1	-3.399605000	1.721018000	3.604668000
6	-4.091081000	-0.154270000	4.256857000
8	-3.077395000	-0.845497000	4.120957000
7	-5.078690000	-0.468090000	5.125112000
1	-4.979802000	-1.289822000	5.707107000
1	-5.899946000	0.106236000	5.247441000
1	-2.795932000	-2.007894000	1.976463000

1	-4.337003000	-1.828474000	1.133995000
1	-2.945495000	-1.051751000	-0.917442000
1	0.845266000	6.276766000	0.060616000
1	-0.882565000	6.022221000	-0.212758000
1	-0.614971000	3.436386000	2.621434000
1	-3.422237000	-3.783146000	0.081575000
6	-3.672866000	-3.256710000	-1.990589000
1	-3.722557000	-4.279686000	-2.381660000
1	-3.170043000	-2.660584000	-2.763973000
6	-5.087481000	-2.729747000	-1.864790000
8	-5.561164000	-2.211461000	-0.867162000
8	-5.773854000	-2.898284000	-3.002736000
1	-1.199102000	-3.867251000	-1.906786000
6	-1.199231000	-5.162804000	-0.202741000
6	1.279105000	-4.445939000	-0.102590000
6	0.261504000	-5.533338000	-0.451092000
1	-1.408111000	-5.109720000	0.873517000
1	-1.868395000	-5.917973000	-0.628788000
1	0.407243000	-5.752306000	-1.519513000
1	0.560362000	-6.433854000	0.093363000
8	2.446594000	-4.797177000	0.078004000
1	4.566322000	-0.473556000	0.715077000
1	3.423481000	-3.041601000	1.069770000
6	3.843828000	-2.359293000	-0.938601000
1	3.833406000	-1.401051000	-1.472058000
1	3.254456000	-3.060336000	-1.532264000
6	5.274289000	-2.882701000	-0.822843000
1	5.872652000	-2.278180000	-0.132104000
1	5.270597000	-3.893614000	-0.397177000
6	5.983669000	-2.931692000	-2.157104000
8	5.522060000	-2.562101000	-3.220769000
8	7.223187000	-3.444054000	-2.025634000
6	3.724507000	-0.935986000	2.641239000
1	3.833132000	0.065866000	3.074768000
1	2.815545000	-1.365189000	3.077634000
6	4.920701000	-1.758180000	3.080159000
8	5.832833000	-2.112923000	2.356763000
8	4.858546000	-2.032926000	4.391817000
6	7.405073000	4.887634000	-2.470016000
1	7.333853000	5.976897000	-2.410287000
1	8.380228000	4.548414000	-2.121511000
1	7.235053000	4.570329000	-3.502236000
6	7.997154000	-3.534454000	-3.237821000
1	8.954078000	-3.965311000	-2.943680000
1	7.495236000	-4.178023000	-3.965447000
1	8.142216000	-2.542550000	-3.674430000

6	5.967745000	-2.782906000	4.931967000
1	6.901799000	-2.232076000	4.794887000
1	5.747250000	-2.906668000	5.991667000
1	6.043785000	-3.755223000	4.438546000
6	-5.497135000	0.136256000	-4.709263000
1	-4.973475000	0.882935000	-5.312075000
1	-5.316172000	-0.866513000	-5.094336000
1	-6.567099000	0.357914000	-4.704101000
6	-7.132000000	-2.407900000	-2.992993000
1	-7.722964000	-2.945452000	-2.247392000
1	-7.141853000	-1.339702000	-2.767454000
1	-7.515734000	-2.594327000	-3.995225000

e) Optimized reduced (doublet) and oxidized (triplet and singlet) state of **F430 model complex** in solvent CH_3CN.

F430 model complex

F430-model-complex-reduced-state-doublet-CH3CN
reduced-state-doublet.gjf.log

Temperature 298.150 Kelvin. Pressure 1.00000 Atm.

Zero-point correction= 0.593165 (Hartree/Particle)
Thermal correction to Energy= 0.625895
Thermal correction to Enthalpy= 0.626839
Thermal correction to Gibbs Free Energy= 0.532091
Sum of electronic and zero-point Energies= -1585.468995
Sum of electronic and thermal Energies= -1585.436265
Sum of electronic and thermal Enthalpies= -1585.435321
Sum of electronic and thermal Free Energies= -1585.530069

28	-0.024744000	-0.179293000	-0.449558000
7	-2.073163000	0.030644000	-0.183850000
7	-0.298480000	-2.185861000	-0.561110000
7	2.072544000	-0.451104000	-0.360140000
7	0.147895000	1.802490000	-0.190641000
6	-2.774568000	1.209313000	-0.298779000
6	-4.283331000	0.962123000	-0.246122000
6	-2.912380000	-0.944277000	0.209810000
6	-4.312971000	-0.423505000	0.443817000
6	1.438721000	2.491481000	0.091418000
6	-0.803995000	2.712145000	-0.268446000
6	-0.282106000	4.124189000	-0.107069000
6	1.023426000	3.898031000	0.683137000
6	-2.193793000	2.445992000	-0.407187000

6	3.012122000	0.476333000	0.325854000
6	2.631713000	-1.610363000	-0.420945000
6	3.937903000	-1.741396000	0.361407000
6	3.879033000	-0.438630000	1.198106000
6	2.324651000	1.637072000	1.024016000
6	0.684869000	-3.205542000	-1.009627000
6	-1.257905000	-2.831209000	0.062356000
6	0.577143000	-4.319037000	0.038089000
6	-0.932089000	-4.297693000	0.312743000
6	2.088330000	-2.672025000	-1.328269000
6	-2.529085000	-2.272917000	0.426344000
6	0.694143000	3.771126000	2.183463000
1	1.590402000	3.580440000	2.781161000
1	0.255590000	4.711915000	2.532916000
1	-0.026603000	2.969754000	2.376809000
6	2.069305000	4.998196000	0.479694000
1	1.706255000	5.937916000	0.909743000
1	3.010538000	4.744966000	0.981671000
1	2.281433000	5.171970000	-0.579704000
1	-0.075424000	4.559001000	-1.095629000
1	-0.988816000	4.785688000	0.402543000
1	-2.844514000	3.310514000	-0.474279000
1	-4.494167000	-0.323873000	1.522491000
1	-5.082965000	-1.092245000	0.047396000
6	-4.820621000	0.854244000	-1.691230000
1	-5.887999000	0.604913000	-1.679714000
1	-4.692374000	1.803803000	-2.222025000
1	-4.291378000	0.073891000	-2.250184000
6	-5.066214000	2.025208000	0.533803000
1	-5.049305000	2.992785000	0.020735000
1	-6.114503000	1.720670000	0.631447000
1	-4.652687000	2.162861000	1.538796000
6	-3.495724000	-3.151298000	0.987586000
7	-4.291604000	-3.870927000	1.449854000
1	-1.219676000	-4.619056000	1.318185000
1	-1.477512000	-4.932305000	-0.400440000
1	0.949796000	-5.280722000	-0.324693000
1	1.136713000	-4.043563000	0.940171000
1	2.772092000	-3.525118000	-1.373561000
1	2.066057000	-2.230761000	-2.331725000
1	3.372033000	-0.626766000	2.153135000
1	4.866408000	-0.018350000	1.409336000
6	5.129316000	-1.739602000	-0.623332000
1	6.071586000	-1.797436000	-0.067318000
1	5.081675000	-2.600620000	-1.299183000
1	5.148362000	-0.829971000	-1.233880000

6	3.975127000	-3.006314000	1.231549000
1	3.966552000	-3.917567000	0.623092000
1	4.891711000	-3.015236000	1.832531000
1	3.120796000	-3.041932000	1.916728000
1	1.683267000	1.266595000	1.829182000
1	3.097170000	2.274206000	1.468654000
1	3.639455000	0.897039000	-0.472548000
6	2.110106000	2.688157000	-1.216958000
7	2.602036000	2.834296000	-2.259242000
1	0.301519000	-3.608755000	-1.959698000

Triplet

F430-model-complex-oxidized-state-triplet-CH3CN

oxidized-state-triplet.gjf.log

Temperature 298.150 Kelvin. Pressure 1.00000 Atm.

Zero-point correction= 0.594765 (Hartree/Particle)
Thermal correction to Energy= 0.627361
Thermal correction to Enthalpy= 0.628306
Thermal correction to Gibbs Free Energy= 0.533402
Sum of electronic and zero-point Energies= -1585.335133
Sum of electronic and thermal Energies= -1585.302537
Sum of electronic and thermal Enthalpies= -1585.301593
Sum of electronic and thermal Free Energies= -1585.396496

28	-0.025589000	-0.189627000	-0.470430000
7	-2.057273000	0.017227000	-0.201459000
7	-0.283979000	-2.202693000	-0.508129000
7	2.049337000	-0.463441000	-0.327478000
7	0.142560000	1.821704000	-0.207607000
6	-2.768534000	1.187743000	-0.371969000
6	-4.269238000	0.916227000	-0.330291000
6	-2.886060000	-0.950519000	0.240638000
6	-4.285791000	-0.425711000	0.442388000
6	1.418397000	2.512786000	0.126518000
6	-0.823081000	2.709993000	-0.299670000
6	-0.327540000	4.119544000	-0.074491000
6	0.957367000	3.888695000	0.751470000
6	-2.202324000	2.426036000	-0.499117000
6	2.992044000	0.496091000	0.318001000
6	2.632150000	-1.608701000	-0.425001000
6	3.972369000	-1.698597000	0.297553000
6	3.911933000	-0.403464000	1.147443000
6	2.302001000	1.639248000	1.043748000
6	0.685090000	-3.217978000	-0.997054000

6	-1.239696000	-2.834533000	0.128414000
6	0.577608000	-4.345997000	0.035259000
6	-0.924139000	-4.303750000	0.350273000
6	2.090164000	-2.686889000	-1.312655000
6	-2.497873000	-2.263248000	0.511597000
6	0.585364000	3.703307000	2.235379000
1	1.468377000	3.511556000	2.851869000
1	0.117197000	4.622649000	2.601921000
1	-0.123726000	2.881745000	2.382471000
6	1.988138000	5.012552000	0.613915000
1	1.592967000	5.930373000	1.061467000
1	2.916978000	4.760086000	1.138021000
1	2.229273000	5.223596000	-0.432251000
1	-0.099778000	4.582034000	-1.045242000
1	-1.063619000	4.751604000	0.429078000
1	-2.859824000	3.280978000	-0.598079000
1	-4.458909000	-0.261132000	1.513970000
1	-5.049738000	-1.124655000	0.091265000
6	-4.770891000	0.707850000	-1.778696000
1	-5.832355000	0.437018000	-1.768284000
1	-4.652774000	1.626566000	-2.362488000
1	-4.215524000	-0.093230000	-2.279300000
6	-5.086620000	2.009203000	0.367326000
1	-5.085291000	2.939746000	-0.209736000
1	-6.127648000	1.683784000	0.468260000
1	-4.692686000	2.222003000	1.366663000
6	-3.457877000	-3.125349000	1.113334000
7	-4.245203000	-3.829013000	1.609151000
1	-1.192285000	-4.631053000	1.358505000
1	-1.502779000	-4.913759000	-0.357134000
1	0.919402000	-5.306822000	-0.356202000
1	1.165390000	-4.099535000	0.927463000
1	2.775902000	-3.537624000	-1.339551000
1	2.081630000	-2.258568000	-2.322030000
1	3.449481000	-0.611221000	2.120265000
1	4.895396000	0.041387000	1.318948000
6	5.117923000	-1.658885000	-0.739883000
1	6.081415000	-1.691907000	-0.220634000
1	5.066593000	-2.519408000	-1.415383000
1	5.086929000	-0.747160000	-1.346251000
6	4.076901000	-2.967525000	1.157516000
1	4.096973000	-3.875104000	0.544814000
1	5.005584000	-2.936419000	1.737822000
1	3.240418000	-3.042120000	1.861093000
1	1.668533000	1.253047000	1.848189000
1	3.074346000	2.270124000	1.495951000

1	3.573128000	0.927068000	-0.507687000
6	2.111438000	2.761838000	-1.162843000
7	2.624153000	2.942818000	-2.188789000
1	0.285027000	-3.592120000	-1.950171000

Singlet

F430-model-complex-oxidized-state-singlet-CH3CN

oxidized-state-singlet.gjf.log

Temperature 298.150 Kelvin. Pressure 1.00000 Atm.

Zero-point correction= 0.596835 (Hartree/Particle)
Thermal correction to Energy= 0.628749
Thermal correction to Enthalpy= 0.629694
Thermal correction to Gibbs Free Energy= 0.537748
Sum of electronic and zero-point Energies= -1585.339175
Sum of electronic and thermal Energies= -1585.307261
Sum of electronic and thermal Enthalpies= -1585.306317
Sum of electronic and thermal Free Energies= -1585.398263

28	-0.030035000	-0.166623000	-0.358016000
7	-1.945207000	0.026366000	-0.179382000
7	-0.263874000	-2.078882000	-0.419304000
7	1.928604000	-0.457983000	-0.236776000
7	0.156113000	1.757763000	-0.248338000
6	-2.683845000	1.145249000	-0.527690000
6	-4.171620000	0.830040000	-0.488006000
6	-2.771175000	-0.888401000	0.382723000
6	-4.163843000	-0.338735000	0.529456000
6	1.394739000	2.461428000	0.205485000
6	-0.783907000	2.656259000	-0.461891000
6	-0.304869000	4.063461000	-0.203885000
6	0.863606000	3.829095000	0.779162000
6	-2.138513000	2.371095000	-0.764536000
6	2.888318000	0.459749000	0.456183000
6	2.548752000	-1.563304000	-0.479347000
6	3.895183000	-1.690516000	0.224618000
6	3.797628000	-0.499812000	1.215991000
6	2.199873000	1.590546000	1.188242000
6	0.603016000	-3.060440000	-1.134056000
6	-1.181956000	-2.750674000	0.231534000
6	0.527740000	-4.311221000	-0.249799000
6	-0.917215000	-4.240994000	0.257374000
6	2.011812000	-2.555276000	-1.455544000
6	-2.385875000	-2.175000000	0.738977000
6	0.314964000	3.630497000	2.204964000

1	1.120978000	3.437496000	2.919019000
1	-0.198095000	4.544798000	2.519876000
1	-0.401315000	2.804015000	2.261135000
6	1.904001000	4.952545000	0.777649000
1	1.453169000	5.867070000	1.176421000
1	2.758430000	4.697530000	1.414547000
1	2.275533000	5.169922000	-0.228009000
1	0.047182000	4.512182000	-1.143059000
1	-1.093529000	4.704964000	0.196996000
1	-2.789984000	3.206832000	-0.984034000
1	-4.298027000	0.036904000	1.552449000
1	-4.936283000	-1.089147000	0.344471000
6	-4.611302000	0.321283000	-1.881594000
1	-5.662034000	0.014108000	-1.846555000
1	-4.505655000	1.113371000	-2.629734000
1	-4.011031000	-0.537967000	-2.200730000
6	-5.051944000	2.000951000	-0.041770000
1	-5.063073000	2.799126000	-0.791277000
1	-6.082974000	1.655974000	0.089910000
1	-4.703411000	2.421205000	0.907283000
6	-3.309233000	-3.008569000	1.428934000
7	-4.062460000	-3.690346000	2.000994000
1	-1.075134000	-4.661405000	1.254540000
1	-1.615831000	-4.743943000	-0.424731000
1	0.756144000	-5.222963000	-0.805781000
1	1.233146000	-4.223175000	0.584191000
1	2.683227000	-3.411579000	-1.550724000
1	2.003377000	-2.046322000	-2.426585000
1	3.318521000	-0.819044000	2.149324000
1	4.769603000	-0.059560000	1.451728000
6	5.040489000	-1.513691000	-0.798341000
1	6.003386000	-1.585749000	-0.282392000
1	5.004353000	-2.297394000	-1.562420000
1	4.995171000	-0.543448000	-1.304089000
6	4.030495000	-3.042323000	0.943232000
1	4.081320000	-3.878041000	0.237622000
1	4.955698000	-3.044068000	1.529797000
1	3.194861000	-3.215005000	1.630157000
1	1.514301000	1.207007000	1.949320000
1	2.955667000	2.208184000	1.683162000
1	3.481989000	0.909933000	-0.348610000
6	2.191718000	2.734153000	-1.019649000
7	2.778844000	2.951577000	-1.997213000
1	0.104945000	-3.270197000	-2.089141000

viii) Optimized structure of reduced and oxidized states of cofactor **A-E** in aqueous solvent and epsilon=4.

a) Optimized reduced (doublet) and oxidized (triplet and singlet) state of **A** in aqueous solvent.

A-reduced-state-doublet-H2O

Reduced-state-doublet.gjf.log

28	-0.167360000	-0.410038000	-0.663154000
7	0.524088000	1.502316000	-0.738705000
7	1.771966000	-1.119355000	-0.459803000
7	-0.870775000	-2.319187000	-0.559588000
7	-2.090097000	0.306762000	-0.662677000
6	-0.221390000	2.669265000	-0.633794000
6	0.648994000	3.818043000	-0.738023000
6	1.819308000	1.881068000	-0.883527000
6	1.924875000	3.323669000	-0.916572000
6	-3.208987000	-0.450231000	-0.570432000
6	-2.472067000	1.610422000	-0.378821000
6	-3.876982000	1.650827000	-0.041562000
6	-4.333148000	0.351500000	-0.157143000
6	-1.600349000	2.701389000	-0.420190000
6	-2.184038000	-2.704971000	-0.776299000
6	-0.167036000	-3.390079000	-0.142362000
6	-1.082938000	-4.583946000	0.101555000
6	-2.266583000	-4.229822000	-0.850091000
6	-3.253936000	-1.857738000	-0.795394000
6	2.098049000	-2.360075000	-0.050052000
6	2.911542000	-0.354638000	-0.591923000
6	3.580691000	-2.463331000	0.330494000
6	4.162547000	-1.206057000	-0.390874000
6	1.202216000	-3.416844000	0.118668000
6	2.927904000	0.988543000	-0.860601000
1	4.516313000	-1.534246000	-1.378790000
6	5.338596000	-0.514410000	0.332126000
1	5.908762000	-1.248669000	0.909004000
1	4.950146000	0.216477000	1.049884000
6	6.339160000	0.179489000	-0.621638000
1	6.796146000	-0.570642000	-1.272948000
1	5.853787000	0.936029000	-1.239735000
6	7.405071000	0.858982000	0.199353000
8	7.359993000	2.012910000	0.610274000
8	8.416328000	0.037643000	0.512069000
1	3.900218000	1.446292000	-0.982821000
6	3.661722000	-2.374966000	1.872071000
1	3.069464000	-3.180215000	2.318721000
1	4.687504000	-2.473559000	2.235692000

1	3.254868000	-1.419723000	2.220654000
1	-0.608401000	-5.527301000	-0.192244000
6	-1.455844000	-4.700582000	1.601350000
1	-2.152004000	-5.538009000	1.726784000
1	-0.552676000	-4.965166000	2.161741000
6	-2.065405000	-3.435356000	2.225842000
1	-1.346750000	-2.605125000	2.183002000
1	-2.934738000	-3.090795000	1.664827000
6	-2.452383000	-3.627066000	3.680343000
8	-1.918114000	-4.424773000	4.428070000
8	-3.435191000	-2.832148000	4.146203000
6	-3.618789000	-4.848450000	-0.483262000
1	-4.370684000	-4.620390000	-1.243407000
1	-3.531024000	-5.939202000	-0.423928000
1	-3.995457000	-4.484513000	0.475442000
6	-1.845044000	-4.703902000	-2.289760000
1	-1.984543000	-5.787311000	-2.358379000
1	-0.789229000	-4.473858000	-2.450941000
1	9.036622000	0.524105000	1.090670000
1	-3.831342000	-2.268251000	3.440119000
1	-2.042725000	3.681491000	-0.260939000
6	4.230179000	-3.780356000	-0.178018000
1	4.003917000	-3.912846000	-1.239267000
1	3.827274000	-4.631729000	0.376876000
6	5.727798000	-3.782437000	0.026007000
8	6.296914000	-4.077691000	1.059632000
1	1.601219000	-4.356136000	0.488932000
6	3.192347000	4.112748000	-1.112912000
1	3.605512000	3.963710000	-2.116541000
1	2.983742000	5.183495000	-1.006585000
6	0.253567000	5.256197000	-0.563430000
1	-0.792159000	5.417197000	-0.845413000
1	0.843817000	5.904338000	-1.221546000
6	4.269700000	3.767310000	-0.093405000
8	4.112622000	3.779979000	1.114251000
8	5.424153000	3.425143000	-0.689714000
1	6.075195000	3.074382000	-0.026331000
6	0.459316000	5.709612000	0.891467000
1	-0.156995000	5.109815000	1.573219000
1	1.497084000	5.539846000	1.205080000
6	0.121722000	7.165244000	1.113553000
8	-0.298971000	7.934665000	0.271465000
8	0.343214000	7.536167000	2.395042000
1	0.103922000	8.480339000	2.470371000
6	-4.651641000	2.876990000	0.344965000
1	-4.054447000	3.519789000	1.001941000

1	-5.543058000	2.603627000	0.920877000
6	-5.651419000	-0.235333000	0.254252000
1	-6.328539000	0.540825000	0.632952000
1	-6.180025000	-0.732914000	-0.566743000
6	-5.090039000	3.694784000	-0.880720000
1	-5.722125000	3.091901000	-1.544394000
1	-4.223500000	3.990909000	-1.484760000
6	-5.852895000	4.946226000	-0.511580000
8	-6.112756000	5.314149000	0.617574000
8	-6.229396000	5.639421000	-1.609861000
1	-6.714120000	6.430238000	-1.303124000
6	-5.468060000	-1.244717000	1.380081000
8	-4.649534000	-1.137299000	2.282488000
8	-6.309088000	-2.284505000	1.282373000
1	-6.154730000	-2.879691000	2.042305000
1	-4.238089000	-2.306055000	-0.895713000
8	6.395612000	-3.363013000	-1.074240000
1	7.347076000	-3.348598000	-0.850036000
6	-2.609722000	-4.013633000	-3.392313000
8	-2.231717000	-3.024322000	-3.990238000
8	-3.802849000	-4.599169000	-3.645083000
1	-4.244819000	-4.070020000	-4.337790000

Triplet

A-oxidized-state-triplet-H2O

Oxidized-state-triplet.gjf.log

28	-0.192444000	-0.381872000	-0.641058000
7	0.597565000	1.480565000	-0.714989000
7	1.708625000	-1.182862000	-0.411713000
7	-0.973381000	-2.271831000	-0.539669000
7	-2.049269000	0.398193000	-0.597712000
6	-0.089916000	2.680376000	-0.578987000
6	0.834701000	3.779732000	-0.703090000
6	1.904279000	1.791322000	-0.897482000
6	2.076574000	3.223404000	-0.933648000
6	-3.211771000	-0.310802000	-0.550409000
6	-2.373331000	1.719048000	-0.308126000
6	-3.774646000	1.815919000	-0.013512000
6	-4.295528000	0.538065000	-0.164765000
6	-1.457686000	2.773247000	-0.341613000
6	-2.297026000	-2.598531000	-0.804563000
6	-0.324269000	-3.366870000	-0.111337000
6	-1.288425000	-4.530403000	0.068759000
6	-2.426830000	-4.114108000	-0.915373000
6	-3.323182000	-1.705988000	-0.819108000
6	1.970201000	-2.433542000	0.024524000

6	2.886578000	-0.480508000	-0.582899000
6	3.445461000	-2.595196000	0.398856000
6	4.086400000	-1.393358000	-0.367120000
6	1.033674000	-3.446637000	0.195631000
6	2.969184000	0.847647000	-0.890387000
1	4.408112000	-1.767985000	-1.348953000
6	5.303207000	-0.743273000	0.326118000
1	5.837641000	-1.493311000	0.915905000
1	4.959800000	0.023290000	1.029015000
6	6.329973000	-0.128152000	-0.652675000
1	6.737586000	-0.916130000	-1.291553000
1	5.883115000	0.641655000	-1.283247000
6	7.439572000	0.508305000	0.146408000
8	7.451220000	1.666196000	0.547873000
8	8.416402000	-0.355666000	0.449536000
1	3.959308000	1.253126000	-1.042136000
6	3.531227000	-2.454328000	1.937504000
1	2.903070000	-3.214249000	2.412864000
1	4.552876000	-2.591672000	2.298932000
1	3.174783000	-1.468617000	2.254108000
1	-0.831263000	-5.478163000	-0.234815000
6	-1.711516000	-4.671770000	1.553559000
1	-2.441946000	-5.484875000	1.628595000
1	-0.838064000	-4.989764000	2.132268000
6	-2.291661000	-3.402132000	2.195446000
1	-1.536212000	-2.604517000	2.218522000
1	-3.121492000	-2.997689000	1.614966000
6	-2.748123000	-3.628988000	3.625791000
8	-2.281160000	-4.478263000	4.360806000
8	-3.711179000	-2.805464000	4.080908000
6	-3.812009000	-4.688282000	-0.606104000
1	-4.530353000	-4.413012000	-1.383225000
1	-3.765475000	-5.782083000	-0.574676000
1	-4.203019000	-4.334982000	0.350743000
6	-1.973599000	-4.569056000	-2.350510000
1	-2.110240000	-5.651158000	-2.431893000
1	-0.914753000	-4.336474000	-2.488393000
1	9.067906000	0.105000000	1.014940000
1	-4.051667000	-2.203878000	3.377987000
1	-1.857122000	3.768802000	-0.174227000
6	4.025773000	-3.959714000	-0.062895000
1	3.783340000	-4.124468000	-1.115862000
1	3.592070000	-4.768764000	0.530298000
6	5.525110000	-4.021237000	0.129682000
8	6.087032000	-4.306476000	1.169175000
1	1.386218000	-4.398771000	0.575852000

6	3.375281000	3.946250000	-1.171367000
1	3.763695000	3.741949000	-2.174366000
1	3.217101000	5.027696000	-1.099636000
6	0.527675000	5.235101000	-0.503738000
1	-0.528078000	5.449482000	-0.696306000
1	1.092707000	5.848467000	-1.214508000
6	4.444723000	3.580486000	-0.149777000
8	4.302136000	3.659498000	1.056817000
8	5.562613000	3.135982000	-0.744652000
1	6.205908000	2.773714000	-0.079222000
6	0.884114000	5.675099000	0.926442000
1	0.284998000	5.123824000	1.661852000
1	1.929771000	5.433343000	1.156502000
6	0.669534000	7.152961000	1.156013000
8	0.261805000	7.947671000	0.331456000
8	0.991298000	7.509519000	2.419267000
1	0.832231000	8.470005000	2.500919000
6	-4.507876000	3.072799000	0.353285000
1	-3.885568000	3.709294000	0.991314000
1	-5.400183000	2.836862000	0.942654000
6	-5.661849000	0.032989000	0.181341000
1	-6.342950000	0.852969000	0.438859000
1	-6.143923000	-0.499909000	-0.647510000
6	-4.930965000	3.873121000	-0.889359000
1	-5.582697000	3.273793000	-1.536675000
1	-4.060339000	4.134758000	-1.503120000
6	-5.659565000	5.150373000	-0.537621000
8	-5.901307000	5.542342000	0.587123000
8	-6.024694000	5.831658000	-1.645738000
1	-6.487655000	6.640491000	-1.352542000
6	-5.635265000	-0.911207000	1.377572000
8	-4.694253000	-1.039428000	2.146422000
8	-6.779927000	-1.593762000	1.500015000
1	-6.718101000	-2.164873000	2.290889000
1	-4.325139000	-2.099364000	-0.953356000
8	6.197747000	-3.669833000	-0.989874000
1	7.152179000	-3.691603000	-0.778525000
6	-2.721074000	-3.862777000	-3.455808000
8	-2.375438000	-2.815113000	-3.967004000
8	-3.856131000	-4.505954000	-3.808123000
1	-4.296622000	-3.967797000	-4.495056000

Singlet

A-oxidized-state-singlet-H2O

Oxidized-state-singlet.gjf.log

28	-0.211079000	-0.361006000	-0.426380000

7	0.613741000	1.387820000	-0.568675000
7	1.558399000	-1.197842000	-0.182905000
7	-1.044443000	-2.119455000	-0.342442000
7	-1.968986000	0.466260000	-0.512324000
6	0.017285000	2.626236000	-0.347645000
6	0.983236000	3.672017000	-0.544501000
6	1.909875000	1.638708000	-0.894087000
6	2.153333000	3.055197000	-0.936268000
6	-3.158742000	-0.166832000	-0.692811000
6	-2.275422000	1.777201000	-0.156888000
6	-3.698136000	1.933483000	-0.049319000
6	-4.248503000	0.716948000	-0.404868000
6	-1.331720000	2.792819000	-0.077398000
6	-2.299770000	-2.439296000	-0.852906000
6	-0.506686000	-3.227857000	0.200820000
6	-1.507909000	-4.368951000	0.212652000
6	-2.418967000	-3.949334000	-0.983694000
6	-3.292158000	-1.535871000	-1.038899000
6	1.772166000	-2.398391000	0.400250000
6	2.773947000	-0.623456000	-0.512904000
6	3.245684000	-2.601018000	0.739182000
6	3.914815000	-1.590456000	-0.245269000
6	0.795871000	-3.345375000	0.661896000
6	2.924247000	0.653395000	-0.957338000
1	4.132096000	-2.135398000	-1.174769000
6	5.222276000	-0.951296000	0.268275000
1	5.750005000	-1.662741000	0.909736000
1	4.987290000	-0.083279000	0.893086000
6	6.208343000	-0.539078000	-0.848023000
1	6.495441000	-1.422360000	-1.424591000
1	5.775165000	0.199167000	-1.524772000
6	7.428697000	0.073280000	-0.206688000
8	7.545611000	1.251515000	0.110002000
8	8.379121000	-0.829844000	0.061323000
1	3.914853000	0.986487000	-1.229117000
6	3.425644000	-2.214214000	2.226096000
1	2.772710000	-2.833561000	2.849208000
1	4.453993000	-2.372078000	2.558923000
1	3.158378000	-1.164700000	2.386422000
1	-1.011258000	-5.322988000	0.007094000
6	-2.206910000	-4.502274000	1.587492000
1	-2.942687000	-5.311536000	1.522124000
1	-1.459493000	-4.827266000	2.318741000
6	-2.888504000	-3.234386000	2.118213000
1	-2.159654000	-2.418979000	2.225342000
1	-3.644218000	-2.858028000	1.427008000

6	-3.524343000	-3.450553000	3.481828000
8	-3.175417000	-4.314481000	4.263977000
8	-4.511427000	-2.600950000	3.818384000
6	-3.845528000	-4.503057000	-0.956199000
1	-4.391937000	-4.216345000	-1.859173000
1	-3.820857000	-5.597321000	-0.920282000
1	-4.412125000	-4.144257000	-0.093964000
6	-1.692138000	-4.419334000	-2.295933000
1	-1.825358000	-5.499754000	-2.400354000
1	-0.623689000	-4.202164000	-2.219305000
1	9.108853000	-0.377253000	0.529524000
1	-4.738053000	-1.985308000	3.081786000
1	-1.685390000	3.795022000	0.139277000
6	3.711282000	-4.060969000	0.486987000
1	3.382510000	-4.384945000	-0.503669000
1	3.277133000	-4.725646000	1.238183000
6	5.214771000	-4.192908000	0.591485000
8	5.836414000	-4.324811000	1.627706000
1	1.081541000	-4.278482000	1.131388000
6	3.458475000	3.707753000	-1.304427000
1	3.739822000	3.475207000	-2.336267000
1	3.360514000	4.796029000	-1.231694000
6	0.785182000	5.136468000	-0.285861000
1	-0.272214000	5.413099000	-0.338390000
1	1.290102000	5.733621000	-1.053351000
6	4.598156000	3.295936000	-0.380432000
8	4.574458000	3.401960000	0.832531000
8	5.626010000	2.774986000	-1.067275000
1	6.310011000	2.391393000	-0.455753000
6	1.345679000	5.522331000	1.093758000
1	0.806823000	4.996547000	1.891709000
1	2.393185000	5.207928000	1.187935000
6	1.261460000	7.006216000	1.364467000
8	0.818196000	7.842667000	0.601864000
8	1.750366000	7.314857000	2.585862000
1	1.669284000	8.282086000	2.696939000
6	-4.410340000	3.203788000	0.312881000
1	-3.849735000	3.756081000	1.074547000
1	-5.383593000	2.978448000	0.761467000
6	-5.675314000	0.270636000	-0.320583000
1	-6.351210000	1.118348000	-0.154489000
1	-6.027646000	-0.215565000	-1.237562000
6	-4.623721000	4.112717000	-0.908566000
1	-5.214171000	3.600343000	-1.677937000
1	-3.667410000	4.362479000	-1.384045000
6	-5.325857000	5.404462000	-0.556494000

8	-5.704321000	5.726490000	0.552636000
8	-5.492255000	6.188355000	-1.644206000
1	-5.948466000	7.001260000	-1.351716000
6	-5.891538000	-0.697506000	0.834365000
8	-5.139096000	-0.811204000	1.790768000
8	-7.012159000	-1.413576000	0.691992000
1	-7.111946000	-1.995128000	1.471369000
1	-4.264894000	-1.889403000	-1.360827000
8	5.817641000	-4.097448000	-0.614903000
1	6.782959000	-4.147679000	-0.467357000
6	-2.194005000	-3.705975000	-3.528217000
8	-1.757000000	-2.649527000	-3.942489000
8	-3.222509000	-4.352378000	-4.118956000
1	-3.511773000	-3.810810000	-4.879679000

b) Optimized reduced (doublet) and oxidized (triplet and singlet) state of **A** in epsilon=4.

A-reduced-state-doublet-eps4
Reduced-state-doublet.gjf.log

Temperature 298.150 Kelvin. Pressure 1.00000 Atm.

Zero-point correction= 0.839162 (Hartree/Particle)
Thermal correction to Energy= 0.900511
Thermal correction to Enthalpy= 0.901455
Thermal correction to Gibbs Free Energy= 0.727098
Sum of electronic and zero-point Energies= -3218.519819
Sum of electronic and thermal Energies= -3218.458469
Sum of electronic and thermal Enthalpies= -3218.457525
Sum of electronic and thermal Free Energies= -3218.631882

6	5.042923000	-1.311170000	1.039976000
6	1.909202000	4.495215000	1.424308000
6	-7.563337000	0.927317000	-1.122287000
6	-0.802230000	-6.076067000	-0.924777000
7	1.859058000	-1.072866000	-0.104385000
6	6.417113000	-1.985054000	1.018140000
6	0.735404000	5.190423000	2.138020000
7	1.073110000	1.749147000	-0.139582000
8	-8.290252000	0.988778000	-0.149743000
8	0.328271000	-6.347544000	-1.281907000
7	-0.944301000	-1.802361000	0.217870000
8	8.455868000	-2.248473000	2.197317000
8	1.110424000	6.790018000	3.930098000
8	7.045846000	-0.672849000	2.972571000

8	1.447859000	4.597848000	4.321219000
6	0.981037000	-3.337349000	0.117004000
6	3.316292000	0.849693000	-0.468777000
6	-0.838986000	3.273633000	0.000335000
6	-3.247293000	-0.938545000	0.308487000
28	0.065562000	-0.034831000	0.036353000
6	2.000444000	-2.443589000	-0.064340000
6	2.389918000	1.884308000	-0.407802000
6	-1.895527000	2.328708000	0.076287000
6	-2.317483000	-1.976046000	0.309949000
6	3.440977000	-2.875216000	-0.394448000
6	2.755035000	3.342409000	-0.728165000
6	-3.299720000	2.660821000	0.150501000
6	-2.640262000	-3.381039000	0.397979000
6	4.207576000	-1.522696000	-0.242772000
6	1.577811000	4.080397000	-0.029305000
6	-3.988305000	1.468097000	0.242400000
6	-1.434768000	-4.050237000	0.376783000
6	3.067235000	-0.510570000	-0.286184000
6	0.502045000	3.004317000	-0.065414000
6	-2.987570000	0.428476000	0.226554000
6	-0.401712000	-3.047790000	0.247070000
6	3.432818000	-3.305519000	-1.883615000
6	-3.886222000	4.048132000	0.094560000
6	-4.019045000	-3.958518000	0.536592000
6	4.778266000	-3.681349000	-2.468270000
6	2.931827000	4.868428000	-2.788032000
6	-3.964746000	4.524518000	-1.343941000
6	-4.542591000	-3.865640000	1.978660000
8	5.871662000	-3.525811000	-1.958347000
8	4.041954000	5.292416000	-3.045594000
8	-4.809581000	4.189734000	-2.152657000
6	2.618776000	3.480001000	-2.287024000
6	-5.474925000	1.267654000	0.281744000
8	-2.946777000	5.355066000	-1.665067000
6	3.917585000	-4.048221000	0.476546000
6	4.164119000	3.755759000	-0.286441000
6	-6.068042000	1.127766000	-1.129649000
6	-1.198299000	-5.538591000	0.437329000
6	7.297133000	-1.544592000	2.164469000
6	1.104060000	5.642440000	3.529109000
7	-1.720201000	0.981606000	0.125594000
8	-8.039368000	0.671440000	-2.365540000
8	-1.871108000	-6.191916000	-1.746069000
1	5.200413000	-0.235112000	1.166643000
1	4.475292000	-1.632041000	1.921417000

1	2.765264000	5.179817000	1.414111000
1	2.207832000	3.612312000	2.000376000
1	6.353974000	-3.075526000	1.044864000
1	6.946217000	-1.753580000	0.084486000
1	0.406125000	6.072116000	1.580723000
1	-0.109279000	4.497072000	2.212564000
1	1.240387000	-4.389487000	0.088776000
1	4.348176000	1.121866000	-0.667111000
1	-1.122860000	4.320983000	-0.012119000
1	4.878627000	-1.364838000	-1.093398000
1	1.273244000	4.974708000	-0.582349000
1	3.032825000	-2.492071000	-2.504681000
1	2.750536000	-4.151310000	-2.028208000
1	-4.907653000	4.043523000	0.486949000
1	-3.295000000	4.757801000	0.681551000
1	-4.029693000	-5.006187000	0.217656000
1	-4.722796000	-3.440354000	-0.125491000
1	-3.881914000	-4.404523000	2.669391000
1	3.327800000	2.793159000	-2.756459000
1	1.603275000	3.200943000	-2.581666000
1	-5.964640000	2.111381000	0.782146000
1	-5.733435000	0.380340000	0.870743000
1	3.901849000	-3.791578000	1.539599000
1	4.927437000	-4.358338000	0.201018000
1	3.261811000	-4.914084000	0.337795000
1	4.312600000	4.834547000	-0.395023000
1	4.349770000	3.480721000	0.755228000
1	4.923360000	3.265640000	-0.904155000
1	-5.846571000	2.027475000	-1.717976000
1	-5.606158000	0.290691000	-1.666905000
1	-2.104716000	-6.054637000	0.768565000
1	-0.390133000	-5.786746000	1.131734000
1	8.974578000	-1.902655000	2.948866000
1	1.681431000	4.967213000	5.194250000
1	-9.006748000	0.568173000	-2.281685000
1	-3.030983000	5.559267000	-2.616729000
1	-1.534370000	-6.485354000	-2.614376000
1	5.530833000	-4.424027000	-4.029432000
1	2.120631000	6.526897000	-3.181189000
1	-4.292909000	-1.227421000	0.375549000
1	-4.544174000	-2.824760000	2.324895000
6	-5.940247000	-4.416427000	2.137402000
8	-6.625954000	-4.891721000	1.254223000
8	-6.369767000	-4.322156000	3.419100000
1	-7.274104000	-4.689931000	3.442763000
8	1.822488000	5.641049000	-2.896591000

8	4.633028000	-4.226030000	-3.699491000

Triplet

A-oxidizedstate-triplet-eps4

Oxidized-state-triplet.gjf.log

Temperature 298.150 Kelvin. Pressure 1.00000 Atm.

Zero-point correction= 0.842819 (Hartree/Particle)
Thermal correction to Energy= 0.902670
Thermal correction to Enthalpy= 0.903615
Thermal correction to Gibbs Free Energy= 0.737670
Sum of electronic and zero-point Energies= -3218.420682
Sum of electronic and thermal Energies= -3218.360830
Sum of electronic and thermal Enthalpies= -3218.359886
Sum of electronic and thermal Free Energies= -3218.525831

28	-0.196228000	-0.380135000	-0.622503000
7	0.597556000	1.470456000	-0.700098000
7	1.699048000	-1.187709000	-0.400121000
7	-0.975191000	-2.265954000	-0.534543000
7	-2.043544000	0.396210000	-0.572093000
6	-0.084385000	2.673365000	-0.558102000
6	0.841991000	3.770531000	-0.688170000
6	1.903245000	1.780236000	-0.891947000
6	2.079027000	3.211376000	-0.931623000
6	-3.210077000	-0.307116000	-0.540881000
6	-2.366684000	1.717742000	-0.284266000
6	-3.768894000	1.820273000	-0.004485000
6	-4.293711000	0.544826000	-0.165294000
6	-1.448994000	2.770145000	-0.314864000
6	-2.297098000	-2.590073000	-0.816298000
6	-0.331171000	-3.368282000	-0.117642000
6	-1.295213000	-4.535987000	0.032535000
6	-2.422938000	-4.102960000	-0.956153000
6	-3.322731000	-1.698626000	-0.824731000
6	1.960110000	-2.439008000	0.035765000
6	2.879593000	-0.489049000	-0.574169000
6	3.433697000	-2.600169000	0.415208000
6	4.076716000	-1.404010000	-0.356393000
6	1.023932000	-3.452802000	0.198419000
6	2.966581000	0.836671000	-0.886700000
1	4.396337000	-1.784280000	-1.337009000
6	5.296233000	-0.754362000	0.332341000
1	5.827950000	-1.503082000	0.926466000
1	4.956521000	0.018211000	1.030290000

6	6.323853000	-0.148967000	-0.651302000
1	6.719662000	-0.940559000	-1.293344000
1	5.881061000	0.627236000	-1.276926000
6	7.443778000	0.475235000	0.142969000
8	7.463209000	1.624970000	0.561960000
8	8.425117000	-0.396441000	0.420838000
1	3.956960000	1.241153000	-1.038898000
6	3.515309000	-2.449312000	1.953183000
1	2.883366000	-3.204078000	2.431803000
1	4.535147000	-2.587633000	2.319312000
1	3.159912000	-1.460664000	2.261636000
1	-0.832751000	-5.476783000	-0.285838000
6	-1.733765000	-4.708411000	1.509039000
1	-2.463966000	-5.523613000	1.560450000
1	-0.867959000	-5.039253000	2.092111000
6	-2.321544000	-3.453442000	2.172062000
1	-1.562024000	-2.662123000	2.237137000
1	-3.132981000	-3.025047000	1.582411000
6	-2.817815000	-3.724048000	3.582694000
8	-2.391053000	-4.613776000	4.290832000
8	-3.773530000	-2.893263000	4.044256000
6	-3.810168000	-4.684819000	-0.672378000
1	-4.520738000	-4.395032000	-1.451304000
1	-3.763359000	-5.779129000	-0.662079000
1	-4.209419000	-4.351365000	0.288204000
6	-1.953893000	-4.528112000	-2.395318000
1	-2.105947000	-5.605671000	-2.508025000
1	-0.889924000	-4.307606000	-2.511014000
1	9.079953000	0.058727000	0.986396000
1	-4.076433000	-2.258597000	3.356076000
1	-1.845737000	3.766346000	-0.145366000
6	4.016234000	-3.967167000	-0.036156000
1	3.776161000	-4.139396000	-1.088628000
1	3.582838000	-4.772583000	0.562346000
6	5.515110000	-4.024922000	0.161085000
8	6.075772000	-4.298474000	1.203101000
1	1.373972000	-4.407359000	0.575002000
6	3.377664000	3.930288000	-1.180911000
1	3.771529000	3.699389000	-2.175947000
1	3.215635000	5.012498000	-1.137729000
6	0.542843000	5.225675000	-0.478726000
1	-0.514311000	5.445993000	-0.656950000
1	1.099933000	5.841006000	-1.193977000
6	4.441267000	3.594148000	-0.142459000
8	4.303436000	3.735660000	1.057373000
8	5.549404000	3.097498000	-0.718584000

1	6.189997000	2.762591000	-0.039478000
6	0.921305000	5.657431000	0.948584000
1	0.328384000	5.108729000	1.690738000
1	1.967728000	5.404794000	1.164271000
6	0.723472000	7.136880000	1.181812000
8	0.344014000	7.941967000	0.354805000
8	1.027501000	7.483494000	2.453552000
1	0.883196000	8.446380000	2.530641000
6	-4.500598000	3.080859000	0.352877000
1	-3.890305000	3.707511000	1.012107000
1	-5.409660000	2.850646000	0.918257000
6	-5.667348000	0.051594000	0.165382000
1	-6.358286000	0.876733000	0.373647000
1	-6.127869000	-0.512722000	-0.656240000
6	-4.885004000	3.894015000	-0.894132000
1	-5.515120000	3.300007000	-1.567810000
1	-3.997050000	4.162335000	-1.479061000
6	-5.629932000	5.164401000	-0.551172000
8	-5.946143000	5.521680000	0.565549000
8	-5.918852000	5.882450000	-1.660253000
1	-6.397994000	6.682110000	-1.368716000
6	-5.676193000	-0.857473000	1.389124000
8	-4.713800000	-1.086163000	2.103583000
8	-6.887301000	-1.392998000	1.599877000
1	-6.839131000	-1.952250000	2.399810000
1	-4.323928000	-2.088973000	-0.972287000
8	6.191664000	-3.681945000	-0.961257000
1	7.143890000	-3.700389000	-0.740740000
6	-2.670738000	-3.780642000	-3.494165000
8	-2.287806000	-2.740543000	-3.990430000
8	-3.828410000	-4.379445000	-3.860036000
1	-4.240111000	-3.814405000	-4.542873000

Singlet
A-oxidizedstate-singlet-eps4
Oxidized-state-singlet.gjf.log

Temperature   298.150 Kelvin. Pressure   1.00000 Atm.

Zero-point correction= 0.844882 (Hartree/Particle)
Thermal correction to Energy= 0.904082
Thermal correction to Enthalpy= 0.905026
Thermal correction to Gibbs Free Energy= 0.742070
Sum of electronic and zero-point Energies= -3218.437295
Sum of electronic and thermal Energies= -3218.378095
Sum of electronic and thermal Enthalpies= -3218.377151

Sum of electronic and thermal Free Energies= -3218.540106

28	-0.215413000	-0.357028000	-0.423306000
7	0.617096000	1.382855000	-0.563139000
7	1.547156000	-1.202226000	-0.185335000
7	-1.053891000	-2.110492000	-0.344917000
7	-1.965041000	0.475538000	-0.498079000
6	0.029952000	2.624746000	-0.334971000
6	1.001232000	3.665970000	-0.531366000
6	1.912713000	1.629571000	-0.893714000
6	2.164671000	3.044400000	-0.932204000
6	-3.158771000	-0.148306000	-0.685664000
6	-2.265498000	1.788082000	-0.144164000
6	-3.686579000	1.954500000	-0.043178000
6	-4.243347000	0.741721000	-0.401922000
6	-1.316151000	2.798372000	-0.061099000
6	-2.311507000	-2.423407000	-0.856249000
6	-0.522084000	-3.224442000	0.193298000
6	-1.526291000	-4.362562000	0.197939000
6	-2.437120000	-3.932106000	-0.994766000
6	-3.299496000	-1.514969000	-1.037060000
6	1.758371000	-2.403917000	0.397430000
6	2.765400000	-0.634329000	-0.517614000
6	3.230461000	-2.607242000	0.740980000
6	3.901781000	-1.604983000	-0.249221000
6	0.779590000	-3.349053000	0.655156000
6	2.922037000	0.640803000	-0.962843000
1	4.115115000	-2.156319000	-1.176031000
6	5.212951000	-0.968615000	0.257794000
1	5.736502000	-1.677039000	0.905970000
1	4.984273000	-0.092332000	0.873139000
6	6.198661000	-0.573455000	-0.864783000
1	6.467459000	-1.461933000	-1.442383000
1	5.772819000	0.172476000	-1.537883000
6	7.432701000	0.019337000	-0.231763000
8	7.571938000	1.192467000	0.088080000
8	8.372935000	-0.901761000	0.025608000
1	3.913887000	0.970240000	-1.234629000
6	3.407718000	-2.208853000	2.225435000
1	2.754208000	-2.824146000	2.852032000
1	4.435334000	-2.362609000	2.562737000
1	3.137838000	-1.158785000	2.377411000
1	-1.031632000	-5.316233000	-0.016387000
6	-2.224986000	-4.506539000	1.571670000
1	-2.962379000	-5.313950000	1.500539000
1	-1.480409000	-4.840385000	2.301847000

6	-2.904825000	-3.242454000	2.113863000
1	-2.172038000	-2.434978000	2.249137000
1	-3.643435000	-2.846241000	1.415148000
6	-3.570006000	-3.485795000	3.460168000
8	-3.253897000	-4.381230000	4.217756000
8	-4.548176000	-2.625870000	3.801633000
6	-3.865761000	-4.480129000	-0.965949000
1	-4.412982000	-4.188338000	-1.866562000
1	-3.846029000	-5.574700000	-0.933414000
1	-4.427223000	-4.122559000	-0.099862000
6	-1.715036000	-4.396958000	-2.311359000
1	-1.864349000	-5.474192000	-2.429817000
1	-0.643977000	-4.195687000	-2.230391000
1	9.109535000	-0.458009000	0.490904000
1	-4.742074000	-1.983100000	3.081282000
1	-1.664131000	3.801903000	0.158605000
6	3.696492000	-4.069089000	0.501658000
1	3.373397000	-4.399206000	-0.489009000
1	3.257536000	-4.727871000	1.255373000
6	5.199073000	-4.201469000	0.617677000
8	5.813955000	-4.332373000	1.656719000
1	1.061409000	-4.283579000	1.124407000
6	3.472595000	3.688870000	-1.304267000
1	3.752801000	3.448584000	-2.334705000
1	3.378282000	4.777884000	-1.237639000
6	0.814762000	5.129381000	-0.259993000
1	-0.241108000	5.413929000	-0.301976000
1	1.316466000	5.730225000	-1.026664000
6	4.611123000	3.279227000	-0.376890000
8	4.593481000	3.403026000	0.832936000
8	5.632446000	2.737908000	-1.061994000
1	6.318908000	2.368007000	-0.447954000
6	1.389867000	5.500764000	1.117861000
1	0.854756000	4.973871000	1.917425000
1	2.435391000	5.176120000	1.201102000
6	1.321267000	6.984030000	1.395826000
8	0.906947000	7.831607000	0.630287000
8	1.790279000	7.279401000	2.629632000
1	1.722278000	8.247621000	2.738207000
6	-4.391315000	3.230323000	0.313730000
1	-3.833623000	3.777978000	1.080998000
1	-5.370167000	3.014455000	0.754841000
6	-5.673171000	0.305342000	-0.322779000
1	-6.343577000	1.157728000	-0.157293000
1	-6.027867000	-0.177403000	-1.241060000
6	-4.587159000	4.141563000	-0.909105000

1	-5.165323000	3.629724000	-1.688457000
1	-3.624919000	4.392547000	-1.371495000
6	-5.298596000	5.430502000	-0.564636000
8	-5.718126000	5.737632000	0.532804000
8	-5.423882000	6.230479000	-1.647921000
1	-5.889223000	7.038337000	-1.356672000
6	-5.901265000	-0.662479000	0.830302000
8	-5.146404000	-0.801025000	1.779432000
8	-7.042353000	-1.352390000	0.694803000
1	-7.145769000	-1.929929000	1.476470000
1	-4.273974000	-1.862398000	-1.360874000
8	5.812874000	-4.105589000	-0.585660000
1	6.775790000	-4.153587000	-0.423853000
6	-2.201158000	-3.661243000	-3.537150000
8	-1.721746000	-2.629719000	-3.962163000
8	-3.271806000	-4.258650000	-4.110630000
1	-3.541002000	-3.700991000	-4.866741000

c) Optimized reduced (doublet) and oxidized (triplet and singlet) state of **B** in aqueous solvent.

B-reduced-state-doublet-H2O

Reduced-state-doublet.gjf.log

28	-0.168168000	-0.403363000	-0.649272000
7	0.542775000	1.502517000	-0.731570000
7	1.763047000	-1.130471000	-0.441791000
7	-0.891591000	-2.305367000	-0.542037000
7	-2.082855000	0.332154000	-0.649165000
6	-0.191816000	2.677241000	-0.636189000
6	0.689394000	3.816656000	-0.747761000
6	1.842068000	1.867750000	-0.876771000
6	1.961194000	3.308837000	-0.919920000
6	-3.208782000	-0.413978000	-0.552627000
6	-2.452693000	1.641622000	-0.375110000
6	-3.857550000	1.697704000	-0.039982000
6	-4.325589000	0.401757000	-0.145909000
6	-1.570856000	2.723894000	-0.423986000
6	-2.208003000	-2.680210000	-0.759806000
6	-0.198662000	-3.380791000	-0.118838000
6	-1.126821000	-4.563468000	0.131409000
6	-2.304878000	-4.204471000	-0.826494000
6	-3.268735000	-1.821462000	-0.772971000
6	2.077401000	-2.373167000	-0.027616000
6	2.911119000	-0.378221000	-0.575177000
6	3.558488000	-2.493183000	0.352276000
6	4.152283000	-1.243598000	-0.373121000

6	1.170192000	-3.419945000	0.143602000
6	2.941692000	0.963983000	-0.847817000
1	4.497818000	-1.575026000	-1.363680000
6	5.340328000	-0.567733000	0.344705000
1	5.903431000	-1.315460000	0.909456000
1	4.963217000	0.164229000	1.067663000
6	6.342803000	0.121715000	-0.611086000
1	6.796913000	-0.631358000	-1.261445000
1	5.861769000	0.880622000	-1.229651000
6	7.412372000	0.796107000	0.208926000
8	7.377905000	1.953353000	0.612218000
8	8.414889000	-0.032289000	0.531380000
1	3.918940000	1.410946000	-0.970324000
6	3.638184000	-2.391450000	1.893501000
1	3.032815000	-3.184779000	2.344650000
1	4.662817000	-2.506917000	2.251694000
1	3.243717000	-1.427771000	2.234310000
1	-0.660199000	-5.512859000	-0.155655000
6	-1.499928000	-4.668283000	1.631959000
1	-2.204205000	-5.498290000	1.762274000
1	-0.599217000	-4.938723000	2.193630000
6	-2.096896000	-3.394190000	2.250705000
1	-1.369425000	-2.571772000	2.204905000
1	-2.961707000	-3.042740000	1.686988000
6	-2.486755000	-3.576117000	3.705725000
8	-1.959532000	-4.374624000	4.457691000
8	-3.463827000	-2.771671000	4.167442000
6	-3.665433000	-4.801283000	-0.453551000
1	-4.422173000	-4.542268000	-1.200418000
1	-3.602274000	-5.894804000	-0.410561000
1	-4.028984000	-4.444484000	0.512851000
6	-1.890143000	-4.703315000	-2.256422000
1	-1.952382000	-5.797885000	-2.270165000
1	-0.849221000	-4.424131000	-2.441721000
6	-2.703491000	-4.092368000	-3.388443000
8	-2.397686000	-3.016421000	-3.905736000
7	-3.798422000	-4.795188000	-3.783508000
1	-4.382398000	-4.424075000	-4.520987000
1	-4.046410000	-5.687116000	-3.381598000
1	9.037036000	0.453082000	1.108839000
1	-3.855153000	-2.207609000	3.458766000
1	-2.004066000	3.709301000	-0.272250000
6	4.195305000	-3.817205000	-0.148690000
1	3.897760000	-3.986007000	-1.189187000
1	3.818004000	-4.655837000	0.444822000
6	5.713926000	-3.821718000	-0.010148000

8	6.277201000	-3.938292000	1.081002000
7	6.412108000	-3.635753000	-1.161431000
1	7.423063000	-3.636968000	-1.124426000
1	5.971182000	-3.615471000	-2.069152000
1	1.560863000	-4.361481000	0.517278000
6	3.235738000	4.085318000	-1.121347000
1	3.646201000	3.928284000	-2.124901000
1	3.037149000	5.158420000	-1.019292000
6	0.306718000	5.259945000	-0.587484000
1	-0.736827000	5.427637000	-0.873600000
1	0.904327000	5.896430000	-1.250350000
6	4.311377000	3.734567000	-0.102001000
8	4.156418000	3.754717000	1.105885000
8	5.461453000	3.378216000	-0.698358000
1	6.109220000	3.022399000	-0.034167000
6	0.512681000	5.726094000	0.863357000
1	-0.110684000	5.138379000	1.549247000
1	1.548026000	5.550589000	1.181618000
6	0.186596000	7.186592000	1.070255000
8	-0.231244000	7.949730000	0.221046000
8	0.414301000	7.570016000	2.347031000
1	0.181666000	8.516575000	2.412581000
6	-4.621684000	2.934966000	0.331823000
1	-4.020864000	3.578658000	0.984672000
1	-5.518329000	2.676389000	0.906442000
6	-5.650691000	-0.169391000	0.266182000
1	-6.318041000	0.615075000	0.645095000
1	-6.185353000	-0.661346000	-0.554262000
6	-5.046414000	3.744303000	-0.904331000
1	-5.681558000	3.140686000	-1.564438000
1	-4.174054000	4.024772000	-1.507422000
6	-5.798187000	5.007307000	-0.552505000
8	-6.061400000	5.389197000	0.571225000
8	-6.160527000	5.693400000	-1.660060000
1	-6.638979000	6.492174000	-1.364295000
6	-5.477768000	-1.179705000	1.392590000
8	-4.666022000	-1.073690000	2.301298000
8	-6.319388000	-2.218477000	1.288142000
1	-6.170993000	-2.814538000	2.048555000
1	-4.257978000	-2.257986000	-0.873395000

Triplet

B-oxidized-state-triplet-H2O

Oxidized-state-triplet.gjf.log

28	-0.199927000	-0.373000000	-0.629003000
7	0.609939000	1.480907000	-0.708787000

7	1.688912000	-1.193211000	-0.393132000
7	-1.002547000	-2.252617000	-0.518131000
7	-2.046696000	0.429069000	-0.593079000
6	-0.064544000	2.688866000	-0.579457000
6	0.871837000	3.776967000	-0.710603000
6	1.920119000	1.775979000	-0.893907000
6	2.107793000	3.205667000	-0.938436000
6	-3.215387000	-0.268842000	-0.543889000
6	-2.358022000	1.753575000	-0.304645000
6	-3.758444000	1.863683000	-0.007422000
6	-4.290421000	0.590582000	-0.156188000
6	-1.431660000	2.797798000	-0.341871000
6	-2.326793000	-2.567299000	-0.790200000
6	-0.367549000	-3.352283000	-0.079437000
6	-1.346686000	-4.502570000	0.104748000
6	-2.472935000	-4.081283000	-0.892237000
6	-3.342809000	-1.662910000	-0.809646000
6	1.936783000	-2.442737000	0.055129000
6	2.875753000	-0.505888000	-0.567826000
6	3.408851000	-2.619219000	0.432819000
6	4.063482000	-1.433033000	-0.346250000
6	0.988236000	-3.444281000	0.232462000
6	2.974100000	0.819571000	-0.882285000
1	4.374143000	-1.818418000	-1.328394000
6	5.293340000	-0.795732000	0.335817000
1	5.816443000	-1.553241000	0.924647000
1	4.963663000	-0.017663000	1.032835000
6	6.324670000	-0.202375000	-0.652372000
1	6.723562000	-1.002476000	-1.282124000
1	5.886007000	0.565903000	-1.290877000
6	7.441955000	0.430268000	0.138556000
8	7.462679000	1.589988000	0.535380000
8	8.415740000	-0.437125000	0.441586000
1	3.969151000	1.212114000	-1.036051000
6	3.491035000	-2.450862000	1.969490000
1	2.847755000	-3.192310000	2.454493000
1	4.509746000	-2.604161000	2.329034000
1	3.148658000	-1.454057000	2.267762000
1	-0.898266000	-5.457707000	-0.188826000
6	-1.778061000	-4.629239000	1.588162000
1	-2.520835000	-5.431108000	1.664397000
1	-0.912114000	-4.956802000	2.172840000
6	-2.342244000	-3.348697000	2.222687000
1	-1.575913000	-2.561460000	2.243574000
1	-3.165174000	-2.936229000	1.637906000
6	-2.804175000	-3.564074000	3.653269000

8	-2.348170000	-4.416327000	4.391927000
8	-3.758264000	-2.728450000	4.104173000
6	-3.869089000	-4.629825000	-0.584511000
1	-4.579498000	-4.340321000	-1.364977000
1	-3.844592000	-5.724421000	-0.548236000
1	-4.261881000	-4.265707000	0.367783000
6	-2.021088000	-4.560690000	-2.316903000
1	-2.144729000	-5.648454000	-2.365380000
1	-0.958929000	-4.338002000	-2.451792000
6	-2.749803000	-3.860220000	-3.455670000
8	-2.384794000	-2.761081000	-3.876606000
7	-3.838481000	-4.501594000	-3.953977000
1	-4.353314000	-4.078194000	-4.714708000
1	-4.111670000	-5.424675000	-3.650533000
1	9.071764000	0.023628000	1.001648000
1	-4.089841000	-2.122109000	3.400167000
1	-1.820201000	3.798291000	-0.177987000
6	3.975051000	-3.994685000	-0.008192000
1	3.657630000	-4.202286000	-1.035317000
1	3.568810000	-4.783679000	0.632065000
6	5.494542000	-4.059160000	0.118110000
8	6.060560000	-4.125655000	1.211437000
7	6.186922000	-3.979742000	-1.048049000
1	7.196490000	-4.041145000	-1.018051000
1	5.738463000	-4.026471000	-1.951381000
1	1.331364000	-4.396738000	0.620645000
6	3.413791000	3.913635000	-1.181595000
1	3.796728000	3.703702000	-2.185584000
1	3.267980000	4.996868000	-1.110727000
6	0.580592000	5.236932000	-0.521073000
1	-0.472731000	5.461356000	-0.715580000
1	1.152407000	5.839463000	-1.235720000
6	4.482287000	3.536894000	-0.163125000
8	4.345345000	3.621408000	1.043834000
8	5.591711000	3.075476000	-0.760715000
1	6.231835000	2.704933000	-0.096370000
6	0.941014000	5.683107000	0.906155000
1	0.335905000	5.143086000	1.645027000
1	1.983988000	5.432154000	1.138444000
6	0.741469000	7.164619000	1.125623000
8	0.339957000	7.957462000	0.296216000
8	1.069041000	7.527040000	2.385742000
1	0.919247000	8.489552000	2.460816000
6	-4.479570000	3.127171000	0.359679000
1	-3.850436000	3.758748000	0.995919000
1	-5.373071000	2.899245000	0.950605000

6	-5.659011000	0.087366000	0.192859000
1	-6.307936000	0.904528000	0.531230000
1	-6.174729000	-0.379049000	-0.654139000
6	-4.898111000	3.930001000	-0.882878000
1	-5.556950000	3.335980000	-1.527885000
1	-4.026479000	4.182744000	-1.498939000
6	-5.613900000	5.214456000	-0.531161000
8	-5.849495000	5.610175000	0.593585000
8	-5.975144000	5.897707000	-1.639375000
1	-6.429952000	6.711158000	-1.346203000
6	-5.600267000	-0.926513000	1.328835000
8	-4.745544000	-0.937611000	2.202991000
8	-6.598349000	-1.816237000	1.270387000
1	-6.521369000	-2.421233000	2.034573000
1	-4.348510000	-2.043496000	-0.952684000

Singlet

B-oxidized-state-singlet-H2O

Oxidized-state-singlet.gjf.log

28	-0.213620000	-0.350603000	-0.416792000
7	0.619085000	1.394128000	-0.567815000
7	1.550813000	-1.194145000	-0.167220000
7	-1.056087000	-2.103691000	-0.319139000
7	-1.966826000	0.484548000	-0.509935000
6	0.027629000	2.636330000	-0.353936000
6	0.998488000	3.676393000	-0.554420000
6	1.917058000	1.637255000	-0.892418000
6	2.166782000	3.052232000	-0.940985000
6	-3.158010000	-0.145724000	-0.687620000
6	-2.269207000	1.798677000	-0.161256000
6	-3.691911000	1.960000000	-0.054712000
6	-4.245051000	0.743470000	-0.404834000
6	-1.321500000	2.810486000	-0.086422000
6	-2.310530000	-2.423519000	-0.829900000
6	-0.525345000	-3.208741000	0.238798000
6	-1.535670000	-4.341399000	0.266435000
6	-2.439623000	-3.934033000	-0.940326000
6	-3.298411000	-1.516351000	-1.023246000
6	1.759499000	-2.391003000	0.424919000
6	2.769729000	-0.628400000	-0.501873000
6	3.232429000	-2.604319000	0.759120000
6	3.904393000	-1.602227000	-0.232489000
6	0.776813000	-3.328747000	0.699308000
6	2.926458000	0.646028000	-0.951064000
1	4.111587000	-2.150544000	-1.163024000
6	5.220443000	-0.973607000	0.271626000

1	5.742829000	-1.694803000	0.905075000
1	4.995687000	-0.103367000	0.897389000
6	6.203010000	-0.566603000	-0.850374000
1	6.486796000	-1.452516000	-1.424867000
1	5.770632000	0.171654000	-1.527834000
6	7.426784000	0.043806000	-0.214181000
8	7.546058000	1.221573000	0.104438000
8	8.379282000	-0.859005000	0.047804000
1	3.918896000	0.972757000	-1.223886000
6	3.417803000	-2.204002000	2.242237000
1	2.756778000	-2.810149000	2.870370000
1	4.444117000	-2.377699000	2.569950000
1	3.162036000	-1.149880000	2.393418000
1	-1.045455000	-5.302148000	0.077055000
6	-2.237113000	-4.449645000	1.642113000
1	-2.980060000	-5.253196000	1.586657000
1	-1.493899000	-4.771256000	2.379146000
6	-2.908638000	-3.169734000	2.157158000
1	-2.173328000	-2.358687000	2.252318000
1	-3.662627000	-2.797566000	1.461815000
6	-3.542261000	-3.365290000	3.525089000
8	-3.188027000	-4.214523000	4.320816000
8	-4.532874000	-2.515276000	3.849633000
6	-3.873235000	-4.469537000	-0.904111000
1	-4.416353000	-4.183389000	-1.810169000
1	-3.865216000	-5.563757000	-0.854139000
1	-4.438743000	-4.092370000	-0.048808000
6	-1.719747000	-4.437022000	-2.240545000
1	-1.828695000	-5.526055000	-2.291262000
1	-0.651740000	-4.210658000	-2.173247000
6	-2.220289000	-3.759775000	-3.509051000
8	-1.805664000	-2.652004000	-3.855362000
7	-3.166490000	-4.432564000	-4.213105000
1	-3.534154000	-4.021676000	-5.061062000
1	-3.483013000	-5.355755000	-3.955256000
1	9.110673000	-0.405781000	0.512745000
1	-4.763409000	-1.910773000	3.104854000
1	-1.670984000	3.815432000	0.124427000
6	3.686822000	-4.067362000	0.513335000
1	3.289629000	-4.409820000	-0.447606000
1	3.278525000	-4.716875000	1.293477000
6	5.204541000	-4.217515000	0.562999000
8	5.834924000	-4.161938000	1.621093000
7	5.822488000	-4.357237000	-0.638433000
1	6.826640000	-4.480928000	-0.655921000
1	5.314307000	-4.505283000	-1.498224000

1	1.059548000	-4.258356000	1.177660000
6	3.475071000	3.697833000	-1.310369000
1	3.754844000	3.463128000	-2.342189000
1	3.382652000	4.786656000	-1.238508000
6	0.806901000	5.142980000	-0.302913000
1	-0.248996000	5.424583000	-0.359604000
1	1.316856000	5.734518000	-1.071434000
6	4.613101000	3.280831000	-0.386813000
8	4.591039000	3.388743000	0.826070000
8	5.637315000	2.753107000	-1.073792000
1	6.318661000	2.364263000	-0.462301000
6	1.365327000	5.532115000	1.076606000
1	0.821931000	5.012130000	1.875265000
1	2.411095000	5.213120000	1.174869000
6	1.287723000	7.017491000	1.341037000
8	0.852911000	7.853330000	0.572914000
8	1.771812000	7.328387000	2.563827000
1	1.695513000	8.296488000	2.670564000
6	-4.400701000	3.234266000	0.299501000
1	-3.836628000	3.792141000	1.054534000
1	-5.372862000	3.014205000	0.753237000
6	-5.672791000	0.293209000	-0.323611000
1	-6.338814000	1.133699000	-0.093658000
1	-6.039012000	-0.142002000	-1.259529000
6	-4.617108000	4.132653000	-0.929124000
1	-5.207715000	3.612348000	-1.693101000
1	-3.661893000	4.379926000	-1.408014000
6	-5.322059000	5.425697000	-0.587677000
8	-5.707820000	5.752919000	0.517408000
8	-5.482731000	6.203874000	-1.680399000
1	-5.942281000	7.017412000	-1.394998000
6	-5.854656000	-0.731339000	0.787433000
8	-5.205542000	-0.740705000	1.823390000
8	-6.806807000	-1.629270000	0.509678000
1	-6.890113000	-2.241752000	1.267270000
1	-4.272493000	-1.866678000	-1.344548000

d) Optimized reduced (doublet) and oxidized (triplet and singlet) state of **B** in epsilon=4.

B-reduced-state-doublet-eps4
Reduced-state-doublet.gjf.log

Temperature 298.150 Kelvin. Pressure 1.00000 Atm.

Zero-point correction= 0.865192 (Hartree/Particle)

Thermal correction to Energy= 0.926020
Thermal correction to Enthalpy= 0.926964
Thermal correction to Gibbs Free Energy= 0.760345
Sum of electronic and zero-point Energies= -3178.778640
Sum of electronic and thermal Energies= -3178.717812
Sum of electronic and thermal Enthalpies= -3178.716868
Sum of electronic and thermal Free Energies= -3178.883487

6	-4.440021000	0.976920000	1.888113000
6	-0.401488000	-4.919696000	1.077413000
6	7.412895000	0.050540000	0.485257000
6	-0.319603000	5.962691000	0.598321000
7	-2.005157000	0.638388000	-0.267022000
6	-5.841557000	1.421734000	2.312055000
6	1.087758000	-5.260769000	1.133823000
7	-0.789416000	-1.999530000	-0.452183000
8	8.228770000	0.140112000	-0.411614000
8	-1.472880000	6.058925000	0.967321000
7	0.546698000	1.874023000	-0.804704000
8	-7.208810000	2.024734000	4.151629000
8	2.807015000	-6.298200000	2.450328000
8	-5.164094000	1.216823000	4.642326000
8	0.748725000	-6.158326000	3.337152000
6	-1.628908000	3.018532000	-0.667048000
6	-3.081246000	-1.503436000	0.205330000
6	1.274445000	-3.136194000	-1.099673000
6	2.952637000	1.424959000	-1.048453000
28	-0.124266000	-0.056295000	-0.657217000
6	-2.441102000	1.938412000	-0.464191000
6	-2.051853000	-2.368094000	-0.146068000
6	2.147671000	-2.020698000	-1.184988000
6	1.863306000	2.289105000	-0.923948000
6	-3.974613000	1.969394000	-0.512856000
6	-2.277801000	-3.864728000	-0.391640000
6	3.587196000	-2.117772000	-1.285198000
6	1.932384000	3.730326000	-0.939507000
6	-4.296177000	0.727357000	0.369416000
6	-0.812944000	-4.387360000	-0.323104000
6	4.073517000	-0.829457000	-1.231077000
6	0.627962000	4.175092000	-0.851237000
6	-3.056486000	-0.114703000	0.104139000
6	-0.035711000	-3.126594000	-0.694452000
6	2.920190000	0.033813000	-1.111437000
6	-0.209576000	3.001981000	-0.761738000
6	-4.352478000	1.639973000	-2.001941000
6	4.370144000	-3.402326000	-1.263140000

6	3.189108000	4.549817000	-0.962113000
7	-5.195633000	-3.815795000	-1.978491000
6	-5.811196000	1.244655000	-2.198528000
6	-3.997225000	-3.243411000	-2.262435000
6	4.302288000	-4.061907000	0.112169000
6	3.791107000	4.691979000	0.445447000
8	-6.744607000	2.034375000	-2.026714000
8	-3.918792000	-2.132466000	-2.797315000
8	4.483064000	-3.480812000	1.167139000
6	-2.760423000	-4.045287000	-1.878648000
6	5.508243000	-0.400985000	-1.139349000
7	-6.013787000	-0.047292000	-2.568863000
8	3.994273000	-5.371564000	0.045449000
6	-4.601768000	3.299351000	-0.090613000
6	-3.256700000	-4.536634000	0.577311000
6	5.954045000	-0.300138000	0.329281000
6	0.138922000	5.599295000	-0.800434000
6	-5.993405000	1.529150000	3.811808000
6	1.634466000	-5.954359000	2.351627000
7	1.758874000	-0.725222000	-1.071866000
8	7.741728000	0.255569000	1.784372000
8	0.728067000	6.145115000	1.440422000
1	-4.207960000	0.043365000	2.412728000
1	-3.691452000	1.704843000	2.222342000
1	-0.994620000	-5.809896000	1.312203000
1	-0.631102000	-4.168688000	1.842055000
1	-6.126596000	2.384159000	1.877763000
1	-6.596610000	0.706141000	1.957787000
1	1.382220000	-5.882924000	0.280718000
1	1.681191000	-4.346869000	1.038366000
1	-2.104072000	3.990228000	-0.745786000
1	-4.041073000	-1.943877000	0.454938000
1	1.724888000	-4.109059000	-1.268410000
1	-5.195669000	0.205440000	0.024029000
1	-0.643343000	-5.192583000	-1.049201000
1	-3.697105000	0.843449000	-2.366847000
1	-4.162047000	2.531795000	-2.608364000
1	4.022708000	-4.117658000	-2.015531000
1	5.428643000	-3.203782000	-1.470970000
1	2.993618000	5.551275000	-1.361984000
1	3.937098000	4.102279000	-1.627065000
1	3.063036000	5.159698000	1.119877000
1	-1.961037000	-3.709551000	-2.544667000
1	-2.928148000	-5.114813000	-2.058550000
1	5.667269000	0.564938000	-1.632806000
1	6.160892000	-1.113391000	-1.656584000

1	-4.304711000	3.581293000	0.924173000
1	-5.690809000	3.250301000	-0.159217000
1	-4.275798000	4.101573000	-0.761889000
1	-3.305302000	-5.616625000	0.391446000
1	-2.961648000	-4.381216000	1.618176000
1	-4.266589000	-4.132989000	0.464678000
1	5.356006000	0.447111000	0.864990000
1	5.766909000	-1.252819000	0.842367000
1	-0.712532000	5.759083000	-1.468918000
1	0.940025000	6.285624000	-1.094086000
1	-7.237599000	2.057628000	5.127095000
1	1.224101000	-6.588030000	4.075331000
1	8.695842000	0.462352000	1.804612000
1	3.820834000	-5.704507000	0.962995000
1	0.355767000	6.315046000	2.326839000
1	-6.969954000	-0.351689000	-2.696897000
1	-5.264380000	-0.731985000	-2.681089000
1	-6.047915000	-3.313604000	-2.186686000
1	3.932832000	1.889459000	-1.120364000
1	4.011710000	3.707482000	0.875695000
6	5.054018000	5.517837000	0.469321000
8	5.542288000	6.108165000	-0.474194000
8	5.610402000	5.543561000	1.705439000
1	6.411428000	6.098577000	1.642915000
1	-5.271898000	-4.730607000	-1.560227000

Triplet

B-oxidized-state-triplet-eps4

Oxidized-state-triplet.gjf.log

Temperature 298.150 Kelvin. Pressure 1.00000 Atm.

Zero-point correction= 0.866822 (Hartree/Particle)
Thermal correction to Energy= 0.927654
Thermal correction to Enthalpy= 0.928598
Thermal correction to Gibbs Free Energy= 0.761324
Sum of electronic and zero-point Energies= -3178.666417
Sum of electronic and thermal Energies= -3178.605584
Sum of electronic and thermal Enthalpies= -3178.604640
Sum of electronic and thermal Free Energies= -3178.771914

28	-0.196218000	-0.376898000	-0.608540000
7	0.608382000	1.469158000	-0.693073000
7	1.692870000	-1.193234000	-0.379010000
7	-0.987266000	-2.256401000	-0.512567000
7	-2.038573000	0.410310000	-0.561198000

6	-0.067287000	2.676498000	-0.559467000
6	0.864642000	3.767512000	-0.696321000
6	1.916251000	1.770416000	-0.885804000
6	2.099185000	3.200143000	-0.935178000
6	-3.208768000	-0.286715000	-0.530091000
6	-2.355307000	1.735509000	-0.282690000
6	-3.758224000	1.847255000	-0.008450000
6	-4.289108000	0.573984000	-0.163312000
6	-1.432095000	2.782304000	-0.318327000
6	-2.308802000	-2.575146000	-0.800225000
6	-0.350854000	-3.359926000	-0.087258000
6	-1.322726000	-4.520667000	0.066010000
6	-2.441075000	-4.087778000	-0.934156000
6	-3.329556000	-1.678074000	-0.808912000
6	1.946286000	-2.443476000	0.064791000
6	2.878793000	-0.503355000	-0.555165000
6	3.418277000	-2.616065000	0.443077000
6	4.069247000	-1.426431000	-0.332796000
6	1.002905000	-3.450412000	0.232209000
6	2.974281000	0.820379000	-0.873901000
1	4.383696000	-1.809816000	-1.315036000
6	5.295953000	-0.785931000	0.352451000
1	5.820753000	-1.542826000	0.940680000
1	4.962204000	-0.009997000	1.049678000
6	6.326593000	-0.186524000	-0.632584000
1	6.725383000	-0.982820000	-1.267373000
1	5.887320000	0.587039000	-1.264286000
6	7.444395000	0.441523000	0.161739000
8	7.457467000	1.590616000	0.584097000
8	8.432600000	-0.422919000	0.435419000
1	3.967423000	1.218211000	-1.025915000
6	3.500740000	-2.453470000	1.980212000
1	2.856752000	-3.196138000	2.462414000
1	4.518975000	-2.609192000	2.340164000
1	3.158011000	-1.457808000	2.281630000
1	-0.863017000	-5.465777000	-0.243887000
6	-1.768768000	-4.683065000	1.541185000
1	-2.503997000	-5.493877000	1.593427000
1	-0.907795000	-5.016262000	2.130165000
6	-2.352668000	-3.422284000	2.196578000
1	-1.588365000	-2.635799000	2.264169000
1	-3.157009000	-2.989959000	1.599928000
6	-2.860511000	-3.686366000	3.604284000
8	-2.447899000	-4.580298000	4.315693000
8	-3.810903000	-2.845976000	4.059407000
6	-3.834687000	-4.655602000	-0.652459000

1	-4.541497000	-4.350369000	-1.430309000
1	-3.801918000	-5.750907000	-0.644716000
1	-4.235018000	-4.321684000	0.307568000
6	-1.971398000	-4.532408000	-2.364190000
1	-2.083883000	-5.620564000	-2.437450000
1	-0.910515000	-4.295916000	-2.484032000
6	-2.694898000	-3.813453000	-3.495280000
8	-2.336632000	-2.706175000	-3.891108000
7	-3.782220000	-4.451043000	-4.011980000
1	-4.272731000	-4.023316000	-4.786116000
1	-4.028779000	-5.395352000	-3.755696000
1	9.083719000	0.035903000	1.002247000
1	-4.102815000	-2.208031000	3.369356000
1	-1.823477000	3.781815000	-0.155762000
6	3.989366000	-3.988063000	-0.002655000
1	3.670195000	-4.193688000	-1.030112000
1	3.587775000	-4.780601000	0.636205000
6	5.509323000	-4.046796000	0.121770000
8	6.078303000	-4.107806000	1.210994000
7	6.200906000	-3.964096000	-1.048773000
1	7.209557000	-4.036680000	-1.015558000
1	5.751512000	-4.034816000	-1.949764000
1	1.348239000	-4.404429000	0.614777000
6	3.401018000	3.911979000	-1.188551000
1	3.795705000	3.671179000	-2.180937000
1	3.243019000	4.995082000	-1.154329000
6	0.573292000	5.225714000	-0.497118000
1	-0.482695000	5.450417000	-0.676926000
1	1.133640000	5.833200000	-1.216587000
6	4.461037000	3.579632000	-0.145475000
8	4.324236000	3.739051000	1.052317000
8	5.564035000	3.063889000	-0.714226000
1	6.199218000	2.727091000	-0.030510000
6	0.954049000	5.665582000	0.927099000
1	0.357978000	5.125418000	1.672993000
1	1.998987000	5.408370000	1.144673000
6	0.764749000	7.147642000	1.150117000
8	0.390510000	7.949532000	0.317576000
8	1.069884000	7.501305000	2.419756000
1	0.931074000	8.465516000	2.489880000
6	-4.484536000	3.114240000	0.336974000
1	-3.874614000	3.741290000	0.996244000
1	-5.398661000	2.892902000	0.897771000
6	-5.666474000	0.088418000	0.163233000
1	-6.350535000	0.917915000	0.378021000
1	-6.131441000	-0.466508000	-0.662029000

6	-4.856013000	3.921952000	-0.917474000
1	-5.484413000	3.326903000	-1.591883000
1	-3.962629000	4.181938000	-1.497821000
6	-5.597052000	5.198074000	-0.588008000
8	-5.923564000	5.562213000	0.523559000
8	-5.869794000	5.913101000	-1.703251000
1	-6.347523000	6.716601000	-1.420131000
6	-5.681190000	-0.826381000	1.382332000
8	-4.731329000	-1.030611000	2.120633000
8	-6.882070000	-1.396070000	1.561658000
1	-6.838864000	-1.953263000	2.363293000
1	-4.332576000	-2.061142000	-0.962266000

Singlet

B-oxidized-state-singlet-eps4

Oxidized-state-singlet.gjf.log

Temperature 298.150 Kelvin. Pressure 1.00000 Atm.

Zero-point correction= 0.868728 (Hartree/Particle)
Thermal correction to Energy= 0.929024
Thermal correction to Enthalpy= 0.929968
Thermal correction to Gibbs Free Energy= 0.765686
Sum of electronic and zero-point Energies= -3178.683341
Sum of electronic and thermal Energies= -3178.623046
Sum of electronic and thermal Enthalpies= -3178.622102
Sum of electronic and thermal Free Energies= -3178.786383

28	-0.218224000	-0.342829000	-0.411780000
7	0.635928000	1.385790000	-0.561671000
7	1.532740000	-1.208624000	-0.165396000
7	-1.080305000	-2.084288000	-0.319989000
7	-1.956412000	0.511157000	-0.495248000
6	0.063105000	2.636346000	-0.344452000
6	1.046591000	3.664000000	-0.548321000
6	1.935254000	1.613972000	-0.891829000
6	2.203489000	3.025233000	-0.941977000
6	-3.156303000	-0.100651000	-0.679513000
6	-2.242450000	1.829224000	-0.148602000
6	-3.662065000	2.011451000	-0.047177000
6	-4.231003000	0.802965000	-0.399690000
6	-1.281464000	2.828450000	-0.072629000
6	-2.338770000	-2.387221000	-0.833450000
6	-0.564617000	-3.198584000	0.233632000
6	-1.585983000	-4.321050000	0.252121000
6	-2.485687000	-3.895514000	-0.951613000

6	-3.315055000	-1.467489000	-1.021244000
6	1.728340000	-2.408157000	0.426447000
6	2.759161000	-0.659103000	-0.500813000
6	3.197840000	-2.635966000	0.765147000
6	3.881719000	-1.645234000	-0.228878000
6	0.735219000	-3.336015000	0.695976000
6	2.932383000	0.611730000	-0.952346000
1	4.083036000	-2.200176000	-1.157330000
6	5.205173000	-1.029529000	0.272651000
1	5.717273000	-1.751111000	0.914052000
1	4.990791000	-0.149871000	0.888510000
6	6.192890000	-0.645361000	-0.852754000
1	6.456567000	-1.538187000	-1.426305000
1	5.773268000	0.101847000	-1.528672000
6	7.431359000	-0.058857000	-0.222312000
8	7.571475000	1.111410000	0.109181000
8	8.376070000	-0.979352000	0.017495000
1	3.928914000	0.927214000	-1.223571000
6	3.384833000	-2.232237000	2.247304000
1	2.717059000	-2.830195000	2.876205000
1	4.408678000	-2.413834000	2.578558000
1	3.137242000	-1.175660000	2.394481000
1	-1.103767000	-5.284511000	0.053202000
6	-2.289811000	-4.438401000	1.625412000
1	-3.039713000	-5.234945000	1.561118000
1	-1.552914000	-4.776398000	2.361463000
6	-2.951970000	-3.159369000	2.155096000
1	-2.205815000	-2.364748000	2.293102000
1	-3.676872000	-2.753713000	1.447353000
6	-3.632796000	-3.388315000	3.496472000
8	-3.333233000	-4.284540000	4.259998000
8	-4.605817000	-2.518443000	3.826013000
6	-3.925279000	-4.414938000	-0.915306000
1	-4.466160000	-4.121197000	-1.820305000
1	-3.929852000	-5.509478000	-0.865932000
1	-4.484474000	-4.033459000	-0.057853000
6	-1.774908000	-4.401014000	-2.255196000
1	-1.884517000	-5.490682000	-2.303332000
1	-0.706662000	-4.174141000	-2.194251000
6	-2.282213000	-3.725583000	-3.522474000
8	-1.879181000	-2.617257000	-3.869295000
7	-3.230094000	-4.405869000	-4.224537000
1	-3.585709000	-4.001482000	-5.080529000
1	-3.521779000	-5.340465000	-3.980342000
1	9.114071000	-0.537865000	0.482671000
1	-4.785291000	-1.871500000	3.105056000

1	-1.617246000	3.837734000	0.139765000
6	3.638518000	-4.104370000	0.526742000
1	3.239405000	-4.446674000	-0.433983000
1	3.222526000	-4.746343000	1.309186000
6	5.154661000	-4.268712000	0.580555000
8	5.785584000	-4.207274000	1.635033000
7	5.773793000	-4.423653000	-0.622558000
1	6.774578000	-4.572631000	-0.630411000
1	5.263577000	-4.599961000	-1.475466000
1	1.006037000	-4.269109000	1.174716000
6	3.518696000	3.652629000	-1.317716000
1	3.799064000	3.398334000	-2.344763000
1	3.435039000	4.743119000	-1.262677000
6	0.877267000	5.132021000	-0.290696000
1	-0.175301000	5.428313000	-0.334850000
1	1.385342000	5.719904000	-1.063257000
6	4.650065000	3.240807000	-0.382862000
8	4.634093000	3.386459000	0.824623000
8	5.662239000	2.670761000	-1.057732000
1	6.340019000	2.295153000	-0.437147000
6	1.457533000	5.510000000	1.083188000
1	0.916450000	4.997619000	1.888135000
1	2.499033000	5.173213000	1.169124000
6	1.407839000	6.996574000	1.346564000
8	1.007469000	7.842374000	0.571619000
8	1.877023000	7.297843000	2.578985000
1	1.821931000	8.267938000	2.677525000
6	-4.353133000	3.296156000	0.303663000
1	-3.787252000	3.843677000	1.065009000
1	-5.332403000	3.092476000	0.749853000
6	-5.664226000	0.372119000	-0.317634000
1	-6.318835000	1.222285000	-0.089207000
1	-6.038607000	-0.061159000	-1.251517000
6	-4.544987000	4.200424000	-0.924907000
1	-5.130972000	3.688128000	-1.698139000
1	-3.582197000	4.438762000	-1.392768000
6	-5.243254000	5.498373000	-0.587710000
8	-5.660210000	5.815954000	0.507761000
8	-5.360314000	6.293676000	-1.675489000
1	-5.817859000	7.107541000	-1.388685000
6	-5.860790000	-0.645783000	0.796932000
8	-5.204004000	-0.674911000	1.825924000
8	-6.842535000	-1.519598000	0.531403000
1	-6.932689000	-2.120303000	1.297285000
1	-4.292614000	-1.802767000	-1.348387000

e) Optimized reduced (doublet) and oxidized (triplet and singlet) state of **C** in aqueous solvent.

C-reduced-state-doublet-H2O
Reduced-state-doublet.gjf.log

28	-0.179921000	-0.158655000	-0.451600000
7	1.021757000	1.488141000	-0.431548000
7	1.374443000	-1.392157000	-0.598259000
7	-1.525561000	-1.693730000	-0.894035000
7	-1.668272000	0.953380000	0.175347000
6	0.612700000	2.776104000	-0.211060000
6	1.796651000	3.742860000	-0.245179000
6	2.358342000	1.446448000	-0.636399000
6	2.941320000	2.845908000	-0.785856000
6	-2.878568000	0.273290000	0.631683000
6	-1.745448000	2.245465000	0.365271000
6	-3.107292000	2.687650000	0.895281000
6	-3.729581000	1.342505000	1.367150000
6	-0.671527000	3.152708000	0.117288000
6	-2.803396000	-1.616837000	-1.028932000
6	-1.058008000	-3.030340000	-1.345158000
6	-2.331558000	-3.913654000	-1.237709000
6	-3.434070000	-2.876293000	-1.638621000
6	-3.591291000	-0.438281000	-0.524767000
6	1.271525000	-2.781601000	-0.379845000
6	2.659129000	-1.032522000	-0.573837000
6	2.591210000	-3.291398000	0.204089000
6	3.577451000	-2.242466000	-0.408028000
6	0.181261000	-3.522825000	-0.645418000
6	3.128833000	0.293514000	-0.689779000
1	3.828027000	-2.596629000	-1.420538000
6	4.892607000	-1.995921000	0.356701000
1	5.165972000	-2.883928000	0.930910000
1	4.756823000	-1.191202000	1.087244000
6	6.064183000	-1.666059000	-0.571290000
1	6.243328000	-2.500806000	-1.262166000
1	5.869145000	-0.794377000	-1.205844000
6	7.351360000	-1.420493000	0.180983000
8	7.516273000	-1.569238000	1.376151000
8	8.339627000	-1.014827000	-0.647585000
1	4.200014000	0.437949000	-0.770240000
6	2.512280000	-3.180693000	1.743204000
1	1.686190000	-3.798977000	2.110847000
1	3.432547000	-3.527468000	2.217746000
1	2.322946000	-2.144512000	2.044531000
1	-0.798474000	-2.919527000	-2.410069000

1	-2.297204000	-4.732644000	-1.965416000
6	-2.542560000	-4.544590000	0.155490000
1	-3.479584000	-5.112600000	0.138627000
1	-1.755438000	-5.282378000	0.337054000
6	-2.566319000	-3.573229000	1.349493000
1	-1.596112000	-3.066508000	1.440395000
1	-3.305004000	-2.781651000	1.217509000
6	-2.828342000	-4.293907000	2.662139000
8	-2.402556000	-5.406325000	2.911763000
8	-3.551836000	-3.634689000	3.585745000
6	-4.854097000	-3.201948000	-1.149706000
1	-5.575517000	-2.469959000	-1.525973000
1	-5.157708000	-4.186194000	-1.522941000
1	-4.938852000	-3.216588000	-0.060903000
6	-3.467695000	-2.760635000	-3.202457000
1	-3.919737000	-3.677014000	-3.596893000
1	-2.451443000	-2.696536000	-3.598299000
6	-4.188464000	-1.516947000	-3.706792000
8	-3.651685000	-0.406054000	-3.671823000
7	-5.447418000	-1.698402000	-4.174190000
1	-5.968279000	-0.898739000	-4.509695000
1	-5.887462000	-2.605874000	-4.216709000
1	-3.784745000	0.252933000	-1.353064000
1	-4.565119000	-0.791286000	-0.171534000
1	-2.557420000	-0.504658000	1.327680000
1	-4.783487000	1.263768000	1.084523000
6	-3.618081000	1.190379000	2.890440000
1	-4.215848000	1.955655000	3.402582000
1	-2.577158000	1.356392000	3.204926000
6	-4.023172000	-0.169401000	3.428170000
8	-4.416028000	-1.105342000	2.745160000
8	-3.904359000	-0.351441000	4.751667000
1	-2.982069000	3.385742000	1.733600000
1	3.857671000	2.953505000	-0.197400000
1	1.587073000	4.580867000	-0.922052000
6	2.079141000	4.304736000	1.163444000
1	2.401664000	3.485281000	1.818109000
1	1.148272000	4.691596000	1.591115000
6	3.130683000	5.413903000	1.166883000
1	4.062608000	5.092064000	0.689757000
1	2.792467000	6.275211000	0.576167000
6	3.457072000	5.903287000	2.557295000
8	2.994273000	5.470098000	3.595015000
8	4.358312000	6.911778000	2.527884000
6	3.284941000	3.113147000	-2.260776000
1	3.925156000	2.308965000	-2.647605000

1	2.382208000	3.114629000	-2.880960000
6	4.041860000	4.406379000	-2.470434000
8	4.723800000	4.971081000	-1.636444000
8	3.907913000	4.861962000	-3.732744000
1	9.142001000	-0.889985000	-0.104584000
1	4.454821000	5.667922000	-3.813724000
1	4.527200000	7.176972000	3.452729000
1	-3.571906000	0.451219000	5.192957000
1	-3.880272000	-2.761026000	3.262277000
1	-0.860690000	4.207090000	0.295282000
6	-3.917452000	3.398964000	-0.206519000
1	-3.309143000	4.198893000	-0.643367000
1	-4.129821000	2.693725000	-1.018864000
6	-5.225661000	3.995406000	0.312873000
1	-5.871129000	3.230806000	0.762804000
1	-5.035643000	4.727693000	1.108291000
6	-6.023181000	4.684102000	-0.771321000
8	-5.701347000	4.778672000	-1.939489000
8	-7.173610000	5.200832000	-0.285371000
1	-7.639041000	5.628312000	-1.030322000
6	2.908105000	-4.742023000	-0.236519000
1	2.709568000	-4.838250000	-1.308965000
1	2.239707000	-5.433291000	0.287317000
6	4.332817000	-5.171406000	0.091662000
8	4.698255000	-5.431714000	1.240275000
7	5.191800000	-5.205818000	-0.961255000
1	6.148389000	-5.493853000	-0.802217000
1	4.890864000	-5.067384000	-1.914690000
1	0.227674000	-4.588962000	-0.447999000

Triplet

C-oxidized-state-triplet-H2O

Oxidized-state-triplet.gjf.log

28	-0.133846000	-0.196769000	-0.189236000
7	0.941249000	1.503907000	-0.308713000
7	1.491597000	-1.320517000	-0.412649000
7	-1.367994000	-1.706985000	-0.922091000
7	-1.707044000	0.843035000	0.368787000
6	0.452855000	2.773931000	-0.111245000
6	1.575306000	3.801883000	-0.185934000
6	2.281951000	1.545025000	-0.551328000
6	2.747031000	2.974479000	-0.770693000
6	-2.922493000	0.104994000	0.696692000
6	-1.868184000	2.132756000	0.476428000
6	-3.276709000	2.506897000	0.921836000
6	-3.865313000	1.135251000	1.367421000

6	-0.842900000	3.089289000	0.216018000
6	-2.649270000	-1.680680000	-1.075502000
6	-0.827280000	-3.026425000	-1.351438000
6	-2.069476000	-3.955220000	-1.280280000
6	-3.199926000	-2.962626000	-1.713258000
6	-3.509382000	-0.563851000	-0.556764000
6	1.455086000	-2.723477000	-0.271748000
6	2.746464000	-0.896150000	-0.450245000
6	2.811345000	-3.183941000	0.260173000
6	3.724173000	-2.060659000	-0.346848000
6	0.404797000	-3.486310000	-0.614557000
6	3.129701000	0.457642000	-0.593874000
1	3.956513000	-2.363091000	-1.379605000
6	5.046592000	-1.760103000	0.386045000
1	5.372510000	-2.643265000	0.939199000
1	4.894722000	-0.973913000	1.133257000
6	6.176559000	-1.366211000	-0.568412000
1	6.369247000	-2.181618000	-1.278199000
1	5.930992000	-0.491715000	-1.181212000
6	7.471590000	-1.082730000	0.157427000
8	7.668889000	-1.245938000	1.345569000
8	8.421426000	-0.624507000	-0.686973000
1	4.183671000	0.668880000	-0.723019000
6	2.772359000	-3.129110000	1.803778000
1	1.992791000	-3.804599000	2.171510000
1	3.724035000	-3.444157000	2.235026000
1	2.538591000	-2.116682000	2.150728000
1	-0.532632000	-2.911487000	-2.405061000
1	-1.978433000	-4.764220000	-2.012848000
6	-2.297195000	-4.607795000	0.099539000
1	-3.200793000	-5.224396000	0.039606000
1	-1.479349000	-5.304412000	0.304447000
6	-2.420722000	-3.654456000	1.301531000
1	-1.488548000	-3.089474000	1.435001000
1	-3.209829000	-2.914543000	1.157241000
6	-2.674112000	-4.413127000	2.596540000
8	-2.171523000	-5.492986000	2.842835000
8	-3.477050000	-3.827434000	3.502325000
6	-4.621940000	-3.354222000	-1.284424000
1	-5.360709000	-2.660078000	-1.696508000
1	-4.856485000	-4.351791000	-1.669790000
1	-4.754021000	-3.374920000	-0.200773000
6	-3.178388000	-2.832536000	-3.275511000
1	-3.550291000	-3.775114000	-3.689566000
1	-2.154983000	-2.697508000	-3.634160000
6	-3.962491000	-1.633505000	-3.794004000

8	-3.547572000	-0.483264000	-3.625484000
7	-5.126815000	-1.903566000	-4.427535000
1	-5.675127000	-1.138250000	-4.798133000
1	-5.454354000	-2.845075000	-4.587324000
1	-3.661556000	0.166577000	-1.357771000
1	-4.494298000	-0.967421000	-0.307194000
1	-2.642474000	-0.687252000	1.391453000
1	-4.890659000	1.007247000	1.010435000
6	-3.857837000	0.991466000	2.895493000
1	-4.513161000	1.742827000	3.353134000
1	-2.849045000	1.187405000	3.287544000
6	-4.263036000	-0.381636000	3.402318000
8	-4.495357000	-1.346554000	2.686379000
8	-4.328298000	-0.539454000	4.730258000
1	-3.218845000	3.209005000	1.763340000
1	3.686837000	3.164321000	-0.245295000
1	1.289354000	4.626052000	-0.849270000
6	1.872581000	4.372051000	1.218195000
1	2.269162000	3.572401000	1.855872000
1	0.934557000	4.697719000	1.679474000
6	2.849926000	5.546759000	1.190118000
1	3.786196000	5.287551000	0.685030000
1	2.439030000	6.383623000	0.611571000
6	3.183414000	6.051900000	2.573646000
8	2.788980000	5.577163000	3.621213000
8	4.002965000	7.125847000	2.522026000
6	2.972144000	3.210741000	-2.274152000
1	3.654060000	2.453454000	-2.681235000
1	2.035250000	3.110442000	-2.833706000
6	3.582732000	4.563484000	-2.571054000
8	4.106425000	5.298430000	-1.756662000
8	3.503339000	4.858463000	-3.883265000
1	9.232357000	-0.477319000	-0.162496000
1	3.941870000	5.721124000	-4.019620000
1	4.183280000	7.398874000	3.442428000
1	-4.118244000	0.287814000	5.200385000
1	-3.861836000	-2.976199000	3.183331000
1	-1.089306000	4.135789000	0.359492000
6	-4.054910000	3.188340000	-0.221922000
1	-3.458744000	4.014006000	-0.625362000
1	-4.199212000	2.480602000	-1.046314000
6	-5.409397000	3.728822000	0.236366000
1	-6.042296000	2.938213000	0.658263000
1	-5.287136000	4.470270000	1.036091000
6	-6.182764000	4.378197000	-0.889326000
8	-5.817010000	4.461705000	-2.045150000

8	-7.364002000	4.868785000	-0.455788000
1	-7.814342000	5.270009000	-1.224262000
6	3.189371000	-4.597037000	-0.245066000
1	2.963086000	-4.665740000	-1.313947000
1	2.575667000	-5.341159000	0.273307000
6	4.646804000	-4.956015000	0.025901000
8	5.065450000	-5.206533000	1.157778000
7	5.467467000	-4.927650000	-1.057062000
1	6.437430000	-5.189818000	-0.937323000
1	5.119083000	-4.832946000	-1.999892000
1	0.497239000	-4.560365000	-0.491635000

Singlet

C-oxidized-state-singlet-H2O

Oxidized-state-singlet.gjf.log

28	0.161084000	-0.129503000	0.362337000
7	-0.966662000	1.453277000	0.324591000
7	-1.369225000	-1.313810000	0.539073000
7	1.410522000	-1.600971000	0.898249000
7	1.628392000	0.879657000	-0.282747000
6	-0.543477000	2.741097000	0.086196000
6	-1.702770000	3.718779000	0.164506000
6	-2.296653000	1.460365000	0.634144000
6	-2.796864000	2.873457000	0.852459000
6	2.825845000	0.142945000	-0.708247000
6	1.746454000	2.161659000	-0.519158000
6	3.099227000	2.528324000	-1.105694000
6	3.676260000	1.146825000	-1.518713000
6	0.723176000	3.107951000	-0.276631000
6	2.678965000	-1.509170000	1.125027000
6	0.902592000	-2.900713000	1.436422000
6	2.181706000	-3.766807000	1.552166000
6	3.231336000	-2.677136000	1.943385000
6	3.528252000	-0.447924000	0.506947000
6	-1.280278000	-2.708398000	0.280264000
6	-2.651058000	-0.961302000	0.556782000
6	-2.588239000	-3.165992000	-0.349620000
6	-3.564591000	-2.160242000	0.349219000
6	-0.232070000	-3.457417000	0.634626000
6	-3.107782000	0.356216000	0.727377000
1	-3.782456000	-2.569361000	1.347868000
6	-4.895726000	-1.864389000	-0.368100000
1	-5.176431000	-2.715439000	-0.991548000
1	-4.776871000	-1.016569000	-1.050886000
6	-6.046955000	-1.598485000	0.604700000
1	-6.204883000	-2.473896000	1.248431000

1	-5.845576000	-0.763181000	1.284596000
6	-7.350116000	-1.321607000	-0.109456000
8	-7.533885000	-1.410421000	-1.307570000
8	-8.323065000	-0.965782000	0.757296000
1	-4.165203000	0.524876000	0.881896000
6	-2.513575000	-2.934881000	-1.875316000
1	-1.694370000	-3.529349000	-2.293077000
1	-3.439449000	-3.241580000	-2.365087000
1	-2.320530000	-1.880468000	-2.100775000
1	0.515956000	-2.683943000	2.442122000
1	2.070085000	-4.491303000	2.365646000
6	2.538467000	-4.557016000	0.275044000
1	3.469514000	-5.103019000	0.461103000
1	1.780177000	-5.327531000	0.109319000
6	2.685178000	-3.744567000	-1.021925000
1	1.740645000	-3.242035000	-1.270392000
1	3.431129000	-2.952294000	-0.930082000
6	3.047450000	-4.624264000	-2.210108000
8	2.744501000	-5.799428000	-2.290163000
8	3.710845000	-4.033189000	-3.218790000
6	4.698001000	-3.034753000	1.663956000
1	5.367224000	-2.246954000	2.023477000
1	4.955678000	-3.956499000	2.195324000
1	4.907385000	-3.191753000	0.603982000
6	3.085781000	-2.364308000	3.473213000
1	3.488319000	-3.220200000	4.023527000
1	2.032330000	-2.265474000	3.746140000
6	3.745551000	-1.054499000	3.885732000
8	3.267427000	0.030547000	3.539984000
7	4.873347000	-1.151973000	4.625014000
1	5.335255000	-0.305749000	4.932227000
1	5.249033000	-2.037352000	4.932333000
1	3.764284000	0.317597000	1.253038000
1	4.476191000	-0.895268000	0.193927000
1	2.490336000	-0.683742000	-1.335207000
1	4.730121000	1.056768000	-1.242814000
6	3.547109000	0.900240000	-3.027630000
1	4.143150000	1.630832000	-3.588986000
1	2.505523000	1.042086000	-3.350018000
6	3.963309000	-0.495041000	-3.458109000
8	4.347595000	-1.367120000	-2.690248000
8	3.869751000	-0.782492000	-4.761274000
1	2.952816000	3.178555000	-1.977114000
1	-3.778031000	3.010917000	0.390732000
1	-1.419781000	4.591176000	0.763172000
6	-2.098451000	4.190484000	-1.252142000

1	-2.484758000	3.336557000	-1.822174000
1	-1.202730000	4.534294000	-1.779356000
6	-3.132876000	5.315448000	-1.235496000
1	-4.027976000	5.040769000	-0.667943000
1	-2.736912000	6.202965000	-0.726296000
6	-3.558899000	5.723068000	-2.626059000
8	-3.169448000	5.226414000	-3.665213000
8	-4.454556000	6.734735000	-2.590662000
6	-2.923846000	3.144328000	2.361122000
1	-3.555657000	2.383564000	2.835776000
1	-1.947754000	3.083049000	2.856404000
6	-3.539567000	4.494780000	2.658329000
8	-4.053462000	5.233472000	1.841192000
8	-3.468665000	4.784482000	3.971812000
1	-9.137380000	-0.818080000	0.238205000
1	-3.900318000	5.650616000	4.108006000
1	-4.689353000	6.948421000	-3.514507000
1	3.546904000	-0.019377000	-5.274024000
1	3.945146000	-3.093042000	-3.024732000
1	0.927038000	4.153444000	-0.475725000
6	3.954202000	3.289578000	-0.071488000
1	3.376825000	4.130056000	0.328289000
1	4.176987000	2.635407000	0.778970000
6	5.256438000	3.820245000	-0.670729000
1	5.872135000	3.014548000	-1.089204000
1	5.055892000	4.505655000	-1.503878000
6	6.098983000	4.555459000	0.347757000
8	5.817262000	4.712921000	1.519242000
8	7.235345000	5.031076000	-0.205129000
1	7.733155000	5.491634000	0.498087000
6	-2.905404000	-4.643277000	-0.015246000
1	-2.707597000	-4.818369000	1.046983000
1	-2.237452000	-5.292984000	-0.590166000
6	-4.333400000	-5.042655000	-0.372280000
8	-4.704964000	-5.194410000	-1.537452000
7	-5.184364000	-5.166340000	0.679762000
1	-6.136925000	-5.457644000	0.502078000
1	-4.869114000	-5.147353000	1.638692000
1	-0.243682000	-4.521658000	0.433059000

f) Optimized reduced (doublet) and oxidized (triplet and singlet) state of **C** in epsilon=4.

C-reduced-state-doublet-eps4

Reduced-state-doublet.gjf.log

Temperature   298.150 Kelvin. Pressure   1.00000 Atm.

Zero-point correction= 0.948754 (Hartree/Particle)
Thermal correction to Energy= 1.011288
Thermal correction to Enthalpy= 1.012232
Thermal correction to Gibbs Free Energy= 0.837793
Sum of electronic and zero-point Energies= -3182.767516
Sum of electronic and thermal Energies= -3182.704982
Sum of electronic and thermal Enthalpies= -3182.704038
Sum of electronic and thermal Free Energies= -3182.878477

6	-4.447103000	0.771047000	1.324081000
6	-1.468392000	-4.339402000	1.636295000
6	6.659595000	-2.402424000	1.972885000
6	1.871936000	5.976140000	1.273618000
7	-2.117894000	0.812319000	-0.836244000
6	-5.687274000	1.289828000	2.054200000
6	-0.116402000	-4.773437000	2.232646000
7	-1.150926000	-1.974399000	-0.550586000
8	6.236967000	-1.929278000	3.009064000
8	2.656512000	5.967483000	2.205371000
7	0.687114000	1.608370000	-0.313418000
8	-6.797399000	1.362421000	4.145581000
8	0.013645000	-5.903778000	4.380853000
8	-4.972080000	0.046204000	4.020359000
8	-0.472392000	-3.704071000	4.317703000
6	-1.238857000	3.089125000	-0.827432000
6	-3.519494000	-1.305827000	-0.571033000
6	0.852586000	-3.379310000	-0.569646000
6	3.010789000	0.906376000	-0.501328000
28	-0.234140000	-0.164131000	-0.648079000
6	-2.337950000	2.076906000	-0.723862000
6	-2.540654000	-2.217063000	-0.487806000
6	1.821815000	-2.415403000	-0.769417000
6	1.978861000	1.851284000	-0.255899000
6	-3.813901000	2.470512000	-0.574979000
6	-2.800826000	-3.729145000	-0.493797000
6	3.273358000	-2.754352000	-1.069569000
6	2.322406000	3.262477000	0.209515000
6	-4.418209000	1.084004000	-0.184954000
6	-1.461185000	-4.242079000	0.093026000
6	3.991739000	-1.432726000	-0.713649000
6	0.959036000	3.975889000	0.088503000
6	-3.437200000	0.137840000	-0.928021000
6	-0.518426000	-3.140460000	-0.370933000
6	2.829911000	-0.442571000	-0.672237000
6	-0.054660000	2.812774000	0.099172000

6	-4.299789000	2.927896000	-1.994263000
6	3.414332000	-3.148726000	-2.548676000
6	3.481426000	3.966520000	-0.552759000
7	-3.238282000	-2.484066000	-3.666928000
6	-5.798923000	3.208198000	-2.051485000
6	-3.825095000	-3.401492000	-2.839538000
6	4.801095000	-3.637082000	-2.904898000
6	4.696198000	4.337080000	0.330580000
8	-6.299561000	4.207805000	-1.540054000
8	-5.043553000	-3.564480000	-2.802186000
8	5.654682000	-3.993358000	-2.116780000
6	-2.859382000	-4.212311000	-1.986684000
6	4.713206000	-1.482799000	0.649335000
7	-6.550631000	2.262845000	-2.679390000
8	4.985834000	-3.664668000	-4.242351000
6	-4.024327000	3.606886000	0.438478000
6	-4.069571000	-4.168837000	0.241850000
6	5.996654000	-2.312608000	0.619102000
6	0.680239000	5.053233000	1.159554000
6	-5.745928000	0.817538000	3.490242000
6	-0.173902000	-4.891104000	3.737128000
7	1.619017000	-1.077014000	-0.698644000
8	7.820321000	-3.097173000	1.910409000
8	2.043298000	6.732687000	0.173415000
1	-4.421034000	-0.315983000	1.449561000
1	-3.538097000	1.139104000	1.813136000
1	-2.233636000	-5.063504000	1.937265000
1	-1.756448000	-3.370945000	2.060048000
1	-5.756484000	2.381263000	2.061070000
1	-6.602771000	0.940210000	1.557528000
1	0.191640000	-5.745053000	1.836587000
1	0.655635000	-4.039823000	1.975640000
1	-0.867752000	3.096580000	-1.861574000
1	-1.626190000	4.089871000	-0.618744000
1	-4.539473000	-1.680719000	-0.572924000
1	1.179235000	-4.414033000	-0.535807000
1	3.619104000	-3.590814000	-0.455022000
1	2.593293000	3.184826000	1.273477000
1	-5.429073000	0.964968000	-0.589780000
1	-1.182994000	-5.219074000	-0.320400000
1	4.715479000	-1.140928000	-1.484935000
1	0.917610000	4.457258000	-0.896990000
1	-3.690755000	0.170541000	-2.003504000
1	-0.434979000	2.645053000	1.118423000
1	-4.015975000	2.183857000	-2.746072000
1	-3.789027000	3.861343000	-2.251574000

1	2.715793000	-3.959655000	-2.793811000
1	3.150053000	-2.311163000	-3.204386000
1	3.091119000	4.879182000	-1.007141000
1	3.821577000	3.339636000	-1.383455000
1	4.356682000	4.548115000	1.350723000
1	-1.854356000	-4.173757000	-2.420096000
1	-3.190005000	-5.255802000	-2.001970000
1	4.027296000	-1.886118000	1.404711000
1	4.953469000	-0.463608000	0.969749000
1	-3.584699000	3.365251000	1.410408000
1	-5.087754000	3.814224000	0.565015000
1	-3.567362000	4.535778000	0.083919000
1	-4.087003000	-5.259750000	0.348386000
1	-4.135315000	-3.719814000	1.236893000
1	-4.957714000	-3.884107000	-0.328166000
1	5.817179000	-3.330114000	0.255931000
1	6.721937000	-1.887616000	-0.086675000
1	0.525551000	4.590549000	2.137818000
1	-0.217584000	5.620807000	0.890417000
1	-6.781354000	1.009643000	5.056105000
1	-0.503811000	-3.855476000	5.281833000
1	8.187799000	-3.119685000	2.814905000
1	5.880572000	-4.022442000	-4.403575000
1	2.991427000	7.013402000	0.147333000
1	-7.548736000	2.408272000	-2.754288000
1	-6.148599000	1.469928000	-3.156557000
1	-3.850412000	-1.809771000	-4.109215000
1	4.033193000	1.266223000	-0.446626000
1	5.426769000	3.526326000	0.381177000
6	5.366780000	5.612096000	-0.125071000
8	4.796996000	6.681159000	-0.300326000
8	6.689922000	5.480756000	-0.306177000
1	7.041230000	6.355838000	-0.564974000
1	-2.289714000	-2.179648000	-3.489769000

Triplet

C-oxidized-state-triplet-eps4

Oxidized-state-triplet.gjf.log

Temperature 298.150 Kelvin. Pressure 1.00000 Atm.

Zero-point correction= 0.950252 (Hartree/Particle)
Thermal correction to Energy= 1.012375
Thermal correction to Enthalpy= 1.013319
Thermal correction to Gibbs Free Energy= 0.842976
Sum of electronic and zero-point Energies= -3182.636276

Sum of electronic and thermal Energies= -3182.574154
Sum of electronic and thermal Enthalpies= -3182.573209
Sum of electronic and thermal Free Energies= -3182.743553

28	-0.161226000	-0.159158000	-0.435149000
7	0.992953000	1.494525000	-0.398119000
7	1.434562000	-1.361689000	-0.552702000
7	-1.447181000	-1.721991000	-0.879164000
7	-1.688138000	0.905522000	0.211751000
6	0.548403000	2.774394000	-0.179365000
6	1.708665000	3.760726000	-0.207585000
6	2.333307000	1.491118000	-0.626776000
6	2.855697000	2.906269000	-0.801569000
6	-2.886183000	0.190078000	0.648845000
6	-1.791939000	2.196204000	0.384651000
6	-3.165020000	2.603808000	0.901793000
6	-3.776175000	1.245763000	1.354410000
6	-0.741983000	3.125680000	0.137553000
6	-2.724373000	-1.665129000	-1.044633000
6	-0.933201000	-3.045741000	-1.328717000
6	-2.193106000	-3.949510000	-1.266830000
6	-3.300215000	-2.928123000	-1.696273000
6	-3.557975000	-0.530048000	-0.524058000
6	1.361760000	-2.754371000	-0.314552000
6	2.704553000	-0.967104000	-0.547525000
6	2.695235000	-3.215037000	0.270409000
6	3.649246000	-2.149023000	-0.369580000
6	0.290895000	-3.514433000	-0.590319000
6	3.136667000	0.369873000	-0.688044000
1	3.891275000	-2.507494000	-1.382583000
6	4.966570000	-1.847915000	0.371934000
1	5.257890000	-2.708435000	0.977359000
1	4.826078000	-1.020177000	1.074510000
6	6.121814000	-1.535425000	-0.582371000
1	6.330478000	-2.404503000	-1.219459000
1	5.892573000	-0.712761000	-1.270595000
6	7.395954000	-1.182859000	0.151898000
8	7.531625000	-1.147805000	1.357600000
8	8.399954000	-0.904165000	-0.709925000
1	4.200334000	0.542838000	-0.792566000
6	2.624848000	-3.079463000	1.808153000
1	1.819229000	-3.713784000	2.192337000
1	3.557430000	-3.397508000	2.276807000
1	2.412726000	-2.044010000	2.097371000
1	-0.639155000	-2.917673000	-2.381487000
1	-2.118779000	-4.755661000	-2.004965000

6	-2.440194000	-4.602141000	0.109392000
1	-3.360760000	-5.192873000	0.047039000
1	-1.645333000	-5.324939000	0.314005000
6	-2.537129000	-3.650190000	1.314206000
1	-1.587761000	-3.115423000	1.451530000
1	-3.300000000	-2.882884000	1.170289000
6	-2.818216000	-4.406620000	2.606532000
8	-2.372499000	-5.512462000	2.835446000
8	-3.576777000	-3.779062000	3.523801000
6	-4.733112000	-3.285403000	-1.274349000
1	-5.449754000	-2.564994000	-1.681222000
1	-4.996018000	-4.272356000	-1.668953000
1	-4.867858000	-3.313497000	-0.191156000
6	-3.271785000	-2.787284000	-3.257900000
1	-3.705598000	-3.699835000	-3.679627000
1	-2.241942000	-2.718250000	-3.616692000
6	-3.969208000	-1.531795000	-3.766675000
8	-3.471155000	-0.417512000	-3.590742000
7	-5.157636000	-1.709408000	-4.391531000
1	-5.633736000	-0.902555000	-4.773936000
1	-5.539243000	-2.623605000	-4.585589000
1	-3.774522000	0.161613000	-1.345148000
1	-4.516239000	-0.931455000	-0.181834000
1	-2.559897000	-0.576550000	1.354714000
1	-4.813982000	1.150041000	1.023408000
6	-3.736865000	1.089538000	2.880992000
1	-4.357357000	1.857331000	3.360409000
1	-2.712932000	1.250974000	3.249677000
6	-4.173640000	-0.276322000	3.385469000
8	-4.445113000	-1.225806000	2.665197000
8	-4.220680000	-0.442458000	4.713438000
1	-3.051293000	3.294424000	1.747273000
1	3.794398000	3.046210000	-0.258804000
1	1.467606000	4.617626000	-0.846301000
6	2.000120000	4.268968000	1.221491000
1	2.326893000	3.426379000	1.843854000
1	1.069744000	4.634442000	1.667877000
6	3.045038000	5.383215000	1.269517000
1	3.996080000	5.078044000	0.822270000
1	2.723126000	6.249531000	0.678258000
6	3.308188000	5.850292000	2.682491000
8	2.766861000	5.425180000	3.683838000
8	4.242811000	6.826312000	2.713403000
6	3.109966000	3.173827000	-2.294897000
1	3.777212000	2.410881000	-2.714699000
1	2.178081000	3.111444000	-2.869657000

6	3.748535000	4.523272000	-2.546910000
8	4.179095000	5.272322000	-1.693317000
8	3.802290000	4.797684000	-3.865235000
1	9.188978000	-0.691311000	-0.174908000
1	4.242542000	5.663996000	-3.967615000
1	4.363145000	7.081390000	3.648509000
1	-3.980172000	0.373658000	5.187474000
1	-3.918448000	-2.911490000	3.206064000
1	-0.954424000	4.175566000	0.307555000
6	-3.970080000	3.317569000	-0.203618000
1	-3.369013000	4.129806000	-0.626442000
1	-4.164905000	2.620124000	-1.026649000
6	-5.290327000	3.891375000	0.310775000
1	-5.930216000	3.116061000	0.750507000
1	-5.117640000	4.623472000	1.110031000
6	-6.084319000	4.571850000	-0.783379000
8	-5.751097000	4.659639000	-1.947302000
8	-7.239665000	5.083224000	-0.305737000
1	-7.702487000	5.505933000	-1.054941000
6	3.046629000	-4.661881000	-0.153012000
1	2.837096000	-4.781225000	-1.221244000
1	2.405104000	-5.362565000	0.391564000
6	4.489044000	-5.043106000	0.166712000
8	4.885173000	-5.214642000	1.317788000
7	5.327688000	-5.117841000	-0.905715000
1	6.274570000	-5.437251000	-0.744634000
1	4.983862000	-5.140772000	-1.854727000
1	0.348494000	-4.576319000	-0.377447000

Singlet

C-oxidized-state-singlet-eps4

Oxidized-state-singlet.gjf.log

Temperature 298.150 Kelvin. Pressure 1.00000 Atm.

Zero-point correction= 0.951884 (Hartree/Particle)
Thermal correction to Energy= 1.013469
Thermal correction to Enthalpy= 1.014413
Thermal correction to Gibbs Free Energy= 0.846797
Sum of electronic and zero-point Energies= -3182.648424
Sum of electronic and thermal Energies= -3182.586838
Sum of electronic and thermal Enthalpies= -3182.585894
Sum of electronic and thermal Free Energies= -3182.753510

28	0.156874000	-0.126407000	0.364126000
7	-0.967190000	1.446804000	0.336753000

7	-1.367636000	-1.312524000	0.535851000
7	1.406944000	-1.593946000	0.903308000
7	1.616453000	0.873048000	-0.293071000
6	-0.548592000	2.735723000	0.093035000
6	-1.707556000	3.712288000	0.177820000
6	-2.298351000	1.454361000	0.649613000
6	-2.795885000	2.866678000	0.873705000
6	2.816978000	0.137969000	-0.717020000
6	1.736000000	2.156269000	-0.529243000
6	3.086268000	2.522432000	-1.120316000
6	3.663283000	1.140979000	-1.532567000
6	0.715181000	3.102109000	-0.281545000
6	2.675829000	-1.499725000	1.129610000
6	0.900463000	-2.892669000	1.446588000
6	2.181233000	-3.755640000	1.567928000
6	3.228505000	-2.662307000	1.955013000
6	3.523613000	-0.444161000	0.499888000
6	-1.276805000	-2.708713000	0.278749000
6	-2.651467000	-0.965699000	0.556735000
6	-2.581039000	-3.167712000	-0.356652000
6	-3.562064000	-2.164503000	0.340084000
6	-0.229858000	-3.454775000	0.642155000
6	-3.109213000	0.350256000	0.737650000
1	-3.786037000	-2.578543000	1.335680000
6	-4.889677000	-1.862248000	-0.382196000
1	-5.160508000	-2.701574000	-1.025620000
1	-4.772497000	-1.000761000	-1.047245000
6	-6.048043000	-1.621962000	0.588632000
1	-6.232264000	-2.523053000	1.187540000
1	-5.837123000	-0.824238000	1.311195000
6	-7.333314000	-1.270543000	-0.126941000
8	-7.472455000	-1.184707000	-1.329652000
8	-8.341701000	-1.057845000	0.748062000
1	-4.167120000	0.516650000	0.892565000
6	-2.499729000	-2.934809000	-1.881993000
1	-1.677774000	-3.527670000	-2.296509000
1	-3.421615000	-3.244070000	-2.376736000
1	-2.307730000	-1.879638000	-2.105714000
1	0.510967000	-2.671355000	2.450575000
1	2.069979000	-4.475857000	2.385735000
6	2.539018000	-4.552268000	0.295271000
1	3.469270000	-5.098894000	0.483888000
1	1.782442000	-5.324407000	0.130549000
6	2.687668000	-3.747279000	-1.005812000
1	1.747851000	-3.234076000	-1.250398000
1	3.445187000	-2.964402000	-0.922515000

6	3.028067000	-4.643303000	-2.191050000
8	2.707560000	-5.812639000	-2.253025000
8	3.687962000	-4.066605000	-3.211042000
6	4.696154000	-3.019288000	1.679860000
1	5.363501000	-2.227772000	2.035254000
1	4.955170000	-3.937227000	2.217305000
1	4.907318000	-3.184063000	0.621457000
6	3.081866000	-2.343147000	3.483047000
1	3.487159000	-3.195307000	4.037943000
1	2.027693000	-2.247046000	3.754984000
6	3.734539000	-1.027176000	3.888466000
8	3.271122000	0.050081000	3.506658000
7	4.842397000	-1.112600000	4.661538000
1	5.283392000	-0.259142000	4.979487000
1	5.194591000	-1.990533000	5.013939000
1	3.763031000	0.326381000	1.239537000
1	4.468607000	-0.896299000	0.184029000
1	2.485124000	-0.693455000	-1.339670000
1	4.718121000	1.053022000	-1.259265000
6	3.531616000	0.888848000	-3.040565000
1	4.121670000	1.621829000	-3.605661000
1	2.487632000	1.023455000	-3.359336000
6	3.955684000	-0.507335000	-3.467055000
8	4.336493000	-1.374798000	-2.694679000
8	3.870553000	-0.795811000	-4.771114000
1	2.936115000	3.171052000	-1.992816000
1	-3.780290000	3.006137000	0.419573000
1	-1.424662000	4.586096000	0.773854000
6	-2.109804000	4.180450000	-1.238195000
1	-2.480565000	3.321278000	-1.811138000
1	-1.218605000	4.537040000	-1.764458000
6	-3.160600000	5.290462000	-1.229914000
1	-4.069844000	4.995849000	-0.696572000
1	-2.794607000	6.173955000	-0.692488000
6	-3.542965000	5.715168000	-2.629140000
8	-3.081430000	5.264737000	-3.658848000
8	-4.485103000	6.683968000	-2.610120000
6	-2.910304000	3.135590000	2.384118000
1	-3.545696000	2.381627000	2.864053000
1	-1.930439000	3.062263000	2.871598000
6	-3.500898000	4.496815000	2.685396000
8	-3.928733000	5.278937000	1.860404000
8	-3.507672000	4.741831000	4.010010000
1	-9.137725000	-0.842121000	0.224669000
1	-3.910072000	5.622035000	4.144706000
1	-4.683922000	6.912159000	-3.538770000

1	3.549659000	-0.035083000	-5.287815000
1	3.935683000	-3.130888000	-3.024705000
1	0.917471000	4.147664000	-0.482107000
6	3.945313000	3.286004000	-0.090903000
1	3.368360000	4.123821000	0.315008000
1	4.175181000	2.633369000	0.758664000
6	5.242807000	3.821743000	-0.696234000
1	5.854032000	3.019772000	-1.129098000
1	5.037559000	4.517717000	-1.519432000
6	6.095425000	4.541397000	0.326635000
8	5.831757000	4.662607000	1.505270000
8	7.215945000	5.043835000	-0.235270000
1	7.720921000	5.491057000	0.471287000
6	-2.896781000	-4.645437000	-0.025059000
1	-2.700167000	-4.822308000	1.037531000
1	-2.226729000	-5.293558000	-0.599657000
6	-4.323652000	-5.046352000	-0.388814000
8	-4.699652000	-5.157568000	-1.553791000
7	-5.173036000	-5.208972000	0.665011000
1	-6.108533000	-5.543414000	0.471136000
1	-4.841248000	-5.280646000	1.615932000
1	-0.236574000	-4.519177000	0.441192000

g) Optimized reduced (doublet) and oxidized (triplet and singlet) state of **D** in aqueous solvent.

D-reduced-state-doublet-H2O

Reduced-state-doublet.gjf.log

28	-0.064069000	-0.283409000	-0.279378000
7	1.455063000	1.123346000	-0.329224000
7	1.274067000	-1.795307000	-0.359711000
7	-1.746401000	-1.560170000	-0.547082000
7	-1.336304000	1.201342000	0.116569000
6	1.294236000	2.482228000	-0.307681000
6	2.638931000	3.207126000	-0.314699000
6	2.772797000	0.817720000	-0.398929000
6	3.629740000	2.058205000	-0.621948000
6	-2.697583000	0.884859000	0.559288000
6	-1.155354000	2.498570000	0.076293000
6	-2.424929000	3.293456000	0.364443000
6	-3.329971000	2.223501000	1.027565000
6	0.090400000	3.136395000	-0.185158000
6	-2.984799000	-1.251227000	-0.720714000
6	-1.594994000	-3.029011000	-0.628513000
6	-3.013542000	-3.573295000	-0.325300000
6	-3.885253000	-2.464828000	-1.006707000

6	-3.488589000	0.151828000	-0.532980000
6	0.943624000	-3.226699000	-0.399823000
6	2.592494000	-1.671068000	-0.266950000
6	2.157429000	-3.978965000	0.242587000
6	3.310695000	-3.016997000	-0.182113000
6	-0.430285000	-3.532500000	0.218459000
6	3.309445000	-0.452198000	-0.312914000
1	-0.521971000	-4.620724000	0.315564000
1	-0.468420000	-3.102549000	1.222468000
1	3.587430000	-3.278679000	-1.214593000
6	4.595024000	-3.085544000	0.665026000
1	4.693085000	-4.089579000	1.090392000
1	4.531227000	-2.398374000	1.515444000
6	5.871708000	-2.811818000	-0.136427000
1	5.975719000	-3.539546000	-0.952512000
1	5.868269000	-1.830719000	-0.622883000
6	7.119659000	-2.901060000	0.712539000
8	7.162260000	-3.227323000	1.882574000
8	8.222816000	-2.573613000	0.003717000
1	4.390310000	-0.512929000	-0.303708000
6	2.035972000	-4.142332000	1.757584000
1	1.163822000	-4.748946000	2.018969000
1	2.915364000	-4.645168000	2.169741000
1	1.938442000	-3.169354000	2.252388000
6	2.228911000	-5.300425000	-0.537674000
7	1.009543000	-3.757811000	-1.776157000
6	1.681165000	-4.934430000	-1.918739000
8	1.815905000	-5.565810000	-2.962473000
1	-1.364693000	-3.280474000	-1.669901000
1	-3.162575000	-4.534922000	-0.830266000
6	-3.317097000	-3.803913000	1.169394000
1	-4.328600000	-4.218922000	1.248755000
1	-2.647346000	-4.582389000	1.548335000
6	-3.215891000	-2.587859000	2.107006000
1	-2.191686000	-2.196674000	2.135959000
1	-3.836564000	-1.758715000	1.767564000
6	-3.595110000	-2.954640000	3.534924000
8	-3.327684000	-4.028637000	4.040584000
8	-4.239737000	-2.019497000	4.254446000
6	-5.332832000	-2.359152000	-0.502215000
1	-5.900111000	-1.627158000	-1.085369000
1	-5.832616000	-3.327424000	-0.615120000
1	-5.403654000	-2.067177000	0.546919000
6	-3.932238000	-2.745386000	-2.549210000
1	-4.553938000	-3.632366000	-2.710298000
1	-2.932045000	-2.972151000	-2.926259000

6	-4.424605000	-1.558243000	-3.367310000
8	-3.707679000	-0.573290000	-3.563019000
7	-5.687291000	-1.643350000	-3.851687000
1	-6.058248000	-0.875794000	-4.396301000
1	-6.275480000	-2.450201000	-3.703157000
1	-3.434844000	0.690686000	-1.484956000
1	-4.543474000	0.117535000	-0.246187000
1	-2.603014000	0.195215000	1.400160000
1	3.237318000	-5.716622000	-0.617588000
1	1.586874000	-6.071734000	-0.093014000
1	0.572001000	-3.298631000	-2.564167000
1	-4.369164000	2.303017000	0.695947000
6	-3.289654000	2.361773000	2.555288000
1	-3.696700000	3.332511000	2.866306000
1	-2.246102000	2.344379000	2.902583000
6	-4.022091000	1.275934000	3.315950000
8	-4.598347000	0.326984000	2.801365000
8	-4.001040000	1.359419000	4.653992000
1	-2.209019000	4.119807000	1.054293000
1	4.490955000	2.067664000	0.053335000
1	2.653947000	3.974744000	-1.098874000
6	2.894607000	3.887710000	1.046219000
1	2.996402000	3.116296000	1.819916000
1	2.019059000	4.485285000	1.321028000
6	4.131868000	4.785194000	1.044434000
1	5.026785000	4.243701000	0.719198000
1	4.018911000	5.604880000	0.323148000
6	4.413533000	5.391333000	2.398132000
8	3.791196000	5.178120000	3.420888000
8	5.476958000	6.226378000	2.354425000
6	4.153192000	2.073487000	-2.068095000
1	4.664022000	1.127658000	-2.292284000
1	3.327452000	2.155381000	-2.782976000
6	5.153940000	3.178038000	-2.327671000
8	5.850681000	3.714585000	-1.487356000
8	5.222251000	3.489358000	-3.638219000
1	8.990425000	-2.660077000	0.601739000
1	5.914727000	4.171139000	-3.742766000
1	5.607646000	6.577241000	3.256656000
1	-3.508140000	2.145205000	4.952546000
1	-4.429880000	-1.204110000	3.729295000
1	0.102263000	4.222132000	-0.174124000
6	-3.000527000	3.890663000	-0.935151000
1	-2.211846000	4.443535000	-1.457776000
1	-3.308207000	3.081771000	-1.608094000
6	-4.181891000	4.827021000	-0.681423000

1	-4.997348000	4.318520000	-0.151988000
1	-3.887826000	5.665227000	-0.036586000
6	-4.756347000	5.400738000	-1.956932000
8	-4.365644000	5.163167000	-3.083019000
8	-5.789017000	6.234503000	-1.701862000
1	-6.113850000	6.567784000	-2.560753000

Triplet

D-oxidized-state-triplet-H2O

Oxidized-state-triplet.gjf.log

28	-0.053195000	-0.278043000	-0.287418000
7	1.415367000	1.161028000	-0.348795000
7	1.343153000	-1.770177000	-0.354446000
7	-1.684812000	-1.607258000	-0.516706000
7	-1.380346000	1.180979000	0.087424000
6	1.209848000	2.518071000	-0.350982000
6	2.532248000	3.273948000	-0.330041000
6	2.745795000	0.892886000	-0.400277000
6	3.557490000	2.157160000	-0.632758000
6	-2.715082000	0.817220000	0.569736000
6	-1.231743000	2.477437000	0.020481000
6	-2.516003000	3.233168000	0.330564000
6	-3.384482000	2.143909000	1.014719000
6	-0.008061000	3.143425000	-0.263918000
6	-2.930680000	-1.330916000	-0.705592000
6	-1.479968000	-3.072450000	-0.615384000
6	-2.884467000	-3.656884000	-0.333781000
6	-3.777309000	-2.572344000	-1.026248000
6	-3.498126000	0.040111000	-0.492746000
6	1.052990000	-3.214199000	-0.384892000
6	2.651072000	-1.593007000	-0.253169000
6	2.290930000	-3.909519000	0.273827000
6	3.410469000	-2.911226000	-0.159264000
6	-0.310667000	-3.548239000	0.238161000
6	3.325689000	-0.351078000	-0.296392000
1	-0.377899000	-4.637412000	0.333442000
1	-0.362471000	-3.122464000	1.243737000
1	3.687036000	-3.160266000	-1.194642000
6	4.700236000	-2.930598000	0.681334000
1	4.829741000	-3.929738000	1.108630000
1	4.617923000	-2.244755000	1.530981000
6	5.964266000	-2.621058000	-0.127331000
1	6.082654000	-3.344699000	-0.944915000
1	5.934561000	-1.639527000	-0.611624000
6	7.216765000	-2.681810000	0.718298000
8	7.269999000	-3.022570000	1.883559000

8	8.306333000	-2.311308000	0.011391000
1	4.406709000	-0.374110000	-0.281461000
6	2.168394000	-4.054914000	1.790467000
1	1.323674000	-4.697404000	2.055443000
1	3.067333000	-4.511613000	2.212977000
1	2.024120000	-3.080931000	2.271659000
6	2.413551000	-5.236742000	-0.489381000
7	1.142908000	-3.752540000	-1.751779000
6	1.855277000	-4.911360000	-1.875589000
8	2.008650000	-5.551293000	-2.908700000
1	-1.233077000	-3.299564000	-1.657327000
1	-2.995161000	-4.618530000	-0.846849000
6	-3.203969000	-3.902151000	1.155043000
1	-4.201832000	-4.351226000	1.214665000
1	-2.516507000	-4.660666000	1.542153000
6	-3.160636000	-2.687125000	2.097896000
1	-2.150133000	-2.264265000	2.155167000
1	-3.800077000	-1.875851000	1.748501000
6	-3.562538000	-3.067920000	3.516839000
8	-3.309550000	-4.149604000	4.012064000
8	-4.206939000	-2.134516000	4.237530000
6	-5.239651000	-2.521508000	-0.558457000
1	-5.814736000	-1.799017000	-1.145596000
1	-5.700622000	-3.504170000	-0.702813000
1	-5.349014000	-2.253796000	0.493834000
6	-3.775922000	-2.834587000	-2.572543000
1	-4.362241000	-3.740643000	-2.754849000
1	-2.761274000	-3.023517000	-2.931237000
6	-4.287510000	-1.652588000	-3.386548000
8	-3.618964000	-0.620855000	-3.496506000
7	-5.503941000	-1.797530000	-3.960965000
1	-5.877202000	-1.041894000	-4.520767000
1	-6.041534000	-2.649276000	-3.891655000
1	-3.514548000	0.576016000	-1.447256000
1	-4.536648000	-0.056210000	-0.165008000
1	-2.572921000	0.151299000	1.423163000
1	3.436724000	-5.615673000	-0.561488000
1	1.800294000	-6.024593000	-0.034194000
1	0.684490000	-3.328178000	-2.547618000
1	-4.421786000	2.182818000	0.671970000
6	-3.366317000	2.308753000	2.540089000
1	-3.815354000	3.268113000	2.825864000
1	-2.328994000	2.337871000	2.904339000
6	-4.068491000	1.203629000	3.303763000
8	-4.571511000	0.214217000	2.787962000
8	-4.101538000	1.316154000	4.637390000

1	-2.302508000	4.064830000	1.013320000
1	4.417869000	2.194493000	0.041274000
1	2.536698000	4.053288000	-1.100767000
6	2.738886000	3.935118000	1.050778000
1	2.840552000	3.152016000	1.812247000
1	1.842643000	4.506037000	1.313899000
6	3.952988000	4.862179000	1.095673000
1	4.869501000	4.350909000	0.783471000
1	3.837298000	5.693387000	0.388695000
6	4.181328000	5.444046000	2.470464000
8	3.534879000	5.191314000	3.468799000
8	5.224117000	6.304465000	2.475075000
6	4.073939000	2.173254000	-2.081598000
1	4.616181000	1.245053000	-2.302573000
1	3.243782000	2.220128000	-2.795147000
6	5.028330000	3.315677000	-2.354582000
8	5.605921000	3.973619000	-1.511143000
8	5.197143000	3.507080000	-3.677557000
1	9.078917000	-2.382569000	0.605071000
1	5.849765000	4.225523000	-3.791527000
1	5.321935000	6.638355000	3.387875000
1	-3.667166000	2.134927000	4.938554000
1	-4.389966000	-1.314241000	3.717624000
1	-0.023873000	4.227862000	-0.272758000
6	-3.125316000	3.812640000	-0.962655000
1	-2.361380000	4.388041000	-1.496908000
1	-3.420643000	2.995477000	-1.630898000
6	-4.328099000	4.715115000	-0.687696000
1	-5.123475000	4.183360000	-0.151089000
1	-4.047641000	5.558656000	-0.044007000
6	-4.930221000	5.277072000	-1.956142000
8	-4.535303000	5.061514000	-3.084979000
8	-5.990187000	6.070120000	-1.688385000
1	-6.332360000	6.399721000	-2.541968000

Singlet

D-oxidized-state-singlet-H2O

Oxidized-state-singlet.gjf.log

28	-0.041770000	-0.251197000	-0.280747000
7	1.345118000	1.128846000	-0.363209000
7	1.302604000	-1.702524000	-0.349449000
7	-1.588311000	-1.493211000	-0.632287000
7	-1.299544000	1.093449000	0.209121000
6	1.138676000	2.490570000	-0.388730000
6	2.448636000	3.257372000	-0.462118000
6	2.686868000	0.889623000	-0.435630000

6	3.461734000	2.143022000	-0.783906000
6	-2.613467000	0.679479000	0.723362000
6	-1.213728000	2.400679000	0.175430000
6	-2.466288000	3.096978000	0.678364000
6	-3.238503000	1.939369000	1.358677000
6	-0.063648000	3.114163000	-0.218863000
6	-2.825555000	-1.187406000	-0.860953000
6	-1.400145000	-2.943013000	-0.904302000
6	-2.827735000	-3.530404000	-0.842743000
6	-3.637224000	-2.344180000	-1.450446000
6	-3.434337000	0.095643000	-0.417078000
6	1.043154000	-3.158787000	-0.441749000
6	2.608380000	-1.544006000	-0.181737000
6	2.214513000	-3.856329000	0.319965000
6	3.361231000	-2.852687000	-0.003797000
6	-0.356889000	-3.534947000	0.020212000
6	3.295169000	-0.320411000	-0.255248000
1	-0.441560000	-4.626153000	0.003520000
1	-0.513890000	-3.199387000	1.047599000
1	3.752820000	-3.105374000	-0.999684000
6	4.553102000	-2.843014000	0.973823000
1	4.634447000	-3.830433000	1.437426000
1	4.376485000	-2.136783000	1.791822000
6	5.901529000	-2.552844000	0.306843000
1	6.093754000	-3.274487000	-0.498465000
1	5.946222000	-1.567490000	-0.167670000
6	7.053363000	-2.644712000	1.283357000
8	6.974406000	-3.005924000	2.440879000
8	8.217665000	-2.277743000	0.706114000
1	4.375352000	-0.336090000	-0.243696000
6	1.949050000	-4.021806000	1.815807000
1	1.087172000	-4.672490000	1.989144000
1	2.805410000	-4.480446000	2.316749000
1	1.753221000	-3.055035000	2.293123000
6	2.410529000	-5.172900000	-0.446971000
7	1.285200000	-3.657071000	-1.806207000
6	1.996055000	-4.822587000	-1.876722000
8	2.246227000	-5.447534000	-2.899218000
1	-1.041351000	-3.043478000	-1.932066000
1	-2.887953000	-4.404698000	-1.499982000
6	-3.292538000	-3.989220000	0.555653000
1	-4.291476000	-4.426284000	0.450263000
1	-2.649258000	-4.811861000	0.881982000
6	-3.338247000	-2.943925000	1.681606000
1	-2.349619000	-2.508222000	1.870882000
1	-3.987450000	-2.098847000	1.439537000

6	-3.806723000	-3.553852000	2.998155000
8	-3.742245000	-4.742506000	3.246733000
8	-4.284645000	-2.701056000	3.918577000
6	-5.140170000	-2.337791000	-1.137091000
1	-5.644439000	-1.505853000	-1.638385000
1	-5.588249000	-3.266092000	-1.505661000
1	-5.359002000	-2.265636000	-0.070612000
6	-3.471463000	-2.355911000	-3.009498000
1	-4.036715000	-3.208982000	-3.396919000
1	-2.425140000	-2.503145000	-3.287930000
6	-3.890517000	-1.043395000	-3.660460000
8	-3.240706000	-0.009935000	-3.473330000
7	-5.000295000	-1.078183000	-4.431018000
1	-5.305716000	-0.231696000	-4.893564000
1	-5.521324000	-1.926610000	-4.598329000
1	-3.506225000	0.784835000	-1.263778000
1	-4.454279000	-0.097325000	-0.072931000
1	-2.450683000	-0.102271000	1.464804000
1	3.435844000	-5.551880000	-0.421284000
1	1.754389000	-5.966441000	-0.068054000
1	0.901179000	-3.225468000	-2.636431000
1	-4.307524000	1.982612000	1.136587000
6	-3.059884000	1.949670000	2.881789000
1	-3.468928000	2.873218000	3.310584000
1	-1.992503000	1.935160000	3.144479000
6	-3.717852000	0.768643000	3.569726000
8	-4.341977000	-0.103076000	2.979098000
8	-3.581585000	0.675444000	4.896663000
1	-2.178373000	3.866450000	1.405282000
1	4.368404000	2.218475000	-0.178168000
1	2.399813000	4.011278000	-1.255441000
6	2.725724000	3.963730000	0.883967000
1	2.868620000	3.205961000	1.664408000
1	1.844468000	4.543461000	1.175145000
6	3.938266000	4.892522000	0.834179000
1	4.844596000	4.369501000	0.513168000
1	3.793195000	5.686705000	0.091282000
6	4.214123000	5.544250000	2.168585000
8	3.587234000	5.362711000	3.194405000
8	5.273558000	6.380723000	2.098444000
6	3.865311000	2.084745000	-2.267712000
1	4.417672000	1.160525000	-2.477518000
1	2.980505000	2.065083000	-2.914729000
6	4.748106000	3.242327000	-2.680782000
8	5.271857000	4.038459000	-1.926390000
8	4.910080000	3.278279000	-4.017592000

1	8.920237000	-2.370284000	1.378629000
1	5.507033000	4.024079000	-4.223552000
1	5.399998000	6.765961000	2.987198000
1	-3.063218000	1.417243000	5.258184000
1	-4.325718000	-1.768954000	3.589780000
1	-0.100648000	4.196882000	-0.221294000
6	-3.222804000	3.788261000	-0.474564000
1	-2.529666000	4.431811000	-1.026848000
1	-3.584763000	3.040794000	-1.188517000
6	-4.395113000	4.632285000	0.025845000
1	-5.121059000	4.030553000	0.586491000
1	-4.052864000	5.410061000	0.720217000
6	-5.140047000	5.307764000	-1.103879000
8	-4.882419000	5.200643000	-2.286531000
8	-6.156409000	6.065186000	-0.638647000
1	-6.597246000	6.470740000	-1.410359000

h) Optimized reduced (doublet) and oxidized (triplet and singlet) state of **D** in epsilon=4.

D-reduced-state-doublet-eps4
Reduced-state-doublet.gjf.log

Temperature 298.150 Kelvin. Pressure 1.00000 Atm.

Zero-point correction= 0.951577 (Hartree/Particle)
Thermal correction to Energy= 1.012685
Thermal correction to Enthalpy= 1.013629
Thermal correction to Gibbs Free Energy= 0.844487
Sum of electronic and zero-point Energies= -3182.786635
Sum of electronic and thermal Energies= -3182.725527
Sum of electronic and thermal Enthalpies= -3182.724582
Sum of electronic and thermal Free Energies= -3182.893724

6	-3.807211000	0.836761000	1.804904000
6	-2.262194000	-4.502674000	0.508994000
6	5.719552000	-2.719952000	3.112165000
6	2.025963000	6.094356000	0.523281000
7	-2.230100000	0.903120000	-1.023870000
6	-4.677355000	1.480062000	2.888943000
6	-1.137472000	-5.133782000	1.350216000
7	-1.206578000	-1.877712000	-1.169131000
8	5.084928000	-2.216184000	4.017547000
8	2.702602000	6.229304000	1.527102000
7	0.680325000	1.577229000	-0.463327000
8	-5.089970000	1.537831000	5.220472000

8	-1.605879000	-6.609644000	3.224404000
8	-3.818261000	-0.163290000	4.467217000
8	-2.045181000	-4.407227000	3.415256000
6	-1.188698000	3.080930000	-1.162490000
6	-3.455977000	-1.203316000	-0.427909000
6	0.616160000	-3.440527000	-0.790962000
6	2.962014000	0.727087000	-0.248952000
28	-0.251092000	-0.157970000	-0.880036000
6	-2.314133000	2.164513000	-0.798975000
6	-2.643227000	-1.932649000	-1.520572000
6	1.651520000	-2.540474000	-0.678214000
6	1.976867000	1.746102000	-0.272729000
6	-3.617792000	2.620064000	-0.135042000
6	-2.952151000	-3.464145000	-1.766979000
6	3.114139000	-2.962134000	-0.629979000
6	2.371580000	3.192065000	0.021936000
6	-4.174884000	1.235108000	0.364260000
6	-1.843109000	-4.158371000	-0.939767000
6	3.809210000	-1.673440000	-0.132929000
6	1.085292000	3.950707000	-0.368156000
6	-3.544567000	0.293479000	-0.696135000
6	-0.747619000	-3.099481000	-0.970189000
6	2.729056000	-0.622240000	-0.367021000
6	0.003205000	2.868490000	-0.223211000
6	-4.516461000	3.211197000	-1.275026000
6	3.574033000	-3.399016000	-2.029953000
6	3.654330000	3.692718000	-0.702348000
7	-2.847039000	-1.342542000	-2.841360000
6	-5.940919000	3.515120000	-0.817827000
6	-2.736996000	-2.225655000	-3.880061000
6	4.973100000	-3.973720000	-2.045318000
6	4.785719000	4.159110000	0.243904000
8	-6.199843000	4.412700000	-0.018545000
8	-2.675807000	-1.924889000	-5.065288000
8	5.595332000	-4.359831000	-1.075321000
6	-2.682969000	-3.624176000	-3.287178000
6	4.194735000	-1.723134000	1.360291000
7	-6.905338000	2.701310000	-1.328832000
8	5.464294000	-4.043672000	-3.301345000
6	-3.367234000	3.689424000	0.940668000
6	-4.389059000	-3.892157000	-1.459040000
6	5.399579000	-2.621399000	1.639761000
6	0.788741000	5.229888000	0.445110000
6	-4.459251000	0.844197000	4.245145000
6	-1.606109000	-5.496265000	2.739568000
7	1.516797000	-1.187715000	-0.621097000

8	6.830328000	-3.463853000	3.328772000
8	2.366495000	6.614588000	-0.670321000
1	-3.936232000	-0.243513000	1.909686000
1	-2.745193000	1.026372000	2.003055000
1	-3.102109000	-5.205476000	0.467852000
1	-2.622141000	-3.603472000	1.018840000
1	-4.517382000	2.555073000	2.995243000
1	-5.742684000	1.363094000	2.645240000
1	-0.758909000	-6.045334000	0.879694000
1	-0.304670000	-4.428359000	1.445494000
1	-0.861705000	2.871576000	-2.187060000
1	-1.509751000	4.125512000	-1.116037000
1	-2.966438000	-1.409780000	0.525860000
1	-4.478473000	-1.587252000	-0.373816000
1	0.862010000	-4.497320000	-0.764499000
1	3.259135000	-3.800392000	0.058138000
1	2.518295000	3.273631000	1.109948000
1	-5.266273000	1.205975000	0.273055000
1	-1.512799000	-5.085629000	-1.424269000
1	4.706033000	-1.441116000	-0.720712000
1	1.159549000	4.222387000	-1.429346000
1	-4.148125000	0.390259000	-1.609393000
1	-0.365749000	2.862248000	0.815021000
1	-4.523517000	2.533728000	-2.135341000
1	-4.075622000	4.155492000	-1.611521000
1	2.904541000	-4.173807000	-2.425871000
1	3.522149000	-2.563756000	-2.737855000
1	3.379896000	4.523907000	-1.354610000
1	4.042120000	2.909640000	-1.361560000
1	4.351281000	4.580692000	1.156980000
1	-1.679383000	-4.016979000	-3.489918000
1	-3.395596000	-4.287157000	-3.786269000
1	3.331443000	-2.066890000	1.943436000
1	4.414313000	-0.708377000	1.709365000
1	-2.689896000	3.327045000	1.719103000
1	-4.306929000	4.006218000	1.393810000
1	-2.915210000	4.581757000	0.495633000
1	-4.520589000	-4.954786000	-1.692330000
1	-4.666610000	-3.744205000	-0.412760000
1	-5.093431000	-3.324202000	-2.077907000
1	5.250930000	-3.635038000	1.252288000
1	6.293477000	-2.252088000	1.120971000
1	0.500538000	4.974926000	1.468420000
1	-0.030800000	5.785724000	-0.023783000
1	-4.924847000	1.067130000	6.060159000
1	-2.334796000	-4.711811000	4.296610000

1	6.971129000	-3.490502000	4.294756000
1	6.348309000	-4.455523000	-3.243568000
1	3.329982000	6.836698000	-0.635905000
1	-7.870242000	2.888120000	-1.089321000
1	-6.719842000	2.024374000	-2.054068000
1	-2.563777000	-0.381356000	-2.995637000
1	3.982060000	1.035266000	-0.041727000
1	5.440157000	3.333448000	0.533109000
6	5.601069000	5.282071000	-0.353542000
8	5.140946000	6.330573000	-0.786422000
8	6.920884000	5.039449000	-0.352178000
1	7.366667000	5.825879000	-0.725008000

Triplet

D-oxidized-state-triplet-eps4

Oxidized-state-triplet.gjf.log

Temperature 298.150 Kelvin. Pressure 1.00000 Atm.

Zero-point correction= 0.952365 (Hartree/Particle)
Thermal correction to Energy= 1.013346
Thermal correction to Enthalpy= 1.014290
Thermal correction to Gibbs Free Energy= 0.846341
Sum of electronic and zero-point Energies= -3182.652077
Sum of electronic and thermal Energies= -3182.591096
Sum of electronic and thermal Enthalpies= -3182.590151
Sum of electronic and thermal Free Energies= -3182.758101

28	-0.046824000	-0.273328000	-0.291380000
7	1.392506000	1.176164000	-0.362199000
7	1.359531000	-1.742359000	-0.346694000
7	-1.655242000	-1.617949000	-0.527669000
7	-1.386468000	1.151872000	0.116712000
6	1.170737000	2.531227000	-0.355761000
6	2.482062000	3.304324000	-0.338992000
6	2.729282000	0.929866000	-0.420958000
6	3.516783000	2.205256000	-0.667460000
6	-2.719116000	0.768839000	0.593566000
6	-1.261618000	2.452723000	0.047485000
6	-2.555007000	3.186962000	0.368217000
6	-3.401244000	2.082704000	1.055006000
6	-0.054697000	3.138714000	-0.249458000
6	-2.903441000	-1.353671000	-0.724147000
6	-1.435888000	-3.081022000	-0.639637000
6	-2.837397000	-3.682430000	-0.379873000
6	-3.731767000	-2.599777000	-1.072241000

6	-3.491479000	0.003843000	-0.484539000
6	1.091063000	-3.193456000	-0.385505000
6	2.667306000	-1.552017000	-0.250524000
6	2.333484000	-3.871892000	0.281095000
6	3.442197000	-2.859104000	-0.146554000
6	-0.272131000	-3.550518000	0.223452000
6	3.327107000	-0.303454000	-0.309209000
1	-0.325765000	-4.641283000	0.308605000
1	-0.341310000	-3.135829000	1.232698000
1	3.730351000	-3.107792000	-1.178943000
6	4.725588000	-2.856241000	0.704530000
1	4.863565000	-3.849771000	1.141703000
1	4.628919000	-2.166034000	1.549135000
6	5.994315000	-2.539433000	-0.094012000
1	6.127239000	-3.265543000	-0.907275000
1	5.962924000	-1.559992000	-0.582701000
6	7.237116000	-2.588146000	0.768417000
8	7.275759000	-2.929392000	1.932744000
8	8.331533000	-2.205287000	0.075120000
1	4.408403000	-0.313445000	-0.299456000
6	2.203177000	-4.019112000	1.796908000
1	1.365405000	-4.672668000	2.057017000
1	3.104782000	-4.464067000	2.225817000
1	2.043784000	-3.047501000	2.278492000
6	2.479530000	-5.196675000	-0.482375000
7	1.201774000	-3.722623000	-1.752050000
6	1.931606000	-4.875736000	-1.874490000
8	2.100767000	-5.506149000	-2.907001000
1	-1.172966000	-3.295460000	-1.680506000
1	-2.932428000	-4.639388000	-0.905306000
6	-3.168952000	-3.946441000	1.102767000
1	-4.162290000	-4.406957000	1.150306000
1	-2.478187000	-4.701756000	1.489958000
6	-3.146348000	-2.742521000	2.059290000
1	-2.149070000	-2.287347000	2.100010000
1	-3.820552000	-1.949191000	1.733378000
6	-3.499575000	-3.157366000	3.484045000
8	-3.200405000	-4.237951000	3.949627000
8	-4.146028000	-2.249895000	4.236106000
6	-5.201325000	-2.567933000	-0.626138000
1	-5.774932000	-1.849232000	-1.219965000
1	-5.649094000	-3.555063000	-0.781032000
1	-5.329673000	-2.307364000	0.425798000
6	-3.708344000	-2.843439000	-2.621405000
1	-4.321907000	-3.726431000	-2.827221000
1	-2.694294000	-3.064805000	-2.963065000

6	-4.156033000	-1.630364000	-3.427566000
8	-3.460890000	-0.613036000	-3.473874000
7	-5.348772000	-1.729607000	-4.060788000
1	-5.659544000	-0.960375000	-4.640159000
1	-5.889288000	-2.582100000	-4.071585000
1	-3.514876000	0.558864000	-1.427807000
1	-4.528123000	-0.115171000	-0.158050000
1	-2.575430000	0.090772000	1.436858000
1	3.507614000	-5.563399000	-0.545172000
1	1.872087000	-5.993148000	-0.034480000
1	0.748058000	-3.303005000	-2.552684000
1	-4.442556000	2.109280000	0.723206000
6	-3.372241000	2.235224000	2.581789000
1	-3.825119000	3.189795000	2.878768000
1	-2.332131000	2.267336000	2.938612000
6	-4.065846000	1.116963000	3.337751000
8	-4.561929000	0.131664000	2.811039000
8	-4.095838000	1.216686000	4.672616000
1	-2.348535000	4.019494000	1.052636000
1	4.391349000	2.255453000	-0.013037000
1	2.471546000	4.096358000	-1.095886000
6	2.688609000	3.943815000	1.052388000
1	2.782133000	3.148658000	1.802852000
1	1.793046000	4.512654000	1.321820000
6	3.903684000	4.867782000	1.125350000
1	4.830592000	4.354944000	0.850649000
1	3.815924000	5.691003000	0.405576000
6	4.077240000	5.463420000	2.503420000
8	3.359831000	5.257300000	3.462369000
8	5.154523000	6.277870000	2.556639000
6	3.997901000	2.229679000	-2.128455000
1	4.565706000	1.320855000	-2.362604000
1	3.147995000	2.242755000	-2.821451000
6	4.890780000	3.413964000	-2.431998000
8	5.333880000	4.194135000	-1.613318000
8	5.159815000	3.494954000	-3.749880000
1	9.096449000	-2.272838000	0.678935000
1	5.754769000	4.258668000	-3.882406000
1	5.208495000	6.626565000	3.467385000
1	-3.667947000	2.035299000	4.981946000
1	-4.364887000	-1.430628000	3.733265000
1	-0.086540000	4.222750000	-0.254271000
6	-3.182875000	3.762588000	-0.918105000
1	-2.431611000	4.349915000	-1.457218000
1	-3.471383000	2.945416000	-1.588942000
6	-4.396147000	4.647666000	-0.632331000

1	-5.179010000	4.105228000	-0.087465000
1	-4.123554000	5.497799000	0.005961000
6	-5.018069000	5.193464000	-1.899715000
8	-4.642369000	4.956508000	-3.029384000
8	-6.070451000	5.994056000	-1.624342000
1	-6.426121000	6.311069000	-2.477096000

Singlet

D-oxidized-state-singlet-eps4

Oxidized-state-singlet.gjf.log

Temperature 298.150 Kelvin. Pressure 1.00000 Atm.

Zero-point correction= 0.954530 (Hartree/Particle)
Thermal correction to Energy= 1.014856
Thermal correction to Enthalpy= 1.015800
Thermal correction to Gibbs Free Energy= 0.851141
Sum of electronic and zero-point Energies= -3182.657729
Sum of electronic and thermal Energies= -3182.597403
Sum of electronic and thermal Enthalpies= -3182.596458
Sum of electronic and thermal Free Energies= -3182.761118

28	-0.039333000	-0.248308000	-0.282631000
7	1.328327000	1.136072000	-0.373248000
7	1.313972000	-1.684098000	-0.346114000
7	-1.571640000	-1.501340000	-0.637856000
7	-1.305393000	1.074132000	0.217337000
6	1.109739000	2.496544000	-0.397733000
6	2.410765000	3.276699000	-0.474270000
6	2.674568000	0.913245000	-0.449054000
6	3.432673000	2.173939000	-0.804898000
6	-2.616217000	0.648506000	0.733057000
6	-1.236468000	2.383586000	0.181115000
6	-2.492886000	3.066401000	0.690958000
6	-3.247816000	1.900799000	1.376582000
6	-0.097735000	3.108365000	-0.220571000
6	-2.811734000	-1.204528000	-0.867903000
6	-1.373238000	-2.949428000	-0.915210000
6	-2.796273000	-3.547738000	-0.859365000
6	-3.612693000	-2.365166000	-1.464955000
6	-3.433381000	0.068204000	-0.411126000
6	1.068925000	-3.144345000	-0.448187000
6	2.620031000	-1.517578000	-0.182584000
6	2.244668000	-3.834738000	0.312419000
6	3.383134000	-2.819343000	-0.003718000
6	-0.327838000	-3.535952000	0.009716000

6	3.295949000	-0.288612000	-0.263764000
1	-0.401348000	-4.627937000	-0.011301000
1	-0.490933000	-3.206740000	1.038212000
1	3.782197000	-3.066921000	-0.998127000
6	4.569431000	-2.799844000	0.980925000
1	4.660676000	-3.788481000	1.439923000
1	4.378678000	-2.102321000	1.803263000
6	5.920086000	-2.489905000	0.327605000
1	6.126633000	-3.200722000	-0.483906000
1	5.961726000	-1.498738000	-0.135140000
6	7.062314000	-2.584403000	1.316771000
8	6.971834000	-2.965497000	2.465705000
8	8.228401000	-2.193443000	0.758393000
1	4.376329000	-0.294354000	-0.253458000
6	1.978414000	-4.011392000	1.807001000
1	1.121663000	-4.669947000	1.976630000
1	2.837426000	-4.466522000	2.306318000
1	1.775442000	-3.049482000	2.291470000
6	2.455793000	-5.145000000	-0.461702000
7	1.319185000	-3.629271000	-1.813631000
6	2.045929000	-4.788925000	-1.892204000
8	2.306561000	-5.398346000	-2.917740000
1	-1.009742000	-3.044247000	-1.942132000
1	-2.848119000	-4.419795000	-1.520752000
6	-3.257231000	-4.016710000	0.536512000
1	-4.252567000	-4.462290000	0.431752000
1	-2.608636000	-4.836166000	0.860000000
6	-3.309719000	-2.978236000	1.668015000
1	-2.330752000	-2.513274000	1.836173000
1	-3.989832000	-2.152774000	1.443936000
6	-3.726310000	-3.613174000	2.992506000
8	-3.614721000	-4.799359000	3.225306000
8	-4.205897000	-2.779893000	3.930554000
6	-5.116512000	-2.369900000	-1.155442000
1	-5.624400000	-1.540716000	-1.658172000
1	-5.557582000	-3.301007000	-1.525590000
1	-5.339122000	-2.301522000	-0.089402000
6	-3.445531000	-2.373038000	-3.023778000
1	-4.022205000	-3.216827000	-3.415476000
1	-2.401068000	-2.535621000	-3.301632000
6	-3.841939000	-1.050695000	-3.669416000
8	-3.200697000	-0.022226000	-3.440737000
7	-4.924580000	-1.072091000	-4.480411000
1	-5.194088000	-0.222026000	-4.958672000
1	-5.421940000	-1.921948000	-4.702541000
1	-3.510801000	0.765281000	-1.250633000

1	-4.451030000	-0.138728000	-0.068030000
1	-2.448440000	-0.136937000	1.469196000
1	3.483902000	-5.515835000	-0.432990000
1	1.804969000	-5.946900000	-0.091288000
1	0.939103000	-3.193412000	-2.643192000
1	-4.319448000	1.933799000	1.164405000
6	-3.056579000	1.908892000	2.898306000
1	-3.462378000	2.832002000	3.331718000
1	-1.986273000	1.894647000	3.150167000
6	-3.707424000	0.723636000	3.589664000
8	-4.316891000	-0.156362000	2.999324000
8	-3.575841000	0.640387000	4.918327000
1	-2.207199000	3.838688000	1.416590000
1	4.343646000	2.260539000	-0.206951000
1	2.352104000	4.034219000	-1.263271000
6	2.683768000	3.978426000	0.875116000
1	2.823417000	3.218265000	1.654071000
1	1.800437000	4.554706000	1.166806000
6	3.893725000	4.911411000	0.839659000
1	4.810430000	4.391362000	0.545093000
1	3.762899000	5.696564000	0.084881000
6	4.129626000	5.577313000	2.175809000
8	3.456324000	5.422389000	3.175439000
8	5.206933000	6.392144000	2.136712000
6	3.824967000	2.120217000	-2.292125000
1	4.390164000	1.205747000	-2.508898000
1	2.934506000	2.085964000	-2.931583000
6	4.678907000	3.297938000	-2.711282000
8	5.127184000	4.145958000	-1.966159000
8	4.902052000	3.288023000	-4.039611000
1	8.923560000	-2.292139000	1.437560000
1	5.470495000	4.054781000	-4.248828000
1	5.301732000	6.789421000	3.023933000
1	-3.070778000	1.391341000	5.278687000
1	-4.282688000	-1.850141000	3.609116000
1	-0.145038000	4.190671000	-0.224684000
6	-3.264687000	3.751815000	-0.455462000
1	-2.581223000	4.397726000	-1.016999000
1	-3.629183000	3.003139000	-1.166591000
6	-4.437019000	4.590606000	0.053814000
1	-5.150545000	3.988196000	0.630074000
1	-4.093278000	5.378991000	0.735334000
6	-5.202739000	5.246014000	-1.075447000
8	-4.968238000	5.110493000	-2.258682000
8	-6.206591000	6.016576000	-0.604367000
1	-6.661238000	6.407027000	-1.375764000

i) Optimized reduced (doublet) and oxidized (triplet and singlet) state of **E** in aqueous solvent.

E-reduced-state-doublet-H2O

Reduced-state-doublet.gjf.log

28	0.364930000	0.070707000	-0.385368000
7	-1.551689000	-0.754864000	-0.424897000
7	-0.373992000	1.952268000	-0.187924000
7	2.349942000	0.760275000	-0.655178000
7	1.057548000	-1.809041000	-0.215050000
6	-1.900324000	-2.058100000	-0.548403000
6	-3.417037000	-2.233656000	-0.532307000
6	-2.681641000	0.021647000	-0.388045000
6	-3.922537000	-0.790146000	-0.706808000
6	2.481247000	-2.033154000	0.094227000
6	0.414575000	-2.941016000	-0.253974000
6	1.267660000	-4.145148000	0.097411000
6	2.684893000	-3.562289000	0.272205000
6	-0.984921000	-3.130859000	-0.565251000
6	3.397236000	0.066144000	-0.939999000
6	2.736203000	2.182723000	-0.517951000
6	4.252801000	2.137399000	-0.206784000
6	4.676491000	0.906894000	-1.074221000
6	3.357727000	-1.430961000	-1.014614000
6	0.425006000	3.188208000	-0.116308000
6	-1.642955000	2.269749000	-0.015270000
6	-0.441220000	4.229170000	0.670949000
6	-1.865142000	3.757488000	0.242535000
6	1.831625000	2.941960000	0.444752000
6	-2.734825000	1.369427000	-0.140638000
1	2.292134000	3.916201000	0.646362000
1	1.739862000	2.414789000	1.397109000
1	-2.073687000	4.203545000	-0.741636000
6	-3.022537000	4.153067000	1.178764000
1	-2.759291000	5.076770000	1.703544000
1	-3.168698000	3.393453000	1.954256000
6	-4.341638000	4.416374000	0.445433000
1	-4.214990000	5.216451000	-0.296326000
1	-4.696029000	3.549415000	-0.121919000
6	-5.450509000	4.837625000	1.383347000
8	-5.336088000	5.030159000	2.577889000
8	-6.621321000	4.988743000	0.725959000
1	-3.728062000	1.795890000	-0.077662000
6	-0.220647000	4.180720000	2.183071000
1	0.813841000	4.428833000	2.437619000
1	-0.862185000	4.902353000	2.696344000

1	-0.442907000	3.184713000	2.582549000
6	-0.084012000	5.572427000	0.016652000
7	0.500099000	3.850051000	-1.430934000
6	0.264230000	5.192936000	-1.423887000
8	0.322404000	5.938734000	-2.395854000
1	2.618217000	2.651728000	-1.501638000
1	4.738562000	3.045774000	-0.581350000
6	4.598453000	2.041303000	1.292947000
1	5.687881000	2.028406000	1.401987000
1	4.267314000	2.966899000	1.776308000
6	4.020947000	0.858604000	2.082737000
1	2.932608000	0.789493000	1.976983000
1	4.410927000	-0.101789000	1.724925000
6	4.351051000	0.948943000	3.562194000
8	5.043707000	1.803823000	4.070651000
8	3.822542000	-0.023277000	4.346014000
6	5.975365000	0.211940000	-0.638988000
1	6.270553000	-0.553779000	-1.363144000
1	6.785555000	0.947849000	-0.590771000
1	5.895435000	-0.278825000	0.331503000
6	4.852385000	1.386896000	-2.556622000
1	5.757652000	2.001477000	-2.603808000
1	4.011054000	2.017503000	-2.855488000
6	4.895683000	0.247019000	-3.566136000
8	3.876185000	-0.374346000	-3.876523000
7	6.111501000	-0.046011000	-4.088639000
1	6.190175000	-0.795842000	-4.763095000
1	6.946297000	0.467608000	-3.847239000
1	2.977328000	-1.741561000	-1.993485000
1	4.368131000	-1.826634000	-0.914002000
1	2.702332000	-1.510714000	1.029581000
1	-0.892248000	6.308849000	0.039614000
1	0.795880000	6.031694000	0.485134000
1	0.739972000	3.358793000	-2.282091000
1	3.345848000	-3.943106000	-0.514364000
6	3.273424000	-3.941018000	1.636657000
1	3.334527000	-5.033218000	1.732835000
1	2.600448000	-3.604309000	2.439099000
6	4.641625000	-3.354080000	1.933658000
8	5.227108000	-2.549071000	1.237585000
8	5.217068000	-3.758179000	3.087975000
1	0.904570000	-4.549543000	1.054808000
6	1.072791000	-5.227692000	-0.962647000
6	-1.427110000	-4.497391000	-0.885647000
6	-0.399400000	-5.638988000	-0.941287000
1	1.352300000	-4.828502000	-1.946747000

1	1.719199000	-6.090951000	-0.765665000
1	-0.592299000	-6.251794000	-0.048213000
1	-0.667633000	-6.261708000	-1.801010000
8	-2.600738000	-4.812933000	-1.128735000
1	-4.740382000	-0.553145000	-0.020052000
1	-3.732523000	-2.889047000	-1.344519000
6	-3.863826000	-2.859919000	0.808365000
1	-3.738347000	-2.120485000	1.609422000
1	-3.218659000	-3.705929000	1.052724000
6	-5.312809000	-3.340089000	0.763302000
1	-5.994210000	-2.549509000	0.429860000
1	-5.420542000	-4.145810000	0.025688000
6	-5.799987000	-3.859199000	2.093887000
8	-5.160558000	-3.890597000	3.128010000
8	-7.075965000	-4.303267000	2.015552000
6	-4.389554000	-0.482063000	-2.139470000
1	-4.530973000	0.599295000	-2.267633000
1	-3.632724000	-0.782477000	-2.872429000
6	-5.707397000	-1.139122000	-2.486703000
8	-6.536694000	-1.532812000	-1.688932000
8	-5.887322000	-1.211648000	-3.821430000
1	-7.290061000	5.269074000	1.380607000
1	-6.768902000	-1.603284000	-3.978373000
1	-7.326928000	-4.621478000	2.904293000
1	4.653473000	-4.401348000	3.553427000
1	3.277321000	-0.635889000	3.822628000

Triplet

E-oxidized-state-triplet-H2O

Oxidized-state-triplet.gjf.log

28	-0.338772000	0.063033000	0.365935000
7	1.580946000	-0.710143000	0.399093000
7	0.344890000	1.982238000	0.187932000
7	-2.331204000	0.677017000	0.649086000
7	-0.991645000	-1.823335000	0.144273000
6	1.949815000	-2.010181000	0.547839000
6	3.465627000	-2.153156000	0.530480000
6	2.693805000	0.090272000	0.339614000
6	3.946624000	-0.696007000	0.662714000
6	-2.394472000	-2.054858000	-0.232852000
6	-0.324779000	-2.935435000	0.210935000
6	-1.140034000	-4.150221000	-0.175389000
6	-2.546516000	-3.581557000	-0.478409000
6	1.058975000	-3.094016000	0.578471000
6	-3.343655000	-0.052863000	0.971168000
6	-2.722179000	2.106614000	0.651034000

6	-4.262438000	2.067067000	0.500380000
6	-4.588821000	0.773837000	1.323272000
6	-3.324701000	-1.546223000	0.874421000
6	-0.486137000	3.201748000	0.127954000
6	1.601619000	2.307962000	-0.035541000
6	0.333910000	4.227753000	-0.723889000
6	1.782167000	3.789366000	-0.336638000
6	-1.912778000	2.912610000	-0.358060000
6	2.715296000	1.432478000	0.070287000
1	-2.413398000	3.873483000	-0.518016000
1	-1.869129000	2.394766000	-1.319622000
1	2.016207000	4.253203000	0.633168000
6	2.902529000	4.178290000	-1.319855000
1	2.596613000	5.072224000	-1.871358000
1	3.049015000	3.394041000	-2.069895000
6	4.231425000	4.506544000	-0.631703000
1	4.102411000	5.338800000	0.073061000
1	4.620379000	3.676856000	-0.032044000
6	5.305997000	4.906904000	-1.617953000
8	5.153560000	5.042721000	-2.815851000
8	6.489468000	5.110239000	-1.000235000
1	3.696057000	1.880056000	-0.019826000
6	0.054283000	4.130399000	-2.223418000
1	-0.991901000	4.362983000	-2.442036000
1	0.667262000	4.842605000	-2.781966000
1	0.270022000	3.125486000	-2.603491000
6	-0.014298000	5.584482000	-0.093829000
7	-0.509114000	3.892698000	1.425350000
6	-0.304129000	5.243022000	1.368212000
8	-0.347425000	6.013694000	2.318513000
1	-2.500980000	2.508083000	1.645139000
1	-4.702926000	2.943390000	0.988247000
6	-4.787718000	2.050277000	-0.948922000
1	-5.882504000	2.051967000	-0.901424000
1	-4.511486000	2.988507000	-1.440323000
6	-4.334728000	0.879720000	-1.845174000
1	-3.273531000	0.972312000	-2.102164000
1	-4.441536000	-0.079493000	-1.342414000
6	-5.116138000	0.864132000	-3.151162000
8	-5.289723000	1.859555000	-3.827646000
8	-5.627694000	-0.315186000	-3.546720000
6	-5.937151000	0.107034000	1.010015000
1	-6.126519000	-0.728157000	1.691354000
1	-6.743652000	0.834311000	1.149024000
1	-6.004545000	-0.278002000	-0.008769000
6	-4.572164000	1.137815000	2.848692000

1	-5.463755000	1.738413000	3.054591000
1	-3.700985000	1.752188000	3.088437000
6	-4.481551000	-0.079492000	3.760513000
8	-3.438778000	-0.734920000	3.841496000
7	-5.598934000	-0.398410000	4.453453000
1	-5.579696000	-1.191885000	5.080925000
1	-6.444378000	0.151393000	4.407456000
1	-3.031969000	-1.968036000	1.841755000
1	-4.336331000	-1.898848000	0.663944000
1	-2.588985000	-1.484356000	-1.144576000
1	0.783942000	6.327821000	-0.169647000
1	-0.916131000	6.020186000	-0.541769000
1	-0.726966000	3.431330000	2.298892000
1	-3.278343000	-3.996899000	0.221916000
6	-2.978517000	-3.930721000	-1.909362000
1	-3.078944000	-5.017705000	-2.017455000
1	-2.194980000	-3.627402000	-2.619495000
6	-4.250917000	-3.268165000	-2.397945000
8	-4.844965000	-2.373264000	-1.811091000
8	-4.713614000	-3.661237000	-3.591205000
1	-0.697374000	-4.572226000	-1.089195000
6	-0.997514000	-5.197716000	0.930680000
6	1.506798000	-4.453462000	0.943230000
6	0.480181000	-5.594722000	1.008116000
1	-1.340990000	-4.775337000	1.883611000
1	-1.620044000	-6.073502000	0.717932000
1	0.725133000	-6.253199000	0.162333000
1	0.703781000	-6.167193000	1.913950000
8	2.676045000	-4.739746000	1.217932000
1	4.750785000	-0.461367000	-0.040059000
1	3.792344000	-2.781028000	1.359959000
6	3.911439000	-2.812846000	-0.796654000
1	3.764601000	-2.101279000	-1.618406000
1	3.278494000	-3.676496000	-1.008177000
6	5.368478000	-3.267649000	-0.752029000
1	6.042438000	-2.454199000	-0.463119000
1	5.501072000	-4.041104000	0.015157000
6	5.838585000	-3.831985000	-2.070826000
8	5.179653000	-3.909864000	-3.089918000
8	7.120356000	-4.256835000	-1.997830000
6	4.420678000	-0.338251000	2.082676000
1	4.541973000	0.748069000	2.181399000
1	3.678699000	-0.634323000	2.832289000
6	5.755120000	-0.962615000	2.429492000
8	6.558200000	-1.406828000	1.632200000
8	5.976168000	-0.939249000	3.758157000

1	7.135414000	5.374607000	-1.683783000
1	6.864184000	-1.315293000	3.917048000
1	7.361253000	-4.606310000	-2.877630000
1	-4.163939000	-4.367867000	-3.975838000
1	-5.437256000	-1.040703000	-2.905504000

Singlet

E-oxidized-state-singlet-H2O

Oxidized-state-singlet.gjf.log

28	-0.334283000	-0.023390000	0.295591000
7	1.549438000	-0.572855000	0.341284000
7	0.121903000	1.899427000	0.333119000
7	-2.249212000	0.300889000	0.744932000
7	-0.787184000	-1.781685000	-0.256003000
6	2.041387000	-1.844699000	0.383351000
6	3.551178000	-1.849858000	0.546185000
6	2.593591000	0.315326000	0.480145000
6	3.851273000	-0.390210000	0.924101000
6	-2.164163000	-2.062246000	-0.704602000
6	-0.051632000	-2.852417000	-0.330145000
6	-0.737807000	-4.013896000	-1.005362000
6	-2.147176000	-3.474295000	-1.340671000
6	1.283958000	-2.991509000	0.150780000
6	-3.146516000	-0.588686000	1.023963000
6	-2.767370000	1.645282000	1.106957000
6	-4.298231000	1.446201000	1.201705000
6	-4.352525000	-0.008626000	1.766589000
6	-3.082509000	-1.983173000	0.508171000
6	-0.804572000	3.052057000	0.460762000
6	1.325981000	2.385493000	0.097068000
6	-0.180422000	4.205525000	-0.386204000
6	1.338519000	3.869975000	-0.227160000
6	-2.247122000	2.689762000	0.137819000
6	2.527073000	1.656822000	0.263861000
1	-2.854098000	3.596494000	0.220920000
1	-2.319757000	2.326901000	-0.889511000
1	1.717835000	4.379725000	0.670606000
6	2.210430000	4.321456000	-1.415331000
1	1.894389000	5.337291000	-1.673728000
1	2.005450000	3.702531000	-2.294741000
6	3.720688000	4.371899000	-1.165194000
1	3.949675000	4.798331000	-0.179154000
1	4.185318000	3.382128000	-1.185629000
6	4.437856000	5.218883000	-2.196983000
8	3.909887000	5.900209000	-3.053200000
8	5.777074000	5.134673000	-2.048662000

1	3.450848000	2.214496000	0.321491000
6	-0.668806000	4.218676000	-1.835391000
1	-1.747069000	4.397022000	-1.876639000
1	-0.187370000	5.019202000	-2.402454000
1	-0.458490000	3.269212000	-2.339941000
6	-0.538519000	5.473295000	0.406635000
7	-0.704688000	3.627894000	1.810462000
6	-0.600704000	4.990468000	1.854600000
8	-0.567016000	5.671526000	2.870890000
1	-2.397131000	1.887633000	2.106729000
1	-4.710126000	2.150581000	1.932279000
6	-5.076303000	1.656542000	-0.114367000
1	-6.142698000	1.538204000	0.106940000
1	-4.958010000	2.696981000	-0.431553000
6	-4.723466000	0.746240000	-1.301616000
1	-3.686934000	0.893645000	-1.628265000
1	-4.801844000	-0.311159000	-1.046556000
6	-5.608048000	1.025327000	-2.510276000
8	-6.112229000	2.108922000	-2.733970000
8	-5.797531000	0.005405000	-3.363820000
6	-5.681426000	-0.752687000	1.576084000
1	-5.646443000	-1.740769000	2.045547000
1	-6.483698000	-0.185212000	2.058421000
1	-5.957140000	-0.891210000	0.529601000
6	-4.042772000	0.031119000	3.304378000
1	-4.920303000	0.447518000	3.808206000
1	-3.200285000	0.694841000	3.513160000
6	-3.644925000	-1.328436000	3.865680000
8	-2.578000000	-1.857640000	3.537880000
7	-4.518962000	-1.912460000	4.714771000
1	-4.283556000	-2.806579000	5.125861000
1	-5.378546000	-1.467882000	5.002511000
1	-2.750426000	-2.662449000	1.300436000
1	-4.088278000	-2.296787000	0.215142000
1	-2.452347000	-1.292699000	-1.422002000
1	0.185908000	6.284300000	0.294384000
1	-1.522156000	5.866409000	0.121301000
1	-0.771125000	3.079240000	2.657833000
1	-2.917108000	-4.099262000	-0.878792000
6	-2.387872000	-3.438006000	-2.854623000
1	-2.350389000	-4.452242000	-3.271483000
1	-1.587912000	-2.872229000	-3.353232000
6	-3.697238000	-2.792314000	-3.260876000
8	-4.482426000	-2.276168000	-2.476520000
8	-3.981266000	-2.758754000	-4.567317000
1	-0.185859000	-4.231540000	-1.930746000

6	-0.607797000	-5.240238000	-0.096366000
6	1.808210000	-4.358395000	0.346370000
6	0.885735000	-5.551900000	0.062166000
1	-1.068464000	-5.032072000	0.877518000
1	-1.133369000	-6.097020000	-0.530724000
1	1.264245000	-5.998157000	-0.868970000
1	1.068358000	-6.288362000	0.850712000
8	2.952703000	-4.597493000	0.736796000
1	4.728212000	0.002093000	0.403485000
1	3.837898000	-2.559181000	1.323124000
6	4.224393000	-2.266778000	-0.784134000
1	4.105050000	-1.458406000	-1.515792000
1	3.720291000	-3.143171000	-1.194999000
6	5.705191000	-2.590129000	-0.597077000
1	6.242911000	-1.775264000	-0.100823000
1	5.824024000	-3.459201000	0.062068000
6	6.397943000	-2.894293000	-1.903536000
8	5.886273000	-2.864092000	-3.006192000
8	7.697438000	-3.213471000	-1.710830000
6	4.043061000	-0.178702000	2.436647000
1	4.043595000	0.891960000	2.676316000
1	3.216734000	-0.620310000	3.005020000
6	5.350398000	-0.752311000	2.939826000
8	6.280285000	-1.111475000	2.244162000
8	5.377941000	-0.796580000	4.285446000
1	6.184031000	5.708054000	-2.726910000
1	6.251687000	-1.143296000	4.552720000
1	8.085804000	-3.395466000	-2.588498000
1	-3.285888000	-3.187630000	-5.098627000
1	-5.373287000	-0.830552000	-3.049940000

j) Optimized reduced (doublet) and oxidized (triplet and singlet) state of **E** in epsilon=4.

E-reduced-state-doublet-eps4
Reduced-state-doublet.gjf.log

Temperature 298.150 Kelvin. Pressure 1.00000 Atm.

Zero-point correction= 0.924258 (Hartree/Particle)
Thermal correction to Energy= 0.983147
Thermal correction to Enthalpy= 0.984091
Thermal correction to Gibbs Free Energy= 0.822719
Sum of electronic and zero-point Energies= -3106.377016
Sum of electronic and thermal Energies= -3106.318127
Sum of electronic and thermal Enthalpies= -3106.317183

Sum of electronic and thermal Free Energies= -3106.478555

28	0.361405000	0.067914000	-0.368578000
7	-1.549380000	-0.750134000	-0.414384000
7	-0.368227000	1.941033000	-0.171702000
7	2.347886000	0.755257000	-0.653367000
7	1.052523000	-1.802201000	-0.197448000
6	-1.900037000	-2.052751000	-0.542593000
6	-3.415628000	-2.229137000	-0.527954000
6	-2.679518000	0.024880000	-0.377855000
6	-3.919909000	-0.785279000	-0.700389000
6	2.476833000	-2.032664000	0.102361000
6	0.409722000	-2.935471000	-0.253029000
6	1.263116000	-4.144514000	0.080629000
6	2.677706000	-3.562720000	0.274632000
6	-0.986104000	-3.125085000	-0.565128000
6	3.393732000	0.058278000	-0.937637000
6	2.739599000	2.176615000	-0.519292000
6	4.254766000	2.127665000	-0.204421000
6	4.677581000	0.894337000	-1.067282000
6	3.347808000	-1.437307000	-1.013925000
6	0.430772000	3.178170000	-0.121646000
6	-1.638049000	2.266556000	-0.006452000
6	-0.430919000	4.231751000	0.652779000
6	-1.855718000	3.757773000	0.232252000
6	1.837785000	2.941879000	0.439517000
6	-2.732048000	1.372178000	-0.127842000
1	2.296826000	3.919388000	0.629406000
1	1.750184000	2.423862000	1.397086000
1	-2.064558000	4.192246000	-0.757304000
6	-3.010390000	4.168681000	1.165399000
1	-2.744885000	5.098241000	1.678685000
1	-3.156913000	3.419720000	1.951020000
6	-4.330926000	4.426620000	0.432679000
1	-4.201423000	5.209687000	-0.326888000
1	-4.696119000	3.551035000	-0.114000000
6	-5.429689000	4.880142000	1.368081000
8	-5.300071000	5.125252000	2.550244000
8	-6.611651000	4.995927000	0.720722000
1	-3.724302000	1.800690000	-0.063829000
6	-0.209111000	4.202493000	2.165168000
1	0.825277000	4.454936000	2.416497000
1	-0.850938000	4.929354000	2.670664000
1	-0.430123000	3.211324000	2.577245000
6	-0.072269000	5.564829000	-0.021035000
7	0.503704000	3.819976000	-1.445475000

6	0.263287000	5.164159000	-1.460250000
8	0.309120000	5.892465000	-2.443314000
1	2.623037000	2.643282000	-1.504432000
1	4.744104000	3.034224000	-0.579813000
6	4.593443000	2.036190000	1.296925000
1	5.682098000	2.020379000	1.414267000
1	4.263700000	2.964195000	1.776355000
6	4.006403000	0.858831000	2.087454000
1	2.920500000	0.783044000	1.958800000
1	4.408719000	-0.102804000	1.747372000
6	4.302528000	0.970606000	3.573434000
8	4.951909000	1.851297000	4.090155000
8	3.785079000	-0.016953000	4.349413000
6	5.971434000	0.195955000	-0.622584000
1	6.264785000	-0.578610000	-1.338035000
1	6.785961000	0.927697000	-0.577651000
1	5.886963000	-0.285405000	0.351922000
6	4.864093000	1.370258000	-2.548949000
1	5.759142000	2.000884000	-2.588123000
1	4.015641000	1.984384000	-2.862029000
6	4.940344000	0.224932000	-3.550726000
8	3.944602000	-0.433838000	-3.850734000
7	6.165152000	-0.028547000	-4.079816000
1	6.261788000	-0.778402000	-4.751586000
1	6.983127000	0.514267000	-3.846494000
1	2.955474000	-1.743814000	-1.989247000
1	4.357224000	-1.838518000	-0.924096000
1	2.707802000	-1.510282000	1.035380000
1	-0.876926000	6.305348000	-0.003135000
1	0.812263000	6.026824000	0.436048000
1	0.716149000	3.310065000	-2.292807000
1	3.348550000	-3.938728000	-0.505973000
6	3.252620000	-3.949205000	1.642680000
1	3.319376000	-5.042143000	1.730202000
1	2.566274000	-3.624157000	2.439140000
6	4.613001000	-3.352118000	1.962089000
8	5.185900000	-2.516997000	1.293761000
8	5.188888000	-3.782130000	3.107866000
1	0.895165000	-4.569790000	1.027353000
6	1.075248000	-5.205640000	-1.002676000
6	-1.429011000	-4.489995000	-0.895880000
6	-0.395210000	-5.624810000	-0.991361000
1	1.353691000	-4.783067000	-1.977241000
1	1.726575000	-6.069840000	-0.824611000
1	-0.584435000	-6.268981000	-0.120059000
1	-0.663129000	-6.218815000	-1.871192000

8	-2.603738000	-4.808189000	-1.120048000
1	-4.739329000	-0.549093000	-0.015023000
1	-3.729419000	-2.883718000	-1.341842000
6	-3.864584000	-2.859176000	0.810341000
1	-3.750277000	-2.119770000	1.612977000
1	-3.213328000	-3.699328000	1.058148000
6	-5.308405000	-3.353770000	0.755629000
1	-5.997095000	-2.569817000	0.420936000
1	-5.403058000	-4.158819000	0.015698000
6	-5.794998000	-3.880188000	2.083413000
8	-5.164798000	-3.896961000	3.122614000
8	-7.062587000	-4.350670000	1.996663000
6	-4.385580000	-0.476513000	-2.133498000
1	-4.524318000	0.605158000	-2.262860000
1	-3.629670000	-0.779485000	-2.866254000
6	-5.705265000	-1.132033000	-2.476848000
8	-6.537864000	-1.513373000	-1.677681000
8	-5.883573000	-1.218498000	-3.812180000
1	-7.269338000	5.301381000	1.374991000
1	-6.765334000	-1.612112000	-3.961312000
1	-7.308339000	-4.669799000	2.886188000
1	4.634093000	-4.449770000	3.547772000
1	3.277392000	-0.646892000	3.810081000

Triplet
E-oxidized-state-triplet-eps4
Oxidized-state-triplet.gjf.log

Temperature 298.150 Kelvin. Pressure 1.00000 Atm.

Zero-point correction= 0.926980 (Hartree/Particle)
Thermal correction to Energy= 0.985101
Thermal correction to Enthalpy= 0.986045
Thermal correction to Gibbs Free Energy= 0.826917
Sum of electronic and zero-point Energies= -3106.236734
Sum of electronic and thermal Energies= -3106.178614
Sum of electronic and thermal Enthalpies= -3106.177669
Sum of electronic and thermal Free Energies= -3106.336797

28	-0.340477000	0.056289000	0.360210000
7	1.569291000	-0.701765000	0.403834000
7	0.331404000	1.969549000	0.183028000
7	-2.324482000	0.660319000	0.658680000
7	-0.989453000	-1.813768000	0.100155000
6	1.941682000	-2.004347000	0.544329000
6	3.456473000	-2.144749000	0.537104000

6	2.684553000	0.097563000	0.354103000
6	3.933371000	-0.688413000	0.686572000
6	-2.393834000	-2.049081000	-0.272416000
6	-0.322795000	-2.928591000	0.171563000
6	-1.134569000	-4.141909000	-0.225746000
6	-2.537555000	-3.572461000	-0.541854000
6	1.053254000	-3.088811000	0.553764000
6	-3.331310000	-0.076972000	0.985907000
6	-2.722663000	2.088684000	0.683953000
6	-4.264786000	2.043643000	0.558282000
6	-4.570221000	0.742158000	1.375265000
6	-3.317252000	-1.567308000	0.851619000
6	-0.501195000	3.191643000	0.139846000
6	1.588660000	2.305842000	-0.035419000
6	0.309141000	4.222230000	-0.714704000
6	1.761481000	3.786854000	-0.338704000
6	-1.933104000	2.906416000	-0.330286000
6	2.705545000	1.438747000	0.080107000
1	-2.437251000	3.868343000	-0.472007000
1	-1.905997000	2.399950000	-1.298595000
1	2.002756000	4.252976000	0.628363000
6	2.872105000	4.176721000	-1.332918000
1	2.559587000	5.069911000	-1.881703000
1	3.011065000	3.394084000	-2.086124000
6	4.209652000	4.508604000	-0.663333000
1	4.087067000	5.333600000	0.051226000
1	4.617914000	3.677184000	-0.079070000
6	5.260321000	4.927869000	-1.669106000
8	5.075486000	5.084357000	-2.858570000
8	6.458241000	5.120645000	-1.076043000
1	3.684869000	1.890124000	-0.005668000
6	0.017844000	4.128889000	-2.212265000
1	-1.030317000	4.360649000	-2.422505000
1	0.624842000	4.843180000	-2.774363000
1	0.232535000	3.125773000	-2.598205000
6	-0.036737000	5.576014000	-0.076784000
7	-0.508222000	3.873647000	1.440148000
6	-0.308365000	5.229173000	1.388260000
8	-0.343559000	5.990609000	2.342072000
1	-2.484674000	2.481320000	1.677910000
1	-4.701357000	2.913636000	1.061386000
6	-4.810066000	2.035083000	-0.883343000
1	-5.904292000	2.034449000	-0.823486000
1	-4.543131000	2.975828000	-1.374873000
6	-4.367764000	0.872152000	-1.792272000
1	-3.299873000	0.945226000	-2.028109000

1	-4.505848000	-0.093337000	-1.308740000
6	-5.123230000	0.902127000	-3.115586000
8	-5.272675000	1.919819000	-3.759774000
8	-5.627801000	-0.263955000	-3.558226000
6	-5.923905000	0.074640000	1.087804000
1	-6.094642000	-0.766134000	1.767642000
1	-6.728650000	0.798885000	1.250948000
1	-6.015893000	-0.301558000	0.067551000
6	-4.522011000	1.088949000	2.903947000
1	-5.427908000	1.655023000	3.143265000
1	-3.667369000	1.732164000	3.127538000
6	-4.356036000	-0.136273000	3.794658000
8	-3.300195000	-0.773390000	3.799457000
7	-5.423249000	-0.484783000	4.551216000
1	-5.337821000	-1.268630000	5.185409000
1	-6.267396000	0.067540000	4.588621000
1	-3.022082000	-2.014373000	1.806798000
1	-4.331136000	-1.910503000	0.635119000
1	-2.602267000	-1.464218000	-1.171851000
1	0.757791000	6.322541000	-0.158694000
1	-0.944364000	6.011195000	-0.513395000
1	-0.713220000	3.410022000	2.315275000
1	-3.279775000	-3.999625000	0.140308000
6	-2.949723000	-3.898997000	-1.984028000
1	-3.033746000	-4.985219000	-2.115384000
1	-2.163773000	-3.569380000	-2.680037000
6	-4.231311000	-3.243525000	-2.463322000
8	-4.840308000	-2.377240000	-1.852249000
8	-4.681499000	-3.611127000	-3.669290000
1	-0.682392000	-4.561781000	-1.136178000
6	-1.002611000	-5.193851000	0.878326000
6	1.501486000	-4.453747000	0.912845000
6	0.473709000	-5.594526000	0.966587000
1	-1.353183000	-4.774097000	1.829950000
1	-1.625766000	-6.067541000	0.658278000
1	0.725255000	-6.252289000	0.122338000
1	0.689631000	-6.168679000	1.872938000
8	2.667695000	-4.736667000	1.190459000
1	4.746458000	-0.448638000	-0.004085000
1	3.779111000	-2.781452000	1.361446000
6	3.911233000	-2.789483000	-0.795057000
1	3.771703000	-2.068223000	-1.609801000
1	3.277982000	-3.648617000	-1.022835000
6	5.366178000	-3.250808000	-0.747709000
1	6.044276000	-2.445114000	-0.447212000
1	5.491196000	-4.034255000	0.010352000

6	5.831868000	-3.801837000	-2.074429000
8	5.169282000	-3.862787000	-3.091493000
8	7.111246000	-4.234182000	-2.008562000
6	4.391354000	-0.341695000	2.114823000
1	4.506824000	0.743985000	2.226899000
1	3.644880000	-0.650043000	2.855189000
6	5.726241000	-0.966876000	2.463103000
8	6.528153000	-1.408272000	1.664823000
8	5.943518000	-0.946449000	3.792334000
1	7.085358000	5.400623000	-1.770723000
1	6.830275000	-1.325196000	3.951378000
1	7.346367000	-4.574343000	-2.893357000
1	-4.119924000	-4.297454000	-4.072015000
1	-5.458699000	-1.005322000	-2.932843000

Singlet

E-oxidized-state-singlet-eps4

Oxidized-state-singlet.gjf.log

Temperature 298.150 Kelvin. Pressure 1.00000 Atm.

Zero-point correction= 0.929213 (Hartree/Particle)
Thermal correction to Energy= 0.986629
Thermal correction to Enthalpy= 0.987573
Thermal correction to Gibbs Free Energy= 0.831860
Sum of electronic and zero-point Energies= -3106.242341
Sum of electronic and thermal Energies= -3106.184926
Sum of electronic and thermal Enthalpies= -3106.183981
Sum of electronic and thermal Free Energies= -3106.339694

28	-0.333044000	-0.024021000	0.287584000
7	1.539717000	-0.582245000	0.340281000
7	0.136892000	1.891133000	0.309622000
7	-2.242096000	0.316925000	0.742855000
7	-0.800355000	-1.774483000	-0.255908000
6	2.023605000	-1.859357000	0.391773000
6	3.533270000	-1.875984000	0.545608000
6	2.595036000	0.296049000	0.463150000
6	3.847119000	-0.416211000	0.908882000
6	-2.180596000	-2.053740000	-0.699332000
6	-0.074864000	-2.855405000	-0.312754000
6	-0.769612000	-4.020045000	-0.973317000
6	-2.173008000	-3.473167000	-1.319207000
6	1.256557000	-3.000937000	0.170905000
6	-3.147132000	-0.564824000	1.025589000
6	-2.754331000	1.666534000	1.098844000

6	-4.286263000	1.477697000	1.189994000
6	-4.350855000	0.026811000	1.763152000
6	-3.094678000	-1.961465000	0.515147000
6	-0.779877000	3.052291000	0.450481000
6	1.344832000	2.372796000	0.074312000
6	-0.149973000	4.205833000	-0.391631000
6	1.366021000	3.859937000	-0.233834000
6	-2.226080000	2.704969000	0.128644000
6	2.540263000	1.636156000	0.233087000
1	-2.823011000	3.618357000	0.214021000
1	-2.305451000	2.344949000	-0.899212000
1	1.743573000	4.355647000	0.672828000
6	2.245216000	4.324230000	-1.411564000
1	1.929333000	5.341572000	-1.663751000
1	2.048116000	3.714329000	-2.299234000
6	3.753876000	4.378251000	-1.150649000
1	3.973346000	4.790372000	-0.156202000
1	4.226245000	3.392428000	-1.184537000
6	4.469503000	5.250453000	-2.164247000
8	3.937736000	5.957769000	-2.994836000
8	5.809161000	5.153984000	-2.026375000
1	3.469605000	2.185771000	0.275567000
6	-0.636077000	4.226690000	-1.841600000
1	-1.712702000	4.414636000	-1.885526000
1	-0.146839000	5.023463000	-2.407032000
1	-0.432497000	3.276408000	-2.347800000
6	-0.500480000	5.471871000	0.407027000
7	-0.670711000	3.618092000	1.801622000
6	-0.559761000	4.983686000	1.854432000
8	-0.518963000	5.654834000	2.873288000
1	-2.383513000	1.911445000	2.098139000
1	-4.696152000	2.188968000	1.915488000
6	-5.055414000	1.685787000	-0.131030000
1	-6.124452000	1.577819000	0.083257000
1	-4.928809000	2.722827000	-0.455685000
6	-4.703169000	0.766046000	-1.310812000
1	-3.656491000	0.886130000	-1.615743000
1	-4.814894000	-0.288685000	-1.056633000
6	-5.555307000	1.074934000	-2.537768000
8	-6.011264000	2.175877000	-2.768070000
8	-5.762291000	0.059508000	-3.393734000
6	-5.684104000	-0.710205000	1.571519000
1	-5.657059000	-1.696746000	2.045051000
1	-6.485279000	-0.136041000	2.047817000
1	-5.957521000	-0.850398000	0.524631000
6	-4.047232000	0.072366000	3.301418000

1	-4.923261000	0.498775000	3.800184000
1	-3.200535000	0.730949000	3.510336000
6	-3.659954000	-1.287567000	3.870251000
8	-2.610980000	-1.837439000	3.524901000
7	-4.525814000	-1.848690000	4.745007000
1	-4.284069000	-2.732875000	5.173650000
1	-5.362026000	-1.379530000	5.060645000
1	-2.763762000	-2.638035000	1.310222000
1	-4.103461000	-2.269098000	0.226216000
1	-2.468902000	-1.290979000	-1.423770000
1	0.226457000	6.280737000	0.296345000
1	-1.483238000	5.870614000	0.126093000
1	-0.733158000	3.067048000	2.647409000
1	-2.949183000	-4.085982000	-0.850981000
6	-2.414496000	-3.455313000	-2.833743000
1	-2.376360000	-4.475075000	-3.237622000
1	-1.614326000	-2.895042000	-3.339099000
6	-3.726120000	-2.812800000	-3.245207000
8	-4.494156000	-2.265098000	-2.467566000
8	-4.025421000	-2.819066000	-4.548982000
1	-0.217445000	-4.256470000	-1.894345000
6	-0.653502000	-5.234963000	-0.046343000
6	1.772203000	-4.372104000	0.380142000
6	0.836285000	-5.561138000	0.120286000
1	-1.113899000	-5.006732000	0.923352000
1	-1.188494000	-6.092510000	-0.468001000
1	1.211373000	-6.032991000	-0.799415000
1	1.009873000	-6.281835000	0.925047000
8	2.915665000	-4.613129000	0.764644000
1	4.726663000	-0.037804000	0.382048000
1	3.820700000	-2.581629000	1.325633000
6	4.193207000	-2.309144000	-0.786651000
1	4.082271000	-1.503153000	-1.522359000
1	3.673269000	-3.178998000	-1.191440000
6	5.669664000	-2.657106000	-0.608674000
1	6.225934000	-1.852134000	-0.116607000
1	5.777934000	-3.528338000	0.049118000
6	6.342037000	-2.971172000	-1.924059000
8	5.819712000	-2.917124000	-3.020091000
8	7.633624000	-3.327942000	-1.745942000
6	4.047365000	-0.193100000	2.418963000
1	4.053878000	0.879082000	2.651790000
1	3.223037000	-0.627865000	2.995742000
6	5.356535000	-0.770443000	2.915727000
8	6.273045000	-1.146821000	2.213287000
8	5.398790000	-0.794514000	4.261506000

1	6.213752000	5.746635000	-2.689204000
1	6.272386000	-1.146140000	4.522525000
1	8.006281000	-3.513352000	-2.629483000
1	-3.344104000	-3.275140000	-5.074994000
1	-5.376829000	-0.787831000	-3.068894000

ix) Single point structure of reactants **A-E** without presence of Ni in **Figure 3B**.

A

A-noNi.gjf.log

6	0.719791069	-1.973467568	-6.313874378
6	0.852310610	-2.210525343	-4.827339606
6	0.889837283	-3.501493476	-4.277037062
6	0.963059502	-1.126803348	-3.944595801
6	1.037275744	-3.701674815	-2.901829071
6	1.104556498	-1.305727193	-2.573784824
6	1.148610959	-2.601370585	-2.043690764
8	1.274524177	-2.844891292	-0.707689717
6	1.611874768	5.366780507	-1.174755406
6	0.049756204	2.601313418	4.655911488
6	3.855769595	-6.557794820	1.602102119
6	4.297297528	-0.858697853	-4.697801900
7	1.509855904	2.104828030	-0.799300110
6	1.550975606	6.634664378	-2.031057536
6	0.805624879	1.768806388	5.707506996
7	0.323625467	1.327043585	1.745823696
8	4.750500461	-6.872317827	2.364006823
8	3.666620310	-0.191216500	-5.496381413
7	3.409595327	-0.074288725	-0.573595789
8	2.239239977	8.902227414	-2.127642904
8	1.907849552	2.860010950	7.587467541
8	2.174356856	7.966273571	-0.083415904
8	2.818051258	3.014988858	5.533628242
6	3.161997319	1.468020463	-2.478678146
6	-0.219083123	3.306940815	0.440903198
6	0.262313548	-0.476824548	3.396497031
6	4.038288628	-2.036200001	0.767318129
6	2.128608875	2.239854347	-2.025769854
6	-0.376669867	2.461604062	1.529881437
6	1.254302600	-1.336646157	2.853686663
6	4.207102462	-1.178894133	-0.316616983
6	1.430046343	3.327090233	-2.858577287
6	-1.466601442	2.673184367	2.593514488
6	1.643341341	-2.619995973	3.395498617
6	5.118001159	-1.404788655	-1.412467821

6	0.789646885	4.177367511	-1.722321474
6	-0.892923064	1.782884487	3.735428714
6	2.761184675	-3.026026283	2.693030969
6	4.847756302	-0.421881010	-2.341282951
6	0.662059715	3.131632885	-0.625065452
6	-0.079618195	0.771607592	2.941541628
6	3.026716003	-1.994673557	1.724313821
6	3.784044636	0.389844372	-1.795203374
6	0.304155179	2.598657890	-3.641514824
6	0.870179121	-3.438933872	4.397882100
6	6.121480770	-2.517244330	-1.498120890
6	-0.708418876	3.476633147	-4.342242441
6	-3.918578601	1.950713025	2.968747270
6	-0.098511141	-4.314086539	3.610682137
6	7.349228874	-2.257799869	-0.611111387
8	-0.672639969	4.684074254	-4.482914915
8	-4.510148139	2.899599340	3.447940167
8	0.144940262	-5.457867682	3.259420412
6	-2.757194763	2.027197923	2.004220725
6	3.491861503	-4.334381587	2.773393128
8	-1.207010992	-3.651160799	3.283673574
6	2.361214169	4.056374250	-3.832351115
6	-1.722327454	4.143886144	2.950140536
6	3.036063088	-5.294995814	1.661047294
6	5.364737725	-0.327276533	-3.753014957
6	2.019599176	7.863173068	-1.284778584
6	1.861223273	2.586092523	6.406730062
7	2.073766458	-0.989234752	1.819792642
8	3.478838578	-7.350043490	0.568322803
8	4.084593278	-2.176469941	-4.505193838
6	-9.989473296	-2.987029937	-1.765697958
6	-9.922045108	-2.055801208	-2.981223031
6	-9.499052883	-0.616273146	-2.641623759
6	-8.117912011	-0.431868491	-1.991755425
6	-6.911015660	-0.758101301	-2.885135482
16	-6.395247339	-2.535037238	-2.963602387
6	-1.498239452	-1.270958866	0.184130216
8	-4.068826347	-3.063661846	-0.487317498
16	-1.929423918	-0.129957799	-1.197932506
6	-2.589096493	-1.606935026	1.206267995
8	-4.565098179	-3.173391405	1.962355798
16	-3.520603738	-3.138223687	0.904211134
8	-2.502976251	-4.246667786	1.043198078
6	-8.353279309	-0.421384549	2.688392201
6	-7.339602770	0.202252807	1.759039760
6	-6.430475674	-0.625413259	1.083753704

6	-7.285825820	1.578151466	1.504388164
6	-5.511287860	-0.106012642	0.179209897
6	-6.349030162	2.118223184	0.617729124
6	-5.452547281	1.274649168	-0.045434398
8	-4.535157087	1.830169385	-0.898188155
6	4.555427883	6.203404499	1.936784350
6	4.235895536	4.931694175	2.722801195
6	3.910162756	3.742535935	1.814185761
7	3.429715599	2.644285733	2.453845504
8	4.124371902	3.772693694	0.600773915
1	0.225542511	-2.815667521	-6.811309988
1	0.133286993	-1.070570925	-6.517719116
1	0.796858507	-4.366209493	-4.930876660
1	0.947749134	-0.118403303	-4.341115717
1	1.064558424	-4.702281682	-2.480651295
1	1.181936484	-0.451521569	-1.913587600
1	1.463183333	-2.014175469	-0.226988832
1	-7.869619717	-1.115495946	3.387273847
1	-9.087318534	-1.012854626	2.123634577
1	-6.418082713	-1.694658860	1.273421488
1	-7.975007536	2.246011573	2.016973573
1	-4.842037393	-0.776267083	-0.347818480
1	-6.288970555	3.189479933	0.450086386
1	-3.796572605	1.189343012	-1.023357514
1	1.206691591	5.616514492	-0.188226320
1	2.647737550	5.058345441	-0.996647531
1	-0.528152675	3.376537439	5.170790680
1	0.781650329	3.113673790	4.021916223
1	2.133216564	6.558461213	-2.952146456
1	0.518266394	6.832537827	-2.352703221
1	0.126231371	1.372468303	6.466656105
1	1.305023837	0.926205683	5.216531079
1	3.524034987	1.666450178	-3.481022905
1	-0.848702711	4.189888618	0.399949152
1	-0.224473941	-0.809060747	4.308571925
1	-0.194045651	4.556786736	-2.015425899
1	-1.676004002	1.309089892	4.338383443
1	-0.250772892	1.923501953	-2.977705405
1	0.751835519	1.957395370	-4.410302748
1	1.532934615	-4.091777521	4.972415661
1	0.312412199	-2.803353524	5.092604276
1	6.450877944	-2.660429787	-2.531974132
1	5.665752318	-3.469053852	-1.198998693
1	7.846187061	-1.323144125	-0.901768516
1	-3.088659338	2.611931089	1.140545573
1	-2.521653207	1.025101360	1.649728595

1	3.323686099	-4.814012643	3.743658426
1	4.574751642	-4.180503156	2.691819164
1	3.214213128	4.498998782	-3.309526618
1	1.820315358	4.837697537	-4.370705033
1	2.757400037	3.357047322	-4.577261916
1	-2.394294579	4.224897180	3.808239794
1	-0.791675253	4.673187056	3.171725878
1	-2.206911920	4.659648517	2.114781685
1	1.987572089	-5.573300555	1.817517609
1	3.073930906	-4.806079572	0.680373079
1	6.272744393	-0.928018830	-3.871057753
1	5.593193597	0.699225262	-4.052152913
1	-8.989370894	-3.172159867	-1.360102452
1	-10.424335072	-3.957345957	-2.033611308
1	-9.235287076	-2.481832259	-3.723724208
1	-10.909171290	-2.015037981	-3.463448092
1	-9.538498443	-0.008944777	-3.558312862
1	-10.250729480	-0.188991476	-1.961217733
1	-8.019263741	0.622070225	-1.696814898
1	-8.047996742	-0.998633882	-1.058890602
1	-6.037367623	-0.185683682	-2.564750466
1	-7.115351634	-0.476688342	-3.924547281
1	-5.683642107	-2.585794353	-1.813044542
1	-0.686686317	-0.750610998	0.699176165
1	-1.063413152	-2.176119985	-0.246917759
1	-2.124584979	-1.751403786	2.184962563
1	-3.334496519	-0.820122282	1.307998464
1	5.287807116	5.986436430	1.153747280
1	3.665599765	6.616528256	1.452882411
1	5.094745719	4.639356554	3.343825252
1	3.402860091	5.092144310	3.419820751
1	-8.922289105	0.302075340	3.287267433
1	1.695167659	-1.832040307	-6.797171747
1	4.979831715	6.959594608	2.605893574
6	-2.441314301	-1.265628570	-2.531759133
1	-2.696582008	-0.641222493	-3.388709733
1	-1.606077605	-1.911880067	-2.798575683
1	-3.295911380	-1.857151159	-2.221575385
1	2.510353172	9.659924143	-1.574447125
1	3.467750944	3.536974366	6.041469276
1	4.045768464	-8.144131262	0.601678501
1	-1.695861902	-4.098804862	2.525317437
1	3.243701593	-2.416665732	-4.943599753
1	-2.346267325	3.339285081	-5.274530869
1	3.138823409	2.694106003	3.422959381
1	3.034672378	1.890658123	1.885229881

1	-5.058906152	0.687522677	3.795059513
1	-10.599213707	-2.552895102	-0.963364599
1	4.715080014	-2.883931693	0.822525535
1	7.054921973	-2.120251441	0.436184309
6	8.371464273	-3.367906082	-0.674042876
8	8.317769769	-4.351914918	-1.384508001
8	9.403632488	-3.143358797	0.175994514
1	10.017828412	-3.896171558	0.078460458
8	-4.242397458	0.671508022	3.259676931
8	-1.725316406	2.727042852	-4.834838842

B

B-noNi.gjf.log

6	1.568962000	-3.133132000	-6.527867000
6	1.626370000	-2.975213000	-5.025844000
6	1.875376000	-4.072995000	-4.187122000
6	1.494031000	-1.713312000	-4.427760000
6	1.988255000	-3.921964000	-2.805504000
6	1.616370000	-1.541951000	-3.049939000
6	1.858584000	-2.651008000	-2.230919000
8	1.959519000	-2.561165000	-0.876876000
6	0.190389000	5.262964000	-1.084470000
6	-0.602297000	2.221616000	5.125923000
6	4.402740000	-5.930690000	1.148142000
6	4.422909000	0.057861000	-4.571064000
7	0.834947000	2.091325000	-0.627269000
6	-0.266947000	6.468314000	-1.907315000
6	0.073457000	1.392880000	6.234258000
7	-0.058193000	1.179325000	2.011954000
8	5.396003000	-6.274185000	1.760689000
8	3.612358000	0.585797000	-5.309206000
7	3.175358000	0.393694000	-0.431438000
8	-0.251407000	8.836387000	-2.016712000
8	0.349796000	2.310424000	8.470381000
8	1.260954000	7.888280000	-0.647660000
8	1.666242000	3.054544000	6.802004000
6	2.575150000	1.845657000	-2.327164000
6	-1.080307000	2.914992000	0.656273000
6	0.334827000	-0.561998000	3.675485000
6	4.230727000	-1.383079000	0.896573000
6	1.381892000	2.338138000	-1.871125000
6	-0.991334000	2.130999000	1.803135000
6	1.447367000	-1.213821000	3.083121000
6	4.224699000	-0.476687000	-0.163879000
6	0.442667000	3.212273000	-2.713656000

6	-2.020132000	2.214672000	2.946851000
6	2.025285000	-2.465428000	3.529461000
6	5.183234000	-0.444422000	-1.236249000
6	-0.371306000	3.907703000	-1.579418000
6	-1.303869000	1.362292000	4.045034000
6	3.149811000	-2.678158000	2.760824000
6	4.698311000	0.463516000	-2.159876000
6	-0.231595000	2.893194000	-0.449293000
6	-0.284995000	0.579393000	3.230081000
6	3.239185000	-1.556217000	1.856344000
6	3.446328000	0.955200000	-1.645735000
6	-0.485766000	2.223319000	-3.499515000
6	1.407867000	-3.418663000	4.520252000
6	6.438569000	-1.263189000	-1.309067000
7	-4.633827000	-0.108638000	3.758872000
6	-1.581088000	2.907074000	-4.312896000
6	-4.332139000	1.201682000	3.564312000
6	0.352402000	-4.250405000	3.800100000
6	7.532097000	-0.736097000	-0.367184000
8	-1.338859000	3.517541000	-5.358409000
8	-4.835221000	2.095450000	4.252585000
8	0.610760000	-5.228765000	3.117675000
6	-3.317025000	1.494936000	2.464673000
6	3.993985000	-3.914296000	2.656363000
7	-2.835325000	2.814739000	-3.802287000
8	-0.879603000	-3.756140000	3.951130000
6	1.179912000	4.129671000	-3.696950000
6	-2.345118000	3.666645000	3.336430000
6	3.495351000	-4.778849000	1.482980000
6	5.256420000	0.776557000	-3.524819000
6	0.342056000	7.766996000	-1.434492000
6	0.678421000	2.271359000	7.300264000
7	2.165152000	-0.700021000	2.046686000
8	3.981823000	-6.584351000	0.034789000
8	4.636764000	-1.274587000	-4.543913000
6	-9.446310000	-3.329546000	-2.651448000
6	-9.006305000	-2.485079000	-3.852651000
6	-8.460601000	-1.096008000	-3.482280000
6	-7.208896000	-1.059147000	-2.590732000
6	-5.938656000	-1.659598000	-3.218841000
16	-5.637511000	-3.455840000	-2.889018000
6	-0.880897000	-2.017707000	0.491700000
8	-3.773977000	-3.435491000	0.100120000
16	-1.085246000	-1.082106000	-1.077373000
6	-2.036149000	-1.923645000	1.485393000
8	-4.302317000	-2.759742000	2.451092000

16	-3.264141000	-3.253054000	1.492685000
8	-2.538721000	-4.477027000	1.987964000
6	-8.077689000	-0.087563000	1.436867000
6	-6.820916000	0.324946000	0.713638000
6	-5.842559000	-0.649411000	0.472551000
6	-6.604073000	1.610720000	0.204796000
6	-4.696961000	-0.362622000	-0.258093000
6	-5.440615000	1.924536000	-0.509155000
6	-4.479982000	0.934630000	-0.730117000
8	-3.322489000	1.263118000	-1.390838000
6	4.968022000	6.246248000	0.551712000
6	3.569252000	5.918075000	1.132126000
6	3.198023000	4.444987000	1.274011000
7	3.635611000	3.601771000	0.302032000
8	2.542846000	4.049547000	2.241492000
1	1.144829000	-4.100577000	-6.819297000
1	0.971364000	-2.342651000	-6.994150000
1	1.984600000	-5.064079000	-4.622726000
1	1.323584000	-0.838351000	-5.049498000
1	2.186461000	-4.772631000	-2.159048000
1	1.518521000	-0.558186000	-2.609668000
1	1.945449000	-1.623020000	-0.598045000
1	-7.836648000	-0.775246000	2.256735000
1	-8.746477000	-0.636865000	0.758245000
1	-5.963390000	-1.655311000	0.862052000
1	-7.346011000	2.387812000	0.374999000
1	-3.980661000	-1.148818000	-0.456997000
1	-5.259387000	2.932060000	-0.872733000
1	-2.645451000	0.561969000	-1.227851000
1	-0.134942000	5.413041000	-0.049138000
1	1.283793000	5.216753000	-1.048171000
1	-1.346296000	2.877629000	5.591163000
1	0.148792000	2.864090000	4.653586000
1	-0.008844000	6.366200000	-2.966819000
1	-1.358701000	6.570294000	-1.879439000
1	-0.646543000	0.728199000	6.720265000
1	0.871560000	0.781317000	5.800930000
1	2.871569000	2.120139000	-3.332558000
1	-1.896560000	3.628686000	0.609662000
1	-0.059127000	-1.005297000	4.584433000
1	-1.422993000	4.042183000	-1.857132000
1	-2.006784000	0.690166000	4.551064000
1	-0.912839000	1.507141000	-2.790097000
1	0.138311000	1.660895000	-4.201795000
1	2.163957000	-4.104678000	4.912998000
1	0.949188000	-2.887248000	5.359897000

1	6.825497000	-1.284204000	-2.332718000
1	6.227670000	-2.308692000	-1.051811000
1	7.785026000	0.304189000	-0.609726000
1	-3.815182000	2.121528000	1.718987000
1	-3.039909000	0.572771000	1.958778000
1	3.954492000	-4.501010000	3.580790000
1	5.049325000	-3.661574000	2.497605000
1	1.897432000	4.775526000	-3.181731000
1	0.479351000	4.741451000	-4.268063000
1	1.734550000	3.532397000	-4.428021000
1	-3.021113000	3.691269000	4.193307000
1	-1.439639000	4.236959000	3.562649000
1	-2.856655000	4.171206000	2.509885000
1	2.501351000	-5.177102000	1.720651000
1	3.362009000	-4.167086000	0.582957000
1	6.295543000	0.441070000	-3.600479000
1	5.222286000	1.846237000	-3.749749000
1	-8.577943000	-3.653370000	-2.067996000
1	-9.983799000	-4.228800000	-2.975120000
1	-8.249869000	-3.040926000	-4.423505000
1	-9.858593000	-2.344727000	-4.532334000
1	-8.243811000	-0.543879000	-4.408885000
1	-9.257567000	-0.534593000	-2.972452000
1	-6.990855000	-0.010590000	-2.349573000
1	-7.407302000	-1.541211000	-1.628594000
1	-5.051822000	-1.123694000	-2.874021000
1	-5.965248000	-1.570735000	-4.310640000
1	-5.127077000	-3.323881000	-1.643209000
1	-0.003235000	-1.563187000	0.953474000
1	-0.619514000	-3.050215000	0.244569000
1	-1.645941000	-1.902722000	2.505183000
1	-2.602193000	-1.010058000	1.326180000
1	5.666828000	5.414308000	0.688891000
1	4.938600000	6.499751000	-0.512495000
1	3.474814000	6.325722000	2.142717000
1	2.786049000	6.403442000	0.535396000
1	-8.673788000	0.726888000	1.869485000
1	2.577904000	-3.078002000	-6.956765000
1	5.358219000	7.098095000	1.118914000
6	-1.740661000	-2.359125000	-2.203840000
1	-1.978722000	-1.862534000	-3.144641000
1	-0.971868000	-3.110402000	-2.385204000
1	-2.631742000	-2.817312000	-1.785372000
1	0.207569000	9.631135000	-1.682846000
1	1.996257000	3.600373000	7.540858000
1	4.610415000	-7.315593000	-0.117363000

1	-1.501339000	-4.186290000	3.287318000
1	3.918748000	-1.699269000	-5.054470000
1	-3.589195000	3.247468000	-4.319082000
1	-3.055323000	2.281915000	-2.962806000
1	3.961652000	3.956788000	-0.585596000
1	3.256009000	2.658110000	0.294228000
1	-5.357479000	-0.312611000	4.436170000
1	-10.102399000	-2.767458000	-1.974987000
1	5.052356000	-2.093440000	0.917884000
1	7.178694000	-0.718392000	0.670700000
6	8.803339000	-1.549915000	-0.422122000
8	9.017588000	-2.494390000	-1.155652000
8	9.723473000	-1.097417000	0.464690000
1	10.510328000	-1.667963000	0.370511000
1	-4.364847000	-0.873324000	3.140370000

C

C-noNi.gjf.log

6	-1.336244000	7.548418000	-0.881778000
6	-0.905451000	6.195267000	-1.397703000
6	0.001508000	6.083175000	-2.463015000
6	-1.370045000	5.009614000	-0.816920000
6	0.431919000	4.842059000	-2.928668000
6	-0.940361000	3.757164000	-1.260061000
6	-0.035488000	3.667777000	-2.324700000
8	0.415256000	2.477345000	-2.816894000
6	0.239613000	-2.149072000	4.299602000
6	3.760305000	-4.535105000	-0.490200000
6	7.765878000	0.346416000	-1.652204000
6	0.792317000	5.852327000	3.580118000
7	-0.706618000	-0.607439000	1.684572000
6	0.247814000	-2.477377000	5.798884000
6	4.770341000	-4.264340000	-1.621530000
7	1.157925000	-2.168078000	-0.027534000
8	7.701548000	-0.485736000	-0.754727000
8	1.798057000	6.353088000	4.051058000
7	1.050055000	1.759068000	1.020446000
8	1.957098000	-2.746032000	7.435270000
8	5.840370000	-2.379248000	-2.691424000
8	2.189853000	-3.916063000	5.527386000
8	5.761000000	-2.422732000	-0.441773000
6	-1.052593000	1.713690000	2.322992000
6	-0.356101000	-3.132595000	1.641027000
6	2.630612000	-1.945865000	-1.953168000
6	2.764812000	2.610887000	-0.483735000

6	-1.109511000	0.239028000	2.567285000
6	0.632266000	-3.225716000	0.741745000
6	2.766644000	-0.584579000	-2.013277000
6	1.856589000	2.710232000	0.601942000
6	-1.649697000	-0.368932000	3.865983000
6	1.308275000	-4.565310000	0.404923000
6	3.583796000	0.116906000	-3.079457000
6	1.791502000	3.987361000	1.437363000
6	-1.146104000	-1.839237000	3.703547000
6	2.282486000	-4.154484000	-0.751202000
6	4.055202000	1.360714000	-2.295428000
6	0.502148000	3.744016000	2.253203000
6	-1.113722000	-1.956924000	2.147904000
6	2.011902000	-2.672831000	-0.910665000
6	2.956412000	1.488938000	-1.245927000
6	0.392656000	2.210798000	2.258110000
6	-3.211589000	-0.281462000	3.802662000
6	2.721701000	0.470193000	-4.299542000
6	1.778156000	5.301401000	0.610955000
7	0.400481000	-7.049447000	-2.046186000
6	-3.898078000	-1.100783000	4.886423000
6	0.900312000	-6.790558000	-0.816151000
6	3.539212000	1.080879000	-5.413232000
6	2.865305000	6.333785000	0.994761000
8	-3.904649000	-0.771861000	6.071121000
8	1.790597000	-7.474668000	-0.295783000
8	4.738804000	1.280806000	-5.393863000
6	0.263525000	-5.588537000	-0.116572000
6	5.382653000	1.109017000	-1.547521000
7	-4.520875000	-2.235494000	4.449949000
8	2.768111000	1.396259000	-6.478829000
6	-1.136064000	0.380963000	5.106693000
6	1.989674000	-5.103598000	1.682184000
6	6.595657000	0.853207000	-2.452457000
6	0.455034000	4.380696000	3.655837000
6	1.553458000	-3.129135000	6.201659000
6	5.471097000	-2.919824000	-1.662287000
7	2.300823000	0.301638000	-1.084002000
8	8.938481000	0.884226000	-2.020184000
8	-0.096453000	6.548297000	2.842941000
6	-10.962871000	1.264254000	-2.940172000
6	-10.710114000	2.085552000	-1.666155000
6	-10.173692000	1.254138000	-0.487248000
6	-8.906850000	0.417684000	-0.729890000
6	-7.575377000	1.171347000	-0.825766000
16	-7.314884000	1.994242000	-2.471531000

6	-0.845205000	-1.209013000	-2.755861000
8	-1.423549000	-3.411738000	-4.893160000
16	-1.579487000	0.171819000	-1.780781000
6	-1.418627000	-2.572602000	-2.397817000
8	-1.466905000	-5.113689000	-3.059179000
16	-0.860534000	-3.839874000	-3.582781000
8	0.628802000	-3.842532000	-3.530776000
6	-8.355677000	-3.228026000	-1.486190000
6	-7.094184000	-2.619884000	-0.932089000
6	-6.072771000	-2.214496000	-1.799379000
6	-6.930539000	-2.352445000	0.434857000
6	-4.932929000	-1.560527000	-1.333440000
6	-5.798402000	-1.697098000	0.918841000
6	-4.788093000	-1.296579000	0.033251000
8	-3.701622000	-0.651181000	0.550095000
6	4.443071000	-1.394279000	5.140954000
6	4.236446000	-1.877050000	3.704961000
6	3.560836000	-0.814039000	2.846750000
7	3.875227000	-0.822867000	1.525535000
8	2.785345000	0.013041000	3.339913000
1	-1.930964000	8.095679000	-1.625004000
1	-1.943236000	7.451345000	0.024475000
1	0.388510000	6.986160000	-2.930800000
1	-2.065312000	5.060654000	0.018079000
1	1.138585000	4.763047000	-3.749672000
1	-1.284854000	2.853250000	-0.769751000
1	0.103406000	1.740851000	-2.248041000
1	-8.202197000	-3.569155000	-2.515956000
1	-9.160280000	-2.480757000	-1.518499000
1	-6.168310000	-2.403694000	-2.865699000
1	-7.708248000	-2.649472000	1.134943000
1	-4.161824000	-1.250459000	-2.026983000
1	-5.698932000	-1.476102000	1.977062000
1	-3.019598000	-0.500164000	-0.144628000
1	0.633921000	-3.028808000	3.784557000
1	0.947504000	-1.341591000	4.084167000
1	3.760816000	-5.614013000	-0.310261000
1	4.123822000	-4.062221000	0.426152000
1	0.072791000	-1.609671000	6.436191000
1	-0.547044000	-3.202760000	6.026135000
1	5.594428000	-4.984963000	-1.514410000
1	4.338123000	-4.441645000	-2.610021000
1	-1.541042000	1.940746000	1.368530000
1	-1.593626000	2.249637000	3.108401000
1	-0.682553000	-4.063585000	2.100554000
1	3.071987000	-2.522107000	-2.754110000

1	4.407523000	-0.519026000	-3.406683000
1	2.659654000	3.984352000	2.115295000
1	-1.867062000	-2.554983000	4.113819000
1	1.978826000	-4.633473000	-1.689187000
1	4.140159000	2.252792000	-2.922976000
1	-0.336119000	4.140807000	1.668940000
1	-2.151308000	-2.068504000	1.795217000
1	0.959197000	1.787895000	3.100535000
1	-3.555973000	-0.576778000	2.810119000
1	-3.506449000	0.763019000	3.950185000
1	2.222744000	-0.423496000	-4.695019000
1	1.925585000	1.174466000	-4.030037000
1	0.802042000	5.767391000	0.732429000
1	1.858712000	5.081363000	-0.456088000
1	3.047642000	6.304833000	2.073132000
1	-0.326594000	-5.969753000	0.726449000
1	-0.431058000	-5.096253000	-0.801235000
1	5.242357000	0.244636000	-0.890487000
1	5.587871000	1.964144000	-0.893180000
1	-0.043335000	0.404842000	5.139844000
1	-1.517751000	-0.087121000	6.016075000
1	-1.493129000	1.416151000	5.108713000
1	2.501652000	-6.046853000	1.488197000
1	2.696414000	-4.377122000	2.095832000
1	1.230758000	-5.286642000	2.450133000
1	6.367971000	0.061174000	-3.174111000
1	6.881724000	1.743928000	-3.016690000
1	1.180848000	3.907874000	4.323004000
1	-0.546779000	4.252009000	4.082796000
1	-10.029600000	1.085313000	-3.486236000
1	-11.650117000	1.778687000	-3.620698000
1	-10.023846000	2.911365000	-1.894020000
1	-11.648590000	2.553730000	-1.339995000
1	-10.000805000	1.916218000	0.373302000
1	-10.968346000	0.558121000	-0.181913000
1	-8.794227000	-0.270603000	0.117225000
1	-9.028491000	-0.217904000	-1.614563000
1	-6.756561000	0.461269000	-0.700479000
1	-7.504481000	1.928203000	-0.037960000
1	-5.967393000	2.054478000	-2.407538000
1	0.226551000	-1.193239000	-2.561291000
1	-1.014980000	-0.983158000	-3.812232000
1	-1.103471000	-2.876738000	-1.396920000
1	-2.509394000	-2.592730000	-2.445383000
1	4.989377000	-0.444029000	5.154344000
1	3.487538000	-1.228970000	5.642935000

1	5.186910000	-2.178407000	3.248363000
1	3.587820000	-2.763404000	3.704562000
1	-8.758803000	-4.080755000	-0.924129000
1	-0.470962000	8.175255000	-0.630227000
1	5.027414000	-2.117607000	5.719830000
6	-2.656167000	0.950897000	-3.041377000
1	-3.187858000	1.762889000	-2.545819000
1	-2.041600000	1.363120000	-3.840392000
1	-3.376485000	0.244643000	-3.452293000
1	2.793118000	-3.217247000	7.619041000
1	6.373927000	-1.648983000	-0.564062000
1	9.635856000	0.469595000	-1.474515000
1	3.359345000	1.776612000	-7.156638000
1	0.352159000	7.369892000	2.524588000
1	-4.901205000	-2.869556000	5.140638000
1	-4.406264000	-2.582350000	3.508377000
1	4.388182000	-1.591920000	1.109698000
1	3.259390000	-0.310990000	0.897050000
1	0.827309000	-7.796572000	-2.578146000
1	-11.380175000	0.276731000	-2.706519000
1	3.377622000	3.477750000	-0.707587000
1	3.808778000	6.137770000	0.479366000
6	2.384085000	7.731042000	0.692487000
8	1.420037000	8.268210000	1.225551000
8	3.103834000	8.359204000	-0.250883000
1	2.709531000	9.244553000	-0.379875000
1	-0.237960000	-6.398770000	-2.515877000

D

D-noNi.gjf.log

6	-1.004572000	7.373654000	1.979822000
6	-0.641465000	6.266684000	1.017662000
6	0.249271000	6.495327000	-0.043164000
6	-1.159042000	4.973863000	1.160031000
6	0.611556000	5.479465000	-0.925594000
6	-0.795472000	3.938653000	0.296034000
6	0.094259000	4.189065000	-0.755798000
8	0.491432000	3.224062000	-1.635666000
6	0.764152000	-3.831418000	3.411670000
6	2.935400000	-4.397852000	-1.897268000
6	7.849827000	1.186517000	-2.405677000
6	1.175221000	3.957598000	5.250048000
7	-0.415862000	-1.511293000	1.417569000
6	0.801718000	-4.571629000	4.754635000
6	4.288325000	-3.822087000	-2.364831000

7	0.757207000	-2.095002000	-1.241032000
8	8.096593000	0.290406000	-1.622659000
8	2.158824000	4.243064000	5.909796000
7	1.272487000	0.958308000	1.430369000
8	2.363620000	-5.635406000	6.192159000
8	6.283188000	-5.119676000	-1.842988000
8	2.716193000	-5.866986000	3.982059000
8	5.366547000	-3.811810000	-0.254301000
6	-0.756699000	0.467646000	2.771231000
6	0.165574000	-3.768592000	0.481207000
6	2.347750000	-1.330522000	-2.916162000
6	2.972765000	2.226336000	0.237441000
6	-0.761330000	-1.011600000	2.549060000
6	-0.043746000	-3.317265000	-0.975207000
6	2.624216000	-0.079336000	-2.435065000
6	2.110402000	1.971053000	1.330714000
6	-1.166004000	-2.032276000	3.615051000
6	0.353145000	-4.349406000	-2.116991000
6	3.430624000	0.955352000	-3.198736000
6	2.118721000	2.887556000	2.556063000
6	-0.628243000	-3.357173000	2.965124000
6	1.704499000	-3.787535000	-2.614694000
6	4.016650000	1.791871000	-2.039211000
6	0.831174000	2.425230000	3.277794000
6	-0.676543000	-2.969097000	1.464241000
6	1.577524000	-2.319104000	-2.246837000
6	3.029153000	1.489819000	-0.919172000
6	0.684845000	0.979061000	2.784295000
6	-2.731411000	-2.016611000	3.653252000
6	2.516312000	1.786757000	-4.113308000
6	2.150664000	4.402738000	2.223664000
7	-1.453099000	-3.067375000	-1.252234000
6	-3.321956000	-3.217057000	4.380519000
6	-1.888781000	-3.440831000	-2.487775000
6	3.299178000	2.714633000	-5.007357000
6	3.223736000	5.227649000	2.979167000
8	-3.159688000	-3.415538000	5.583417000
8	-3.005728000	-3.212310000	-2.946341000
8	4.504052000	2.697767000	-5.184878000
6	-0.746022000	-4.138367000	-3.188700000
6	5.439580000	1.347190000	-1.646215000
7	-4.047584000	-4.067320000	3.599785000
8	2.491711000	3.598511000	-5.635156000
6	-0.596795000	-1.682626000	4.998839000
6	0.361260000	-5.816273000	-1.677857000
6	6.496250000	1.807673000	-2.651596000

6	0.817589000	2.555350000	4.812538000
6	2.053345000	-5.412843000	4.895125000
6	5.420215000	-4.335026000	-1.513679000
7	2.280986000	0.383952000	-1.192995000
8	8.802703000	1.743776000	-3.191694000
8	0.329342000	4.883492000	4.757023000
6	-10.944324000	2.332428000	-1.511580000
6	-10.502930000	2.652905000	-0.074507000
6	-9.868215000	1.462383000	0.667421000
6	-8.672036000	0.760095000	0.002613000
6	-7.313942000	1.469246000	0.053251000
16	-7.183699000	2.869403000	-1.160697000
6	-0.888313000	0.367179000	-3.078574000
8	-0.004584000	0.583328000	-5.963925000
16	-1.725117000	0.883750000	-1.529614000
6	-1.791184000	-0.182855000	-4.169195000
8	-1.795263000	-1.020263000	-6.664910000
16	-0.792953000	-0.644361000	-5.630845000
8	0.070996000	-1.780215000	-5.180547000
6	-8.461733000	-2.452436000	-1.710106000
6	-7.094656000	-2.145737000	-1.160396000
6	-6.129015000	-1.545335000	-1.978291000
6	-6.764606000	-2.373960000	0.182646000
6	-4.872530000	-1.191987000	-1.490646000
6	-5.517323000	-2.012146000	0.693045000
6	-4.564572000	-1.426443000	-0.147644000
8	-3.328309000	-1.136022000	0.380302000
6	4.645942000	-3.176068000	4.514768000
6	4.471371000	-3.110059000	2.999734000
6	3.707488000	-1.862290000	2.563091000
7	3.807273000	-1.540522000	1.249373000
8	3.042933000	-1.200791000	3.368561000
1	-1.616079000	8.147052000	1.496539000
1	-1.570852000	6.986372000	2.833280000
1	0.678566000	7.486505000	-0.174141000
1	-1.838541000	4.757794000	1.981292000
1	1.307417000	5.661905000	-1.739247000
1	-1.179230000	2.935231000	0.447036000
1	0.158900000	2.353109000	-1.336025000
1	-8.457803000	-2.409164000	-2.804882000
1	-9.193521000	-1.708695000	-1.368758000
1	-6.354260000	-1.361763000	-3.025810000
1	-7.496620000	-2.830328000	0.845243000
1	-4.123377000	-0.777379000	-2.153633000
1	-5.283045000	-2.169833000	1.741243000
1	-2.837392000	-0.537038000	-0.230641000

1	1.134376000	-4.540389000	2.666949000
1	1.471142000	-2.997135000	3.423982000
1	2.946965000	-5.480063000	-2.065548000
1	2.837429000	-4.241620000	-0.817267000
1	0.733834000	-3.908428000	5.618346000
1	-0.049870000	-5.263447000	4.834441000
1	4.489330000	-4.098269000	-3.403056000
1	4.272804000	-2.729973000	-2.293528000
1	-1.301432000	0.961407000	1.958276000
1	-1.253642000	0.718206000	3.712813000
1	1.229979000	-3.674737000	0.712217000
1	-0.116989000	-4.819375000	0.594351000
1	2.743590000	-1.600721000	-3.888118000
1	4.204232000	0.482729000	-3.810033000
1	2.996790000	2.619839000	3.165168000
1	-1.328904000	-4.181720000	3.138761000
1	1.821974000	-3.925454000	-3.695303000
1	4.025059000	2.864256000	-2.266871000
1	-0.001767000	3.012211000	2.874336000
1	-1.721560000	-3.087867000	1.152138000
1	1.294783000	0.310920000	3.406291000
1	-3.119921000	-1.942940000	2.634752000
1	-3.058131000	-1.116458000	4.185446000
1	1.891561000	1.153771000	-4.758950000
1	1.817907000	2.385996000	-3.517677000
1	1.170101000	4.817217000	2.451416000
1	2.285788000	4.557742000	1.151722000
1	3.353899000	4.851950000	3.996466000
1	-0.425523000	-3.463265000	-3.992153000
1	-1.085884000	-5.071492000	-3.648259000
1	5.463432000	0.253976000	-1.558158000
1	5.684681000	1.741354000	-0.653345000
1	0.495482000	-1.626771000	4.980227000
1	-0.917730000	-2.421421000	5.734980000
1	-0.974580000	-0.711224000	5.335052000
1	0.638821000	-6.455489000	-2.523835000
1	1.058181000	-6.020351000	-0.860810000
1	-0.640291000	-6.117945000	-1.349464000
1	6.198369000	1.573734000	-3.680907000
1	6.608254000	2.898777000	-2.633780000
1	1.540249000	1.873565000	5.269144000
1	-0.180998000	2.302358000	5.189817000
1	-10.089109000	2.343660000	-2.197460000
1	-11.671439000	3.064381000	-1.879846000
1	-9.812867000	3.506251000	-0.087622000
1	-11.373859000	2.977544000	0.510813000

1	-9.574040000	1.781890000	1.677491000
1	-10.651610000	0.703041000	0.804679000
1	-8.518825000	-0.195266000	0.519448000
1	-8.906783000	0.502264000	-1.036636000
1	-6.530449000	0.752554000	-0.203889000
1	-7.119260000	1.854800000	1.059188000
1	-5.835209000	2.933394000	-1.187053000
1	-0.170341000	-0.384579000	-2.752165000
1	-0.335081000	1.231908000	-3.452858000
1	-2.329003000	-1.079621000	-3.851873000
1	-2.510473000	0.562294000	-4.521341000
1	5.175543000	-2.291209000	4.884045000
1	3.678656000	-3.209938000	5.021604000
1	5.443518000	-3.135981000	2.492356000
1	3.911553000	-3.987020000	2.648599000
1	-8.876416000	-3.430293000	-1.431838000
1	-0.105968000	7.863639000	2.376489000
1	5.223878000	-4.061501000	4.800667000
6	-2.876545000	2.160981000	-2.143292000
1	-3.266146000	2.684606000	-1.271048000
1	-2.334546000	2.871005000	-2.767722000
1	-3.702836000	1.723774000	-2.701868000
1	3.160916000	-6.199786000	6.202050000
1	6.108601000	-4.192438000	0.252912000
1	9.638859000	1.279699000	-2.994320000
1	3.058614000	4.137010000	-6.220053000
1	0.793157000	5.756971000	4.763280000
1	-4.415099000	-4.912915000	4.015838000
1	-4.117019000	-3.963080000	2.597832000
1	4.250322000	-2.178359000	0.598021000
1	3.161753000	-0.850978000	0.869483000
1	-2.004904000	-2.422209000	-0.698803000
1	-11.390514000	1.332824000	-1.587342000
1	3.635175000	3.082335000	0.310825000
1	4.187761000	5.187926000	2.465597000
6	2.750977000	6.653556000	3.100693000
8	1.868818000	7.034344000	3.861650000
8	3.355177000	7.496422000	2.247820000
1	2.953480000	8.378965000	2.371708000

E

E-noNi.gjf.log

6	1.132166000	-3.538850000	6.669017000
6	0.733118000	-3.524052000	5.213606000
6	-0.249857000	-4.395914000	4.718124000
6	1.311820000	-2.620331000	4.316462000

6	-0.655612000	-4.356698000	3.385251000
6	0.919049000	-2.566993000	2.978933000
6	-0.072704000	-3.434687000	2.507669000
8	-0.505916000	-3.418832000	1.213528000
6	-0.443102000	5.113737000	0.409137000
6	-3.304563000	2.003575000	-3.793886000
6	-7.867381000	-2.545911000	-0.298062000
6	-0.518836000	0.844240000	6.806740000
7	0.553830000	2.005816000	0.209590000
6	-0.274731000	6.574509000	0.887549000
6	-4.617221000	1.211700000	-3.680970000
7	-0.899802000	0.744899000	-2.027948000
8	-8.113097000	-1.361425000	-0.421889000
8	-1.358073000	1.012172000	7.668217000
7	-1.153137000	0.257647000	1.982102000
8	-2.074309000	6.707192000	2.418805000
8	-5.996429000	3.211708000	-3.731143000
8	-2.269485000	7.916500000	0.526390000
8	-6.517419000	1.551648000	-2.296010000
6	0.998910000	1.389428000	2.496870000
6	-0.241540000	3.132090000	-1.873706000
6	-2.530442000	-0.953993000	-2.621742000
6	-2.898679000	-1.477279000	2.158401000
6	1.034360000	2.344578000	1.349964000
6	-0.157655000	1.841398000	-2.711064000
6	-2.719861000	-1.579357000	-1.428531000
6	-2.008144000	-0.466104000	2.669676000
6	1.581005000	3.772487000	1.426905000
6	-0.735721000	1.896037000	-4.193426000
6	-3.491211000	-2.867765000	-1.275189000
6	-1.995881000	-0.116135000	4.156284000
6	0.894526000	4.397376000	0.157253000
6	-2.065439000	1.114467000	-4.064372000
6	-4.009191000	-2.724473000	0.167304000
6	-0.623543000	0.546263000	4.299482000
6	0.761805000	3.131376000	-0.729498000
6	-1.798255000	0.251655000	-2.844584000
6	-3.007311000	-1.746075000	0.775658000
6	-0.461037000	1.176897000	2.913430000
6	3.131658000	3.638756000	1.229295000
6	-2.580543000	-4.092683000	-1.455496000
6	-2.285683000	-1.312264000	5.051615000
7	1.225339000	1.438842000	-2.923027000
6	3.807963000	4.966552000	0.915609000
6	1.524981000	0.948223000	-4.156999000
6	-3.362842000	-5.381756000	-1.456367000

6	-3.606875000	-1.928771000	4.612893000
6	-3.686083000	-2.266587000	3.123554000
8	3.914985000	5.872775000	1.739752000
8	2.606597000	0.461956000	-4.479119000
8	-4.570863000	-5.492395000	-1.562287000
6	0.290440000	1.076778000	-5.019309000
6	-5.427371000	-2.110647000	0.203456000
7	4.291442000	5.083932000	-0.354577000
8	-2.549121000	-6.455865000	-1.345738000
8	-4.453876000	-3.187328000	2.812799000
6	1.290714000	4.455232000	2.772629000
6	-0.855258000	3.306142000	-4.778023000
6	-6.505381000	-3.155717000	-0.076068000
6	-0.474561000	1.535999000	5.463528000
6	-1.620817000	7.164691000	1.226383000
6	-5.760764000	2.110372000	-3.279693000
7	-2.325162000	-1.092663000	-0.191214000
8	-8.834202000	-3.493701000	-0.362998000
8	0.503706000	-0.034193000	6.948005000
6	10.917228000	-3.012944000	0.313904000
6	10.527349000	-2.123501000	1.505545000
6	9.914548000	-0.769418000	1.103763000
6	8.701355000	-0.786321000	0.158547000
6	7.343346000	-1.179459000	0.750959000
16	7.176273000	-3.009667000	1.022263000
6	0.714812000	-2.350346000	-1.966849000
8	-0.391508000	-4.320425000	-3.961458000
16	1.678936000	-1.782666000	-0.516239000
6	1.500443000	-2.619882000	-3.238421000
8	1.239620000	-3.542188000	-5.694196000
16	0.356265000	-3.169090000	-4.556875000
8	-0.524276000	-1.989819000	-4.831008000
6	8.355093000	-0.065475000	-3.407716000
6	7.022929000	0.211735000	-2.761538000
6	5.987380000	-0.728056000	-2.836658000
6	6.792963000	1.379770000	-2.020770000
6	4.763647000	-0.519962000	-2.202193000
6	5.582091000	1.595997000	-1.362554000
6	4.562284000	0.643449000	-1.453813000
8	3.372702000	0.905043000	-0.813407000
6	-4.630797000	5.232433000	0.384397000
6	-4.903117000	3.871471000	-0.248817000
6	-3.942227000	2.798655000	0.269029000
7	-4.223858000	1.526971000	-0.108620000
8	-2.966107000	3.083060000	0.967912000
1	1.726128000	-4.427153000	6.922191000

1	1.729483000	-2.657137000	6.923098000
1	-0.725782000	-5.103127000	5.394020000
1	2.067474000	-1.923566000	4.671653000
1	-1.436740000	-5.013222000	3.014275000
1	1.358767000	-1.839875000	2.306200000
1	-0.120418000	-2.643081000	0.755487000
1	8.290402000	-0.923153000	-4.085909000
1	9.113744000	-0.310093000	-2.653640000
1	6.130991000	-1.638535000	-3.413070000
1	7.578516000	2.128187000	-1.943289000
1	3.961157000	-1.238947000	-2.310392000
1	5.427163000	2.493427000	-0.771798000
1	2.852028000	0.069718000	-0.732499000
1	-1.002728000	5.155351000	-0.530681000
1	-1.074115000	4.545696000	1.096708000
1	-3.416991000	2.738757000	-4.595578000
1	-3.144243000	2.565158000	-2.865075000
1	0.366436000	6.634064000	1.768210000
1	0.171654000	7.179373000	0.093634000
1	-4.868212000	0.764081000	-4.651782000
1	-4.533391000	0.394029000	-2.964591000
1	1.419909000	0.425905000	2.193257000
1	1.583843000	1.763157000	3.341116000
1	-1.262428000	3.230780000	-1.493632000
1	-0.021811000	4.000336000	-2.501470000
1	-2.962274000	-1.413997000	-3.502842000
1	-4.304159000	-2.930156000	-2.003651000
1	-2.780016000	0.644905000	4.318062000
1	1.566743000	5.108069000	-0.336035000
1	-2.256857000	0.505162000	-4.954502000
1	-4.013748000	-3.667942000	0.708451000
1	0.129695000	-0.241677000	4.406352000
1	1.750558000	2.972982000	-1.176517000
1	-0.988563000	2.143336000	2.880737000
1	3.343653000	2.897020000	0.455666000
1	3.566574000	3.262312000	2.161434000
1	-2.021278000	-4.049483000	-2.401346000
1	-1.832542000	-4.141688000	-0.656800000
1	-2.335930000	-1.007624000	6.102689000
1	-1.477889000	-2.043372000	4.955544000
1	-4.429395000	-1.226166000	4.819537000
1	-3.836386000	-2.849651000	5.157282000
1	-0.049252000	0.052949000	-5.213880000
1	0.537714000	1.540872000	-5.979034000
1	-5.491305000	-1.306973000	-0.540756000
1	-5.614590000	-1.656393000	1.179033000

1	0.218398000	4.559153000	2.960066000
1	1.763066000	5.438395000	2.807107000
1	1.719007000	3.873469000	3.595253000
1	-1.263676000	3.252861000	-5.793604000
1	-1.501537000	3.963762000	-4.190662000
1	0.133589000	3.774671000	-4.843644000
1	-6.256427000	-3.762336000	-0.956001000
1	-6.570485000	-3.865999000	0.756672000
1	-1.268103000	2.287843000	5.444262000
1	0.490558000	2.051714000	5.384092000
1	10.040287000	-3.528455000	-0.094988000
1	11.642775000	-3.779677000	0.606738000
1	9.840185000	-2.674344000	2.160523000
1	11.418828000	-1.913138000	2.111692000
1	9.648645000	-0.209207000	2.011664000
1	10.701954000	-0.185543000	0.605638000
1	8.565665000	0.232157000	-0.225280000
1	8.907360000	-1.410816000	-0.718383000
1	6.554537000	-0.888005000	0.053372000
1	7.175930000	-0.664659000	1.702489000
1	5.827170000	-3.043016000	1.068244000
1	-0.008797000	-1.552013000	-2.125050000
1	0.174464000	-3.250276000	-1.665355000
1	2.011863000	-1.728933000	-3.610900000
1	2.226590000	-3.428164000	-3.110976000
1	-4.857838000	5.224560000	1.455753000
1	-3.576136000	5.490588000	0.273027000
1	-5.936873000	3.546149000	-0.075927000
1	-4.784544000	3.931037000	-1.339184000
1	8.759846000	0.775368000	-3.986229000
1	0.250278000	-3.546633000	7.322492000
1	-5.216891000	6.024939000	-0.093289000
6	2.777038000	-3.209721000	-0.217137000
1	3.547936000	-3.294731000	-0.981354000
1	3.246524000	-3.051663000	0.753161000
1	2.183626000	-4.122950000	-0.178728000
1	-2.972079000	7.070046000	2.543719000
1	-7.242235000	2.174267000	-2.090282000
1	-9.676200000	-3.026870000	-0.525696000
1	-3.116825000	-7.249012000	-1.392664000
1	0.390835000	-0.471797000	7.813358000
1	4.680378000	5.971894000	-0.643970000
1	4.116253000	4.390728000	-1.067670000
1	-5.021767000	1.312515000	-0.692658000
1	-3.535206000	0.800518000	0.065391000
1	1.876133000	1.285400000	-2.159729000

1	11.345041000	-2.428452000	-0.510376000

x) Single point structure of reactants of cofactor **A-E** without presence of Ni as mentioned in **Figure 4.**

A

A-noNi-cofactor.gjf.log

6	5.042923000	-1.311170000	1.039976000
6	1.909202000	4.495215000	1.424308000
6	-7.563337000	0.927317000	-1.122287000
6	-0.802230000	-6.076067000	-0.924777000
7	1.859058000	-1.072866000	-0.104385000
6	6.417113000	-1.985054000	1.018140000
6	0.735404000	5.190423000	2.138020000
7	1.073110000	1.749147000	-0.139582000
8	-8.290252000	0.988778000	-0.149743000
8	0.328271000	-6.347544000	-1.281907000
7	-0.944301000	-1.802361000	0.217870000
8	8.455868000	-2.248473000	2.197317000
8	1.110424000	6.790018000	3.930098000
8	7.045846000	-0.672849000	2.972571000
8	1.447859000	4.597848000	4.321219000
6	0.981037000	-3.337349000	0.117004000
6	3.316292000	0.849693000	-0.468777000
6	-0.838986000	3.273633000	0.000335000
6	-3.247293000	-0.938545000	0.308487000
6	2.000444000	-2.443589000	-0.064340000
6	2.389918000	1.884308000	-0.407802000
6	-1.895527000	2.328708000	0.076287000
6	-2.317483000	-1.976046000	0.309949000
6	3.440977000	-2.875216000	-0.394448000
6	2.755035000	3.342409000	-0.728165000
6	-3.299720000	2.660821000	0.150501000
6	-2.640262000	-3.381039000	0.397979000
6	4.207576000	-1.522696000	-0.242772000
6	1.577811000	4.080397000	-0.029305000
6	-3.988305000	1.468097000	0.242400000
6	-1.434768000	-4.050237000	0.376783000
6	3.067235000	-0.510570000	-0.286184000
6	0.502045000	3.004317000	-0.065414000
6	-2.987570000	0.428476000	0.226554000
6	-0.401712000	-3.047790000	0.247070000
6	3.432818000	-3.305519000	-1.883615000
6	-3.886222000	4.048132000	0.094560000
6	-4.019045000	-3.958518000	0.536592000

6	4.778266000	-3.681349000	-2.468270000
6	2.931827000	4.868428000	-2.788032000
6	-3.964746000	4.524518000	-1.343941000
6	-4.542591000	-3.865640000	1.978660000
8	5.871662000	-3.525811000	-1.958347000
8	4.041954000	5.292416000	-3.045594000
8	-4.809581000	4.189734000	-2.152657000
6	2.618776000	3.480001000	-2.287024000
6	-5.474925000	1.267654000	0.281744000
8	-2.946777000	5.355066000	-1.665067000
6	3.917585000	-4.048221000	0.476546000
6	4.164119000	3.755759000	-0.286441000
6	-6.068042000	1.127766000	-1.129649000
6	-1.198299000	-5.538591000	0.437329000
6	7.297133000	-1.544592000	2.164469000
6	1.104060000	5.642440000	3.529109000
7	-1.720201000	0.981606000	0.125594000
8	-8.039368000	0.671440000	-2.365540000
8	-1.871108000	-6.191916000	-1.746069000
1	5.200413000	-0.235112000	1.166643000
1	4.475292000	-1.632041000	1.921417000
1	2.765264000	5.179817000	1.414111000
1	2.207832000	3.612312000	2.000376000
1	6.353974000	-3.075526000	1.044864000
1	6.946217000	-1.753580000	0.084486000
1	0.406125000	6.072116000	1.580723000
1	-0.109279000	4.497072000	2.212564000
1	1.240387000	-4.389487000	0.088776000
1	4.348176000	1.121866000	-0.667111000
1	-1.122860000	4.320983000	-0.012119000
1	4.878627000	-1.364838000	-1.093398000
1	1.273244000	4.974708000	-0.582349000
1	3.032825000	-2.492071000	-2.504681000
1	2.750536000	-4.151310000	-2.028208000
1	-4.907653000	4.043523000	0.486949000
1	-3.295000000	4.757801000	0.681551000
1	-4.029693000	-5.006187000	0.217656000
1	-4.722796000	-3.440354000	-0.125491000
1	-3.881914000	-4.404523000	2.669391000
1	3.327800000	2.793159000	-2.756459000
1	1.603275000	3.200943000	-2.581666000
1	-5.964640000	2.111381000	0.782146000
1	-5.733435000	0.380340000	0.870743000
1	3.901849000	-3.791578000	1.539599000
1	4.927437000	-4.358338000	0.201018000
1	3.261811000	-4.914084000	0.337795000

1	4.312600000	4.834547000	-0.395023000
1	4.349770000	3.480721000	0.755228000
1	4.923360000	3.265640000	-0.904155000
1	-5.846571000	2.027475000	-1.717976000
1	-5.606158000	0.290691000	-1.666905000
1	-2.104716000	-6.054637000	0.768565000
1	-0.390133000	-5.786746000	1.131734000
1	8.974578000	-1.902655000	2.948866000
1	1.681431000	4.967213000	5.194250000
1	-9.006748000	0.568173000	-2.281685000
1	-3.030983000	5.559267000	-2.616729000
1	-1.534370000	-6.485354000	-2.614376000
1	5.530833000	-4.424027000	-4.029432000
1	2.120631000	6.526897000	-3.181189000
1	-4.292909000	-1.227421000	0.375549000
1	-4.544174000	-2.824760000	2.324895000
6	-5.940247000	-4.416427000	2.137402000
8	-6.625954000	-4.891721000	1.254223000
8	-6.369767000	-4.322156000	3.419100000
1	-7.274104000	-4.689931000	3.442763000
8	1.822488000	5.641049000	-2.896591000
8	4.633028000	-4.226030000	-3.699491000

B

B-noNi-cofactor.gjf.log

6	-4.440021000	0.976920000	1.888113000
6	-0.401488000	-4.919696000	1.077413000
6	7.412895000	0.050540000	0.485257000
6	-0.319603000	5.962691000	0.598321000
7	-2.005157000	0.638388000	-0.267022000
6	-5.841557000	1.421734000	2.312055000
6	1.087758000	-5.260769000	1.133823000
7	-0.789416000	-1.999530000	-0.452183000
8	8.228770000	0.140112000	-0.411614000
8	-1.472880000	6.058925000	0.967321000
7	0.546698000	1.874023000	-0.804704000
8	-7.208810000	2.024734000	4.151629000
8	2.807015000	-6.298200000	2.450328000
8	-5.164094000	1.216823000	4.642326000
8	0.748725000	-6.158326000	3.337152000
6	-1.628908000	3.018532000	-0.667048000
6	-3.081246000	-1.503436000	0.205330000
6	1.274445000	-3.136194000	-1.099673000
6	2.952637000	1.424959000	-1.048453000
6	-2.441102000	1.938412000	-0.464191000

6	-2.051853000	-2.368094000	-0.146068000
6	2.147671000	-2.020698000	-1.184988000
6	1.863306000	2.289105000	-0.923948000
6	-3.974613000	1.969394000	-0.512856000
6	-2.277801000	-3.864728000	-0.391640000
6	3.587196000	-2.117772000	-1.285198000
6	1.932384000	3.730326000	-0.939507000
6	-4.296177000	0.727357000	0.369416000
6	-0.812944000	-4.387360000	-0.323104000
6	4.073517000	-0.829457000	-1.231077000
6	0.627962000	4.175092000	-0.851237000
6	-3.056486000	-0.114703000	0.104139000
6	-0.035711000	-3.126594000	-0.694452000
6	2.920190000	0.033813000	-1.111437000
6	-0.209576000	3.001981000	-0.761738000
6	-4.352478000	1.639973000	-2.001941000
6	4.370144000	-3.402326000	-1.263140000
6	3.189108000	4.549817000	-0.962113000
7	-5.195633000	-3.815795000	-1.978491000
6	-5.811196000	1.244655000	-2.198528000
6	-3.997225000	-3.243411000	-2.262435000
6	4.302288000	-4.061907000	0.112169000
6	3.791107000	4.691979000	0.445447000
8	-6.744607000	2.034375000	-2.026714000
8	-3.918792000	-2.132466000	-2.797315000
8	4.483064000	-3.480812000	1.167139000
6	-2.760423000	-4.045287000	-1.878648000
6	5.508243000	-0.400985000	-1.139349000
7	-6.013787000	-0.047292000	-2.568863000
8	3.994273000	-5.371564000	0.045449000
6	-4.601768000	3.299351000	-0.090613000
6	-3.256700000	-4.536634000	0.577311000
6	5.954045000	-0.300138000	0.329281000
6	0.138922000	5.599295000	-0.800434000
6	-5.993405000	1.529150000	3.811808000
6	1.634466000	-5.954359000	2.351627000
7	1.758874000	-0.725222000	-1.071866000
8	7.741728000	0.255569000	1.784372000
8	0.728067000	6.145115000	1.440422000
1	-4.207960000	0.043365000	2.412728000
1	-3.691452000	1.704843000	2.222342000
1	-0.994620000	-5.809896000	1.312203000
1	-0.631102000	-4.168688000	1.842055000
1	-6.126596000	2.384159000	1.877763000
1	-6.596610000	0.706141000	1.957787000
1	1.382220000	-5.882924000	0.280718000

1	1.681191000	-4.346869000	1.038366000
1	-2.104072000	3.990228000	-0.745786000
1	-4.041073000	-1.943877000	0.454938000
1	1.724888000	-4.109059000	-1.268410000
1	-5.195669000	0.205440000	0.024029000
1	-0.643343000	-5.192583000	-1.049201000
1	-3.697105000	0.843449000	-2.366847000
1	-4.162047000	2.531795000	-2.608364000
1	4.022708000	-4.117658000	-2.015531000
1	5.428643000	-3.203782000	-1.470970000
1	2.993618000	5.551275000	-1.361984000
1	3.937098000	4.102279000	-1.627065000
1	3.063036000	5.159698000	1.119877000
1	-1.961037000	-3.709551000	-2.544667000
1	-2.928148000	-5.114813000	-2.058550000
1	5.667269000	0.564938000	-1.632806000
1	6.160892000	-1.113391000	-1.656584000
1	-4.304711000	3.581293000	0.924173000
1	-5.690809000	3.250301000	-0.159217000
1	-4.275798000	4.101573000	-0.761889000
1	-3.305302000	-5.616625000	0.391446000
1	-2.961648000	-4.381216000	1.618176000
1	-4.266589000	-4.132989000	0.464678000
1	5.356006000	0.447111000	0.864990000
1	5.766909000	-1.252819000	0.842367000
1	-0.712532000	5.759083000	-1.468918000
1	0.940025000	6.285624000	-1.094086000
1	-7.237599000	2.057628000	5.127095000
1	1.224101000	-6.588030000	4.075331000
1	8.695842000	0.462352000	1.804612000
1	3.820834000	-5.704507000	0.962995000
1	0.355767000	6.315046000	2.326839000
1	-6.969954000	-0.351689000	-2.696897000
1	-5.264380000	-0.731985000	-2.681089000
1	-6.047915000	-3.313604000	-2.186686000
1	3.932832000	1.889459000	-1.120364000
1	4.011710000	3.707482000	0.875695000
6	5.054018000	5.517837000	0.469321000
8	5.542288000	6.108165000	-0.474194000
8	5.610402000	5.543561000	1.705439000
1	6.411428000	6.098577000	1.642915000
1	-5.271898000	-4.730607000	-1.560227000

C

C-noNi-cofactor.gjf.log

6	-4.447103000	0.771047000	1.324081000
6	-1.468392000	-4.339402000	1.636295000
6	6.659595000	-2.402424000	1.972885000
6	1.871936000	5.976140000	1.273618000
7	-2.117894000	0.812319000	-0.836244000
6	-5.687274000	1.289828000	2.054200000
6	-0.116402000	-4.773437000	2.232646000
7	-1.150926000	-1.974399000	-0.550586000
8	6.236967000	-1.929278000	3.009064000
8	2.656512000	5.967483000	2.205371000
7	0.687114000	1.608370000	-0.313418000
8	-6.797399000	1.362421000	4.145581000
8	0.013645000	-5.903778000	4.380853000
8	-4.972080000	0.046204000	4.020359000
8	-0.472392000	-3.704071000	4.317703000
6	-1.238857000	3.089125000	-0.827432000
6	-3.519494000	-1.305827000	-0.571033000
6	0.852586000	-3.379310000	-0.569646000
6	3.010789000	0.906376000	-0.501328000
6	-2.337950000	2.076906000	-0.723862000
6	-2.540654000	-2.217063000	-0.487806000
6	1.821815000	-2.415403000	-0.769417000
6	1.978861000	1.851284000	-0.255899000
6	-3.813901000	2.470512000	-0.574979000
6	-2.800826000	-3.729145000	-0.493797000
6	3.273358000	-2.754352000	-1.069569000
6	2.322406000	3.262477000	0.209515000
6	-4.418209000	1.084004000	-0.184954000
6	-1.461185000	-4.242079000	0.093026000
6	3.991739000	-1.432726000	-0.713649000
6	0.959036000	3.975889000	0.088503000
6	-3.437200000	0.137840000	-0.928021000
6	-0.518426000	-3.140460000	-0.370933000
6	2.829911000	-0.442571000	-0.672237000
6	-0.054660000	2.812774000	0.099172000
6	-4.299789000	2.927896000	-1.994263000
6	3.414332000	-3.148726000	-2.548676000
6	3.481426000	3.966520000	-0.552759000
7	-3.238282000	-2.484066000	-3.666928000
6	-5.798923000	3.208198000	-2.051485000
6	-3.825095000	-3.401492000	-2.839538000
6	4.801095000	-3.637082000	-2.904898000
6	4.696198000	4.337080000	0.330580000
8	-6.299561000	4.207805000	-1.540054000
8	-5.043553000	-3.564480000	-2.802186000
8	5.654682000	-3.993358000	-2.116780000

6	-2.859382000	-4.212311000	-1.986684000
6	4.713206000	-1.482799000	0.649335000
7	-6.550631000	2.262845000	-2.679390000
8	4.985834000	-3.664668000	-4.242351000
6	-4.024327000	3.606886000	0.438478000
6	-4.069571000	-4.168837000	0.241850000
6	5.996654000	-2.312608000	0.619102000
6	0.680239000	5.053233000	1.159554000
6	-5.745928000	0.817538000	3.490242000
6	-0.173902000	-4.891104000	3.737128000
7	1.619017000	-1.077014000	-0.698644000
8	7.820321000	-3.097173000	1.910409000
8	2.043298000	6.732687000	0.173415000
1	-4.421034000	-0.315983000	1.449561000
1	-3.538097000	1.139104000	1.813136000
1	-2.233636000	-5.063504000	1.937265000
1	-1.756448000	-3.370945000	2.060048000
1	-5.756484000	2.381263000	2.061070000
1	-6.602771000	0.940210000	1.557528000
1	0.191640000	-5.745053000	1.836587000
1	0.655635000	-4.039823000	1.975640000
1	-0.867752000	3.096580000	-1.861574000
1	-1.626190000	4.089871000	-0.618744000
1	-4.539473000	-1.680719000	-0.572924000
1	1.179235000	-4.414033000	-0.535807000
1	3.619104000	-3.590814000	-0.455022000
1	2.593293000	3.184826000	1.273477000
1	-5.429073000	0.964968000	-0.589780000
1	-1.182994000	-5.219074000	-0.320400000
1	4.715479000	-1.140928000	-1.484935000
1	0.917610000	4.457258000	-0.896990000
1	-3.690755000	0.170541000	-2.003504000
1	-0.434979000	2.645053000	1.118423000
1	-4.015975000	2.183857000	-2.746072000
1	-3.789027000	3.861343000	-2.251574000
1	2.715793000	-3.959655000	-2.793811000
1	3.150053000	-2.311163000	-3.204386000
1	3.091119000	4.879182000	-1.007141000
1	3.821577000	3.339636000	-1.383455000
1	4.356682000	4.548115000	1.350723000
1	-1.854356000	-4.173757000	-2.420096000
1	-3.190005000	-5.255802000	-2.001970000
1	4.027296000	-1.886118000	1.404711000
1	4.953469000	-0.463608000	0.969749000
1	-3.584699000	3.365251000	1.410408000
1	-5.087754000	3.814224000	0.565015000

1	-3.567362000	4.535778000	0.083919000
1	-4.087003000	-5.259750000	0.348386000
1	-4.135315000	-3.719814000	1.236893000
1	-4.957714000	-3.884107000	-0.328166000
1	5.817179000	-3.330114000	0.255931000
1	6.721937000	-1.887616000	-0.086675000
1	0.525551000	4.590549000	2.137818000
1	-0.217584000	5.620807000	0.890417000
1	-6.781354000	1.009643000	5.056105000
1	-0.503811000	-3.855476000	5.281833000
1	8.187799000	-3.119685000	2.814905000
1	5.880572000	-4.022442000	-4.403575000
1	2.991427000	7.013402000	0.147333000
1	-7.548736000	2.408272000	-2.754288000
1	-6.148599000	1.469928000	-3.156557000
1	-3.850412000	-1.809771000	-4.109215000
1	4.033193000	1.266223000	-0.446626000
1	5.426769000	3.526326000	0.381177000
6	5.366780000	5.612096000	-0.125071000
8	4.796996000	6.681159000	-0.300326000
8	6.689922000	5.480756000	-0.306177000
1	7.041230000	6.355838000	-0.564974000
1	-2.289714000	-2.179648000	-3.489769000

D

D-noNi-cofactor.gjf.log

7	1.450890000	1.127341000	-0.335493000
7	1.265201000	-1.779825000	-0.350297000
7	-1.745260000	-1.540473000	-0.551061000
7	-1.325696000	1.197690000	0.132785000
6	1.290835000	2.485322000	-0.318150000
6	2.634868000	3.210957000	-0.339582000
6	2.768454000	0.822240000	-0.411216000
6	3.622680000	2.060927000	-0.650859000
6	-2.683477000	0.878096000	0.582985000
6	-1.149453000	2.497275000	0.093497000
6	-2.416897000	3.286456000	0.406233000
6	-3.302125000	2.210036000	1.083852000
6	0.088008000	3.139141000	-0.182215000
6	-2.981694000	-1.227373000	-0.733350000
6	-1.594894000	-3.007367000	-0.654481000
6	-3.017370000	-3.554880000	-0.378902000
6	-3.877744000	-2.435611000	-1.055851000
6	-3.486168000	0.170348000	-0.516607000
6	0.934781000	-3.211226000	-0.399936000
6	2.585688000	-1.661073000	-0.259570000

6	2.141154000	-3.967256000	0.250182000
6	3.299102000	-3.008875000	-0.169229000
6	-0.443772000	-3.522174000	0.202628000
6	3.305398000	-0.446119000	-0.316608000
1	-0.537564000	-4.611367000	0.287181000
1	-0.494790000	-3.102740000	1.210360000
1	3.581741000	-3.273671000	-1.199443000
6	4.576959000	-3.083160000	0.687034000
1	4.671340000	-4.089943000	1.106620000
1	4.507398000	-2.402249000	1.542109000
6	5.861895000	-2.807259000	-0.100479000
1	5.958519000	-3.514705000	-0.935613000
1	5.877903000	-1.813695000	-0.559797000
6	7.103173000	-2.944770000	0.752476000
8	7.138789000	-3.347936000	1.897364000
8	8.211767000	-2.566482000	0.075158000
1	4.386249000	-0.508169000	-0.312061000
6	2.008233000	-4.127311000	1.764503000
1	1.135933000	-4.736247000	2.020507000
1	2.885689000	-4.625120000	2.186848000
1	1.901960000	-3.152993000	2.254881000
6	2.211838000	-5.288313000	-0.530403000
7	1.012653000	-3.736579000	-1.777431000
6	1.687340000	-4.913917000	-1.919545000
8	1.839488000	-5.536202000	-2.963856000
1	-1.348561000	-3.243335000	-1.695923000
1	-3.159488000	-4.508897000	-0.900783000
6	-3.338279000	-3.809443000	1.108126000
1	-4.349484000	-4.228498000	1.171645000
1	-2.672465000	-4.593359000	1.482496000
6	-3.249538000	-2.611586000	2.068929000
1	-2.233516000	-2.199102000	2.092735000
1	-3.890983000	-1.787695000	1.753908000
6	-3.603011000	-3.016685000	3.494904000
8	-3.345979000	-4.110148000	3.958225000
8	-4.208152000	-2.087746000	4.257264000
6	-5.333416000	-2.339795000	-0.573652000
1	-5.888868000	-1.590253000	-1.146036000
1	-5.833877000	-3.303455000	-0.720547000
1	-5.421296000	-2.077406000	0.482059000
6	-3.899667000	-2.690623000	-2.602318000
1	-4.485849000	-3.597883000	-2.785581000
1	-2.887952000	-2.873239000	-2.973222000
6	-4.429236000	-1.508691000	-3.404889000
8	-3.775186000	-0.473461000	-3.532719000
7	-5.661906000	-1.660163000	-3.954223000

1	-6.044197000	-0.906963000	-4.510604000
1	-6.190997000	-2.516072000	-3.879012000
1	-3.440902000	0.725771000	-1.459164000
1	-4.538768000	0.128493000	-0.220612000
1	-2.585581000	0.171119000	1.409047000
1	3.216872000	-5.714659000	-0.598710000
1	1.556851000	-6.054090000	-0.095021000
1	0.606210000	-3.259398000	-2.571149000
1	-4.352301000	2.292587000	0.789325000
6	-3.208058000	2.325041000	2.611262000
1	-3.599706000	3.292491000	2.953340000
1	-2.152026000	2.296773000	2.918533000
6	-3.923247000	1.229191000	3.376282000
8	-4.555065000	0.317417000	2.863838000
8	-3.822626000	1.265934000	4.714519000
1	-2.190749000	4.111229000	1.095637000
1	4.491482000	2.075099000	0.014608000
1	2.643450000	3.975740000	-1.127026000
6	2.900845000	3.896844000	1.016434000
1	3.000433000	3.128843000	1.793765000
1	2.029496000	4.499476000	1.293368000
6	4.142315000	4.788443000	1.007871000
1	5.038249000	4.239667000	0.698908000
1	4.039919000	5.595703000	0.270781000
6	4.411426000	5.419116000	2.353092000
8	3.762923000	5.247945000	3.366443000
8	5.497066000	6.227573000	2.312779000
6	4.128571000	2.066250000	-2.102907000
1	4.634455000	1.118042000	-2.328624000
1	3.294288000	2.142831000	-2.808807000
6	5.125168000	3.168985000	-2.383703000
8	5.801728000	3.743773000	-1.553307000
8	5.215347000	3.432312000	-3.704973000
1	8.971946000	-2.694125000	0.674906000
1	5.903983000	4.116258000	-3.817257000
1	5.612538000	6.594541000	3.210346000
1	-3.281822000	2.020972000	5.007641000
1	-4.394986000	-1.257082000	3.759203000
1	0.097655000	4.224932000	-0.173334000
6	-3.021029000	3.889381000	-0.877593000
1	-2.246743000	4.453330000	-1.409428000
1	-3.332363000	3.085417000	-1.554259000
6	-4.206053000	4.813167000	-0.595367000
1	-5.014076000	4.290870000	-0.067946000
1	-3.911393000	5.642106000	0.061796000
6	-4.794243000	5.409306000	-1.854829000

8	-4.395283000	5.219647000	-2.985498000
8	-5.851456000	6.206788000	-1.575425000
1	-6.178315000	6.558036000	-2.426089000

E

E-noNi-cofactor.gjf.log

7	-1.549380000	-0.750134000	-0.414384000
7	-0.368227000	1.941033000	-0.171702000
7	2.347886000	0.755257000	-0.653367000
7	1.052523000	-1.802201000	-0.197448000
6	-1.900037000	-2.052751000	-0.542593000
6	-3.415628000	-2.229137000	-0.527954000
6	-2.679518000	0.024880000	-0.377855000
6	-3.919909000	-0.785279000	-0.700389000
6	2.476833000	-2.032664000	0.102361000
6	0.409722000	-2.935471000	-0.253029000
6	1.263116000	-4.144514000	0.080629000
6	2.677706000	-3.562720000	0.274632000
6	-0.986104000	-3.125085000	-0.565128000
6	3.393732000	0.058278000	-0.937637000
6	2.739599000	2.176615000	-0.519292000
6	4.254766000	2.127665000	-0.204421000
6	4.677581000	0.894337000	-1.067282000
6	3.347808000	-1.437307000	-1.013925000
6	0.430772000	3.178170000	-0.121646000
6	-1.638049000	2.266556000	-0.006452000
6	-0.430919000	4.231751000	0.652779000
6	-1.855718000	3.757773000	0.232252000
6	1.837785000	2.941879000	0.439517000
6	-2.732048000	1.372178000	-0.127842000
1	2.296826000	3.919388000	0.629406000
1	1.750184000	2.423862000	1.397086000
1	-2.064558000	4.192246000	-0.757304000
6	-3.010390000	4.168681000	1.165399000
1	-2.744885000	5.098241000	1.678685000
1	-3.156913000	3.419720000	1.951020000
6	-4.330926000	4.426620000	0.432679000
1	-4.201423000	5.209687000	-0.326888000
1	-4.696119000	3.551035000	-0.114000000
6	-5.429689000	4.880142000	1.368081000
8	-5.300071000	5.125252000	2.550244000
8	-6.611651000	4.995927000	0.720722000
1	-3.724302000	1.800690000	-0.063829000
6	-0.209111000	4.202493000	2.165168000
1	0.825277000	4.454936000	2.416497000

1	-0.850938000	4.929354000	2.670664000
1	-0.430123000	3.211324000	2.577245000
6	-0.072269000	5.564829000	-0.021035000
7	0.503704000	3.819976000	-1.445475000
6	0.263287000	5.164159000	-1.460250000
8	0.309120000	5.892465000	-2.443314000
1	2.623037000	2.643282000	-1.504432000
1	4.744104000	3.034224000	-0.579813000
6	4.593443000	2.036190000	1.296925000
1	5.682098000	2.020379000	1.414267000
1	4.263700000	2.964195000	1.776355000
6	4.006403000	0.858831000	2.087454000
1	2.920500000	0.783044000	1.958800000
1	4.408719000	-0.102804000	1.747372000
6	4.302528000	0.970606000	3.573434000
8	4.951909000	1.851297000	4.090155000
8	3.785079000	-0.016953000	4.349413000
6	5.971434000	0.195955000	-0.622584000
1	6.264785000	-0.578610000	-1.338035000
1	6.785961000	0.927697000	-0.577651000
1	5.886963000	-0.285405000	0.351922000
6	4.864093000	1.370258000	-2.548949000
1	5.759142000	2.000884000	-2.588123000
1	4.015641000	1.984384000	-2.862029000
6	4.940344000	0.224932000	-3.550726000
8	3.944602000	-0.433838000	-3.850734000
7	6.165152000	-0.028547000	-4.079816000
1	6.261788000	-0.778402000	-4.751586000
1	6.983127000	0.514267000	-3.846494000
1	2.955474000	-1.743814000	-1.989247000
1	4.357224000	-1.838518000	-0.924096000
1	2.707802000	-1.510282000	1.035380000
1	-0.876926000	6.305348000	-0.003135000
1	0.812263000	6.026824000	0.436048000
1	0.716149000	3.310065000	-2.292807000
1	3.348550000	-3.938728000	-0.505973000
6	3.252620000	-3.949205000	1.642680000
1	3.319376000	-5.042143000	1.730202000
1	2.566274000	-3.624157000	2.439140000
6	4.613001000	-3.352118000	1.962089000
8	5.185900000	-2.516997000	1.293761000
8	5.188888000	-3.782130000	3.107866000
1	0.895165000	-4.569790000	1.027353000
6	1.075248000	-5.205640000	-1.002676000
6	-1.429011000	-4.489995000	-0.895880000
6	-0.395210000	-5.624810000	-0.991361000

1	1.353691000	-4.783067000	-1.977241000
1	1.726575000	-6.069840000	-0.824611000
1	-0.584435000	-6.268981000	-0.120059000
1	-0.663129000	-6.218815000	-1.871192000
8	-2.603738000	-4.808189000	-1.120048000
1	-4.739329000	-0.549093000	-0.015023000
1	-3.729419000	-2.883718000	-1.341842000
6	-3.864584000	-2.859176000	0.810341000
1	-3.750277000	-2.119770000	1.612977000
1	-3.213328000	-3.699328000	1.058148000
6	-5.308405000	-3.353770000	0.755629000
1	-5.997095000	-2.569817000	0.420936000
1	-5.403058000	-4.158819000	0.015698000
6	-5.794998000	-3.880188000	2.083413000
8	-5.164798000	-3.896961000	3.122614000
8	-7.062587000	-4.350670000	1.996663000
6	-4.385580000	-0.476513000	-2.133498000
1	-4.524318000	0.605158000	-2.262860000
1	-3.629670000	-0.779485000	-2.866254000
6	-5.705265000	-1.132033000	-2.476848000
8	-6.537864000	-1.513373000	-1.677681000
8	-5.883573000	-1.218498000	-3.812180000
1	-7.269338000	5.301381000	1.374991000
1	-6.765334000	-1.612112000	-3.961312000
1	-7.308339000	-4.669799000	2.886188000
1	4.634093000	-4.449770000	3.547772000
1	3.277392000	-0.646892000	3.810081000
